# Sirtuin 2 inhibits global protein synthesis via Rheb-GTPase degradation

**DOI:** 10.1038/s44319-026-00724-5

**Published:** 2026-03-11

**Authors:** Amarjeet Shrama, Yanlin Zi, Anwit Shriniwas Pandit, Kirtika Jha, Vikrant Kumar Sinha, Dimple Nagesh, Bhoomika Shivanaiah, Venkatraman Ravi, Souvik Ghosh, Danish Khan, Arathi Bangalore Prabhashankar, Thoniparambil Sunil Sumi, Satish Rajpurohit, Sunayana Ningaraju, Sukanya Raghu, Anand Srivastava, Mahavir Singh, Hening Lin, Nagalingam R Sundaresan

**Affiliations:** 1 Department of Microbiology and Cell Biology, Indian Institute of Science, Bengaluru, Karnataka 560012 India; 2 Department of Chemistry and Chemical Biology, Cornell University, Ithaca, NY 14853 USA; 3 Molecular Biophysics Unit, Indian Institute of Science, Bengaluru, Karnataka 560012 India; 4 Howard Hughes Medical Institute; Department of Medicine and Department of Chemistry, The University of Chicago, Chicago, IL 60637 USA

**Keywords:** SIRT2, Protein Synthesis, Rheb, Ubiquitination, Cardiac Hypertrophy, Post-translational Modifications & Proteolysis, Translation & Protein Quality

## Abstract

Increased global protein synthesis is associated with the development and progression of several aging-related diseases and disorders. Strategies like calorie restriction and pharmacological inhibition of protein synthesis have exhibited health-promoting effects. However, the complex molecular events that regulate global protein synthesis are not completely understood. Here, we report that SIRT2, a histone deacetylase, negatively regulates global protein synthesis by inhibiting the mTORC1 pathway via deacetylating Rheb and promoting its degradation. Our in vitro results suggest that SIRT2 deficiency increases protein synthesis, whereas SIRT2 overexpression suppresses protein synthesis. SIRT2-deficient mice exhibit increased global protein synthesis in the hearts, which may contribute to the development of cardiac hypertrophy. Conversely, cardiac-specific overexpression reduces global protein synthesis in the hearts of SIRT2 transgenic mice. Mechanistically, SIRT2 binds to and deacetylates Rheb at K151 residue to enhance ubiquitin-proteosome-mediated degradation of Rheb. Depletion of Rheb rescues increased protein synthesis in SIRT2-inhibited conditions. Our findings suggest that SIRT2 activation could be a potential therapeutic strategy for treating diseases associated with increased protein synthesis.

## Synopsis

SIRT2 inhibits global protein synthesis by deacetylating Rheb at Lys151, promoting its degradation and thereby attenuating Rheb–mTOR signalling.

- SIRT2 directly binds to Rheb-GTPase and deacetylates it at Lys151, promoting Rheb degradation.
- *Sirt2*-KO mice exhibit upregulated Rheb levels concomitant with increased mTOR activity and protein synthesis. *Sirt2*-KO mice develop age-associated cardiac hypertrophy.
- Rapamycin treatment attenuates the increase in global protein synthesis induced by SIRT2 inhibition.

## Abstract (web summary)

SIRT2 inhibits global protein synthesis by deacetylating Rheb at Lys151, promoting its degradation and thereby attenuating Rheb–mTOR signalling.

## Graphical abstract

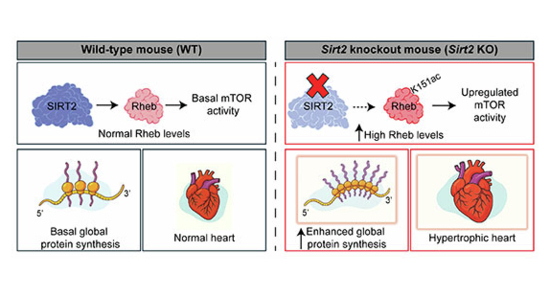

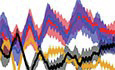

## Introduction

Protein synthesis and degradation are tightly regulated processes controlled by numerous regulators to maintain cellular proteostasis (Klaips et al, 2018). Protein synthesis is a highly energy-consuming process that competes with other cellular processes in terms of energy consumption (Princiotta et al, 2003). Dysregulation in protein synthesis is associated with diseases like cancer (Jia et al, 2023; Rubio et al, 2022), neurodegenerative diseases (Chartier-Harlin et al, 2011; Wiebe et al, 2020), and cardiac diseases (Liao et al, 2021; Wang et al, 2021). Protein synthesis is one of the key cellular processes regulated by aging-related pathways (Tavernarakis, 2008). Studies suggest that downregulation of protein synthesis can enhance the lifespan of model organisms (Syntichaki et al, 2007). A reduction in global protein synthesis downregulates the accumulation of damaged and misfolded proteins, thereby improving cellular health (Kikis et al, 2010). Protein synthesis activation largely correlates with nutrient availability and cellular energy state (Jewell and Guan, 2013). NAD^+^ levels provide an assessment of cellular energetic states, where high NAD^+^ levels typically indicate decreased cellular energy (Katsyuba et al, 2020). NAD^+^ and NAD^+^-dependent enzymes regulate multiple cellular processes and metabolic signaling pathways (Ying, 2006). Low levels of NAD^+^ are associated with the progression of various diseases, including cancers, metabolic disorders, cardiovascular disorders, and neurodegenerative diseases (Elhassan et al, 2017; Zapata-Pérez et al, 2021). Sirtuins, a group of NAD^+^-dependent deacetylase enzymes, act as cellular energy sensors by sensing high NAD^+^ levels and utilizing NAD^+^ to deacylate/deacetylate numerous proteins and regulate their functions (Massudi et al, 2012; Ottens et al, 2021). Seven isoforms of sirtuins (SIRT1-7) are reported in mammals; each has distinct subcellular localization and function (North and Verdin, 2004). Sirtuins regulate multiple cellular processes, including cellular metabolism, transcription, DNA repair, and protein synthesis (Michan and Sinclair, 2007). Previous findings have emphasized the involvement of SIRT1 (Shan et al, 2017), SIRT6 (Ravi et al, 2019), and SIRT7 (Tsai et al, 2014) in regulating global protein synthesis. However, the role of other sirtuins in this area remains unclear. SIRT2, a major cytoplasm-localized sirtuin, is involved in regulating various cellular events, including cytoskeleton organization, cellular metabolic activities, inflammation, and nuclear membrane dynamics (de Oliveira et al, 2012). However, as the only cytoplasmic sirtuin, where most of the translational machinery is present, the contribution of SIRT2 to regulating protein synthesis remains unexplored (Table 1).

**Table 1:** Oligonucleotide sequences used in this study.

Category	Target/gene	Application	Primers/siRNA	Sequence (5’–3’)	Reference
Genotyping	αMHC-Cre	Genotyping PCR	Forward primer	ATGACAGACAGATCCCTCCTATCTCC	This study
Genotyping	αMHC-Cre	Genotyping PCR	Reverse primer	CTCATCACTCGTTGCATCATCGAC	This study
SDM Confirmation	Rheb	PCR	Forward primer	CTACCGGTCTGTGGGGAAAT	This study
Mutagenesis	Rheb K151R	Site-directed mutagenesis	Mutagenic primer	ACAGCAGTCTGATTTTCTCTAGCAGAAGATTCCAAAAAAGCTG	This study
Mutagenesis	Rheb K151Q	Site-directed mutagenesis	Mutagenic primer	CATCCACAGCAGTCTGATTTTCCTGAGCAGAAGATTCCAAAAAAGCT	This study
Mutagenesis	SIRT2 N168A	Site-directed mutagenesis	Mutagenic primer	GCGCTGCTACACGCAGGCCATAGACACGCTGGAAC	PMID: 19037106
siRNA	SIRT2 (human)	Gene silencing	siRNA 1	GCCAACCAUCUGUCACUAC	This study
siRNA	SIRT2 (human)	Gene silencing	siRNA 2	GACUCCAAGAAGGCCUACA	This study
siRNA	Control	Negative control	siRNA	AAUUCUCCGAACGUGUCACGU	PMID: 31372634
qRT-PCR	mBNP	Expression analysis	Forward primer	AAGGGAGAATACGGCATCATTG	—
qRT-PCR	mBNP	Expression analysis	Reverse primer	ACAGCACCTTCAGGAGATCCA	—
qRT-PCR	mANP	Expression analysis	Forward primer	CCTGTGTACAGTGCGGTGTC	—
qRT-PCR	mANP	Expression analysis	Reverse primer	CCTCATCTTCTACCGGCATC	—
qRT-PCR	mActin	Expression analysis	Forward primer	TTCTACAATGAGCTGCGTGTG	—
qRT-PCR	mActin	Expression analysis	Reverse primer	GGGGTGTTGAAGGTCTCAAA	—
qRT-PCR	mbMHC	Expression analysis	Forward primer	CCGAGTCCCAGGTCAACAA	—
qRT-PCR	mbMHC	Expression analysis	Reverse primer	CTTCACGGGCACCCTTGGA	—
qRT-PCR	mRPL32	Expression analysis	Forward primer	ATCAGGCACCAGTCAGACCGAT	—
qRT-PCR	mRPL32	Expression analysis	Reverse primer	GTTGCTCCCATAACCGATGTTGG	—
qRT-PCR	rGAPDH	Expression analysis	Forward primer	TACCAGGGCTGCCTTCTCTTG	—
qRT-PCR	rGAPDH	Expression analysis	Reverse primer	ATCTCGCTCCTGGAAGATGGT	—

The mechanistic Target of Rapamycin (mTOR) is one of the master controllers of protein synthesis in cells (Saxton and Sabatini, 2017b). mTOR is a protein kinase that belongs to the PI3K-related kinase family and is the main constituent of two distinct protein complexes known as mTORC1 and mTORC2; these complexes have different functions, with mTORC1 governing global protein synthesis (Saxton and Sabatini, 2017b; Wullschleger et al, 2006). Ras homolog enriched in the brain (Rheb), a small GTPase of the Ras Superfamily, is crucial for mTORC1 activation (Long et al, 2005). In the absence of growth stimulation, Rheb remains bound with the TSC1/2 complex. TSC2 acts as Rheb GTPase-activating protein and induces intrinsic GTPase activity of Rheb to keep it inactive in Rheb-GDP form (Zhang et al, 2003). Upon receiving adequate growth-stimulatory signals, Akt-dependent multisite phosphorylation of TSC2 releases the TSC1/2 complex from Rheb, thereby leading to Rheb activation. Active Rheb in its GTP-bound form recruits mTORC1 on the lysosomal membrane and activates mTOR kinase activity within the mTORC1 complex (Garami et al, 2003; Long et al, 2005; Saxton and Sabatini, 2017a). Active mTORC1, with the help of its accessory subunit raptor, phosphorylates distinct mTORC1 downstream targets like S6 kinase and 4EBP1. This further results in increased protein synthesis (Morita et al, 2015; Saxton and Sabatini, 2017a), mainly due to increased translation of cap-dependent mRNAs (Dibble and Manning, 2013; Le Sage et al, 2016; Ma and Blenis, 2009).

Previous reports suggest that protein synthesis is upregulated during cardiac hypertrophy to meet the immense protein demand of the hypertrophic heart (Grund et al, 2019; Yan et al, 2021). The hypertrophic heart often exhibits enhanced activation of mTORC1 signaling, and inhibition of the mTORC1 pathway improves cardiac function in mouse models via reducing cardiac hypertrophy and fibrosis (Gao et al, 2006). Current knowledge about sirtuins provides insight into their possible roles in cardiac health (Ravi et al, 2021b). Our recent study on SIRT6 has elucidated the importance of SIRT6 in the regulation of protein synthesis in the context of cardiac hypertrophy (Ravi et al, 2019). Recent findings show that SIRT2 levels decrease in the cardiac tissues of aged mice, and *Sirt2*-KO mice develop aging-associated hypertrophy (Tang et al, 2017). Moreover, *Sirt2*-KO mice display increased sensitivity towards neurohormonal stress-induced cardiac hypertrophy and fibrosis (Sarikhani et al, 2018a; Tang et al, 2017). These recent reports emphasize the importance of understanding whether SIRT2 plays a role in regulating global protein synthesis.

In the current study, we found that SIRT2 plays a key role in regulating global protein synthesis within cells by deacetylating Rheb and facilitating its proteasome-mediated degradation. We have utilized the *Sirt2*-KO mice model to understand the significance of increased Rheb levels, mTORC1 activity, and upregulated global protein synthesis in the development of cardiac hypertrophy in *Sirt2*-KO mice hearts.

## Results

### SIRT2 negatively regulates global protein synthesis

**Figure 1:**
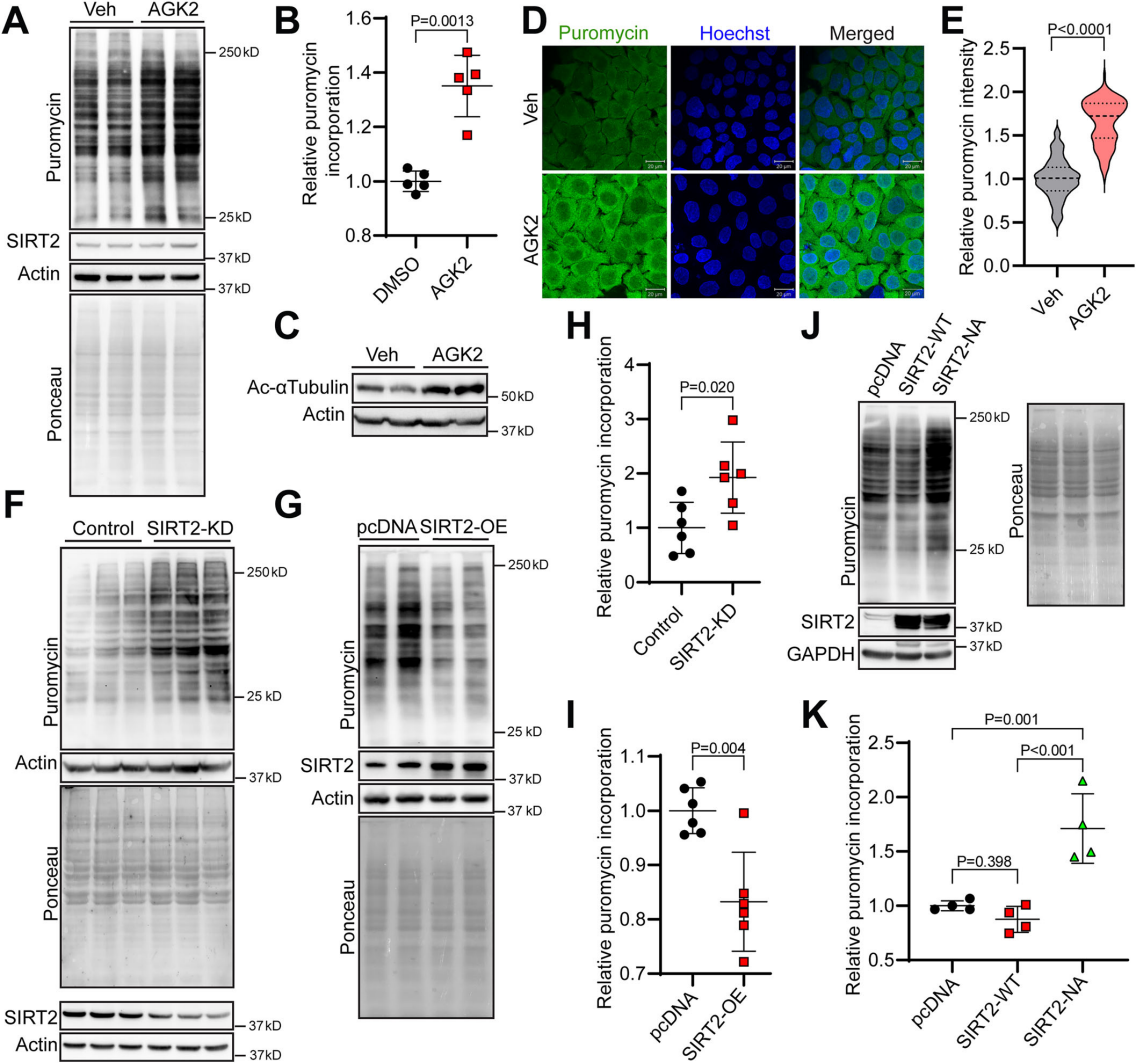
SIRT2 negatively regulates global protein synthesis. (**A**) Representative western blot images of SUnSET analysis depicting changes in protein synthesis rate in HeLa cells treated with DMSO or SIRT2 inhibitor AGK2. (**B**) Quantification of puromycin incorporation depicted in (**A**). The results are expressed as the fold change relative to DMSO-treated controls, *n* = 5. *P* values shown are from unpaired, two-tailed Student’s *t* test. Data are presented as mean ± s.d. (**C**) Representative western blot images showing changes in acetylated α-tubulin (Lysine 40) levels in HeLa cells after DMSO (Vehicle) and AGK2 treatment. (**D**) Representative images of IFC—SUnSET analysis in HeLa cells treated with vehicle (DMSO) and AGK2. Puromycin staining is shown in green. Nuclei are stained with Hoechst 33342 and shown in blue. (**E**) Quantitative representation of IFC-SUnSET analysis shown in (**D**), Puromycin intensity was normalized against vehicle, *n* = 90–100 cells per group. *P* values shown are from the Mann–Whitney test. Data are shown as median with 25 and 75 percentiles, *n* = 3, *N* = 108–110. (**F**) Representative images of western blotting SUnSET analysis depicting the changes in protein synthesis rate in control and SIRT2-depleted (SIRT2-KD) HeLa cells. SIRT2 was depleted using SIRT2-specific siRNA, and scrambled siRNA was used as a control. A separate SDS-PAGE was run, and SIRT2 knockdown was confirmed via immunoblotting. (**G**) Representative images of western blotting SUnSET analysis depicting the changes in protein synthesis rate in control and SIRT2 overexpressing HeLa cells. HeLa cells were transfected with either pcDNA (control) or SIRT2-WT plasmids, and SIRT2 overexpression was confirmed via immunoblotting. (**H**) Quantitative representation of puromycin incorporation depicted in (**F**). The values are shown as the fold change relative to scrambled siRNA-treated control groups. *n* = 6. *P* values shown are from unpaired, two-tailed Student’s *t* test. Data are presented as mean ± s.d. (**I**) Quantitative representation of puromycin incorporation depicted in (**G**). The results are expressed as the fold change relative to pcDNA-transfected control groups. *n* = 6. *P* values shown are from unpaired, two-tailed Student’s *t* test. Data are presented as mean ± s.d. (**J**) Representative images of western blotting SUnSET analysis in HeLa cells transfected with pcDNA, SIRT2-WT, or SIRT2-N168A plasmids. The plasmids were transfected for 48 h, and the expression of SIRT2 and its catalytic mutant was confirmed by western blotting. (**K**) Quantitative representation of puromycin incorporation depicted in (**J**). The results are expressed as the fold change relative to pcDNA-transfected control groups. *n* = 4. *P* values shown are from ordinary one-way ANOVA with Holm–Sidak’s multiple comparisons test. Data are presented as mean ±  s.d. Source data are available online for this figure.

**Figure EV1:**
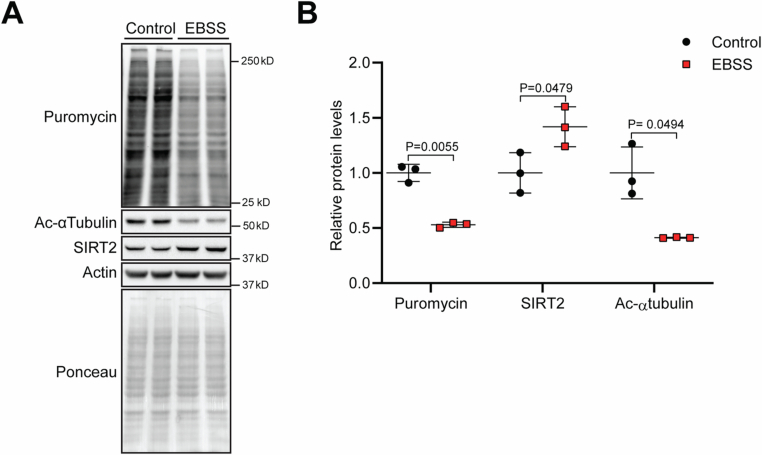
SIRT2 levels and activity are upregulated in nutrient-deprived conditions along with decreased protein synthesis rates. (**A**) Representative images of Western blotting analysis indicating the changes in protein synthesis rate (tracked using WB-SUnSET assay) and concomitant changes in the levels of acetyl-tubulin (Lysine 40) and SIRT2, in HeLa cells treated with high glucose DMEM media supplemented with 10% FBS (CM) and complete starvation using EBSS. (**B**) Scatterplot representing quantification of immunoblots from Fig. EV1A. The graph represents relative protein levels of puromycin-incorporated peptides, acetyl-tubulin, and SIRT2. *n* = 3. *P* values shown are from unpaired, two-tailed Student’s *t* tests (Unpaired *t* test with Welch’s correction), calculated separately for individual proteins. Data are presented as mean ± s.d. *n* = 3.

Our previous findings showed that SIRT2 levels are downregulated in the cardiac tissue of mice injected with cardiac stress-inducing drugs Isoproterenol (ISO) and Phenylephrine (PE) (Sarikhani et al, 2018a). The rate of protein synthesis is also reported to be upregulated in the heart of mice that develop ISO-induced hypertrophy (Sarikhani et al, 2018a). Therefore, we hypothesized that SIRT2 might have a role in regulating global protein synthesis. To determine if SIRT2 directly regulates protein synthesis, we inhibited SIRT2 using AGK2 (SIRT2 inhibitor). We observed that protein synthesis rates were significantly upregulated upon SIRT2 inhibition, as confirmed by both Western blotting and confocal microscopy-based assessments of puromycin incorporation under SIRT2 inhibition, suggesting a direct involvement of SIRT2 in regulating protein synthesis (Fig. 1A–E). We verified tubulin acetylation to confirm whether SIRT2 inhibition by AGK2 was effective (Fig. 1C). We observed a significantly enhanced rate of protein synthesis in cells that were transiently depleted for SIRT2 using SIRT2-specific siRNA (Fig. 1F,H). Since we observed that inhibiting SIRT2 activity or depleting its endogenous levels could significantly enhance the rate of protein synthesis, we further confirmed if SIRT2 overexpression could reduce protein synthesis. Transient overexpression of SIRT2 led to a significantly lower rate of protein synthesis (Fig. 1G,I). SIRT2 is known to remove acetylation (Kim et al, 2011) or long-chain acylation (Jing et al, 2017) from the lysine residues of its target proteins. We transiently overexpressed a catalytically inactive SIRT2 mutant (SIRT2 N168A) to investigate whether SIRT2-mediated regulation of protein synthesis depends on its catalytic activity. Interestingly, we observed that the rate of protein synthesis was significantly higher in cells overexpressing the SIRT2 N168A mutant than in cells expressing the pcDNA control (Fig. 1J,K). This observation suggests a possible dominant negative effect of the SIRT2 N168A mutant, as SIRT2 dimerization is crucial for SIRT2 deacetylase activity (Yang et al, 2023); previous studies have also reported the SIRT2 N168A mutant as a catalytically inactive dominant mutant (Li et al, 2008). We also investigated whether and how SIRT2 responds during complete cellular starvation, a state in which protein synthesis remains inhibited. We treated cells with EBSS (Earle’s Balanced Salt Solution) and observed that complete cell starvation increases SIRT2 levels and activity concomitant with decreased global protein synthesis (Fig. EV1A,B). These findings suggest that SIRT2 is a negative regulator of protein synthesis.

### SIRT2 affects protein synthesis by regulating mTOR kinase activity

**Figure 2:**
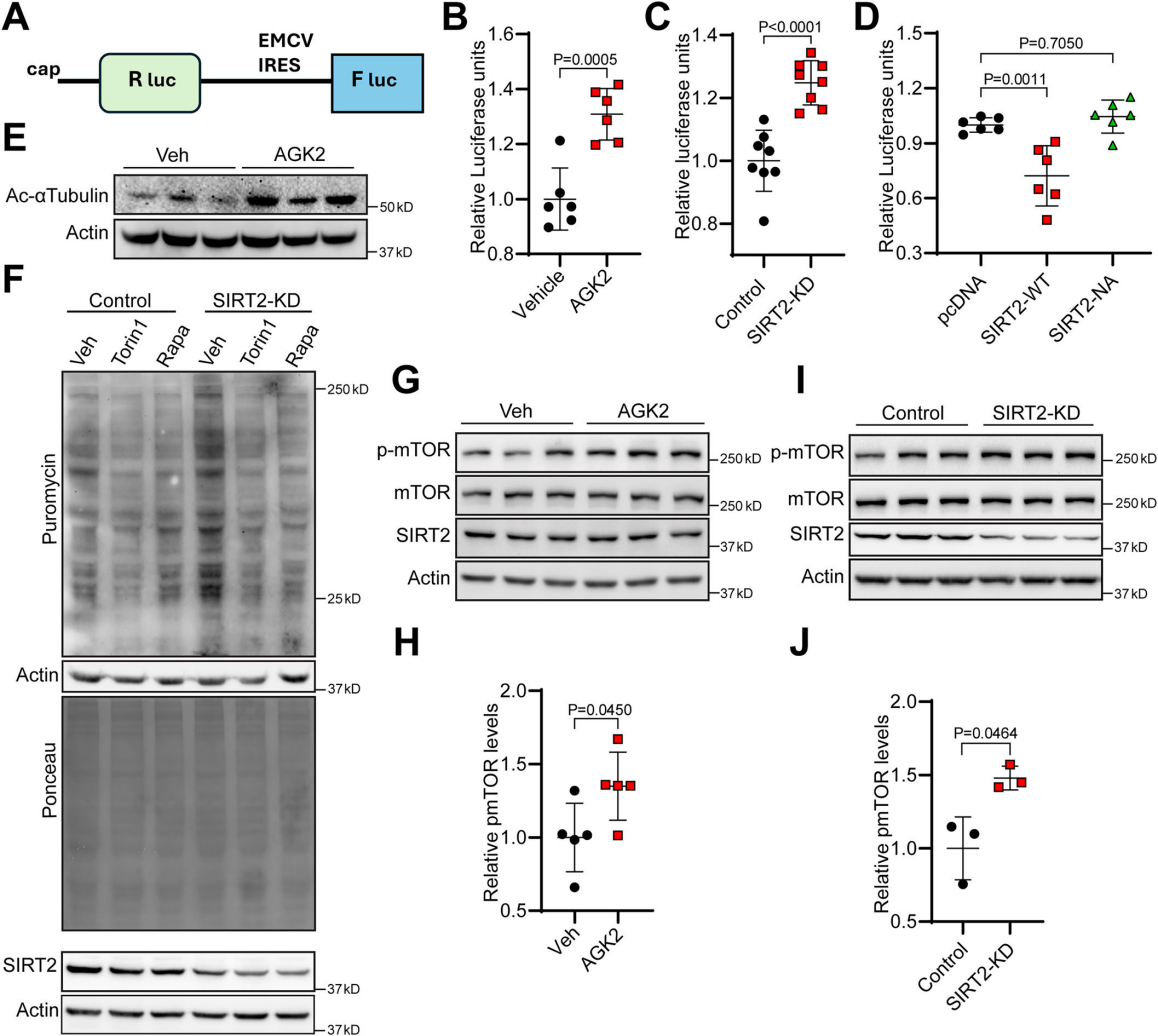
SIRT2 affects protein synthesis by regulating mTOR kinase activity. (**A**) Schematic representation of luciferase reporter EMCV-bicis construct for assessing cap-dependent translation. (**B**) Quantitative representation of cap-dependent translation measured using luciferase reporter EMCV-bicis construct in HeLa cells treated with either DMSO (Vehicle) or AGK2. The results are expressed as the fold change relative to control group. *P* values shown are from unpaired, two-tailed Student’s *t* test. Data are presented as mean ± s.d., *n* = 6. (**C**) Quantitative representation of cap-dependent translation measured using luciferase reporter EMCV-bicis construct in HeLa cells transfected with either Control or SIRT2-specific siRNA. The results are expressed as the fold change relative to the control group. *P* values shown are from unpaired, two-tailed Student’s *t* test. Data are presented as mean ± s.d., *n* = 8. (**D**) Quantitative representation of cap-dependent translation rates measured using luciferase reporter EMCV-bicis construct in control, SIRT2-WT, and SIRT2 N168A mutant overexpressing HeLa cells. The results are expressed as the fold change relative to the control group. *P* values shown are from ordinary one-way ANOVA with Dunnett’s multiple comparisons test. Data are presented as mean ± s.d., *n* = 6. (**E**) Representative images of western blotting analysis showing changes in Acetyl tubulin levels (Lysine 40) in HeLa cells after DMSO (Vehicle) and AGK2 treatment. (**F**) Representative images of Western blotting SUnSET analysis depicting the changes in protein synthesis rate in control and SIRT2-depleted (SIRT2-KD) HeLa cells treated with either vehicle or mTOR inhibitors Torin1 and Rapamycin. SIRT2 was depleted using SIRT2-specific siRNA, and Scrambled siRNA was used as a control. A separate SDS-PAGE was run, and SIRT2 knockdown was confirmed via immunoblotting. *n* = 3. (**G**) Representative western blotting images depicting changes in mTOR activation (mTOR phosphorylation at serine 2448) in HeLa cells treated with DMSO (Vehicle) or SIRT2 inhibitor AGK2. (**H**) Quantitative representation of mTOR activation depicted in (**G**). The results are expressed as the fold change relative to vehicle-treated controls. *P* values shown are from unpaired, two-tailed Student’s *t* test. Data are presented as mean ± s.d., *n* = 5. (**I**) Representative western blotting images depicting changes in mTOR activation (mTOR phosphorylation at serine 2448) in control and SIRT2-depleted HeLa cells. (**J**) Quantitative representation of mTOR activation depicted in (**I**). The results are expressed as the fold change relative to Scrambled siRNA-transfected controls. *P* values shown are from unpaired, two-tailed Student’s *t* test. Data are presented as mean ± s.d., *n* = 3. Source data are available online for this figure.

Most mRNAs in eukaryotic cells undergo translation through a process known as cap-dependent translation (Sonenberg and Hinnebusch, 2009). Since cap-dependent translation is the primary translational pathway in cells, we were interested in understanding whether SIRT2 regulates global protein synthesis by modulating the cap-dependent translation process. To test this, we utilized the EMCV-bicis construct, a dual-luciferase reporter vector that enables the measurement of cap-dependent and cap-independent translation separately. The Renilla/firefly ratio provides a measure of cap-dependent translation in cells (Choo et al, 2008; Dave et al, 2019) (Fig. 2A). Significant upregulation was observed in cap-dependent translation in cells upon SIRT2 inhibition using AGK2 (Fig. 2B). AGK2-mediated SIRT2 inhibition was confirmed by increased acetylation of α-tubulin (Fig. 2E). Furthermore, we also observed a significant increase in cell cap-dependent translation upon SIRT2 knockdown (Fig. 2C). We found a reduction in cap-dependent translation under SIRT2 overexpression, whereas overexpression of catalytically inactive mutant SIRT2 N168A did not show such a reduction in cap-dependent translation (Fig. 2D). Initiation of translation in a cap-dependent manner is the most extensively evaluated regulatory step, which depends on the eIF4F complex recognizing the 5’ cap region. This complex consists of cap-binding factor eIF4E, scaffold protein eIF4G, and helicase eIF4A (Gandin et al, 2022). Previous reports have shown that the assembly of the eIF4F complex is largely governed by mTORC1 (Gingras et al, 1999; Sonenberg and Hinnebusch, 2009). mTORC1 activation often correlates with the sufficiency of dietary intake, and its activation is inhibited during fasting or when an energy source is limited (Condon and Sabatini, 2019). Activated mTOR kinase phosphorylates downstream targets like S6 kinase and 4EBP1, which leads to an upregulation of protein synthesis, increasing mostly the translation of cap-dependent mRNAs (Le Sage et al, 2016). Previous studies have shown that phosphorylation of Ser-2448 at mTOR is dependent on mTOR kinase activity (Bonnet et al, 2024; Chiang and Abraham, 2005; Ochiai et al, 2024; Ravi et al, 2019). Anabolic factors such as insulin, nutrients, and amino acids can upregulate Ser-2448 phosphorylation (Bonnet et al, 2024; Chiang and Abraham, 2005). To understand whether SIRT2 regulates protein synthesis through the regulation of the mTOR pathway, we treated SIRT2-depleted cells with mTOR inhibitors, Torin1 and Rapamycin, and observed that cells treated with these inhibitors do not exhibit an increase in protein synthesis rate (Fig. 2F). In addition, concomitant with the previously observed increased cap-dependent translation, phosphorylation of mTOR at Ser-2448 was significantly upregulated upon SIRT2 inhibition (Fig. 2G,H). Increased p-mTOR levels were further observed under the SIRT2 knocked-down condition (Fig. 2I,J). These findings strongly indicate that SIRT2 regulates protein synthesis by regulating mTOR kinase activity.

### SIRT2 deacetylates Rheb GTPase and promotes its proteasomal degradation

**Figure 3:**
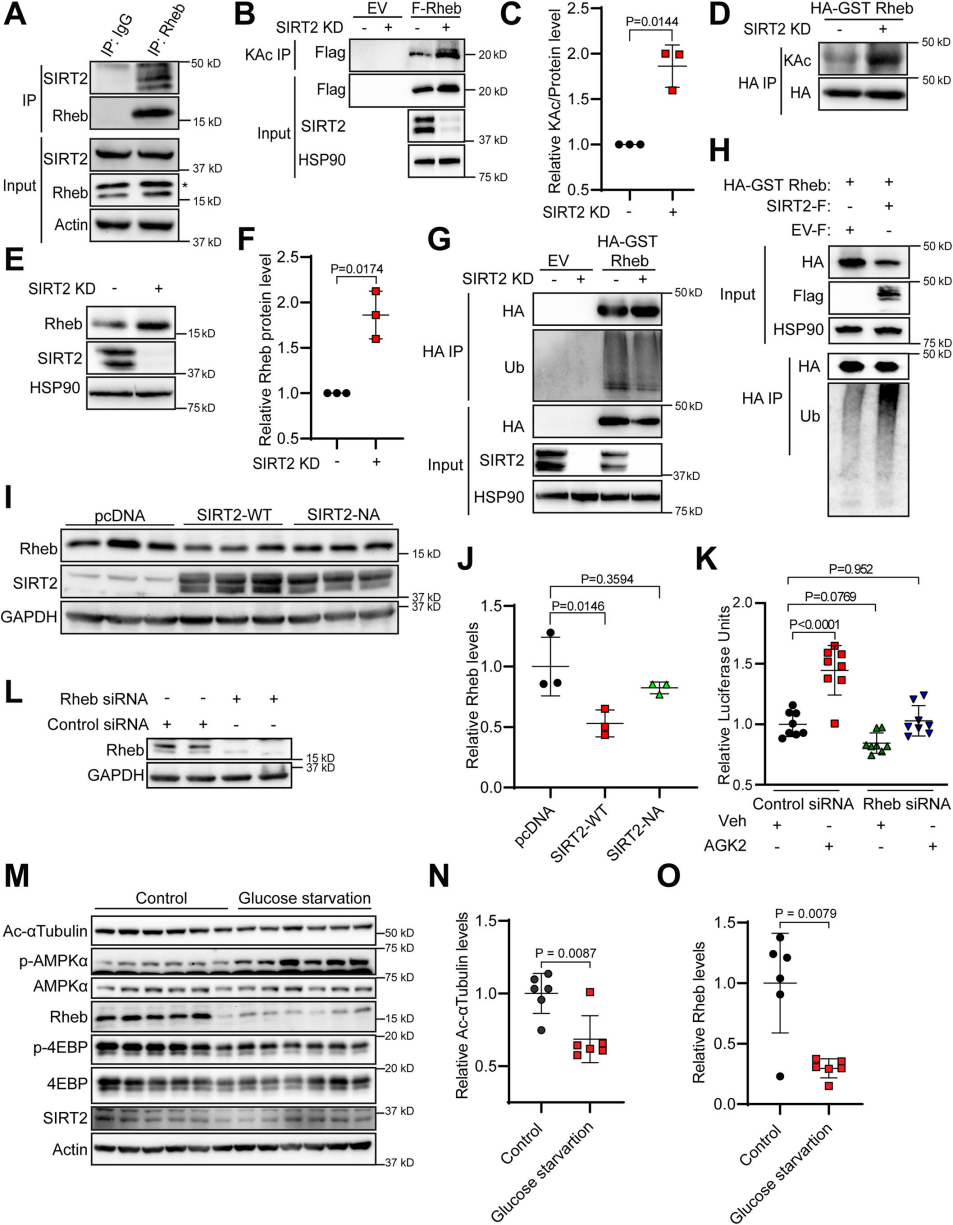
SIRT2 deacetylates Rheb GTPase and promotes its proteasomal degradation. (**A**) Representative immunoblot images showing the interaction of SIRT2 with Rheb in HeLa cells. Rheb was immunoprecipitated and probed for SIRT2. Whole cell lysate (WCL) was also immunoblotted to check the loading. *n* = 3, * indicates non-specific band. (**B**) Rheb is deacetylated by SIRT2. Flag-tagged Rheb or Flag-tagged empty vector was transfected into control and SIRT2 knockdown HEK293T cells. Acetylation was determined using acetyl lysine pull-down and western blot for Flag-Rheb. (**C**) Quantification of Rheb acetylation (normalized to protein level). *P* values shown are from ratio paired Student’s *t* test. Data are presented as mean ±  s.d., *n* = 3. (**D**) Rheb is deacetylated by SIRT2. HA-GST Rheb was transfected into control and SIRT2 knockdown HEK293T cells. After HA IP pull down, lysine acetyl antibody was used to detect acetylated Rheb in western blot. This blot was also used in Fig. 4E to check ubiquitination of Rheb WT and Rheb mutants under SIRT2 knockdown. (**E**) Endogenous Rheb protein level increases when SIRT2 is knocked down. Endogenous Rheb levels in control and SIRT2 knockdown HEK293T cells were evaluated using western blot. (**F**) Relative Rheb to total protein level ratios were quantified. *P* values shown are from ratio paired, two-tailed Student’s *t* test. Data are presented as mean ± s.d., *n* = 3. (**G**) Knockdown of SIRT2 decreases Rheb ubiquitination. HA-GST Rheb was expressed in control and SIRT2 knockdown HEK293T cells and was pulled down by HA beads. The ubiquitination level was analyzed using an anti-ubiquitin western blot. (**H**) Overexpression of SIRT2 increases Rheb ubiquitination. Flag-tagged SIRT2 or empty vector, along with HA-GST Rheb, were transfected into HEK293T cells. Rheb was pulled down by HA beads, and ubiquitination levels were analyzed using western blot. (**I**) Representative western blot images depicting changes in Rheb protein levels in control HeLa cells or cells overexpressing SIRT2-WT or SIRT2 N168A mutant. (**J**) Quantification of Rheb protein levels depicted in (**I**). The results are expressed as the fold change relative to the control group. *P* values shown are from the Kruskal-Wallis test for nonparametric data with Dunn’s multiple comparisons test. Data are presented as mean ± s.d., *n* = 3. (**K**) Quantification of cap-dependent translation measured using luciferase reporter EMCV-bicis construct in HeLa cells transfected with scrambled siRNA or Rheb siRNA and treated with either vehicle (DMSO) or AGK2. The results are expressed as the fold change relative to the control group. P values shown are from ordinary two-way ANOVA with Dunnett’s multiple comparisons test. Data are presented as mean ± s.d., *n* = 8. (**L**) Representative western blot images depicting changes in Rheb protein levels in HeLa cells transfected with scrambled siRNA or Rheb siRNA. (**M**) Representative western blot images depicting changes in levels of the proteins involved in the mTOR signaling pathway in neonatal rat cardiomyocytes under glucose-deprived conditions (p-AMPKα at Threonine 172, p-4EBP at Threonine 37/46). Acetylation of α-tubulin was assessed to evaluate SIRT2 activity. (**N**) Quantification of α-tubulin acetylation depicted in (**M**) (normalized with housekeeping control actin). The results are expressed as the fold change relative to the complete media control group. *P* values shown are from an unpaired, two-tailed Student’s *t* test. Data are presented as mean ± s.d., *n* = 6. (**O**) Quantitative representation showing changes in Rheb protein levels depicted in Fig. 3M (normalized with housekeeping control actin). The results are expressed as the fold change relative to the complete media control group. P values shown are from an unpaired, two-tailed Student’s *t* test. Data are presented as mean ± s.d., *n* = 6. Source data are available online for this figure.

**Figure EV2:**
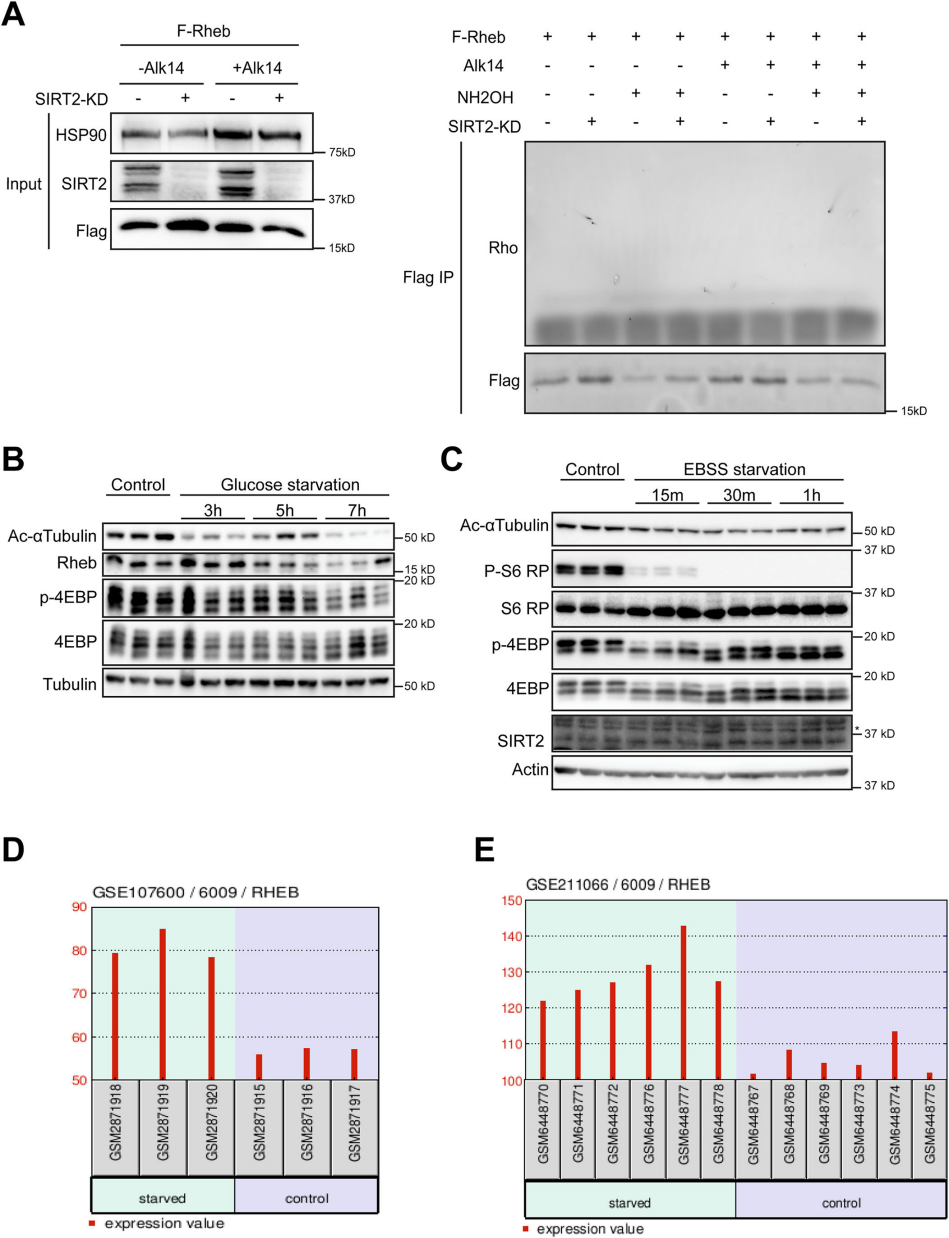
Rheb is not endogenously long-chain fatty acylated under normal conditions. (**A**) Alk14 metabolic labeling was used to detect Rheb palmitoylation. Flag-tagged RheB was overexpressed in control and SIRT2 knockdown HEK293T cells. Cells were labeled with Alk14, and samples were treated with or without hydroxylamine (which removes cysteine palmitoylation, but not lysine palmitoylation). Click chemistry was performed to conjugate a rhodamine-azide fluorescent label to proteins that are labeled with Alk14. Flag-Rheb was then pulled down, and its potential palmitoylation was detected by in-gel fluorescence. The lack of fluorescence signal indicates Rheb is not palmitoylated under the conditions tested. (**B**) Representative western blot images depicting changes in levels of Rheb protein in HEK293T cells under glucose-deprivation provided for 3, 5, 7 h. Acetylation of α-tubulin (Lysine 40) was assessed to evaluate SIRT2 activity. 4EBP phosphorylation (Threonine 37/46) was assessed to check mTORC1 activity, *n* = 3 independent experiments per group. (**C**) Representative western blot images depicting changes in levels of the proteins involved in the mTOR signaling pathway in neonatal rat cardiomyocytes under EBSS starvation conditions. Acetylation of α-tubulin (Lysine 40) was assessed to evaluate SIRT2 activity, phosphorylation of S6-RP at Serine 235/236, and phosphorylation of 4EBP at Threonine 37/46 was checked. *n* = 3 independent experiments per group. * indicates non-specific band. (**D**) Representative graph obtained from RNA-seq database (GSE107600/6009/ RHEB), depicting Rheb expression levels in Human HAP1 cells with 6-h EBSS starvation. (**E**) Representative graph obtained from RNA-seq database (GSE211066/6009/RHEB), depicting Rheb expression levels in HeLa cells with 4-h HBSS starvation.

SIRT2 primarily resides in the cytoplasm and regulates crucial cellular processes by post-translationally modifying its various substrate proteins. Since SIRT2 affects protein synthesis and mTOR activity, we were interested in identifying novel SIRT2 substrates that are part of the mTOR signaling pathway. Recent findings suggest that SIRT2 can deacylate multiple small GTPases inside cells to protect against Shigella infection, and Rheb GTPase is one of the substrates for SIRT2 deacylation during Shigella infection, where effector molecules from *Shigella* reportedly fatty acylate lysine residues of Rheb and other small GTPases (Wang et al, 2022). A recent study on the SIRT2 interactome in HeLa cells also suggests a possible interaction between SIRT2 and the Rheb protein (Eldridge et al, 2020). Therefore, we verified if SIRT2 could directly interact with Rheb and deacylate Rheb. Co-immunoprecipitation experiments demonstrated that endogenous Rheb interacts with SIRT2 even under basal conditions (Fig. 3A). Since SIRT2 was previously reported for removing Rheb’s lysine fatty acylation during *Shigella* infection, we were interested in studying whether SIRT2 could also mediate its regulation of mTOR signaling by removing the fatty acylation on Rheb protein. To achieve this, we first employed a click chemistry-based approach. Flag-tagged Rheb was overexpressed in control and SIRT2 knockdown (KD) HEK293T cells. Alk14 was used to label acylated proteins in cells, and the acylation level could be observed using in-gel fluorescence after click chemistry to conjugate a rhodamine fluorescent dye. Using this method, we found that Rheb was not acylated in either the control or the SIRT2 KD cells. This suggests that in a normal, healthy cellular environment, when there is no *Shigella* infection, the Rheb protein is not acylated (Fig. EV2A). Previous reports suggest a possible role of Rheb acetylation in mTORC1 activation in multiple cells, where acetylated FKBP12 interacts with acetylated Rheb and facilitates mTORC1 kinase activity (Hu et al, 2021). However, it remains unknown how Rheb acetylation is regulated and how the acetylation of Rheb promotes its ability to induce mTORC1 activation. Therefore, we hypothesize that SIRT2 may regulate Rheb-mediated mTORC1 activation by deacetylating Rheb.

To determine whether Rheb is a deacetylation substrate of SIRT2, we overexpressed Flag-tagged Rheb in control and SIRT2 KD HEK293T cells. Anti-acetyl lysine beads were used to pull down the acetylated proteins in the sample, and an anti-Flag western blot was used to analyze the acetylation level of Rheb. The acetylation level of Rheb increased in SIRT2 KD cells, indicating that SIRT2 could deacetylate Rheb (Fig. 3B,C). We also pulled down Rheb and detected its acetylation level using an anti-acetyl lysine immunoblotting. Again, Rheb was more acetylated in SIRT2 KD cells than in control cells (Fig. 3D). Thus, our data support the notion that Rheb is a substrate for deacetylation by SIRT2. Interestingly, total protein levels of Rheb were also significantly upregulated in SIRT2 KD cells (Fig. 3E,F). This observation suggests a possible role of SIRT2 in regulating Rheb’s stability. Multiple studies have shown that lysine acetylation can promote protein stability and decrease protein ubiquitination (Buchwald et al, 2009; Inuzuka et al, 2012; Nihira et al, 2017; Shimizu et al, 2021). To investigate whether lysine acetylation of Rheb facilitates its stability, we examined Rheb ubiquitination in control and SIRT2-knockdown cells. In line with increased Rheb protein levels, we observed that SIRT2 KD cells exhibit decreased ubiquitination of Rheb compared to control cells (Fig. 3G). Furthermore, Rheb ubiquitination increased upon SIRT2 overexpression (Fig. 3H). Overexpression of wild-type SIRT2 led to decreased Rheb protein levels, whereas overexpression of SIRT2 N168A mutant did not (Fig. 3I,J). In addition, we report that reducing Rheb levels can reverse the increase in cap-dependent translation induced by SIRT2 inhibition. (Fig. 3K,L). During cellular stress, particularly in the context of nutrient deprivation, translation is tightly controlled through multiple interconnected layers. Canonical upstream nutrient sensors, such as AMPK and TSC1/2, inhibit mTORC1 signaling, reducing the phosphorylation of key effectors (e.g., S6K, 4EBP1) and lowering protein synthesis (Gwinn et al, 2008; Tee et al, 2003). Given SIRT2’s dependence on NAD⁺, its activity is linked to the cellular metabolic state (Wang et al, 2007), providing a sensor mechanism that couples energy metabolism to translational control. Moreover, starvation triggers the dephosphorylation and activation of SIRT2 in a cyclin E/CDK2 suppression-dependent manner, inducing autophagy, thereby linking cellular energy status to SIRT2 activation (Sun et al, 2022). We were interested in understanding whether SIRT2-mediated Rheb regulation is relevant during physiological stress in cells. Recently, it was reported that Rheb undergoes K48-linked polyubiquitination under low glucose availability, leading to the inactivation of mTORC1 (Li et al, 2024), suggesting the essentiality of regulating Rheb levels and activity during glucose starvation. To investigate whether SIRT2 levels or activity are affected during glucose starvation, we subjected different cell types to glucose deprivation and assessed Rheb levels, SIRT2 levels, and acetylated α-tubulin levels (as an indicator of SIRT2 activity). Our findings reveal a significant downregulation of Rheb protein levels in primary cardiomyocytes isolated from neonatal rat hearts (NRCMs) following glucose deprivation (Fig. 3M,O). Although we did not observe changes in SIRT2 protein levels, SIRT2 activity was significantly upregulated in neonatal rat cardiomyocytes (NRCMs) subjected to glucose deprivation (Fig. 3M,N). Glucose deprivation was validated by increased phosphorylation of AMPK in cardiomyocytes cultured in glucose-free media (Fig. 3M). Moreover, similar findings were observed in HEK293T cells, indicating a conserved response across different cell types (Fig. EV2B). In addition, we assessed previously published RNA-seq data to check if Rheb is transcriptionally regulated under nutrient starvation and found no significant changes in Rheb expression under 6 h of EBSS starvation in Human HAP1 cells (GSE107600) and 4 h of HBSS starvation in  HeLa cells (GSE211066) (Fig. EV2D,E), suggesting that Rheb regulation during starvation does not occur at transcription levels. These findings suggest that SIRT2 deacetylates Rheb, promoting its ubiquitination and subsequent degradation.

### The lysine 151 residue in Rheb is the site for SIRT2-mediated deacetylation, and acetylation of lysine 151 stabilizes the Rheb

**Figure 4:**
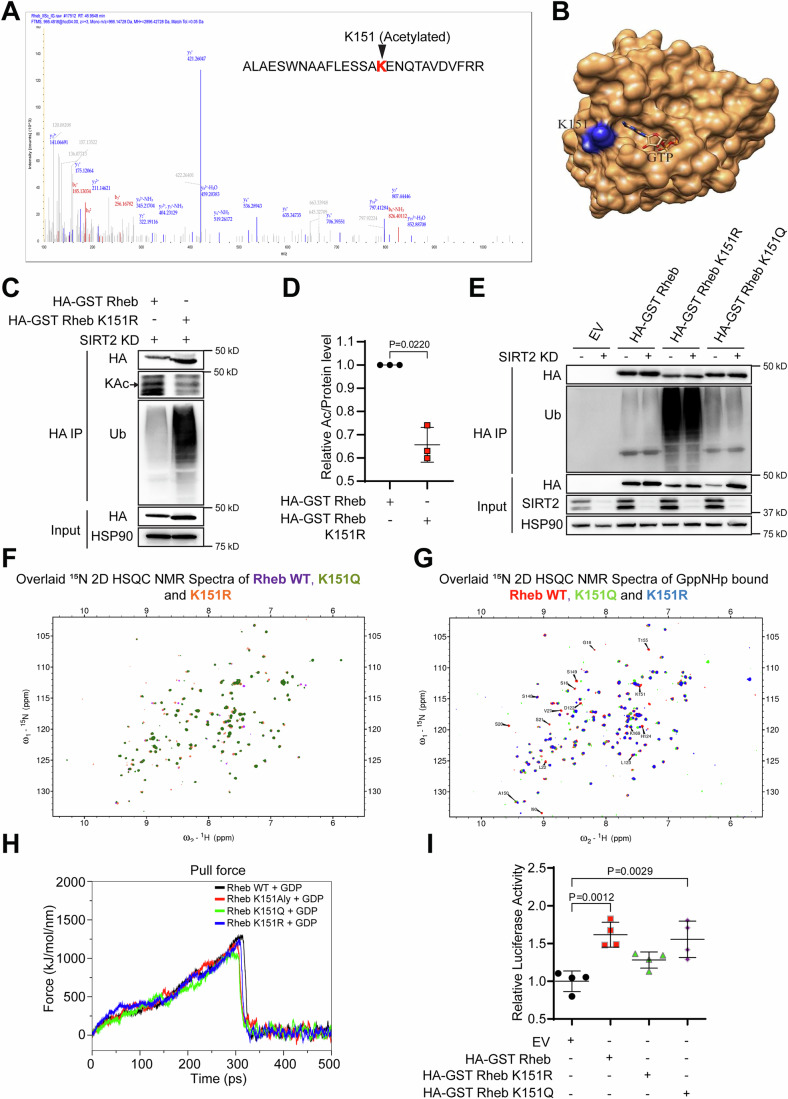
The lysine 151 residue in the Rheb is the site for SIRT2-mediated deacetylation, and acetylation of lysine 151 stabilizes the Rheb. (**A**) LC/MS spectra depicting acetylation of lysine residue 151 (K151) in Rheb protein under AGK2 treatment in HeLa cells. (**B**) 3D structure of GTP-bound Rheb depicting the position of K151 residue. (**C**) SIRT2 deacetylates Rheb at K151, which in turn increases Rheb’s ubiquitination. HA-GST Rheb or HA-GST Rheb K151R were transfected into SIRT2 knockdown HEK293T cells and pulled down with HA IP beads. Acetylation and ubiquitination were detected using western blots. (**D**) Relative Rheb acetylation levels (normalized to Rheb protein levels) were quantified and compared between HA-GST Rheb and HA-GST Rheb K151R in SIRT2 knockdown HEK293T cells. *P* values shown are from a ratio paired, two-tailed Student’s *t* test. Data are presented as mean ± s.d., *n* = 3. (**E**) Deacetylation of Rheb at K151 by SIRT2 increases ubiquitination. HA-GST Rheb, HA-GST Rheb K151R, and HA-GST Rheb K151Q were transfected into control or SIRT2 knockdown HEK293T cells. The ubiquitination level of Rheb was analyzed by HA IP, and western blot. (**F**) Representative overlaid 2D ^1^H-^15^N HSQC NMR spectra of Rheb WT, K151Q, and K151R Rheb mutants. (**G**) Representative overlaid 2D ^1^H-^15^N HSQC NMR spectra of GppNHp-bound Rheb WT, K151Q, and K151R Rheb mutants. (**H**) Graphical depiction of Pulling force simulation analysis showing the strength of GDP association with Rheb WT, Rheb K151Aly, and K151R, K151Q Rheb mutants. (**I**) Quantitative representation of cap-dependent translation measured using luciferase reporter EMCV-bicis construct in HeLa cells overexpressing HA-GST Rheb, HA-GST Rheb K151R, and HA-GST Rheb K151Q mutants. The results are expressed as the fold change relative to the Empty vector (EV) transfected control group. *P* values shown are from ordinary one-way ANOVA with Tukey’s multiple comparisons test. Data are presented as mean ± s.d., *n* = 4. Source data are available online for this figure.

**Figure EV3:**
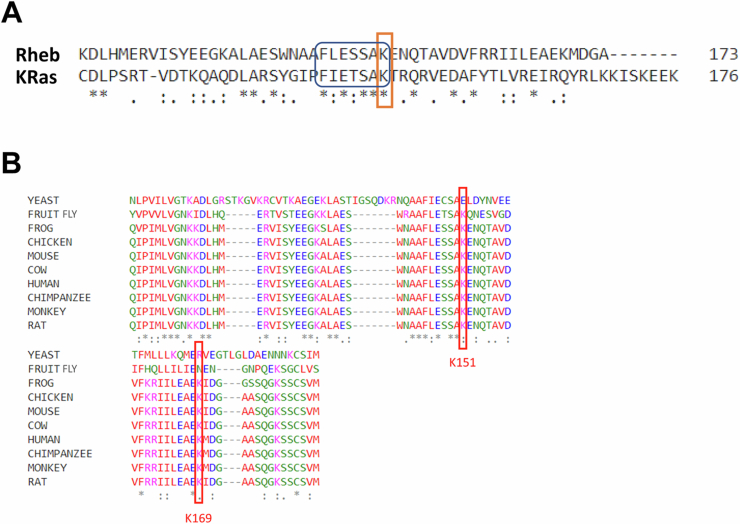
Lysine 151 is a conserved residue across different species; Rheb and KRas share a common sequence stretch for SIRT2-targeted Lysine residue. (**A**) Representative image showing sequence similarity in SIRT2 targeted region of Rheb and KRas, Rheb and KRas share a similar sequence stretch of amino acids for K151 and K147 residues, respectively. (**B**) Sequence alignment data depicting K151 residue in Rheb is conserved across different species.

To mark the lysine residues in Rheb that are targeted by SIRT2 for deacetylation, we performed a mass-spectrometric analysis in cells treated with AGK2 (SIRT2 inhibitor). Our mass spectrometry data reveal lysine 151 (K151) of Rheb protein as a possible acetylated residue under SIRT2 inhibition, suggesting that SIRT2 may deacetylate Rheb at the K151 position (Fig. 4A). Previous reports suggest that SIRT2 regulates the K147 acetylation status of a small GTPase, KRas (Song et al, 2016). Interestingly, Rheb and KRas share a similar sequence stretch of amino acids for the K151 and K147 residues, respectively (Fig. EV3A). Moreover, our sequence alignment data suggest that the lysine residue at position K151 is conserved across different species (Fig. EV3B). Structural analysis of Rheb Shows that K151 is located near the GTP-binding pocket of Rheb (Fig. 4B). To understand the structural and functional significance of K151 acetylation, we performed site-directed mutagenesis in the pRK5 HA-GST Rheb construct to generate Rheb K151R (mimicking the deacetylated form) and Rheb K151Q (mimicking the acetylated form) mutants of Rheb. HA-GST Rheb (Rheb WT) and Rheb K151R were expressed in SIRT2 KD cells and were pulled down using anti-HA beads. Acetylation blot showed a consistent and significant decrease in acetylation levels of Rheb K151R compared to Rheb WT, confirming that SIRT2 deacetylates Rheb at K151 (Fig. 4C,D). Moreover, when the western blot of the same samples was blotted with an anti-ubiquitin antibody, increased ubiquitination was observed in the K151R mutant (Fig. 4C). To further confirm that deacetylation was indeed responsible for the increase in Rheb ubiquitination, we overexpressed Rheb WT, K151R mutant, or K151Q mutant in control and SIRT2 knockdown cells, consistent with previous observations. The K151R mutant exhibited significantly increased Rheb ubiquitination, while the K151Q mutant, which mimics the acetylated form of Rheb, did not increase Rheb ubiquitination (Fig. 4E). Our data confirmed that SIRT2 deacetylates Rheb at K151, promoting Rheb ubiquitination and degradation.

**Figure EV4:**
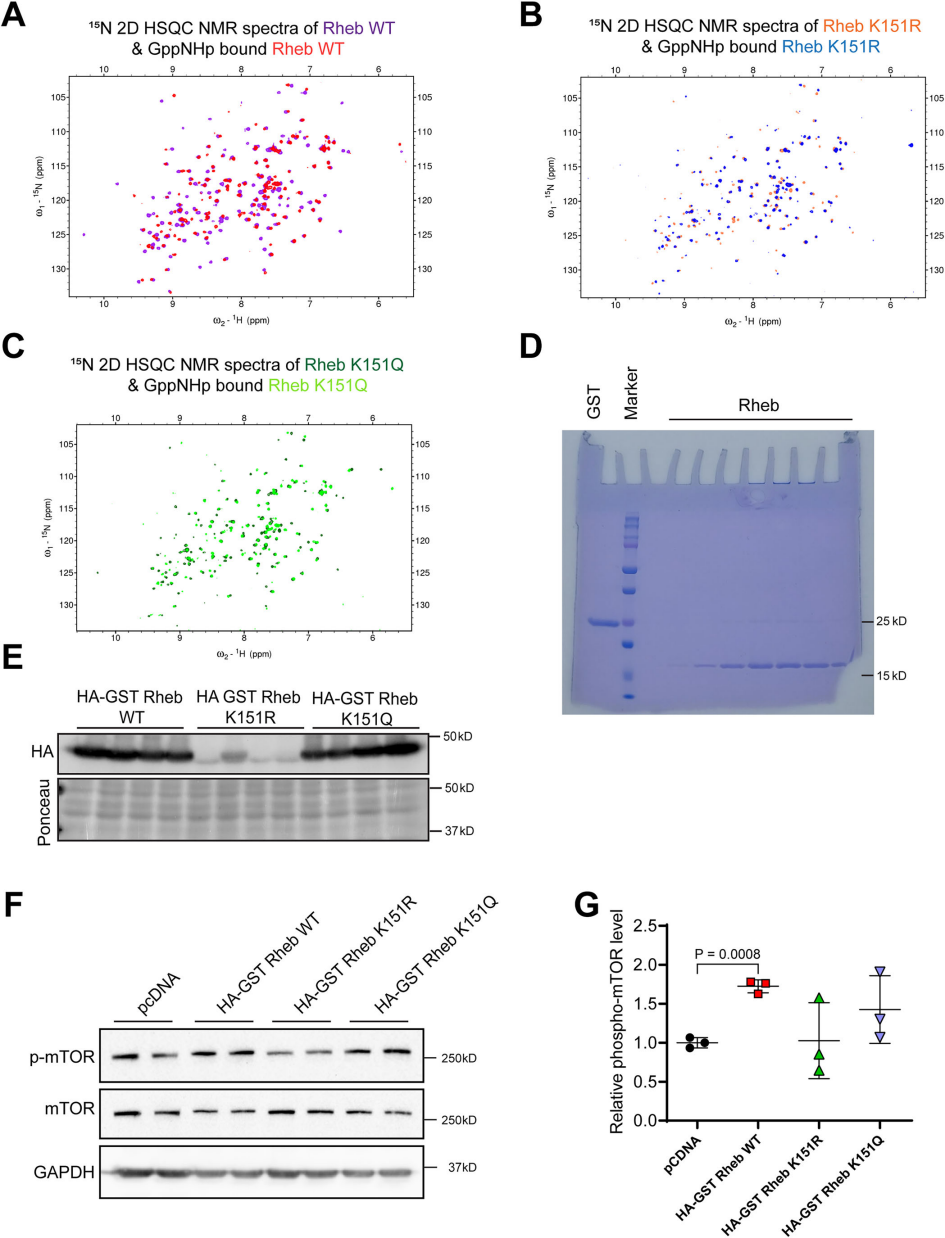
Rheb mutants K151R and K151Q are structurally stable in basal and GppNHp-bound form. (**A**) Representative overlaid 2D ^1^H-^15^N HSQC NMR spectra of Rheb WT & GppNHp bound Rheb WT. (**B**) Representative overlaid 2D ^1^H-^15^N HSQC NMR spectra of Rheb K151R & GppNHp bound Rheb K151R. (**C**) Representative overlaid 2D ^1^H-^15^N HSQC NMR spectra of Rheb K151Q & GppNHp-bound Rheb K151Q. (**D**) Representative SDS-PAGE gel image showing purified Rheb protein at 17 kD molecular weight. (**E**) Representative images of western blot analysis showing differential expression of HA-GST Rheb, HA-GST Rheb K151R, and HA-GST Rheb K151Q. Ponceau of the same blot is used as a loading control. (**F**) Representative western blotting images depicting changes in mTOR phosphorylation at Serine 2448 in HeLa cells transfected with pcDNA, HA-GST Rheb WT, HA-GST Rheb K151R, and HA-GST Rheb K151Q, *n* = 3. (**G**) Quantification of mTOR phosphorylation depicted in Figure EV4F. The results are expressed as the fold change relative to pcDNA control. *P* values shown are from the one-way ANOVA test with Dunnett’s T3 multiple comparisons. Data are presented as mean ± s.d., *n* = 3.

We used NMR spectroscopy to characterize the structural significance of these mutants. 2D ^1^H-^15^N HSQC NMR spectra were recorded for Rheb WT, Rheb K151Q, and Rheb K151R in basal and non-hydrolyzable GTP analog, GppNHp, bound form. Our analysis showed that K151R or K151Q mutation does not affect the overall structure or the GTP binding ability of Rheb (Figs. 4F,G and EV4A–D). GDP dissociation inhibitors are a class of factors that inhibit GDP–GTP exchange and regulate the activity of multiple small GTPases (Collins, 2003; DerMardirossian and Bokoch, 2005; Han et al, 2021). To understand if acetylation of Rheb at K151 has any role in GDP release from Rheb, we performed pulling force simulations to investigate the GDP binding strength in Rheb WT, Rheb K151acetylated, and mutants (K151R, K151Q). We did not observe any significant difference in the pulling force required to remove GDP among these groups (Fig. 4H), suggesting that acetylation of Rheb at K151 might not affect GTP binding and GDP dissociation. Next, we assessed the ability of these mutants to induce cap-dependent translation. We observed a significant increase in cap-dependent translation in cells transfected with Rheb WT and the K151Q mutant compared with the control group, whereas overexpression of the K151R mutant did not induce cap-dependent translation (Figs. 4I and EV4E). Additionally, overexpressing the K151R mutant did not induce mTOR phosphorylation, whereas we observed significant upregulation of mTOR phosphorylation when Rheb WT was overexpressed (Fig. EV4F,G). These findings suggest that SIRT2 deacetylates Rheb by targeting the K151 residue, facilitating Rheb ubiquitination and degradation, and thereby inhibiting Rheb-mediated mTORC1 activation.

### Acetylation of Rheb at K151 does not affect its membrane binding

**Figure 5:**
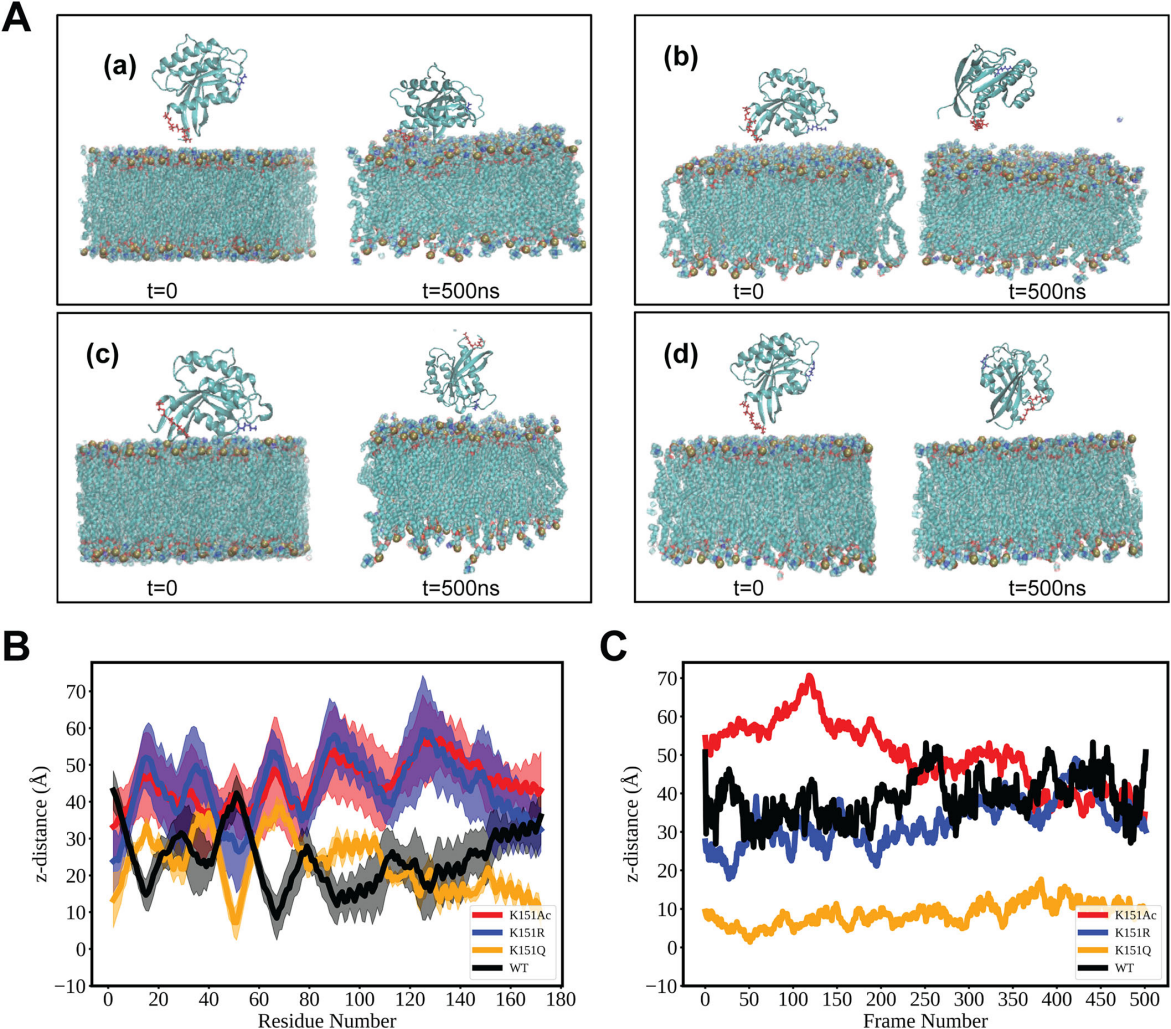
Molecular Simulation for studying the effect of acetylation upon the C-terminal farnesylated Rheb protein on membrane association. (**A**) Schematic diagram representing Rheb-bilayer orientations in different protein states considered for the study. initial at *t* = 0 and final at *t* = 500 ns. (a) WT Rheb with bilayer, (b) K151Acetylated with bilayer, (c) K151Q as acetylated mimic with bilayer, and (d) K151R as deacetylated mimic, respectively. Rheb protein has been shown in a cartoon representation, and K151 residue has been marked in bonds represented in blue color. The Farnesyl anchor is shown in red. Membrane phosphate residues have been shown in vdw representation. Water and ions have not been shown for clarity of image. (**B**) Graphical diagram representing the z-distance between the Cα of each residue and the phosphate plane of the bilayer. (**C**) Graphical diagram representing z-distance between 172 residue and the phosphate plane of the bilayer.

We performed all-atom molecular dynamics simulations to understand the effect of C-terminal farnesylation for wild-type as well as mutations that serve as acetylated or deacetylated mimics on membrane association. To gain molecular insights, protein-bilayer systems have been simulated. Four different systems, i.e., (a) WT Rheb with bilayer, (b) K151Acetylated with bilayer, (c) K151Q as an acetylated mimic with bilayer, and (d) K151R as a deacetylated mimic with bilayer, were set up. To cancel any bias generated by starting orientation, all four systems were initialized with the same initial orientation of the protein on the bilayer (Fig. 5A). For mimicking the membrane in simulation, a bilayer composition of 320 POPC and 96 POPS, in a ratio of 80:20, was used. Simulation outputs were analyzed by a simple Z-distance calculation between the Cα atoms of each residue with the z-coordinate of the phosphate plane of the bilayer. If the z-distance is less than zero, it means membrane association and vice versa. The analysis was performed over 200 snapshots from the last 20 ns run. Interestingly, there was no significant effect of the mutation/acetylation mimic on membrane association (Fig. 5B), though traces of membrane association were observed in the K151Q-bilayer simulations. Moreover, to confirm the membrane association, we have calculated the z-distance between the side chain atom of the 172nd residue and the phosphate plane (Fig. 5C). It clearly shows that the association is quite transient. Thereby, confirming no significant impact of this acetylation upon membrane association.

### *Sirt2*-KO mice hearts exhibit hyperactivated mTOR kinase activity

**Figure 6:**
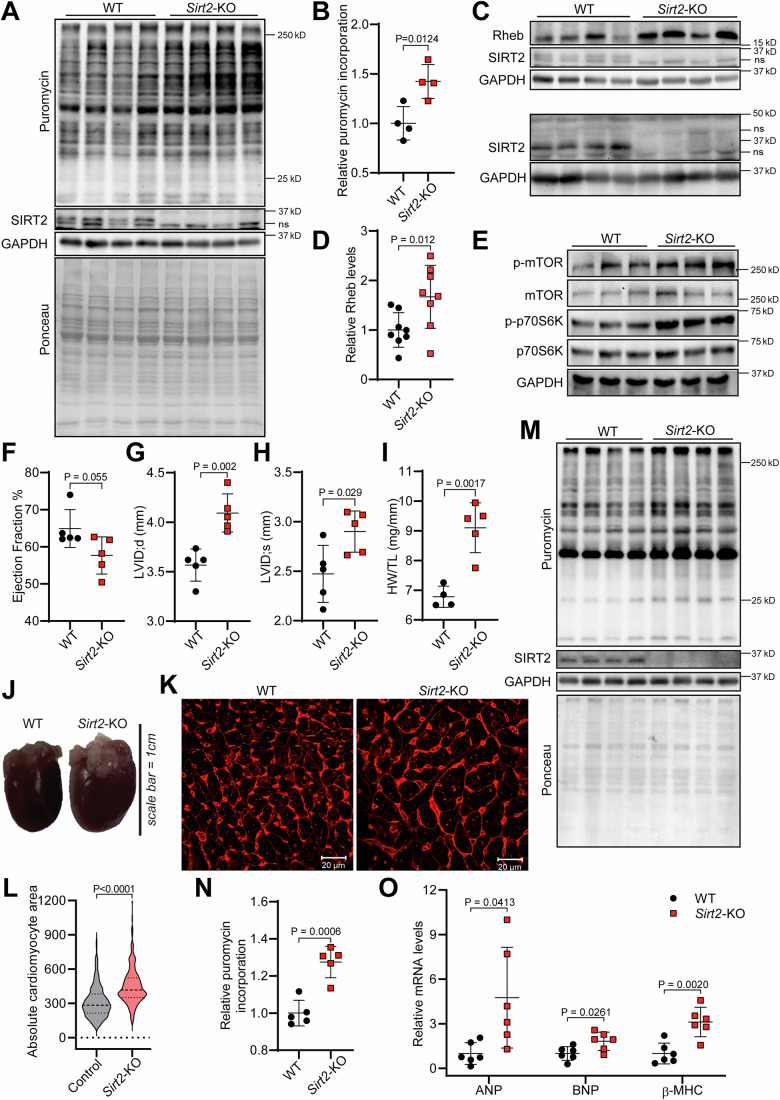
*Sirt2*-KO mice hearts exhibit hyperactivated mTOR kinase activity concomitant with an increased rate of protein synthesis. (**A**) Representative western blot images of SUnSET analysis depicting changes in protein synthesis rate in the heart tissues of 4-month-old *Sirt2*-KO and WT mice. ns indicates non-specific band. (**B**) Quantification of puromycin incorporation depicted in (**A**). The results are expressed as the fold change relative to WT controls. *P* values shown are from an unpaired, two-tailed Student’s *t* test. Data are presented as mean ± s.d., *n* = 4 mice per group. (**C**) Representative western blot images depicting changes in Rheb protein levels in heart tissues of 4-month-old *Sirt2*-KO and WT mice. A separate SDS-PAGE was run for an extended time and immunoblotted for SIRT2 to reveal distinct SIRT2 bands with improved resolution. ns indicates non-specific band. (**D**) Quantification of Rheb levels depicted in (**C**). The results are expressed as the fold change relative to WT controls. *P* values shown are from an unpaired, two-tailed Student’s *t* test. Data are presented as mean ± s.d., *n* = 4 mice per group. (**E**) Representative western blot images of analysis of mTOR signaling in the heart tissues of 4-month-old *Sirt2*-KO and WT mice. (mTOR phosphorylation at Serine 2448, p70S6K phosphorylation at Threonine 289). (**F**) A representative graph of echocardiographic analysis showing changes in the ejection fraction of 9-month-old *Sirt2*-KO mice when compared with age-matched wild-type mice controls. *P* values shown are from an unpaired, two-tailed Student’s *t* test. Data are presented as mean ± s.d., *n* = 5 mice per group. (**G**) A representative graph of echocardiographic analysis showing changes in Left ventricular internal diameter, diastolic (LVID; d), of 9-month-old *Sirt2*-KO mice when compared with age-matched controls. *P* values shown are from an unpaired, two-tailed Student’s *t* test. Data are presented as mean ± s.d., *n* = 5 mice per group. (**H**) Representative graph of echocardiographic analysis showing changes in Left ventricular internal diameter, systolic (LVID; s) of 9-month-old *Sirt2*-KO mice when compared with age-matched controls. *P* values shown are from an unpaired, two-tailed Student’s *t* test. Data are presented as mean ± s.d., *n* = 5 mice per group. (**I**) Scatterplot representing the heart weight of 9-month-old *Sirt2*-KO and WT mice; the heart weight is normalized with the respective mice’s tibia length. The results are expressed as the fold change relative to WT controls. *P* values shown are from an unpaired, two-tailed Student’s *t* test. Data are presented as mean ± s.d., *n* = 4–5 mice per group. (**J**) Representative images of the mouse hearts showing the difference between the heart size of WT and *Sirt2*-KO mice. (**K**) Representative confocal images of WGA-stained cardiac tissue sections showing the cross-sectional area of cardiac cells in WT and *Sirt2*-KO mice at 9 months of age. Scale bar = 20 μm. (**L**) Violin plot showing quantification of WGA images represented in (**K**). *P* values shown are from unpaired, two-tailed *t* test. *P* values shown are from the Mann–Whitney test. data are shown as median with 25 and 75 percentiles. *n* = 4, *N* = 217–228. (**M**) Representative images of western blotting SUnSET analysis depicting changes in protein synthesis rate in heart tissues of 9-month-old *Sirt2*-KO mice and age-matched wild-type control, *n* = 5 mice per group. (**N**) Quantification of puromycin incorporation is depicted in (**M**). The results are expressed as the fold change relative to WT controls. *P* values shown are from an unpaired, two-tailed Student’s *t* test. Data are presented as mean ± s.d., *n* = 5 mice per group. (**O**) qPCR analysis of relative mRNA expression levels of hypertrophy marker mRNAs in WT and *Sirt2*-KO mice heart tissues, normalized with Actin. *P* values shown are from unpaired, two-tailed Student’s *t* tests calculated separately for individual markers. Data are presented as mean ± s.d., *n* = 6 mice per group. Source data are available online for this figure.

**Figure EV5:**
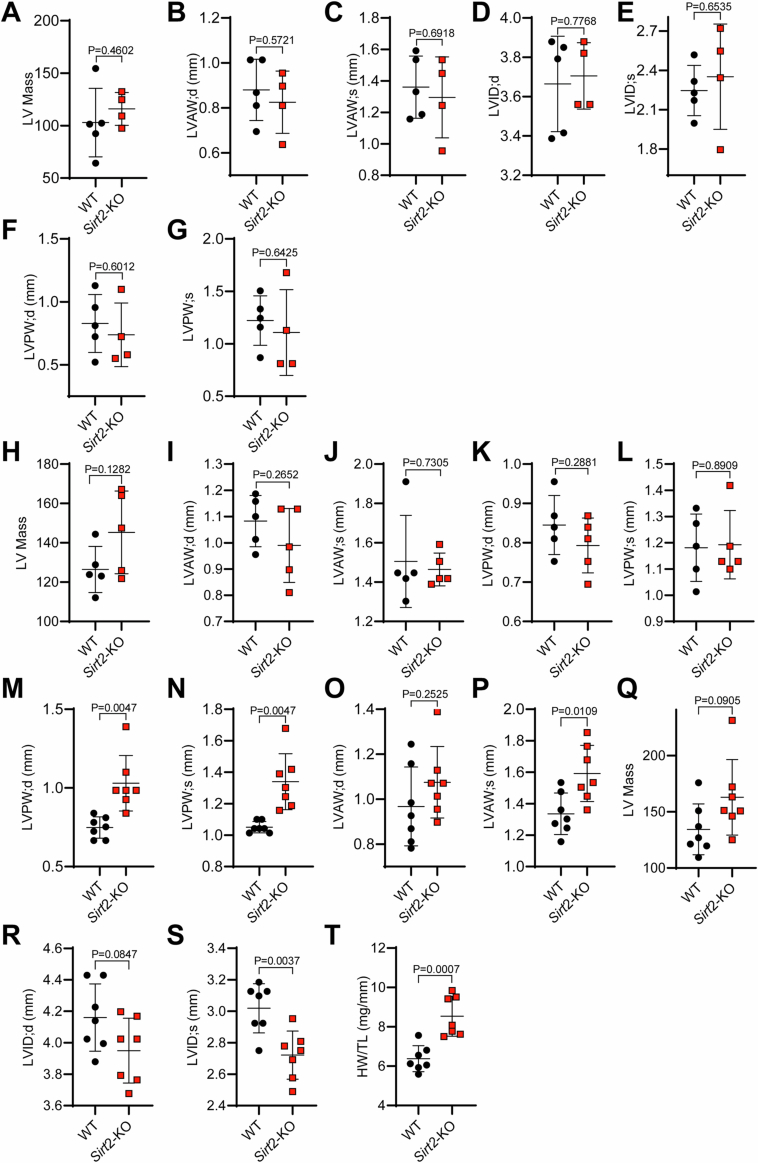
Representative graph of echocardiographic analysis showing changes in the various cardiac parameters of 4, 9, and 12-months-old *Sirt2-KO* mice. (**A**–**G**) Representative graph of echocardiographic analysis showing changes in the various cardiac parameters of 4-month-old *Sirt2*-KO mice when compared with age-matched wild-type mice controls. Data are presented as mean ± s.d., *n* = 4–5 mice per group. *P* values shown are from unpaired, 2-tailed Student’s *t* tests. Data are shown as mean ± s.d. (**H**–**L**) Representative graph of echocardiographic analysis showing changes in the various cardiac parameters of 9-month-old *Sirt2*-KO mice when compared with age-matched wild-type mice controls. Data are presented as mean ± s.d., *n* = 5 mice per group. *P* values shown are from unpaired, 2-tailed Student’s *t* tests. Data are shown as mean ± s.d. (**M**–**T**) Representative graph of echocardiographic analysis showing changes in the various cardiac parameters of 12-month-old *Sirt2*-KO mice when compared with age-matched wild-type mice controls. Data are presented as mean ± s.d., *n* = 7 mice per group. *P* values shown are from unpaired, 2-tailed Student’s *t* tests. Data are shown as mean ± s.d.

Previous reports suggest that *Sirt2*-KO mice develop cardiac hypertrophy in an age-dependent manner, and *Sirt2*-KO mice are more sensitive to neurohormonal-induced cardiac hypertrophy (Sarikhani et al, 2018a; Tang et al, 2017). As mentioned earlier, increased protein synthesis is a well-known hallmark of mouse hearts exhibiting cardiac hypertrophy. Cardiomyocytes develop various mechanisms to meet the increased demand for protein in response to the increased cardiomyocyte mass during the hypertrophic stage of the heart. We used this model to determine if *Sirt2*-KO mice hearts are prone to developing hypertrophy due to the upregulated protein synthesis in the absence of SIRT2. To verify this, we first used young *Sirt2*-KO mice, 4-month-old, whose heart function was as normal as that of control mice (Fig. EV5A–G). We tested for the protein synthesis levels in the hearts of these young *Sirt2*-KO mice using the SUnSET assay. The protein synthesis was significantly upregulated in the heart tissue of *Sirt2*-KO mice compared to WT controls (Fig. 6A,B). Since our in vitro results suggest that SIRT2 promotes Rheb degradation, we examined Rheb levels in the heart tissue of *Sirt2*-KO mice. We found that Rheb protein levels were significantly upregulated in these tissues compared to WT controls (Fig. 6C,D).

**Figure EV6:**
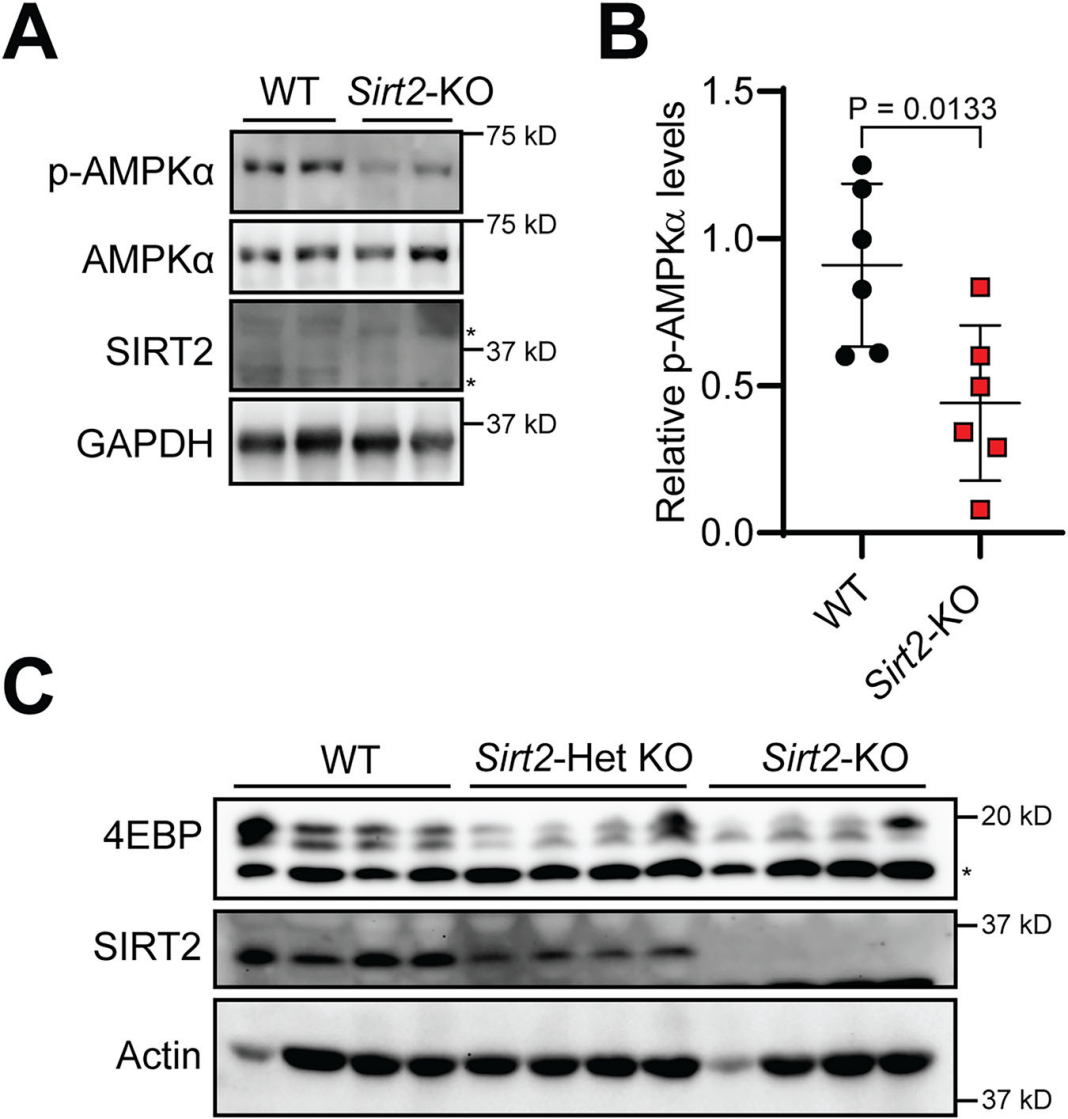
*Sirt2*-KO mice exhibit inhibition of AMPKα and a decrease in 4EBP levels. (**A**) Representative western blotting images depicting changes in AMPKα phosphorylation Threonine 172, in heart tissues of WT controls and *Sirt2*-KO mice, *n* = 6 animals per group. * Indicates non-specific band. (**B**) Scatterplot representing the changes in AMPKα protein phosphorylation in heart tissues of WT controls and *Sirt2*-KO mice depicted in Fig. EV6A. *P* values shown are from unpaired, two-tailed Student’s *t* test. Data are presented as mean ± s.d., *n* = 6 per group. (**C**) Representative western blotting images depicting changes in 4EBP levels in heart tissues of WT controls, *Sirt2*- Het KO and *Sirt2*-KO mice, *n* = 4 mice per group. * Indicates non-specific band.

Furthermore, we examined activation of mTOR signaling in *Sirt2*-KO mice’s heart tissues by determining the levels of p-mTOR and p-p70S6 Kinase 1 (p-p70S6K1), and we found that activation of mTOR signaling was upregulated in *Sirt2*-KO mouse hearts (Fig. 6E). In addition, we observed a significant reduction in AMPKα phosphorylation in the hearts of *Sirt2*-KO mice (Fig. EV6A,B), along with a decrease in 4EBP protein levels (Fig. EV6C). This suggests that multiple proteins involved in mTOR signaling are deregulated in the hearts of *Sirt2*-KO mice, contributing to the upregulated protein synthesis. Moreover, we report that the heart tissues of 9-month-old *Sirt2*-KO mice exhibited hypertrophic changes compared to wild-type controls (Figs. 6F–I and EV5H–L). Our echocardiography analysis demonstrates that *Sirt2*-KO mice show a decrease in Ejection Fraction (Fig. 6F), whereas left ventricular internal diameter, diastolic (LVID; d) (Fig. 6G), left ventricular internal diameter, systolic (LVID; s) (Fig. 6H), and heart weight to tibia length ratio (HW/TL) (Fig. 6I,J) were significantly upregulated. In addition, protein synthesis was significantly upregulated in these mice’s heart tissues (Fig. 6M,N). Since increased protein synthesis in cardiomyocytes correlates with increased cardiomyocyte size during hypertrophic changes, we performed WGA staining on heart tissue sections from 9-month-old *Sirt2*-KO mice. We found that *Sirt2*-KO mice display an increase in cardiomyocyte cross-sectional area compared to wild-type controls (Fig. 6K,L). We assessed for hypertrophic mRNA markers in these hearts and found a significant increase in levels of hypertrophic mRNA markers ANP, BNP, and β-MHC (Fig. 6O). These findings suggest that enhanced protein synthesis in *Sirt2*-KO mice, even at the early stage, can be a crucial factor for developing cardiac hypertrophy in *Sirt2*-KO mice when they age (Fig. EV5M–T).

### Acute SIRT2 inhibition affects mTORC1-dependent protein synthesis in NRCMs

**Figure 7:**
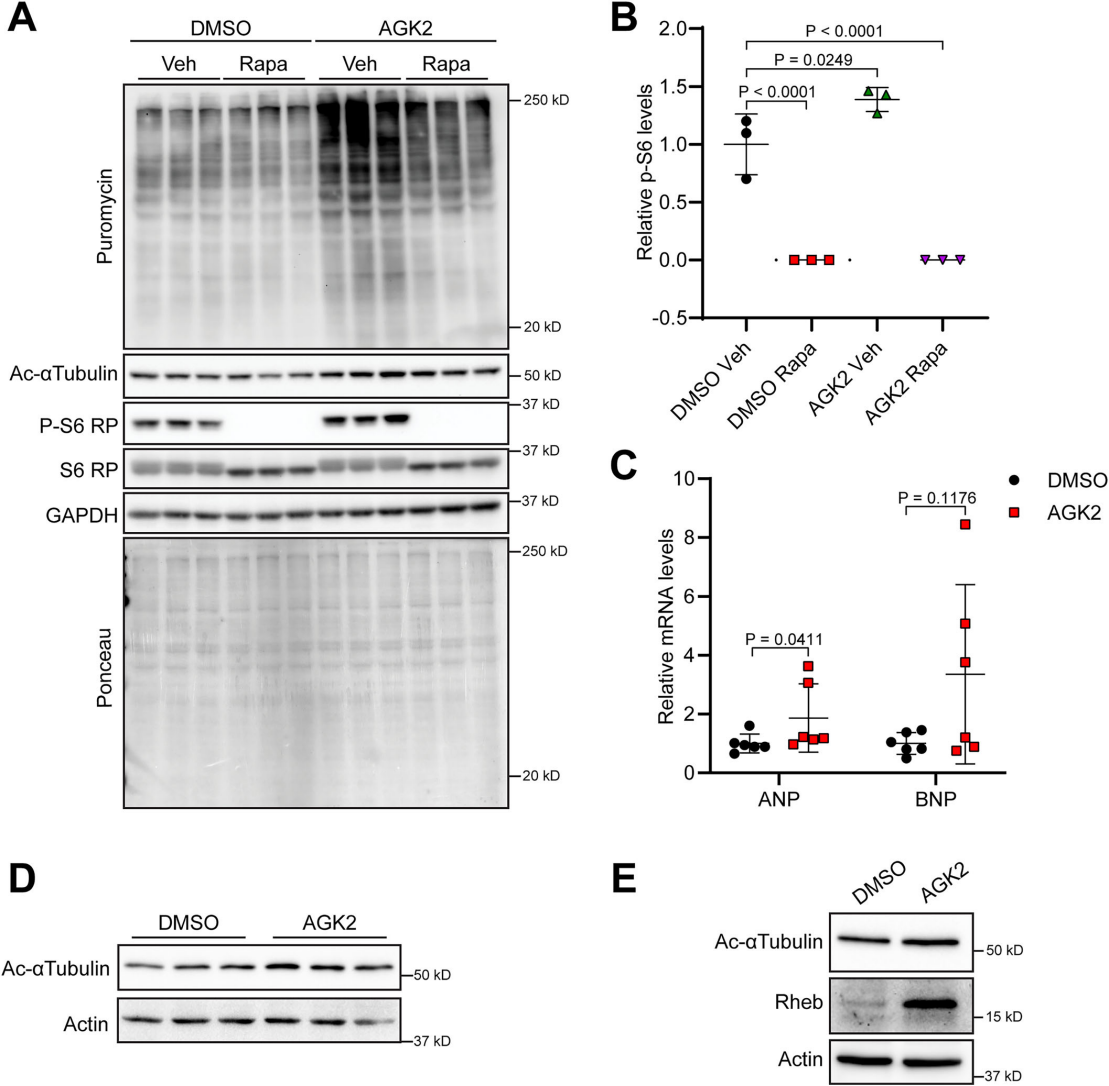
Acute SIRT2 inhibition affects mTORC1-dependent protein synthesis in NRCM. (**A**) Representative western blot images of SUnSET analysis depicting changes in protein synthesis rate in NRCMs treated with DMSO or SIRT2 inhibitor AGK2 for 24 h, followed by Vehicle or Rapamycin treatment of 6 h. S6 phosphorylation was assessed to check mTOR activity, and the Acetylation of α-tubulin was assessed to evaluate SIRT2 activity. (**B**) Scatterplot representing the changes in S6 ribosomal protein (S6 RP) phosphorylation (Serine 235/236) across different groups depicted in (**A**). *P* values shown are from two-way ANOVA with Dunnett’s multiple comparisons test. Data are presented as mean ± s.d., *n* = 3 per group. (**C**) qPCR analysis of relative mRNA expression levels of hypertrophy marker mRNAs ANP and BNP in DMSO and AGK2-treated NRCMs, normalized with GAPDH. *P* values shown are from unpaired, two-tailed Student’s *t* tests calculated separately for individual markers. Data are presented as mean ± s.d., *n* = 6 mice per group. (**D**) Representative western blot images showing changes in acetylated α-tubulin levels in NRCMs after DMSO (Vehicle) and AGK2 treatment, *n* = 3. (**E**) Representative western blot images of IP analysis depicting changes in Rheb protein levels in NRCMs treated with AGK2 compared to DMSO-treated control, *n* = 2. Source data are available online for this figure.

Since our in vitro results demonstrate that SIRT2 is a negative regulator of protein synthesis, and *Sirt2*-KO mice hearts exhibit upregulated protein synthesis concomitant with increased mTORC1 activity, we wanted to investigate whether acute SIRT2 manipulation has a similar effect in cardiac cells. To verify this, we inhibited SIRT2 in primary cardiomyocytes isolated from neonatal rat hearts (NRCMs) by treating AGK2 for 24 h. We observed an upregulated protein synthesis in these cardiomyocytes under AGK2 treatment, along with increased mTORC1 activity, as assessed by increased puromycin-incorporated peptides and increased phosphorylation of S6 ribosomal protein (Fig. 7A,B). Furthermore, to check if this upregulation was dependent on mTORC1 activation, we inhibited mTORC1 using Rapamycin and observed that Rapamycin treatment abrogated the upregulation of protein synthesis and mTORC1 activity in NRCMs treated with AGK2 (Fig. 7A). Previously, we reported that AGK2 treatment induces a hypertrophic phenotype in NRCMs (Sarikhani et al, 2018b). Moreover, another study has also emphasized that SIRT2 depletion/inhibition can exacerbate pathological stress-induced hypertrophy in cardiomyocytes (Tang et al, 2017). We assessed hypertrophy markers in NRCMs treated with AGK2 and observed a slight increase in ANP mRNA levels, with a trend towards increasing BNP levels (Fig. 7C). AGK2-mediated SIRT2 inhibition was confirmed by assessing acetylation of α-tubulin (Fig. 7D). In addition, we assessed Rheb levels in NRCMs treated with AGK2 and observed an increase in Rheb levels upon AGK2 treatment, concomitant with increased α-tubulin acetylation (Fig. 7E). These findings suggest that SIRT2 inhibition for a short duration can induce hypertrophic changes in cardiomyocytes concomitant with increased protein synthesis and upregulated mTORC1 activity.

### *Sirt2*-KO mice exhibit cardiac dysfunction under physiological stress

**Figure 8:**
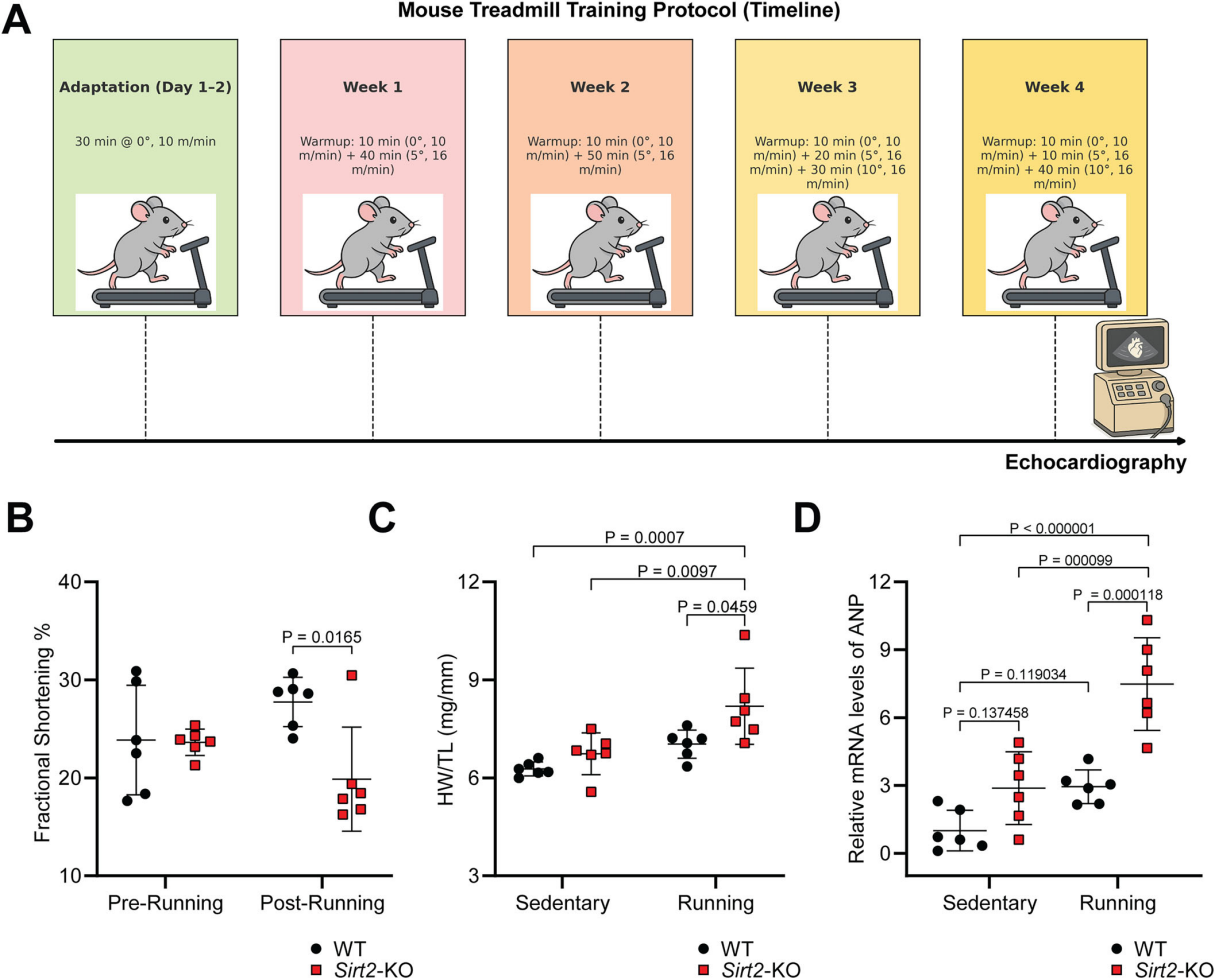
*Sirt2*-KO mice exhibit cardiac dysfunction under physiological stress. (**A**) Schematic diagram representing the treadmill exercise protocol used to provide physiological stress in WT and *Sirt2*-KO mice. Echocardiography was performed on these mice at the beginning (Day 0) and end (Day 30) of this training procedure. (**B**) A representative graph of echocardiographic analysis showing changes in the fractional shortening in *Sirt2*-KO and WT mice subjected to treadmill running for 30 days. *P* values shown are from two-way ANOVA with Tukey’s multiple comparisons test. Data are presented as mean ± s.d., *n* = 6 mice per group. (**C**) Scatterplot representing changes in heart weight to tibia length ratio (HW/TL) in *Sirt2*-KO mice and WT mice subjected to treadmill running for 30 days when compared with their respective sedentary controls. *P* values shown are from two-way ANOVA with Tukey’s multiple comparisons test. Data are presented as mean ± s.d., *n* = 6 mice per group. (**D**) qPCR analysis of relative mRNA expression levels of hypertrophy marker ANP in the hearts of *Sirt2*-KO mice subjected to treadmill running (*Sirt2-*KO Running) and WT mice subjected to treadmill running (WT Running) at D30, when compared with their respective sedentary controls, values are normalized with the Ribosomal Protein Large Subunit gene (RPL32). *P* values shown are from two-way ANOVA with Tukey’s multiple comparisons test. Data are presented as mean ± s.d., *n* = 6 mice per group. Source data are available online for this figure.

Although SIRT2’s role in the regulation of hypertrophic growth in the heart under pathological stress is well documented, no study has emphasized the importance of SIRT2 in the heart during physiological stress. Moreover, Rheb has been reported as a crucial protein during exercise-induced cardiac remodeling (Blackwood et al, 2019). To assess the cardiac function of *Sirt2*-KO mice under physiological stress, we utilized rigorous exercise as a model to induce physiological stress. We subjected *Sirt2-*KO mice to 1 month of rigorous exercise training using a treadmill (physiological stress), as described in Fig. 8A, and assessed their cardiac function using echocardiography. We have observed that the cardiac functions of WT running mice remained preserved, whereas *Sirt2*-KO mice under exercise training exhibited decreased Fractional Shortening (Fig. 8B) and increased HW/TL (Fig. 8C), along with upregulated expression of the hypertrophy mRNA marker ANP (Fig. 8D), suggesting that *Sirt2-*KO mice exhibit malfunctioned cardiac adaptation in response to physiological stress.

### Cardiac-specific SIRT2 overexpression causes a reduction in mTOR activity and protein synthesis in mice hearts

**Figure 9:**
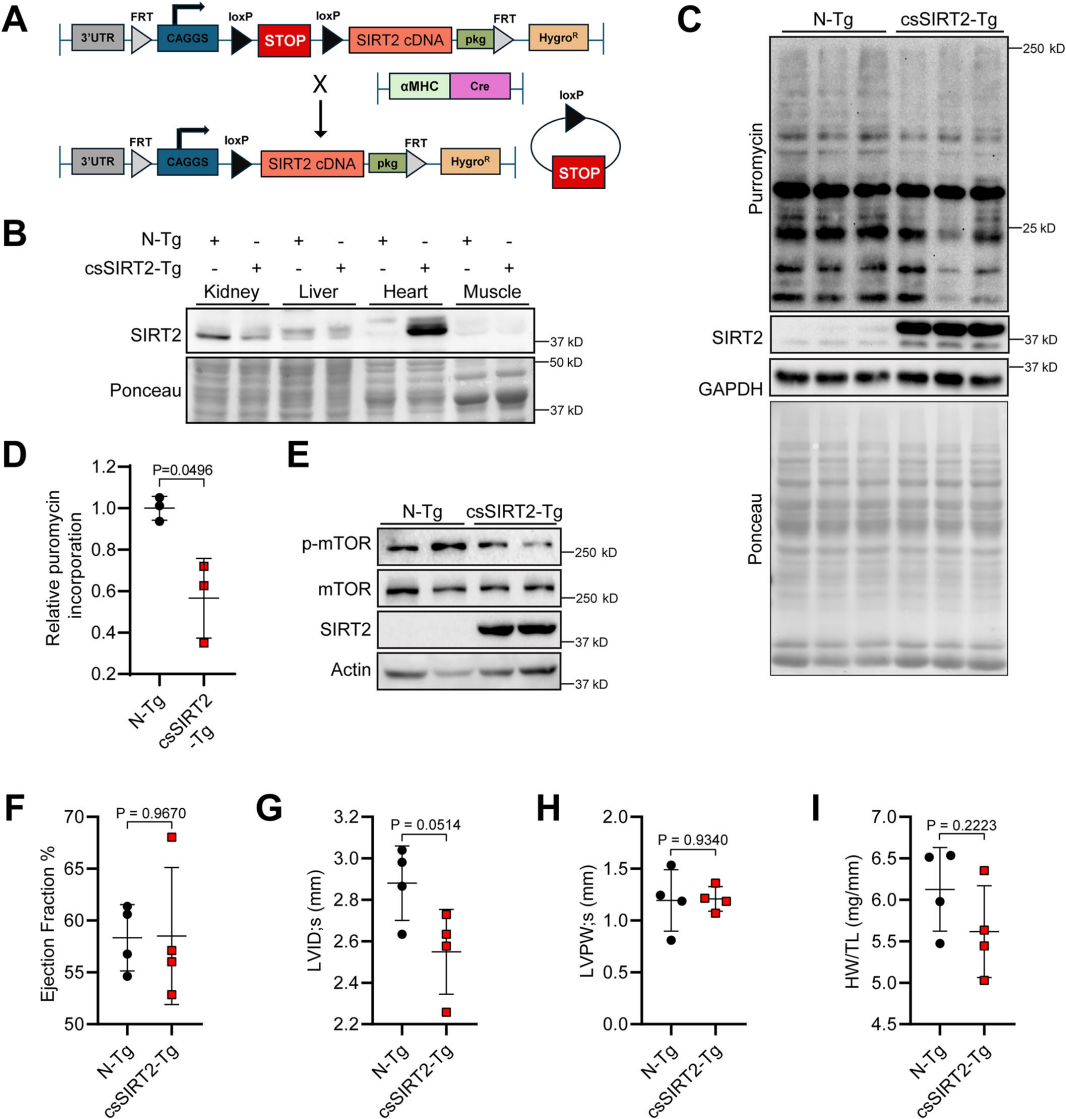
csSIRT2-tg mice hearts display reduced mTOR kinase activity concomitant with a reduced rate of protein synthesis. (**A**) Schematic diagram representing the generation of the cardiac-specific SIRT2-transgenic (csSIRT2-Tg) mice line. (**B**) Representative images of western blotting analysis in different organs of csSIRT2-Tg and control mice showing cardiac-specific overexpression of SIRT2 in the hearts of csSIRT2-Tg mice. (**C**) Representative images of western blotting SUnSET analysis depicting changes in protein synthesis rate in the heart tissues of Non-Transgenic(N-Tg) controls and csSIRT2-Tg mice. (**D**) Scatterplot representing the quantification of puromycin incorporation depicted in (**C**). The results are expressed as the fold change relative to non-transgenic controls. *P* values shown are from unpaired, two-tailed Student’s *t* test. Data are presented as mean ± s.d., *n* = 3 mice per group. (**E**) Representative western blotting images depicting changes in mTOR activation (mTOR phosphorylation at Serine 2448) in heart tissues of Non-Transgenic(N-Tg) controls and csSIRT2-Tg mice. (**F**) A representative graph of echocardiographic analysis showing changes in the ejection fraction of csSIRT2-Tg mice when compared with age-matched N-Tg mice controls. *P* values shown are from an unpaired, two-tailed Student’s *t* test. Data are presented as mean ± s.d., *n* = 4 mice per group. (**G**) A representative graph of echocardiographic analysis showing changes in the Left ventricular internal diameter, systolic (LVID; s) of csSIRT2-Tg mice when compared with age-matched N-Tg mice controls. *P* values shown are from an unpaired, two-tailed Student’s *t* test. Data are presented as mean ± s.d., *n* = 4 mice per group. (**H**) A representative graph of echocardiographic analysis showing changes in the Left ventricular posterior wall thickness, systolic (LVPW; s) of csSIRT2-Tg mice when compared with age-matched N-Tg mice controls. *P* values shown are from an unpaired, two-tailed Student’s *t* test. Data are presented as mean ± s.d., *n* = 4 mice per group. (**I**) Scatterplot representing the heart weight of age-matched csSIRT2-Tg mice and N-Tg mice. Heart weight is normalized with the respective mice’s tibia length. The results are expressed as the fold change relative to WT controls. *P* values shown are from an unpaired, two-tailed Student’s *t* test. Data are presented as mean ± s.d., *n* = 4 mice per group. Source data are available online for this figure.

Next, to understand if cardiac-specific overexpression of SIRT2 can reduce protein synthesis in these mice hearts, we developed cardiac-specific SIRT2 overexpressing (csSIRT2-Tg) mice (Fig. 9A). We confirmed cardiac-specific overexpression through western blotting (Fig. 9B). We tested protein synthesis levels in the heart tissue of these young mice using the SUnSET assay. We found that protein synthesis was significantly downregulated in the heart tissue of csSIRT2-Tg mice compared to control mice (N-Tg) (Fig. 9C,D). In addition, we observed a reduction in mTOR phosphorylation in the heart tissue of csSIRT2-Tg mice compared to control mice (Fig. 9E). These findings suggest that csSIRT2-Tg mice show reduced protein synthesis and decreased mTOR activation. In addition, echocardiographic assessment of young csSIRT2-Tg mice revealed no significant differences in cardiac function, as parameters such as ejection fraction, LVID;s and LVPW;s remained comparable to those of control mice (Fig. 9F–H). Moreover, the heart weight to tibia length ratio (HW/TL) was also similar between csSIRT2-Tg and control mice (Fig. 9I). These findings suggest that while young csSIRT2-Tg mice exhibit downregulation of protein synthesis, their cardiac function remains comparable to that of control mice.

## Discussion

Our work demonstrates a new role of SIRT2 in regulating protein synthesis. We identified Rheb as a novel substrate of SIRT2 and demonstrated a unique regulation of Rheb protein levels in cells through its post-translational modifications. Specifically, SIRT2-mediated deacetylation of Rheb on K151 promotes Rheb ubiquitination and proteasomal degradation; Rheb is a crucial activator of mTORC1. Thus, this regulation leads to the downregulation of mTOR activity and, consequently, a reduction in protein synthesis.

SIRT2’s effect on protein synthesis also holds true in mouse models and regulates cardiac hypertrophy in mice. Multiple reports have demonstrated that SIRT2 deficiency in the heart leads to age-associated and neurohormonal-induced cardiac hypertrophy (Sarikhani et al, 2018a; Tang et al, 2017). We observed increased protein synthesis rates in the heart tissues of young *Sirt2*-KO mice, accompanied by elevated Rheb levels and upregulated mTOR activity. We believe that a continuous load of increased protein synthesis in the hearts of these mice contributes to the development of cardiac hypertrophy during aging. Pathological cardiac hypertrophy is a complex remodeling process involving metabolic reprogramming, enhanced mTOR-dependent protein synthesis, re-expression of fetal gene programs, and myofibrillar remodeling (Cox and Marsh, 2014; Dai et al, 2023; Hong and Zhang, 2022; Machackova et al, 2006; Wang et al, 2021) which is controlled by distinct signaling effectors acting at different biological levels. While our previous work demonstrated that SIRT2 regulates cardiac hypertrophy through NFATc2-dependent transcriptional mechanisms (Sarikhani et al, 2018a), the current study specifically focuses on defining a complementary SIRT2–Rheb–mTORC1 axis that controls hypertrophic growth at the level of mTOR-dependent translational regulation. Thus, the present findings extend our earlier work by identifying an additional SIRT2-regulated pathway that contributes to pathological hypertrophy through control of protein synthesis. Furthermore, studies have shown that Rheb levels are upregulated in pressure overload and aging-induced hypertrophic hearts (Blackwood et al, 2019; Ravi et al, 2019). Rheb is a crucial protein for cardiac hypertrophic growth even in the absence of any pathological stimuli. A recent study has highlighted the importance of Rheb in the physiological adaptation of cardiomyocytes during exercise, where Rheb is transcriptionally upregulated in the hearts of exercised mice (Blackwood et al, 2019). Furthermore, another study suggests that Rheb overexpression alone is sufficient for cardiac hypertrophic growth concomitant with increased mTORC1 activation (Wang et al, 2008), suggesting that regulation of Rheb levels is important for cardiac health. While adequate levels of Rheb are crucial for cardiac remodeling during exercise, higher Rheb levels may have an adverse effect even under physiological stress. Our data demonstrate that *Sirt2*-KO mice display deregulated cardiac remodeling during treadmill-based exercise training.

We generated a cardiac-specific SIRT2-overexpressing mouse model and found that the heart tissues of these mice exhibit a decreased rate of protein synthesis, accompanied by decreased mTOR kinase activity. Moreover, a recent study from Tang et al (Tang et al, 2017) suggests that csSIRT2-Tg mice are protective against Ang II-induced cardiac hypertrophy. Our findings suggest that SIRT2-mediated control of protein synthesis may play a protective role in csSIRT2-Tg mice against cardiac hypertrophy.

While we focus on cardiac hypertrophy, the SIRT2-Rheb-mTORC1 regulation axis may also be relevant for other human diseases. Deregulated mTOR is strongly associated with various pathologies like arthritis, cancers, neurological disorders, osteoporosis, and insulin resistance (Benjamin et al, 2011). SIRT2 has also been connected to several human diseases. For example, SIRT2 is reported to improve insulin sensitivity, and the inhibition of SIRT2 is associated with an enhanced progression of diabetic osteoarthritis. SIRT2 also has a disease-promoting role in neurological disorders, positively associated with the progression and development of multiple neurological disorders (Zhu et al, 2022). We suspect that the SIRT2-Rheb-mTORC1 regulatory pathway may explain the diverse roles of SIRT2 in various human diseases.

Sirtuin activities are thought to be regulated by calorie restriction and nutrient availability. This has been demonstrated for SIRT1 and, more recently, for SIRT2 (Guarente, 2013; Wang et al, 2007; Zullo et al, 2018). It has been reported that SIRT2 levels and deacetylase activity are upregulated in response to amino acid starvation (Zullo et al, 2018). Recently, it has been reported that SIRT2 deacetylase activity is required to inhibit lipogenesis when cells undergo nutrient stress, primarily due to amino acid deficiency (Karim et al, 2024). In this current study, we also observed that complete cell starvation increases SIRT2 levels and activity, concomitant with a decrease in global protein synthesis. These findings suggest that SIRT2 is a crucial nutrient-responsive protein that regulates multiple cellular processes in response to changes in nutrient availability.

Moreover, it has been reported that Rheb undergoes K48-linked polyubiquitination under low glucose availability, leading to the inactivation of mTORC1 (Li et al, 2024), which suggests the essentiality of regulating Rheb levels and activity during glucose starvation. In the current study, using different cell types, we observed that SIRT2 activity increases during glucose starvation concomitant with decreased Rheb levels, suggesting that increased SIRT2 activity during glucose deprivation contributes to regulating Rheb levels. In our study, we demonstrate that SIRT2 promotes Rheb degradation through deacetylation. A crucial question that remains unanswered in this study is which E3 ubiquitin ligase(s) work with SIRT2 to promote Rheb degradation. Recent advancements in the field suggest that candidates, such as the CUL4-DDB1 E3 ubiquitin ligase complex (Li et al, 2024) and the RNF152 E3 ligase (Deng et al, 2019), may regulate Rheb stability under specific nutrient stress conditions. However, the roles of these E3 ligases were not characterized in the present study, and further investigations are required to address this question. We also recognize that SIRT2 likely acts on additional substrates that can influence translation. Our companion manuscript demonstrates that SIRT2 deacetylates 4EBP1, promoting its stabilization and consequent suppression of translation initiation (preprint: Zi et al, 2025). Notably, in the current study, we report that hearts from *Sirt2*-KO mice exhibit reduced 4EBP levels (Fig. EV6C), indicating that multiple components of mTORC1 signaling are dysregulated in these animals, contributing to enhanced global protein synthesis. These findings further support the role of SIRT2 in modulating global protein synthesis by deacetylating a diverse range of substrates. Furthermore, assessment of mTORC1 signaling in NRCMs under EBSS treatment revealed decreased S6 RP phosphorylation alongside increased 4EBP levels (Fig. EV2C), suggesting that these mechanisms act concurrently during EBSS-induced nutrient stress. It is important to note that EBSS lacks multiple nutrients and growth factors, which may exacerbate the combined effects of various nutrient and growth factor stresses. Notably, the two studies employ distinct nutrient stress paradigms: our current work uses EBSS and glucose deprivation to investigate SIRT2’s regulatory role under these conditions, whereas our companion study examines amino acid starvation to probe SIRT2-mediated responses. Despite these mechanistic and experimental differences, both studies converge on a common phenotypic outcome: SIRT2-dependent suppression of global protein synthesis under stress. Together, these findings reveal complementary SIRT2-dependent mechanisms that coordinate both rapid and sustained repression of translation. While our study focuses on SIRT2-mediated Rheb deacetylation, it is important to note that acetyltransferases may also play a complementary role in this regulatory cycle. Identifying the specific acetyl-transferases responsible for Rheb acetylation and understanding how their activity intersects with SIRT2 highlights an important scope for future research. Such studies would provide a more comprehensive understanding of the dynamic acetylation–deacetylation cycle that governs Rheb function and stability.

In summary, our study highlights the intricacy of protein synthesis regulation, a process that holds a controlling influence on cell health and survival. The finding that SIRT2, through Rheb and mTORC1, regulates protein synthesis and cardiac hypertrophy may also help uncover new targets for treating cardiovascular diseases.

## Methods

**Table:** Reagents and tools table

Reagent/resource	Reference or source	Identifier or catalog number
**Experimental models**		
Sirt2-KO mice	Jackson Laboratories, USA	B6.129-Sirt2tm1.1Fwa/J, RRID: IMSR_JAX:012772
SIRT2 flox/flox mice	Jackson Laboratories, USA	B6.Cg-Col1a1tm1(CAG-Sirt2) Jmi/DsinJ
αMHC-Cre mice	Jackson Laboratories, USA	B6.FVB-Tg(Myh6-cre)2182Mds/J
C57 WT mice (C57BL/6 J)	Jackson Laboratories, USA	RRID:IMSR_JAX:000664
HEK293T (Cell line)	ATCC	Cat#CRL-3216,RRID:CVCL_0063
HeLa (Cell line)	ATCC	Cat#CRM-CCL-2,RRID:CVCL_0030
**Recombinant DNA**		
pcDNA3.1	Invitrogen	V790-20
SIRT2 Flag	Addgene	13813
pRK5-HA GST Rheb1	Addgene	19310
pCDNA3-EMCV bicis Reporter plasmid	Previously described	PMID: 31455634
pEGFP-C1-Rheb	Addgene	133767
pcDNA3-FLAG-Rheb	Addgene	19996
pGEX-4T-3 GST	GE Healthcare	28-9545-52
**Antibodies**		
Anti-SIRT2	Cell Signaling Technology	D4O5O
Anti Rheb {Rheb (E1G1R)}	Cell Signaling Technology	13879
Acetyl-α-Tubulin (Lys40)	Cell Signaling Technology	5335
anti-Rabbit IgG light chain HRP	Cell Signaling Technology	58802
Normal Rabbit IgG	Cell Signaling Technology	2729
Anti-p-mTOR	Cell Signaling Technology	5536
Anti mTOR	Cell Signaling Technology	2983
Anti-Rabbit HRP	Cell Signaling Technology	7074
Anti-Mouse HRP	Cell Signaling Technology	7076
Anti-HSP90	Cell Signaling Technology	4877
Anti-Ubiquitin	Cell Signaling Technology	E4I2J
Anti-HA	Cell Signaling Technology	C29F4
Anti-phospho p70S6K	Cell Signaling Technology	9205
Anti-p70S6K	Cell Signaling Technology	2708
Anti-AMPK α	Cell Signaling Technology	2532 s
Anti-p-AMPK α	Cell Signaling Technology	40H9
Anti-p-4E-BP1	Cell Signaling Technology	236B4
Anti 4E-BP1	Cell Signaling Technology	9452
Anti-GAPDH	Sigma-Aldrich	G9545
Anti-Actin	Sigma-Aldrich	A3854
Anti-SIRT2	Sigma-Aldrich	S8447
Anti-FLAG HRP-conjugated	Sigma-Aldrich	A8592
Anti-S6 ribosomal protein	Thermo Fisher Scientific	MA5-15164
Anti-phospho S6 ribosomal protein	Thermo Fisher Scientific	MA5-15140
Donkey anti-mouse, Alexa Fluor 488	Thermo Fisher Scientific	A-21202
Goat anti-rabbit, Alexa Fluor 546	Thermo Fisher Scientific	A-11035
Clean-Blot IP Detection Reagent	Thermo Fisher Scientific	21230
Anti-HA	ABclonal	AE008
Anti-Kac	PTM Biolabs Inc	PTM-101
Anti-Puromycin	DSHB	PMY-2A4
Anti-Puromycin	abcam	ab315888
**Oligonucleotides and other sequence-based reagents**		
SignalSilence® Rheb siRNA I	Cell Signaling Technology	14267
Genotyping Primers	This study	Table 1
siRNAs	This study	Table 1
Mutagenic Primers	This Study	Table 1
Rheb sequencing Primer	This Study	Table 1
qRT-PCR Primers	This Study	Table 1
**Chemicals, enzymes, and other reagents**		
Dulbecco’s Modified Eagle’s Medium- High glucose	HIMEDIA	AL007A-500ml
Antibiotic-Antimycotic mixture	Thermo Fisher Scientific	15240062
Trypsin-EDTA	Thermo Fisher Scientific	25200056
Trypsin	Thermo Fisher Scientific	15050057
Fetal Bovine Serum	Thermo Fisher Scientific	10500064
Acetyl-Lysine Affinity Beads	Cytoskeleton, Inc	AAC04
Lipofectamine ®2000 Reagent	Thermo Fisher Scientific	11668-019
Lipofectamine ®3000 Reagent	Thermo Fisher Scientific	L3000-015
Lipofectamine RNAiMAX	Thermo Fisher Scientific	13778150
Anti-HA Magnetic beads	Thermo Fisher Scientific	PI88837
Dual-Luciferase® Reporter Assay System	Promega	E1910
QuikChange Site-Directed Mutagenesis Kit	Agilent Technologies	200518
Protein A/G MagBeads	GenScript	L00277
Clarity ECL Western Blotting Substrate	Bio-Rad	5060
Clarity-max western ECL substrate	Bio-Rad	1705062
Anti-FLAG affinity gel	Sigma-Aldrich	A2220
Protein Assay Dye Reagent Concentrate	Bio-Rad	L007096 D
PowerUp™SYBR™ Green Master Mix	Applied biosystems® by Thermo Fisher Scientific	A25742
PhiScript™ – cDNA Sythesis Mastermix (5X)	dxbidt	R6203
RNAiso Plus	TaKaRa	9109
SIRT2 Inhibitor, AGK2	Sigma-Aldrich, Merck	304896-28-4
cOmplete™, Mini Protease Inhibitor Cocktail	Sigma-Aldrich, ROCHE	11836153001
Polyethyleneimine	Polysciences	24765
MG132	MedChemExpress	HY-13259
Fluoromount	Sigma-Aldrich	F4680
Wheat germ agglutinin Alexa Fluor™ 594 Conjugate	Thermo Fisher Scientific	W11262
Hoechst 33342	Thermo Fisher Scientific	H3570
Puromycin dihydrochloride	Santa Cruz	CAS 58-58-2
Rapamycin	Cayman Chemical	13346
Torin1	Cayman Chemical	10997
Ponceau S	Sigma-Aldrich	P3504
Amersham Hybond PVDF membrane	GE Healthcare	10600023
Rapamycin	Sigma-Aldrich	R8781
**Software**		
ImageJ	https://imagej.net/ij/	
Image Lab	https://www.bio-rad.com/en-in/product/image-lab-software?ID=KRE6P5E8Z	
ZEISS ZEN lite	ZEISS	
**Other**		
QuantStudio™ 6 Flex Real-Time PCR System	Applied biosystems® (Life technologies)	REF- 4484642
Visual Sonics high-frequency ultrasound system	Vevo 1100	
LSM 880 confocal microscope	Zeiss	
ChemiDoc™ Touch Imaging System	Bio-Rad	
Luminometer	Turner Machine, TD-20/20	
Treadmill for rodents	Ugo basile®	47300-001
ProFlex PCR System	Applied biosystems® (Life technologies)	

### Cell culture, treatment, and transfection

HeLa cells were grown in a high-glucose-containing DMEM medium with 10% fetal bovine serum supplement along with antibiotic-antimycotic. After seeding, cells were subjected to mix-humified incubator at 37 °C, 5% CO_2_. The starvation experiment was performed by starving cells for 4 h using EBSS (Earle’s balanced salt solution). Transfection was performed in cells when they were at 70–80% confluency. Lipofectamine®3000 reagent was used for the plasmid transfection, whereas Lipofectamine RNAiMAX was used for the transfection of siRNAs, all these transfections were performed according to the manufacturer’s protocol, and the cells were harvested at 48 h post-transfection in case of plasmid-mediated overexpression and at 72 h for siRNAs-mediated gene knockdown. AGK2 treatment (10 μM) was used for the inhibition of SIRT2 activity. Protein synthesis rate was measured through the Surface Sensing of Translation (SUnSET) assay as described in our previously published study (Ravi et al, 2020). Puromycin (1 μM) was added to the complete DMEM media 30 min before cell harvesting. Primary cardiomyocytes were isolated from Sprague-Dawley rats following our previous publication (Ravi et al, 2021a).

### Animal studies

All animal studies were carried out after getting approval from the Institutional Animal Ethics Committee of IISc, Bengaluru, India. The animals were given a normal chow diet and were considerably maintained in cages provided with good ventilation. A 12-hour light/dark cycle was followed for these animals throughout the study. For the in vivo SUnSET assay, mice were injected with puromycin. Puromycin was given at a dose of 40nmol/g of body weight via an intraperitoneal injection. Post 30 min of puromycin administration, the mice were sacrificed, and tissue harvesting was performed. Harvested tissues were snap-frozen and stored at −80 °C until further use. 4-month and 9-month age-matched wild-type control, and SIRT2 Knockout (*Sirt2*-KO) mice were used for the SUnSET assay. 2-month-old control and csSIRT2-Tg were administered with puromycin to evaluate the protein synthesis rate and mTOR kinase activity. Change in mTOR activation was also evaluated in the same age-grouped csSIRT2-Tg mice. Cardiac functions of *Sirt2-*KO mice at 4, 9, 12 months and age-matched control mice were examined via Visual Sonics high-frequency ultrasound system as mentioned previously (Sarikhani et al, 2018b).

### Treadmill exercise training

Wild-type (WT) and *Sirt2*-KO mice of 4–4.5 months of age were used for this experiment. The experiment was carried out for 4 weeks, adopting a previously used protocol to assess cardiac growth in mice in response to treadmill running (Fulghum et al, 2024). A schematic of the exercise procedure is described in Fig. 8A. Briefly, Mild training was given to the mice before the experiment began, where they were subjected to treadmill running for 30 min at a 0° inclination with a speed of 10 m/minute. Mice underwent treadmill exercise five days per week. To provide a progressive overload stimulus for physiological adaptation, the duration of daily exercise was gradually increased throughout the training period. A 10-minute warm-up at 0° inclination with a speed of 10 m/minute was compulsory at the beginning of the exercise and throughout the entire course of the experiment. During the first week, mice ran for 40 min per day at a 5° inclination with a speed of 16 m/min; in the second week, the duration increased to 50 min per day; and during week three, mice trained for initial 20 min at 5° inclination with a speed of 16 m/min, while keeping the speed constant inclination was increased to 10° for additional 30 min. In week four, mice were initially trained for 10 min at a 5° inclination with a speed of 16 m/min, followed by an additional 40 min at a 10° inclination with the same speed. Mice were immediately removed from the treadmill upon meeting one or more of the following exhaustion criteria: (1) remaining on the electric grid for 10 consecutive seconds; (2) spending more than 50% of the time on the treadmill grid; and/or (3) displaying a lack of response to manual prodding, indicating diminished motivation to continue running. To assess cardiac adaptations to the training regimen, echocardiographic analysis was performed, and the mice were then harvested to evaluate hypertrophic adaptation.

### Cell and tissue lysate preparation

Cells were harvested from their respective culture dishes by using a cell scraper after adding required volume of RIPA lysis buffer (20 mM Tris–HCl pH 7.5, 1 mM EDTA, 150 mM NaCl, 1 mM EGTA, 1% NP-40, 1% sodium deoxycholate, 1 mM sodium orthovanadate, 2.5 mM sodium pyrophosphate, 1 mM PMSF and 1× protease inhibitor cocktail). The same buffer was used to lyse tissue samples. Prior to the lysis, Tissue samples were frozen in liquid N_2_ and ground by using a mechanical approach. Centrifugation at 13,000 rpm, 10 min at 4 °C was performed to clear out the lysate, and the supernatant was collected and stored at -80 °C to ensure long-term stability.

### Site-directed mutagenesis

Rheb mutants, K151Q, and K151R were constructed by using site-directed mutagenesis of pRK5 HA GST Rheb1 plasmid. We have used QuikChange II Site-Directed Mutagenesis Kit (Agilent Technologies) and performed SDM according to the manufacturer’s protocol. These mutants contain a single point mutation of the indicated Lysine (K) residues, where Lysine has been replaced by either Arginine (R) or Glutamine (Q). SIRT2 catalytic mutant N168A was also generated using the same kit as mentioned above, and all these mutants were confirmed by sequencing analysis.

### Electrophoresis and immunoblotting

Quantification of protein lysates was done by using the Bradford assay (Bio-Rad). The protein samples were normalized by using an equal amount of protein. In all, 2× Laemmli sample buffer was added to the lysates along with 5% beta-mercaptoethanol, and the mixture was boiled at 95 °C for 5 min. Samples were subjected to electrophoresis on 10% SDS-PAGE gels. After the adequate time of electrophoresis, proteins were transferred onto 0.45 μm PVDF membranes by using fast or slow wet transfer. After the completion of transfer, the membrane was then blocked for 1 h at RT with 5% skim milk prepared in 1× TBST (Tris-Buffered Saline supplemented with 0.05% Tween 20). Specific antibodies diluted either in 5% BSA in TBST or 5% Skim milk in TBST were added to the membranes, followed by overnight incubation at 4 °C. The membrane-bound primary antibodies were then recognized by using HRP-conjugated secondary antibodies diluted in 1% skim milk for 1 hr at RT. Washings in the subsequent steps were done by using TBST. Chemiluminescence signal was detected with the use of ECL reagents, and the images were obtained with the help of a Chemiluminescence imager.

### Immunofluorescence microscopy

To perform immunofluorescence microscopy-based studies, the cells were first seeded on sterile glass-made coverslips in a 12-well plate. After subsequent transfections and treatment, at the experimentally decided time, cells were washed thrice with 1× PBS. After completely draining the PBS out the cells were fixed by using 4% paraformaldehyde (PFA) for 15 min at RT. Upon fixation, the extra PFA was removed by giving PBS washes, and the fixed cells were permeabilized and blocked in a single step with 0.1% Saponin - 5% BSA solution in PBS for 1 h at RT. After the blocking, specific antibodies prepared in 1% BSA - 0.1% saponin in PBS solution were added to the cells. Cells were then incubated overnight at 4 °C. After the completion of incubation, respective secondary antibodies conjugated with Alexa Fluor 488 and/or Alexa Fluor 594 prepared in 1% BSA - 0.1% saponin in PBS along with Hoechst 33342 were added to the cells for 1 h. The cells were washed three times with 1× PBS and mounted on a clean ethanol-wiped glass slide by using Fluoromount G. Images were obtained with a Zeiss LSM 710 or 880 confocal microscope.

### Immunoprecipitation assay

To check protein-protein interaction, HeLa cells were lysed using cell lysis buffer (20 mM Tris–HCl pH 7.5, 1 mM EDTA, 150 mM NaCl, 1 mM EGTA, 1% Triton X100, 1 mM sodium orthovanadate, 2.5 mM sodium pyrophosphate, 1 mM PMSF and 1× protease inhibitor cocktail). A total of 750–1000 μg protein was incubated with 2 μg of specific antibody. This antibody-protein mixture was incubated overnight at 4 °C, 5 rpm in a rotator. Upon overnight incubation, immunoprecipitated complexes were pulled down from the mixture by using magnetic protein G Sepharose beads (Bio-Rad). After subsequent washing with the PBS, these beads were resuspended in 2× Laemmli buffer and the mixture was subjected to heat-mediated dissociation at 95 °C for 5 min; further 5% β-mercaptoethanol was added, and the mixture was again incubated at 95 °C for 2 min, and proteins were detected using western blotting. For Rheb-SIRT2 interaction, endogenous Rheb was pulled down using a Rheb-specific antibody. Western blotting analysis was performed, and Rheb-SIRT2 interaction was confirmed using Rheb and SIRT2-specific antibodies.

### Cap-dependent translation assay

pcDNA3-EMCV bicis plasmid was used to evaluate cap-dependent translation in cells under different treatment conditions. Briefly, this plasmid contains R Luc under a 5’ caped site (representing cap-dependent translation) and F Luc under an EMCV IRES sequence (used as internal control). This bicis plasmid was transfected in HeLa cells under different treatment conditions. Lipofectamine 3000 was used as a transfection reagent to enhance transfection efficiency. Luciferase readings were measured using a dual-luciferase reagent kit (Promega) according to the manufacturer’s protocol.

### Real-time PCR

The integrity of the extracted RNA was evaluated to ensure suitability for downstream applications. Subsequently, 1 μg of high-quality total RNA was reverse transcribed into complementary DNA (cDNA) using a reverse transcription kit. Quantitative real-time PCR (qPCR) was then carried out using SYBR Green PCR Master Mix on a real-time PCR system to assess gene expression levels.

### Protein expression and purification

Human Ras homolog enriched in brain (Rheb) 1–169 aa and its mutants K151 and K151Q were expressed using GST-tagged pGEX 4 T vector in Rossetta BL21 cells. Expression was induced using 0.5 mM IPTG at 0.6 OD_600_ for 5 h at 37 °C, and cells were pelleted at 4500 rpm. ^15^N-labeled and unlabeled hRheb and mutants were expressed in a similar manner.

Sonication/lysis buffer 50 mM Tris, pH 7.5, 150 mM NaCl, 1 mM MgCl2 was used for resuspension of pellets, and cell lysis was performed using sonication. Lysed cells were subjected to centrifugation at 13,000 rpm for one hour. Supernatant was loaded on pre-packed GST column. Bound GST-tagged Rheb or the mutants were eluted in 50 mM Tris, 100 mM NaCl, 10 mM reduced Glutathione, pH 8.0. The GST tag was removed using thrombin by incubating for 16 h at 4–8 °C. After incubation period, 0.1–1 mM PMSF was used to inhibit thrombin followed by the sample, which was subjected to gel filtration (S75 column) to separate GST and Rheb (and mutants). During gel filtration, Rheb or mutants were eluted in 50 mM Phosphate, pH 7.5, 100 mM NaCl 5 mM MgCl_2_.

### NMR experiments

2D ^1^H-^15^N HSQC NMR spectra of Rheb without GTP analog (GppNHp) and with GTP analog were recorded on Bruker 700 MHz spectrometer at 298 K. 180 μM Rheb was prepared in 50 mM Phosphate, pH 7.5, 100 mM NaCl 5 mM MgCl_2_, and same buffer was used for GTP analog (GppNHp) solution. Titration experiments were performed for Rheb WT, Rheb K151Q and Rheb K151R with GppNHp at five different ratio (1:0.25, 1:0.5, 1:1, 1:2 and 1:5). All the samples contained 10% D_2_O for the spectrometer deuterium lock. All NMR results were processed using Bruker TOPSPIN 3.1, and data were analyzed using NMRFAM-SPARKY. Resonance assignment of few residues were taken from a previously published assignment from Biological Magnetic Resonance Data Bank (BMRB) entry 15202 (Schwarten et al, 2007).

### Pulling simulation (umbrella sampling)

To examine binding of GDP with WT Rheb, Rheb K151Aly, Rheb K151K, and Rheb K151Q, pulling force simulation was performed (Lemkul and Bevan, 2010). Molecular topology of all four systems were generated using Charmm36 forcefield parameters included in GROMACS package. Box dimension was set for 6.560 × 4.362 × 12, and center of mass is placed at 3.280, 2.181, 2.4775 in this box. Energy minimization and equilibration (NPT) were performed similarly as mentioned MDS. Over a period of 500 ps, GDP was pulled away from all four Rheb systems, and during this 500 ps, 500 configurations (also called reaction coordinates) were generated (see also Fig. 6H). Results were analyzed using the Grace plotting tool.

### Rheb acetylation assay

Flag or HA-tagged Rheb plasmids were transfected into HEK293T control and SIRT2 knockdown cells using PEI reagent. An empty plasmid with a C-terminal flag tag was used as a negative control. Cells were collected the day after the transfection and were washed with PBS prior to collecting via centrifugation of 3000 rpm for 5 min at 4 °C. 1 ml of 1% NP40 lysis buffer with final pH 7.4 (150 mM Tris-HCl, pH 8, 150 mM NaCl, 10% glycerol, 1% NP40) was used to lyse each sample at 4 °C for 1 h. After spinning down the samples at 13,000 rpm for 10 min at 4 °C, the supernatant was collected and normalized after using the Bradford reagent to determine the protein concentration. In total, 40 µl of each normalized sample was collected as input and was boiled at 95 °C for 5 min after 8 µl of six times loading dye was added to each input sample. The rest of the normalized samples were incubated with 20 µl of HA, Flag, or Acetyl Lysine beads at 4 °C overnight. The affinity beads were then washed with IP washing buffer (25 mM Tris, pH 8.0, 150 mM NaCl, 0.2% NP40) three times and were dried with a 1 ml syringe. Depending on the experiment, 50 µl of 1× loading dye or 35 µl of 2× loading dye were added to the dried beads, and each sample was boiled at 95 °C for 5 min. Standard western blot analysis was used to analyze the samples.

### Molecular simulation studies

#### All-atom simulations

A series of NPT simulations were performed on four systems, each with the same initial orientation of Rheb protein placed on the membrane. For Rheb, structure deposited as RCSB PDB: 1XTS was used (Yu et al, 2005). The systems differed mainly in protein mutations i.e., (a) Wild-type (WT), (b) K151A (mutant), (c) K151Q (Acetylated mimic) and (d) K151R (Deacetylated mimic). Lipid membranes were generated using CHARMM-GUI online server (Jo et al, 2008). The membrane composed of identical 80:20 mixture of POPC, and POPS for all four systems. The bilayer PHD system was energy minimized in a vacuum. Further, the systems were solvated with TIP3 water molecules (Mark and Nilsson, 2001) and Potassium and Chloride ions were added to neutralize each system and bring the salt concentration to 150 mM. Each system is simulated using GROMACS (Pronk et al, 2013) with CHARMM36m protein force field (Huang et al, 2017) and the CHARMM36 lipid force field (Klauda et al, 2010) with a 2-fs time step and visualized using VMD (Humphrey et al, 1996). Simulations were performed for 500 ns at *T* = 303 K and 1 atm pressure. Systems were minimized using steepest descent algorithm followed by the equilibration of 5–10 ns. Verlet Cutoff-scheme was used. The frequency to update the neighbor list (nstlist) was taken as 20. Periodic boundary conditions were used in all directions i.e., xyz was used for pbc. Temperature was coupled using Nose–Hoover thermostat (Evans and Holian, 1985). Pressure coupling was performed using Parrinello–Rahman barostat (Parrinello and Rahman, 1981) using a semi-isotropic coupling scheme. Non-bonded forces were calculated with a 12 Å cutoff (10 Å switching distance). Long-range electrostatic forces were calculated every other time step using the particle mesh Ewald method (Essmann et al, 1995). The system is maintained at a temperature of 303 K. The choice of the initial systems was guided by inputs from Orientations of proteins in membranes (OPM) database (Lomize et al, 2006) and from a published work by Gorfe group (Prakash and Gorfe, 2022). For carrying out the z-analysis for membrane association, in-house TCL script was used.

### Mass spectrometry sample preparation

Rheb was immunoprecipitated from control, and AGK-2-treated HeLa cells, and the samples were run in SDS-PAGE until the entire volume of the samples was stacked in SDS-Polyacrylamide gel. Gel was cut at the stacked area using a sharp, sterile blade for further processing. The gel piece was treated with 5 mM TCEP and alkylated with 50 mM iodoacetamide. Shrink the gel band with Acetonitrile and air-dry for a few minutes at room temperature, then digested with trypsin (1:50, trypsin/lysate ratio), for 16 h at 37 °C. Digests were speed vac for 1 h, and the pellet was dissolved in Buffer A (5% acetonitrile, 0.1% formic acid) and then cleaned using a C18 silica cartridge and dried using a speed vac. The dried pellet was resuspended in buffer A (5% acetonitrile, 0.1% formic acid). Analysis was performed using an EASY-nLC 1000 system (Thermo Fisher Scientific) coupled to a QExactive mass spectrometer (Thermo Fisher Scientific) equipped with a nanoelectrospray ion source. In all, 1.0 µg of the peptide mixture was resolved using a 15 cm PicoFrit column (360 µm outer diameter, 75 µm inner diameter, 10 µm tip) filled with 1.9 µm of C18-resin (Dr Maeisch, Germany). The peptides were loaded with buffer A and eluted with a 0–40% gradient of buffer B (95% acetonitrile, 0.1% formic acid) at a flow rate of 300 nl/min for 45 min. MS data was acquired using a data-dependent top10 method, dynamically choosing the most abundant precursor ions from the survey scan. RAW files obtained in this study were analyzed using Proteome Discoverer against the sequence given. For Sequest search, the precursor and fragment mass tolerances were set at 10 ppm and 0.5 Da, respectively. The protease used to generate peptides, i.e., enzyme specificity, was set for trypsin/P (cleavage at the C terminus of “K/R: unless followed by “P”) along with maximum missed cleavage value of two. Carbamidomethyl on cysteine as fixed modification and oxidation of methionine, N-terminal acetylation (K), and lysine Ub-amide were considered variable modifications for database search. Both peptide spectrum match and protein false discovery rate were set to 0.01 FDR.

### Histology

Heart tissue samples were collected in 10% neutral buffered formalin soon after euthanasia. After fixing samples (seventy-two hours incubation in formalin), formalin was withdrawn from the samples by putting the samples overnight under a running water tap (minimum 9 h.). When the formalin was eliminated, samples were subjected to dehydration in alcohol and then xylene, and later covered with paraffin wax by using an automated tissue processor (Leica TP1020, Germany). Then, samples were embedded using paraffin wax to make blocks to ensure easy sectioning (Leica EG 1150H, Germany). These blocks were then solidified using a cooling machine (Leica EG 1150 C, Germany). Sections were cut (5 µm thickness) and put on glass slides with the help of a microtome (Leica RM2245, Germany). Wheat Germ Agglutinin (WGA) (5 µg/ml) stain was used to evaluate the cross-sectional area of cardiomyocytes.

### Click chemistry

Flag-Rheb plasmids were transfected into HEK293T control and SIRT2 knockdown cells using PEI. Twenty-four hours later, half of the samples were treated with 50 μM Alk14 for 6 h before collecting. The samples that were not treated with Alk14 served as negative controls. Samples were collected via centrifugation at 3000 rpm for 5 min at 4 °C. In total, 1 ml of 1% NP40 lysis buffer with final pH 7.4 (150 mM Tris-HCl, pH 8, 150 mM NaCl, 10% glycerol, 1% NP40) was used to lyse each sample at 4 °C for 1 h. After spinning down the samples with 13,000 rpm for 10 min at 4 °C, the supernatant was collected and normalized after using the Bradford reagent to determine the protein concentration. In all, 40 μL of each normalized sample was collected as input and was boiled at 95 °C for 5 min after 8 μL of six times loading dye was added to each input sample. The rest of the normalized sample was incubated with 20 μL of Flag beads at 4 °C overnight. The flag beads were then washed with IP washing buffer (25 mM Tris, pH 8.0, 150 mM NaCl, 0.2% NP40) three times and were dried with a 1 ml syringe. Each sample was then added with 24 μL of click chemistry mixture (20 μL IP washing buffer, 1 μL of 40 mM CuSO4, 1 μL of 10 mM TBTA, 1 μL of 2 mM TAMARA-azide, and 1 μL of 40 mM TCEP). The samples were then incubated in the dark for 30 min, and 12 μL of 6X SDS loading dye were added to each sample. After boiling at 95 °C for 5 min, samples were divided in half, and half of the samples were added with 400 mM hydroxylamine and boiled for another 10 min. All samples were then analyzed with SDS-PAGE, and the palmitoylation signal was checked using the Rhodamine channel of the ChemiDoc Imaging System.

### Quantification and statistical analysis

All statistical analyses and graphical representations of data were performed using GraphPad Prism version 8.4.2. For pair-wise comparisons, if the data values follow the Gaussian distribution, then a parametric *t-test* with Welch’s correction was used. If the data values did not follow a Gaussian distribution, then the nonparametric Mann–Whitney test was used. One-way ANOVA and two-way ANOVA were used for comparisons between more than two groups, as recommended by GraphPad Prism version 8.4.2 software. ZEN-Black software was used for confocal image processing and analysis. Image Lab software (Bio-Rad) was used for processing western blot images acquired from the Chemi-Doc machine, and ImageJ software was used to quantify western blots and confocal images.

## Supplementary information

Peer Review File

Source data Fig. 1

Source data Fig. 2

Source data Fig. 3

Source data Fig. 4

Source data Fig. 6

Source data Fig. 7

Source data Fig. 8

Source data Fig. 9

Expanded View Figures

## Data Availability

This study includes no data deposited in external repositories. The source data of this paper are collected in the following database record: biostudies:S-SCDT-10_1038-S44319-026-00724-5.
