# Author Correction: Sirtuin 2 inhibits global protein synthesis via Rheb-GTPase degradation

**DOI:** 10.1038/s44319-026-00796-3

**Published:** 2026-05-05

**Authors:** Amarjeet Shrama, Yanlin Zi, Anwit Shriniwas Pandit, Kirtika Jha, Vikrant Kumar Sinha, Dimple Nagesh, Bhoomika Shivanaiah, Venkatraman Ravi, Souvik Ghosh, Danish Khan, Arathi Bangalore Prabhashankar, Thoniparambil Sunil Sumi, Satish Rajpurohit, Sunayana Ningaraju, Sukanya Raghu, Anand Srivastava, Mahavir Singh, Hening Lin, Nagalingam R Sundaresan

**Affiliations:** 1 Department of Microbiology and Cell Biology, Indian Institute of Science, Bengaluru, Karnataka 560012 India; 2 Department of Chemistry and Chemical Biology, Cornell University, Ithaca, NY 14853 USA; 3 Molecular Biophysics Unit, Indian Institute of Science, Bengaluru, Karnataka 560012 India; 4 Howard Hughes Medical Institute; Department of Medicine and Department of Chemistry, The University of Chicago, Chicago, IL 60637 USA

## Abstract

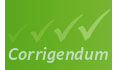

**Correction to:** *EMBO Reports* (2026). 10.1038/s44319-026-00724-5 | Published online 11 March 2026

**A new citation is added to manuscript**

**The Reference list is updated**

A new citation is added to the Discussion section, at the end of the following sentence in line 547. Citation is in bold:

Our companion manuscript demonstrates that SIRT2 deacetylates 4EBP1, promoting its stabilization and consequent suppression of translation initiation **(preprint:** Zi et al., 2025**)**.

The following reference is added to the Reference list:
