# Supplementary figures and images for: Sirtuin 2 inhibits global protein synthesis via Rheb-GTPase degradation (part 1 of 2)
Source: EMBO Rep. 2026 Mar 11;27(11):3001–34. doi: 10.1038/s44319-026-00724-5 (PMC13261059; doi:10.1038/s44319-026-00724-5)

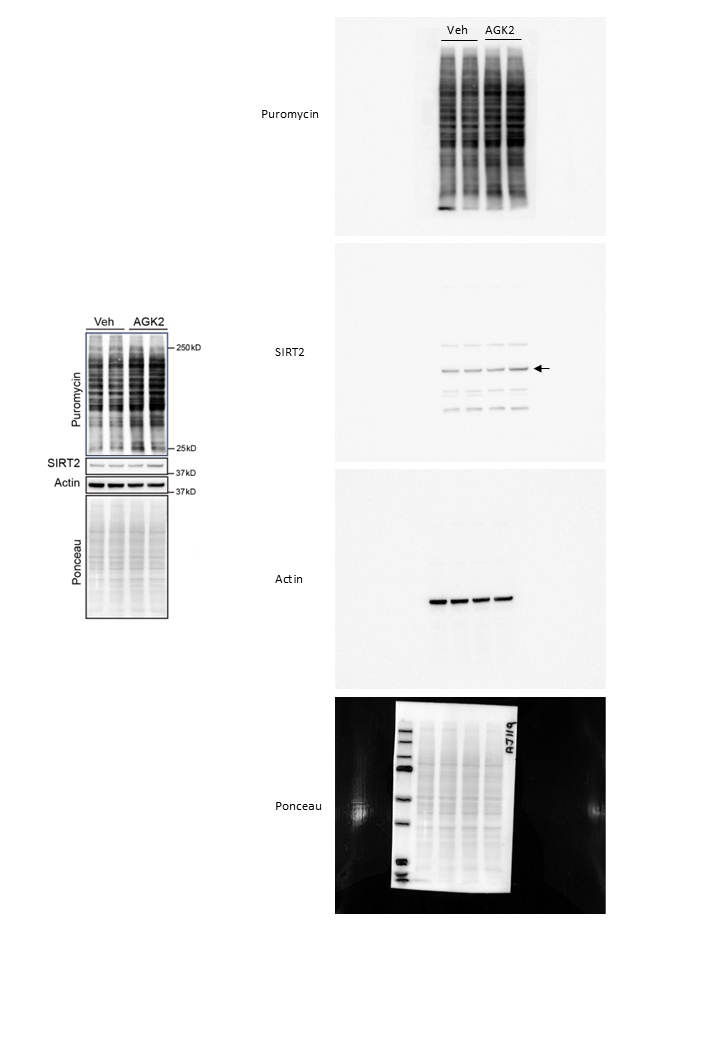

Supplement: Supplementary file 2 — Source data Fig. 1 [file 44319_2026_724_MOESM2_ESM.zip › Figure 1/1A/1A.tif]

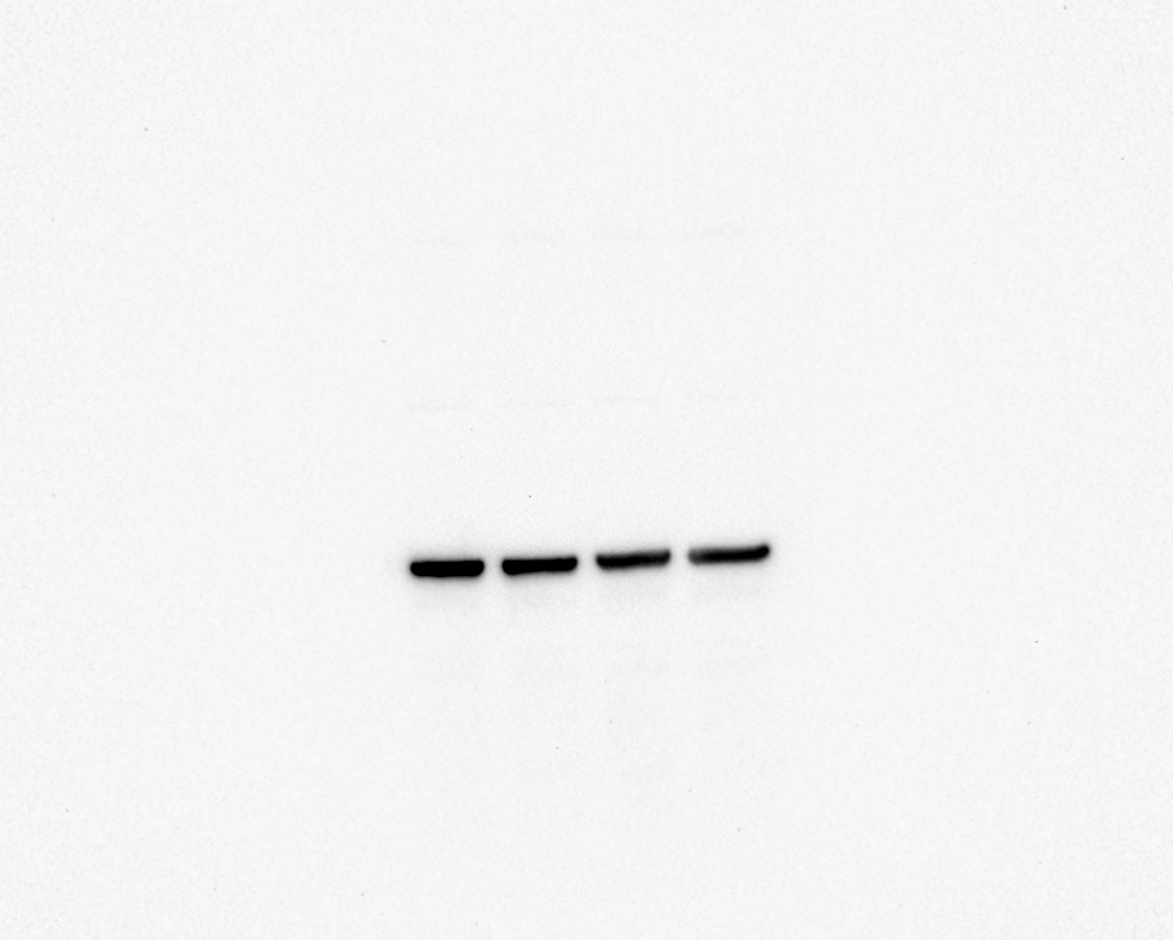

Supplement: Supplementary file 2 — Source data Fig. 1 [file 44319_2026_724_MOESM2_ESM.zip › Figure 1/1A/fig.1A Actin.tif]

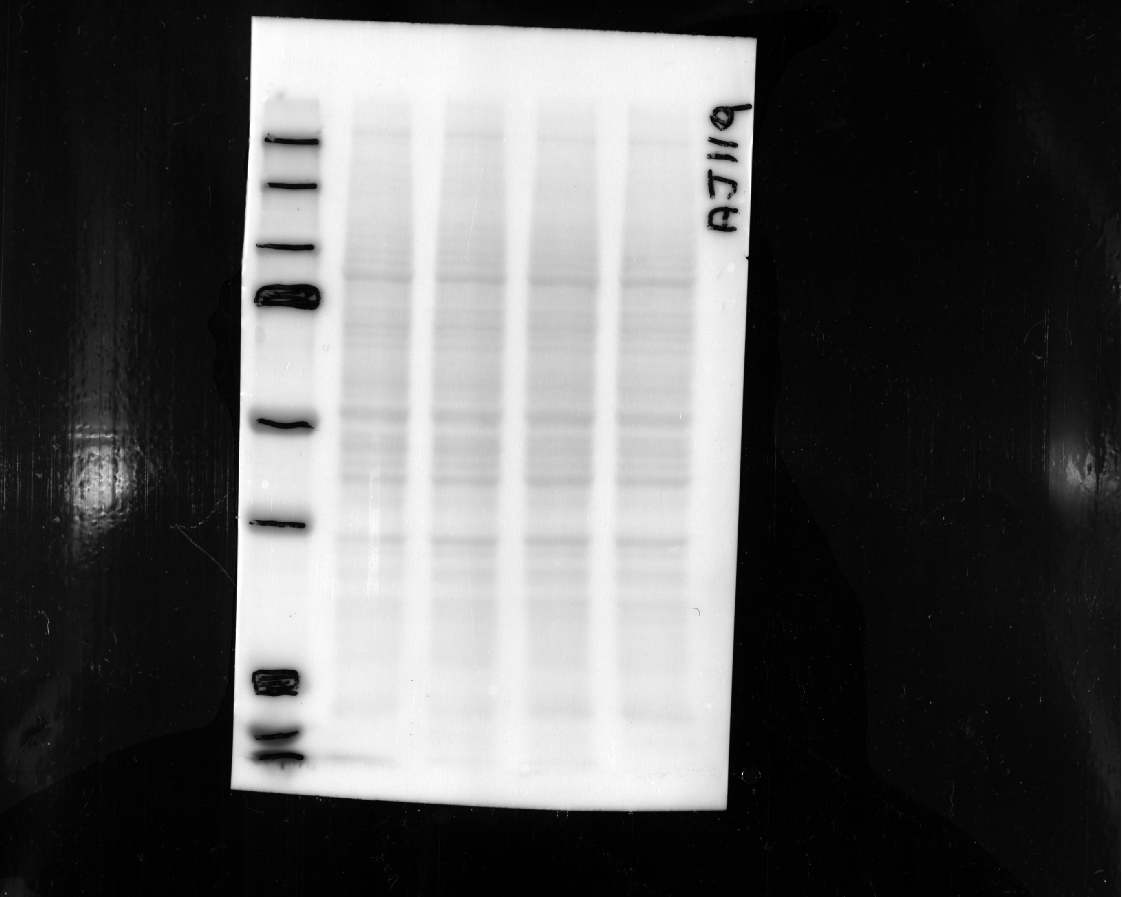

Supplement: Supplementary file 2 — Source data Fig. 1 [file 44319_2026_724_MOESM2_ESM.zip › Figure 1/1A/fig. 1A Ponceau.tif]

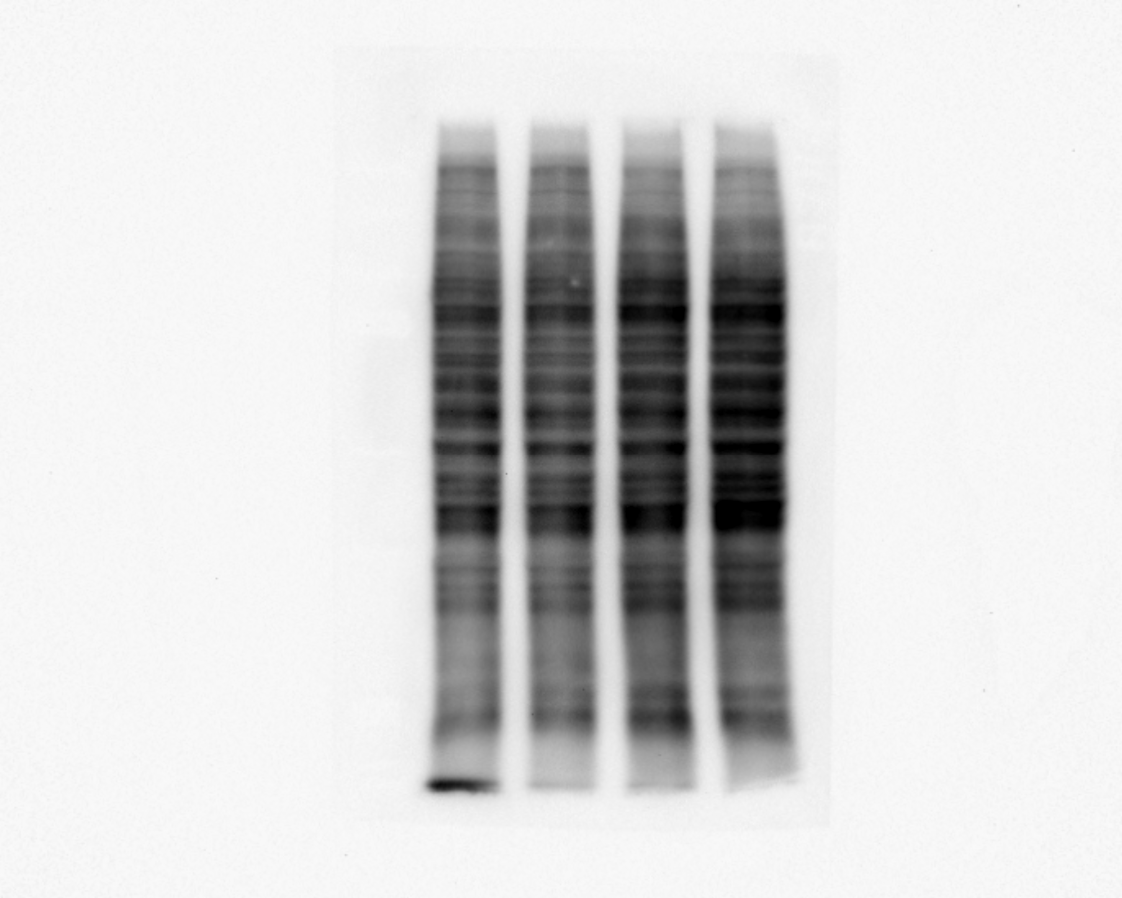

Supplement: Supplementary file 2 — Source data Fig. 1 [file 44319_2026_724_MOESM2_ESM.zip › Figure 1/1A/Fig. 1A Puromycin.tif]

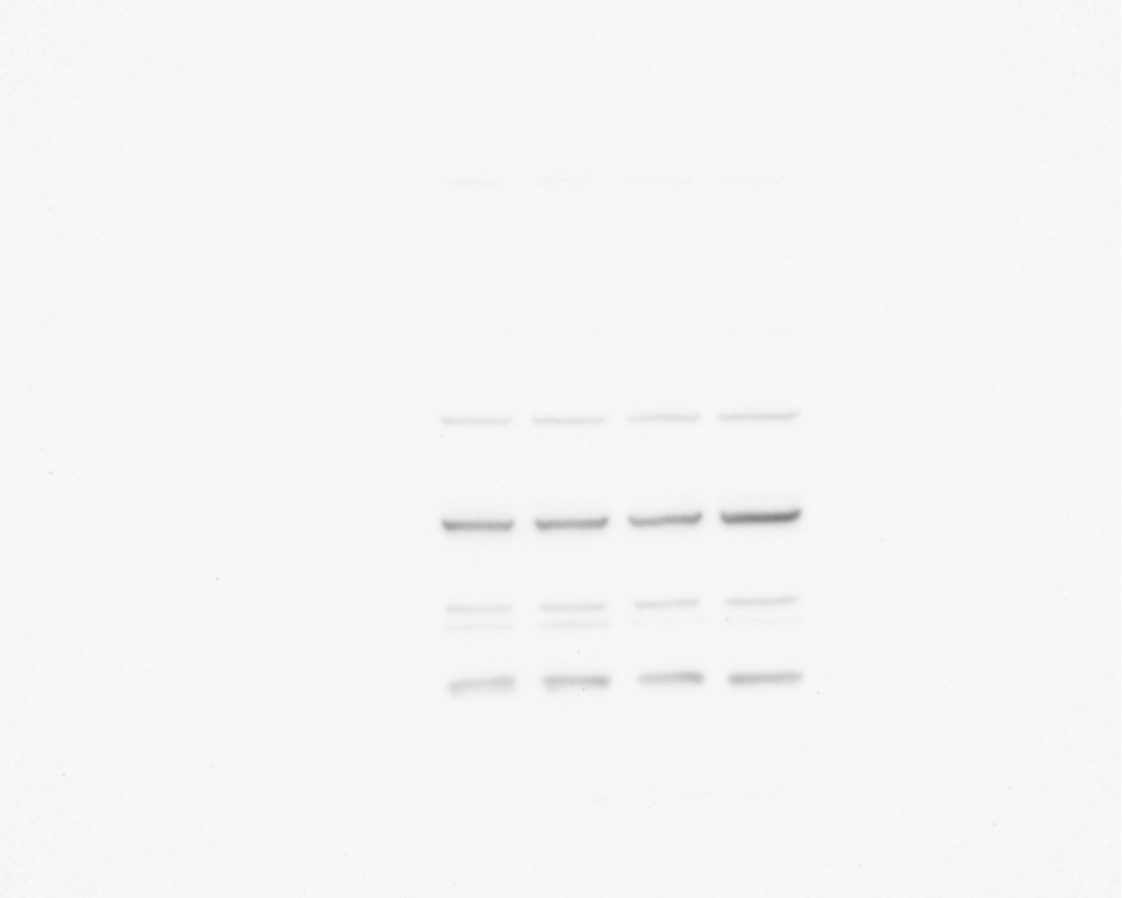

Supplement: Supplementary file 2 — Source data Fig. 1 [file 44319_2026_724_MOESM2_ESM.zip › Figure 1/1A/fig. 1A Sirt2.tif]

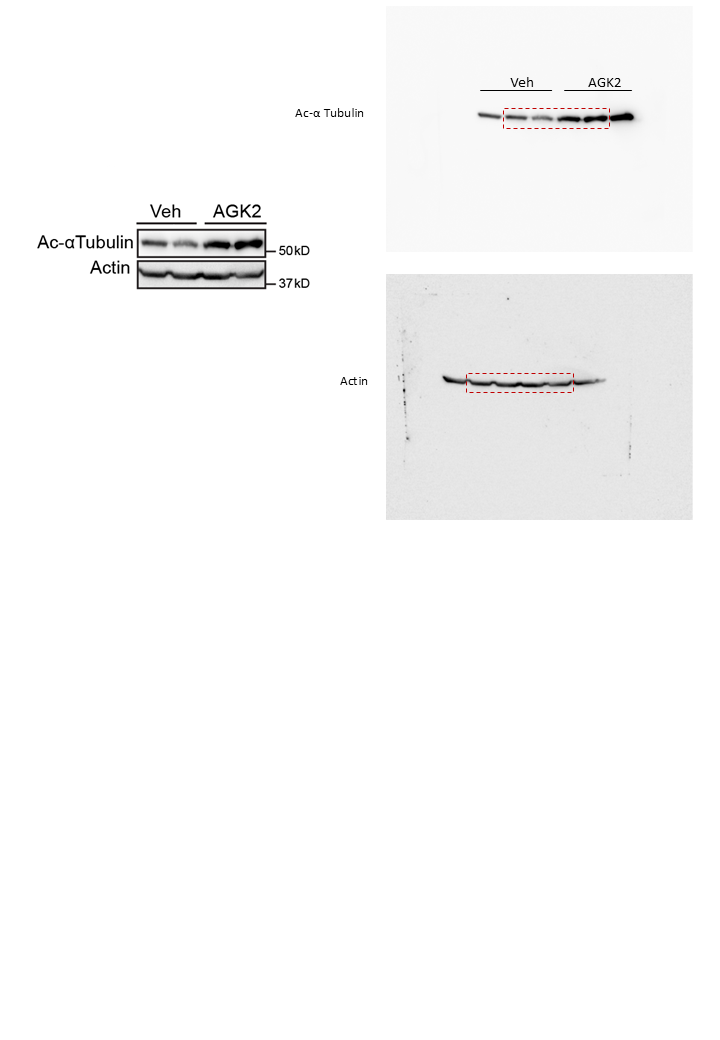

Supplement: Supplementary file 2 — Source data Fig. 1 [file 44319_2026_724_MOESM2_ESM.zip › Figure 1/1C/1C.tif]

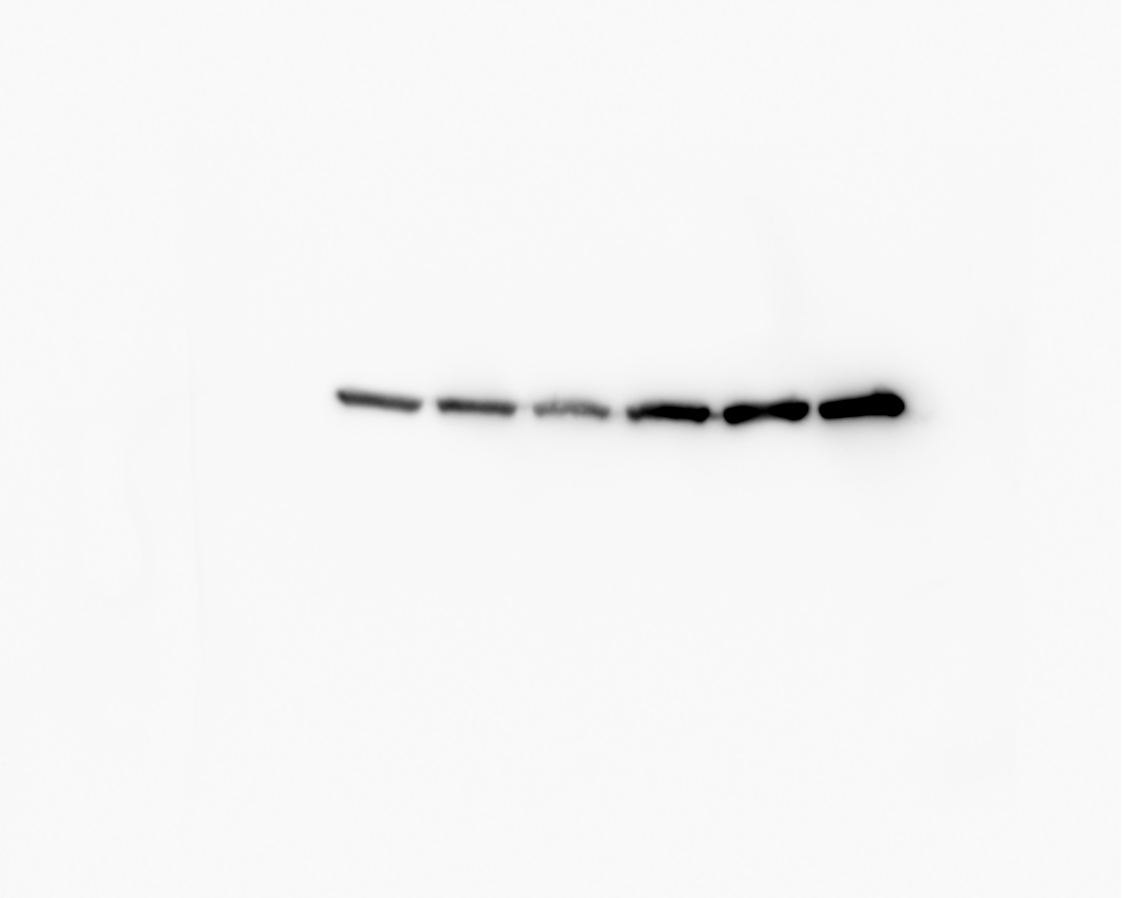

Supplement: Supplementary file 2 — Source data Fig. 1 [file 44319_2026_724_MOESM2_ESM.zip › Figure 1/1C/fig.1C Ac-Tubulin.tif]

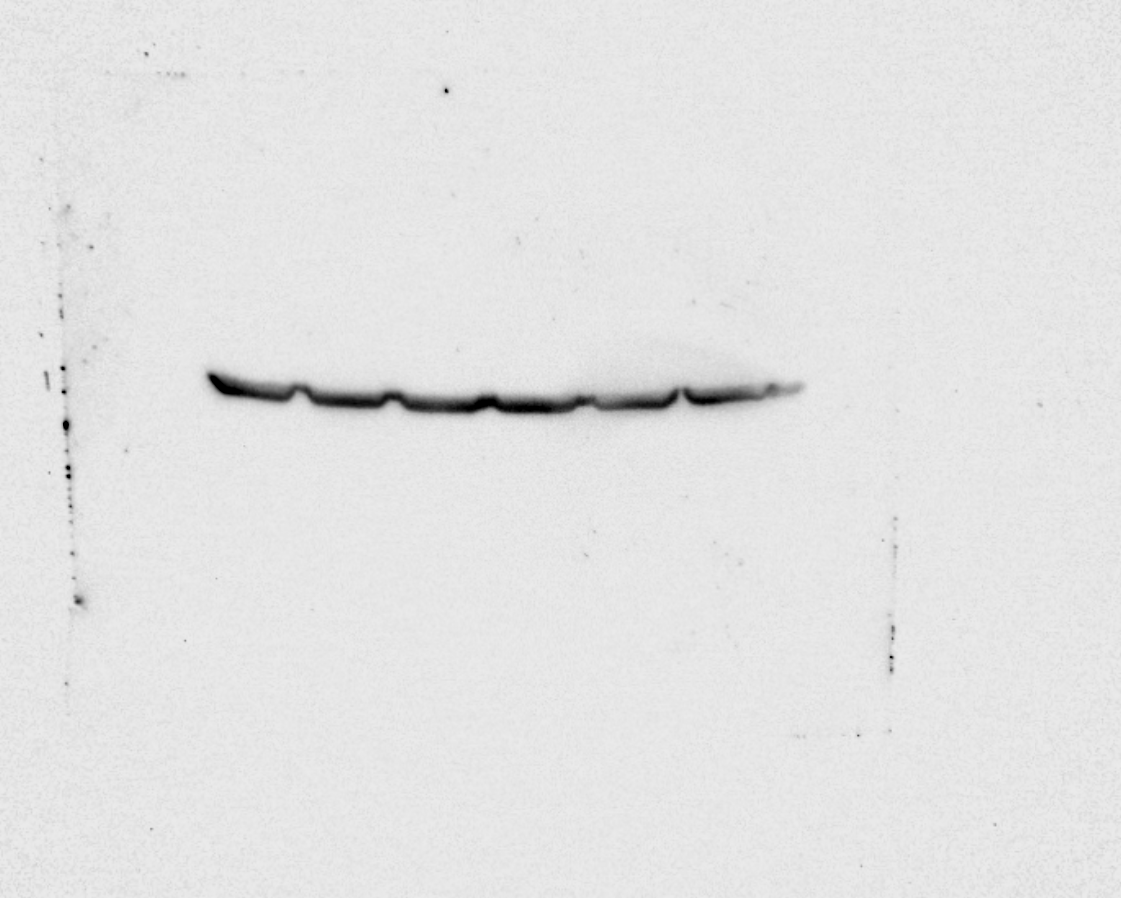

Supplement: Supplementary file 2 — Source data Fig. 1 [file 44319_2026_724_MOESM2_ESM.zip › Figure 1/1C/fig. 1C Actin.tif]

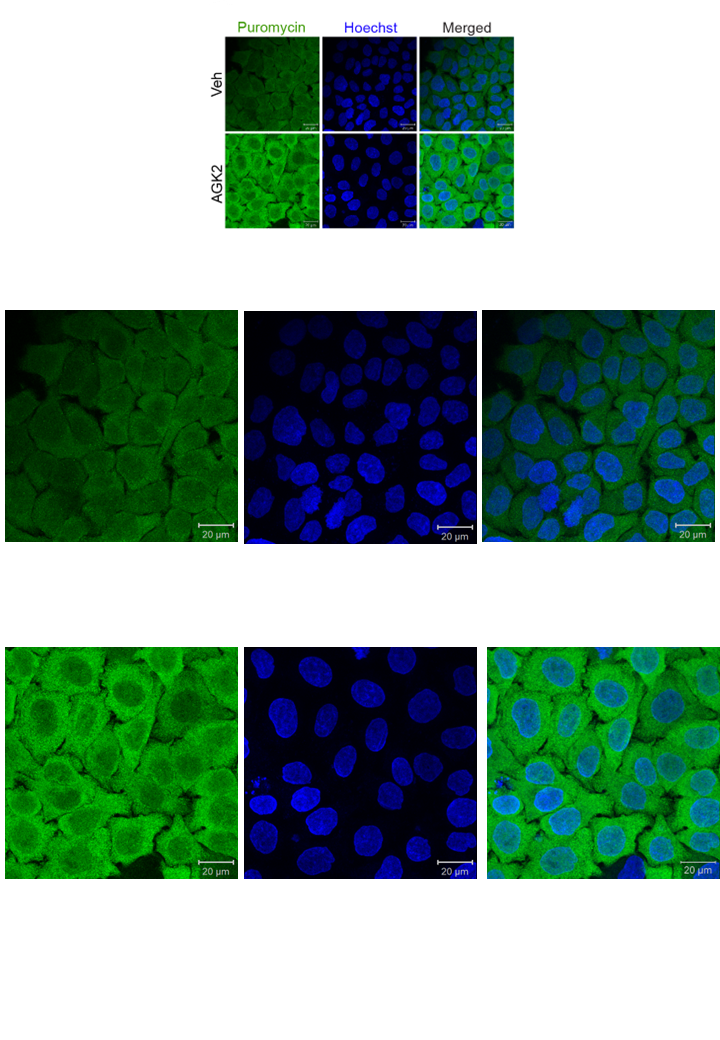

Supplement: Supplementary file 2 — Source data Fig. 1 [file 44319_2026_724_MOESM2_ESM.zip › Figure 1/1D/1D.tif]

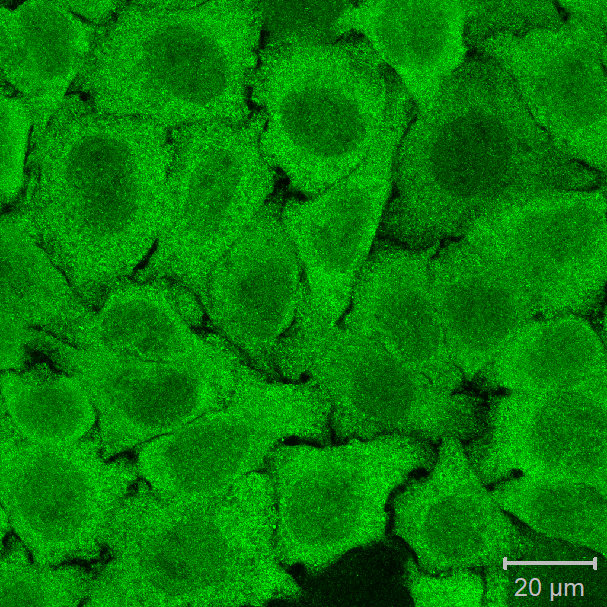

Supplement: Supplementary file 2 — Source data Fig. 1 [file 44319_2026_724_MOESM2_ESM.zip › Figure 1/1D/fig.1D AGK2 Puromycin.tif]

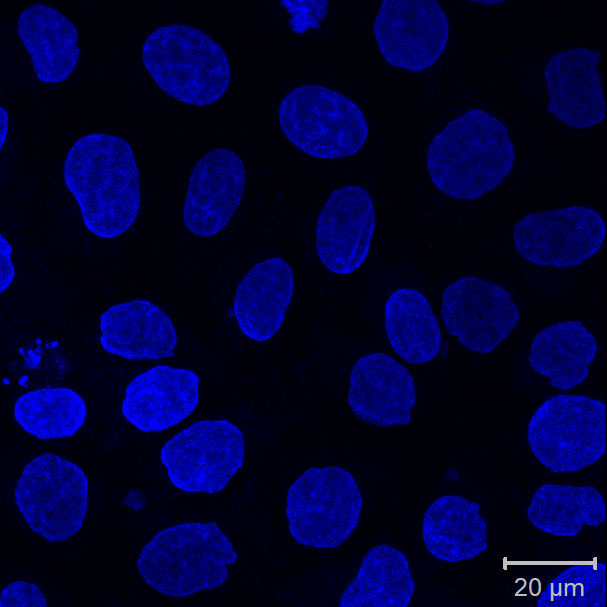

Supplement: Supplementary file 2 — Source data Fig. 1 [file 44319_2026_724_MOESM2_ESM.zip › Figure 1/1D/fig.1D AGK2 DAPI.tif]

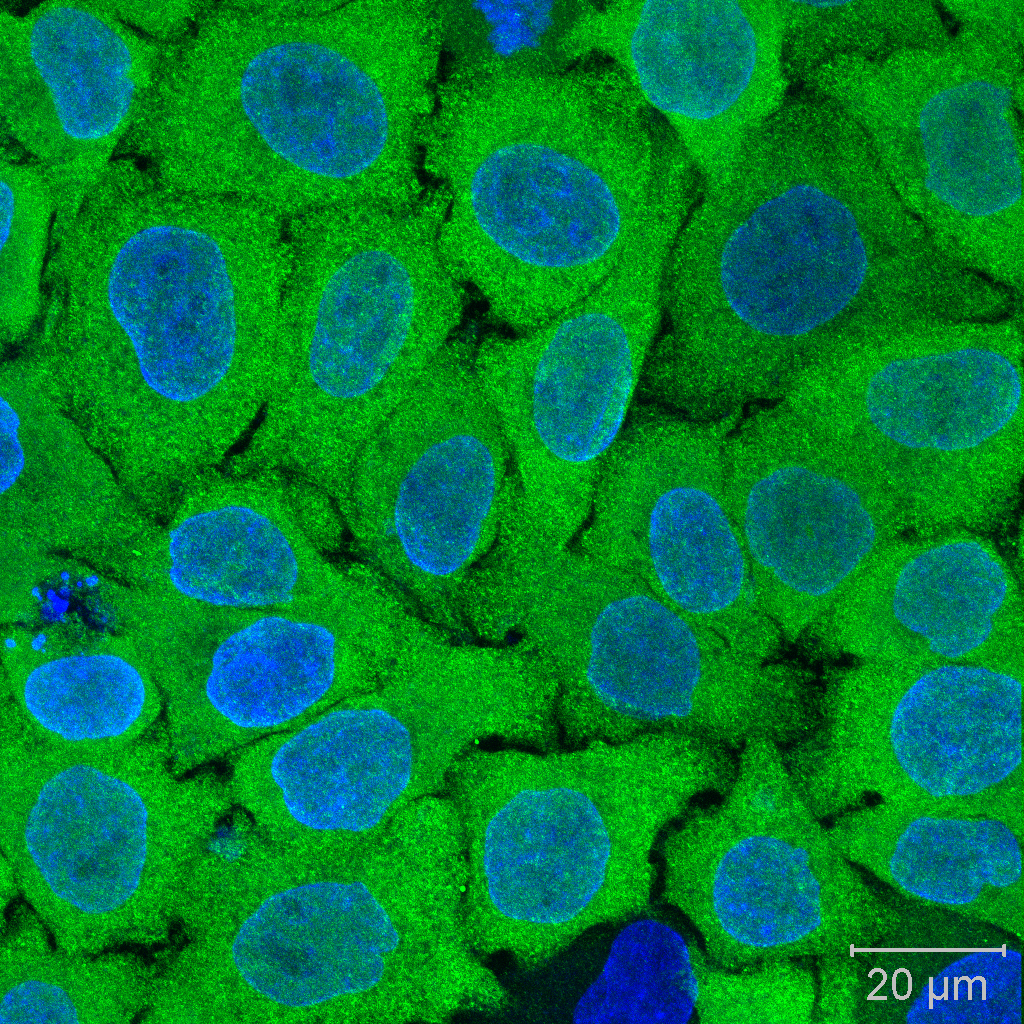

Supplement: Supplementary file 2 — Source data Fig. 1 [file 44319_2026_724_MOESM2_ESM.zip › Figure 1/1D/fig.1D AGK2 Puromycin DAPI merged.tif]

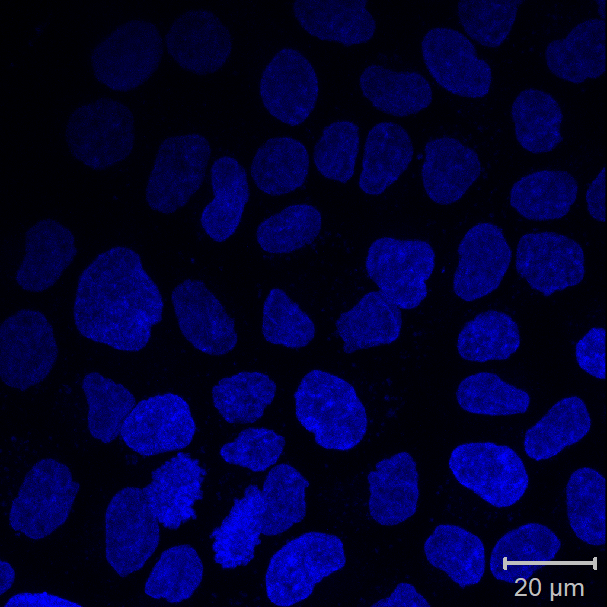

Supplement: Supplementary file 2 — Source data Fig. 1 [file 44319_2026_724_MOESM2_ESM.zip › Figure 1/1D/fig.1D DMSO DAPI.tif]

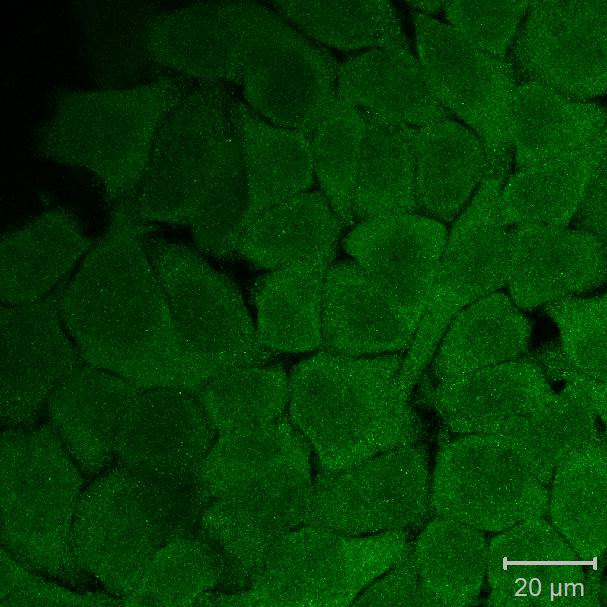

Supplement: Supplementary file 2 — Source data Fig. 1 [file 44319_2026_724_MOESM2_ESM.zip › Figure 1/1D/fig.1D DMSO Puromycin.tif]

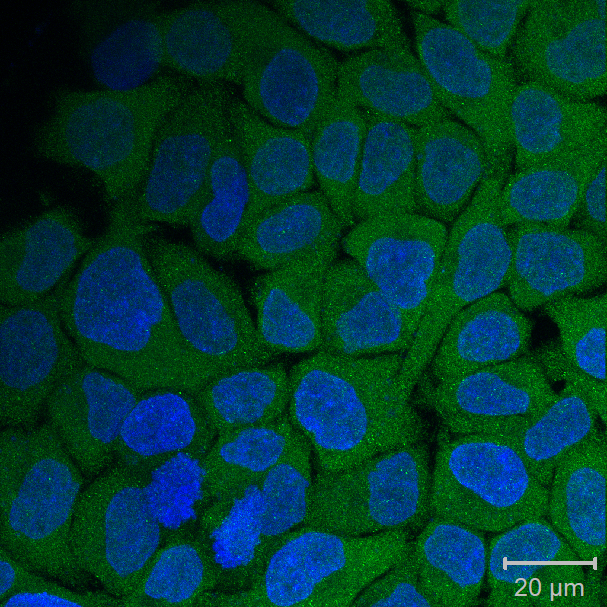

Supplement: Supplementary file 2 — Source data Fig. 1 [file 44319_2026_724_MOESM2_ESM.zip › Figure 1/1D/fig.1D DMSO Puromycin DAPI merged.tif]

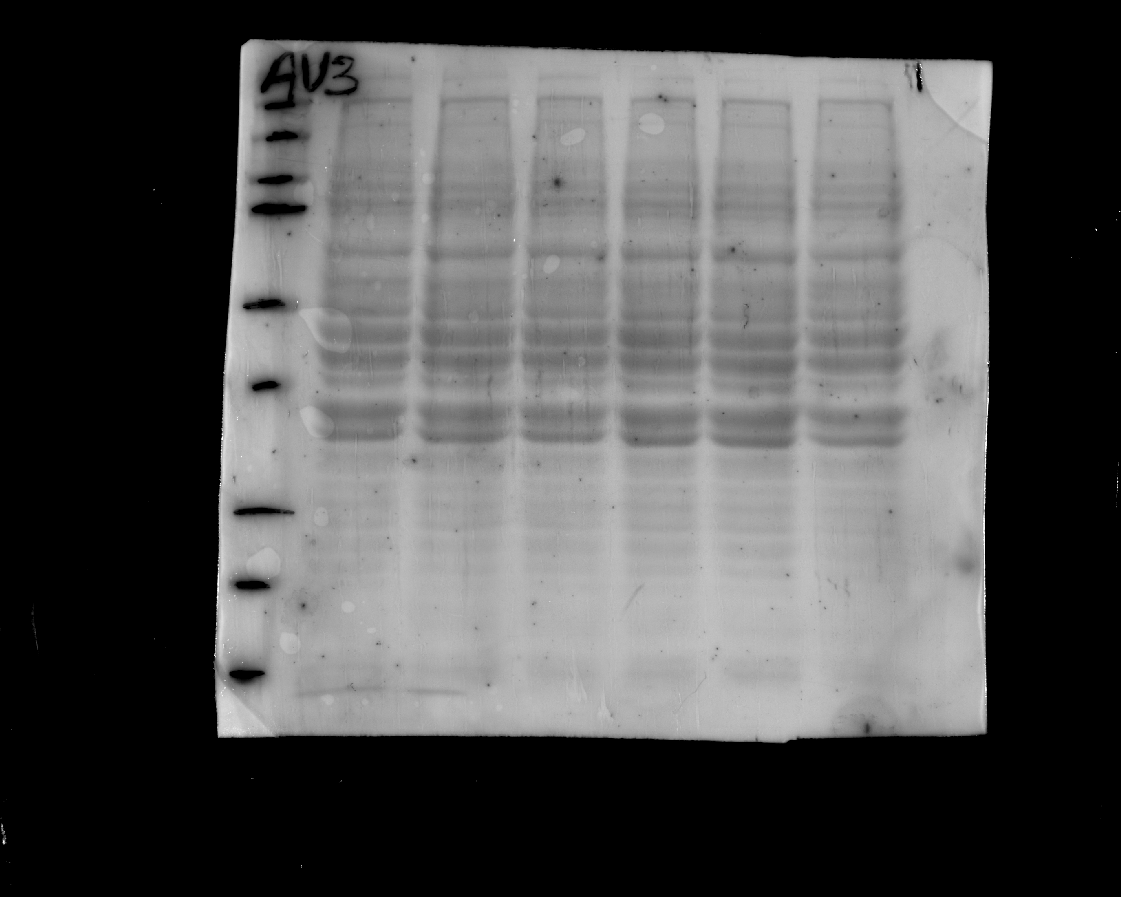

Supplement: Supplementary file 2 — Source data Fig. 1 [file 44319_2026_724_MOESM2_ESM.zip › Figure 1/1F/fig. 1F Ponceau.tif]

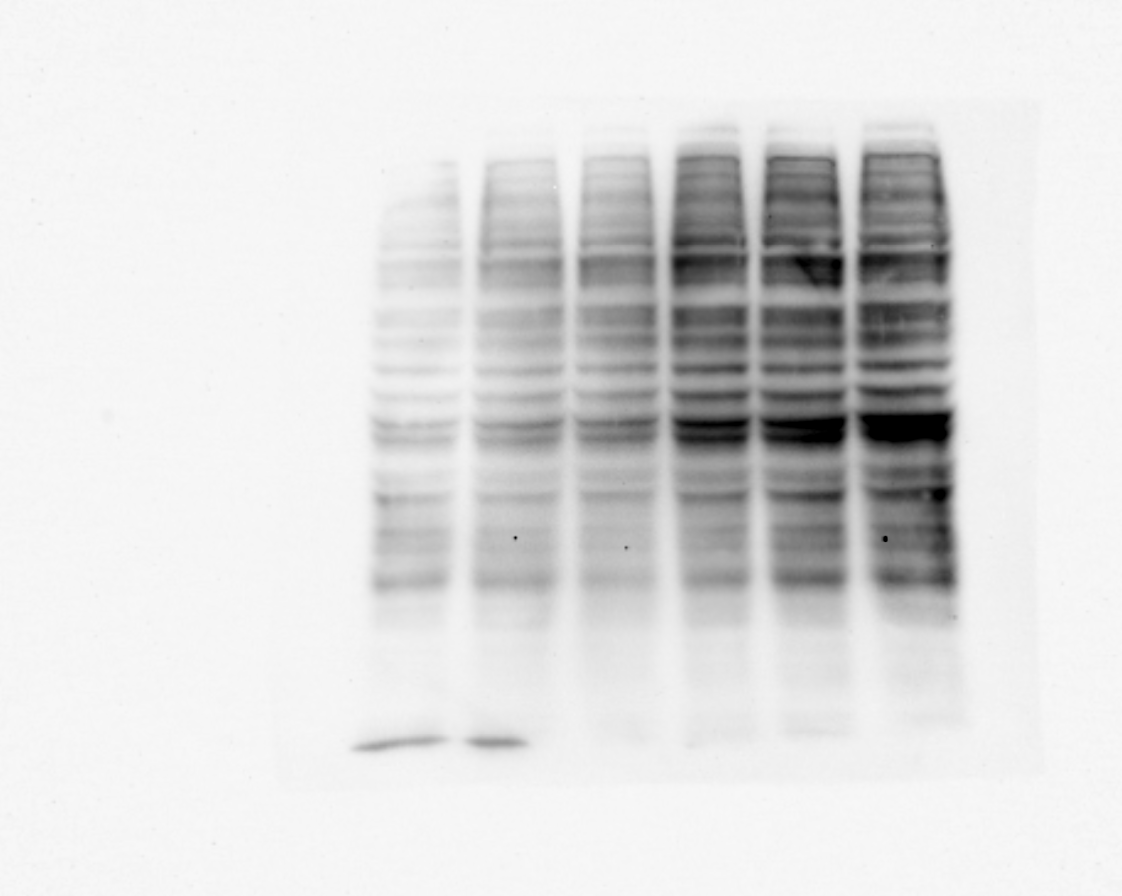

Supplement: Supplementary file 2 — Source data Fig. 1 [file 44319_2026_724_MOESM2_ESM.zip › Figure 1/1F/fig. 1F Puromycin.tif]

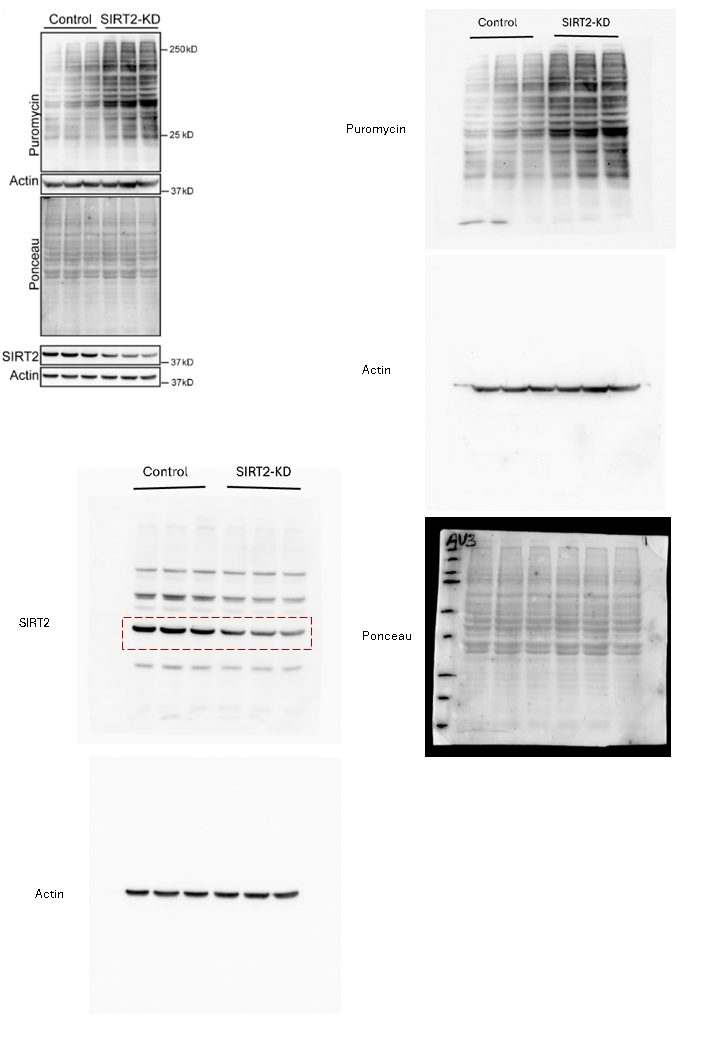

Supplement: Supplementary file 2 — Source data Fig. 1 [file 44319_2026_724_MOESM2_ESM.zip › Figure 1/1F/1F.tif]

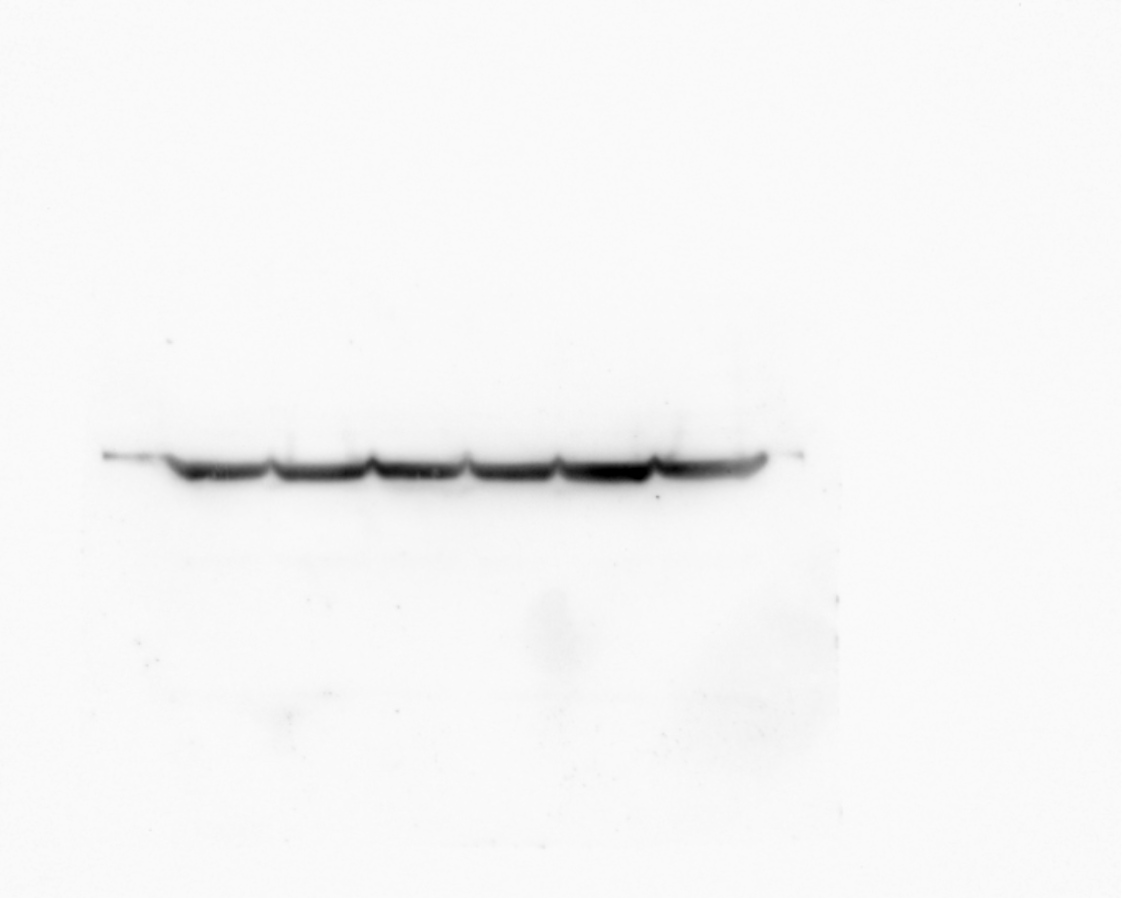

Supplement: Supplementary file 2 — Source data Fig. 1 [file 44319_2026_724_MOESM2_ESM.zip › Figure 1/1F/fig. 1F actin.tif]

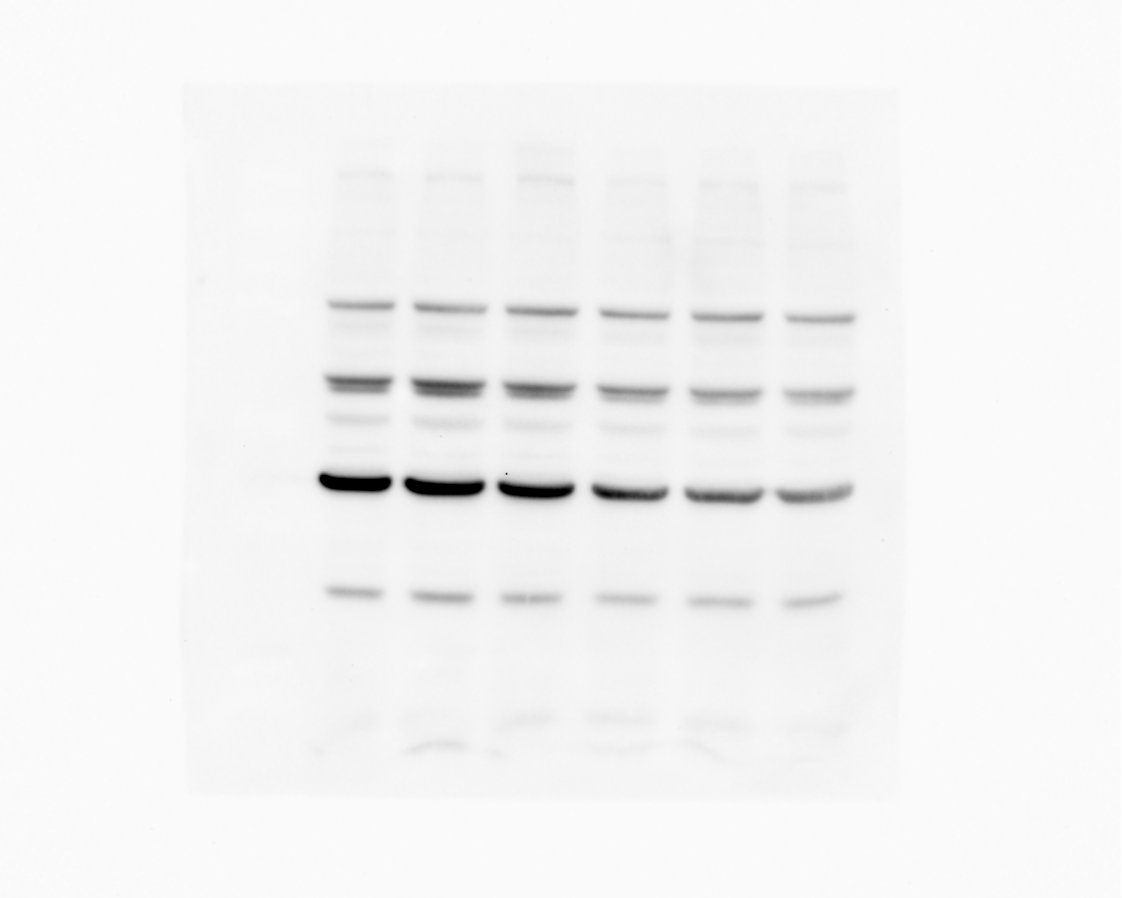

Supplement: Supplementary file 2 — Source data Fig. 1 [file 44319_2026_724_MOESM2_ESM.zip › Figure 1/1F/fig. 1F sirt2.tif]

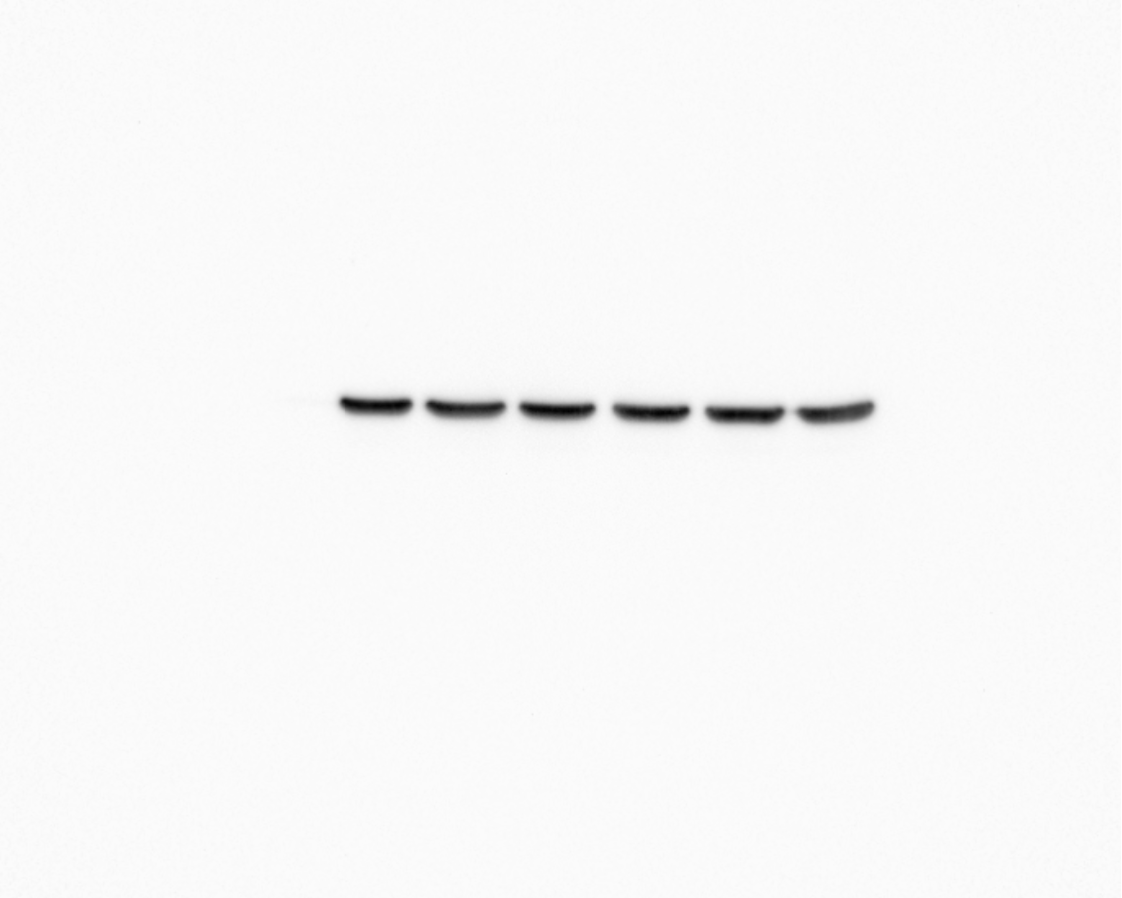

Supplement: Supplementary file 2 — Source data Fig. 1 [file 44319_2026_724_MOESM2_ESM.zip › Figure 1/1F/fig. 1F actin .tif]

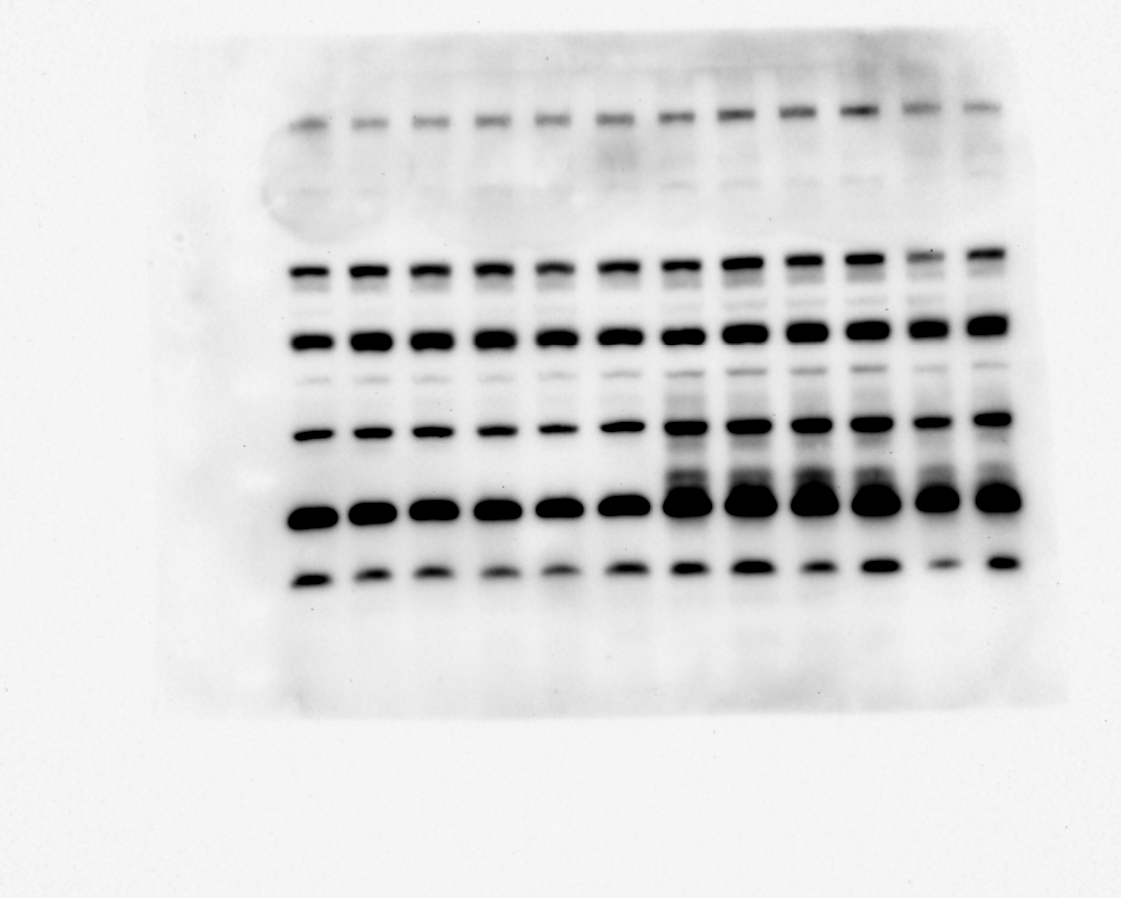

Supplement: Supplementary file 2 — Source data Fig. 1 [file 44319_2026_724_MOESM2_ESM.zip › Figure 1/1G/fig.1G sirt2.tif]

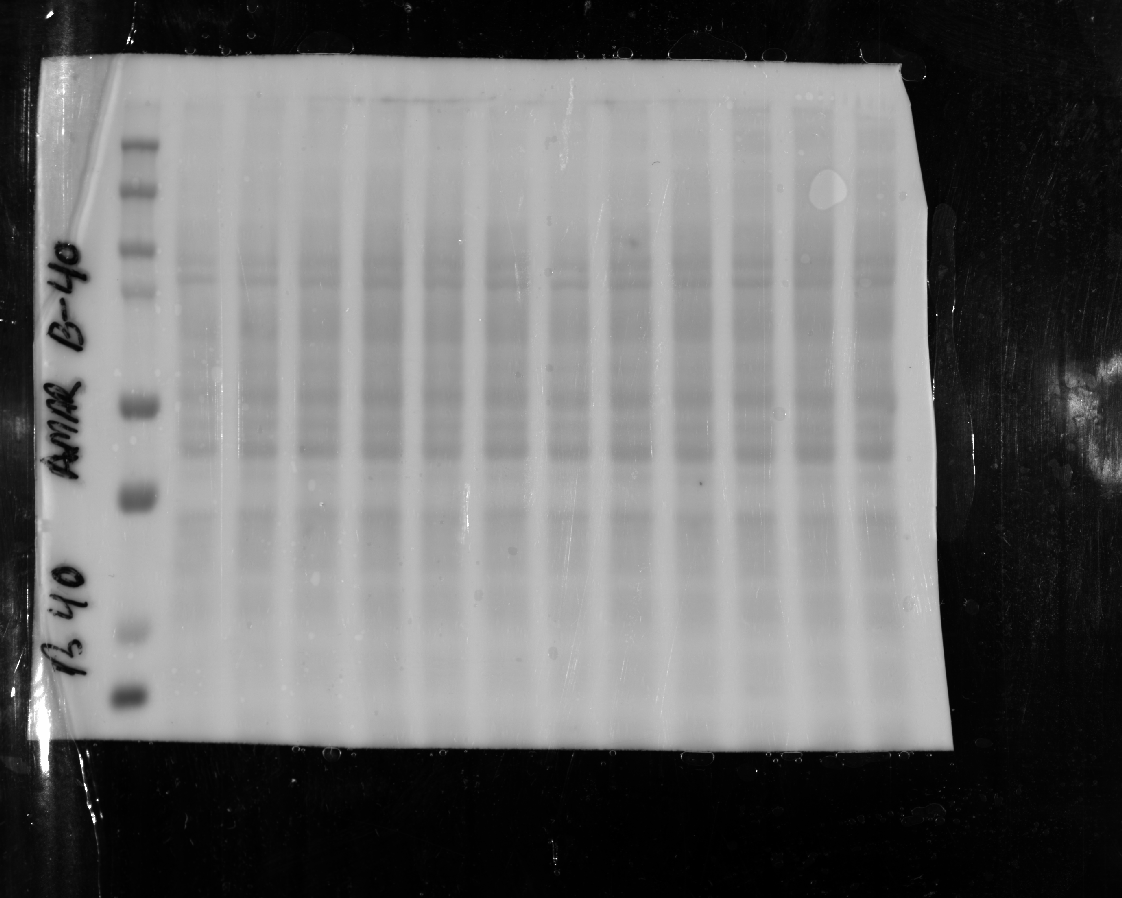

Supplement: Supplementary file 2 — Source data Fig. 1 [file 44319_2026_724_MOESM2_ESM.zip › Figure 1/1G/fig.1G Ponceau.tif]

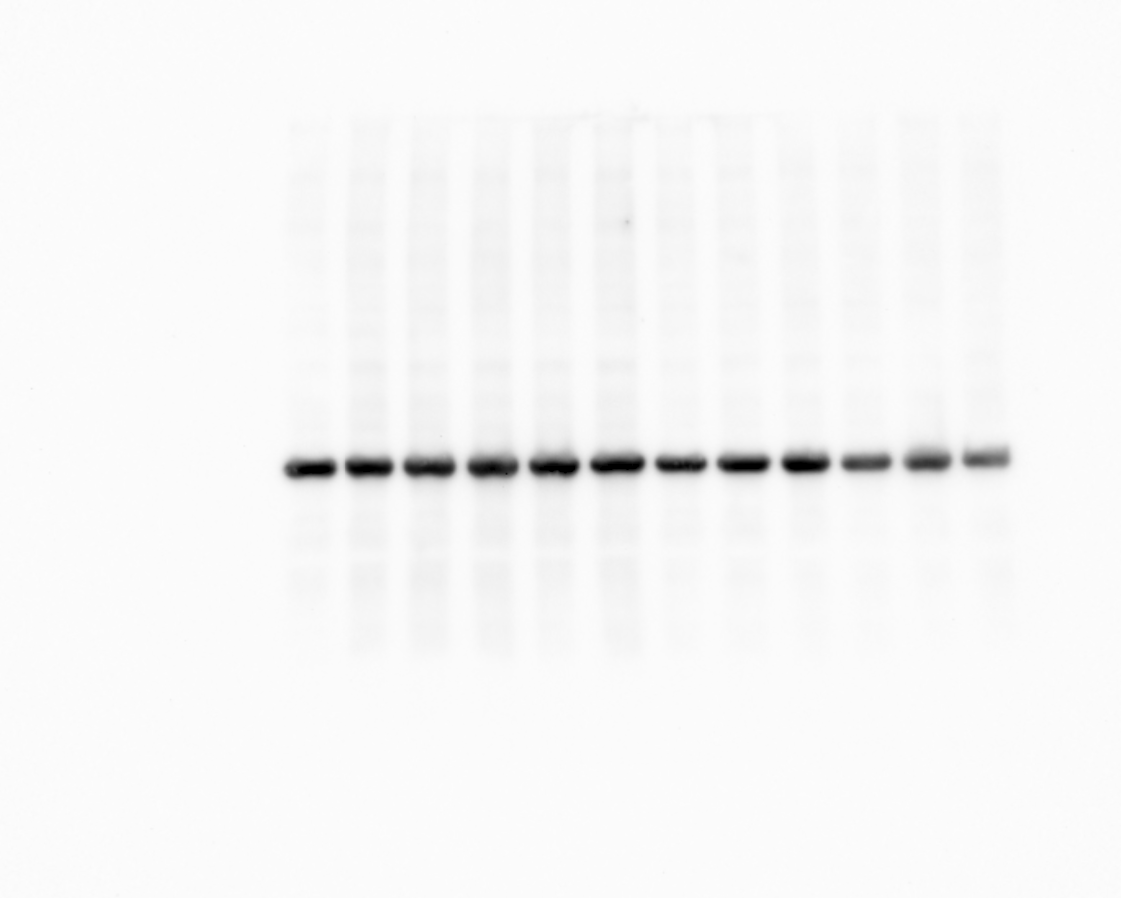

Supplement: Supplementary file 2 — Source data Fig. 1 [file 44319_2026_724_MOESM2_ESM.zip › Figure 1/1G/fig. 1G Actin.tif]

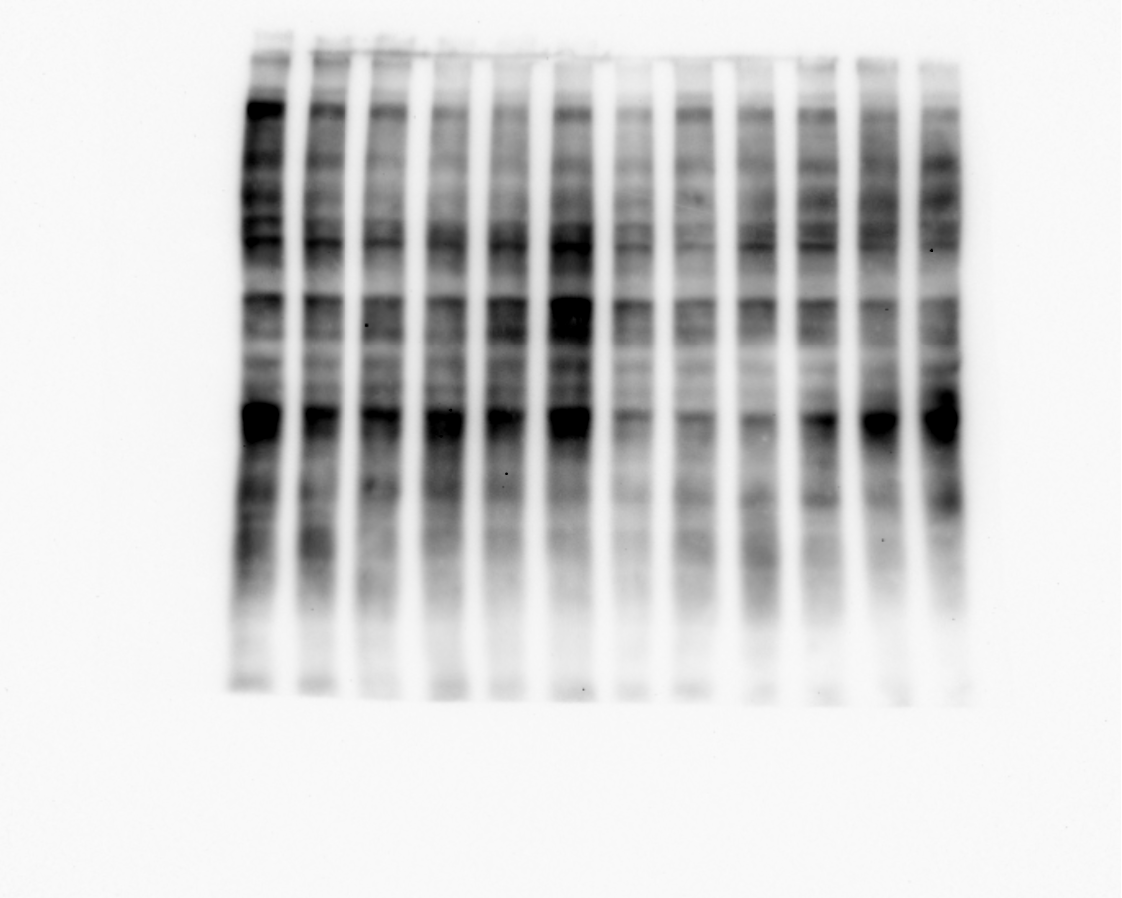

Supplement: Supplementary file 2 — Source data Fig. 1 [file 44319_2026_724_MOESM2_ESM.zip › Figure 1/1G/fig, 1G Puromycin.tif]

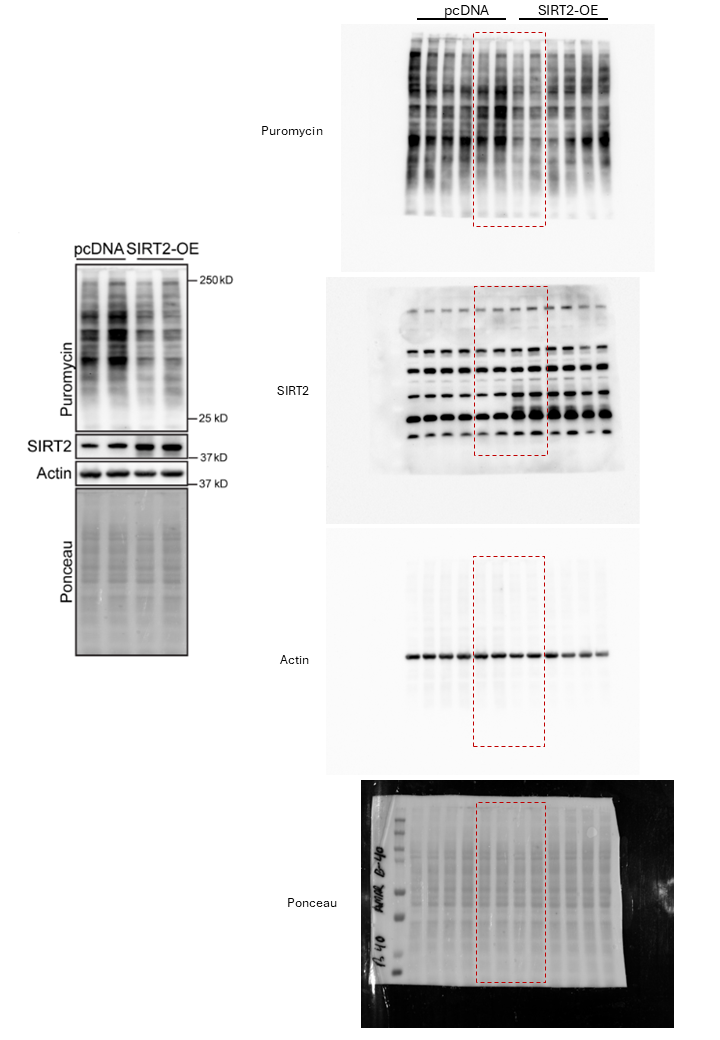

Supplement: Supplementary file 2 — Source data Fig. 1 [file 44319_2026_724_MOESM2_ESM.zip › Figure 1/1G/1G.tif]

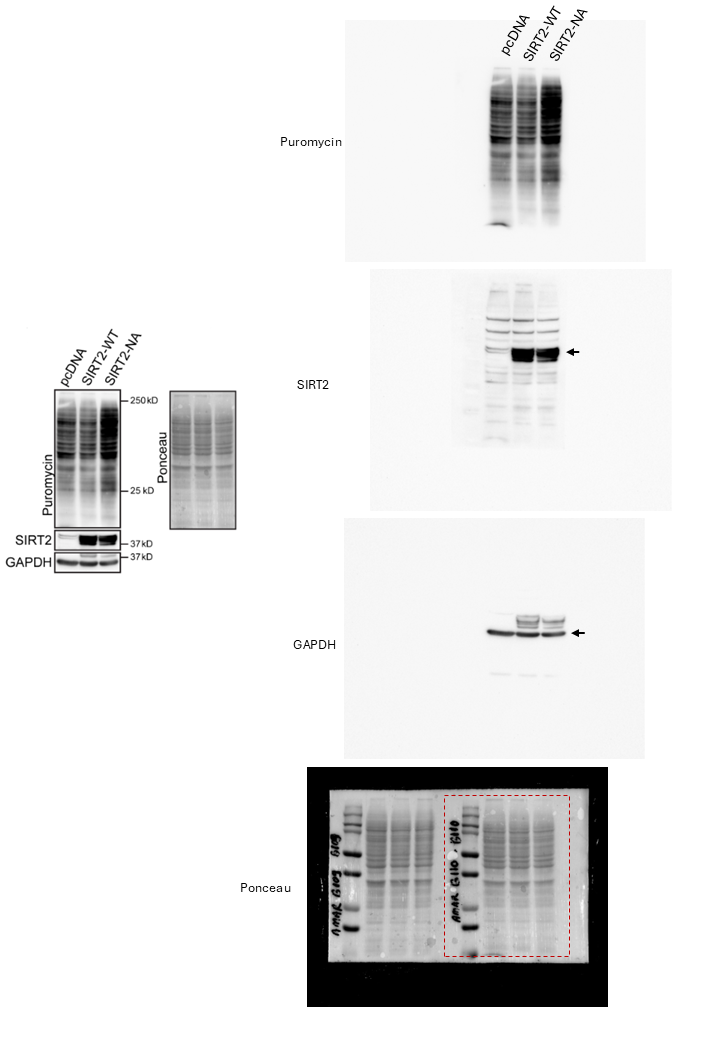

Supplement: Supplementary file 2 — Source data Fig. 1 [file 44319_2026_724_MOESM2_ESM.zip › Figure 1/1J/1J.tif]

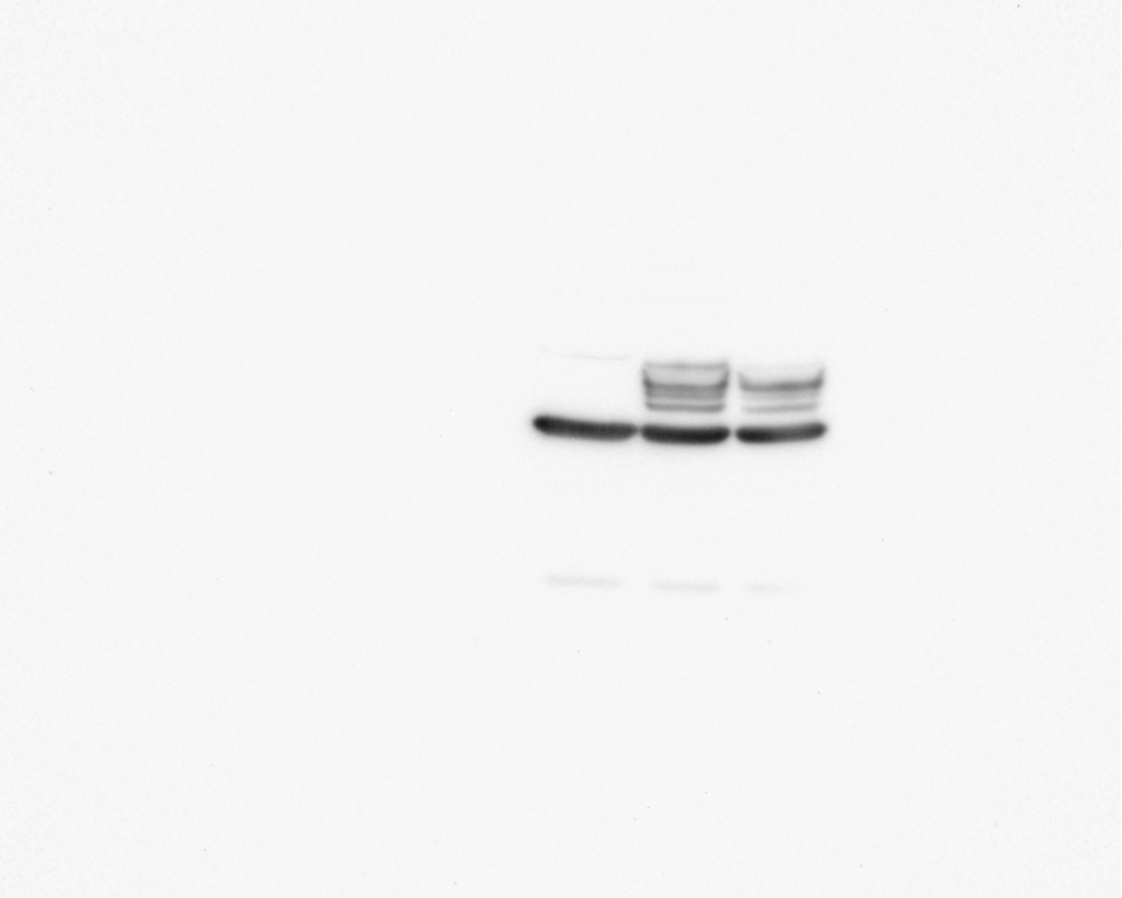

Supplement: Supplementary file 2 — Source data Fig. 1 [file 44319_2026_724_MOESM2_ESM.zip › Figure 1/1J/fig. 1J GAPDH.tif]

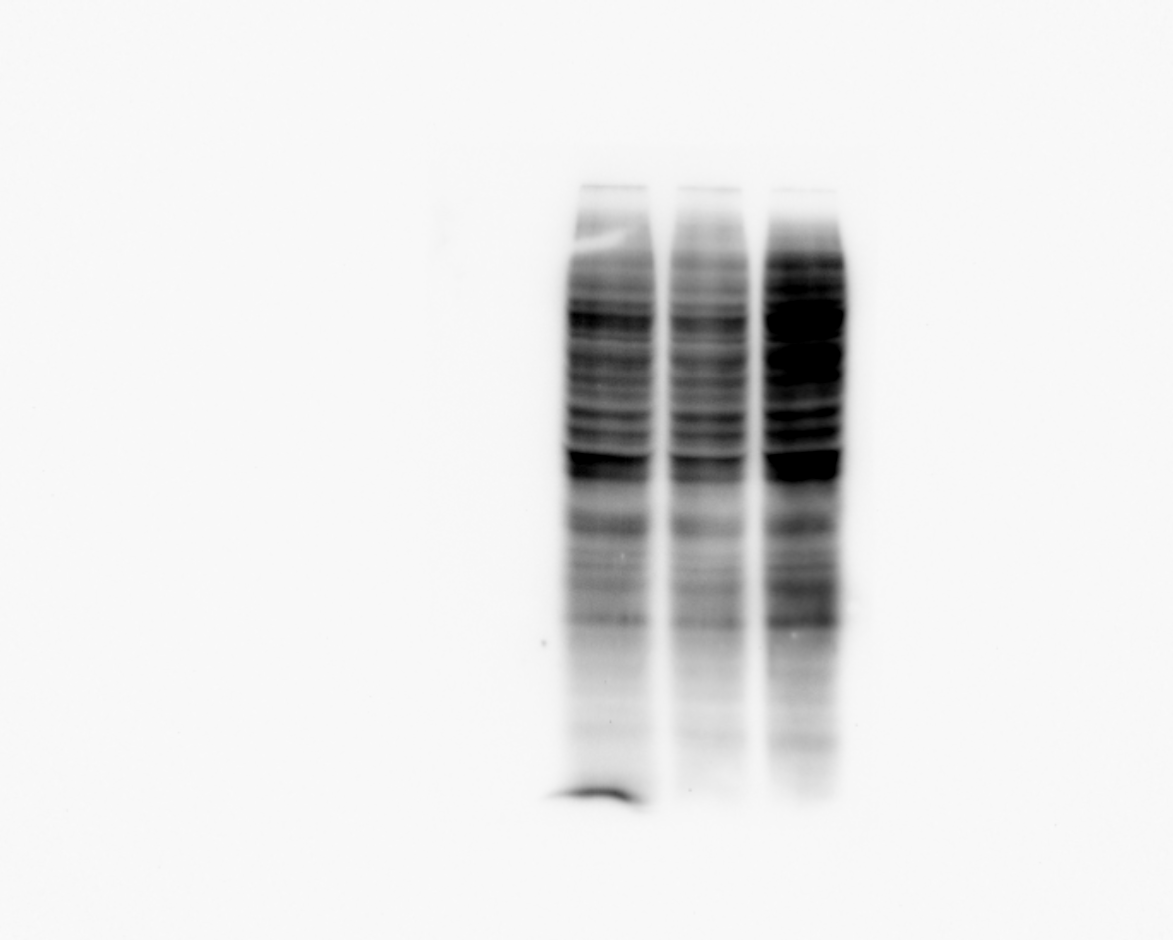

Supplement: Supplementary file 2 — Source data Fig. 1 [file 44319_2026_724_MOESM2_ESM.zip › Figure 1/1J/fig. 1J Puromycin.tif]

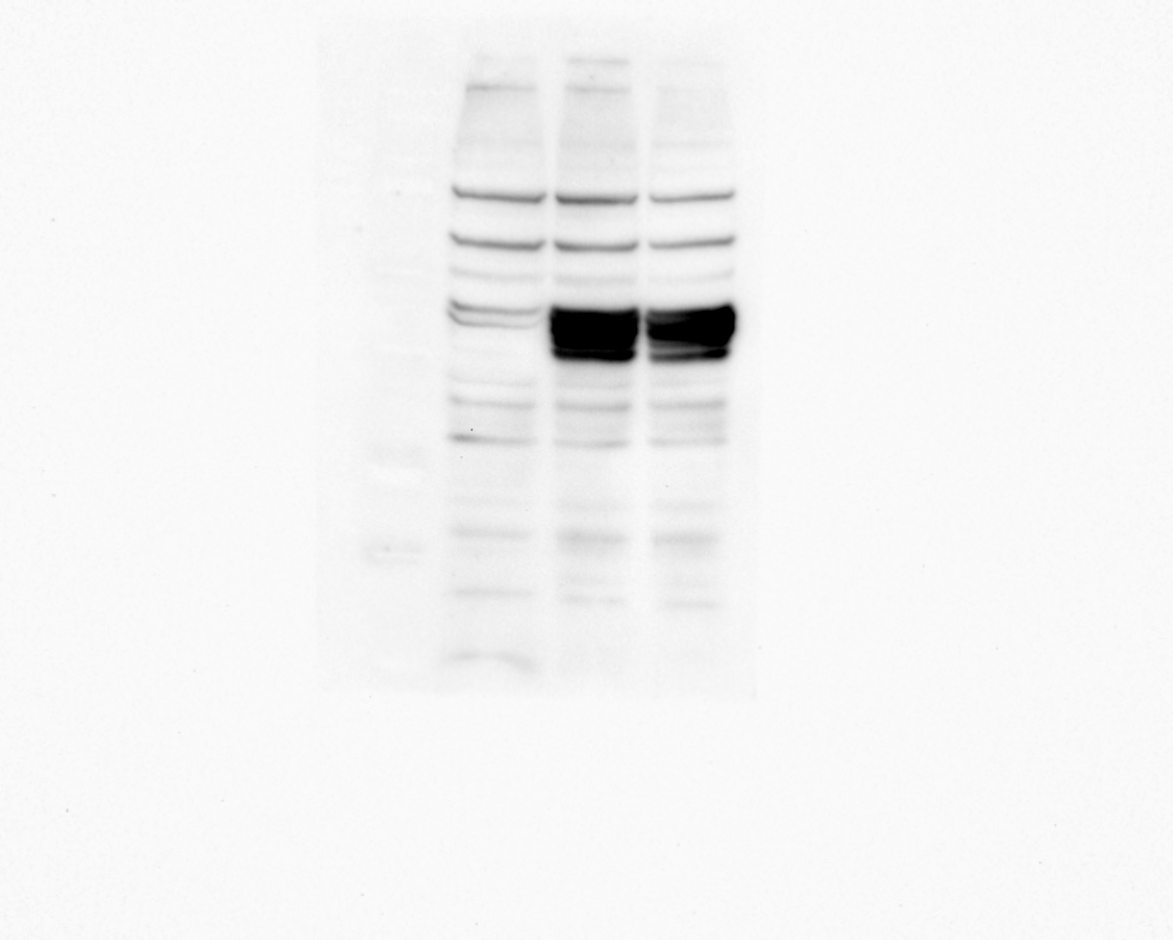

Supplement: Supplementary file 2 — Source data Fig. 1 [file 44319_2026_724_MOESM2_ESM.zip › Figure 1/1J/fig. 1J SIRT2.tif]

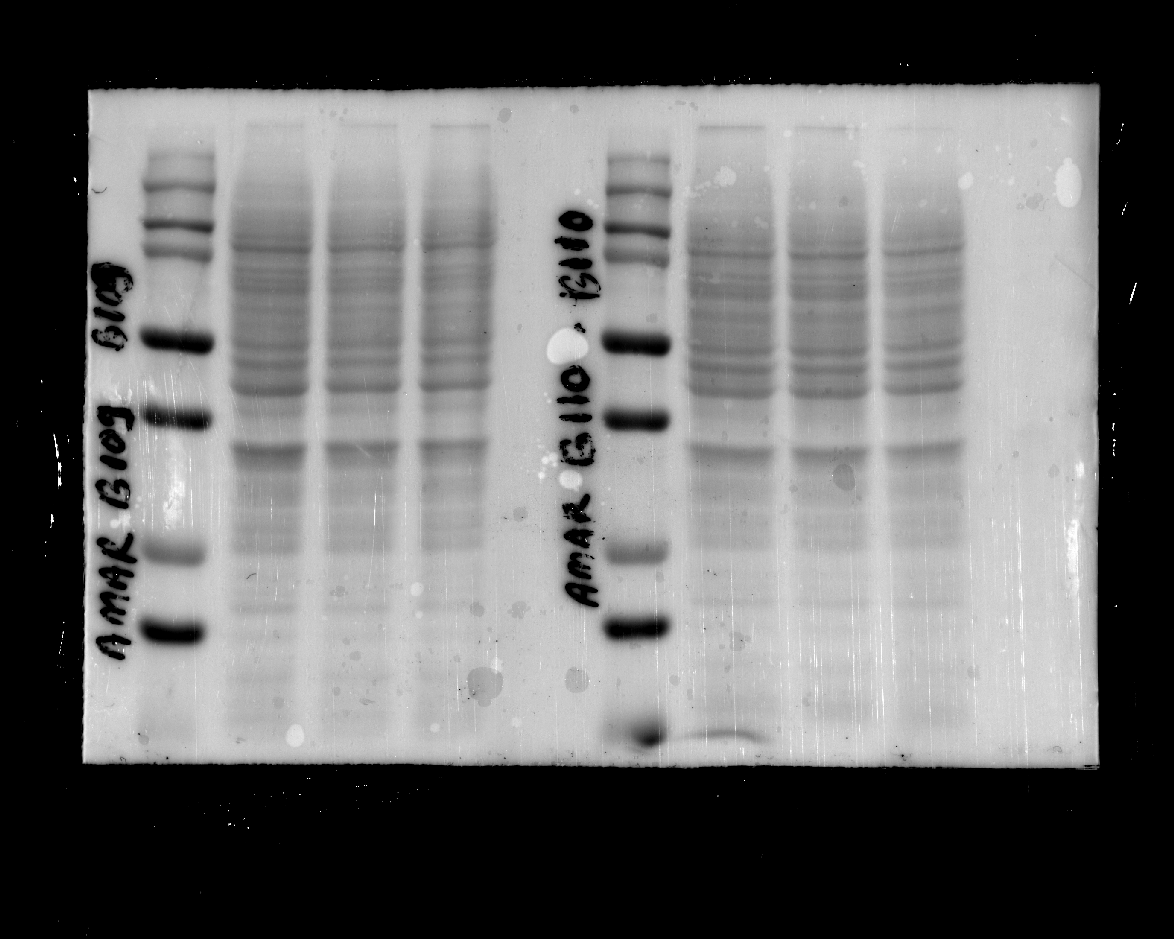

Supplement: Supplementary file 2 — Source data Fig. 1 [file 44319_2026_724_MOESM2_ESM.zip › Figure 1/1J/FIG. 1j PONCEAU.tif]

## Slide 1
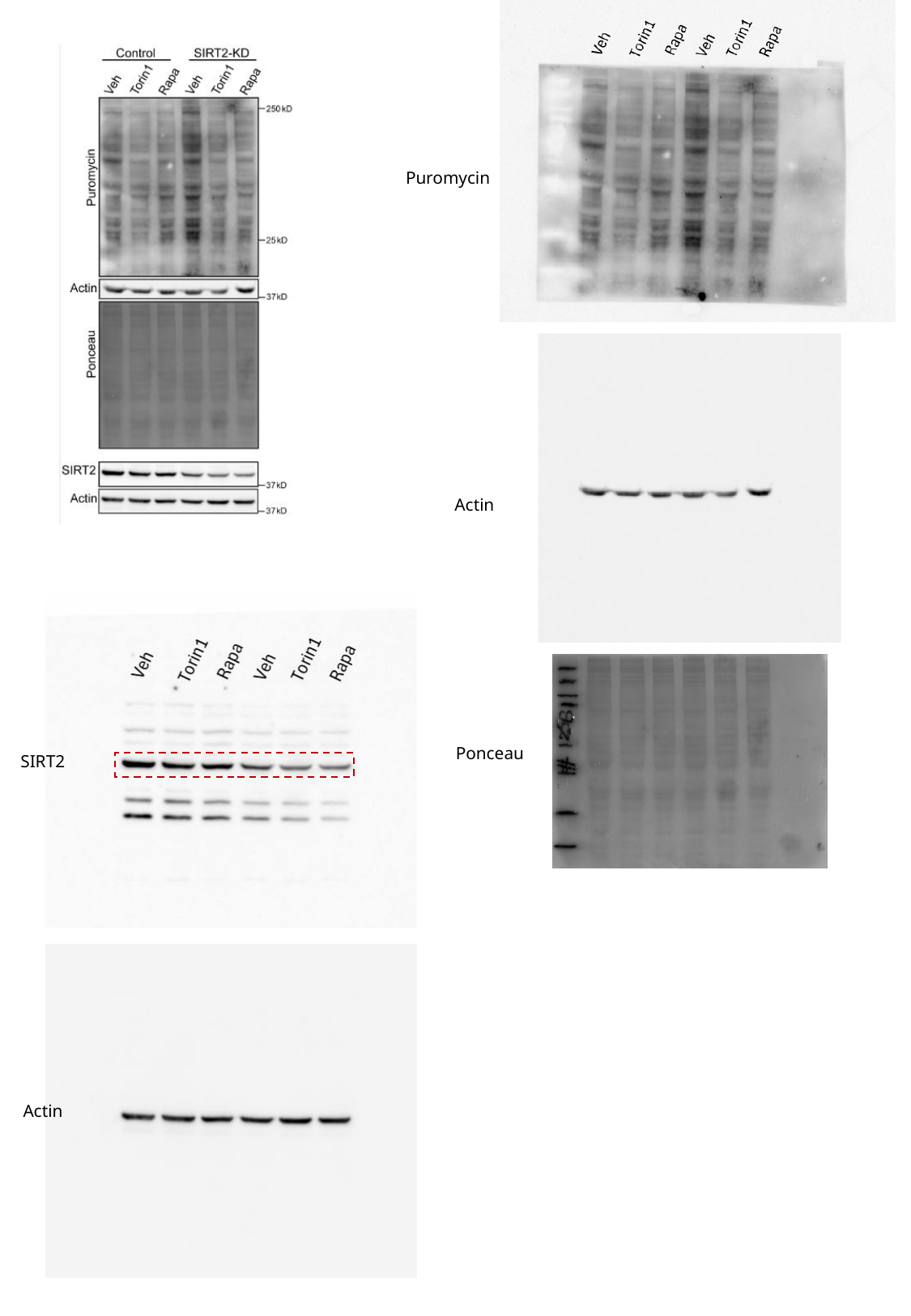

Puromycin
Actin
Ponceau
SIRT2
Actin

Supplement: Supplementary file 3 — Source data Fig. 2 [file 44319_2026_724_MOESM3_ESM.zip › Figure 2/figure 2.pptx]

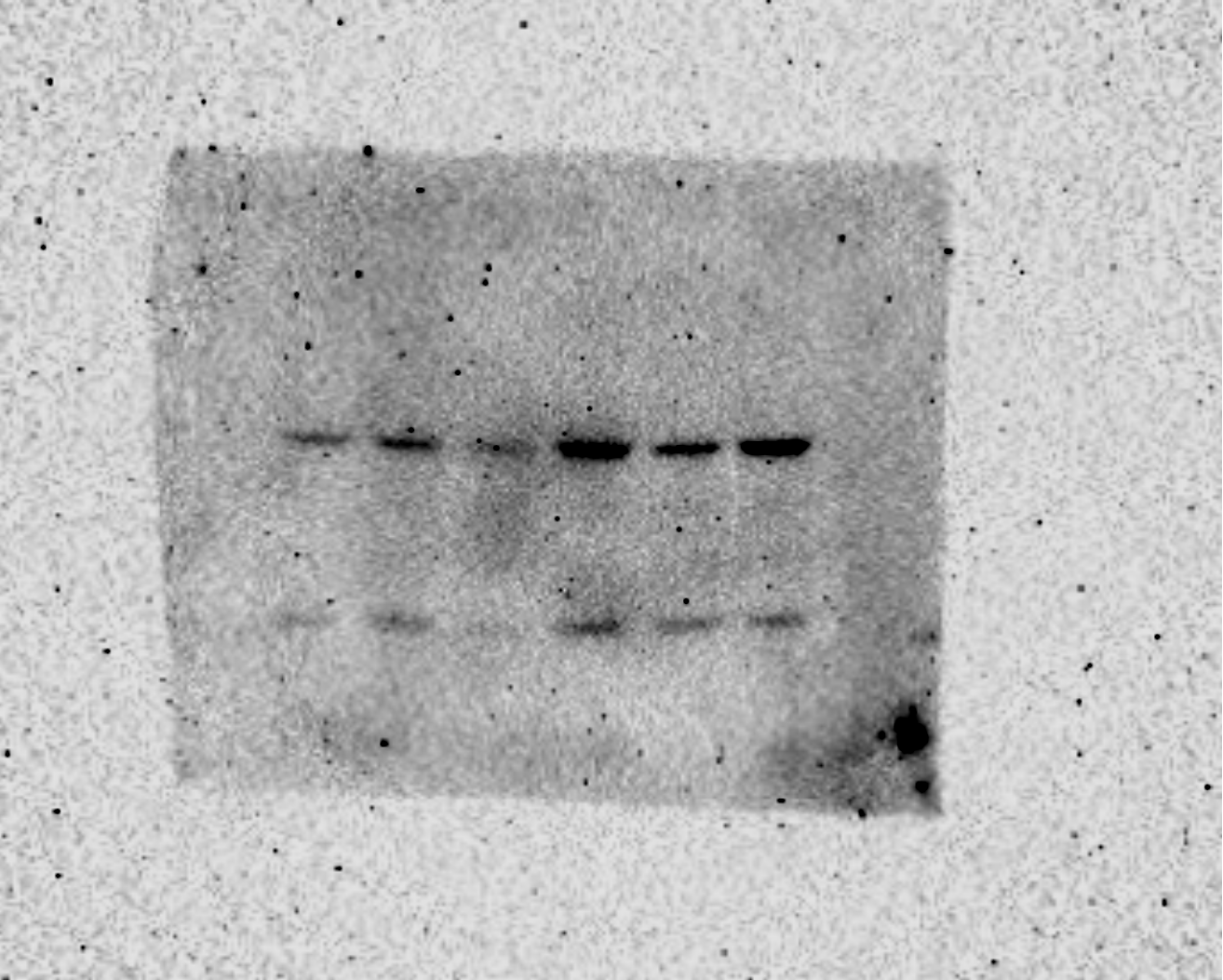

Supplement: Supplementary file 3 — Source data Fig. 2 [file 44319_2026_724_MOESM3_ESM.zip › Figure 2/2E/fig. 2E Ac-Tubulin.tif]

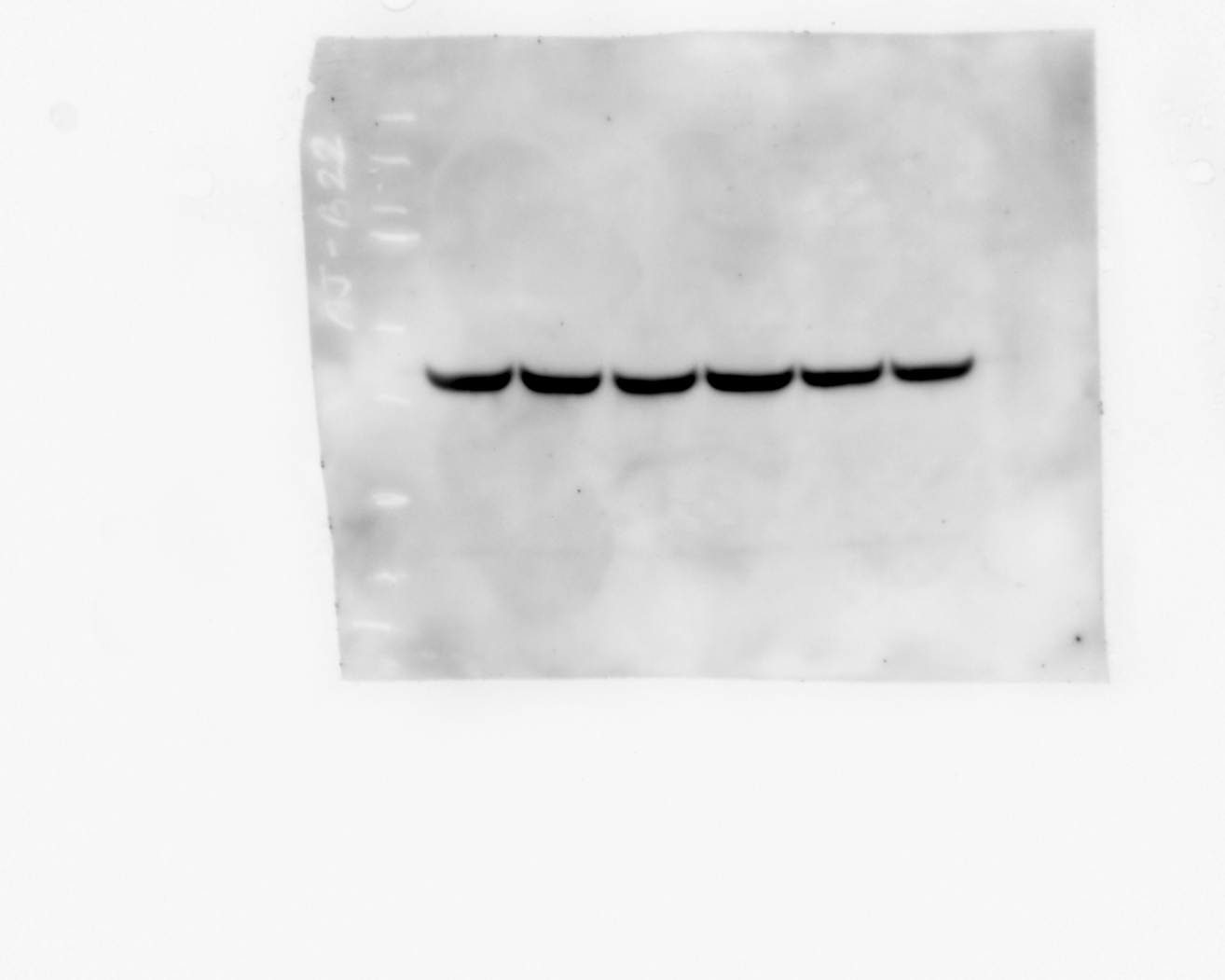

Supplement: Supplementary file 3 — Source data Fig. 2 [file 44319_2026_724_MOESM3_ESM.zip › Figure 2/2E/fig. 2E Actin.tif]

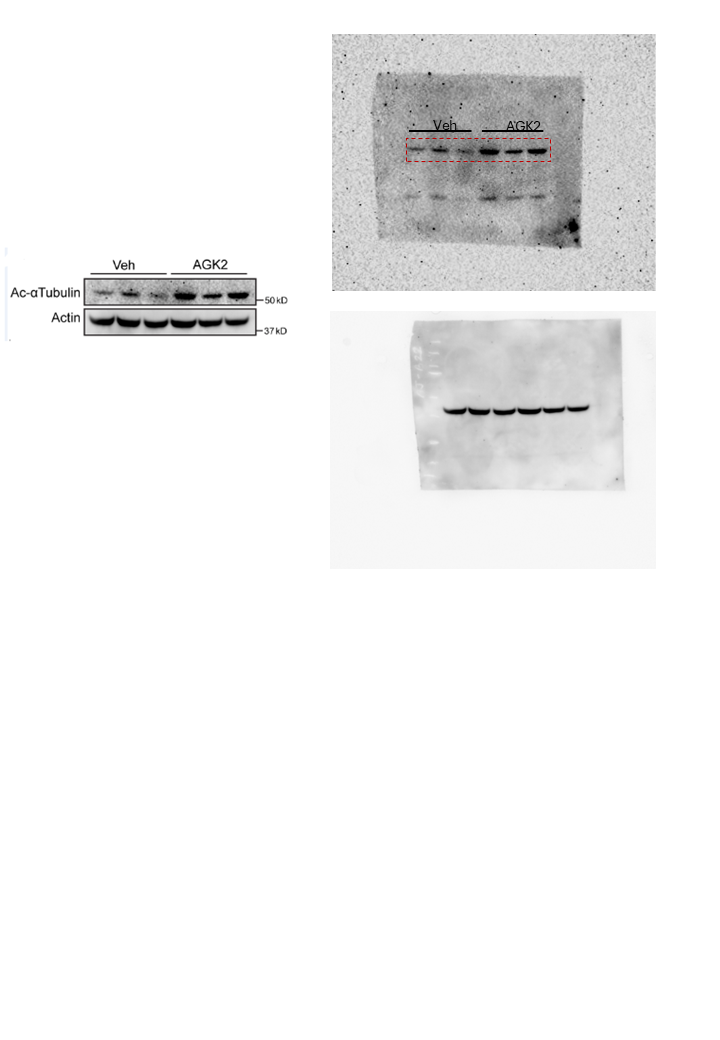

Supplement: Supplementary file 3 — Source data Fig. 2 [file 44319_2026_724_MOESM3_ESM.zip › Figure 2/2E/2E.tif]

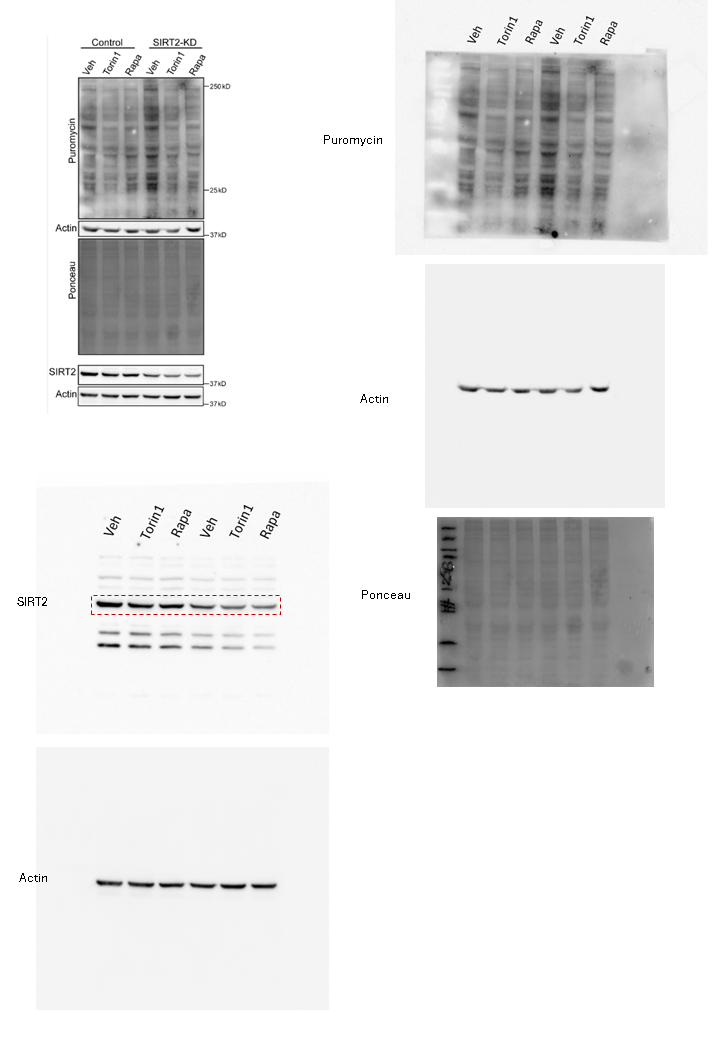

Supplement: Supplementary file 3 — Source data Fig. 2 [file 44319_2026_724_MOESM3_ESM.zip › Figure 2/2F/2F.tif]

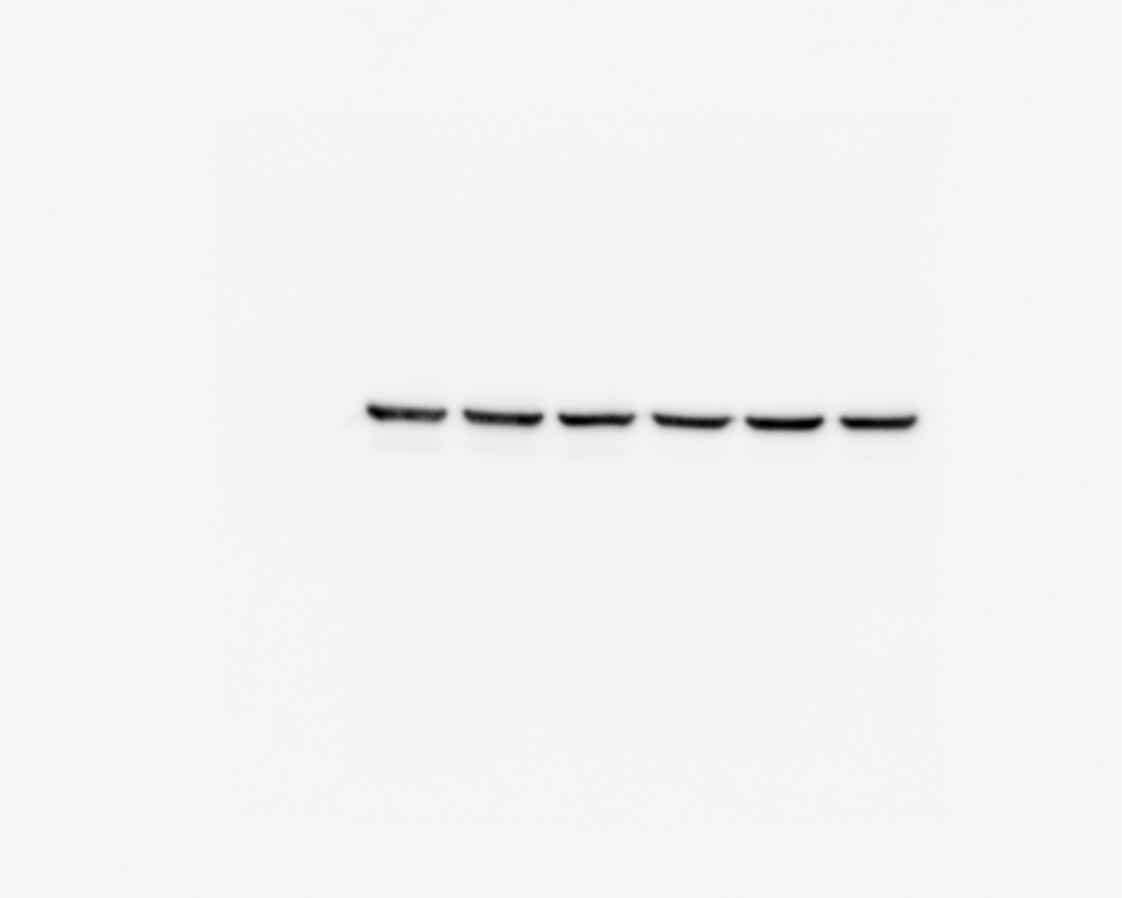

Supplement: Supplementary file 3 — Source data Fig. 2 [file 44319_2026_724_MOESM3_ESM.zip › Figure 2/2F/Fig. 2F Actin for SIRT2.tif]

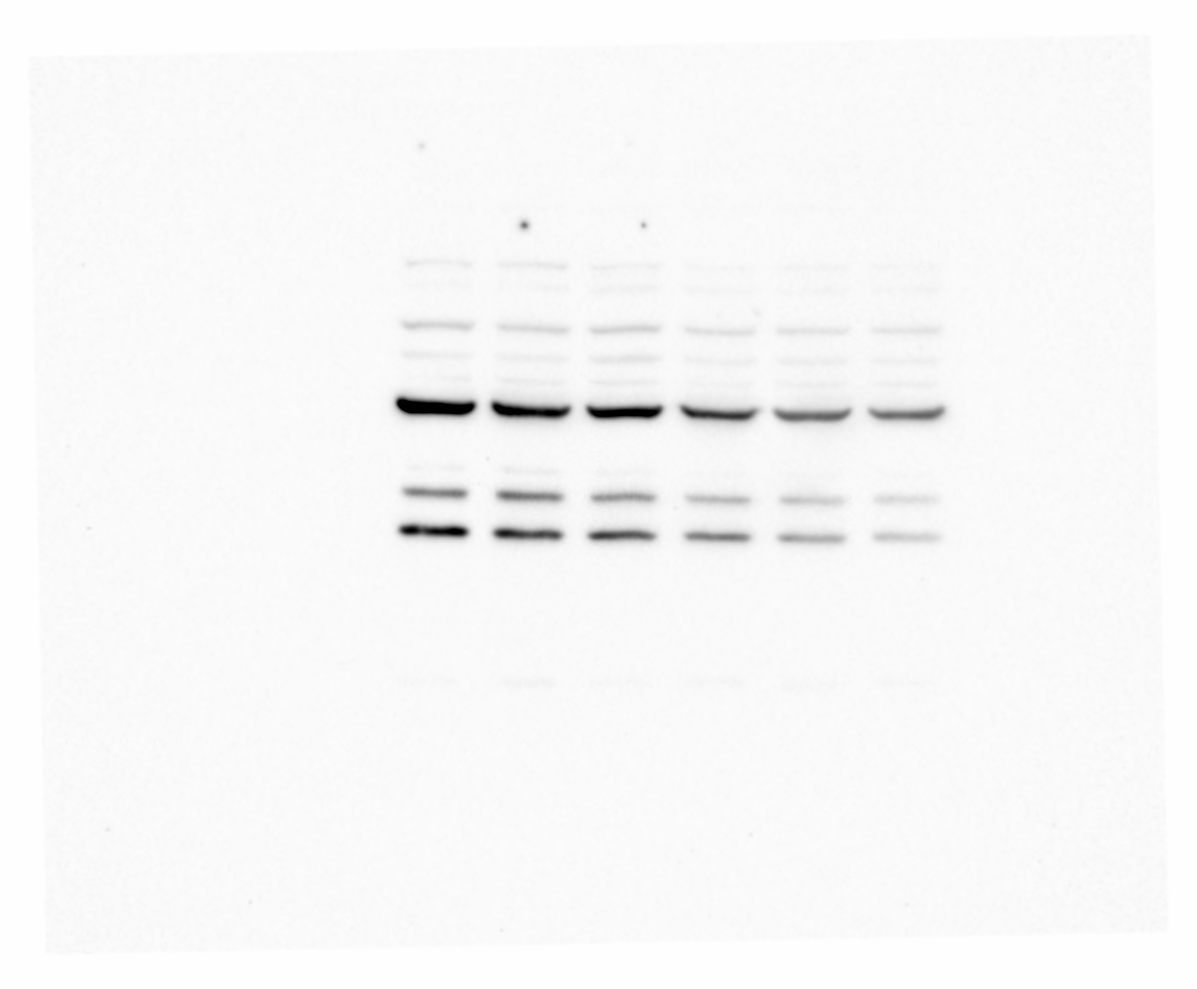

Supplement: Supplementary file 3 — Source data Fig. 2 [file 44319_2026_724_MOESM3_ESM.zip › Figure 2/2F/fig. 2F SIRT2.tif]

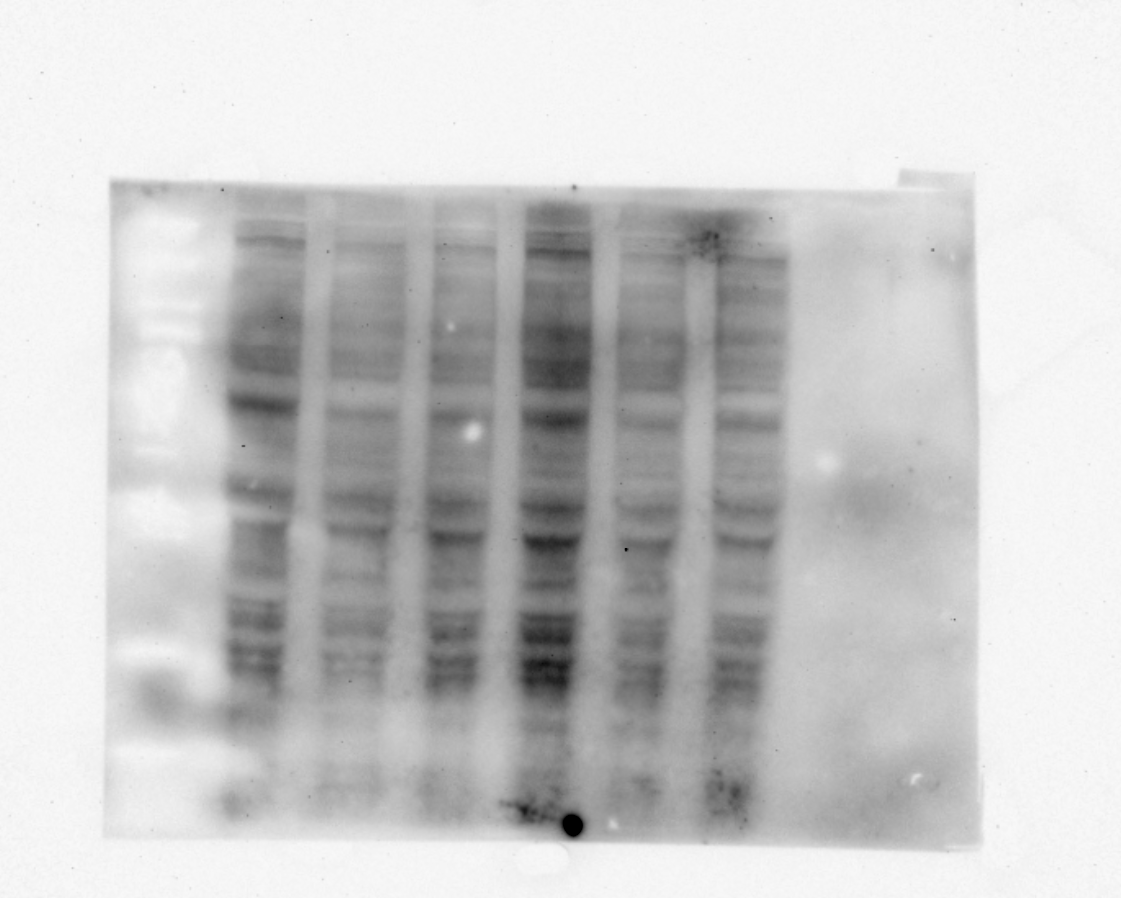

Supplement: Supplementary file 3 — Source data Fig. 2 [file 44319_2026_724_MOESM3_ESM.zip › Figure 2/2F/fig. 2F Puromycin.tif]

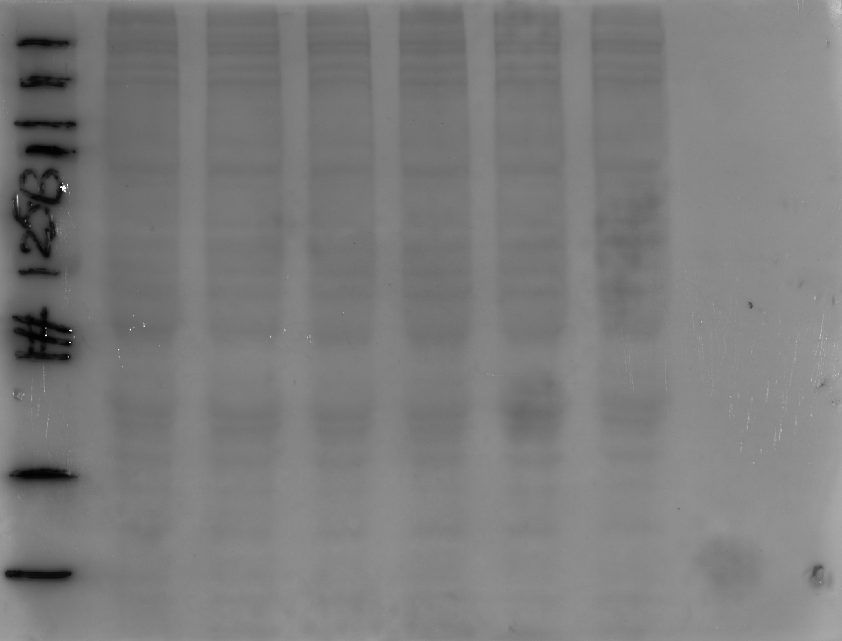

Supplement: Supplementary file 3 — Source data Fig. 2 [file 44319_2026_724_MOESM3_ESM.zip › Figure 2/2F/fig. 2F Ponceau.tif]

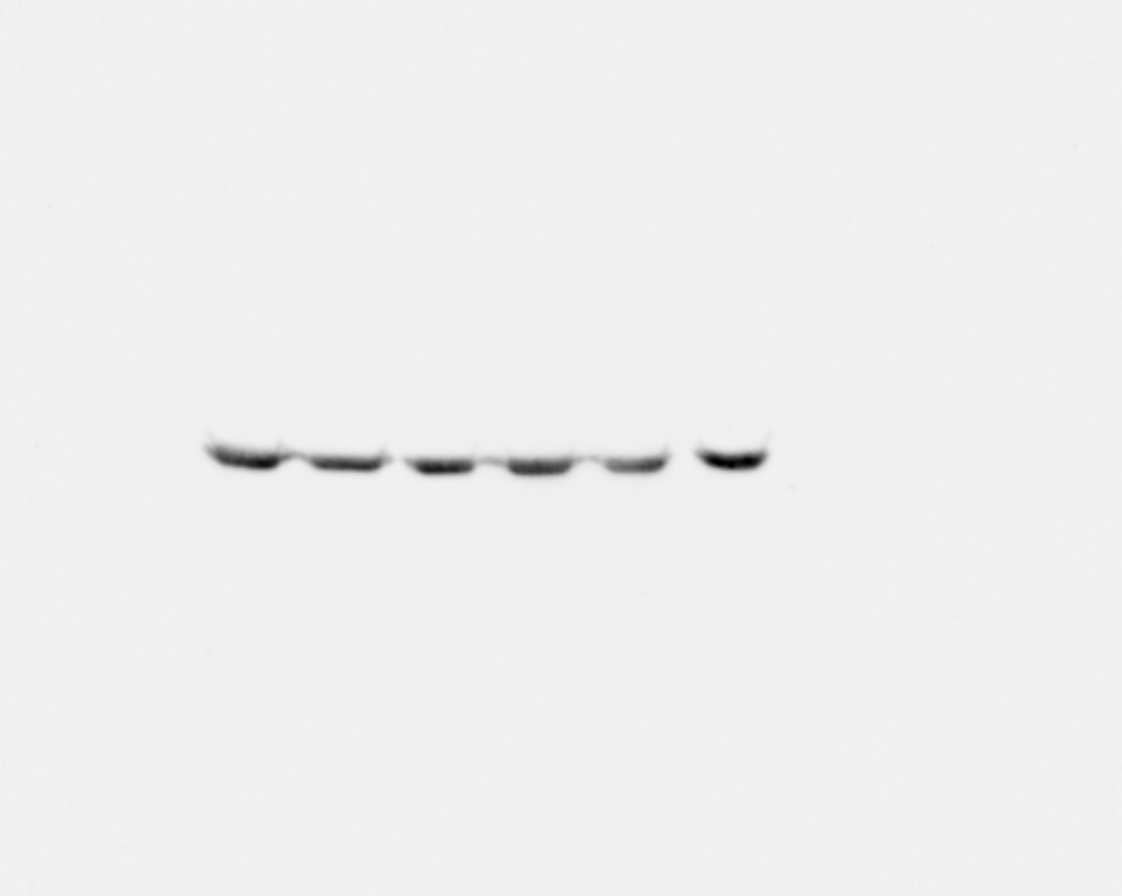

Supplement: Supplementary file 3 — Source data Fig. 2 [file 44319_2026_724_MOESM3_ESM.zip › Figure 2/2F/fig. 2F actin.tif]

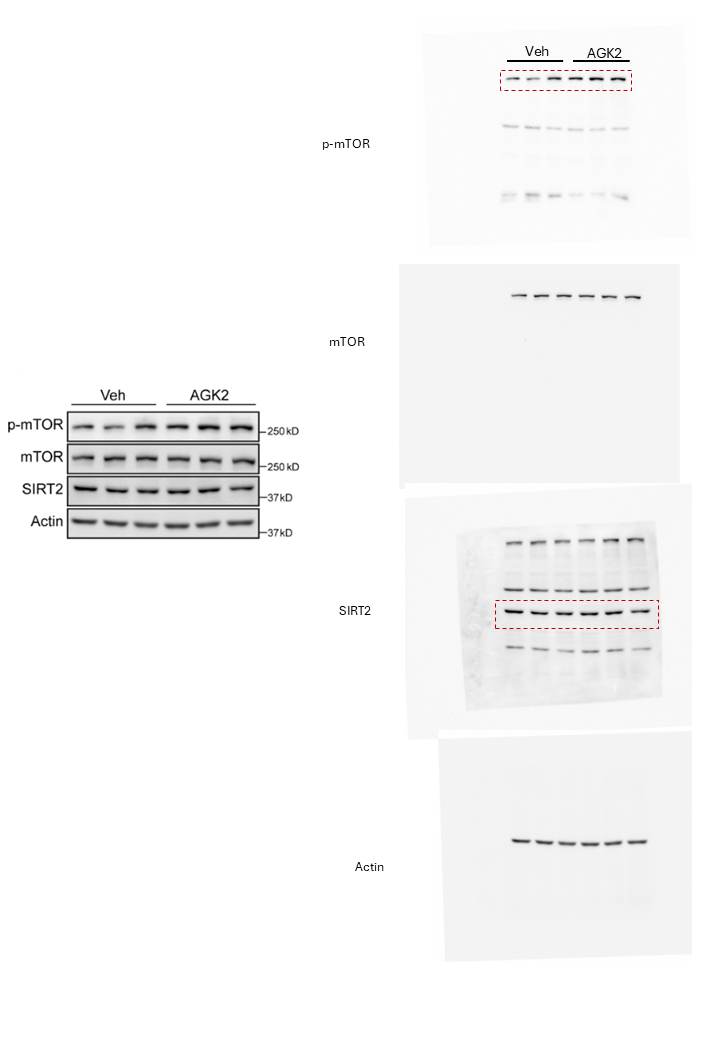

Supplement: Supplementary file 3 — Source data Fig. 2 [file 44319_2026_724_MOESM3_ESM.zip › Figure 2/2G/2G.tif]

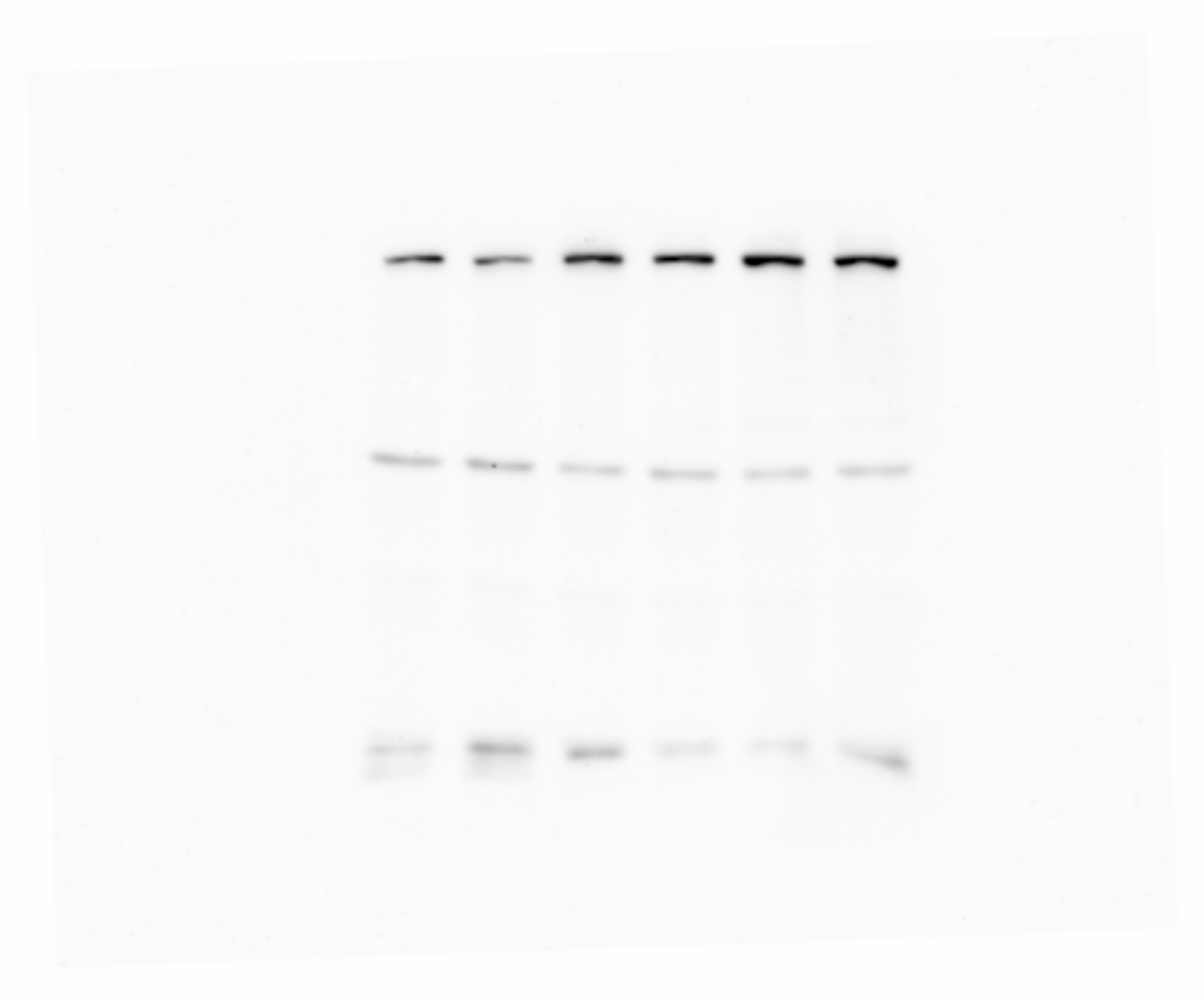

Supplement: Supplementary file 3 — Source data Fig. 2 [file 44319_2026_724_MOESM3_ESM.zip › Figure 2/2G/fig. 2G p-mTOR.tif]

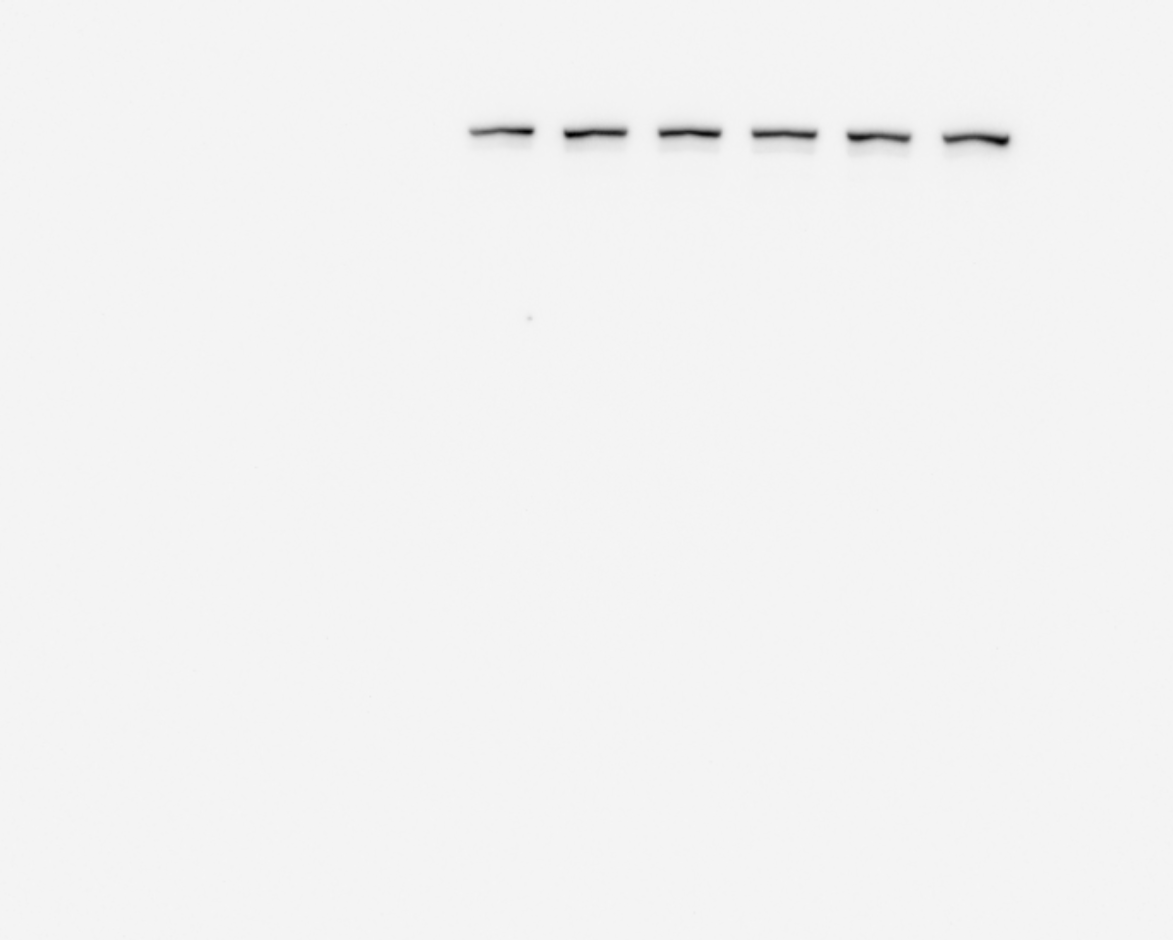

Supplement: Supplementary file 3 — Source data Fig. 2 [file 44319_2026_724_MOESM3_ESM.zip › Figure 2/2G/fig. 2G mTOR.tif]

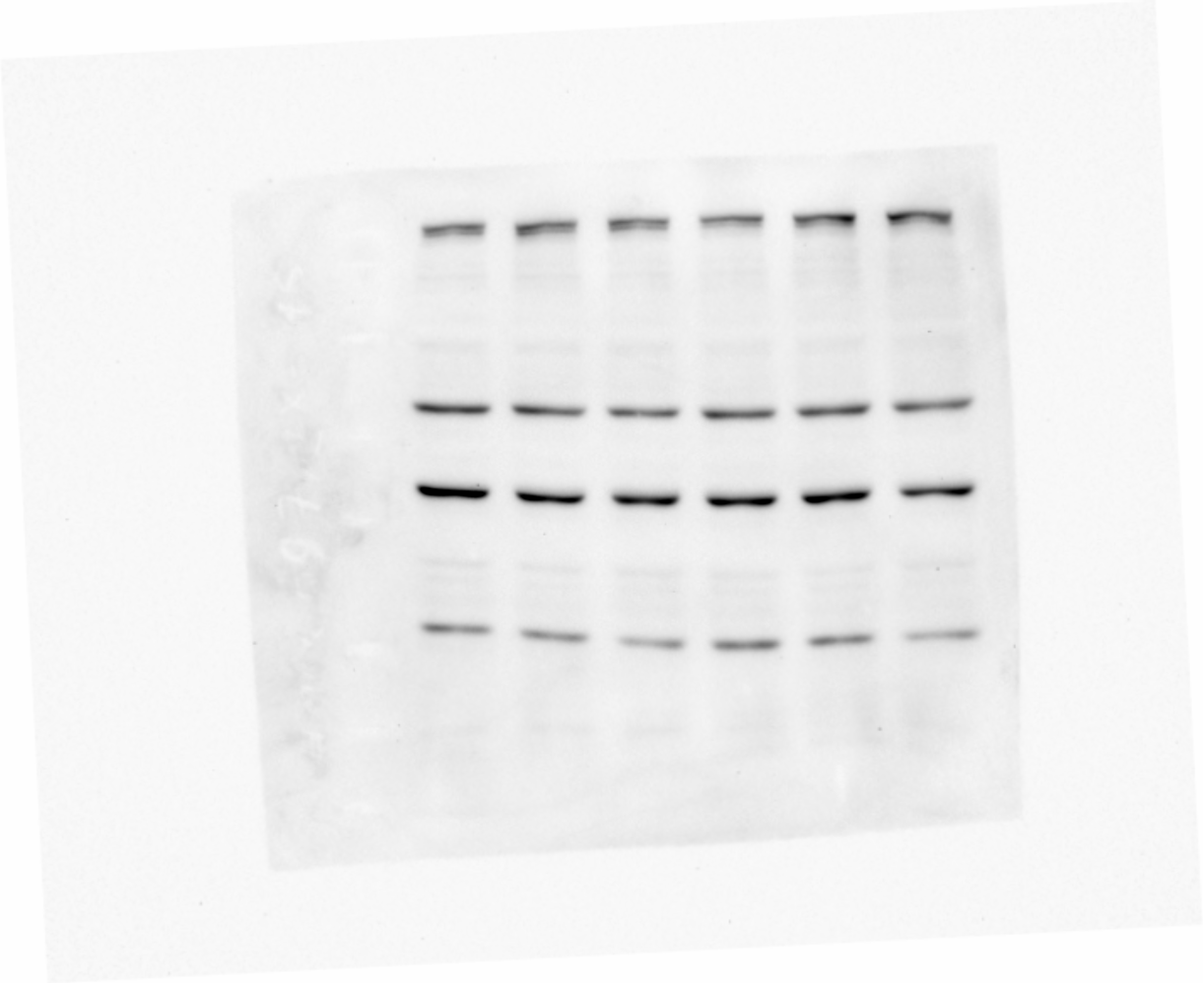

Supplement: Supplementary file 3 — Source data Fig. 2 [file 44319_2026_724_MOESM3_ESM.zip › Figure 2/2G/fig. 2G SIRT2.tif]

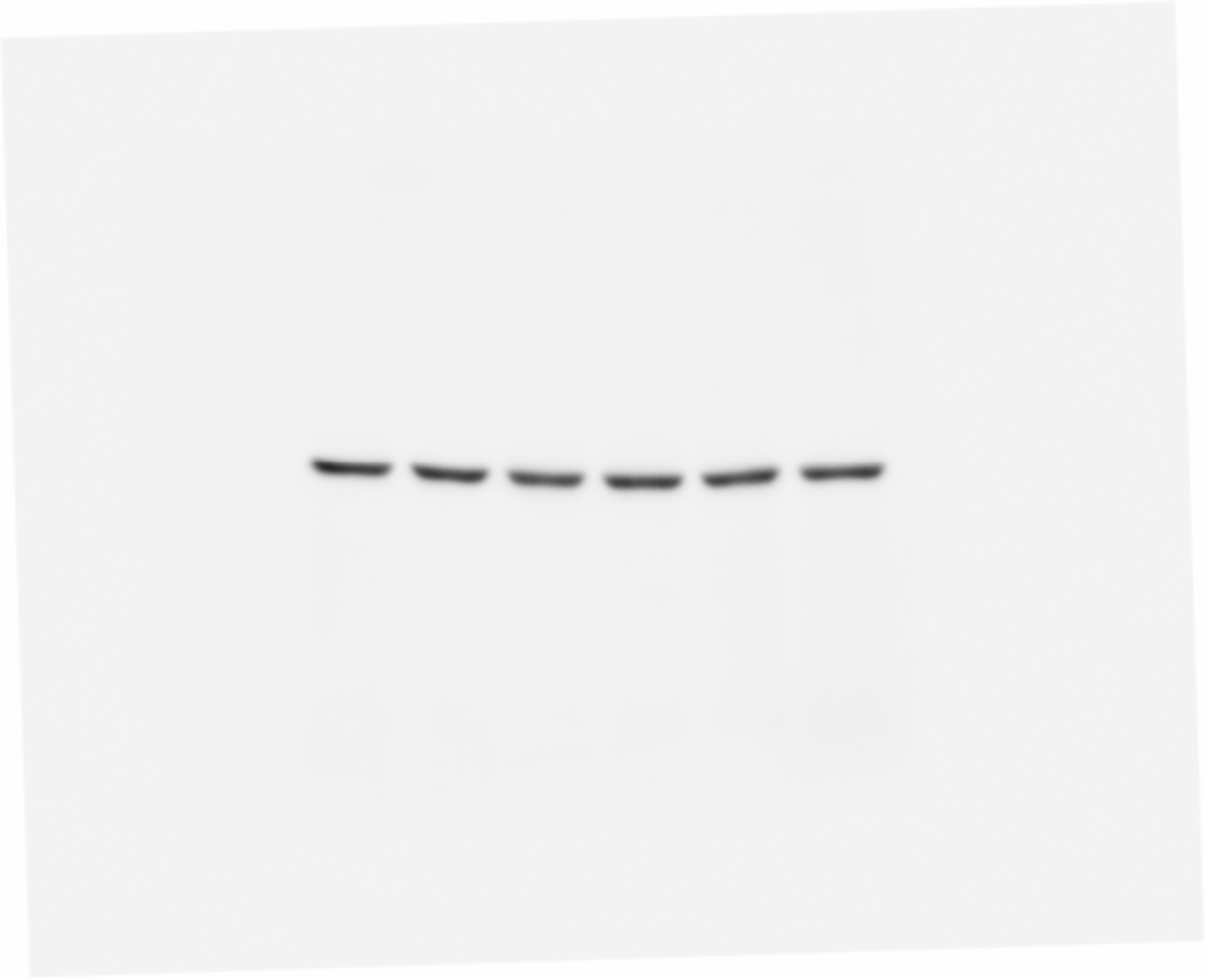

Supplement: Supplementary file 3 — Source data Fig. 2 [file 44319_2026_724_MOESM3_ESM.zip › Figure 2/2G/fig. 2G Actin.tif]

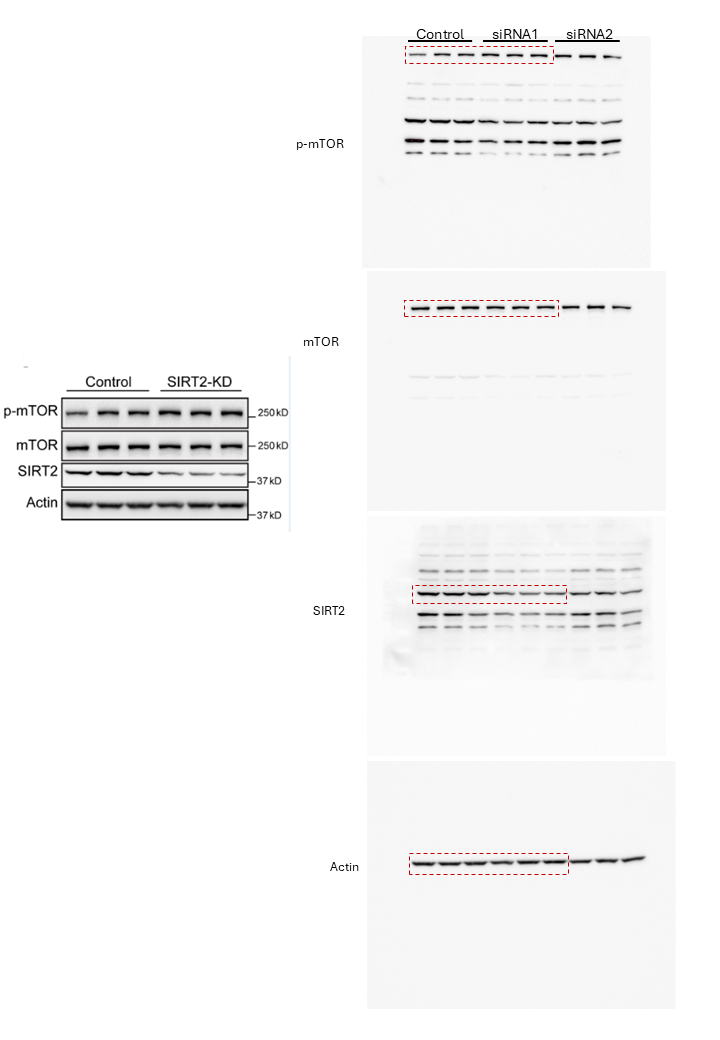

Supplement: Supplementary file 3 — Source data Fig. 2 [file 44319_2026_724_MOESM3_ESM.zip › Figure 2/2I/2I.tif]

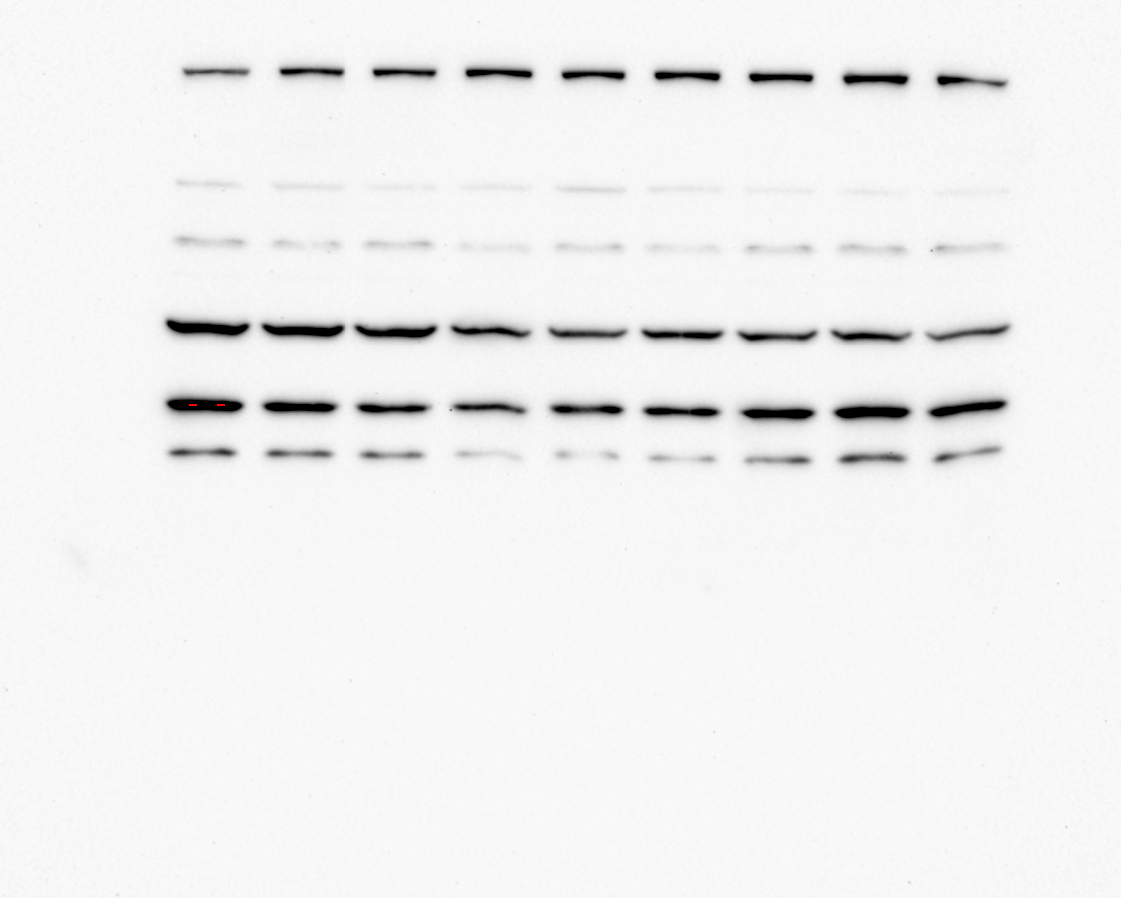

Supplement: Supplementary file 3 — Source data Fig. 2 [file 44319_2026_724_MOESM3_ESM.zip › Figure 2/2I/fig. 2I p-mtor.tif]

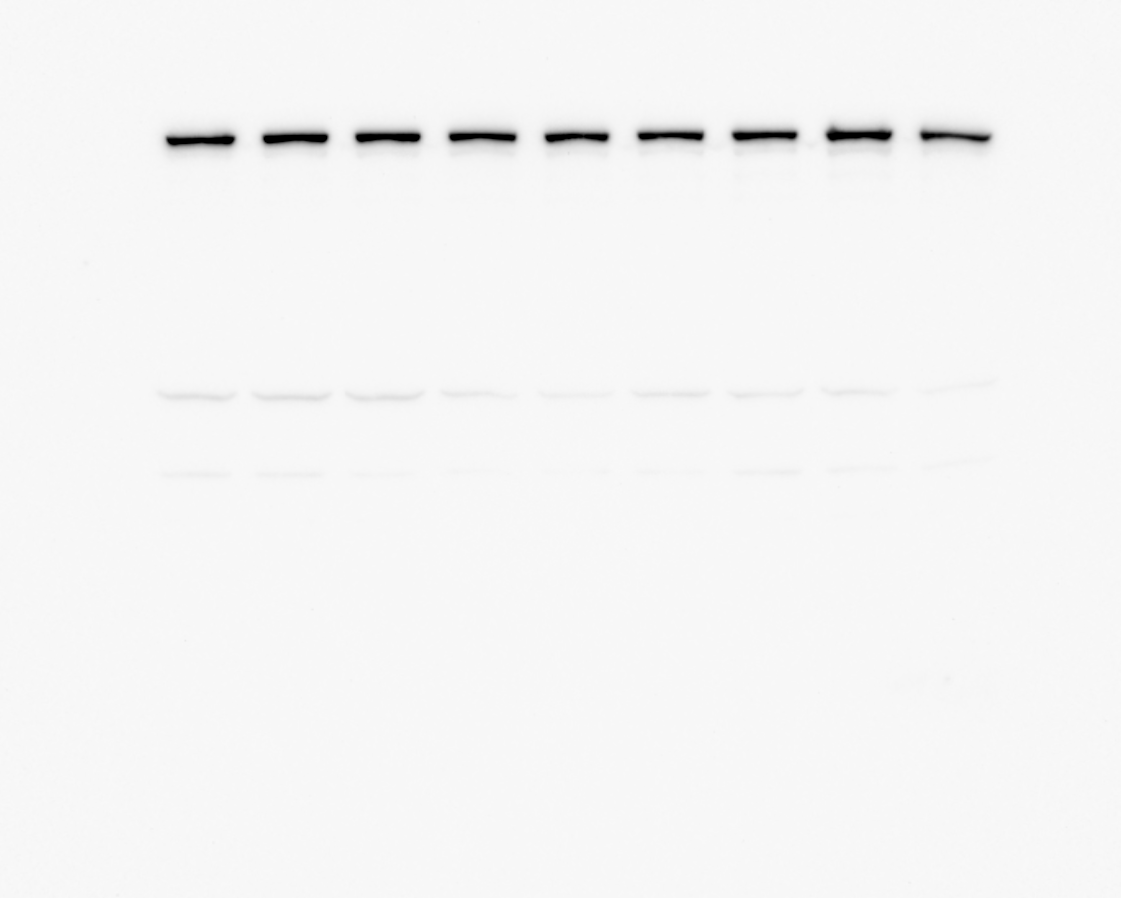

Supplement: Supplementary file 3 — Source data Fig. 2 [file 44319_2026_724_MOESM3_ESM.zip › Figure 2/2I/fig. 2I mtor.tif]

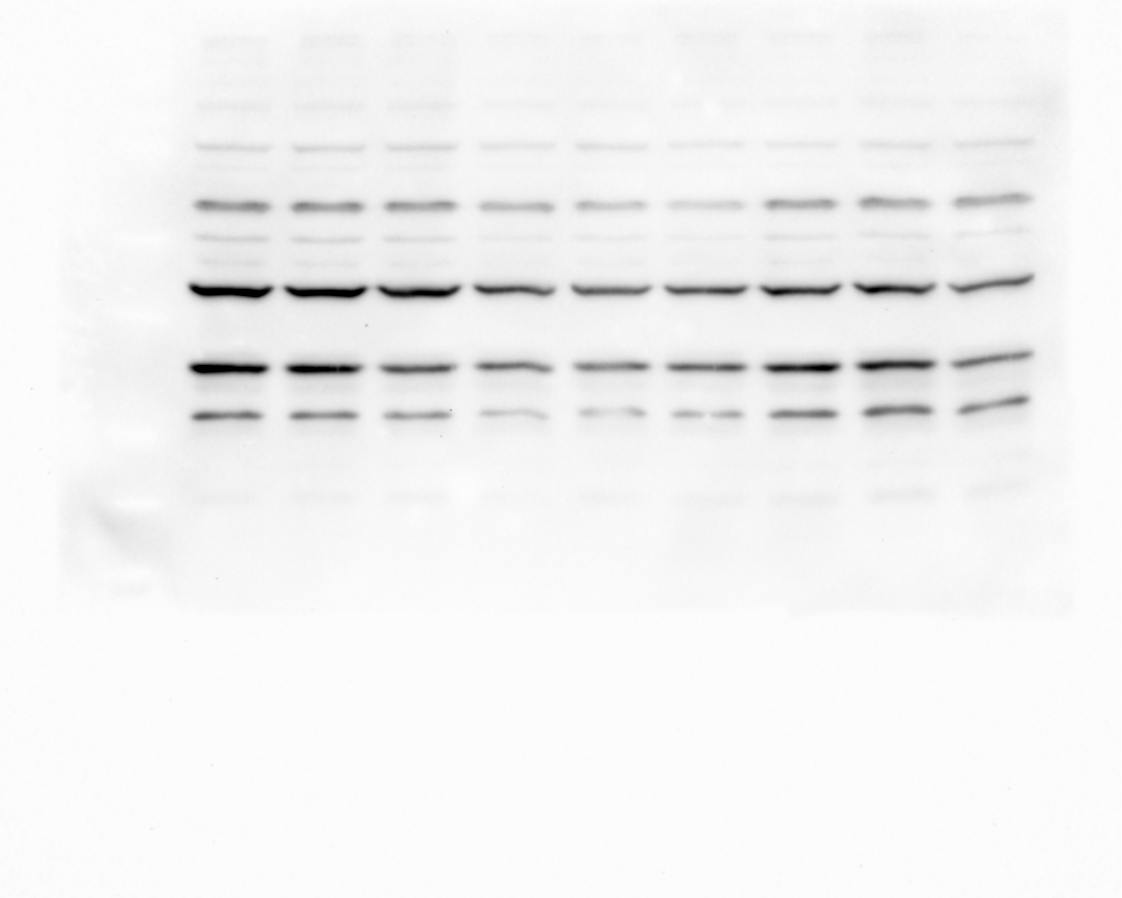

Supplement: Supplementary file 3 — Source data Fig. 2 [file 44319_2026_724_MOESM3_ESM.zip › Figure 2/2I/fig. 2I SIRT2.tif]

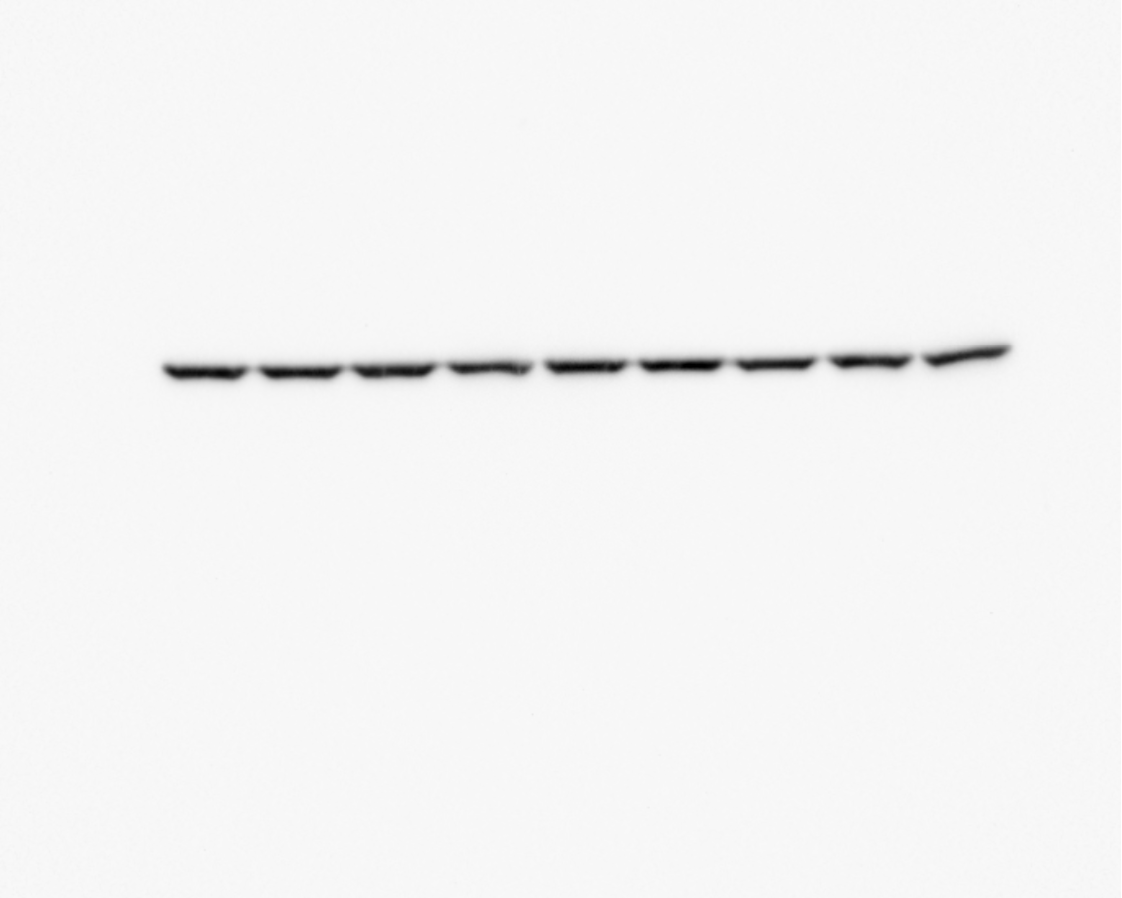

Supplement: Supplementary file 3 — Source data Fig. 2 [file 44319_2026_724_MOESM3_ESM.zip › Figure 2/2I/fig. 2I Actin.tif]

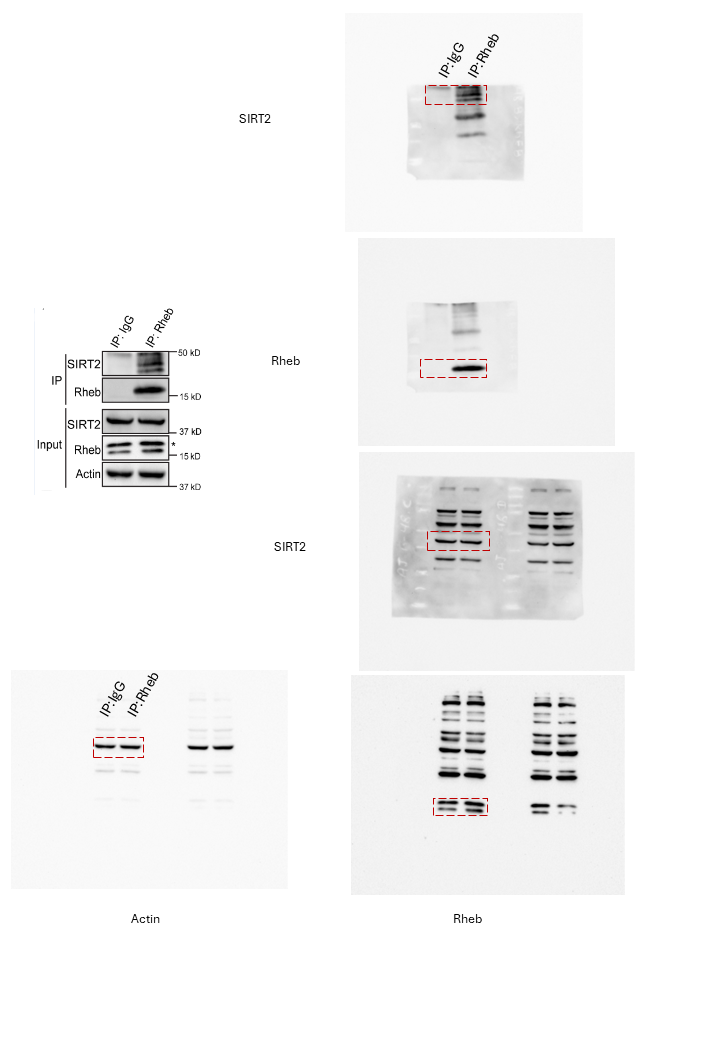

Supplement: Supplementary file 4 — Source data Fig. 3 [file 44319_2026_724_MOESM4_ESM.zip › Figure 3/3A/3A.tif]

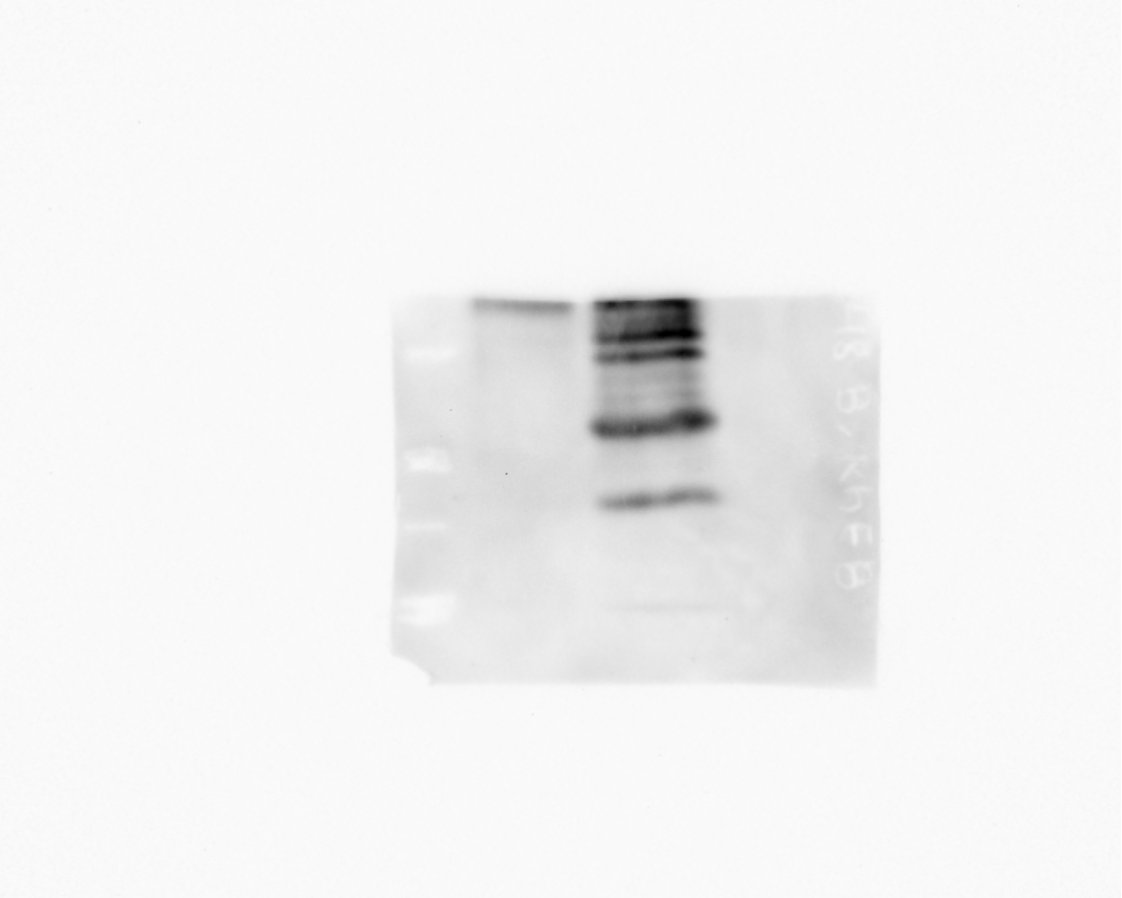

Supplement: Supplementary file 4 — Source data Fig. 3 [file 44319_2026_724_MOESM4_ESM.zip › Figure 3/3A/fig. 3A IP sirt2_20.tif]

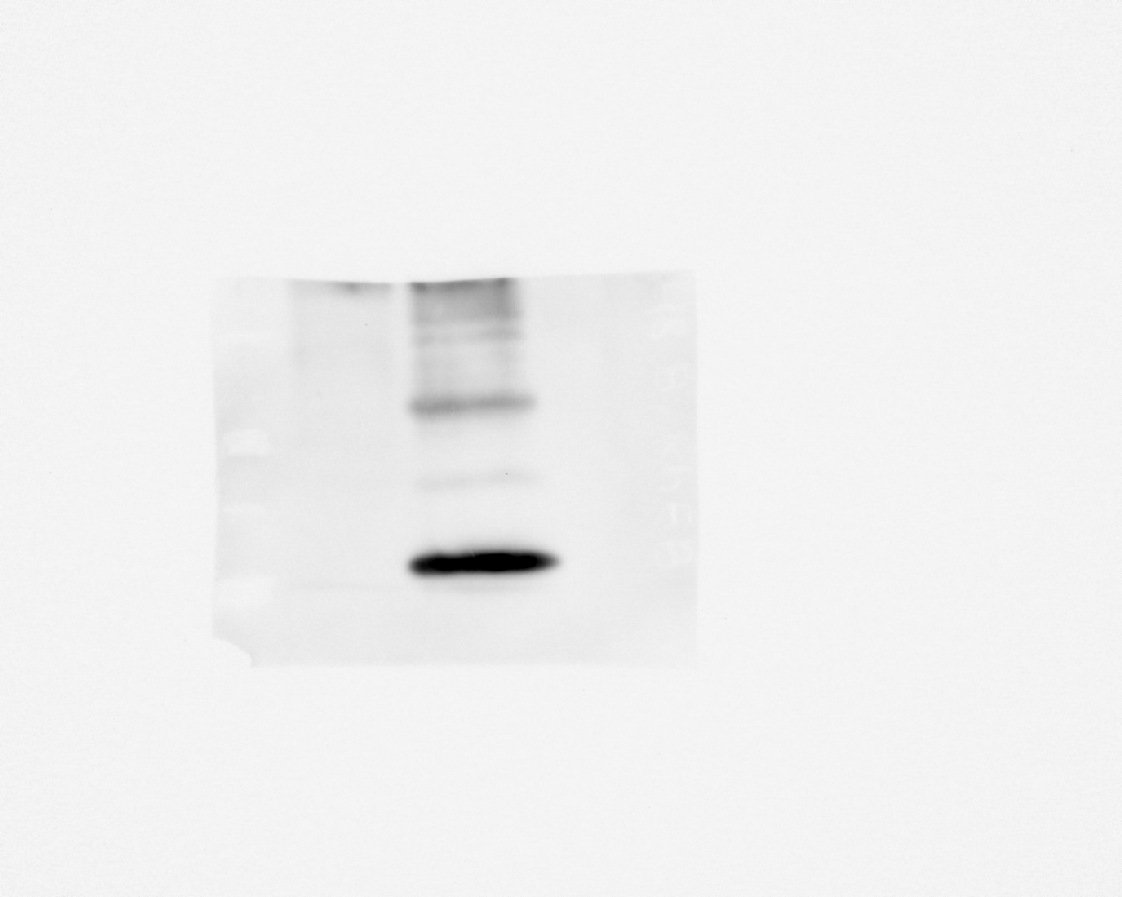

Supplement: Supplementary file 4 — Source data Fig. 3 [file 44319_2026_724_MOESM4_ESM.zip › Figure 3/3A/fig. 3A IP rheb_12.tif]

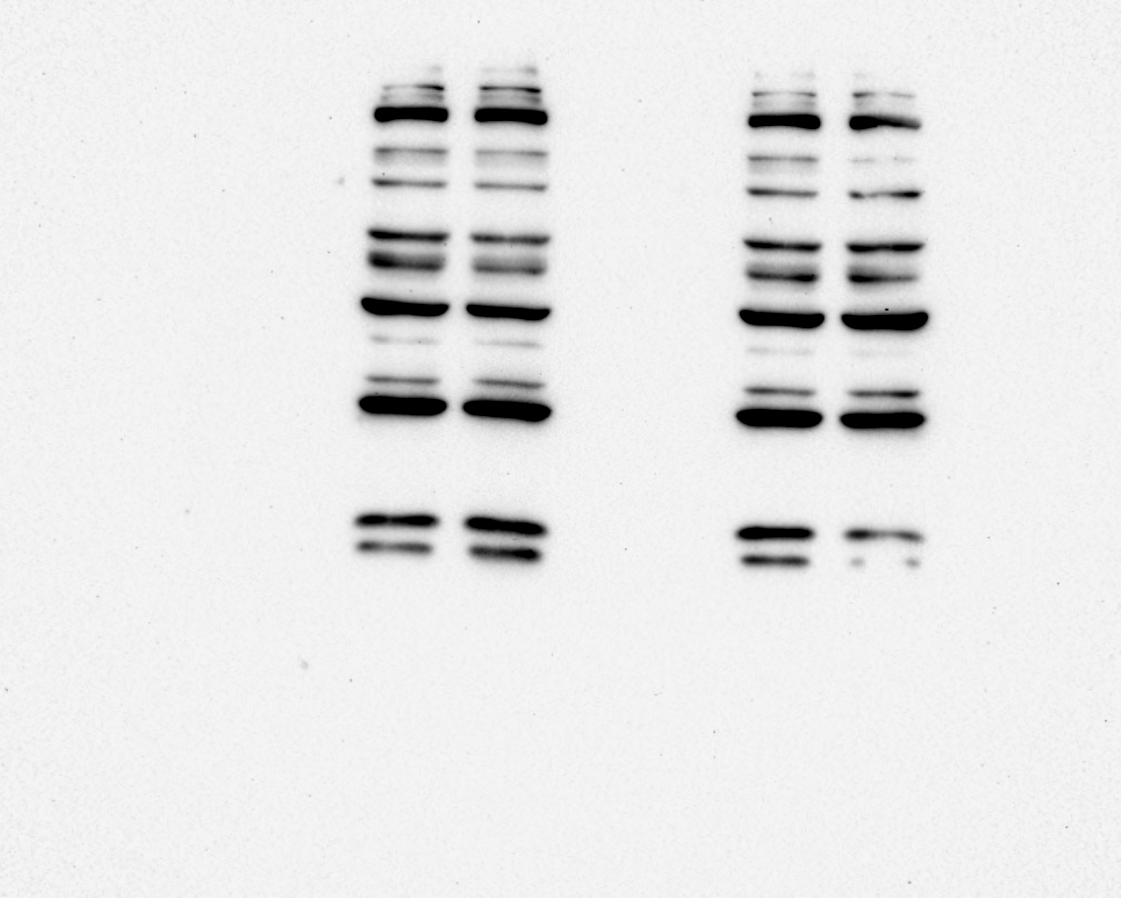

Supplement: Supplementary file 4 — Source data Fig. 3 [file 44319_2026_724_MOESM4_ESM.zip › Figure 3/3A/fig. 3A rheb WCL.tif]

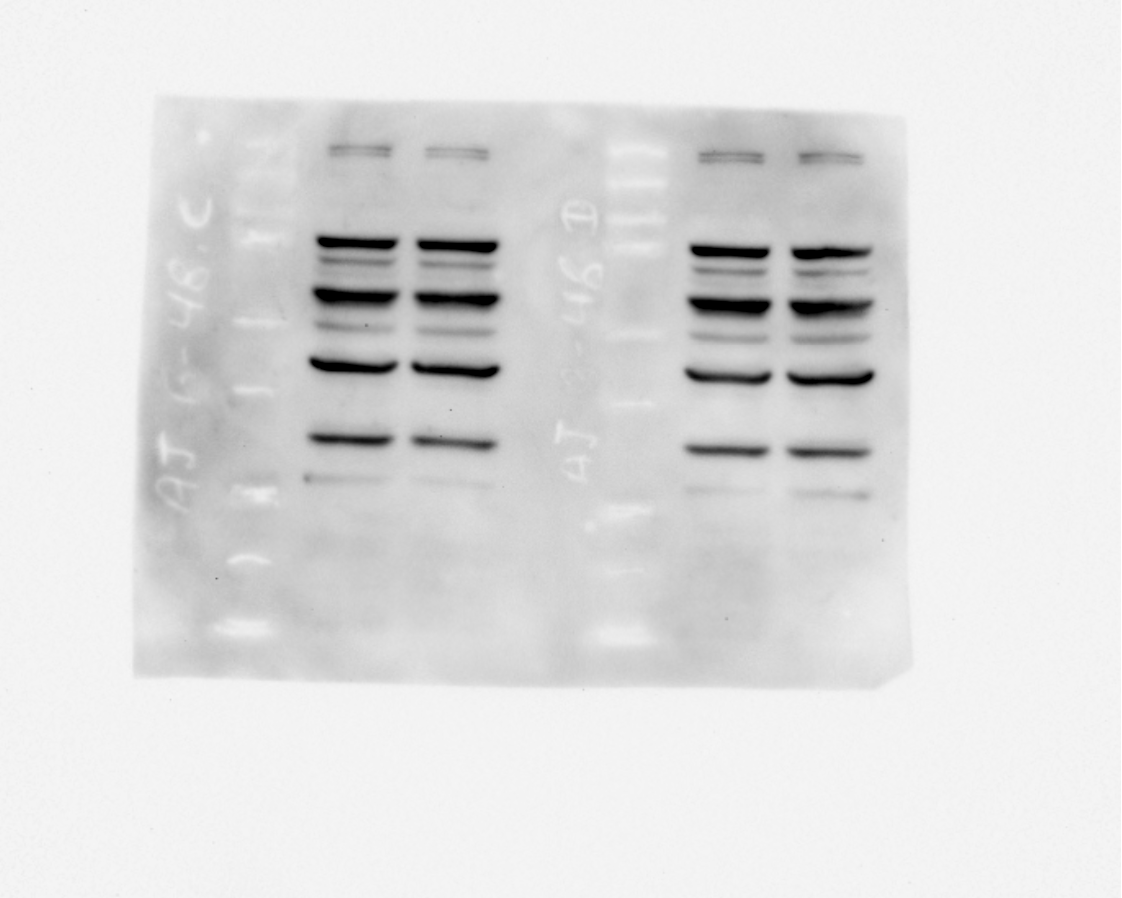

Supplement: Supplementary file 4 — Source data Fig. 3 [file 44319_2026_724_MOESM4_ESM.zip › Figure 3/3A/fig. 3A sirt2 WCL.tif]

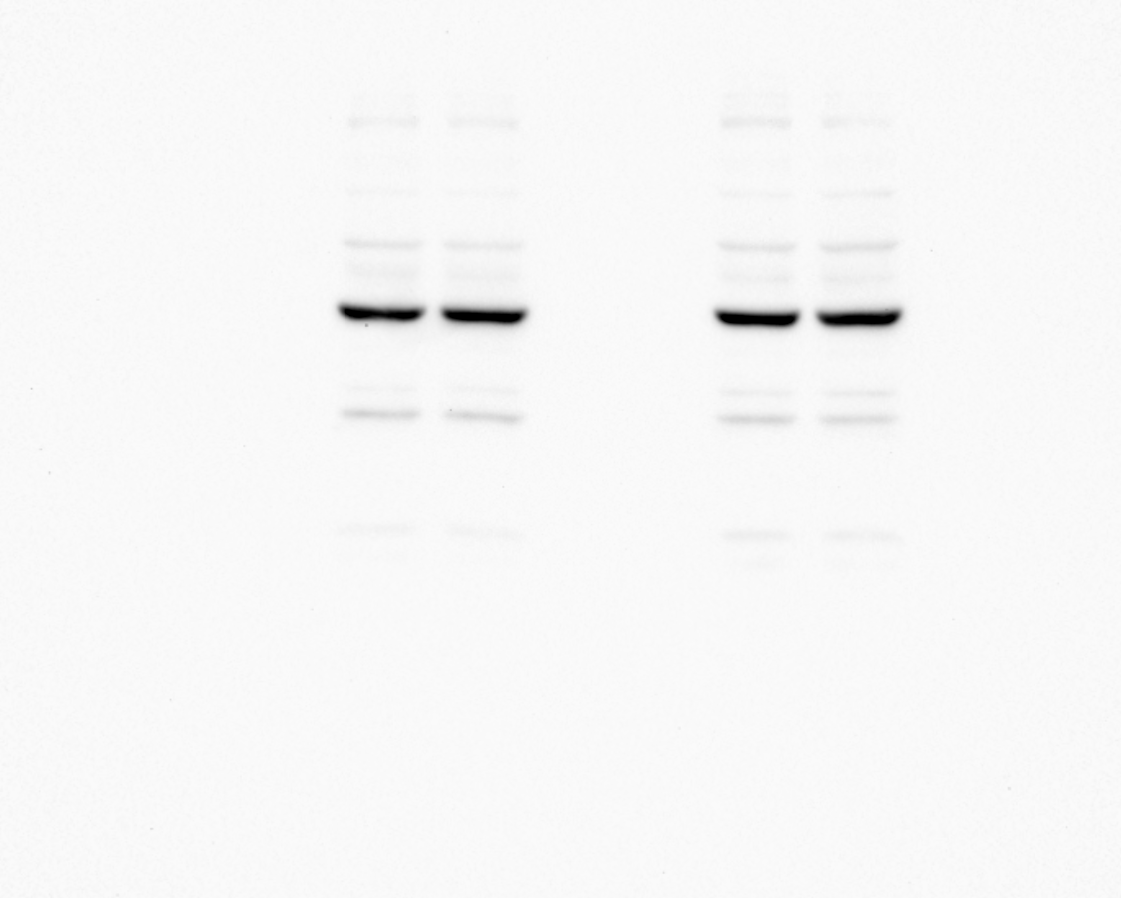

Supplement: Supplementary file 4 — Source data Fig. 3 [file 44319_2026_724_MOESM4_ESM.zip › Figure 3/3A/fig.3A actin WCl.tif]

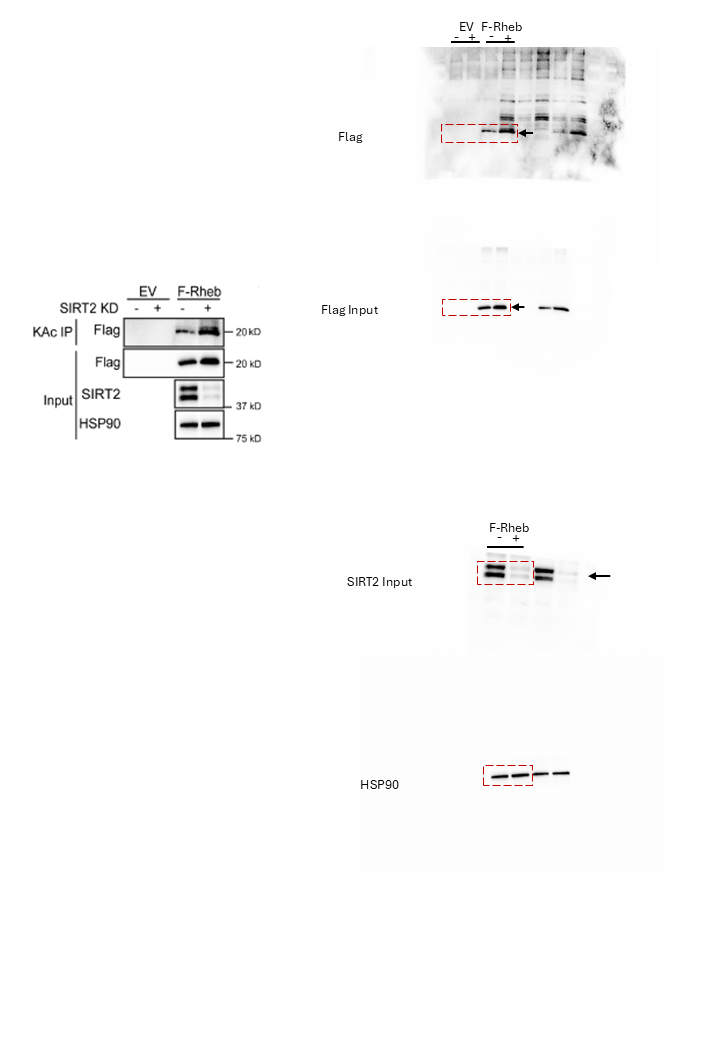

Supplement: Supplementary file 4 — Source data Fig. 3 [file 44319_2026_724_MOESM4_ESM.zip › Figure 3/3B/3B.tif]

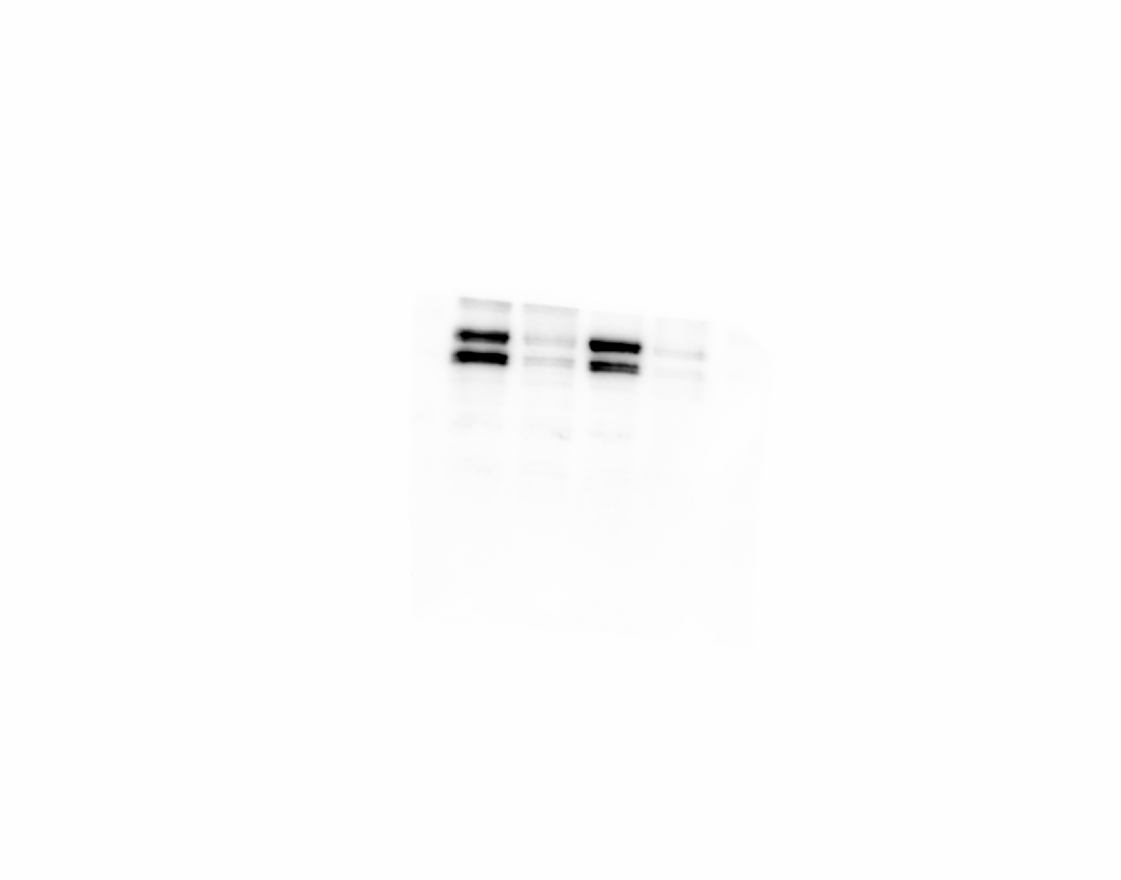

Supplement: Supplementary file 4 — Source data Fig. 3 [file 44319_2026_724_MOESM4_ESM.zip › Figure 3/3B/fig. 3B SIRT2.tif]

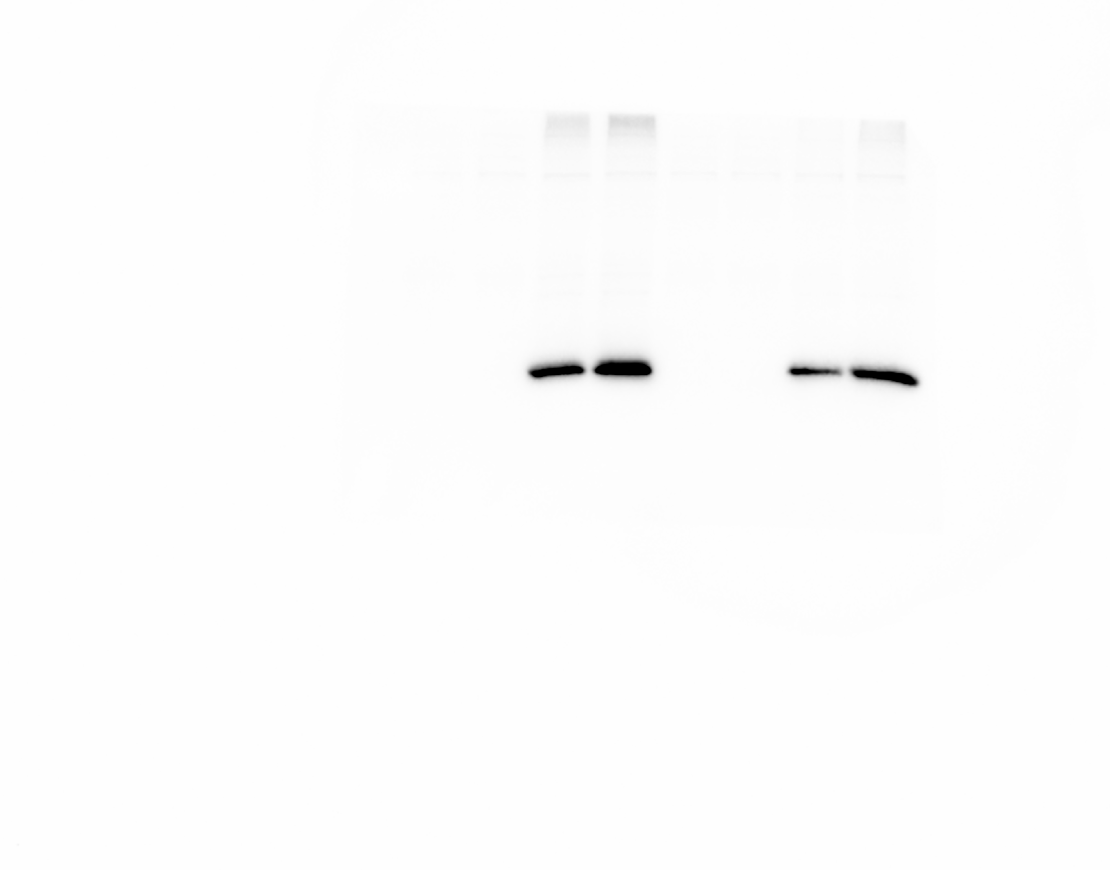

Supplement: Supplementary file 4 — Source data Fig. 3 [file 44319_2026_724_MOESM4_ESM.zip › Figure 3/3B/fig. 3B Flag.tif]

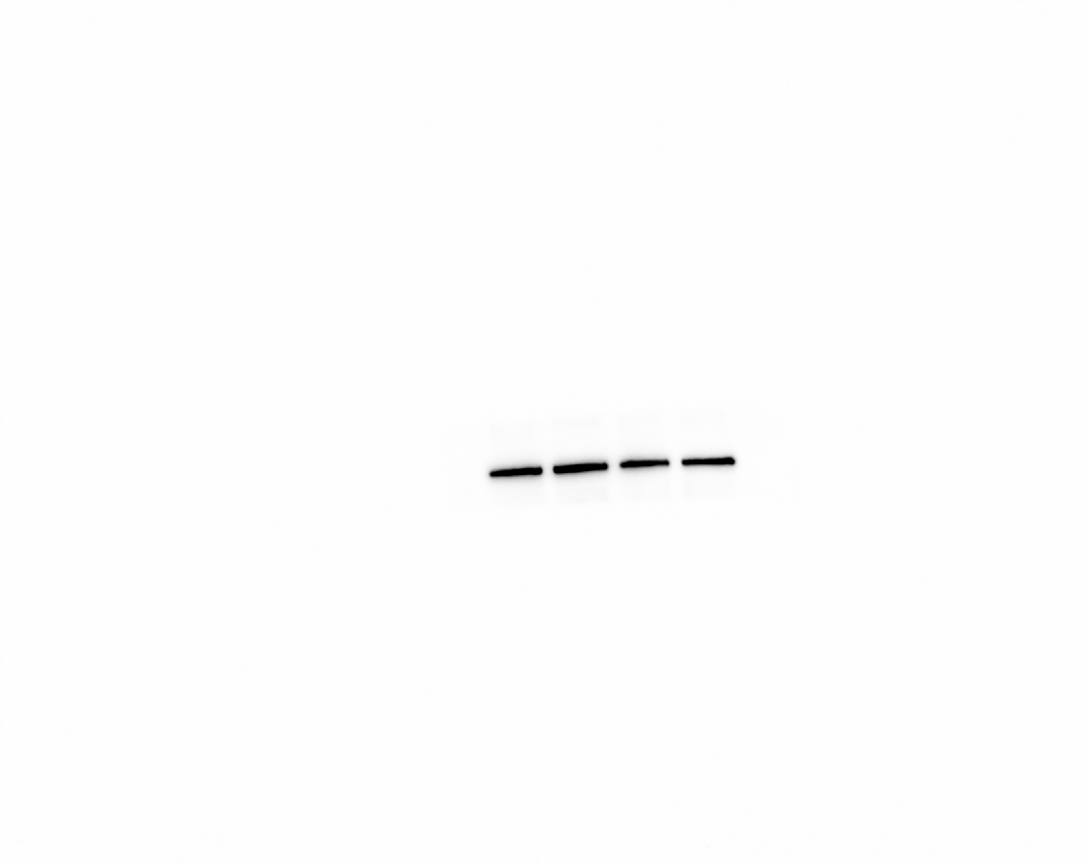

Supplement: Supplementary file 4 — Source data Fig. 3 [file 44319_2026_724_MOESM4_ESM.zip › Figure 3/3B/fig. 3B HSP90.tif]

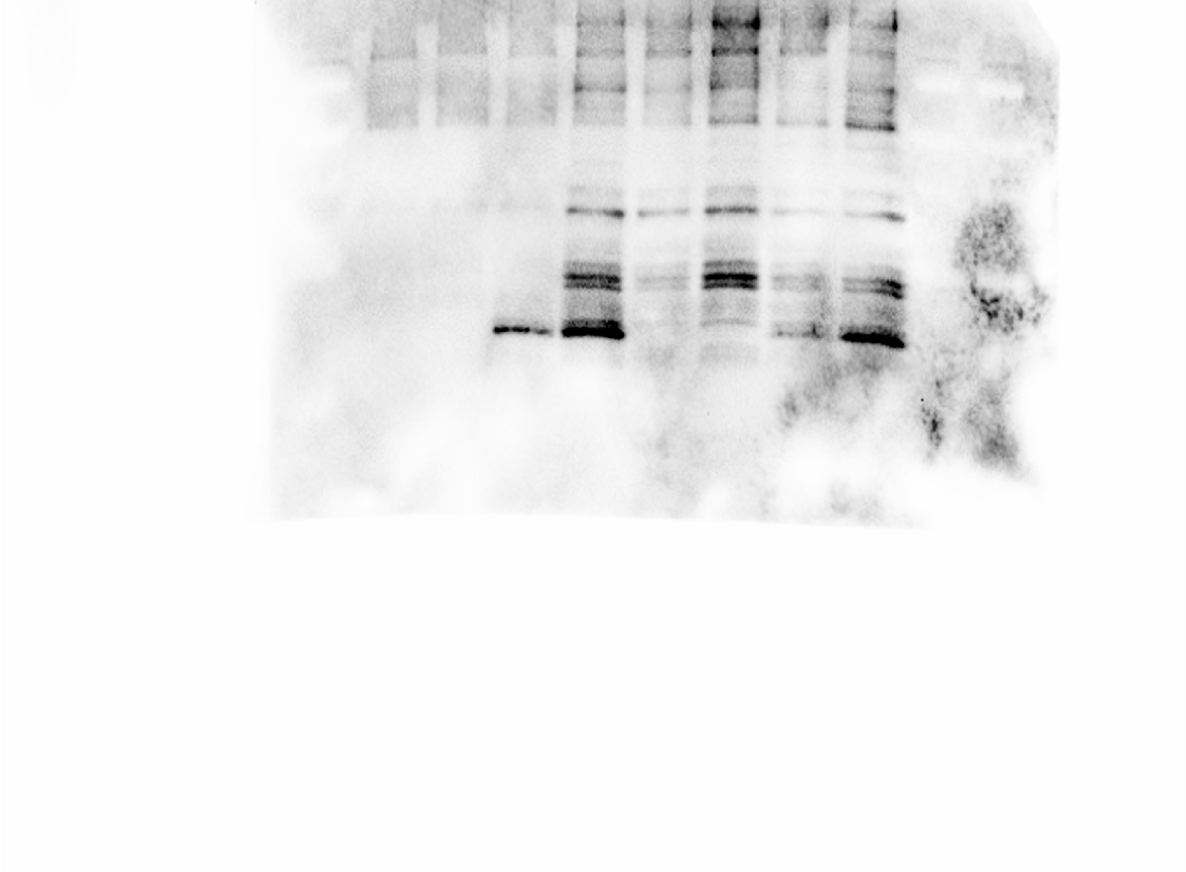

Supplement: Supplementary file 4 — Source data Fig. 3 [file 44319_2026_724_MOESM4_ESM.zip › Figure 3/3B/fig. 3B KAc IP.tif]

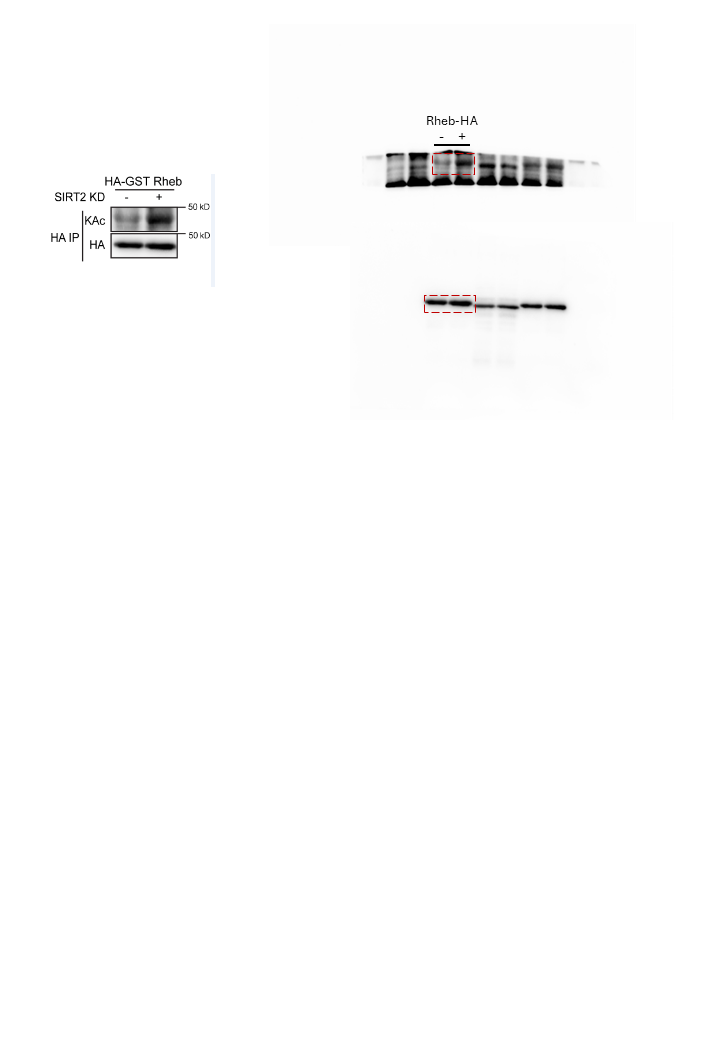

Supplement: Supplementary file 4 — Source data Fig. 3 [file 44319_2026_724_MOESM4_ESM.zip › Figure 3/3D/3D.tif]

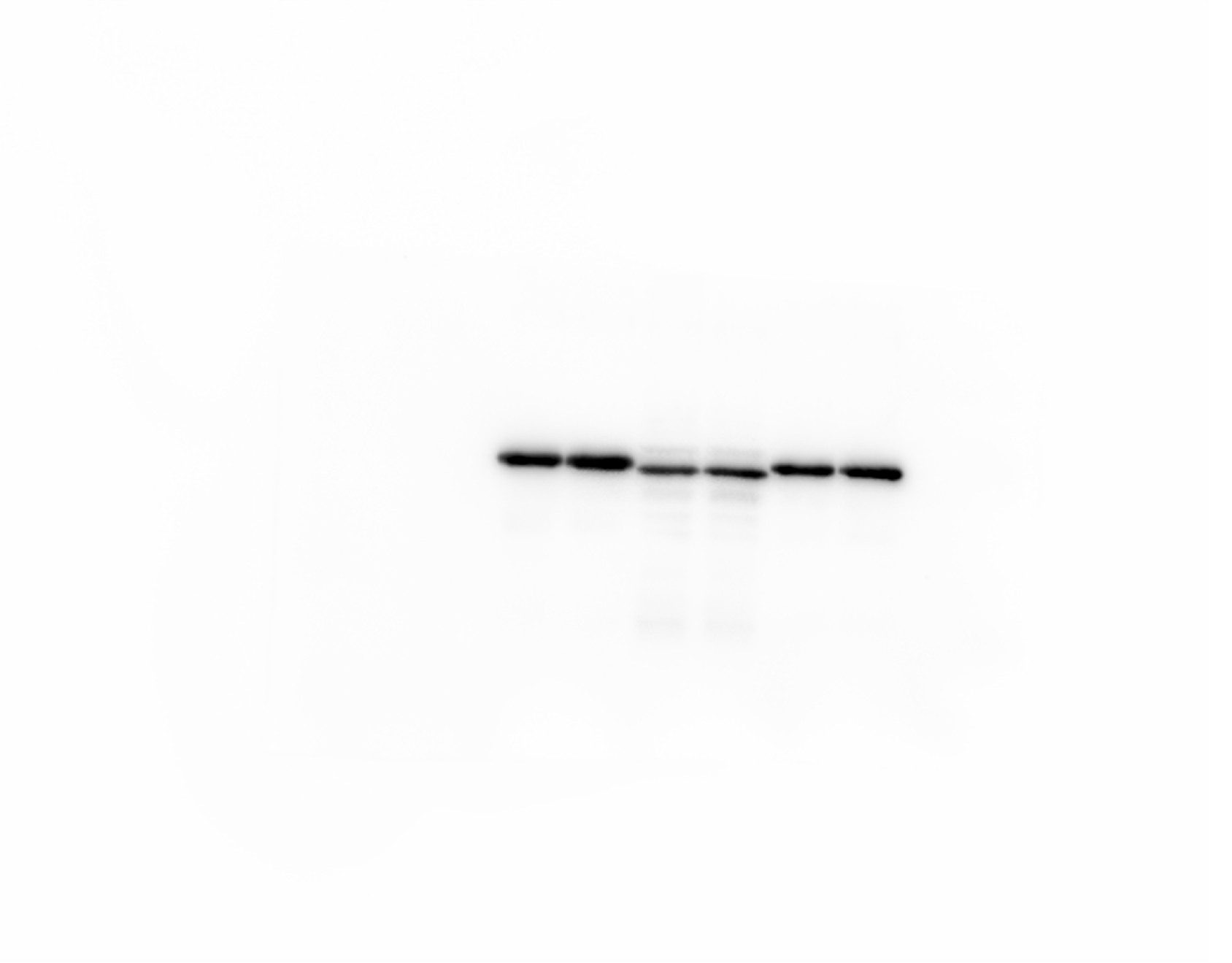

Supplement: Supplementary file 4 — Source data Fig. 3 [file 44319_2026_724_MOESM4_ESM.zip › Figure 3/3D/fig. 3D Rheb HA.tif]

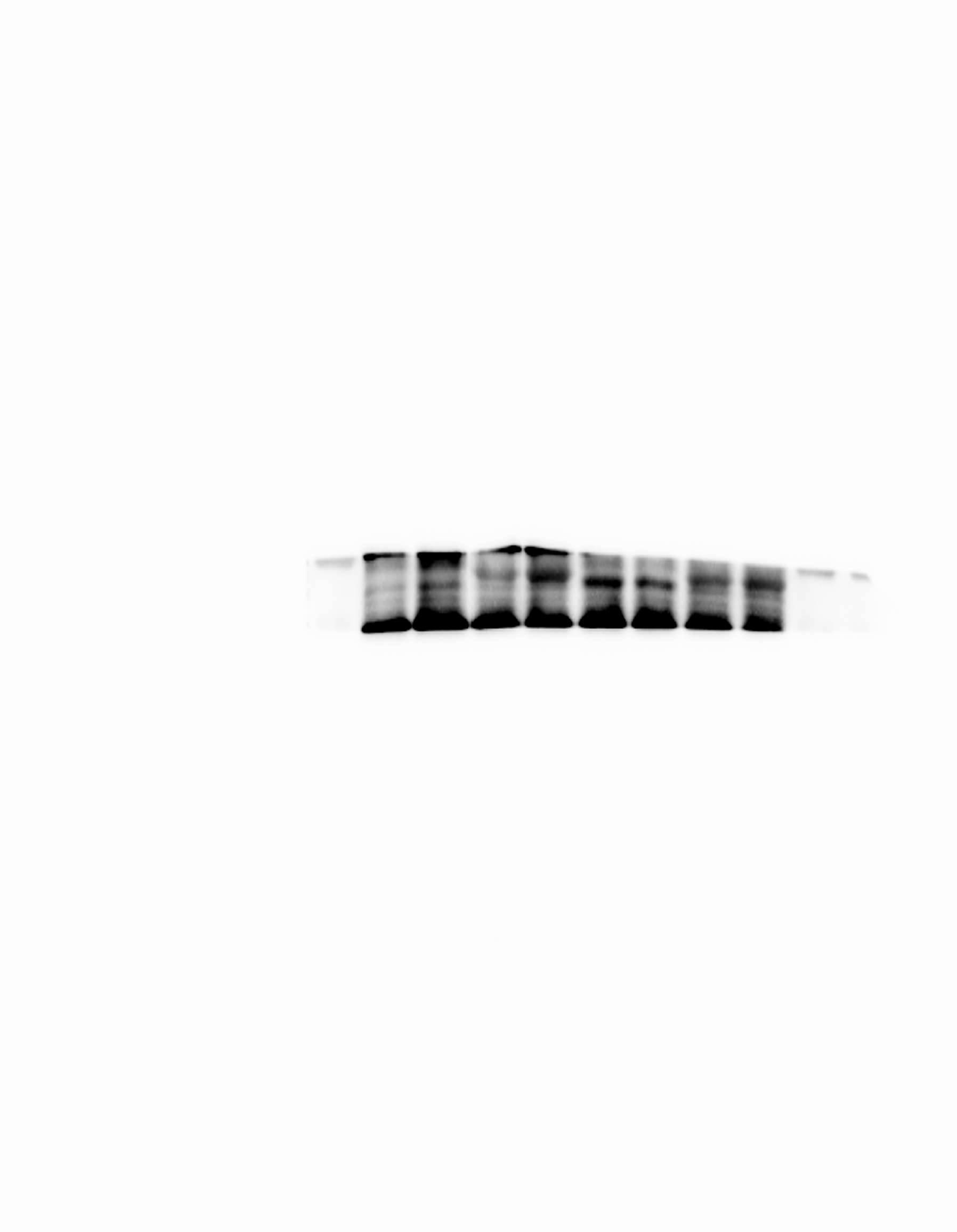

Supplement: Supplementary file 4 — Source data Fig. 3 [file 44319_2026_724_MOESM4_ESM.zip › Figure 3/3D/fig. 3D Rheb-HA Ac.tif]

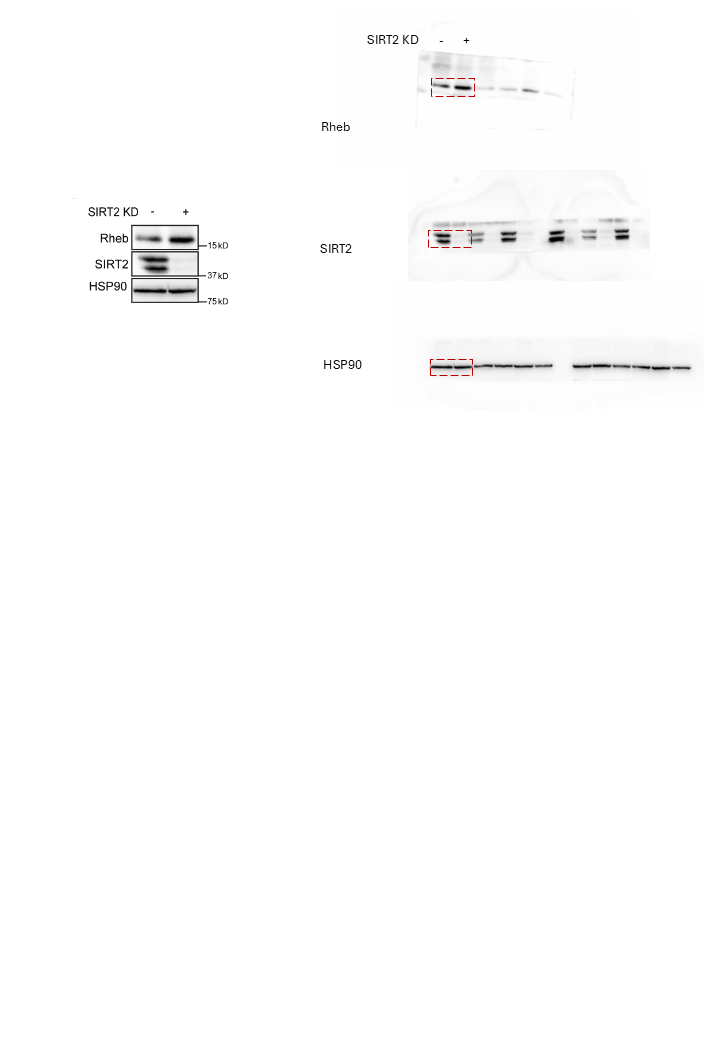

Supplement: Supplementary file 4 — Source data Fig. 3 [file 44319_2026_724_MOESM4_ESM.zip › Figure 3/3E/3E.tif]

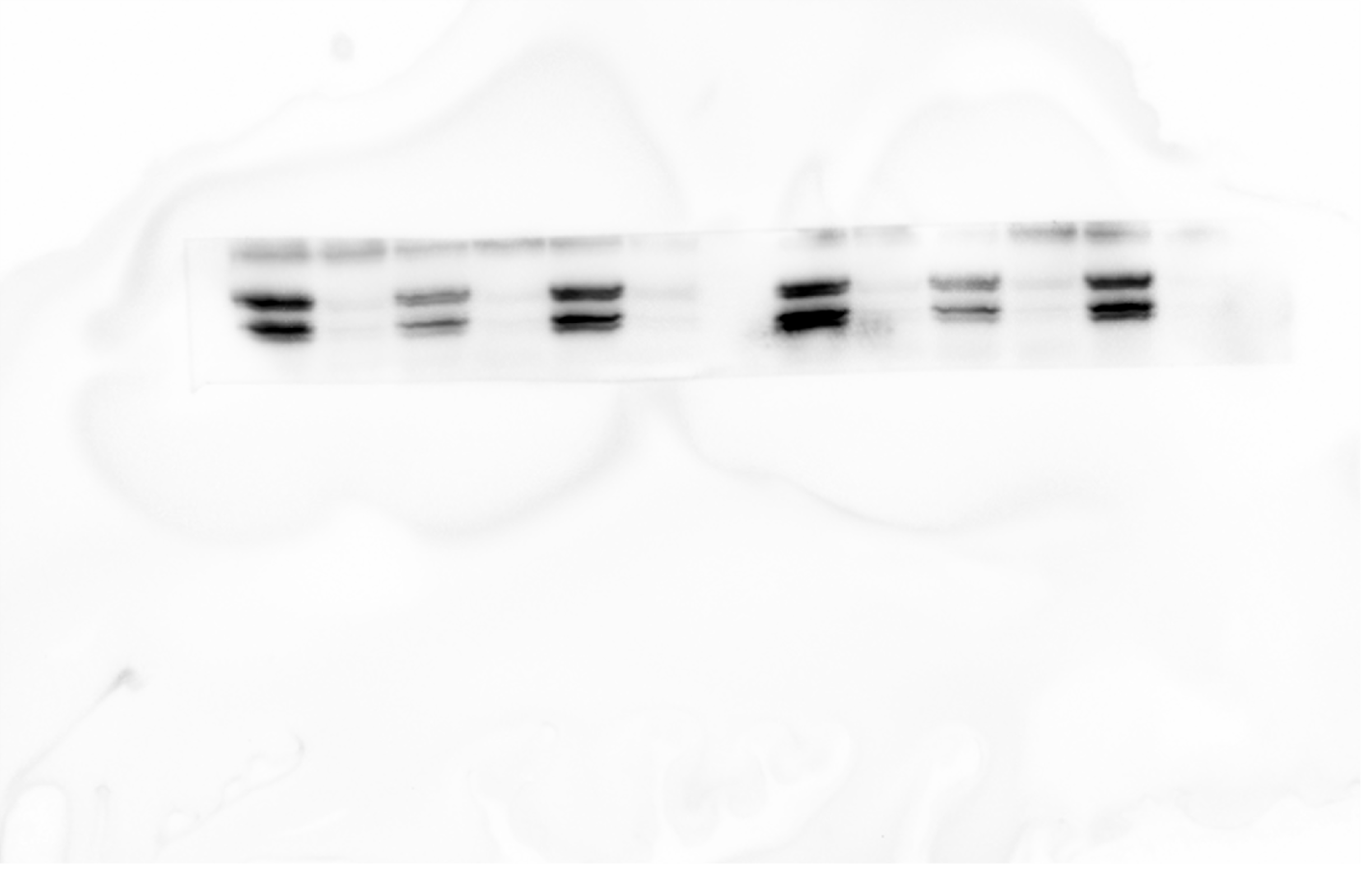

Supplement: Supplementary file 4 — Source data Fig. 3 [file 44319_2026_724_MOESM4_ESM.zip › Figure 3/3E/fig. 3E SIRT2.tif]

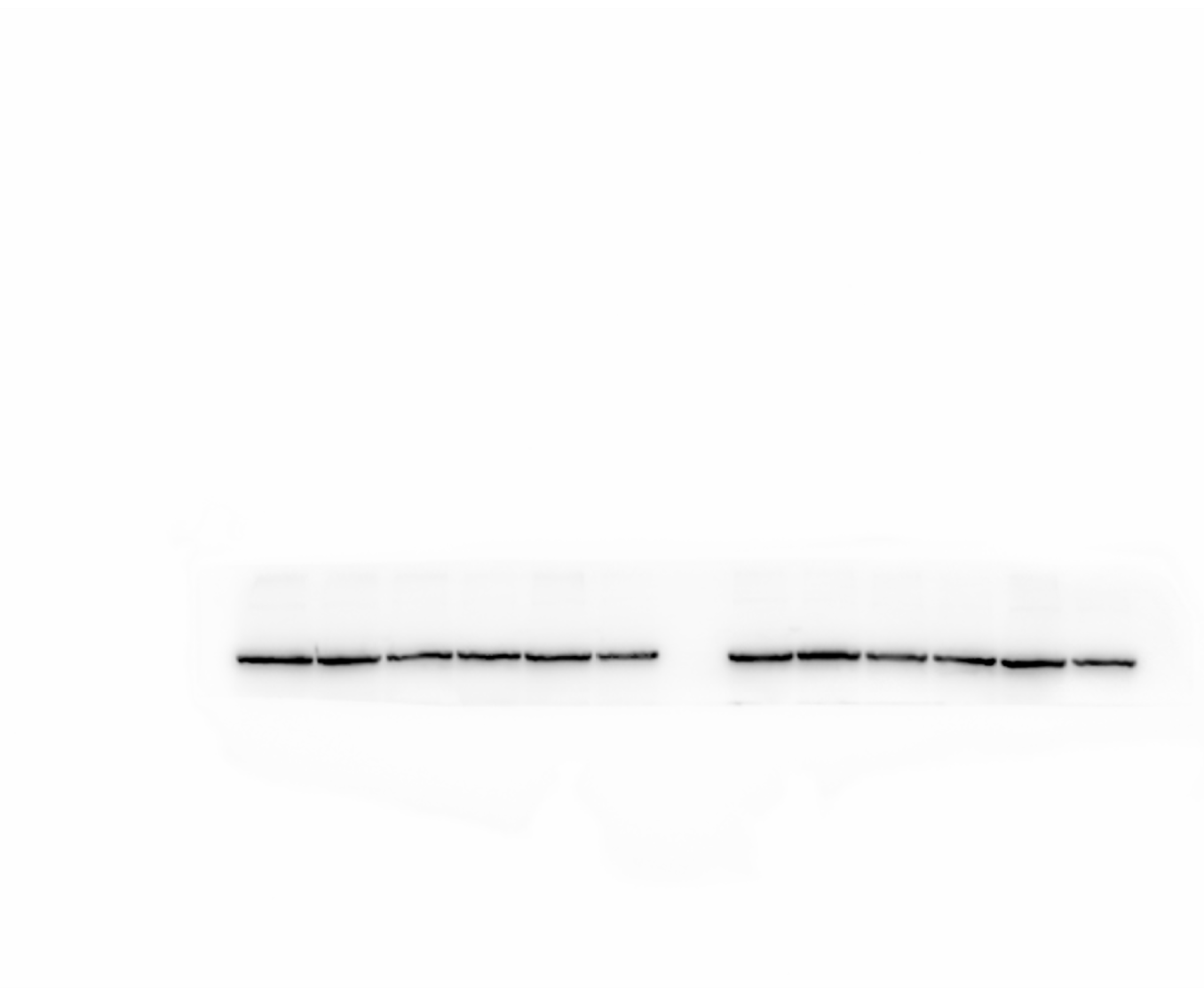

Supplement: Supplementary file 4 — Source data Fig. 3 [file 44319_2026_724_MOESM4_ESM.zip › Figure 3/3E/fig. 3E HSP90.tif]

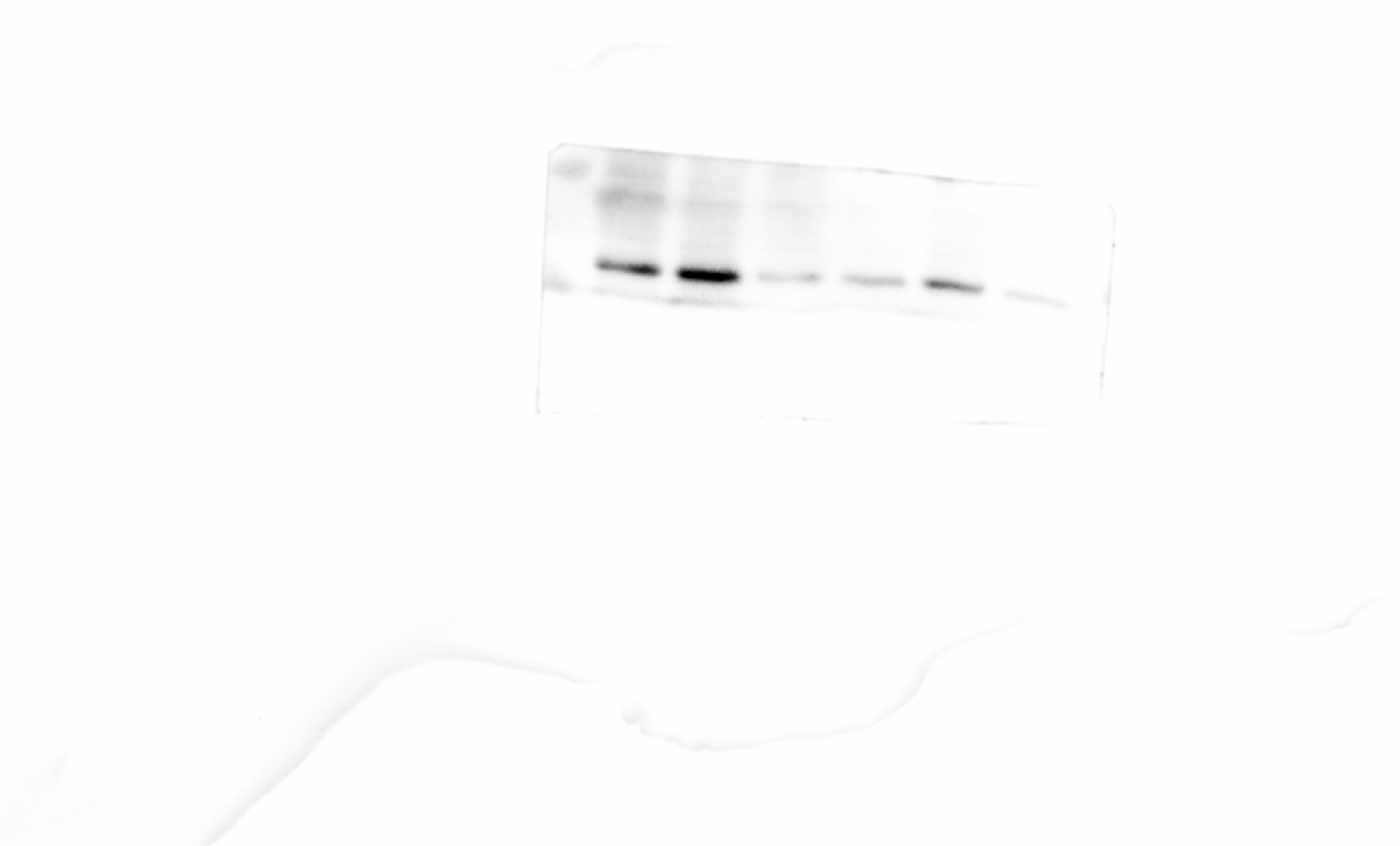

Supplement: Supplementary file 4 — Source data Fig. 3 [file 44319_2026_724_MOESM4_ESM.zip › Figure 3/3E/fig. 3E Rheb.tif]

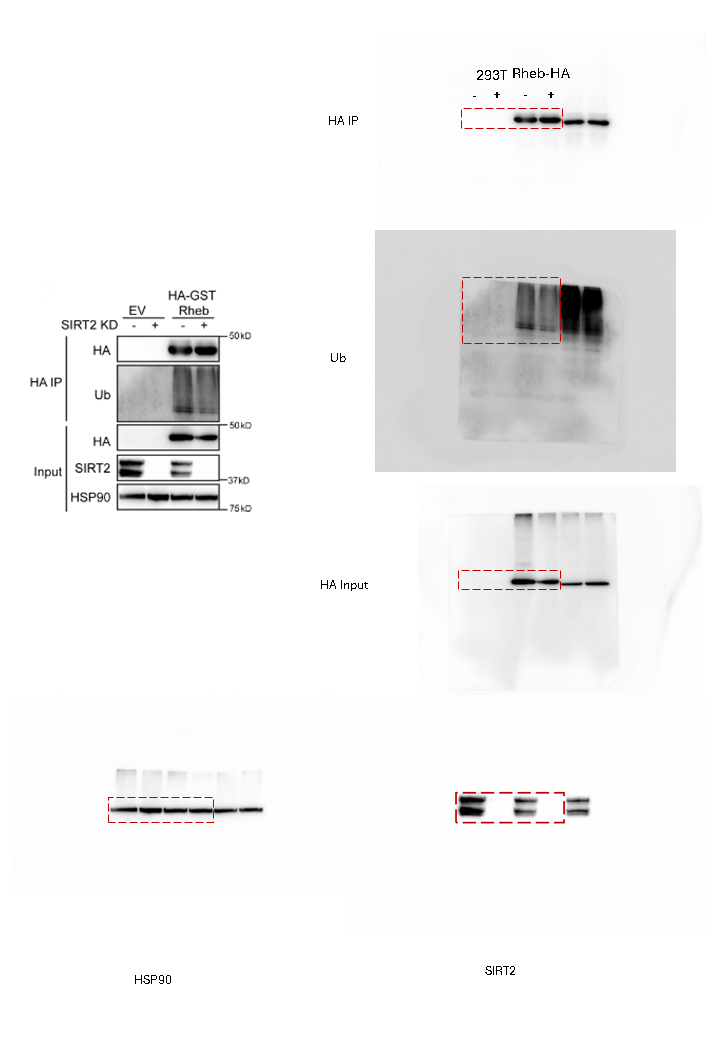

Supplement: Supplementary file 4 — Source data Fig. 3 [file 44319_2026_724_MOESM4_ESM.zip › Figure 3/3G/3G.tif]

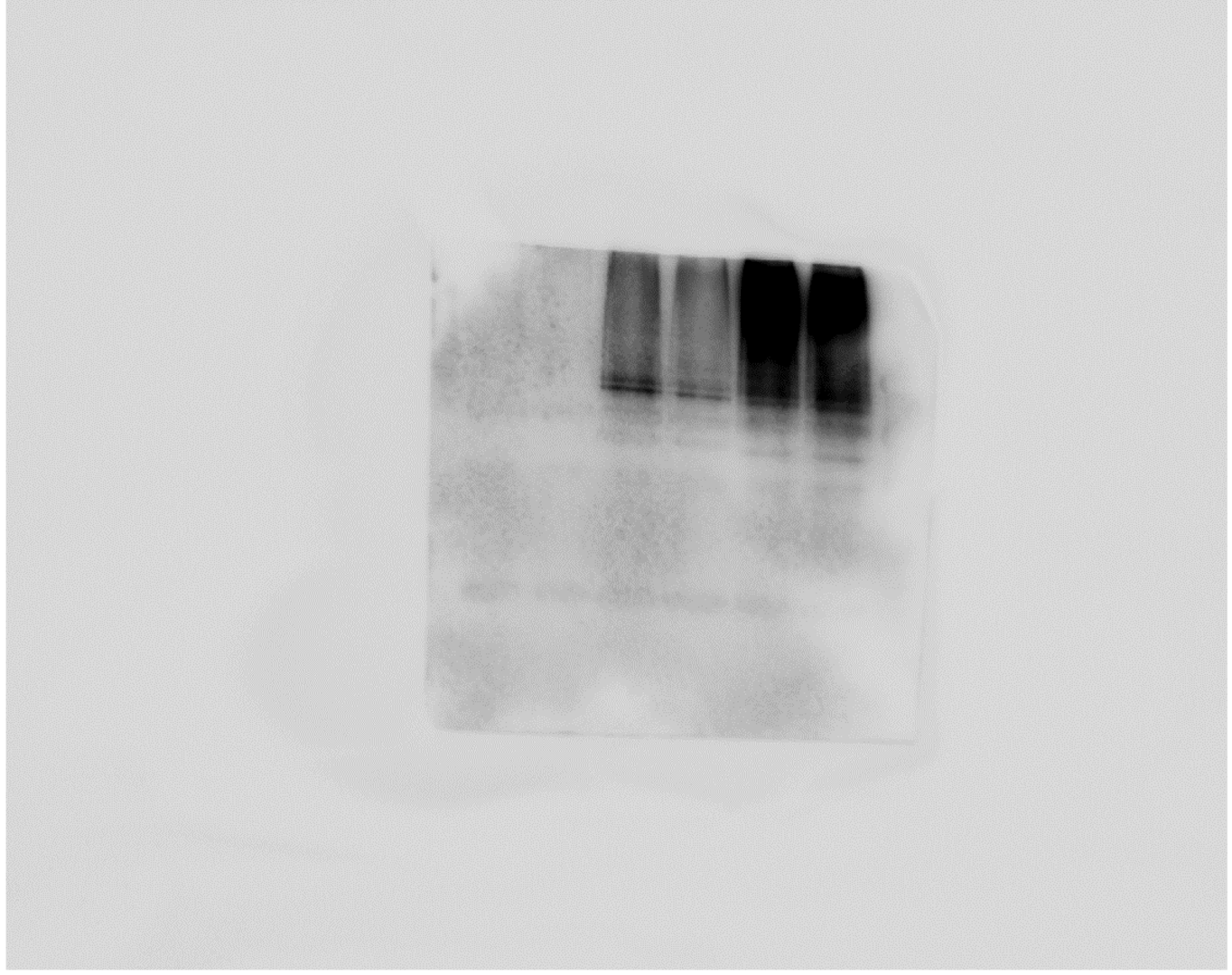

Supplement: Supplementary file 4 — Source data Fig. 3 [file 44319_2026_724_MOESM4_ESM.zip › Figure 3/3G/fig. 3G Ub.tif]

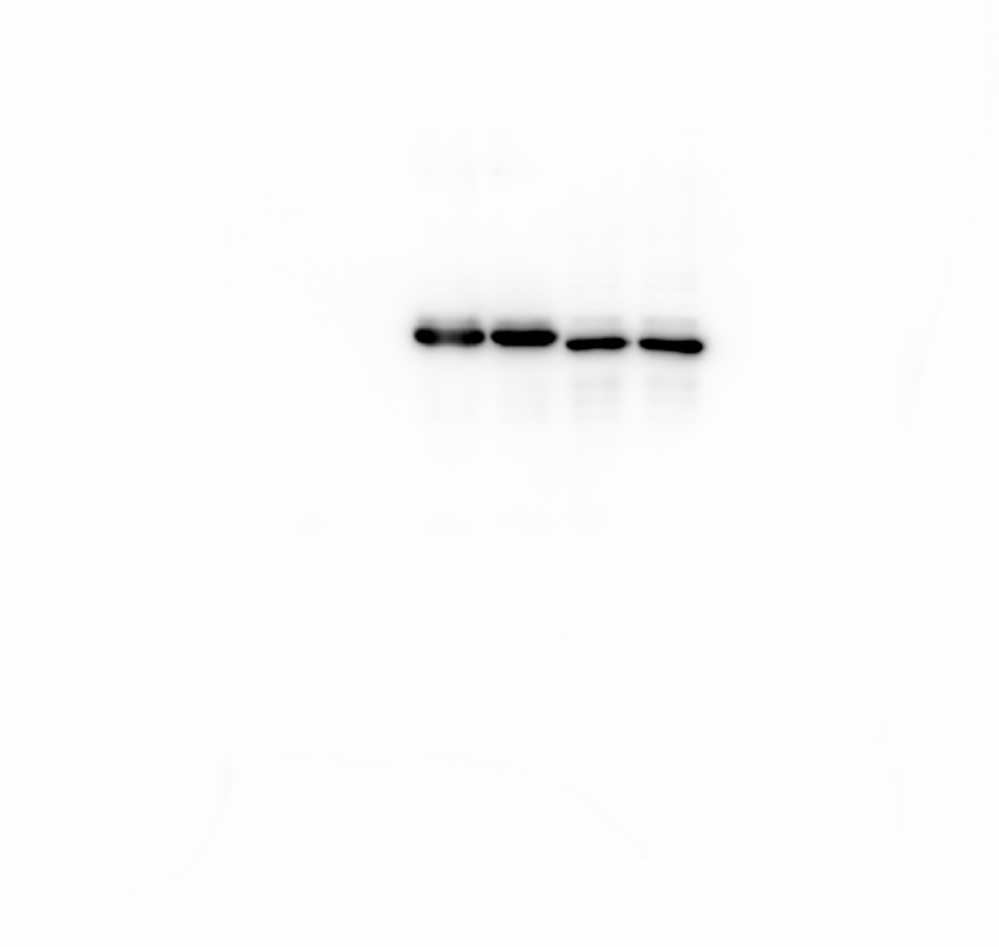

Supplement: Supplementary file 4 — Source data Fig. 3 [file 44319_2026_724_MOESM4_ESM.zip › Figure 3/3G/fig. 3G HA IP.tif]

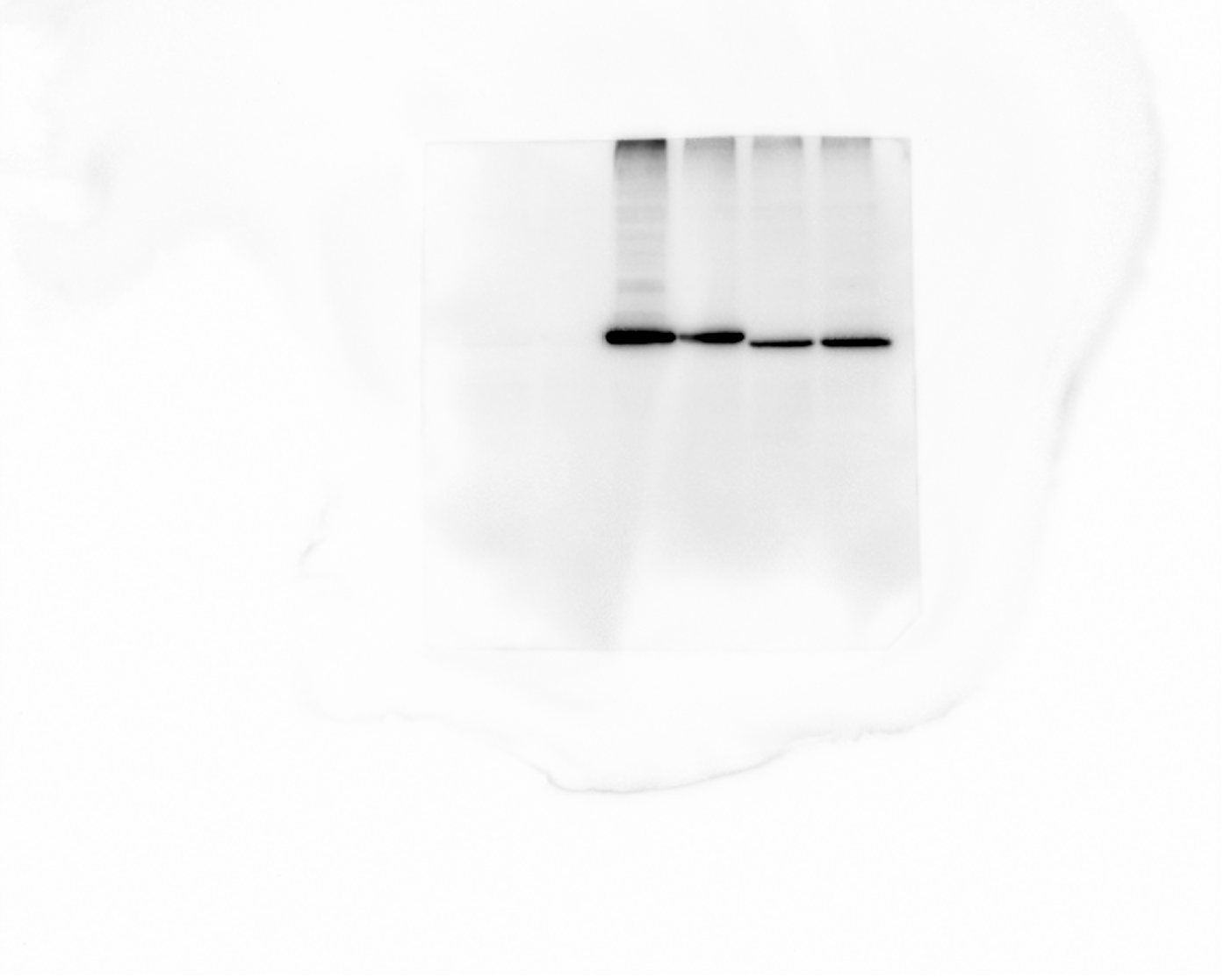

Supplement: Supplementary file 4 — Source data Fig. 3 [file 44319_2026_724_MOESM4_ESM.zip › Figure 3/3G/fig. 3G HA input.tif]

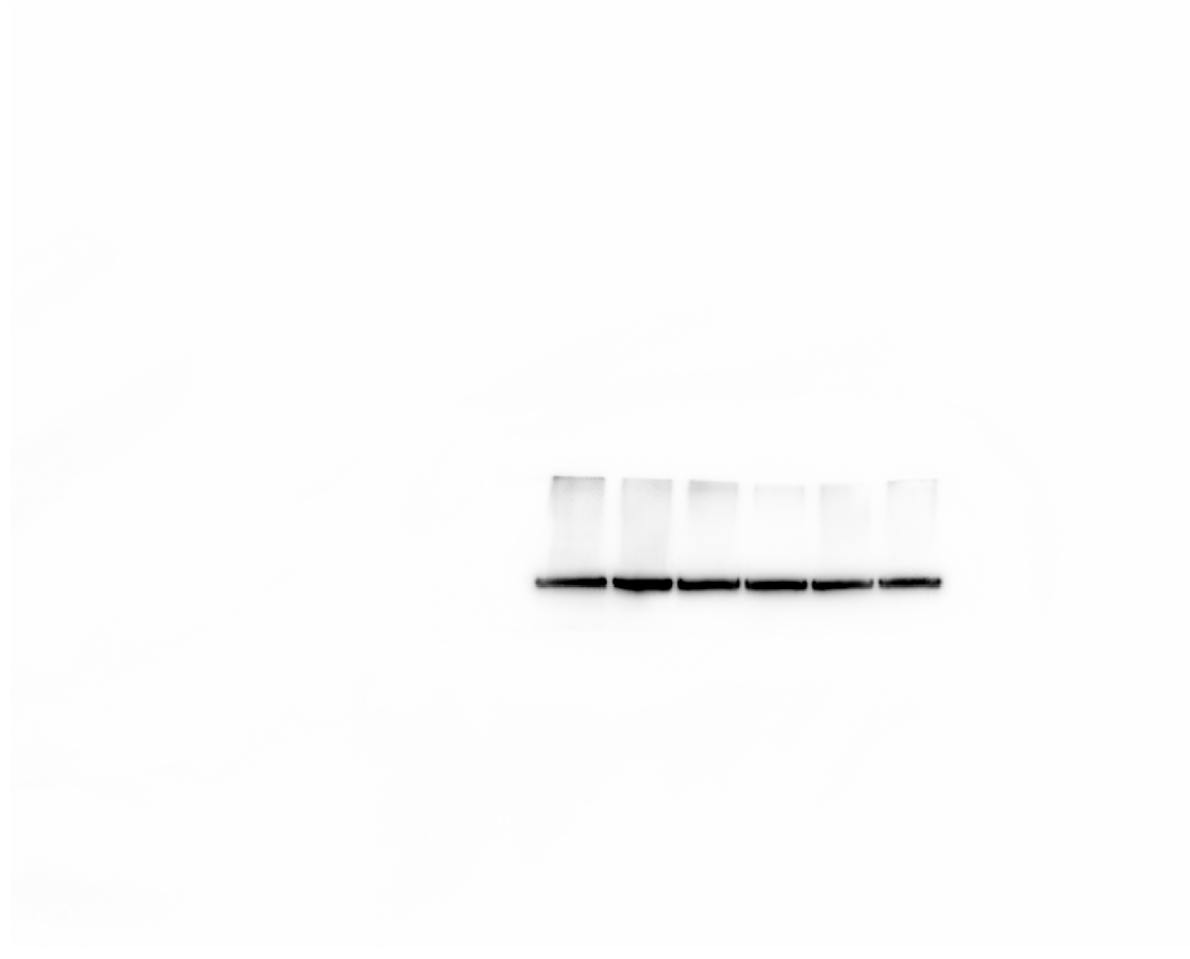

Supplement: Supplementary file 4 — Source data Fig. 3 [file 44319_2026_724_MOESM4_ESM.zip › Figure 3/3G/fig. 3G HSP input.tif]

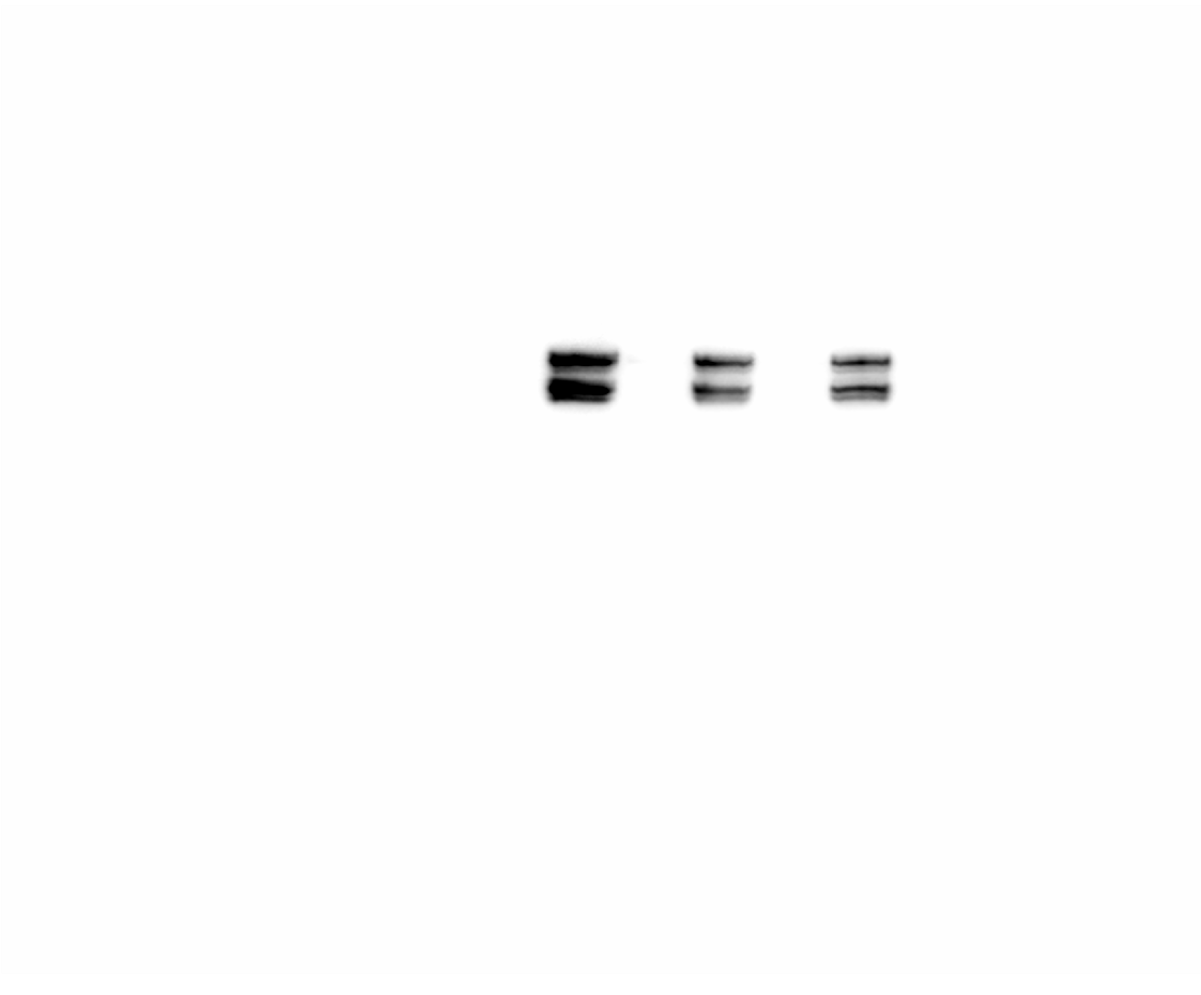

Supplement: Supplementary file 4 — Source data Fig. 3 [file 44319_2026_724_MOESM4_ESM.zip › Figure 3/3G/fig. 3G SIRT2 Input.tif]

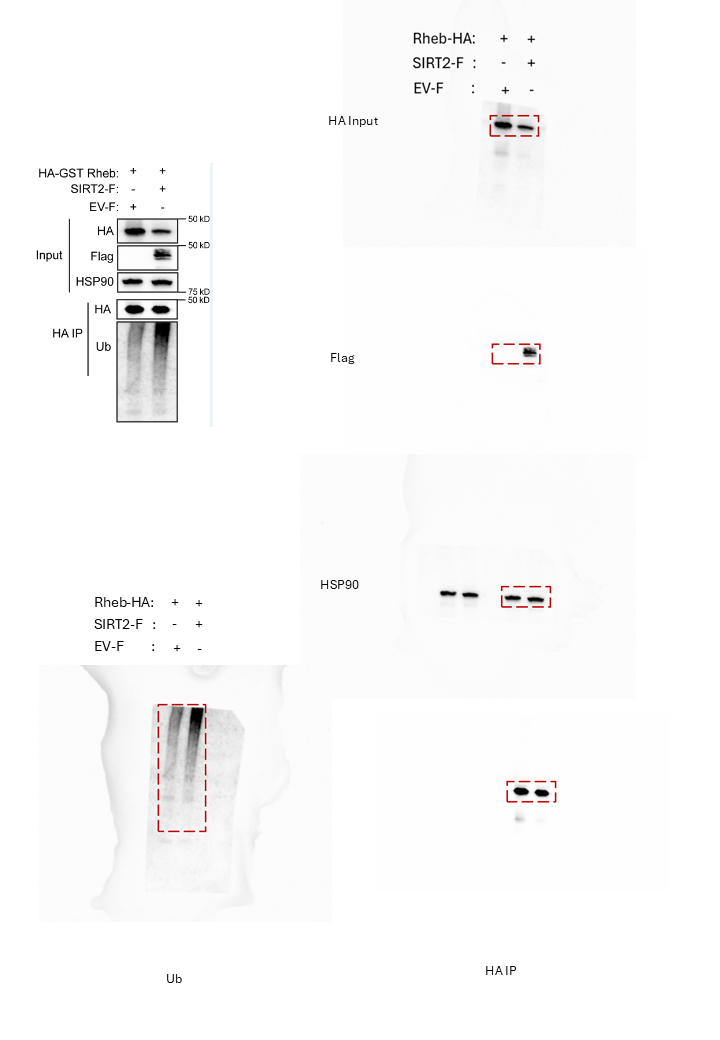

Supplement: Supplementary file 4 — Source data Fig. 3 [file 44319_2026_724_MOESM4_ESM.zip › Figure 3/3H/3H.tif]

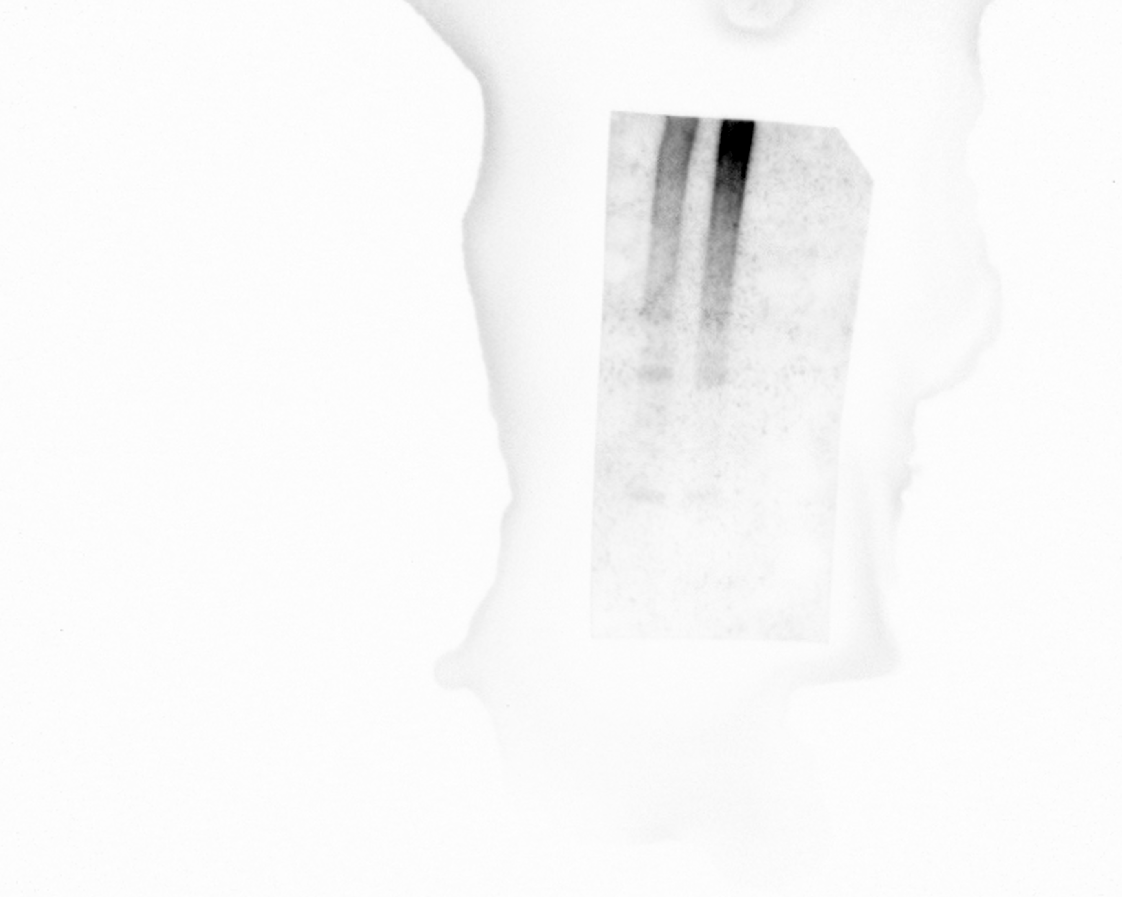

Supplement: Supplementary file 4 — Source data Fig. 3 [file 44319_2026_724_MOESM4_ESM.zip › Figure 3/3H/fig. 3H Ub.tif]

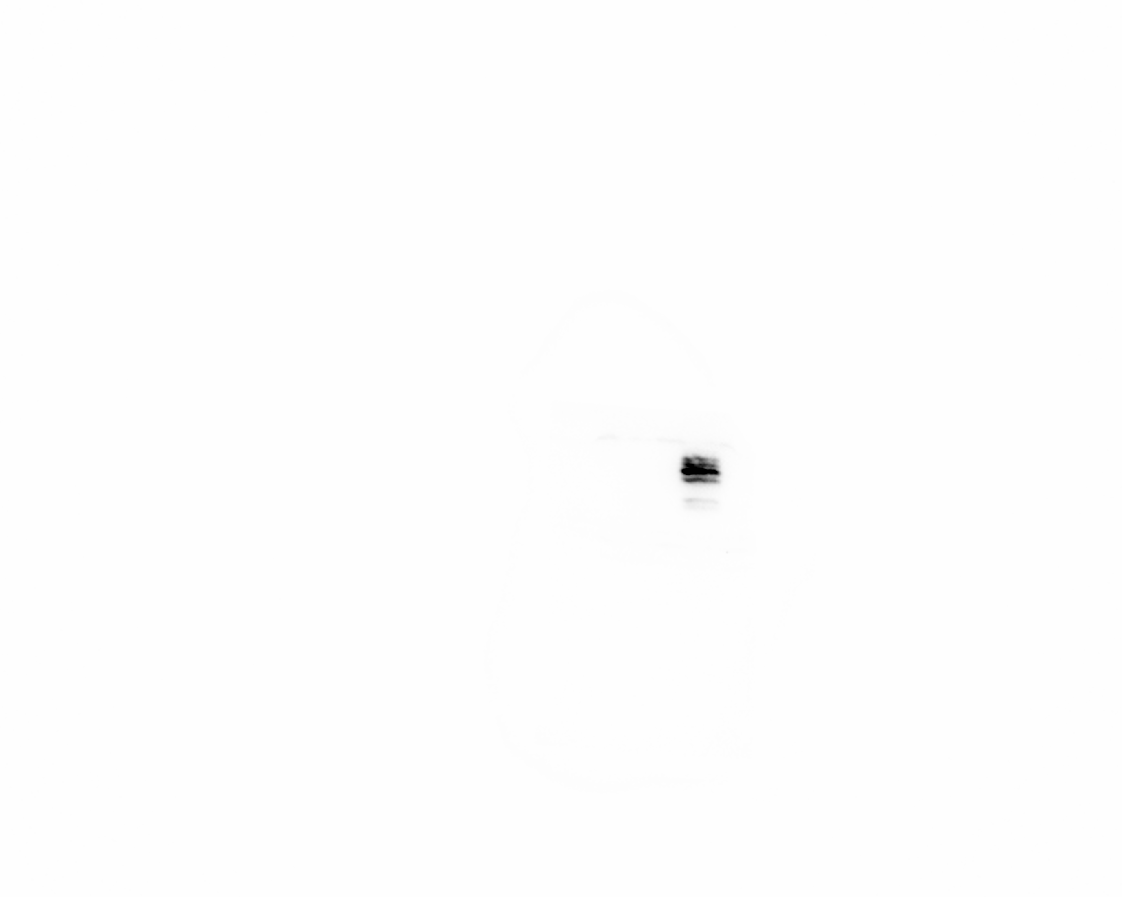

Supplement: Supplementary file 4 — Source data Fig. 3 [file 44319_2026_724_MOESM4_ESM.zip › Figure 3/3H/fig. 3H Flag Input.tif]

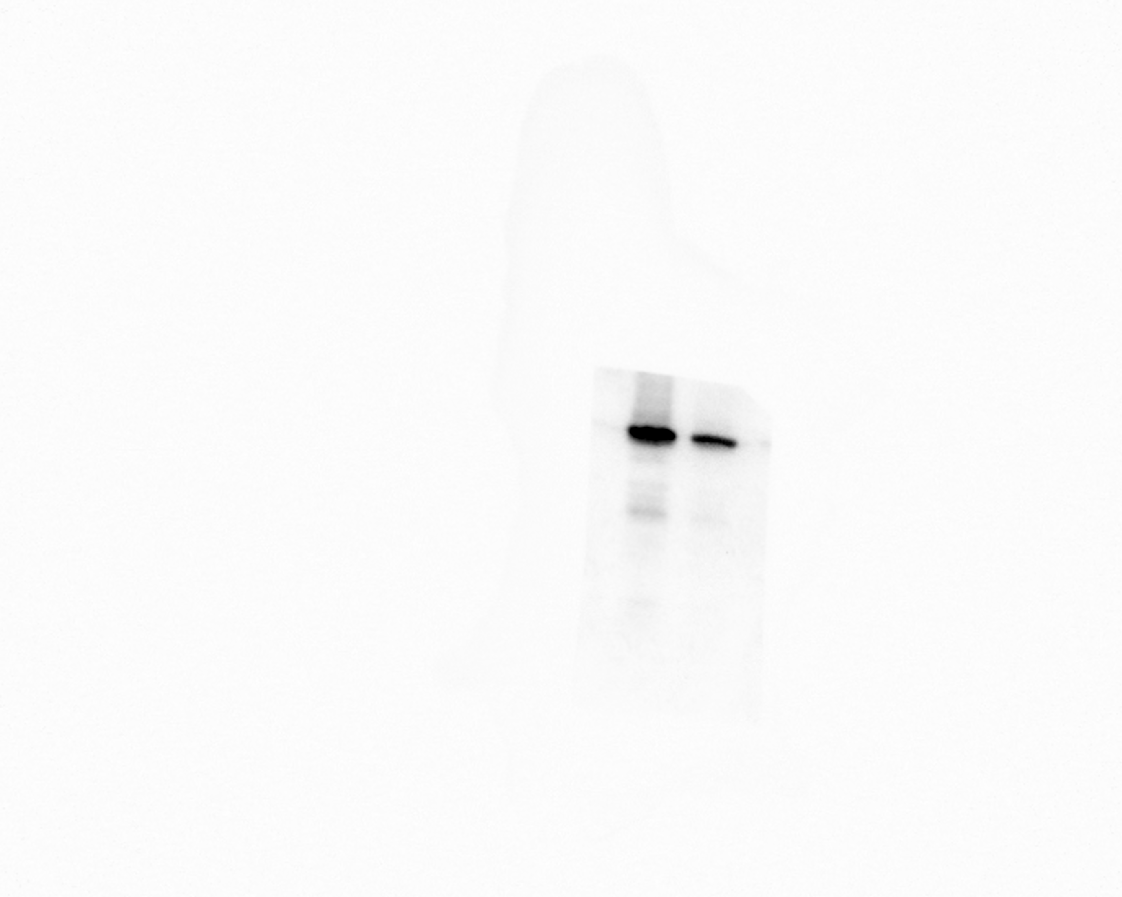

Supplement: Supplementary file 4 — Source data Fig. 3 [file 44319_2026_724_MOESM4_ESM.zip › Figure 3/3H/fig. 3H HA Input.tif]

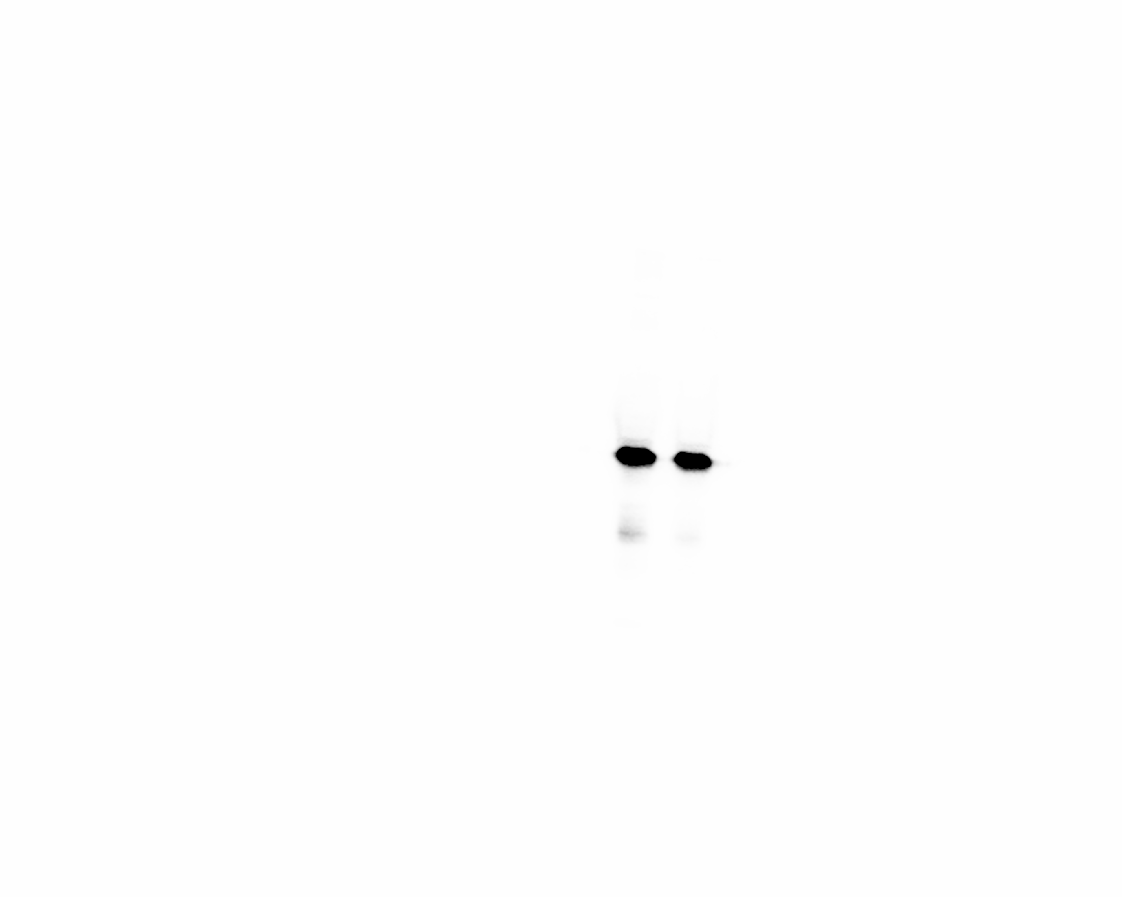

Supplement: Supplementary file 4 — Source data Fig. 3 [file 44319_2026_724_MOESM4_ESM.zip › Figure 3/3H/fig. 3H HA IP.tif]

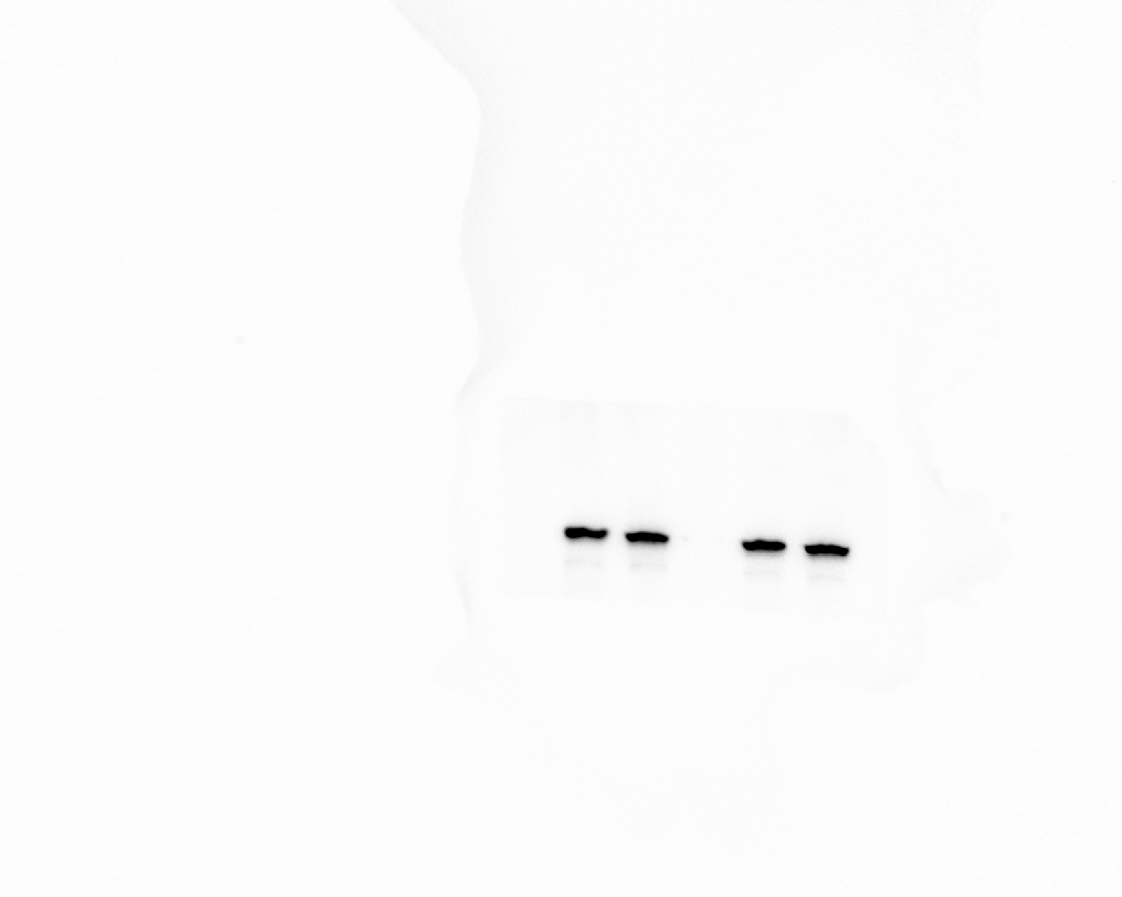

Supplement: Supplementary file 4 — Source data Fig. 3 [file 44319_2026_724_MOESM4_ESM.zip › Figure 3/3H/fig. 3H HSP Input.tif]

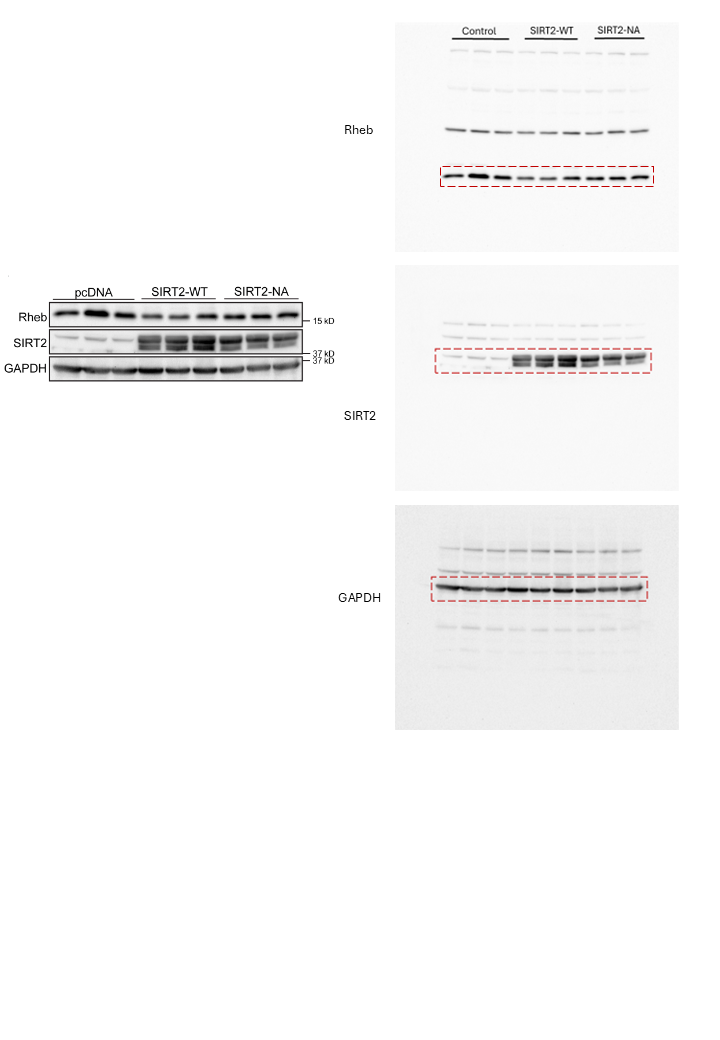

Supplement: Supplementary file 4 — Source data Fig. 3 [file 44319_2026_724_MOESM4_ESM.zip › Figure 3/3I/3I.tif]

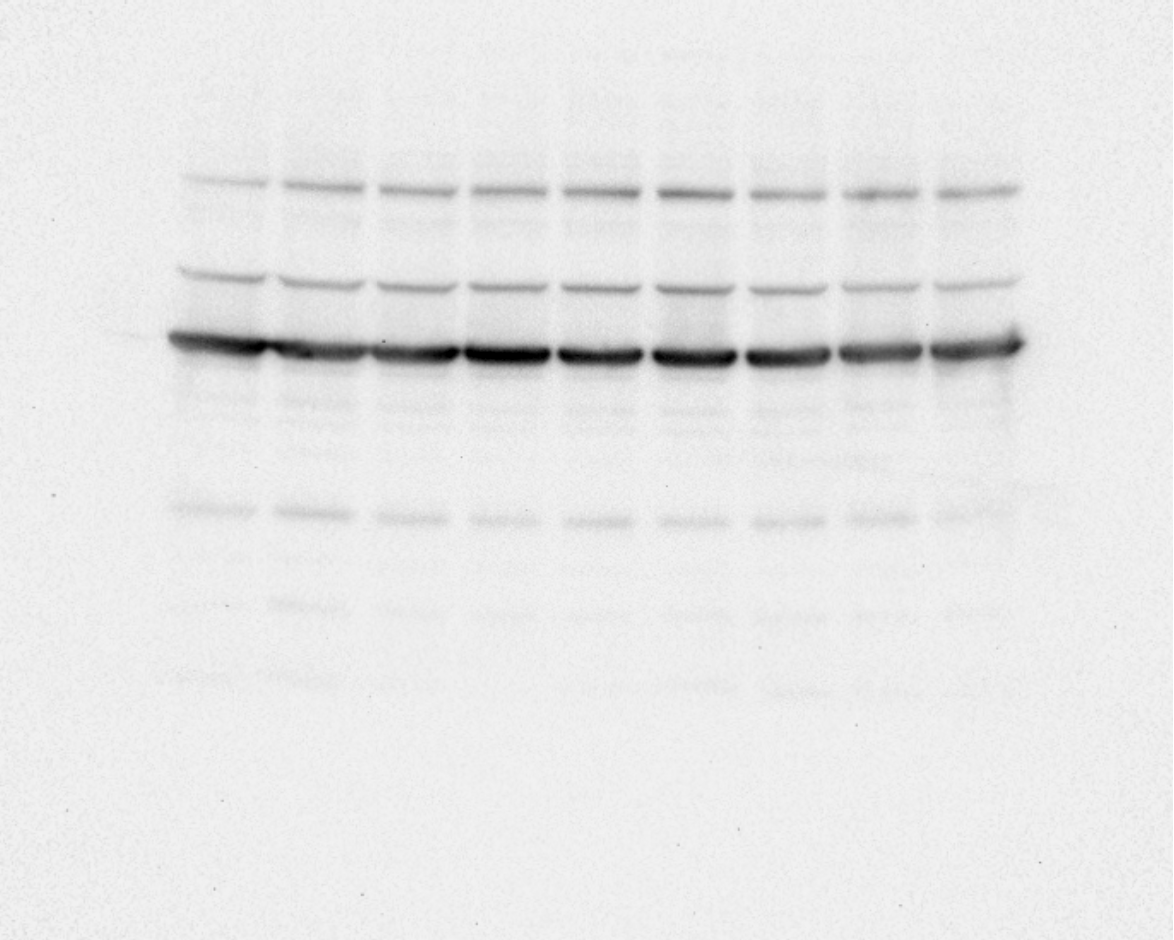

Supplement: Supplementary file 4 — Source data Fig. 3 [file 44319_2026_724_MOESM4_ESM.zip › Figure 3/3I/fig. 3I GAPDH.tif]

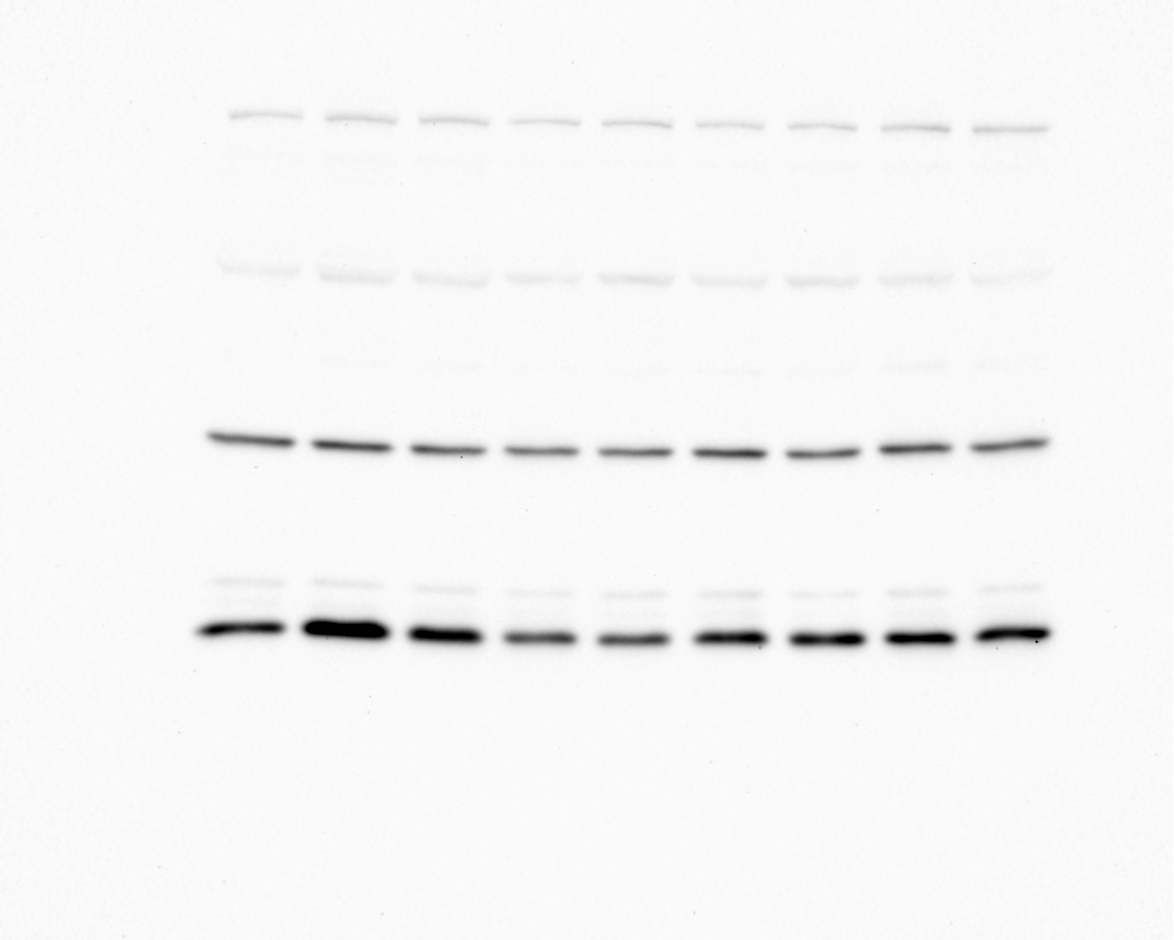

Supplement: Supplementary file 4 — Source data Fig. 3 [file 44319_2026_724_MOESM4_ESM.zip › Figure 3/3I/fig. 3I Rheb.tif]

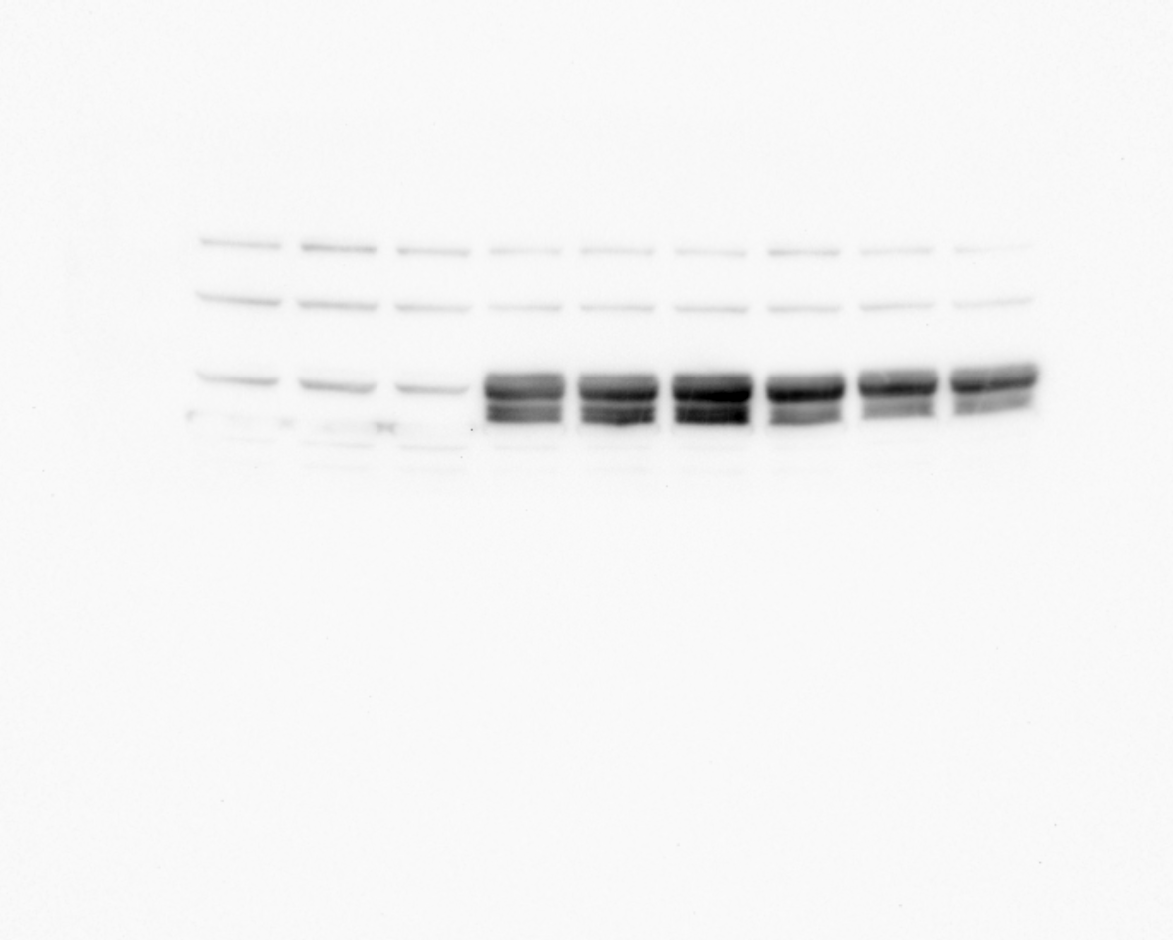

Supplement: Supplementary file 4 — Source data Fig. 3 [file 44319_2026_724_MOESM4_ESM.zip › Figure 3/3I/fig. 3I SIRT2.tif]

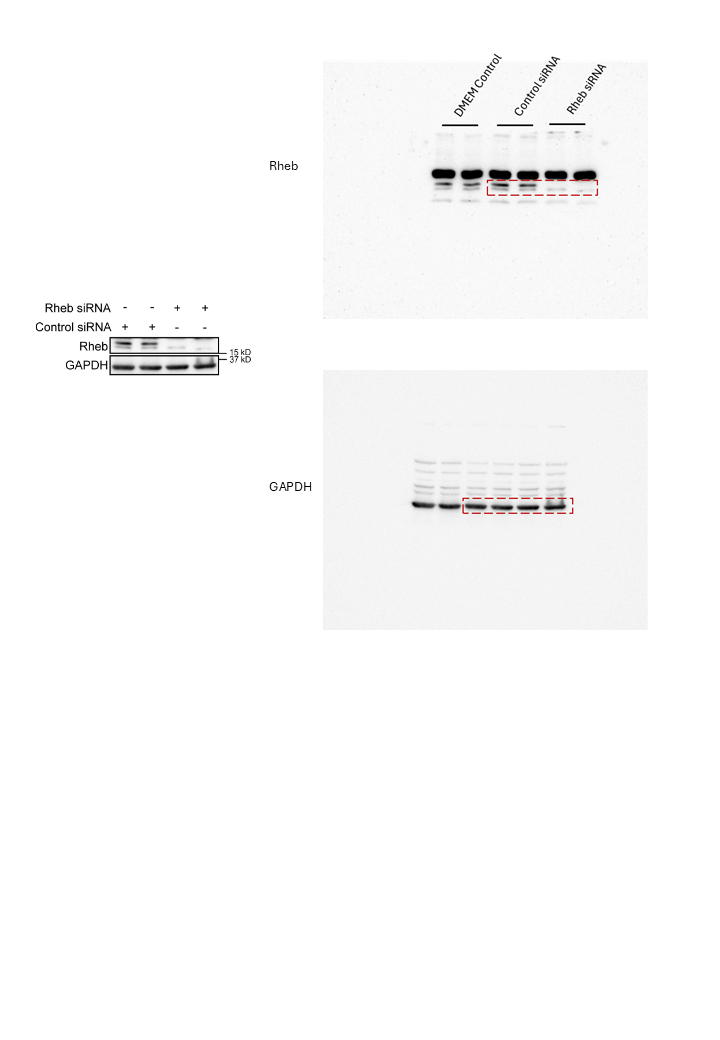

Supplement: Supplementary file 4 — Source data Fig. 3 [file 44319_2026_724_MOESM4_ESM.zip › Figure 3/3L/3L.tif]

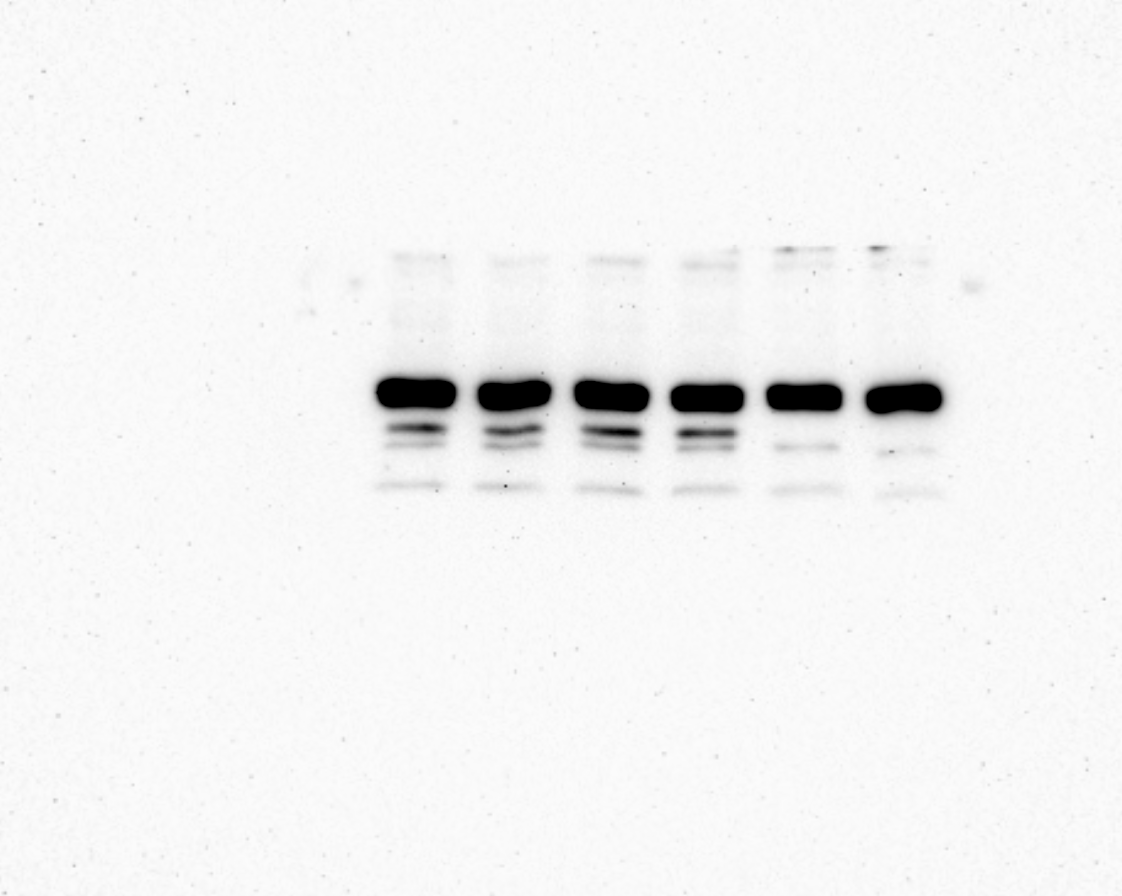

Supplement: Supplementary file 4 — Source data Fig. 3 [file 44319_2026_724_MOESM4_ESM.zip › Figure 3/3L/fig. 3L Rheb.tif]

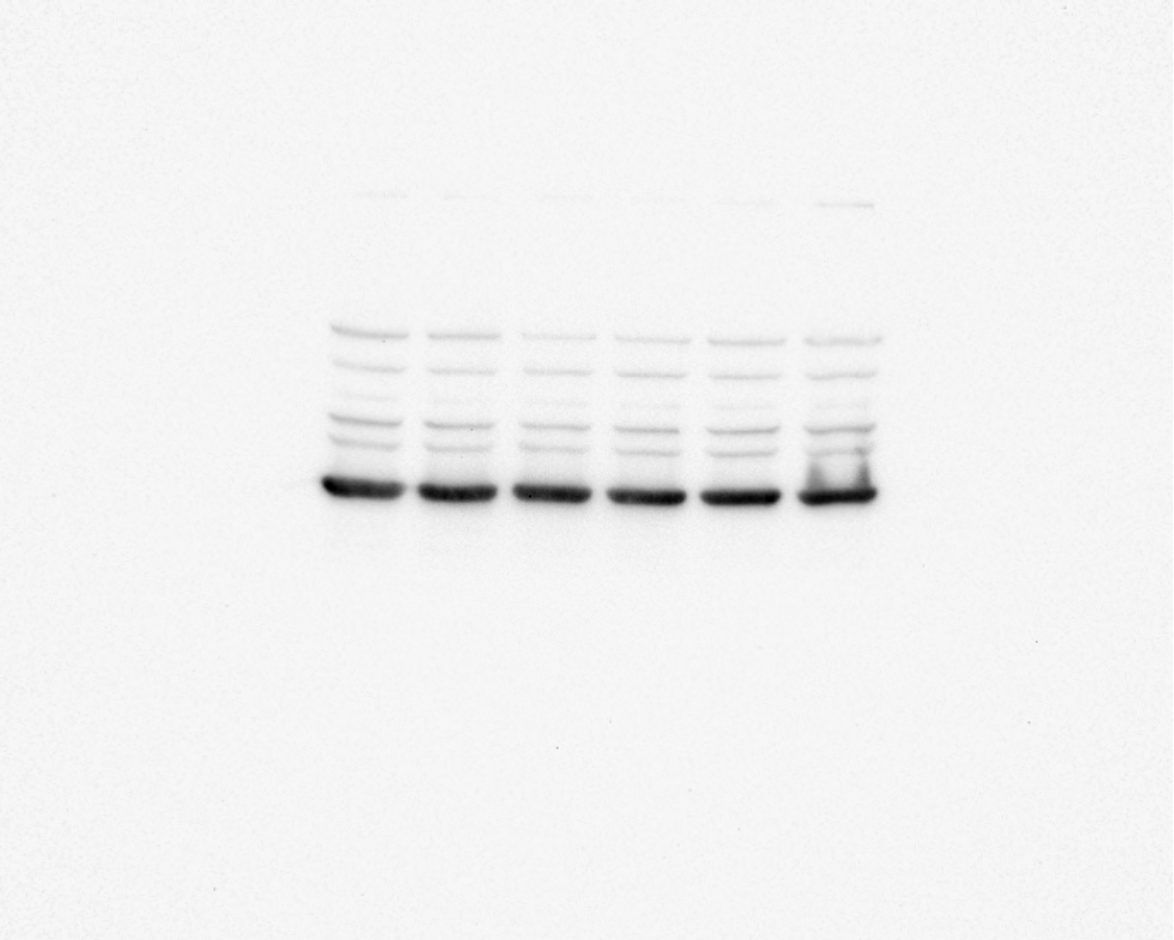

Supplement: Supplementary file 4 — Source data Fig. 3 [file 44319_2026_724_MOESM4_ESM.zip › Figure 3/3L/fig. 3L GAPDH.tif]

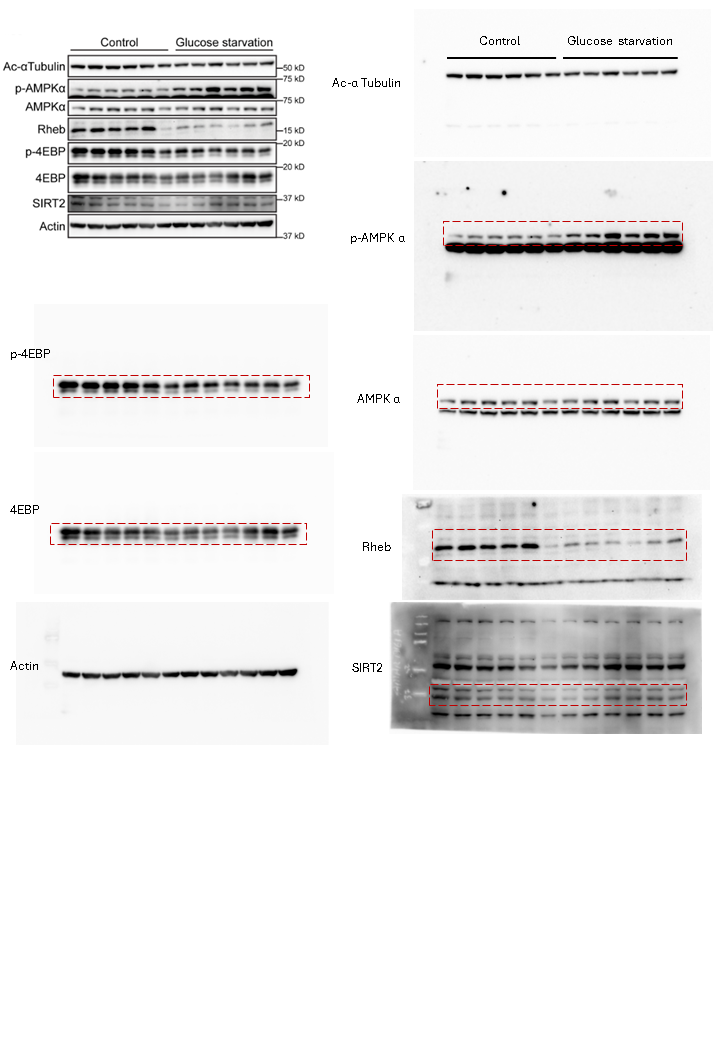

Supplement: Supplementary file 4 — Source data Fig. 3 [file 44319_2026_724_MOESM4_ESM.zip › Figure 3/3M/3M.tif]

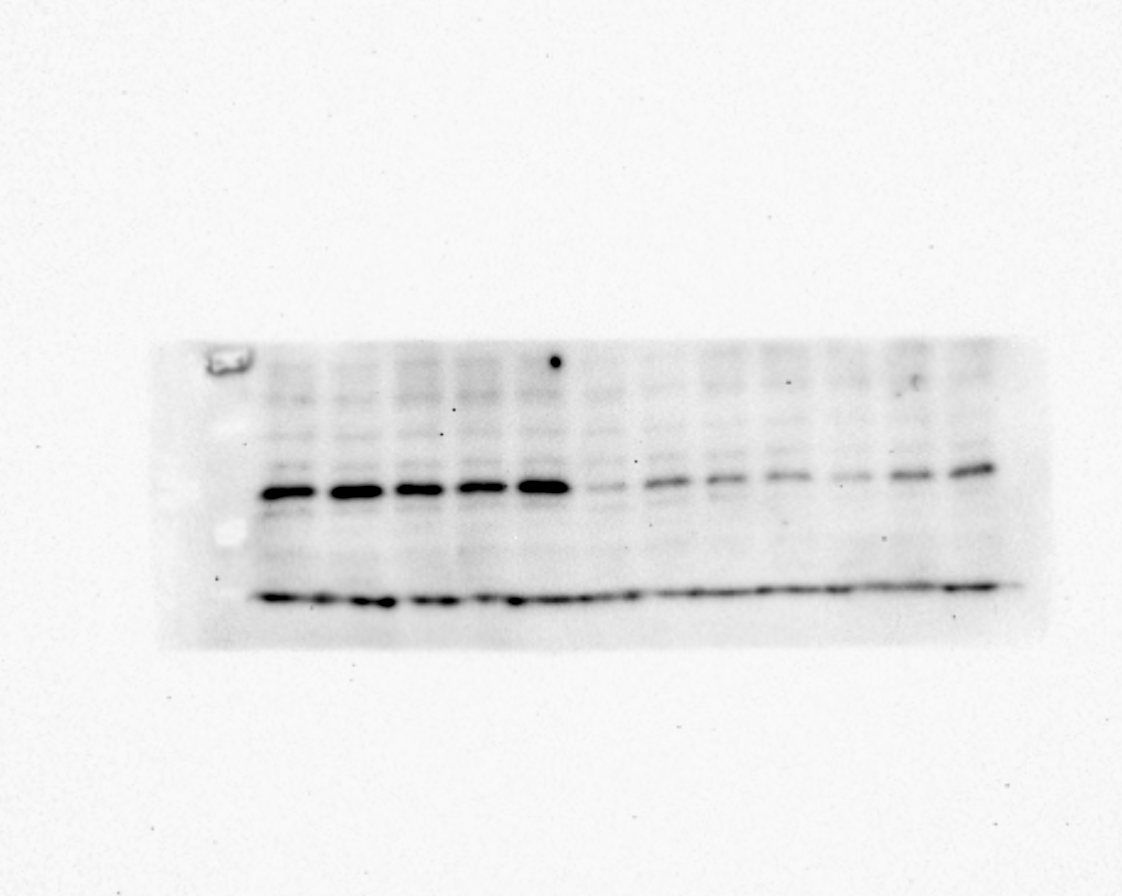

Supplement: Supplementary file 4 — Source data Fig. 3 [file 44319_2026_724_MOESM4_ESM.zip › Figure 3/3M/fig. 3M rheb.tif]

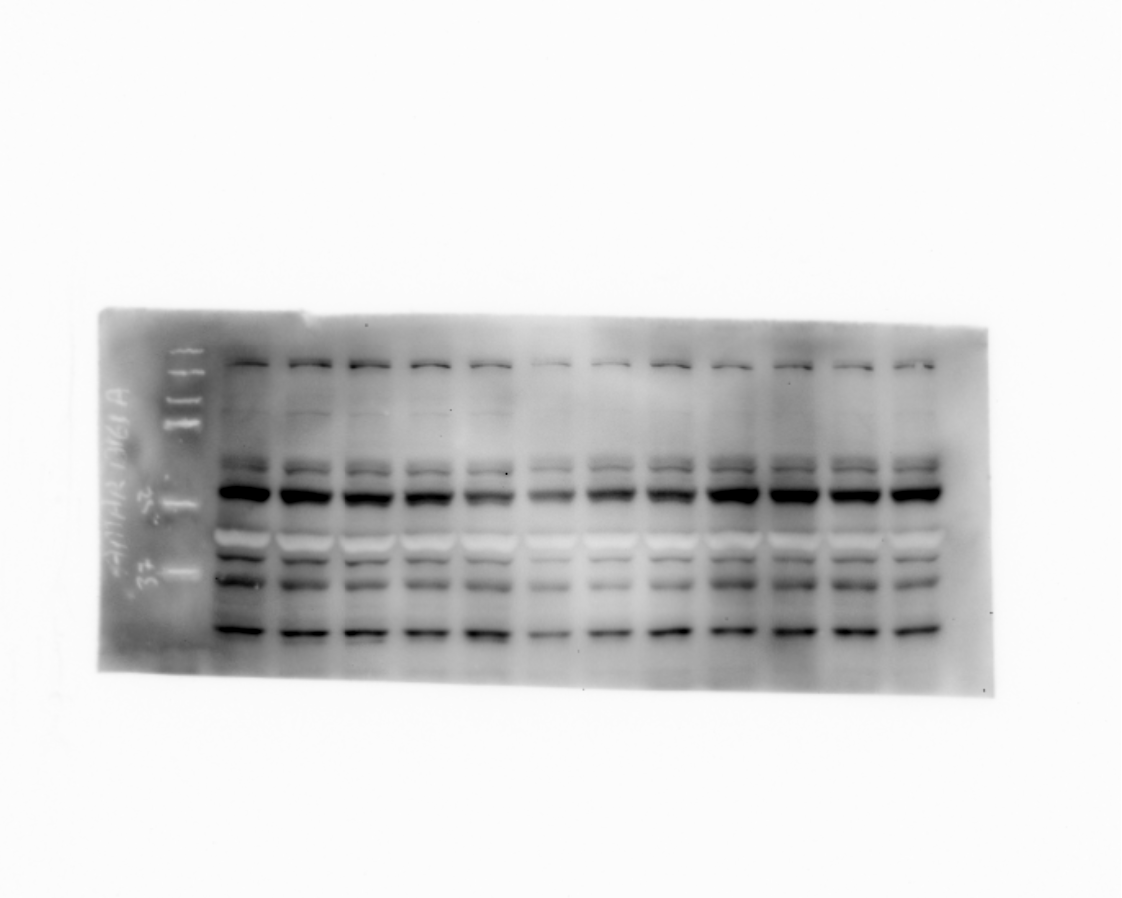

Supplement: Supplementary file 4 — Source data Fig. 3 [file 44319_2026_724_MOESM4_ESM.zip › Figure 3/3M/fig. 3M sirt2 .tif]

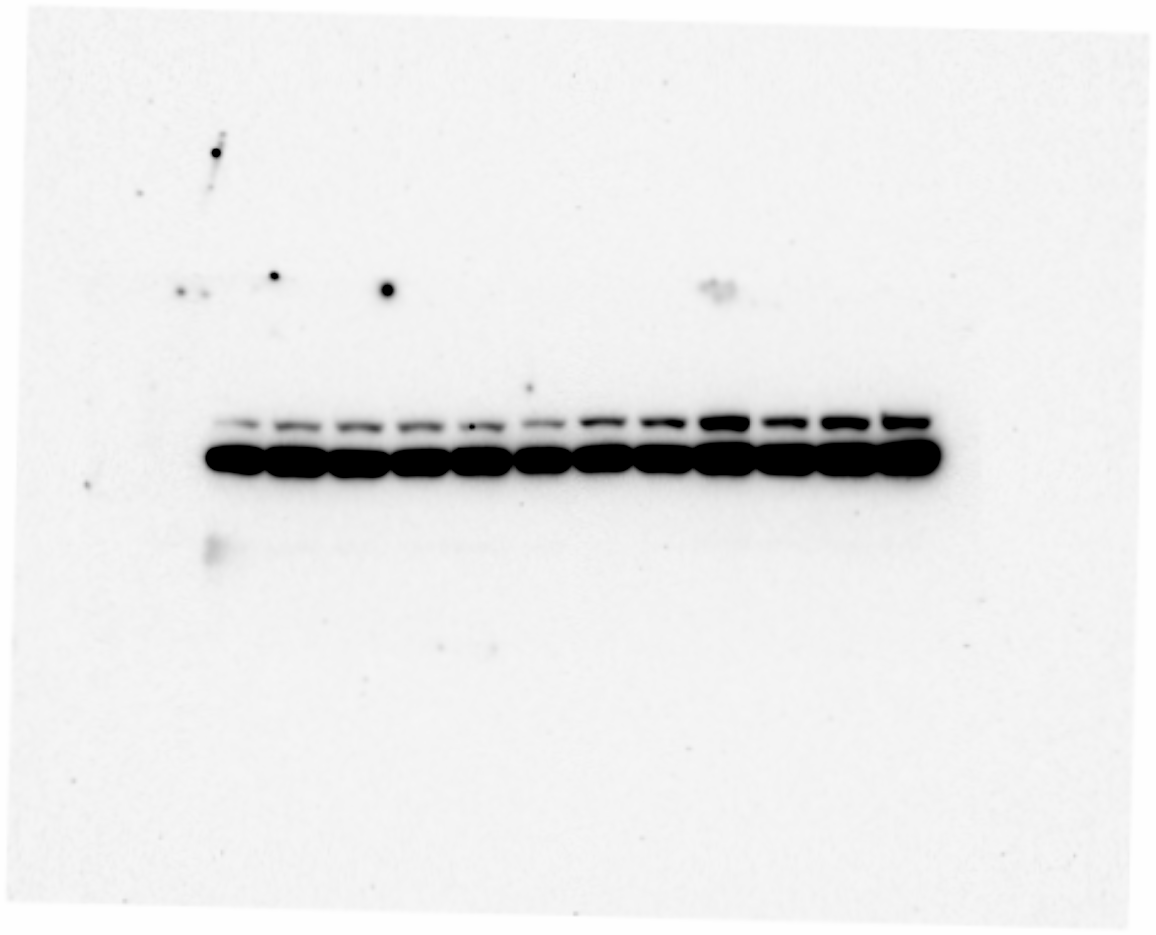

Supplement: Supplementary file 4 — Source data Fig. 3 [file 44319_2026_724_MOESM4_ESM.zip › Figure 3/3M/fig. 3M phospho ampk.tif]

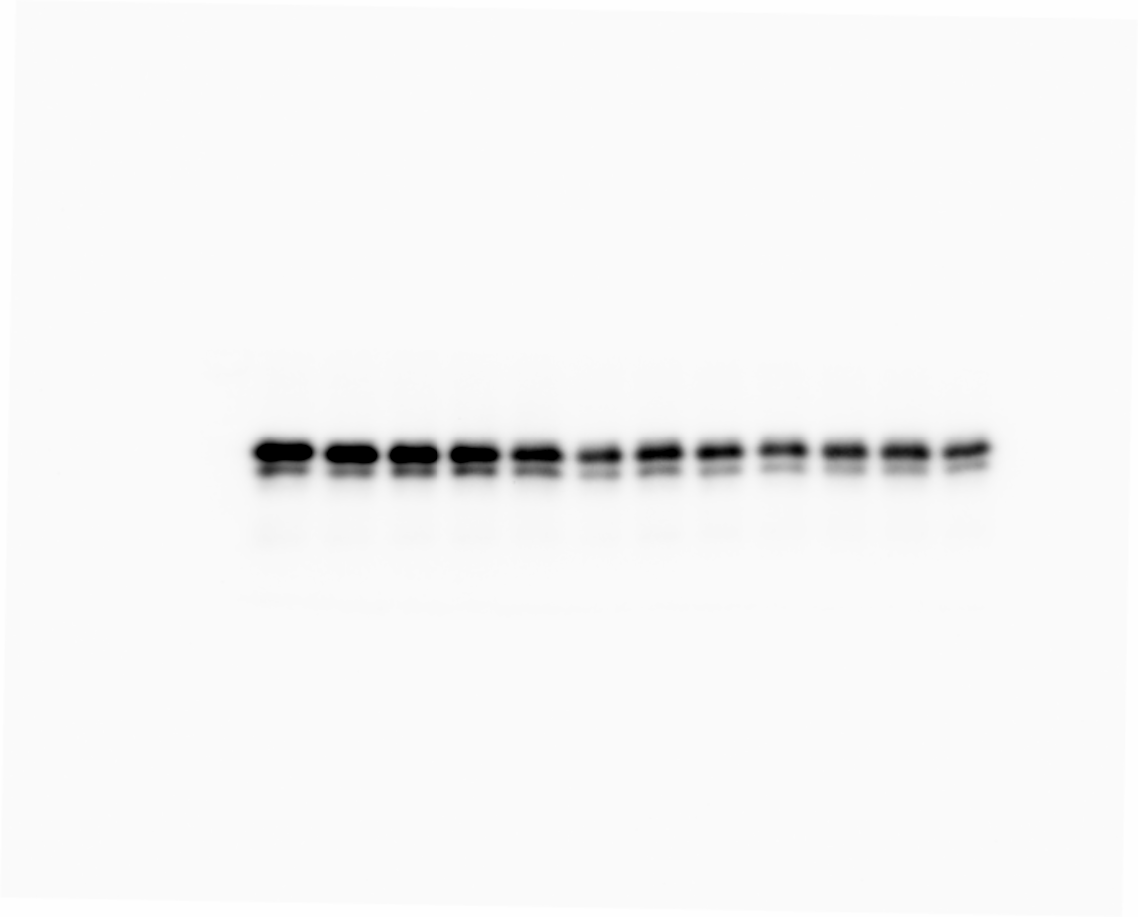

Supplement: Supplementary file 4 — Source data Fig. 3 [file 44319_2026_724_MOESM4_ESM.zip › Figure 3/3M/fig. 3M phospho 4ebp.tif]

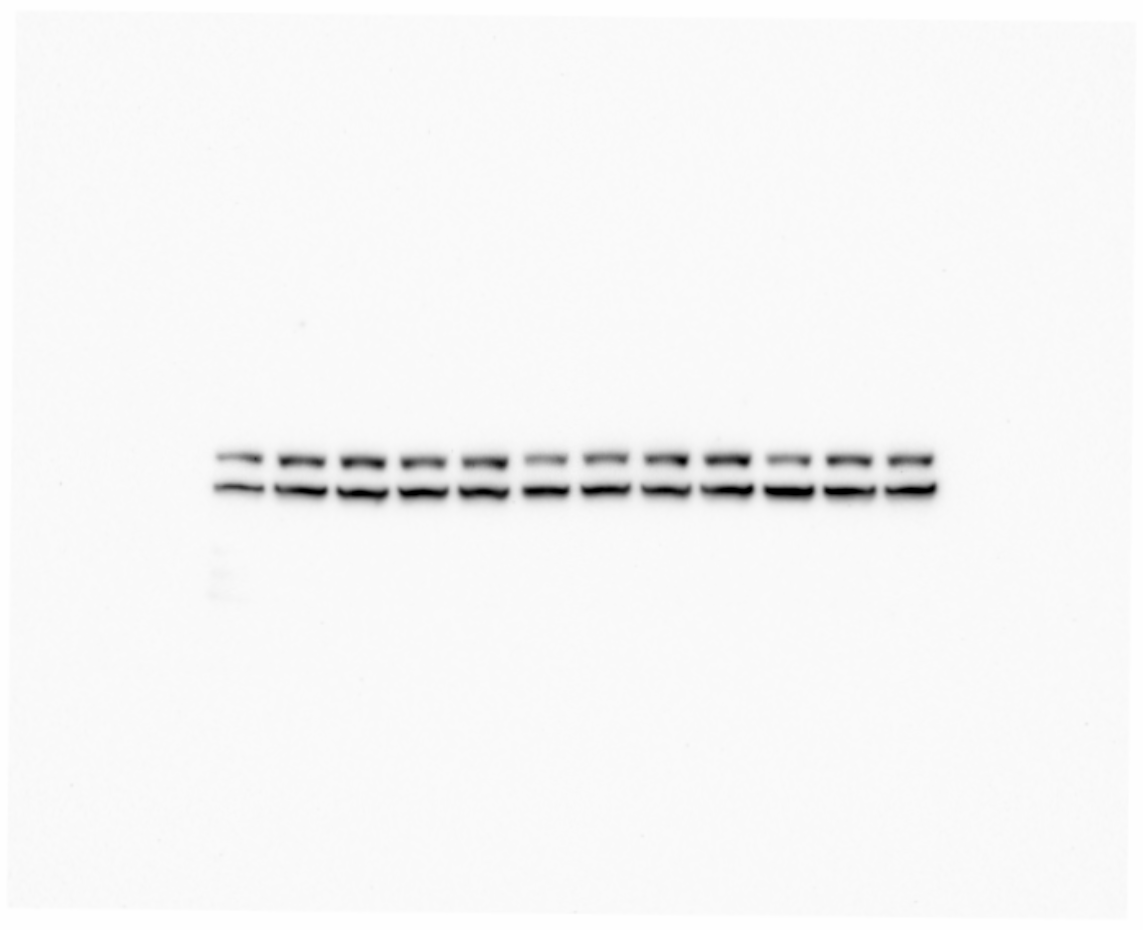

Supplement: Supplementary file 4 — Source data Fig. 3 [file 44319_2026_724_MOESM4_ESM.zip › Figure 3/3M/fig. 3M ampk.tif]

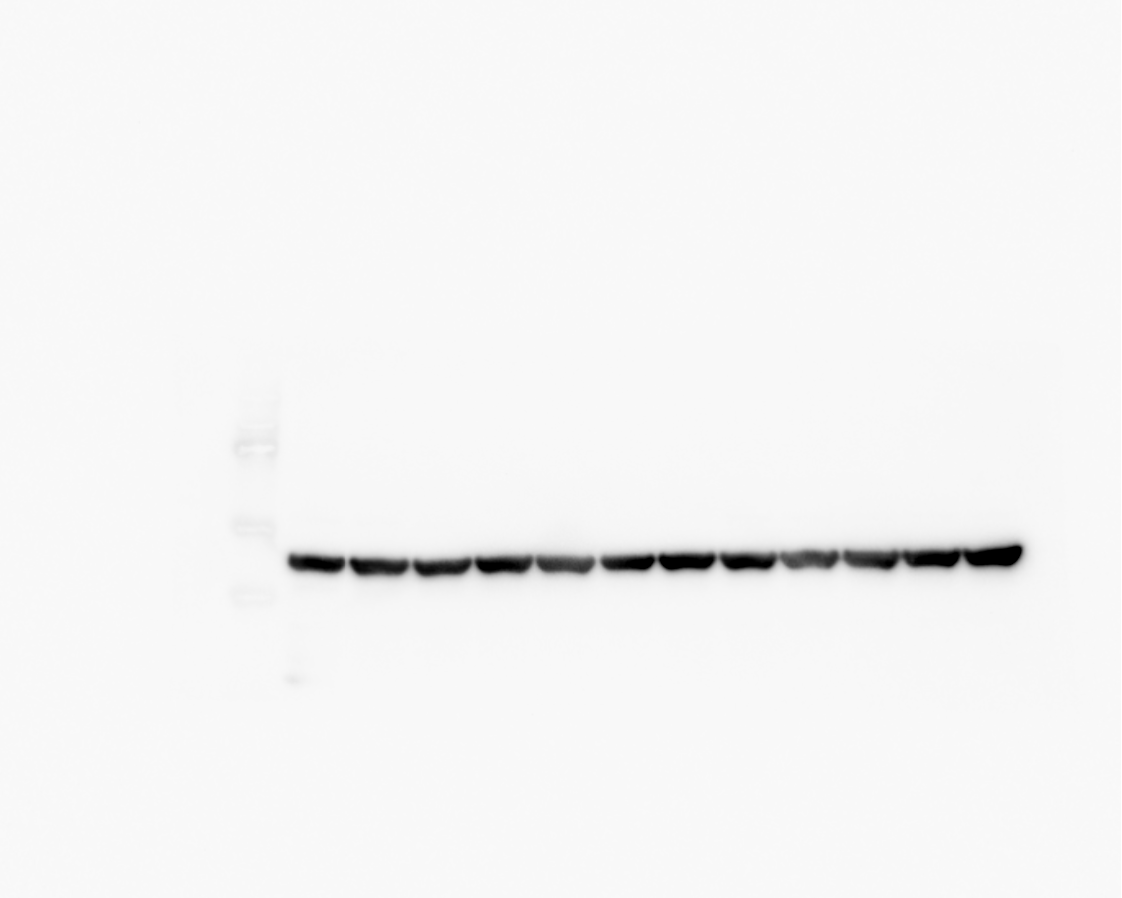

Supplement: Supplementary file 4 — Source data Fig. 3 [file 44319_2026_724_MOESM4_ESM.zip › Figure 3/3M/fig. 3M actin.tif]

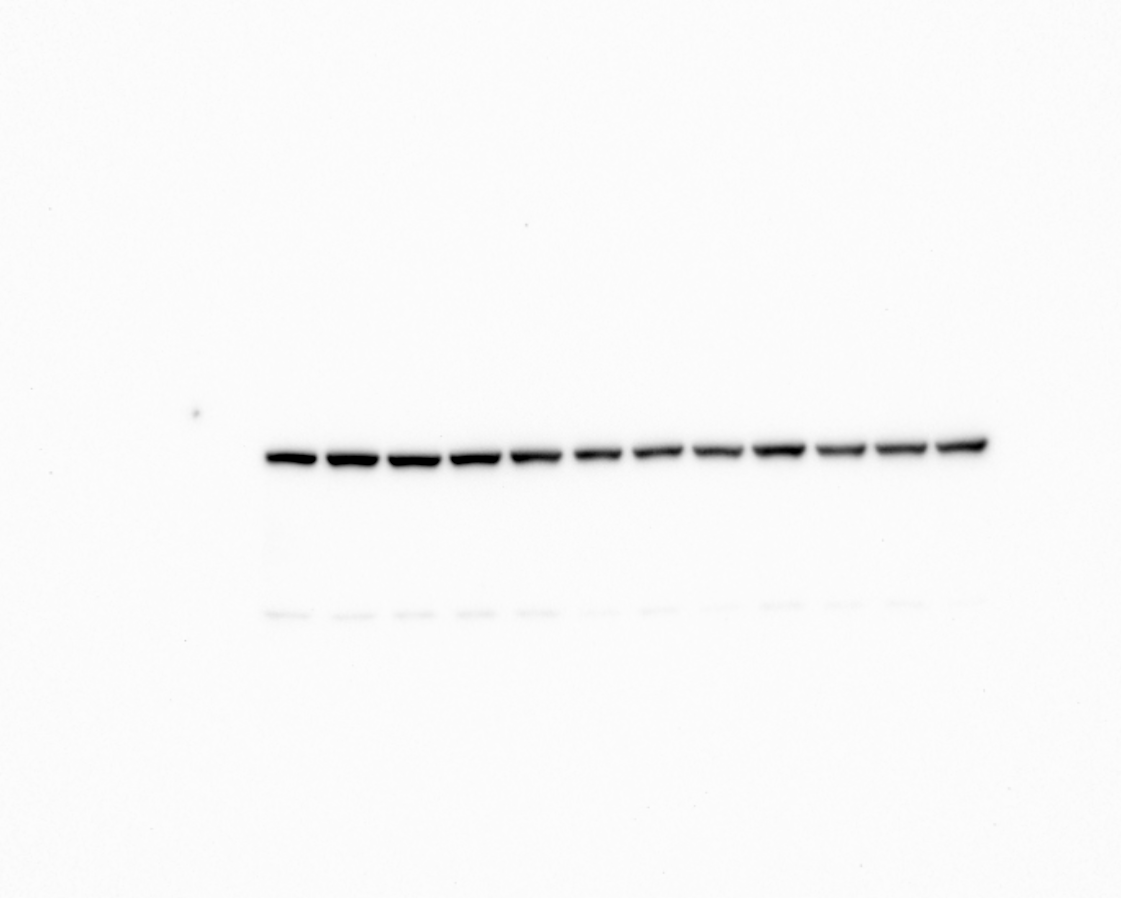

Supplement: Supplementary file 4 — Source data Fig. 3 [file 44319_2026_724_MOESM4_ESM.zip › Figure 3/3M/fig. 3M acetyl tub.tif]

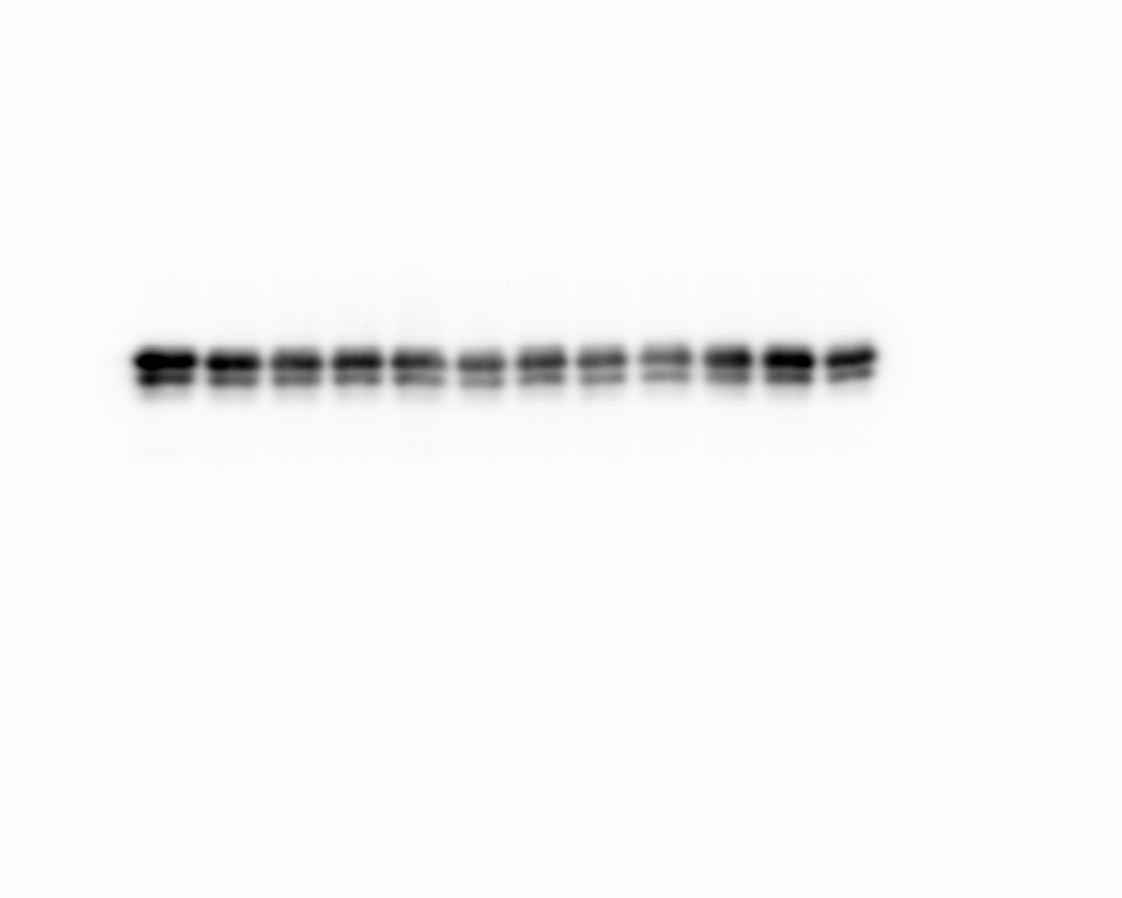

Supplement: Supplementary file 4 — Source data Fig. 3 [file 44319_2026_724_MOESM4_ESM.zip › Figure 3/3M/fig. 3M 4ebp.tif]

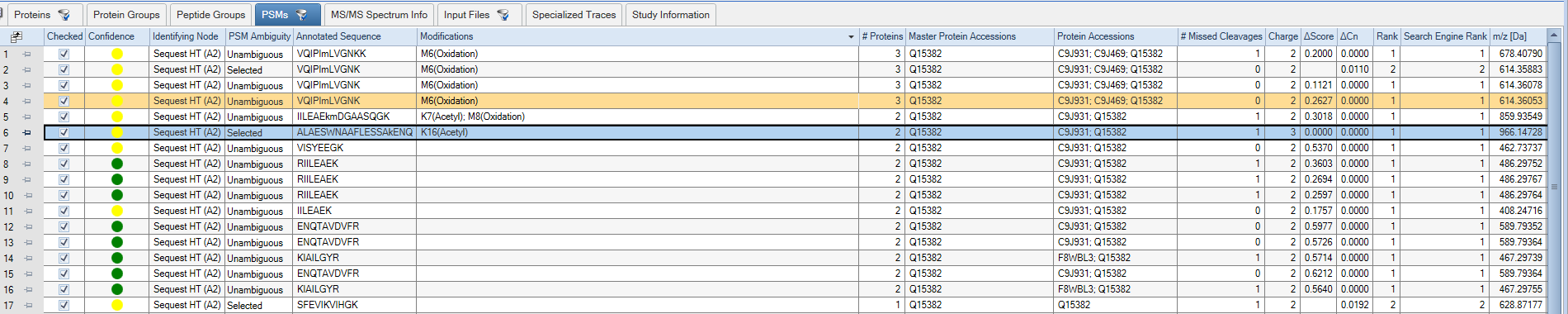

Supplement: Supplementary file 5 — Source data Fig. 4 [file 44319_2026_724_MOESM5_ESM.zip › Figure 4/4A/IG.PNG]

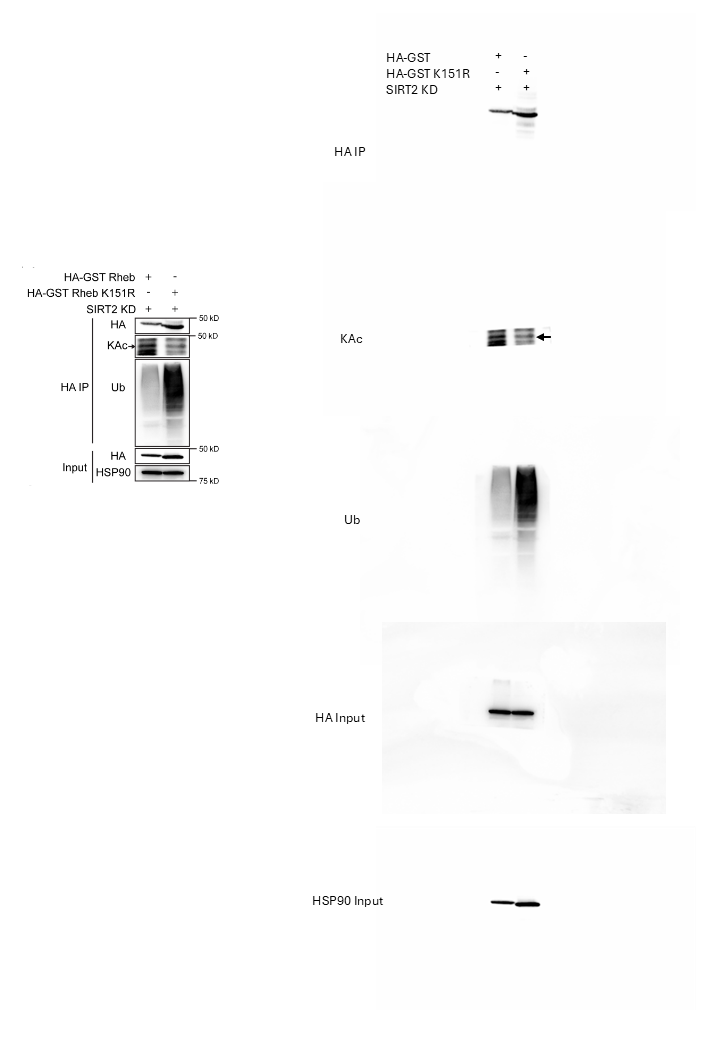

Supplement: Supplementary file 5 — Source data Fig. 4 [file 44319_2026_724_MOESM5_ESM.zip › Figure 4/4C/4C.tif]

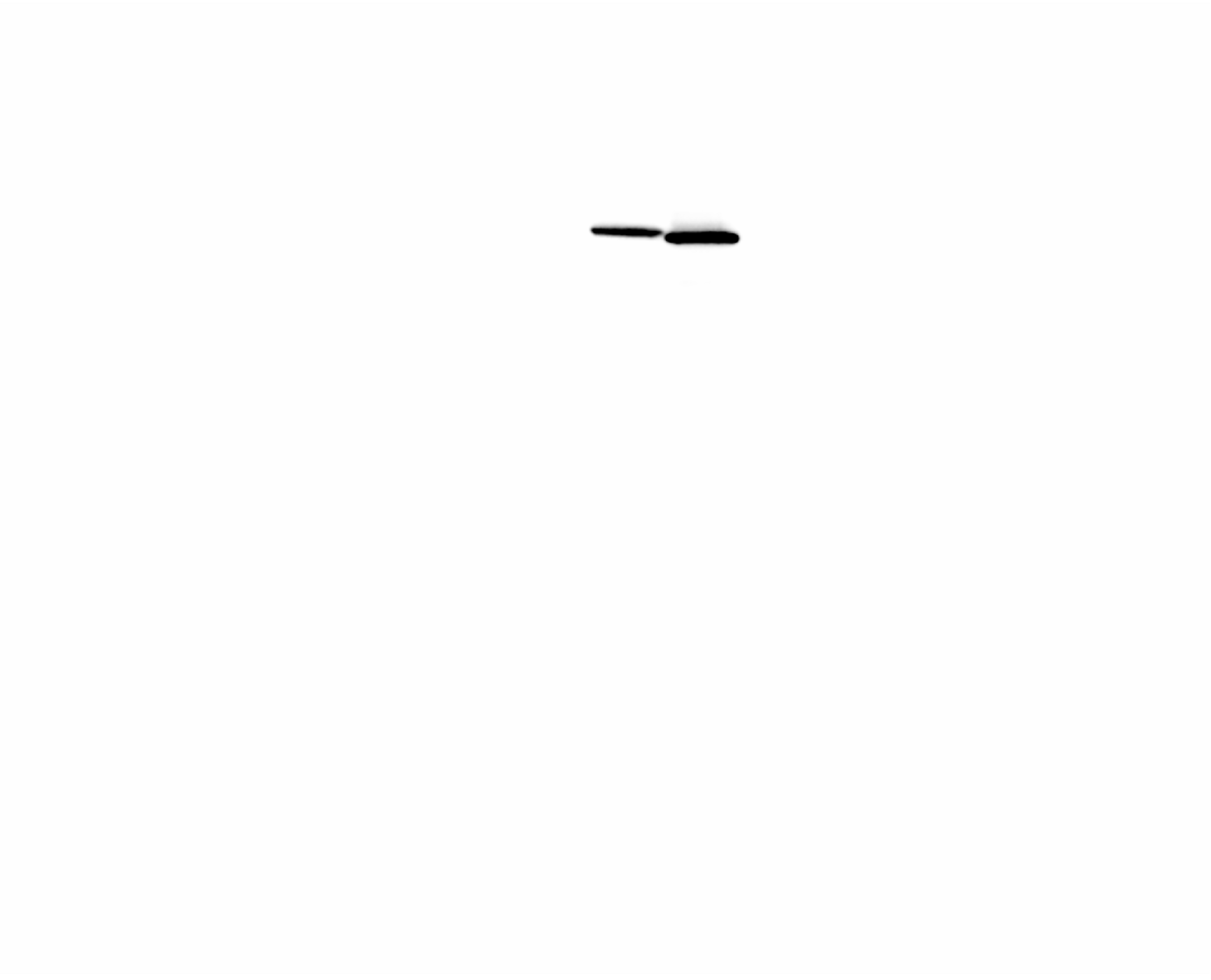

Supplement: Supplementary file 5 — Source data Fig. 4 [file 44319_2026_724_MOESM5_ESM.zip › Figure 4/4C/fig. 4C HA input.tif]
