# Supplementary material for: Sirtuin 2 inhibits global protein synthesis via Rheb-GTPase degradation
Source: EMBO Rep. 2026 Mar 11;27(11):3001–34. doi: 10.1038/s44319-026-00724-5 (PMC13261059; doi:10.1038/s44319-026-00724-5)
Supplement: Supplementary file 4 — Source data Fig. 3 [file 44319_2026_724_MOESM4_ESM.zip › Figure 3/Fig 3.pptx]

## Slide 1
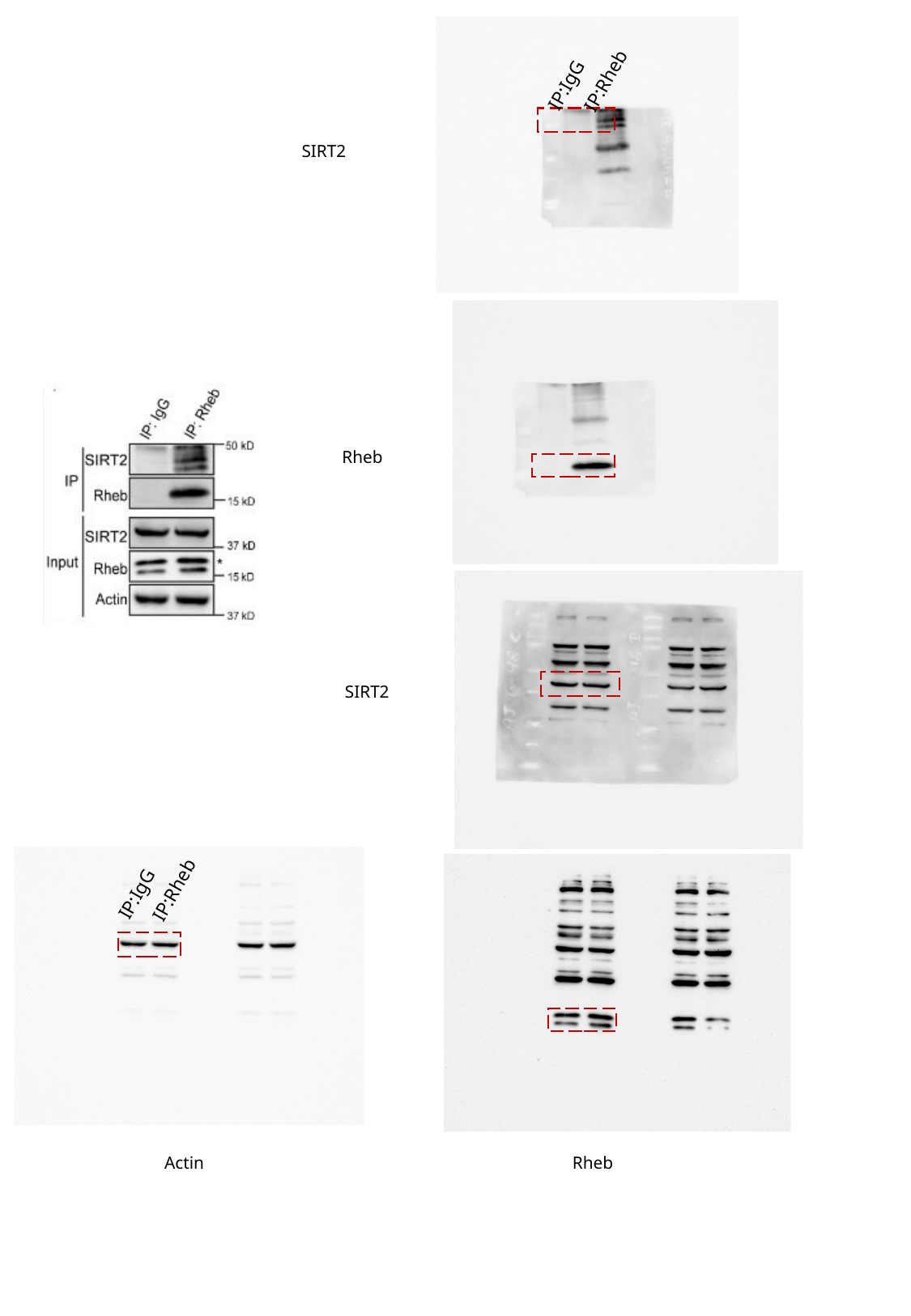

IP:Rheb
IP:IgG
SIRT2
Rheb
SIRT2
IP:Rheb
IP:IgG
Actin
Rheb

## Slide 2
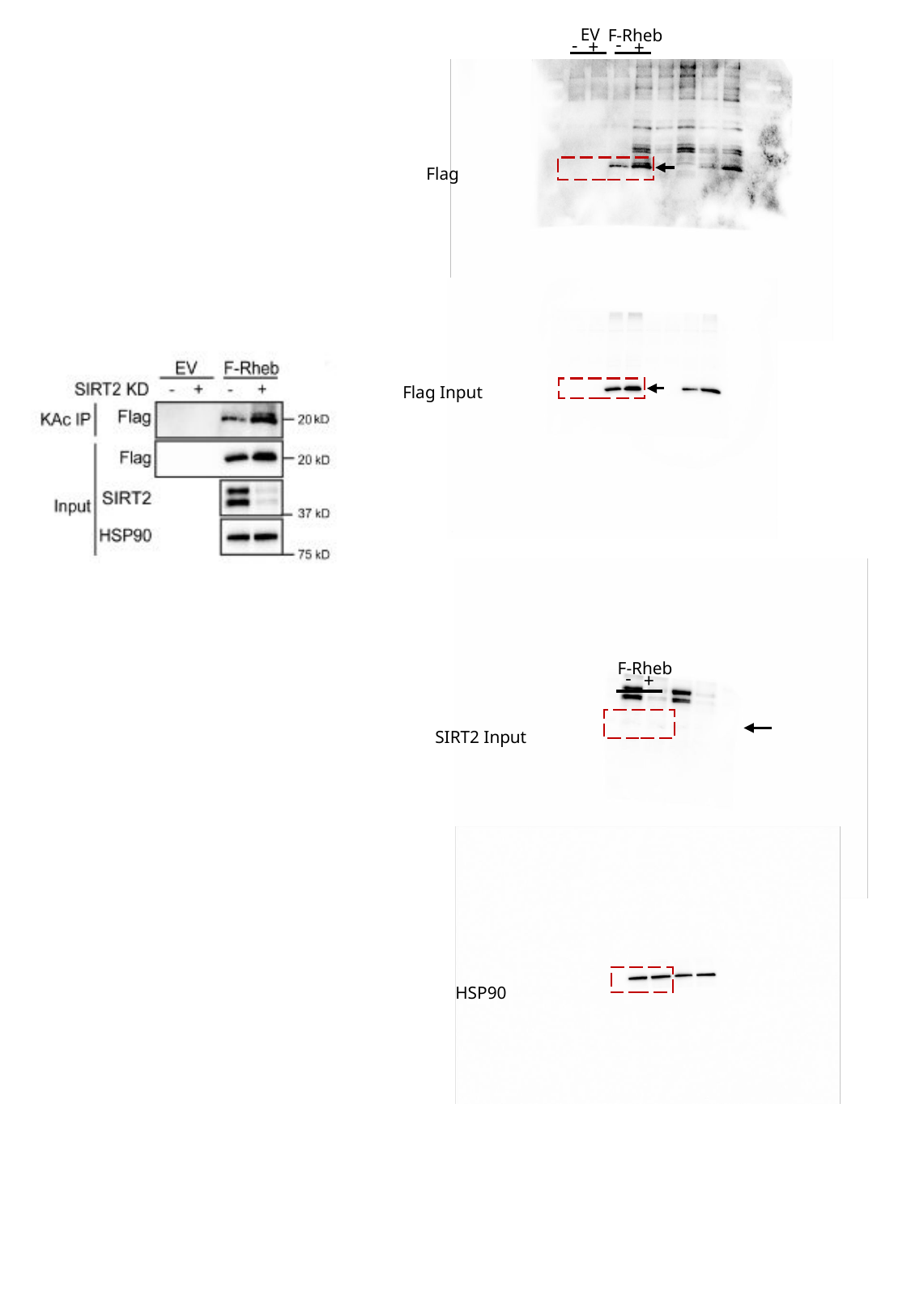

EV
F-Rheb
-
-
+
+
Flag
Flag Input
F-Rheb
-
+
SIRT2 Input
HSP90

## Slide 3
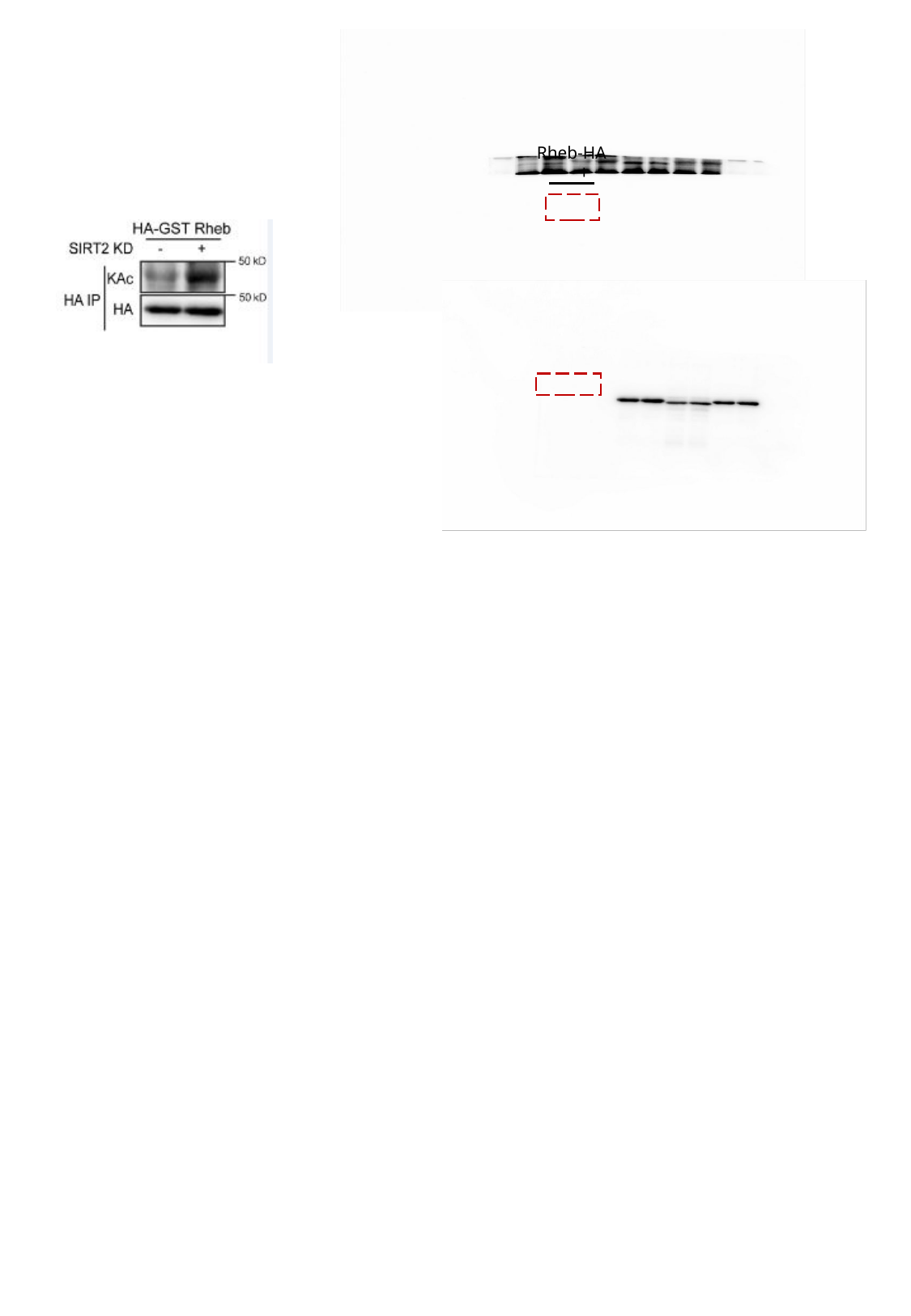

Rheb-HA
-
+

## Slide 4
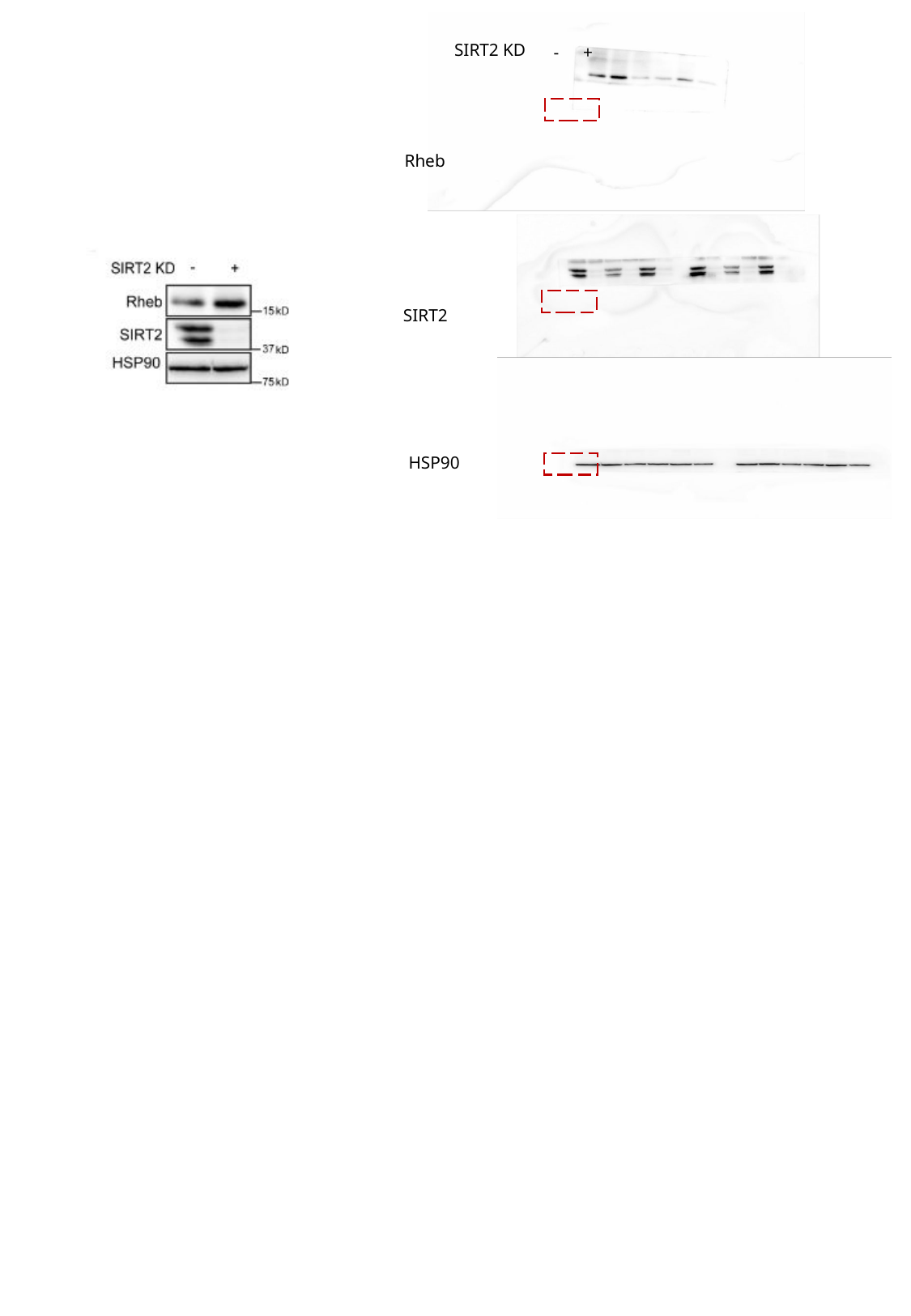

SIRT2 KD
-
+
Rheb
SIRT2
HSP90

## Slide 5
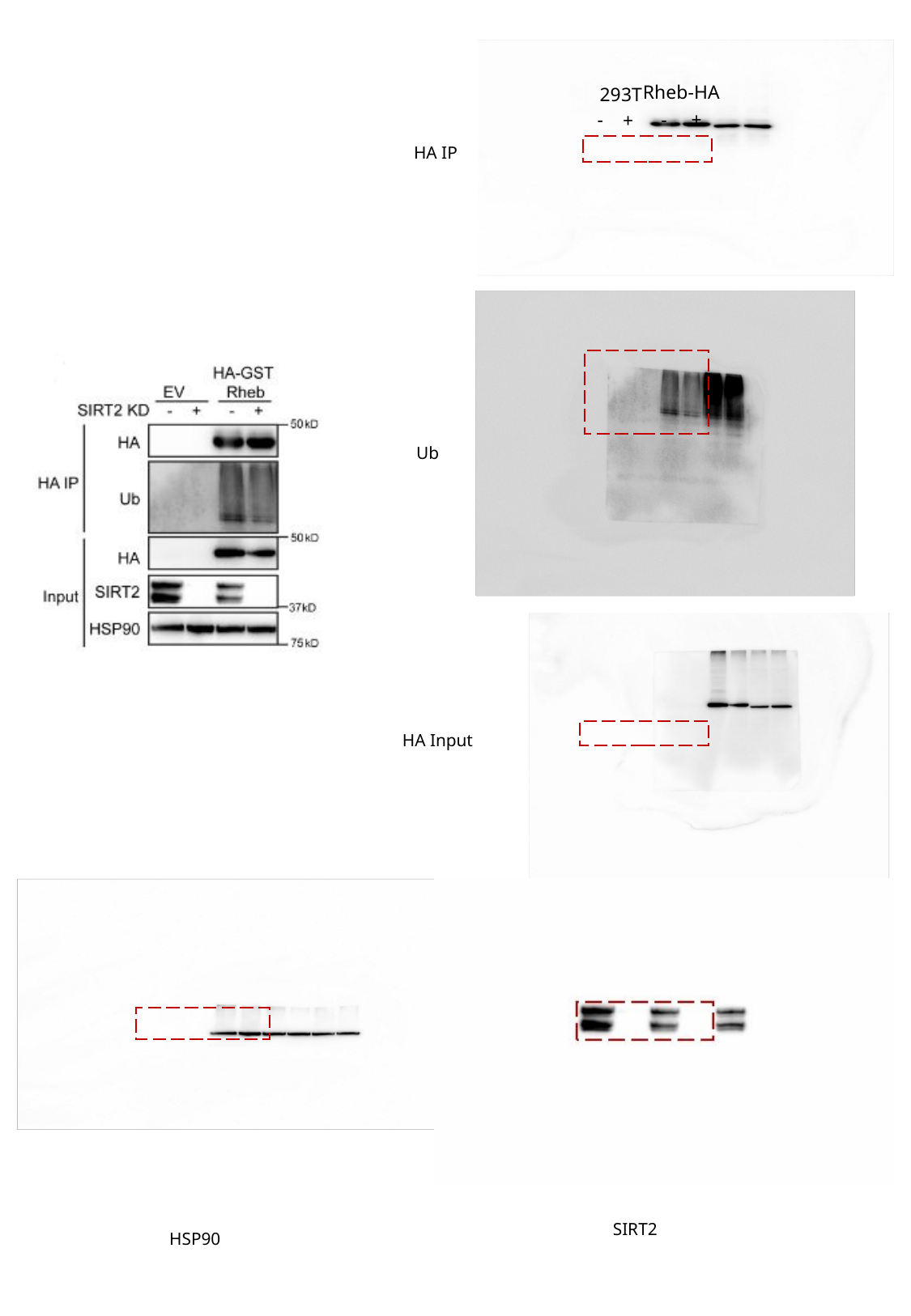

Rheb-HA
293T
-
-
+
+
HA IP
Ub
HA Input
SIRT2
HSP90

## Slide 6
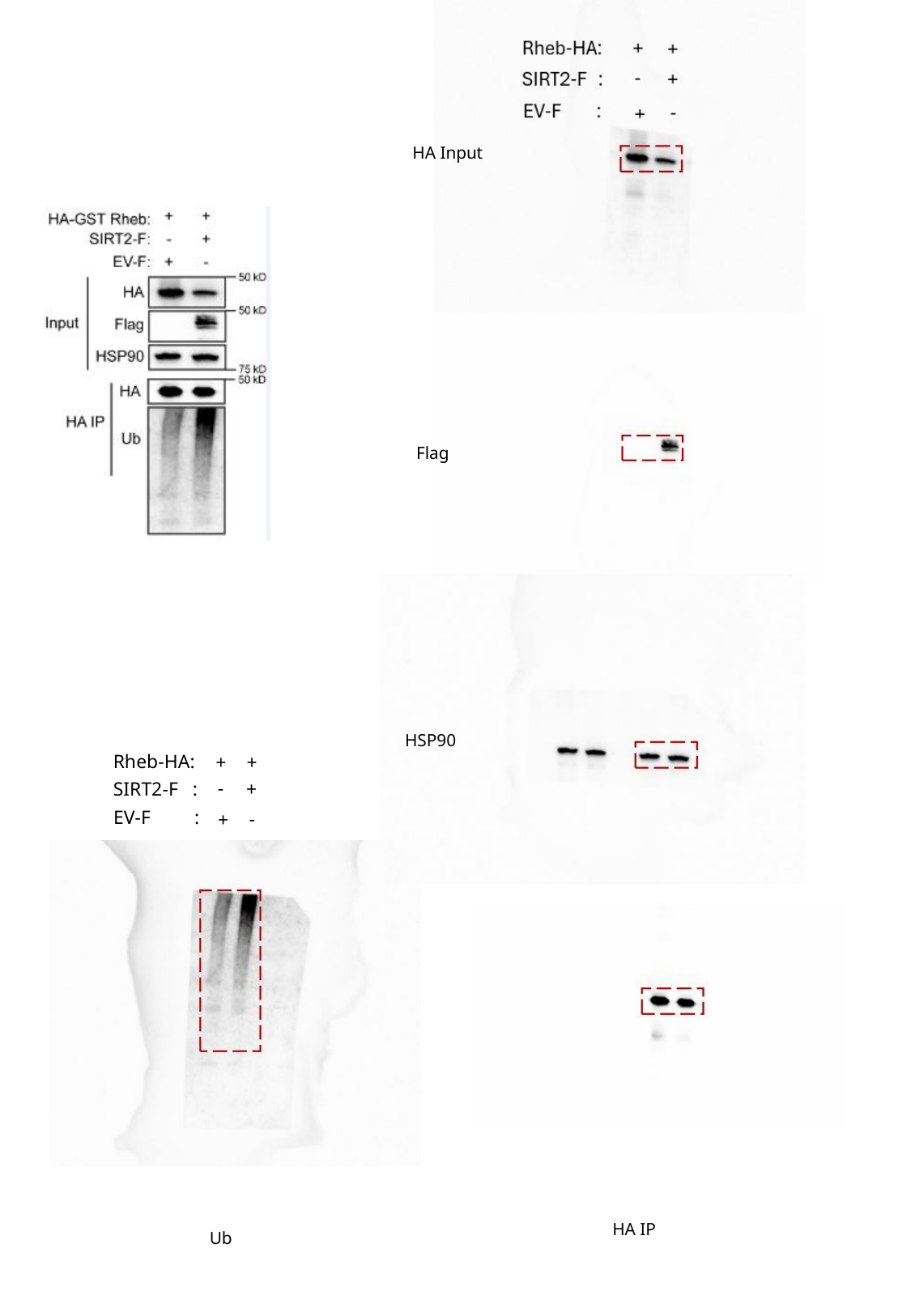

HA Input
Flag
HSP90
Rheb-HA:
+
+
-
SIRT2-F :
+
EV-F :
-
+
HA IP
Ub

## Slide 7
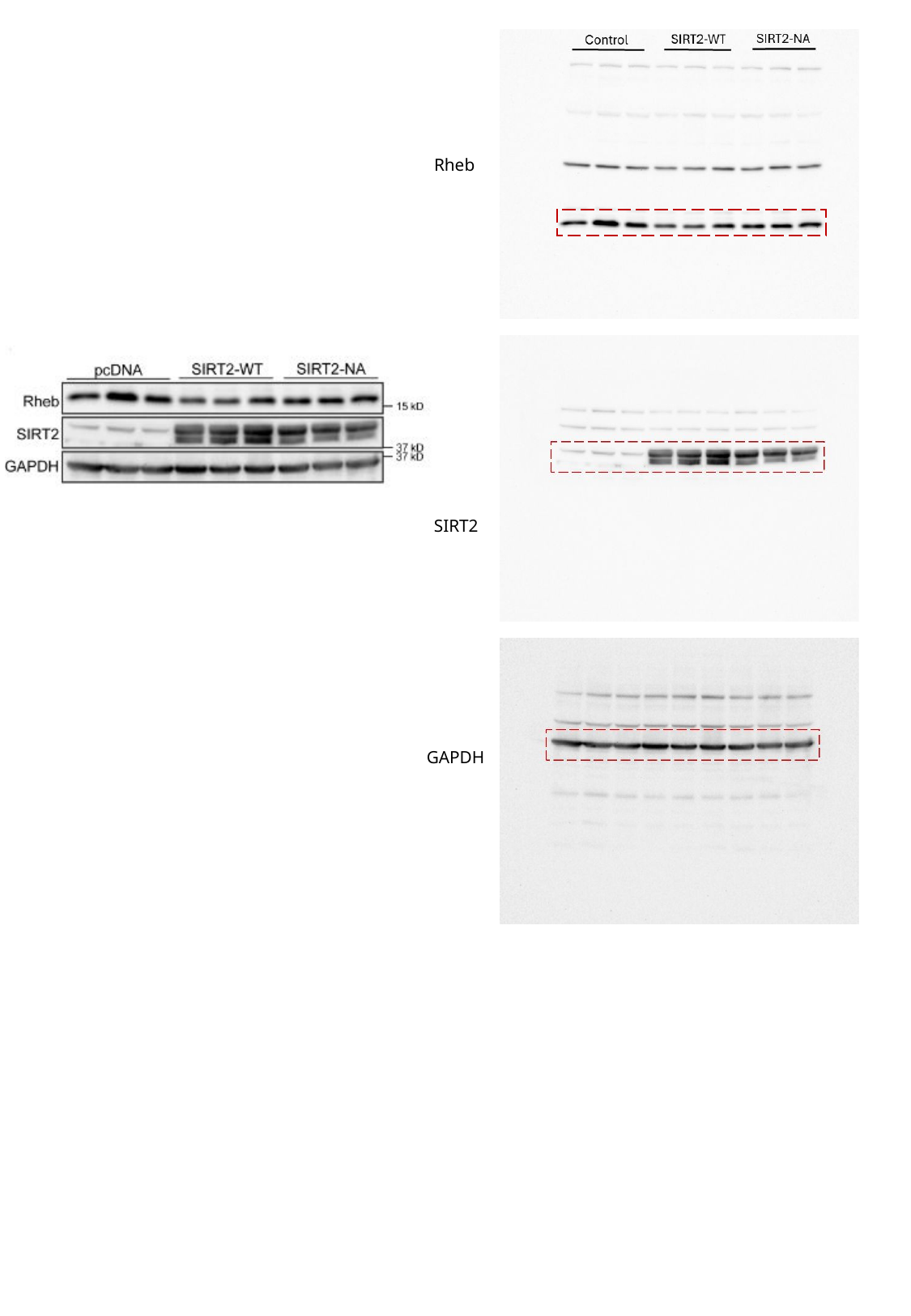

Rheb
SIRT2
GAPDH

## Slide 8
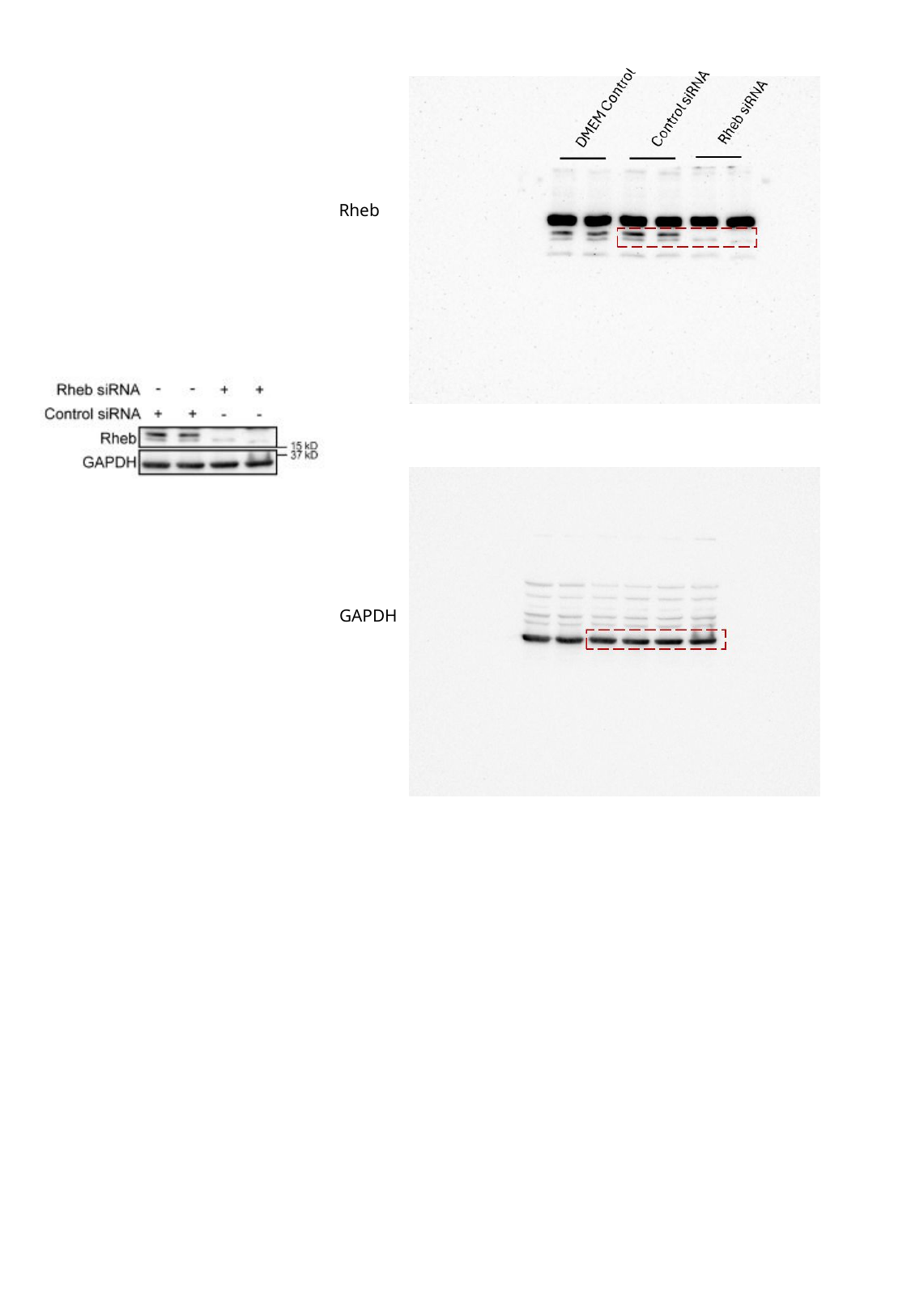

Rheb
GAPDH

## Slide 9
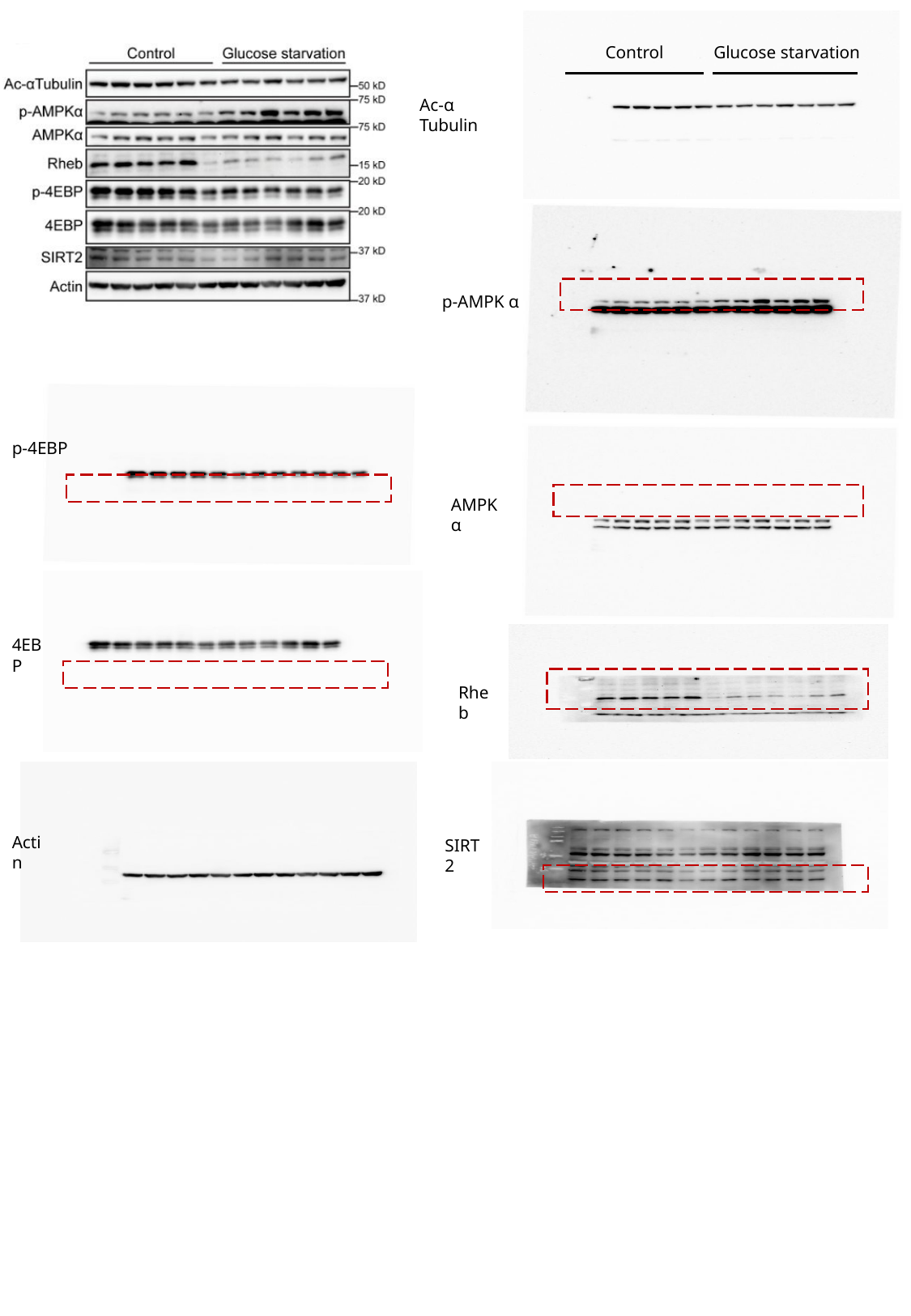

Control
Glucose starvation
Ac-α Tubulin
p-AMPK α
p-4EBP
AMPK α
4EBP
Rheb
Actin
SIRT2
