# Supplementary material for: Sirtuin 2 inhibits global protein synthesis via Rheb-GTPase degradation
Source: EMBO Rep. 2026 Mar 11;27(11):3001–34. doi: 10.1038/s44319-026-00724-5 (PMC13261059; doi:10.1038/s44319-026-00724-5)
Supplement: Supplementary file 5 — Source data Fig. 4 [file 44319_2026_724_MOESM5_ESM.zip › Figure 4/fig 4.pptx]

## Slide 1
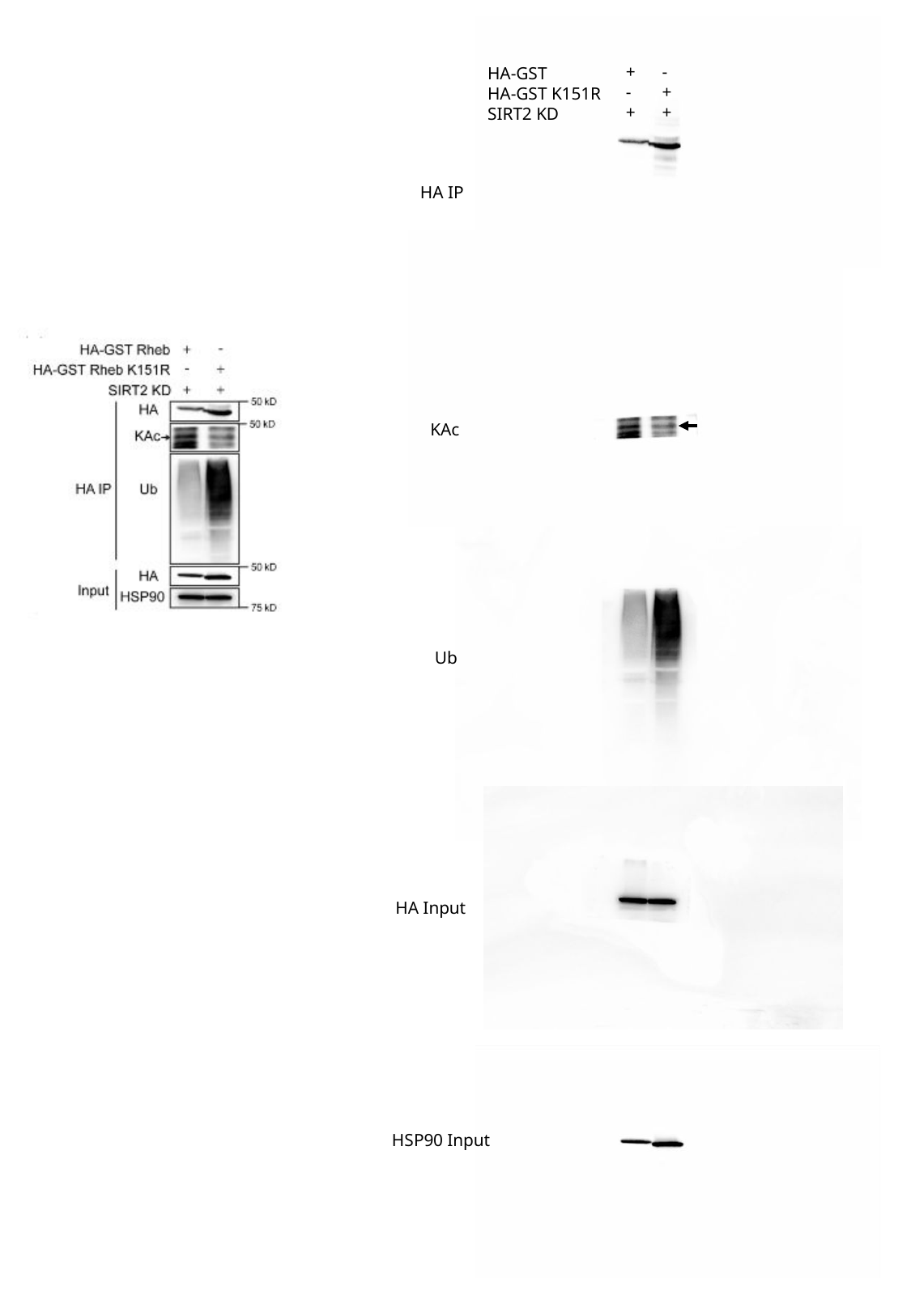

+
-
+
-
+
+
HA-GST
HA-GST K151R
SIRT2 KD
HA IP
KAc
Ub
HA Input
HSP90 Input

## Slide 2
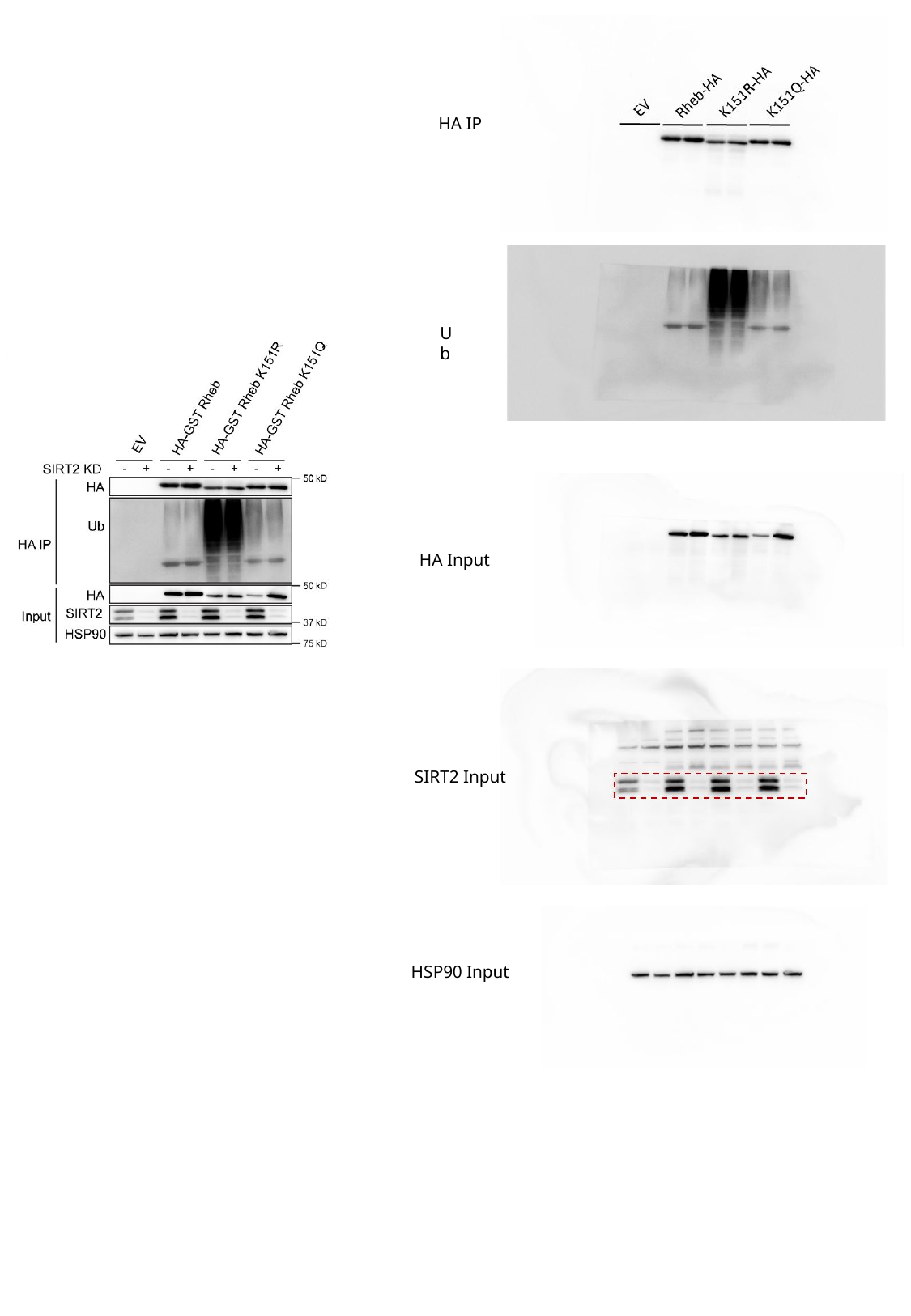

HA IP
Ub
HA Input
SIRT2 Input
HSP90 Input
