# Supplementary material for: Sirtuin 2 inhibits global protein synthesis via Rheb-GTPase degradation
Source: EMBO Rep. 2026 Mar 11;27(11):3001–34. doi: 10.1038/s44319-026-00724-5 (PMC13261059; doi:10.1038/s44319-026-00724-5)
Supplement: Supplementary file 5 — Source data Fig. 4 [file 44319_2026_724_MOESM5_ESM.zip › Figure 4/4A/STUDY INFO.docx]

====================================================================================================

Samples & Files

====================================================================================================

================================================================================

Factors

================================================================================

None

================================================================================

Quan Methods

================================================================================

None

================================================================================

Files

================================================================================

[F1] Rheb_IISc_IG

H:\Rheb_IISc_IG.raw

================================================================================

Samples

================================================================================

[S1] Rheb_IISc_IG

================================================================================

Files to Samples

================================================================================

[F1] Rheb_IISc_IG

[S1] Rheb_IISc_IG

====================================================================================================

Analysis Settings

====================================================================================================

================================================================================

Consensus Step : Workflow

================================================================================

Result name: Rheb_IISc_IG

Result file: F:\2023\Internal\AMARJEET_SHARMA\New Study_16012024\Rheb_IISc_IG.pdResult

Description: Result filtered for high confident peptides.

Workflow based on template: CWF_Basic

Creation date: 1/16/2024 12:09:17 PM

Created with Discoverer version: 2.5.0.400

------------------------------------------------------------------

The workflow tree:

------------------------------------------------------------------

|-(0) MSF Files

|-(1) PSM Grouper

|-(2) Peptide Validator

|-(3) Peptide and Protein Filter

|-(4) Protein Scorer

|-(5) Protein Grouping

|-(6) Protein FDR Validator

Post-processing nodes:

--------------------------------

|-(7) Display Settings

------------------------------------------------------------------

Processing node 0: MSF Files

------------------------------------------------------------------

1. Storage Settings:

- Spectra to Store: Identified or Quantified

- Feature Traces to Store: All

2. Merging of Identified Peptide and Proteins:

- Merge Mode: Globally by Search Engine Type

3. FASTA Title Line Display:

- Reported FASTA Title Lines: Best match

- Title Line Rule: standard

4. PSM Filters:

- Maximum Delta Cn: 0.05

- Maximum Rank: 0

- Maximum Delta Mass: 0 ppm

Hidden Parameters:

- MSF File(s): D:\2023\Internal\AMARJEET_SHARMA\New Study_16012024\Rheb_IISc_IG.msf

------------------------------------------------------------------

Processing node 1: PSM Grouper

------------------------------------------------------------------

1. Peptide Group Modifications:

- Site Probability Threshold: 75

------------------------------------------------------------------

Processing node 2: Peptide Validator

------------------------------------------------------------------

1. General Validation Settings:

- Validation Mode: Automatic (Control peptide level error rate if possible)

- Target FDR (Strict) for PSMs: 0.01

- Target FDR (Relaxed) for PSMs: 0.05

- Target FDR (Strict) for Peptides: 0.01

- Target FDR (Relaxed) for Peptides: 0.05

2. Specific Validation Settings:

- Validation Based on: q-Value

- Target/Decoy Selection for PSM Level FDR Calculation Based on Score: Automatic

- Reset Confidences for Nodes without Decoy Search (Fixed score thresholds): False

------------------------------------------------------------------

Processing node 3: Peptide and Protein Filter

------------------------------------------------------------------

1. Peptide Filters:

- Peptide Confidence At Least: Medium

- Keep Lower Confident PSMs: False

- Minimum Peptide Length: 6

- Remove Peptides Without Protein Reference: False

2. Protein Filters:

- Minimum Number of Peptide Sequences: 1

- Count Only Rank 1 Peptides: False

- Count Peptides Only for Top Scored Protein: False

------------------------------------------------------------------

Processing node 4: Protein Scorer

------------------------------------------------------------------

No parameters

------------------------------------------------------------------

Processing node 5: Protein Grouping

------------------------------------------------------------------

1. Protein Grouping:

- Apply strict parsimony principle: True

------------------------------------------------------------------

Processing node 6: Protein FDR Validator

------------------------------------------------------------------

1. Confidence Thresholds:

- Target FDR (Strict): 0.01

- Target FDR (Relaxed): 0.05

------------------------------------------------------------------

Processing node 7: Display Settings

------------------------------------------------------------------

1. General:

- Filter Set:

### Filter Set MasterProteinFilter.filterset contains the following filters:

### Row Filter for TargetProtein:

### Master is equal to Master

###

'magellan filter set' 1 'MasterProteinFilter.filterset' FiltersetProperties 1 'LastFileName' 'C:\Users\frank.berg\Desktop\MasterProteinFilter.filterset' Filter 'TargetProtein' 1 NARY_AND 1 = FilterConditionProperties 1 'NamedComparableFilterCondition/DisplayPropertyHint' 'Master' property 'Thermo.PD.EntityDataFramework.MasterProteinAssessment, Thermo.Magellan.EntityDataFramework' 'IsMasterProtein' constant 'Thermo.PD.EntityDataFramework.MasterProteinAssessment, Thermo.Magellan.EntityDataFramework' 'IsMasterProtein'

------------------------------------------------------------------

Workflow messages:

------------------------------------------------------------------

01/16/2024 12:13 PM Job Execution: Processing D:\2023\Internal\AMARJEET_SHARMA\New Study_16012024\Rheb_IISc_IG.pdResult

01/16/2024 12:13 PM (0) MSF Files: D:\2023\Internal\AMARJEET_SHARMA\New Study_16012024\Rheb_IISc_IG.msf

01/16/2024 12:13 PM (0) MSF Files: All 1 files are ready for processing.

01/16/2024 12:13 PM (0) MSF Files: Start transferring results of 1 msf files...

01/16/2024 12:13 PM (0) MSF Files: Transferred 29387 Proteins to result file in 3.2 s.

01/16/2024 12:13 PM (0) MSF Files: Transferred 0 Decoy Proteins to result file in 136.7 ms.

01/16/2024 12:13 PM (0) MSF Files: Added 17566 Mass Spectra to result file.

01/16/2024 12:13 PM (0) MSF Files: Added 2 traces to result file.

01/16/2024 12:13 PM (0) MSF Files: Added 1 Input Files to result file.

01/16/2024 12:13 PM (0) MSF Files: Added 26423 PSMs to result file.

01/16/2024 12:13 PM (0) MSF Files: Added 19316 MS/MS Spectrum Info to result file.

01/16/2024 12:13 PM (0) MSF Files: Added 19316 Precursors to result file.

01/16/2024 12:13 PM (0) MSF Files: Added 1 Mass Recalibrations to result file.

01/16/2024 12:13 PM (0) MSF Files: Added 67 Correction Landmarks to result file.

01/16/2024 12:13 PM (0) MSF Files: Added 2 Specialized Traces to result file.

01/16/2024 12:13 PM (0) MSF Files: Copying data took 10.2 s.

01/16/2024 12:13 PM (0) MSF Files: Finding unique sequences took 914.3 ms.

01/16/2024 12:13 PM (0) MSF Files: Filtering proteins took 246.1 ms.

01/16/2024 12:13 PM (0) MSF Files: -- Total execution of MSF Files (0) took 16.5 s --

01/16/2024 12:13 PM (1) PSM Grouper: Grouping 'PSMs'

01/16/2024 12:13 PM (1) PSM Grouper: Found 24650 Peptide Groups.

01/16/2024 12:13 PM (1) PSM Grouper: Found 0 Decoy Peptide Groups.

01/16/2024 12:13 PM (1) PSM Grouper: -- Total execution of PSM Grouper (1) took 13.7 s --

01/16/2024 12:13 PM (2) Peptide Validator: Start PSM and Peptide validation in 'Automatic (Control peptide level error rate if possible)' mode...

01/16/2024 12:13 PM (2) Peptide Validator: No decoy search was performed for the following search nodes: - Sequest HT (A2) in workflow Workflow

. FDR + fixed threshold validation is used instead.

01/16/2024 12:13 PM (2) Peptide Validator: Starting revalidation PSMs by FDR using Concatenated mode.

01/16/2024 12:13 PM (2) Peptide Validator: No revalidation of PSMs necessary.

01/16/2024 12:13 PM (2) Peptide Validator: Set peptide group confidences to the best PSM confidence.

01/16/2024 12:13 PM (2) Peptide Validator: Transfering confidences to Peptide Groups

01/16/2024 12:13 PM (2) Peptide Validator: Transfering confidences to Decoy Peptide Groups

01/16/2024 12:13 PM (2) Peptide Validator: Calculating Mascot thresholds.

01/16/2024 12:13 PM (2) Peptide Validator: -- Total execution of Peptide Validator (2) took 731.6 ms --

01/16/2024 12:13 PM (3) Peptide and Protein Filter: Filter Peptide Groups

01/16/2024 12:13 PM (3) Peptide and Protein Filter: Filter 19902/24650 Peptide Groups (4748 excluded) and 21353/26423 PSMs (5070 excluded) took 658.9 ms

01/16/2024 12:13 PM (3) Peptide and Protein Filter: Filtering Proteins

01/16/2024 12:14 PM (3) Peptide and Protein Filter: Filter 25457/29387 Proteins (3930 excluded) took 453.6 ms

01/16/2024 12:14 PM (3) Peptide and Protein Filter: Filter Decoy Peptide Groups

01/16/2024 12:14 PM (3) Peptide and Protein Filter: Filter 0/0 Decoy Peptide Groups (0 excluded) and 0/0 Decoy PSMs (0 excluded) took 11.1 ms

01/16/2024 12:14 PM (3) Peptide and Protein Filter: Skip filtering of Decoy Proteins as none will be excluded!

01/16/2024 12:14 PM (3) Peptide and Protein Filter: Updating counts took 5.1 s.

01/16/2024 12:14 PM (3) Peptide and Protein Filter: -- Total execution of Peptide and Protein Filter (3) took 6.9 s --

01/16/2024 12:14 PM (4) Protein Scorer: Calculating Coverage and Counts

01/16/2024 12:14 PM (4) Protein Scorer: Calculated counts and coverages in 2.1 s.

01/16/2024 12:14 PM (4) Protein Scorer: Scoring target proteins

01/16/2024 12:14 PM (4) Protein Scorer: Scored 25457 proteins in 3.4 s.

01/16/2024 12:14 PM (4) Protein Scorer: Scoring decoy proteins

01/16/2024 12:14 PM (4) Protein Scorer: Scored 0 decoy proteins in 30.7 ms.

01/16/2024 12:14 PM (4) Protein Scorer: -- Total execution of Protein Scorer (4) took 5.6 s --

01/16/2024 12:14 PM (5) Protein Grouping: Retrieving 10781 protein groups took 10.6 s.

01/16/2024 12:14 PM (5) Protein Grouping: Storing, updating and connecting protein groups, PSMs and peptides took 4.6 s.

01/16/2024 12:14 PM (5) Protein Grouping: Check 10781 protein groups.

01/16/2024 12:14 PM (5) Protein Grouping: Excluded 3097 protein groups with no valid PSM remaining.

01/16/2024 12:14 PM (5) Protein Grouping: Applying strict parsimony took 6.5 s.

01/16/2024 12:14 PM (5) Protein Grouping: Found 7509 protein groups.

01/16/2024 12:14 PM (5) Protein Grouping: Storing, updating and connecting protein groups, PSMs and peptides took 320.1 ms.

01/16/2024 12:14 PM (5) Protein Grouping: Found 0 decoy protein groups.

01/16/2024 12:14 PM (5) Protein Grouping: -- Total execution of Protein Grouping (5) took 25.2 s --

01/16/2024 12:14 PM (6) Protein FDR Validator: Apply confidences based on protein scores of 25457 proteins.

01/16/2024 12:14 PM (6) Protein FDR Validator: Cannot validate proteins because there are no decoy proteins in the file.

01/16/2024 12:14 PM (6) Protein FDR Validator: -- Total execution of Protein FDR Validator (6) took 26.4 ms --

01/16/2024 12:14 PM (7) Display Settings: Applying display filter and layout

01/16/2024 12:14 PM (7) Display Settings: -- Total execution of Display Settings (7) took 22.4 ms --

01/16/2024 12:14 PM Job Execution: Finalizing file took 5.3 s.

01/16/2024 12:14 PM Job Execution: Finished D:\2023\Internal\AMARJEET_SHARMA\New Study_16012024\Rheb_IISc_IG.pdResult

01/16/2024 12:14 PM Job Execution: ----- Total Job execution took: 1 min 14 s. -----

================================================================================

Processing Step A: Workflow

================================================================================

Result name: Rheb_IISc_IG

Result file: D:\2023\Internal\AMARJEET_SHARMA\New Study_16012024\Rheb_IISc_IG.msf

Description: Basic processing workflow with score threshold validation to be used for searches of low complexity samples or employing a small FASTA database. Specify the FASTA database and any additional modifications.

Workflow based on template: PWF_Tribrid_Basic_SequestHT

Creation date: 1/16/2024 12:09:15 PM

Created with Discoverer version: 2.5.0.400

------------------------------------------------------------------

The workflow tree:

------------------------------------------------------------------

|-(0) Spectrum Files RC

|-(1) Spectrum Selector

|-(2) Sequest HT

|-(3) Fixed Value PSM Validator

------------------------------------------------------------------

Processing node 0: Spectrum Files RC

------------------------------------------------------------------

1. Search Settings:

- File Name(s): H:\Rheb_IISc_IG.raw

- Protein Database: Human_uniprot-proteome_UP000005640.fasta

- Enzyme Name: Trypsin (Full)

- Precursor Mass Tolerance: 20 ppm

- Fragment Mass Tolerance: 0.5 Da

- 1. Static Modification: Carbamidomethyl / +57.021 Da (C)

2. Regression Settings:

- Regression Model: Non-linear Regression

- Parameter Tuning: Coarse

------------------------------------------------------------------

Processing node 1: Spectrum Selector

------------------------------------------------------------------

1. General Settings:

- Precursor Selection: Use MS1 Precursor

- Use Isotope Pattern in Precursor Reevaluation: True

- Provide Profile Spectra: Automatic

2. Spectrum Properties Filter:

- Lower RT Limit: 0

- Upper RT Limit: 0

- First Scan: 0

- Last Scan: 0

- Lowest Charge State: 0

- Highest Charge State: 0

- Min. Precursor Mass: 350 Da

- Max. Precursor Mass: 5000 Da

- Total Intensity Threshold: 0

- Minimum Peak Count: 1

3. Scan Event Filters:

- MS Order: Is Not MS1

- Min. Collision Energy: 0

- Max. Collision Energy: 1000

- Scan Type: Is Full

4. Peak Filters:

- S/N Threshold (FT-only): 1.5

5. Replacements for Unrecognized Properties:

- Unrecognized Charge Replacements: Automatic

- Unrecognized Mass Analyzer Replacements: ITMS

- Unrecognized MS Order Replacements: MS2

- Unrecognized Activation Type Replacements: CID

- Unrecognized Polarity Replacements: +

- Unrecognized MS Resolution@200 Replacements: 60000

- Unrecognized MSn Resolution@200 Replacements: 30000

6. Precursor Pattern Extraction:

- Precursor Clipping Range Before: 2.5 Da

- Precursor Clipping Range After: 5.5 Da

------------------------------------------------------------------

Processing node 2: Sequest HT

------------------------------------------------------------------

1. Input Data:

- Protein Database: Human_uniprot-proteome_UP000005640.fasta

- Enzyme Name: Trypsin (Full)

- Max. Missed Cleavage Sites: 2

- Min. Peptide Length: 6

- Max. Peptide Length: 144

- Max. Number of Peptides Reported: 10

2. Tolerances:

- Precursor Mass Tolerance: 10 ppm

- Fragment Mass Tolerance: 0.8 Da

- Use Average Precursor Mass: False

- Use Average Fragment Mass: False

3. Spectrum Matching:

- Use Neutral Loss a Ions: True

- Use Neutral Loss b Ions: True

- Use Neutral Loss y Ions: True

- Use Flanking Ions: True

- Weight of a Ions: 0

- Weight of b Ions: 1

- Weight of c Ions: 0

- Weight of x Ions: 0

- Weight of y Ions: 1

- Weight of z Ions: 0

4. Dynamic Modifications:

- Max. Equal Modifications Per Peptide: 3

- Max. Dynamic Modifications Per Peptide: 4

- 1. Dynamic Modification: Oxidation / +15.995 Da (M)

- 2. Dynamic Modification: Acetyl / +42.011 Da (K)

- 3. Dynamic Modification: Ub-amide / +196.109 Da (K)

6. Dynamic Modifications (protein terminus):

- 1. N-Terminal Modification: Acetyl / +42.011 Da (N-Terminus)

7. Static Modifications:

- 1. Static Modification: Carbamidomethyl / +57.021 Da (C)

------------------------------------------------------------------

Processing node 3: Fixed Value PSM Validator

------------------------------------------------------------------

1. Input Data:

- Maximum Delta Cn: 0.05

- Maximum Rank: 0

------------------------------------------------------------------

Workflow messages:

------------------------------------------------------------------

01/16/2024 12:09 PM Job Execution: Processing D:\2023\Internal\AMARJEET_SHARMA\New Study_16012024\Rheb_IISc_IG.msf

01/16/2024 12:09 PM (0) Spectrum Files RC: Start processing file F1: Rheb_IISc_IG.raw...

01/16/2024 12:09 PM (0) Spectrum Files RC: Retrieving 20965 spectra took 25.2 s

01/16/2024 12:09 PM (0) Spectrum Files RC: There is already an adequate target FASTA index for Human_uniprot-proteome_UP000005640(9efa730d-768d-4098-b716-a7717e0abc6e).fasta.

01/16/2024 12:09 PM (0) Spectrum Files RC: Start searching spectra (HCD (High Energy Collision Dissociation))...

01/16/2024 12:09 PM (0) Spectrum Files RC: Ise (2.0.0.24, x64) started at 1/16/2024 12:09:44 PM on DESKTOP-T4DR924 (x64) [32 CPUs] running Microsoft Windows NT 6.2.9200.0 (64bit) [.NET: 4.0.30319.42000]

01/16/2024 12:09 PM (0) Spectrum Files RC: Workload level: #parallel tasks: 10

01/16/2024 12:09 PM (0) Spectrum Files RC: Workload level: #spectra loaded and processed at once: 10000

01/16/2024 12:09 PM (0) Spectrum Files RC: On-Disk search is performed

01/16/2024 12:09 PM (0) Spectrum Files RC: Average search time per spectrum was 0.5 ms.

01/16/2024 12:09 PM (0) Spectrum Files RC: Start reading spectrum results...

01/16/2024 12:09 PM (0) Spectrum Files RC: Start calculating calibration...

01/16/2024 12:09 PM (0) Spectrum Files RC: Calibrated F1 using constant shift of 0.0 ppm because number of landmarks was only 67.

01/16/2024 12:09 PM (0) Spectrum Files RC: Processing file F1 took 38.1 s.

01/16/2024 12:09 PM (0) Spectrum Files RC: -- Total execution of Spectrum Files RC (0) took 38.1 s --

01/16/2024 12:09 PM (1) Spectrum Selector: Profile spectra are not sent.

01/16/2024 12:09 PM (1) Spectrum Selector: Reading from file 1 of 1 F1: H:\Rheb_IISc_IG.raw (24461 spectra total)

01/16/2024 12:09 PM (2) Sequest HT: Sequence Database: Human_uniprot-proteome_UP000005640.fasta

01/16/2024 12:10 PM (1) Spectrum Selector: Sent 19316 spectra from file F1.

01/16/2024 12:10 PM (1) Spectrum Selector: Sent 19316 spectra from 1 files (processing time: 17.5 s).

01/16/2024 12:10 PM (1) Spectrum Selector: -- Total execution of Spectrum Selector (1) took 56.5 s --

01/16/2024 12:10 PM (2) Sequest HT: Storing spectra took 38.4 s.

01/16/2024 12:10 PM (2) Sequest HT: There is already an adequate target FASTA index for Human_uniprot-proteome_UP000005640(9efa730d-768d-4098-b716-a7717e0abc6e).fasta.

01/16/2024 12:10 PM (2) Sequest HT: Start Sequest HT target search for 19316 spectra (19316 precursors)...

01/16/2024 12:10 PM (2) Sequest HT: Ise (2.0.0.24, x64) started at 1/16/2024 12:10:53 PM on DESKTOP-T4DR924 (x64) [32 CPUs] running Microsoft Windows NT 6.2.9200.0 (64bit) [.NET: 4.0.30319.42000]

01/16/2024 12:10 PM (2) Sequest HT: Workload level: #parallel tasks: 10

01/16/2024 12:10 PM (2) Sequest HT: Workload level: #spectra loaded and processed at once: 10000

01/16/2024 12:10 PM (2) Sequest HT: On-Disk search is performed

01/16/2024 12:11 PM (2) Sequest HT: Average search time per spectrum was 3.1 ms.

01/16/2024 12:11 PM (2) Sequest HT: Performing target search took 1 min 2 s.

01/16/2024 12:12 PM (2) Sequest HT: Stored 26423 PSMs for 19316 spectra

01/16/2024 12:12 PM (2) Sequest HT: Discarded 70161 peptide(s) that did not match the conditions for protein terminal modifications.

01/16/2024 12:12 PM (2) Sequest HT: Reading search results took 3.8 s.

01/16/2024 12:12 PM (2) Sequest HT: Saving results took 2.6 s.

01/16/2024 12:12 PM (2) Sequest HT: Saving proteins took 5.5 s.

01/16/2024 12:12 PM (2) Sequest HT: Reading results took 12.2 s.

01/16/2024 12:12 PM (2) Sequest HT: Finalizing search results...

01/16/2024 12:12 PM (2) Sequest HT: -- Total search time was 1 min 15 s --

01/16/2024 12:12 PM (3) Fixed Value PSM Validator: Evaluating peptides of Sequest HT (2) started

01/16/2024 12:12 PM (3) Fixed Value PSM Validator: -- Total execution of Fixed Value PSM Validator (3) took 1.3 s --

01/16/2024 12:12 PM Job Execution: Finished D:\2023\Internal\AMARJEET_SHARMA\New Study_16012024\Rheb_IISc_IG.msf

01/16/2024 12:12 PM Job Execution: ----- Total Job execution took: 3 min 0 s. -----

====================================================================================================

Validation

====================================================================================================

================================================================================

Consensus Step Validation

================================================================================

Result name: Rheb_IISc_IG

Result file: F:\2023\Internal\AMARJEET_SHARMA\New Study_16012024\Rheb_IISc_IG.pdResult

Description: Result filtered for high confident peptides.

Workflow based on template: CWF_Basic

Creation date: 1/16/2024 12:09:17 PM

Created with Discoverer version: 2.5.0.400

------------------------------------------------------------------

Peptide Validator nodes:

------------------------------------------------------------------

------------------------------------------------------------------

Processing node 2: Peptide Validator

------------------------------------------------------------------

1. General Validation Settings:

- Validation Mode: Automatic (Control peptide level error rate if possible)

- Target FDR (Strict) for PSMs: 0.01

- Target FDR (Relaxed) for PSMs: 0.05

- Target FDR (Strict) for Peptides: 0.01

- Target FDR (Relaxed) for Peptides: 0.05

2. Specific Validation Settings:

- Validation Based on: q-Value

- Target/Decoy Selection for PSM Level FDR Calculation Based on Score: Automatic

- Reset Confidences for Nodes without Decoy Search (Fixed score thresholds): False

Additional information:

--------------------------------

Used validation mode: 'Automatic (Control peptide level error rate if possible)'.

No decoy search was performed for the following search nodes: - Sequest HT (A2) in workflow Workflow

. FDR + fixed threshold validation is used instead.

FDR evaluation will be started.

------------------------------------------------------------------

Protein Validator nodes:

------------------------------------------------------------------

------------------------------------------------------------------

Processing node 6: Protein FDR Validator

------------------------------------------------------------------

1. Confidence Thresholds:

- Target FDR (Strict): 0.01

- Target FDR (Relaxed): 0.05

================================================================================

Processing Step A: Validation

================================================================================

Result name: Rheb_IISc_IG

Result file: D:\2023\Internal\AMARJEET_SHARMA\New Study_16012024\Rheb_IISc_IG.msf

Description: Basic processing workflow with score threshold validation to be used for searches of low complexity samples or employing a small FASTA database. Specify the FASTA database and any additional modifications.

Workflow based on template: PWF_Tribrid_Basic_SequestHT

Creation date: 1/16/2024 12:09:15 PM

Created with Discoverer version: 2.5.0.400

------------------------------------------------------------------

Psm Validator nodes:

------------------------------------------------------------------

------------------------------------------------------------------

Processing node 3: Fixed Value PSM Validator

------------------------------------------------------------------

1. Input Data:

- Maximum Delta Cn: 0.05

- Maximum Rank: 0

------------------------------------------------------------------

Validation for Processing Node: Sequest HT (2)

------------------------------------------------------------------

No decoy search, confidences were assigned based on the following thresholds:

High Confidence Thresholds:

--------------------------------

Score used to assign confidences: XCorr Score Versus Charge

Minimal Score for charge state = 1: 1.2

Minimal Score for charge state = 2: 1.9

Minimal Score for charge state = 3: 2.3

Minimal Score for charge state = 4: 2.6

Minimal Score for charge state = 5: 2.6

Minimal Score for charge state = 6: 2.6

Minimal Score for charge state = 7: 2.6

Minimal Score for charge state > 7: 2.6

Medium Confidence Thresholds:

--------------------------------

Score used to assign confidences: XCorr Score Versus Charge

Minimal Score for charge state = 1: 0.7

Minimal Score for charge state = 2: 0.8

Minimal Score for charge state = 3: 1

Minimal Score for charge state = 4: 1.2

Minimal Score for charge state = 5: 1.2

Minimal Score for charge state = 6: 1.2

Minimal Score for charge state = 7: 1.2

Minimal Score for charge state > 7: 1.2

================================================================================

Filters and Counts

================================================================================

------------------------------------------------------------------

Applied display filters:

------------------------------------------------------------------

This file contains the following filters:

Row Filter for PSMs:

------------------------------------

Master Protein Accessions contains Q15382

------------------------------------

Row Filter for Proteins:

------------------------------------

Master is equal to Master

------------------------------------

Row Filter for Input Files:

------------------------------------

FileName does not end with msf

------------------------------------

------------------------------------------------------------------

Number of result items:

------------------------------------------------------------------

Proteins:

7509 filtered / 25457 included / 29387 total

Protein Groups:

7509 included / 10781 total

Peptide Groups:

19902 included / 24650 total

PSMs:

47 filtered / 21353 included / 26423 total

MS/MS Spectrum Info:

19316 total

Input Files:

1 filtered / 2 included / 2 total

Study Information:

1 total

Specialized Traces:

2 total

================================================================================

FDR Values for Entire Result

================================================================================

No FDR values available (no decoy search results from at least one search node).

====================================================================================================

Configuration

====================================================================================================

================================================================================

Consensus Workflow Configuration

================================================================================

Result name: Rheb_IISc_IG

Result file: F:\2023\Internal\AMARJEET_SHARMA\New Study_16012024\Rheb_IISc_IG.pdResult

Description: Result filtered for high confident peptides.

Workflow based on template: CWF_Basic

Creation date: 1/16/2024 12:09:17 PM

Created with Discoverer version: 2.5.0.400

------------------------------------------------------------------

Configuration for: MSF Files

------------------------------------------------------------------

Scores:

- PSM scores (Hidden):

Mascot: Ions Score

Sequest HT: XCorr

SEQUEST: XCorr

MSPepSearch: dot Score

MSPepSearch: rev-dot Score

MSPepSearch: MSPepSearch Score

PMI-Byonic: |Log Prob|

PMI-Byonic: Byonic Score

MS Amanda: Amanda Score

------------------------------------------------------------------

Configuration for: Protein Scorer

------------------------------------------------------------------

Configuration Settings for Protein Score 'SequestSummationScore':

Protein Scoring Options:

- Peptide Relevance Factor: 0.4

------------------------------------------------------------------

Configuration for: Display Settings

------------------------------------------------------------------

Default Display Filter:

- Default Filter Set (Hidden):

### Master Proteins Default Filter:

### Row Filter for TargetProtein:

### Master is equal to Master

###

'magellan filter set' 1 'MasterFilter.filterset' Filter 'TargetProtein' FilterProperties 1 'FilterConditionProperties/FilterScope' 'FilterConditionProperties/FilterScopeValueMainGrid' 1 NARY_AND 1 = FilterConditionProperties 1 'NamedComparableFilterCondition/DisplayPropertyHint' 'Master' property 'Thermo.PD.EntityDataFramework.MasterProteinAssessment, Thermo.Magellan.EntityDataFramework' 'IsMasterProtein' constant 'Thermo.PD.EntityDataFramework.MasterProteinAssessment, Thermo.Magellan.EntityDataFramework' 'IsMasterProtein'

Default Layout:

- Default Layout: (not specified)

================================================================================

Processing Workflow A: Configuration

================================================================================

Result name: Rheb_IISc_IG

Result file: D:\2023\Internal\AMARJEET_SHARMA\New Study_16012024\Rheb_IISc_IG.msf

Description: Basic processing workflow with score threshold validation to be used for searches of low complexity samples or employing a small FASTA database. Specify the FASTA database and any additional modifications.

Workflow based on template: PWF_Tribrid_Basic_SequestHT

Creation date: 1/16/2024 12:09:15 PM

Created with Discoverer version: 2.5.0.400

------------------------------------------------------------------

Configuration for: Spectrum Files RC

------------------------------------------------------------------

Search settings:

- Ion Series settings (Hidden):

CID: by

HCD: by

ECD: cyz

ETD: cyz

EThcD: bcyz

UVPD: abxyz

------------------------------------------------------------------

Configuration for: Sequest HT

------------------------------------------------------------------

1. Workload Level:

- Automatic: True

- Number of Spectra Processed At Once: 3000

- Number of Parallel Tasks: 0

2. XCorr Confidence Thresholds (low-resolution data):

- z=1: High Confidence XCorr: 1.5

- z=1: Medium Confidence XCorr: 0.7

- z=2: High Confidence XCorr: 2

- z=2: Medium Confidence XCorr: 0.9

- z=3: High Confidence XCorr: 2.5

- z=3: Medium Confidence XCorr: 1.2

- z>=4: High Confidence XCorr: 3

- z>=4: Medium Confidence XCorr: 1.5

3. XCorr Confidence Thresholds (high-resolution data):

- z=1: High Confidence XCorr: 1.2

- z=1: Medium Confidence XCorr: 0.7

- z=2: High Confidence XCorr: 1.9

- z=2: Medium Confidence XCorr: 0.8

- z=3: High Confidence XCorr: 2.3

- z=3: Medium Confidence XCorr: 1

- z>=4: High Confidence XCorr: 2.6

- z>=4: Medium Confidence XCorr: 1.2
