# Supplementary figures and images for: Sirtuin 2 inhibits global protein synthesis via Rheb-GTPase degradation (part 2 of 2)
Source: EMBO Rep. 2026 Mar 11;27(11):3001–34. doi: 10.1038/s44319-026-00724-5 (PMC13261059; doi:10.1038/s44319-026-00724-5)

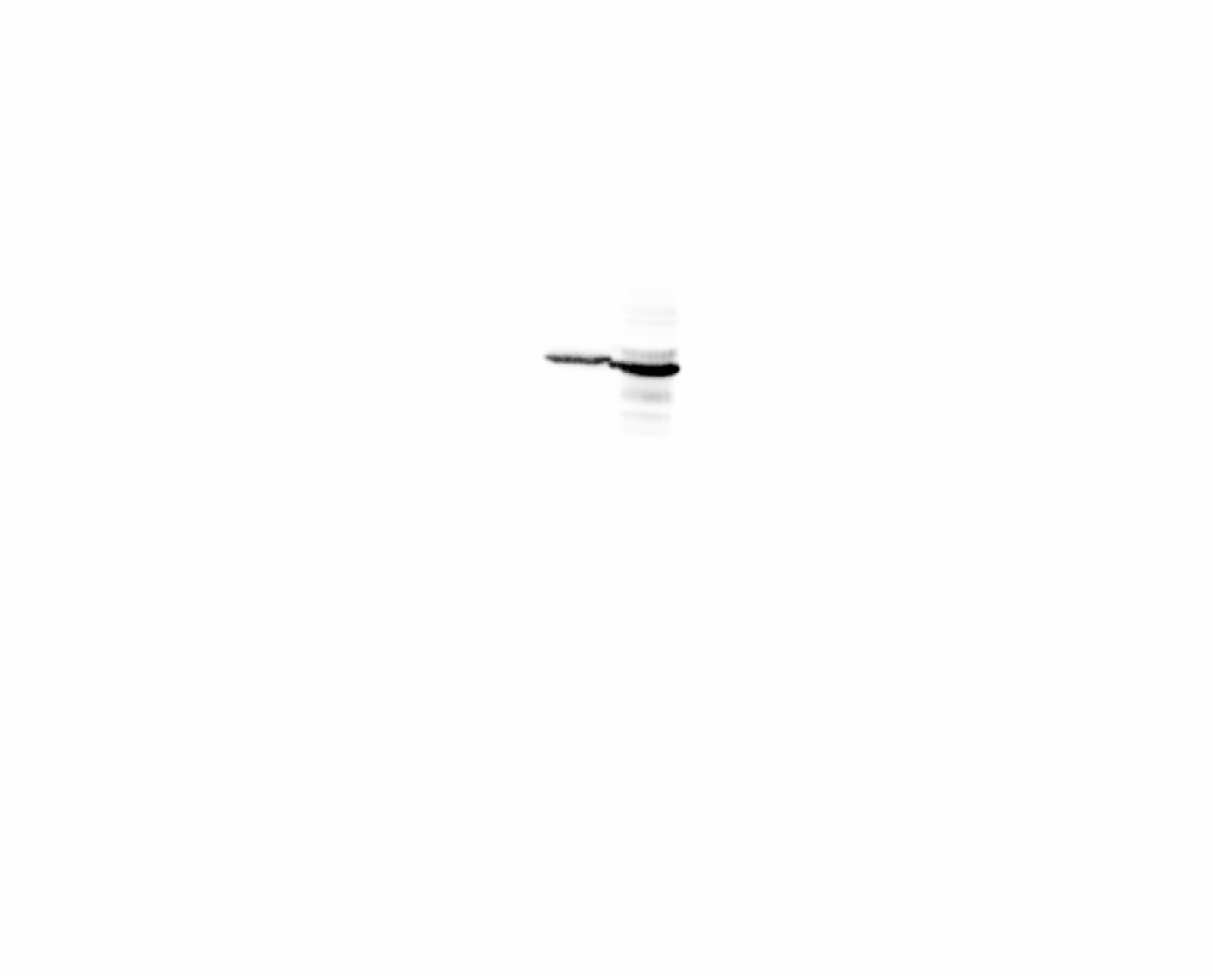

Supplement: Supplementary file 5 — Source data Fig. 4 [file 44319_2026_724_MOESM5_ESM.zip › Figure 4/4C/fig. 4C HA IP.tif]

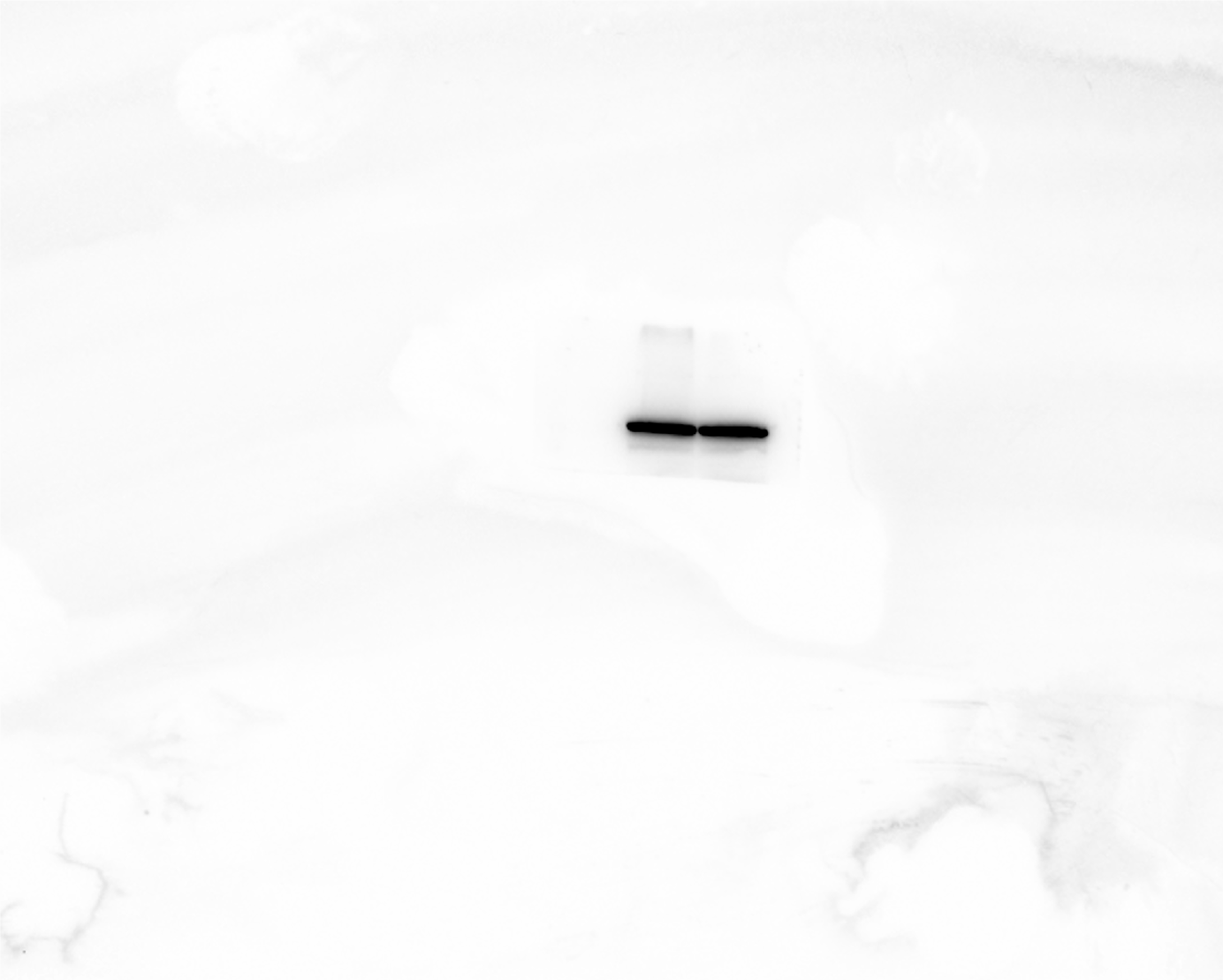

Supplement: Supplementary file 5 — Source data Fig. 4 [file 44319_2026_724_MOESM5_ESM.zip › Figure 4/4C/fig. 4C HSP90 input.tif]

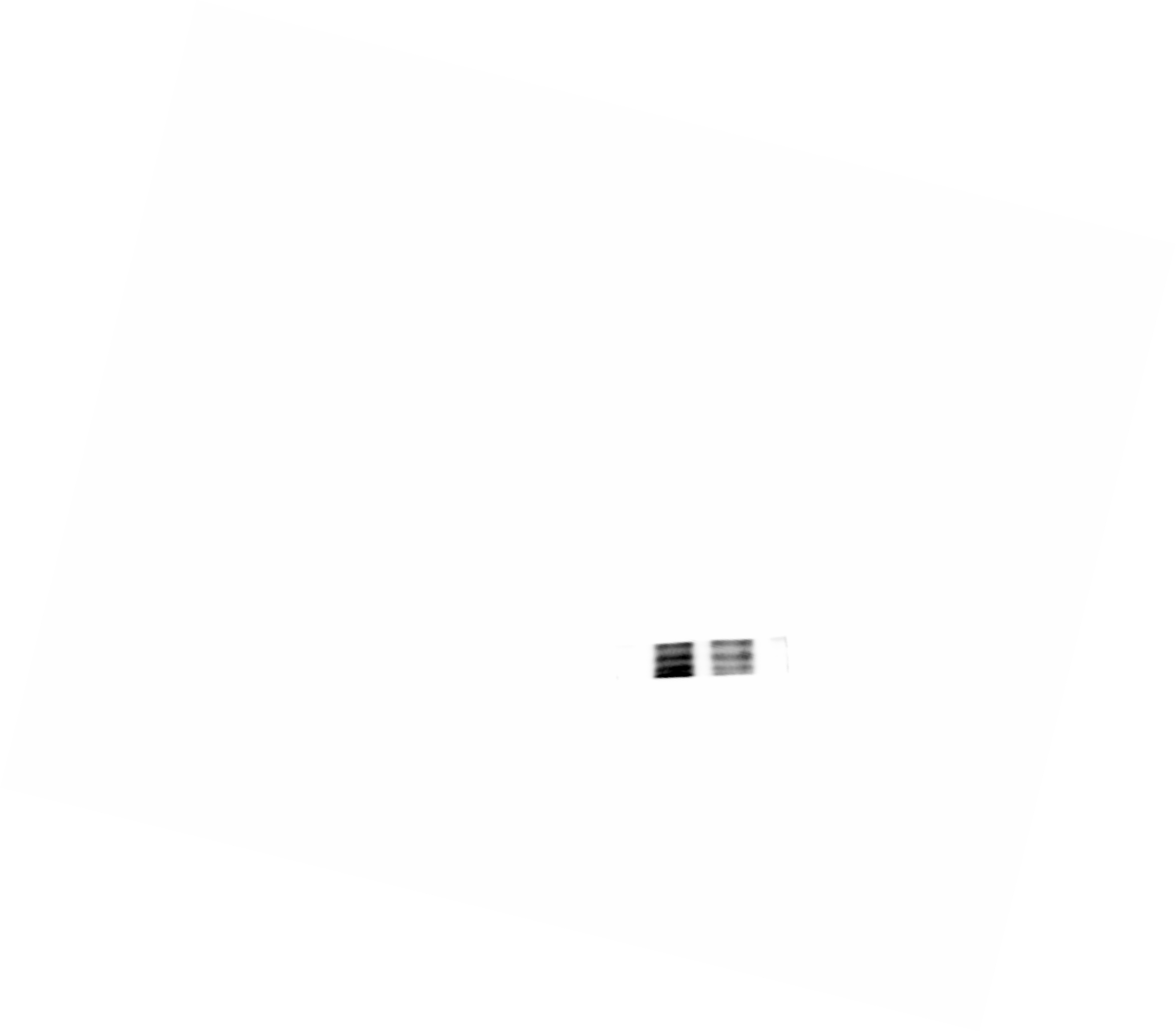

Supplement: Supplementary file 5 — Source data Fig. 4 [file 44319_2026_724_MOESM5_ESM.zip › Figure 4/4C/fig. 4C KAc IP.tif]

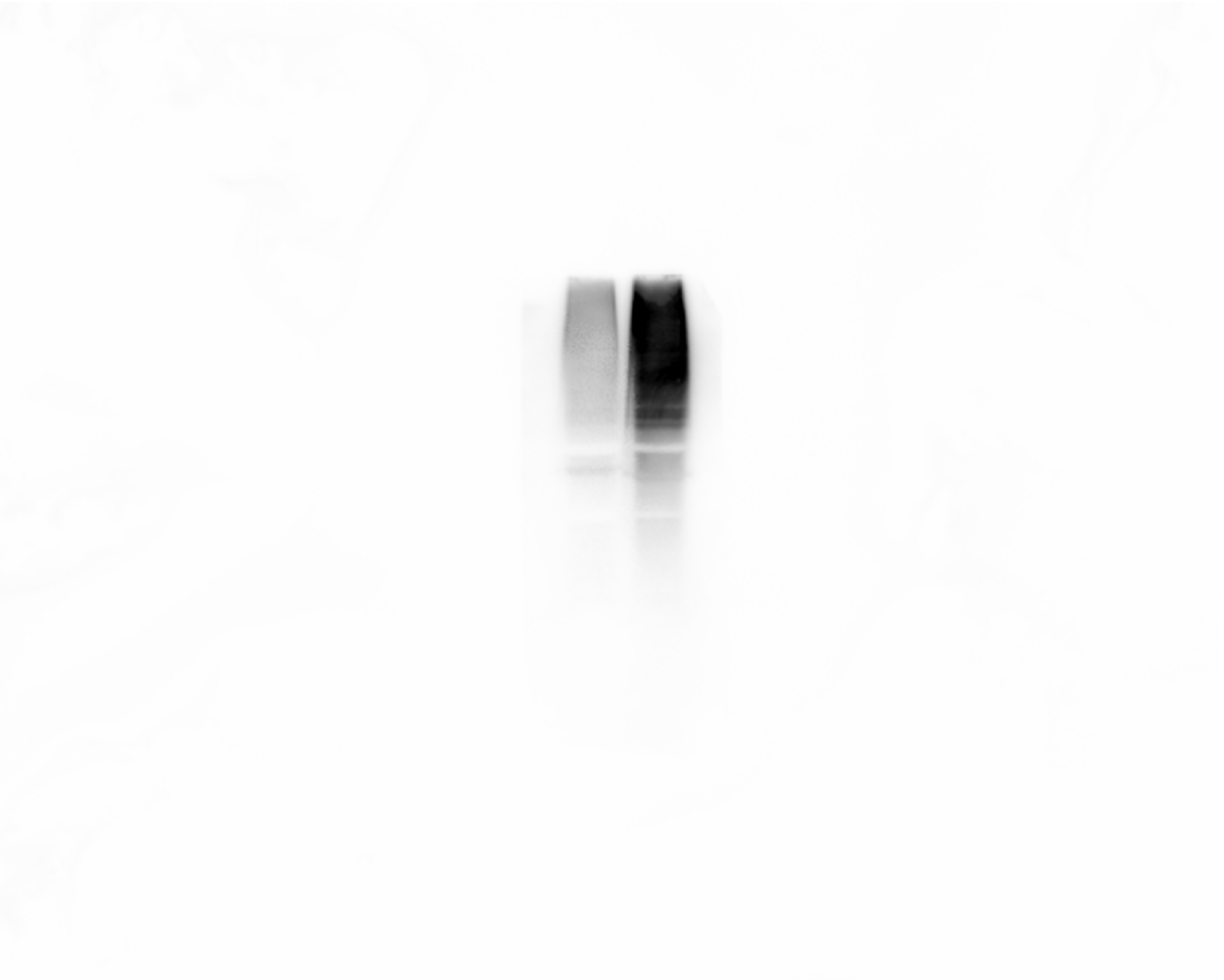

Supplement: Supplementary file 5 — Source data Fig. 4 [file 44319_2026_724_MOESM5_ESM.zip › Figure 4/4C/fig. 4C Ub IP.tif]

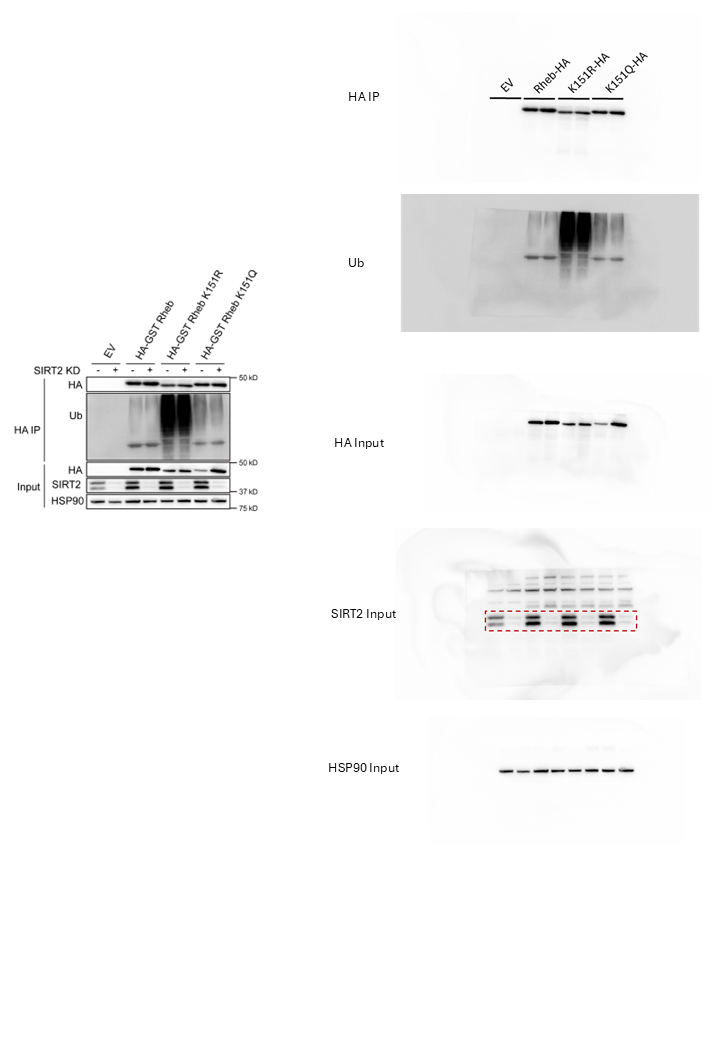

Supplement: Supplementary file 5 — Source data Fig. 4 [file 44319_2026_724_MOESM5_ESM.zip › Figure 4/4E/4E.tif]

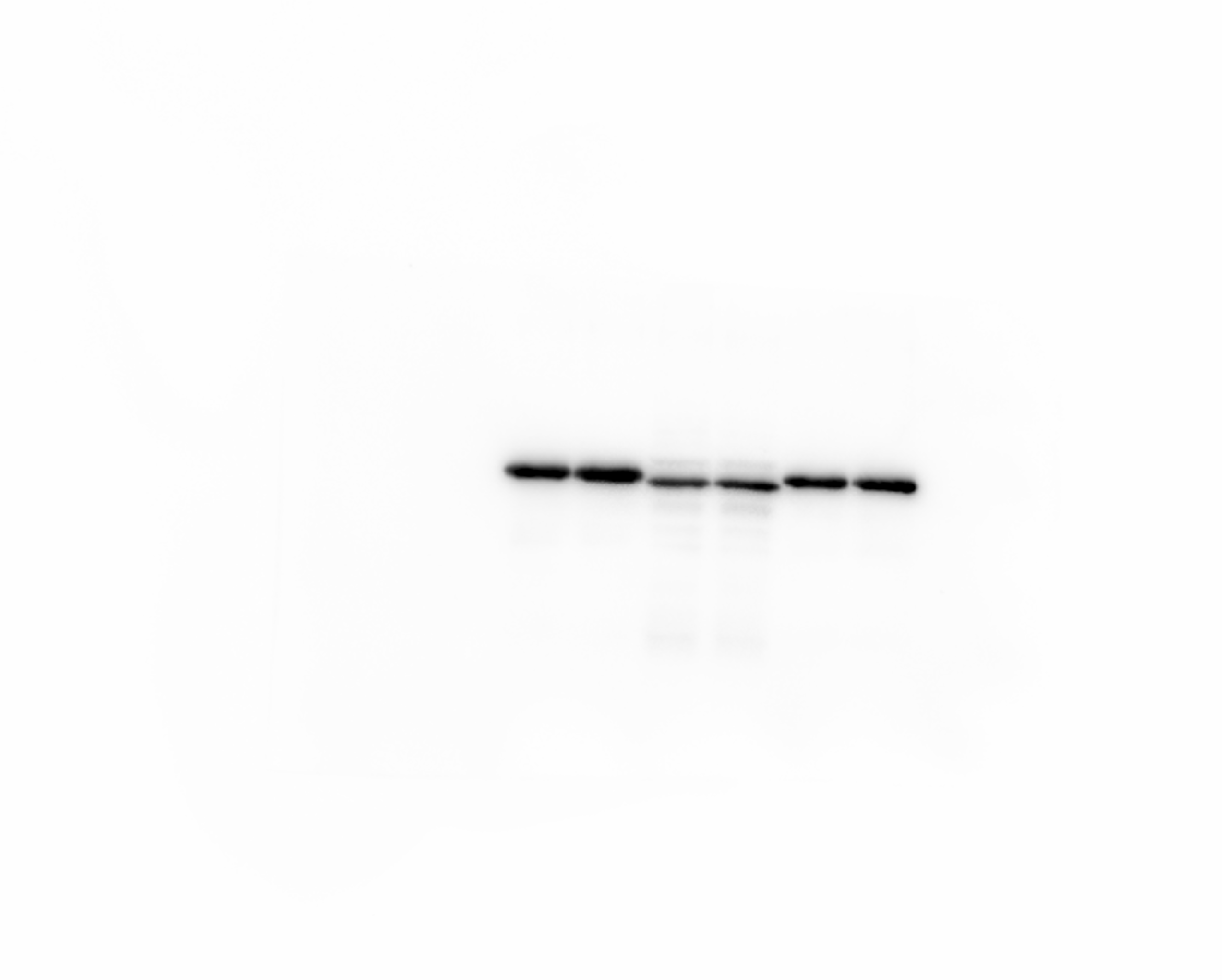

Supplement: Supplementary file 5 — Source data Fig. 4 [file 44319_2026_724_MOESM5_ESM.zip › Figure 4/4E/fig. 4E HA.tif]

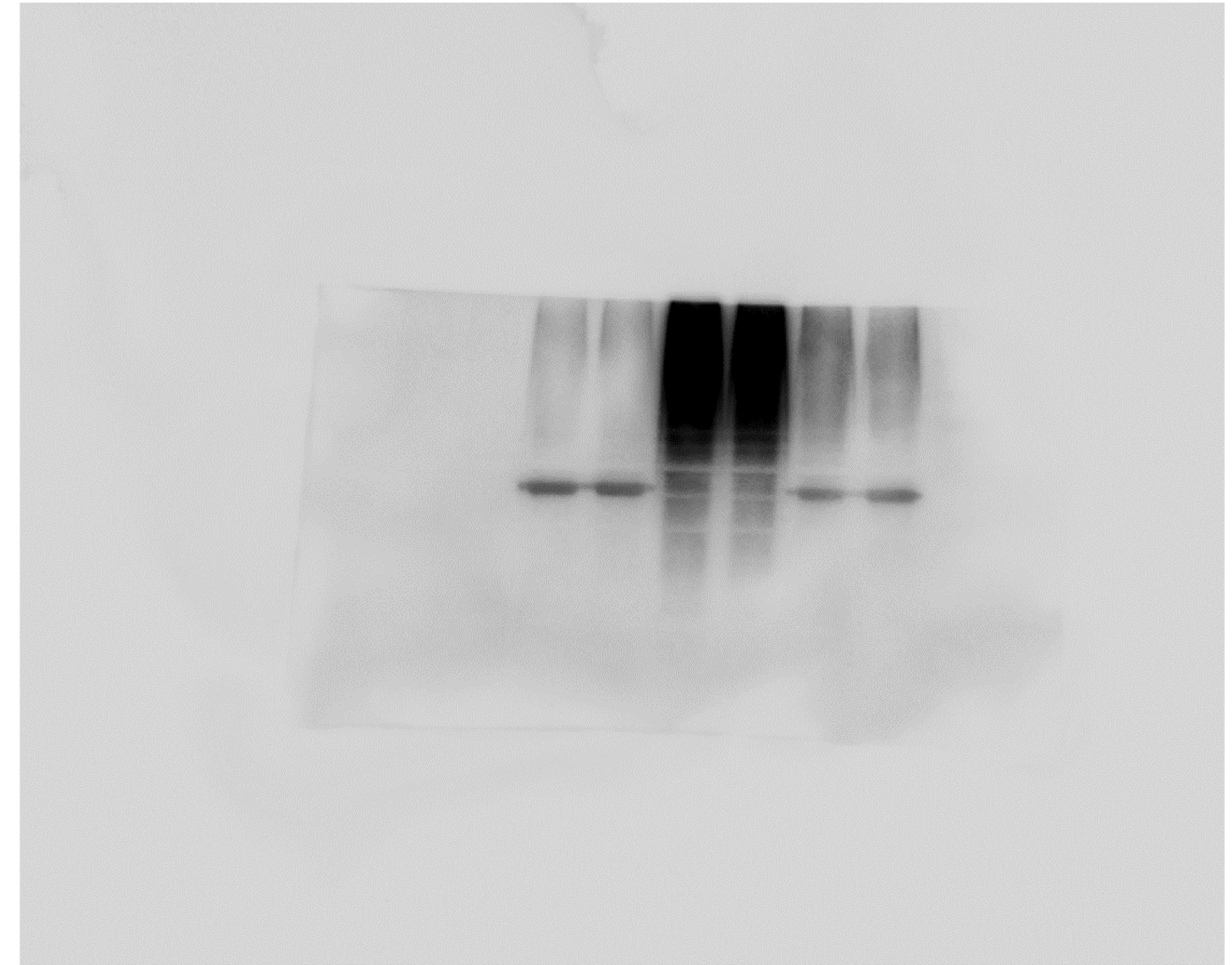

Supplement: Supplementary file 5 — Source data Fig. 4 [file 44319_2026_724_MOESM5_ESM.zip › Figure 4/4E/fig. 4E Ub.tif]

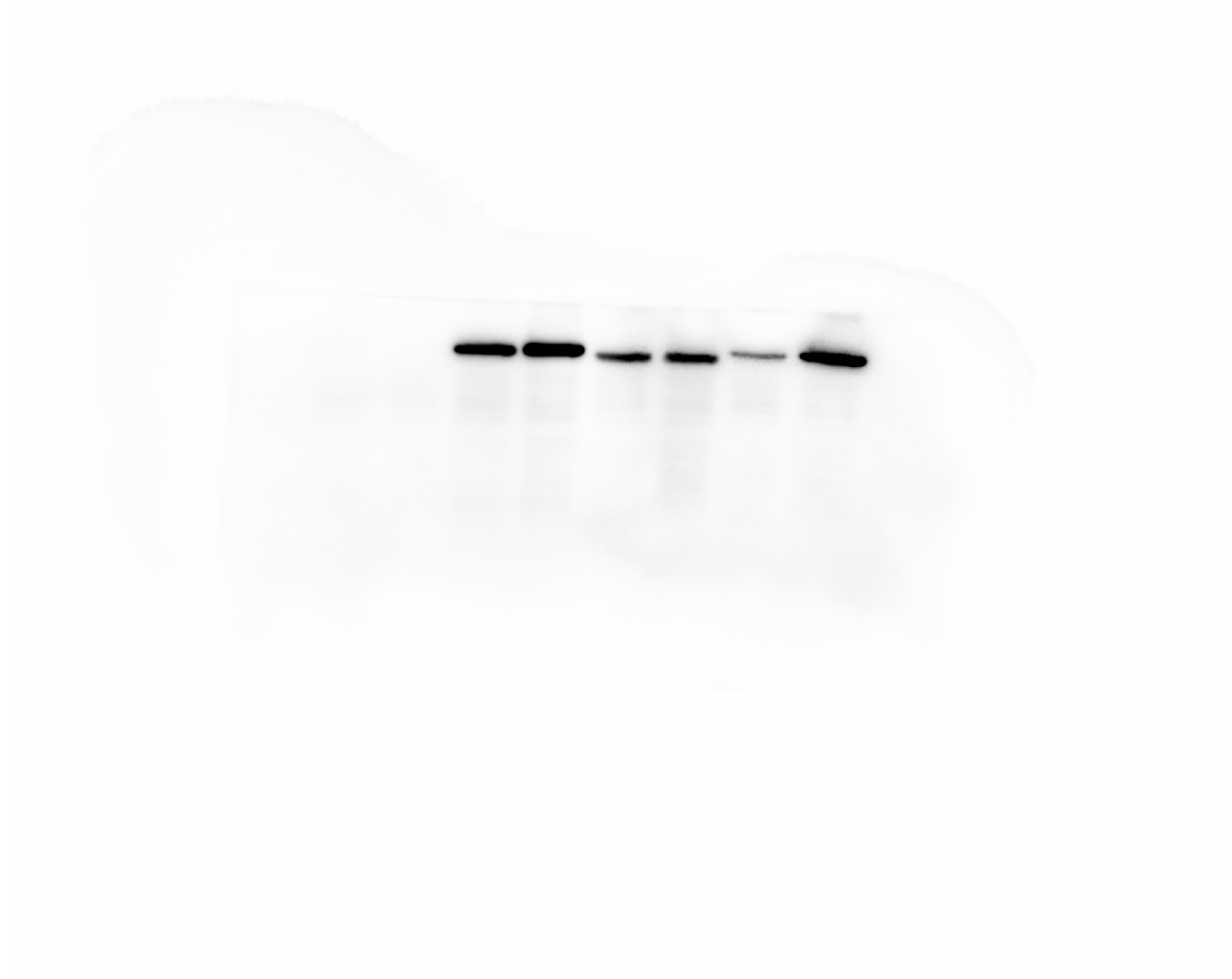

Supplement: Supplementary file 5 — Source data Fig. 4 [file 44319_2026_724_MOESM5_ESM.zip › Figure 4/4E/fig.4E HA input.tif]

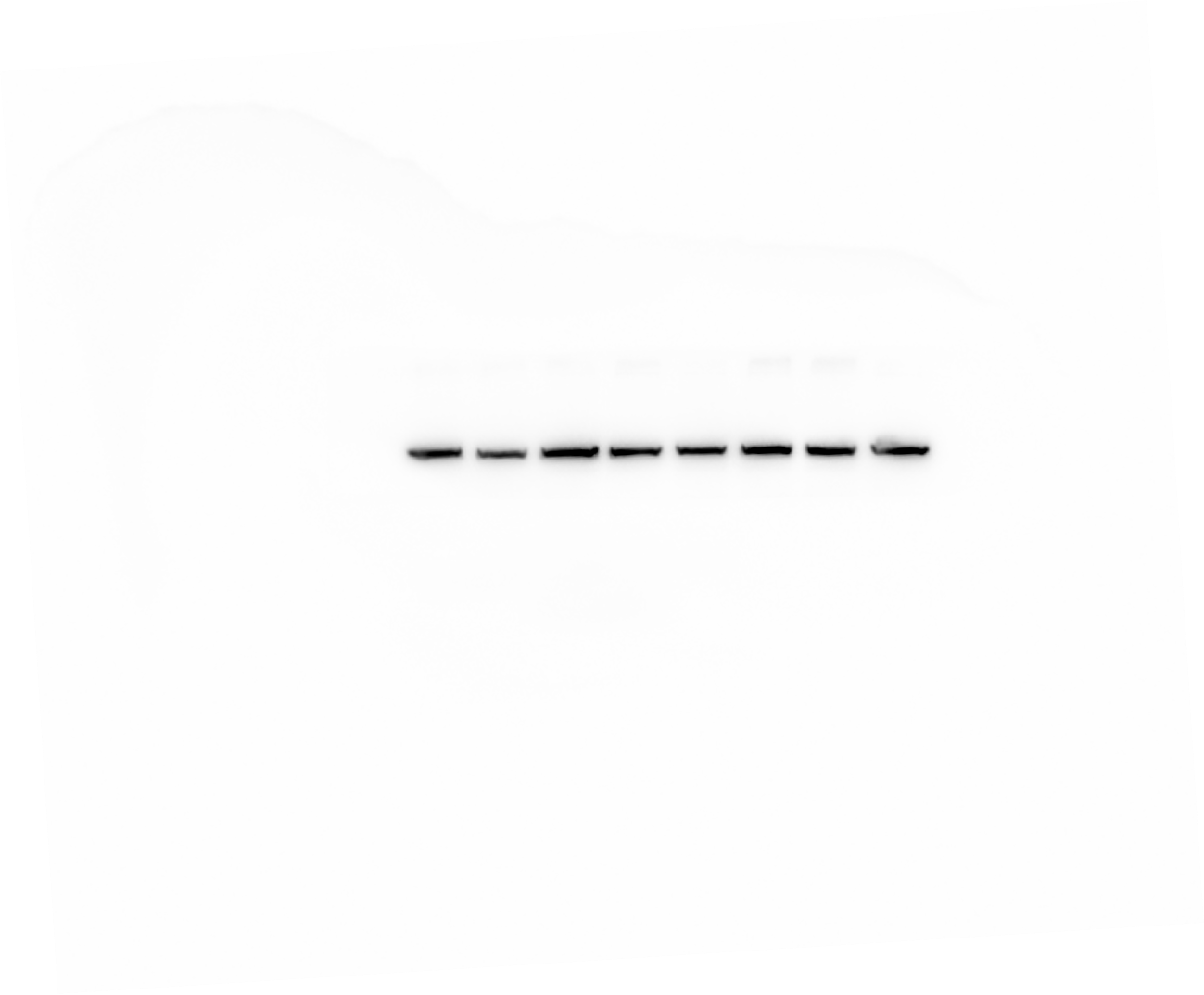

Supplement: Supplementary file 5 — Source data Fig. 4 [file 44319_2026_724_MOESM5_ESM.zip › Figure 4/4E/fig. 4E HSP90.tif]

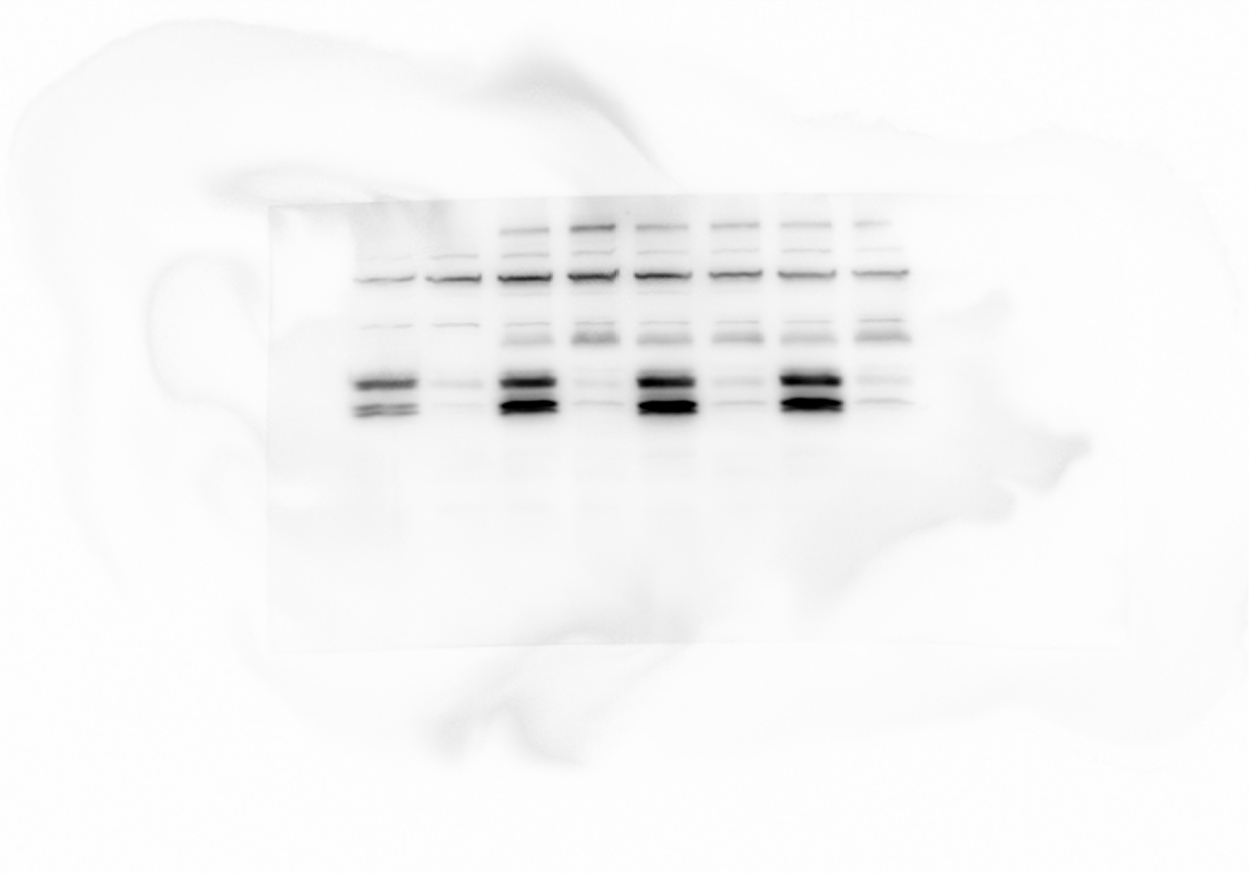

Supplement: Supplementary file 5 — Source data Fig. 4 [file 44319_2026_724_MOESM5_ESM.zip › Figure 4/4E/fig. 4E sirt2.tif]

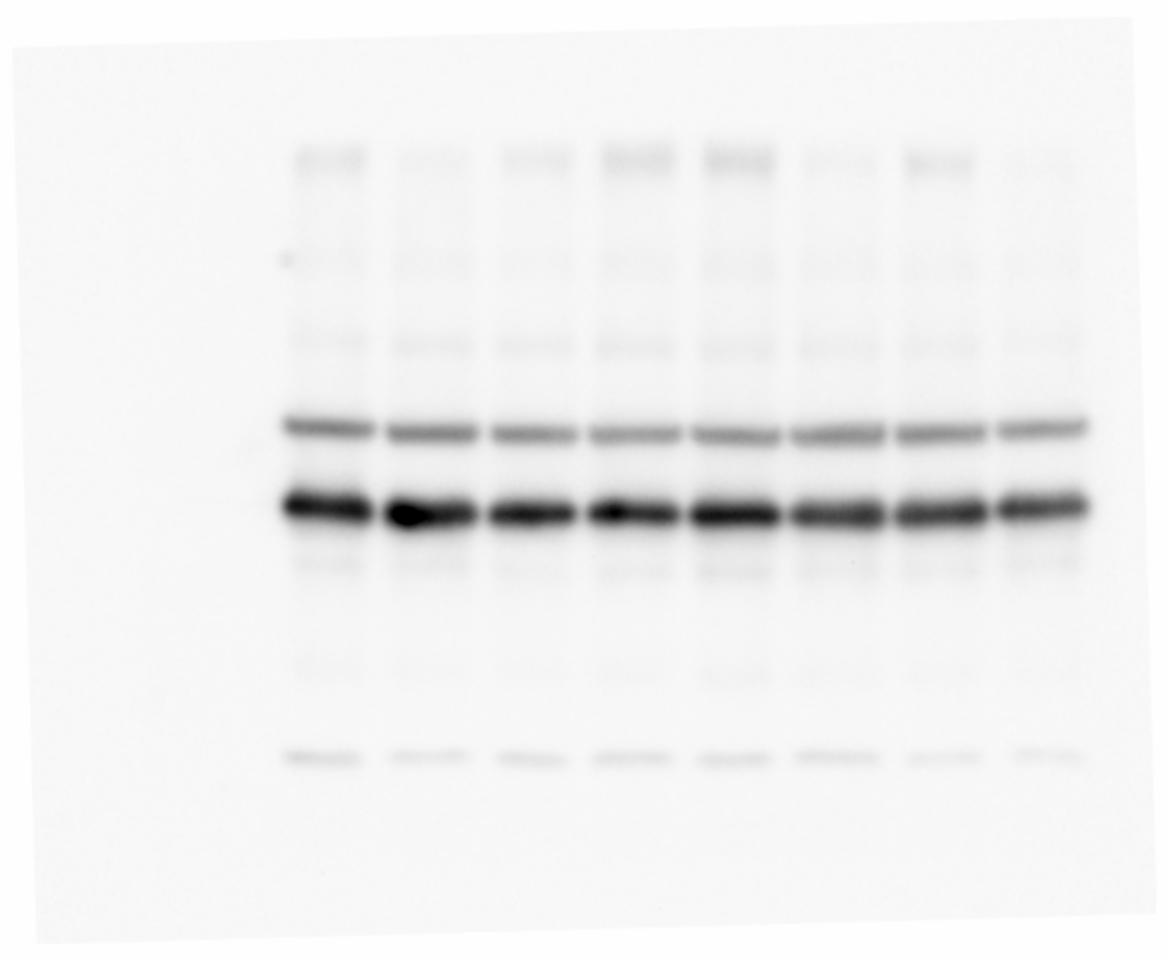

Supplement: Supplementary file 6 — Source data Fig. 6 [file 44319_2026_724_MOESM6_ESM.zip › Figure 6/Figure 6A/Figure 6A GAPDH.tif]

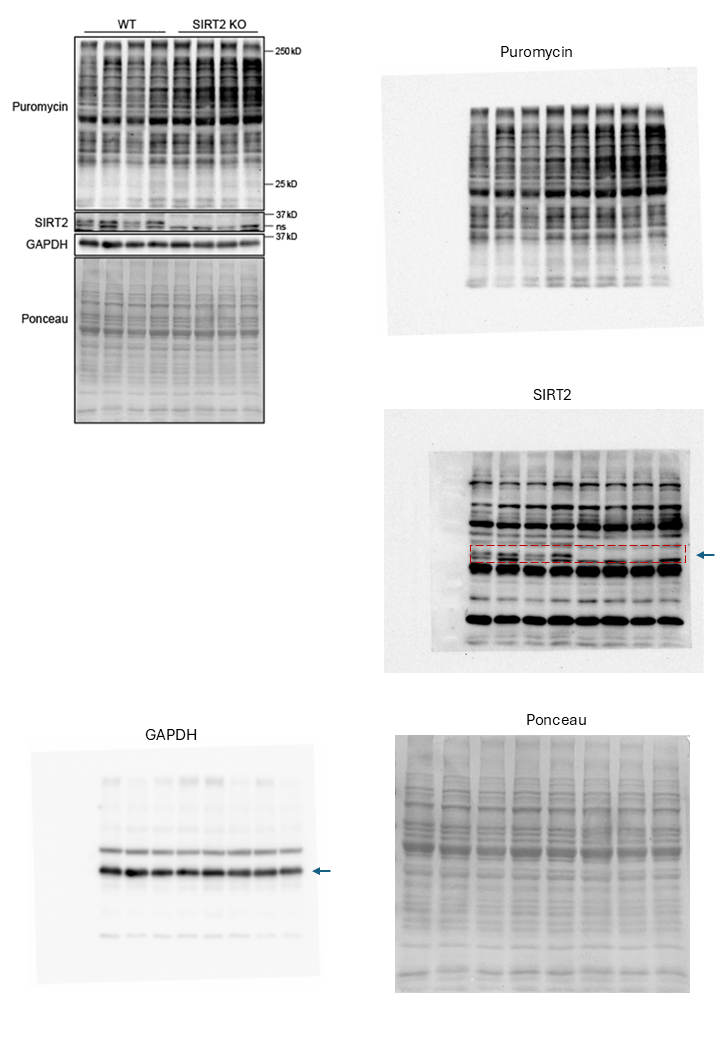

Supplement: Supplementary file 6 — Source data Fig. 6 [file 44319_2026_724_MOESM6_ESM.zip › Figure 6/Figure 6A/Figure 6A merged.tif]

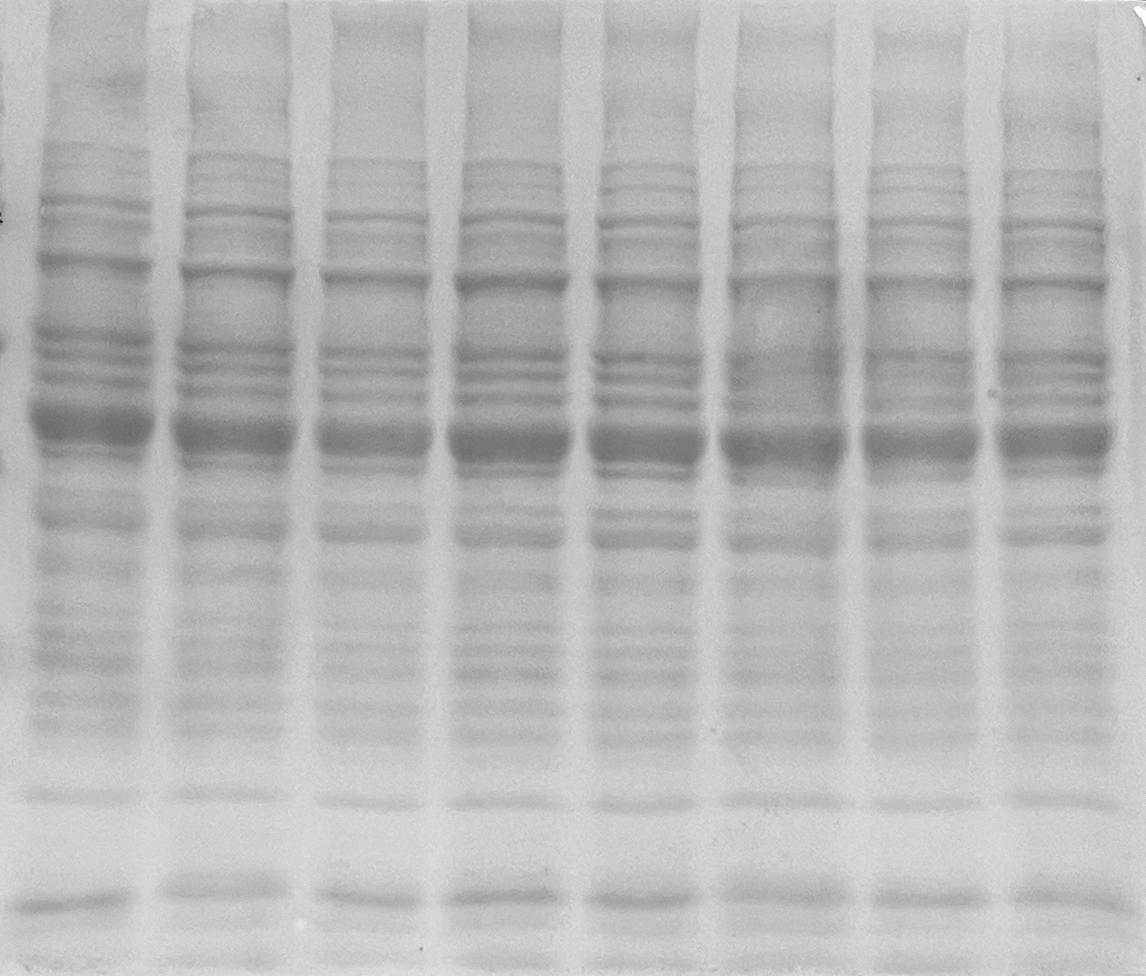

Supplement: Supplementary file 6 — Source data Fig. 6 [file 44319_2026_724_MOESM6_ESM.zip › Figure 6/Figure 6A/Figure 6A Ponceau.tif]

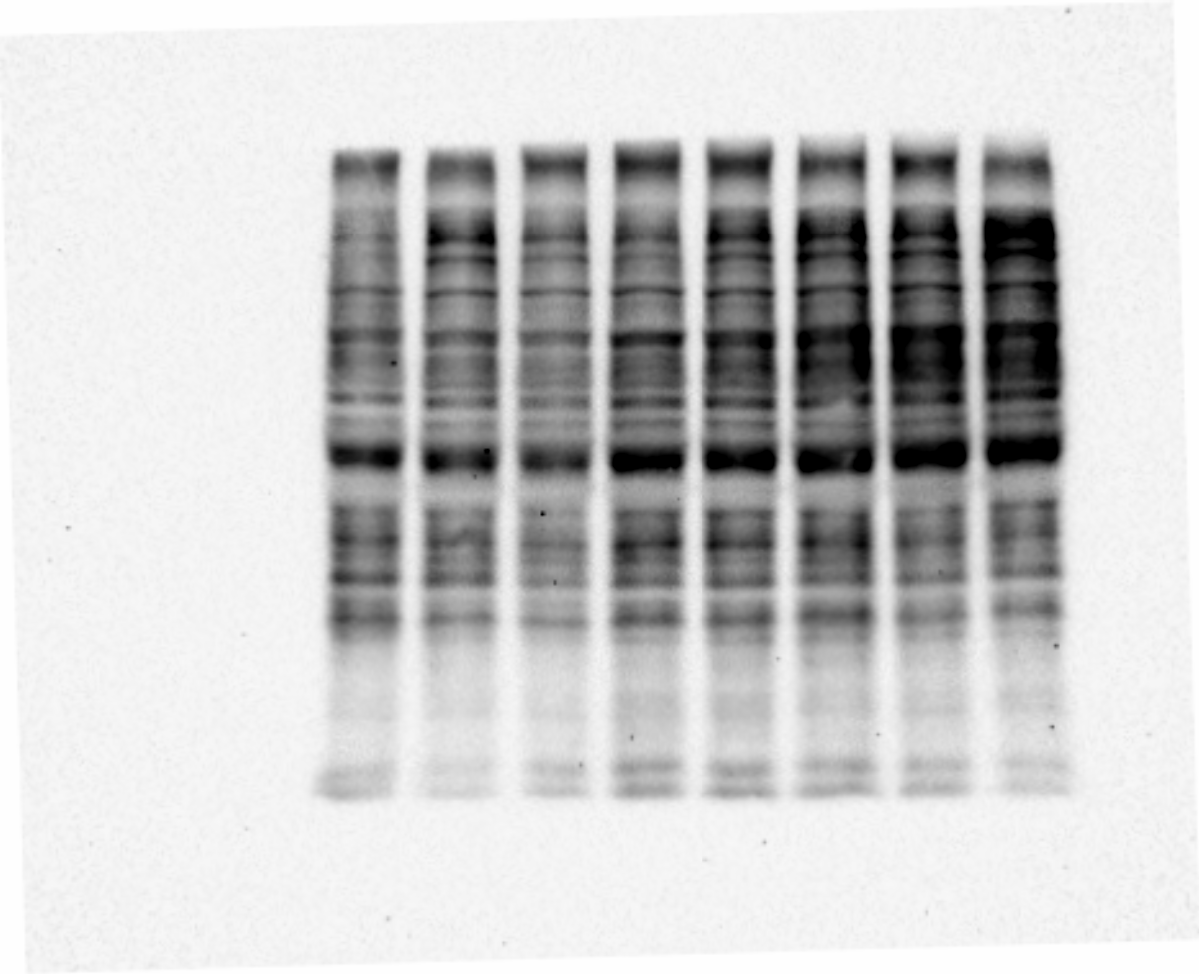

Supplement: Supplementary file 6 — Source data Fig. 6 [file 44319_2026_724_MOESM6_ESM.zip › Figure 6/Figure 6A/Figure 6A Puromycin.tif]

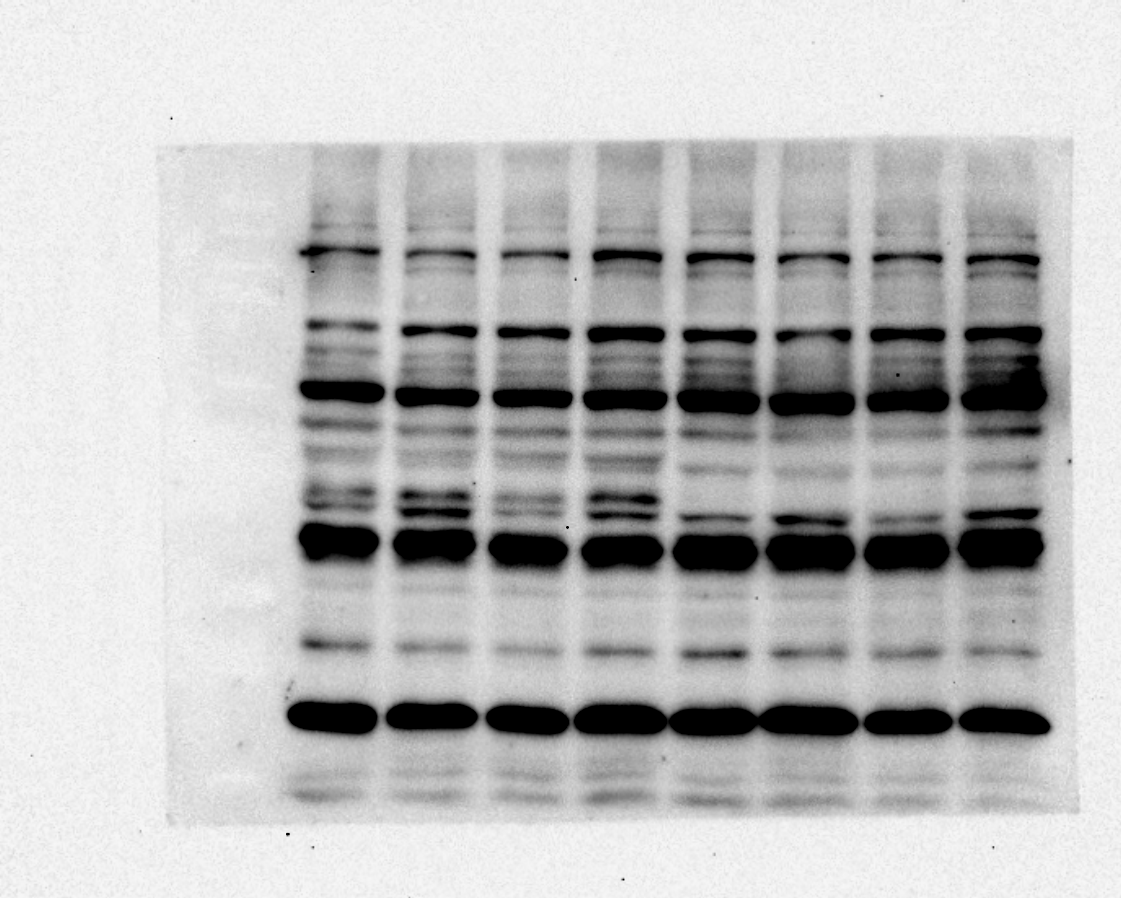

Supplement: Supplementary file 6 — Source data Fig. 6 [file 44319_2026_724_MOESM6_ESM.zip › Figure 6/Figure 6A/Figure 6A SIRT2.tif]

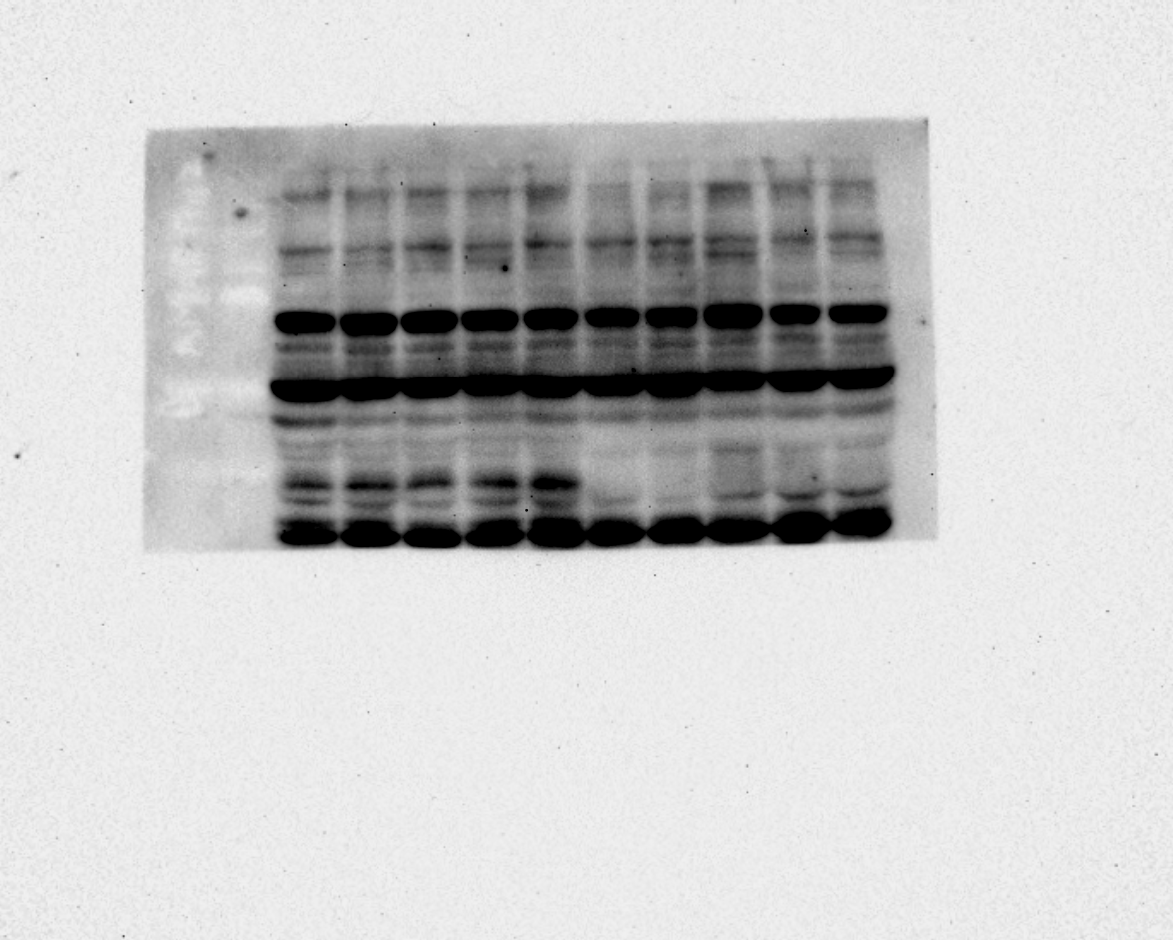

Supplement: Supplementary file 6 — Source data Fig. 6 [file 44319_2026_724_MOESM6_ESM.zip › Figure 6/Figure 6C/Figure 6C extra Resolved SIRT2.tif]

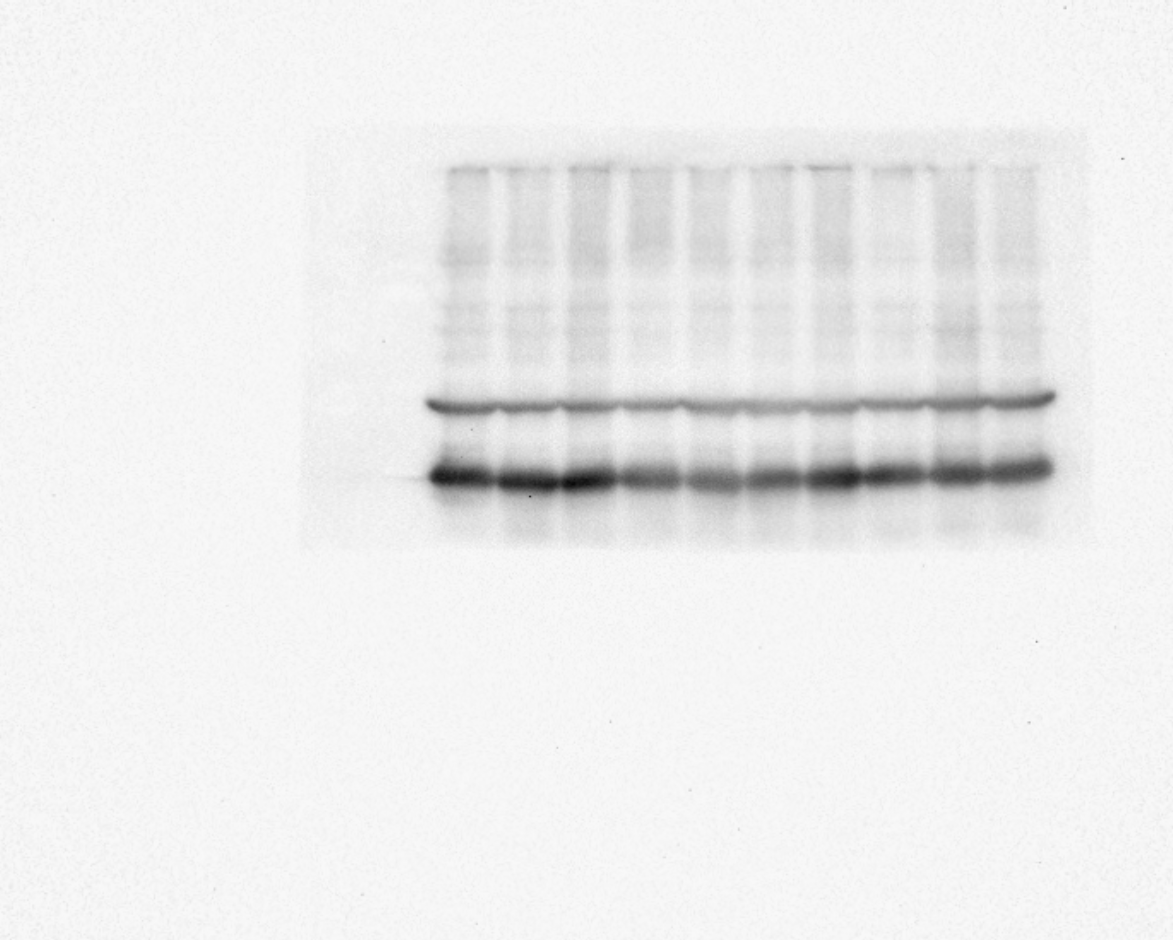

Supplement: Supplementary file 6 — Source data Fig. 6 [file 44319_2026_724_MOESM6_ESM.zip › Figure 6/Figure 6C/Figure 6C GAPDH extra resolved blot.tif]

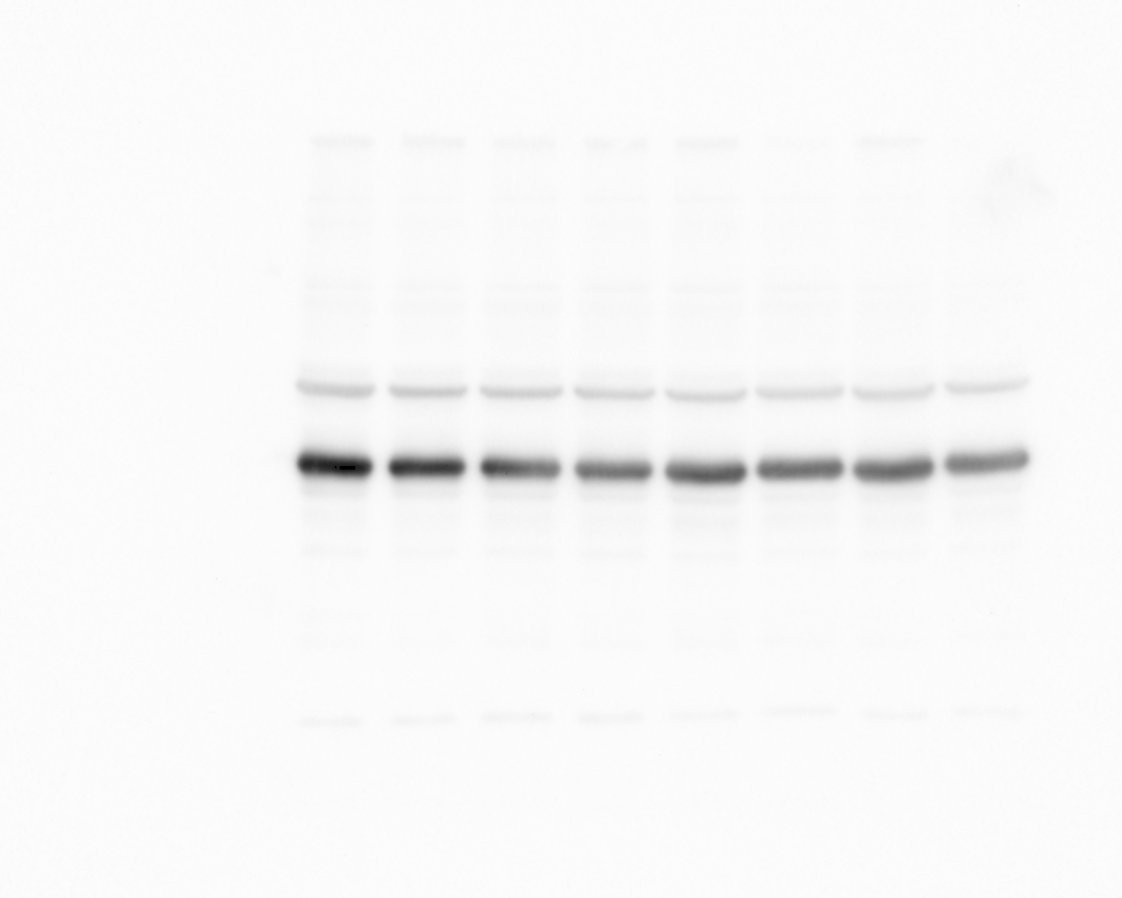

Supplement: Supplementary file 6 — Source data Fig. 6 [file 44319_2026_724_MOESM6_ESM.zip › Figure 6/Figure 6C/Figure 6C GAPDH.tif]

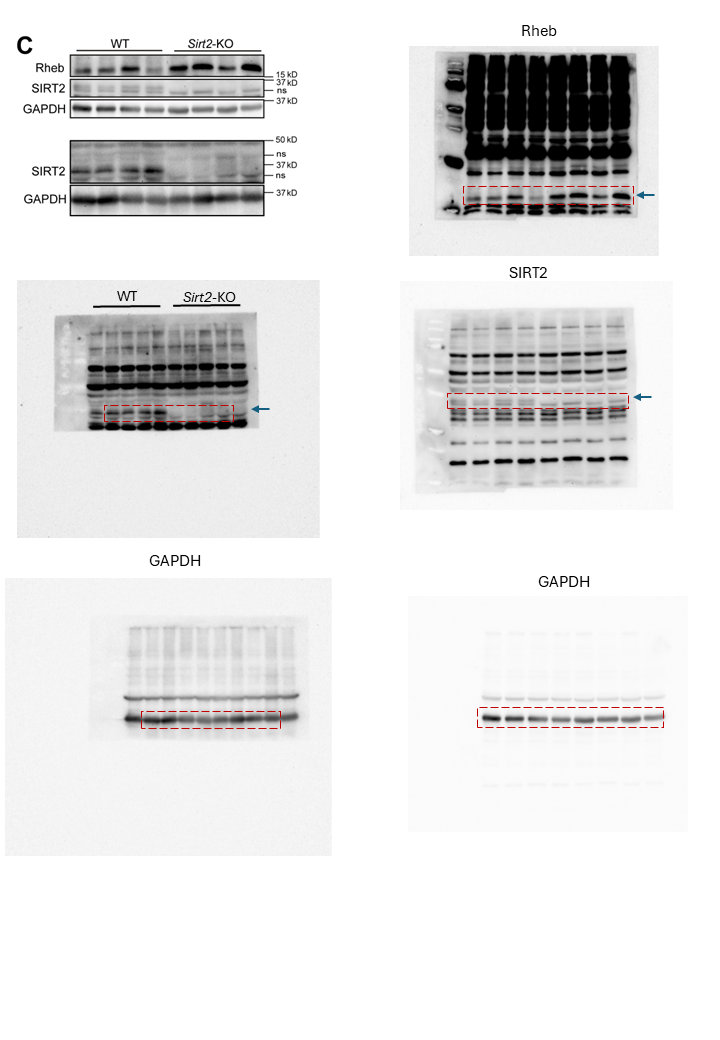

Supplement: Supplementary file 6 — Source data Fig. 6 [file 44319_2026_724_MOESM6_ESM.zip › Figure 6/Figure 6C/Figure 6C merged.tif]

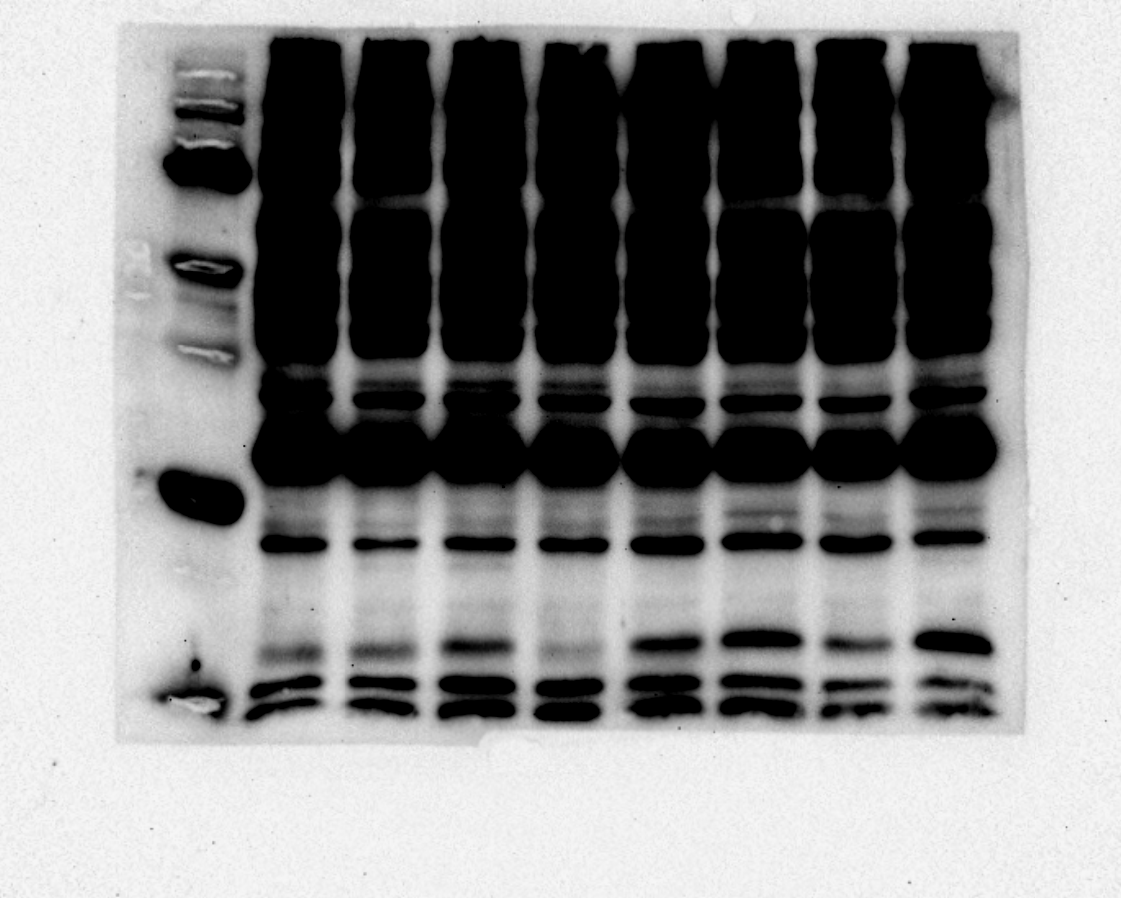

Supplement: Supplementary file 6 — Source data Fig. 6 [file 44319_2026_724_MOESM6_ESM.zip › Figure 6/Figure 6C/Figure 6C Rheb.tif]

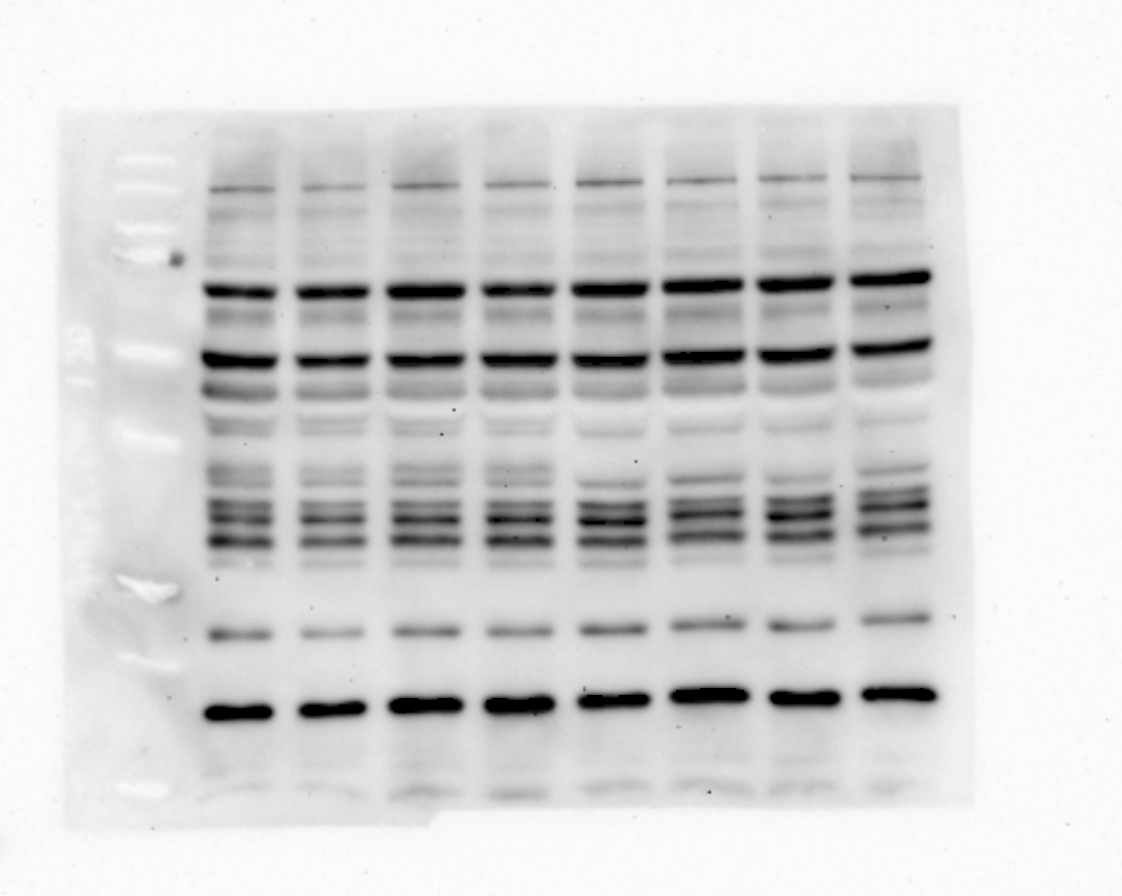

Supplement: Supplementary file 6 — Source data Fig. 6 [file 44319_2026_724_MOESM6_ESM.zip › Figure 6/Figure 6C/Figure 6C SIRT2.tif]

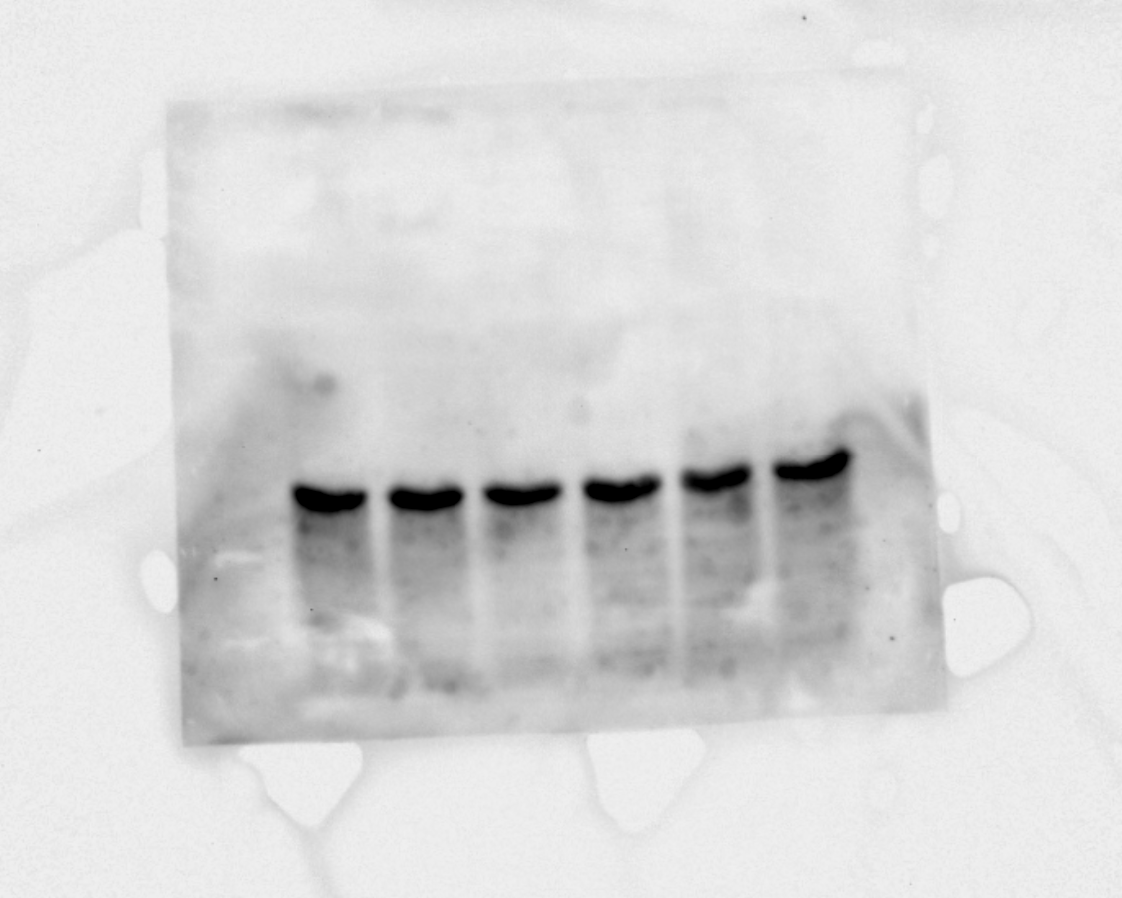

Supplement: Supplementary file 6 — Source data Fig. 6 [file 44319_2026_724_MOESM6_ESM.zip › Figure 6/Figure 6E/Figure 6E GAPDH.tif]

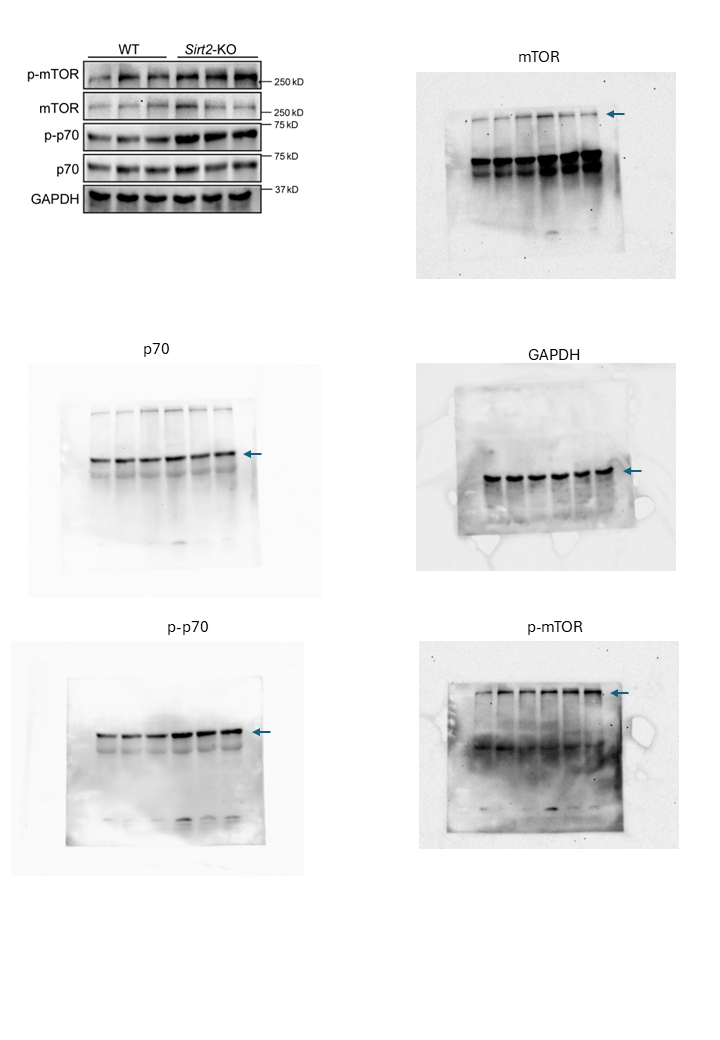

Supplement: Supplementary file 6 — Source data Fig. 6 [file 44319_2026_724_MOESM6_ESM.zip › Figure 6/Figure 6E/Figure 6E merged.tif]

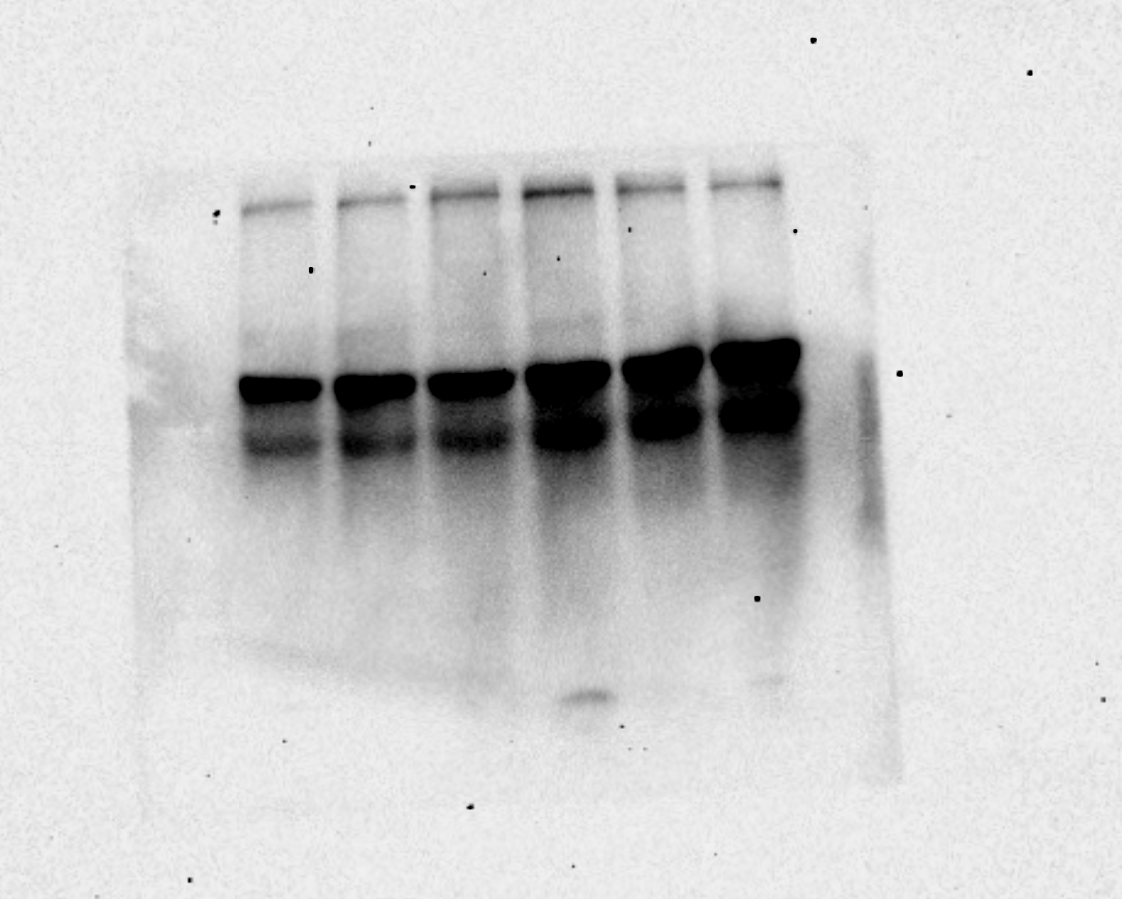

Supplement: Supplementary file 6 — Source data Fig. 6 [file 44319_2026_724_MOESM6_ESM.zip › Figure 6/Figure 6E/Figure 6E mTOR.tif]

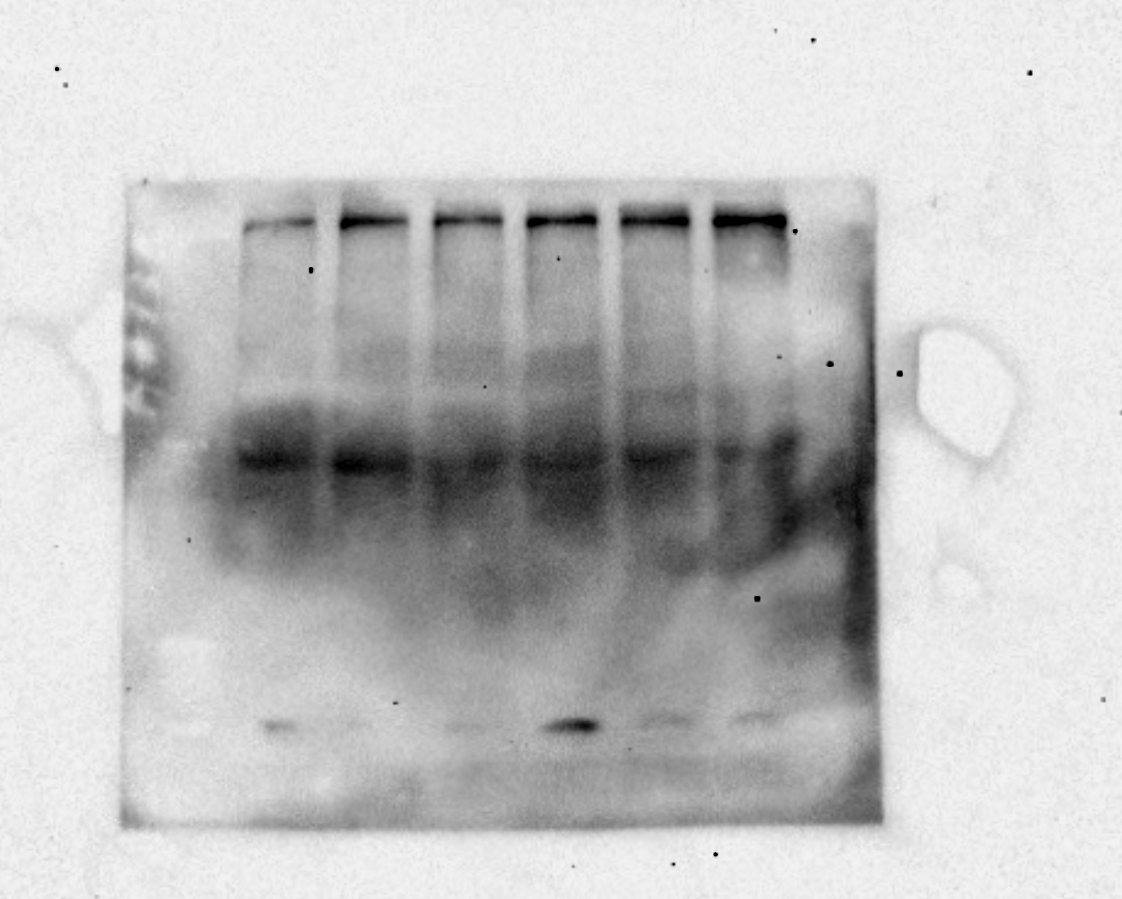

Supplement: Supplementary file 6 — Source data Fig. 6 [file 44319_2026_724_MOESM6_ESM.zip › Figure 6/Figure 6E/Figure 6E p-mTOR.tif]

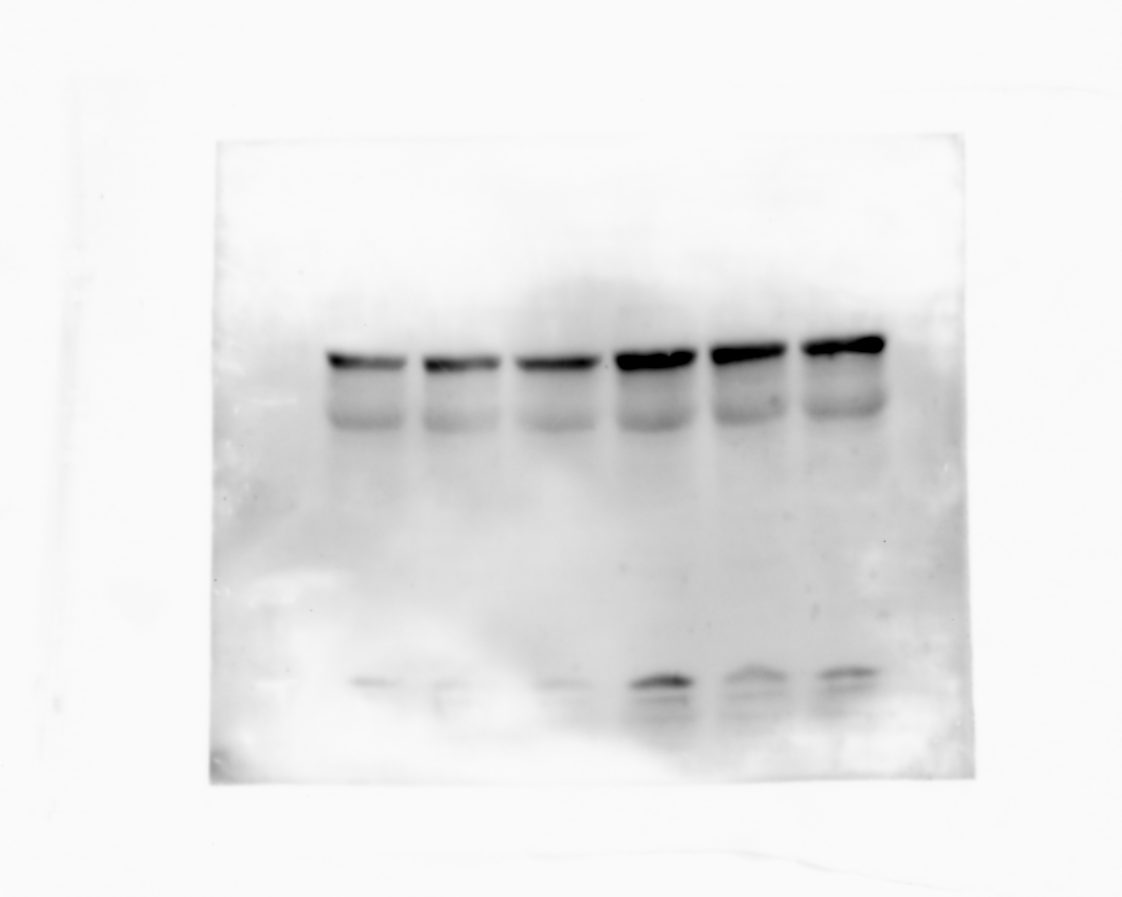

Supplement: Supplementary file 6 — Source data Fig. 6 [file 44319_2026_724_MOESM6_ESM.zip › Figure 6/Figure 6E/Figure 6E p-p70.tif]

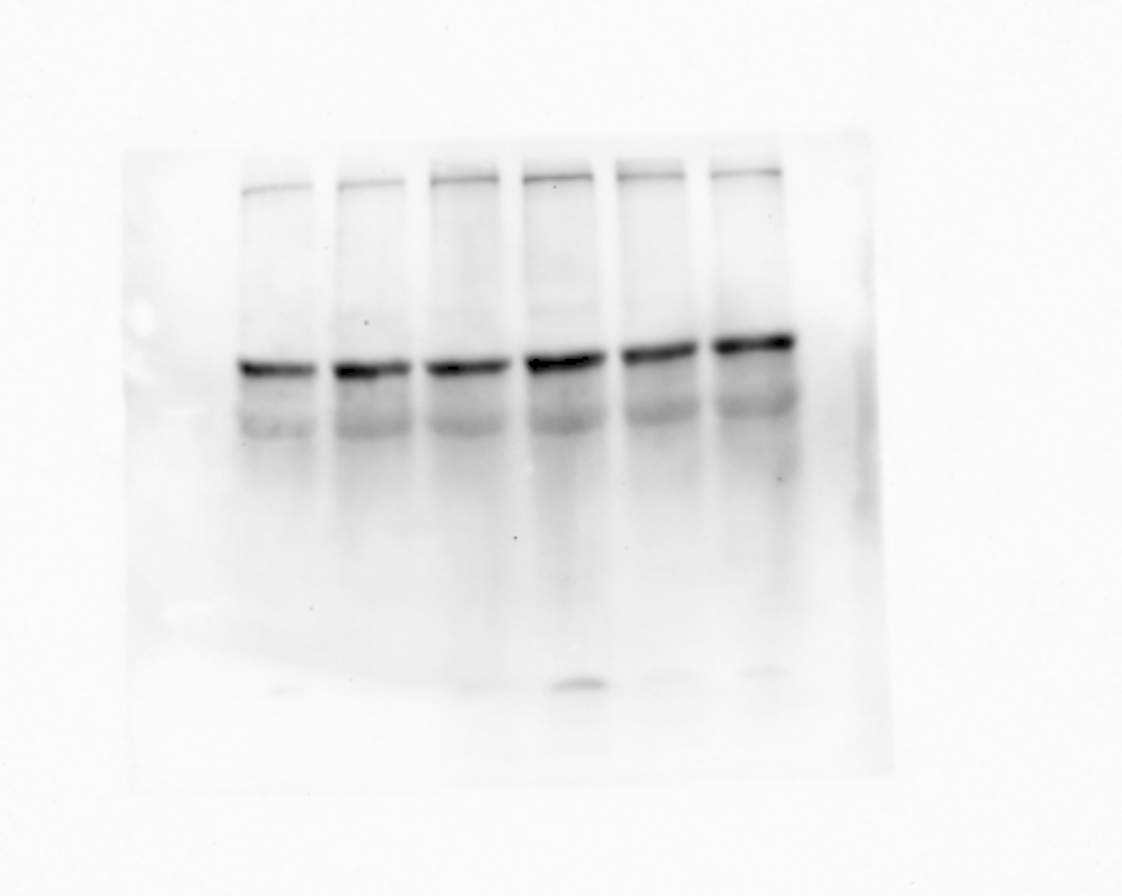

Supplement: Supplementary file 6 — Source data Fig. 6 [file 44319_2026_724_MOESM6_ESM.zip › Figure 6/Figure 6E/Figure 6E p70.tif]

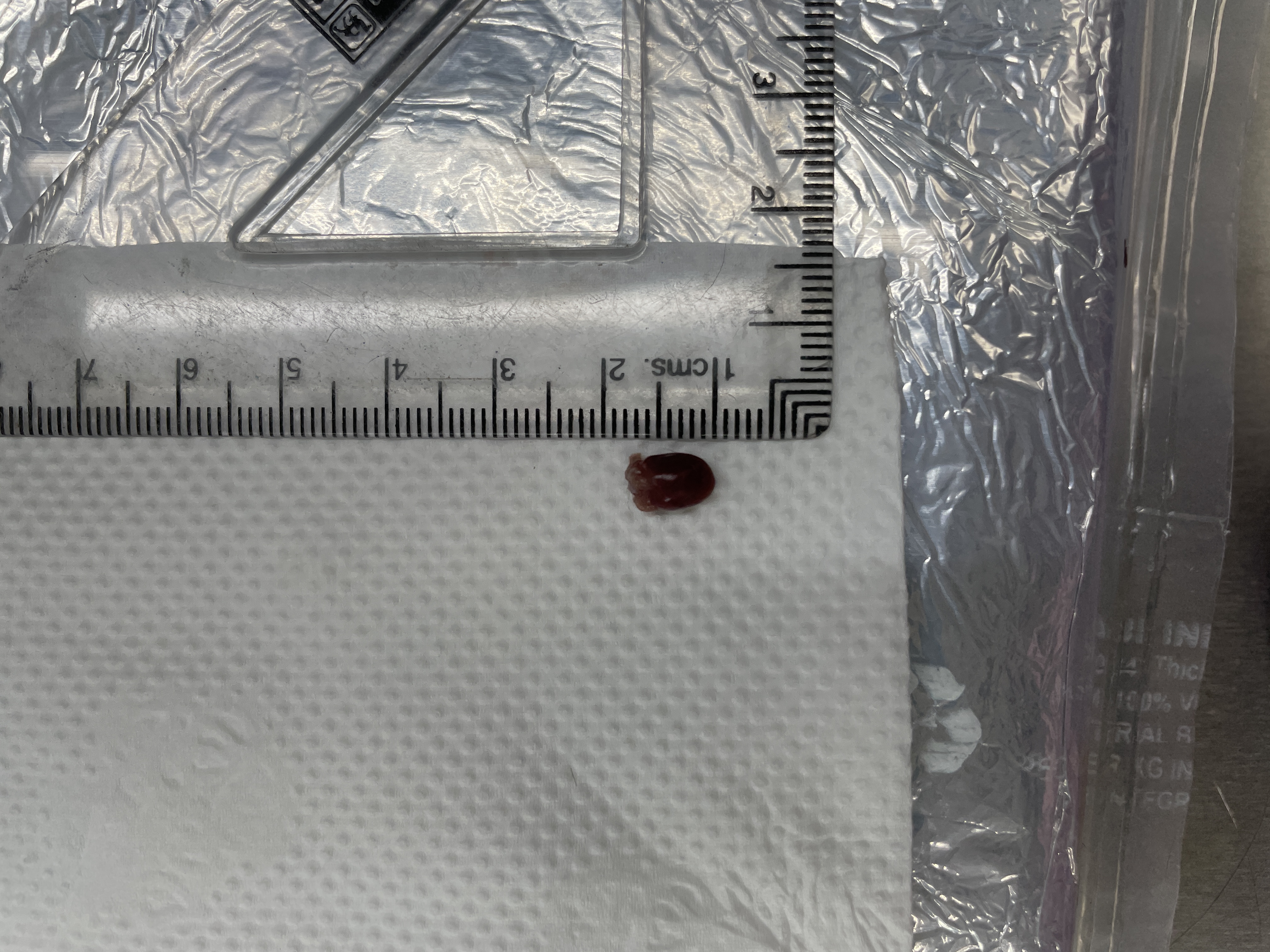

Supplement: Supplementary file 6 — Source data Fig. 6 [file 44319_2026_724_MOESM6_ESM.zip › Figure 6/Figure 6J/WT heart image.JPG]

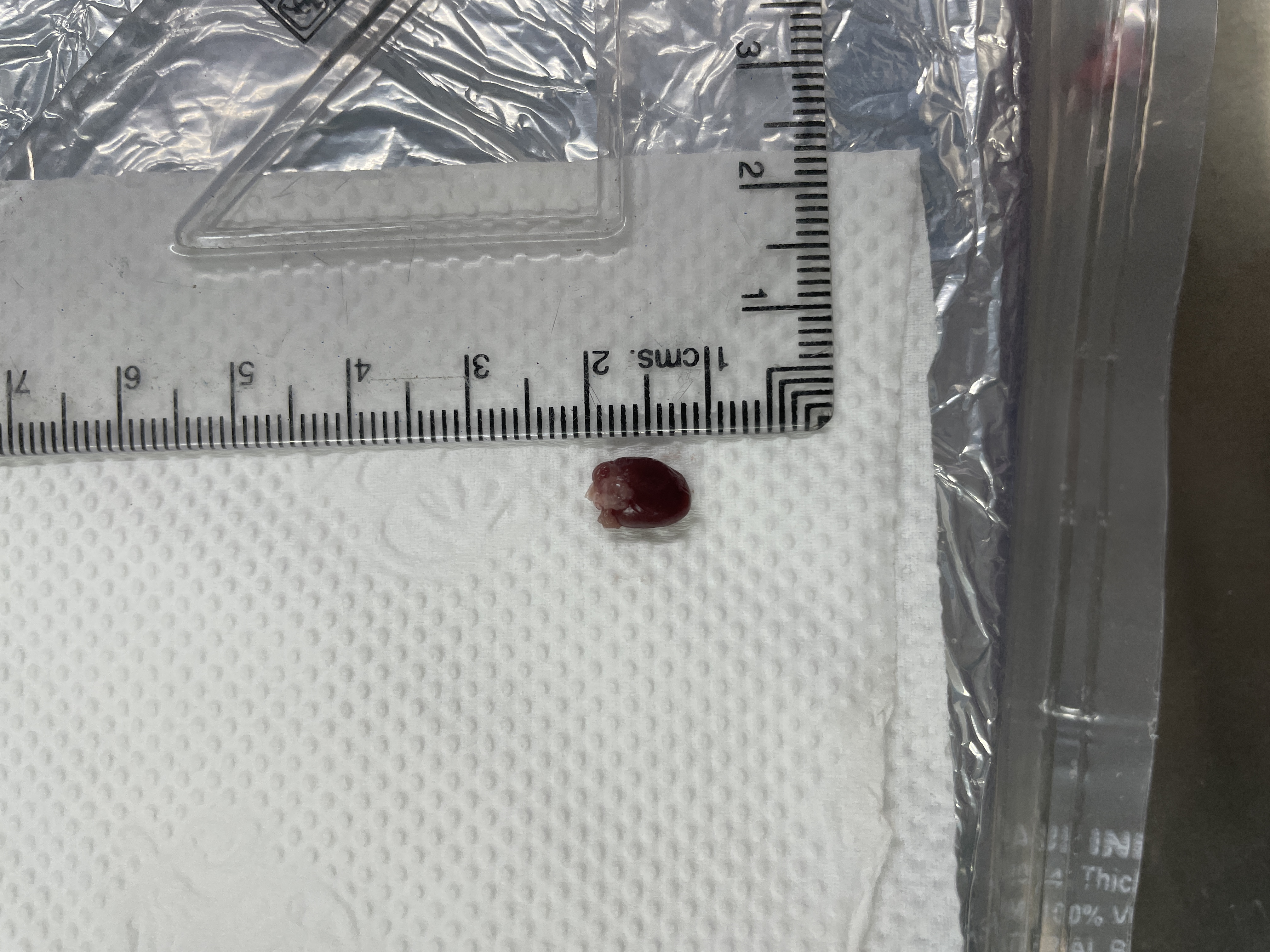

Supplement: Supplementary file 6 — Source data Fig. 6 [file 44319_2026_724_MOESM6_ESM.zip › Figure 6/Figure 6J/Sirt2-KO heart image.JPG]

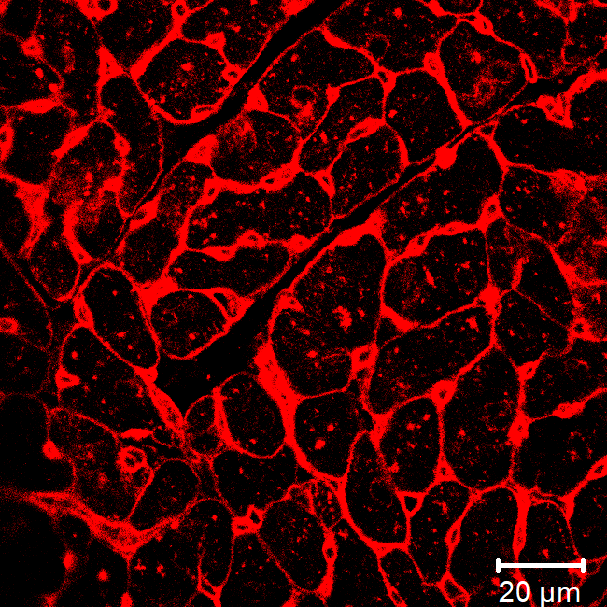

Supplement: Supplementary file 6 — Source data Fig. 6 [file 44319_2026_724_MOESM6_ESM.zip › Figure 6/Figure 6K/Sirt2-KO representative.tif]

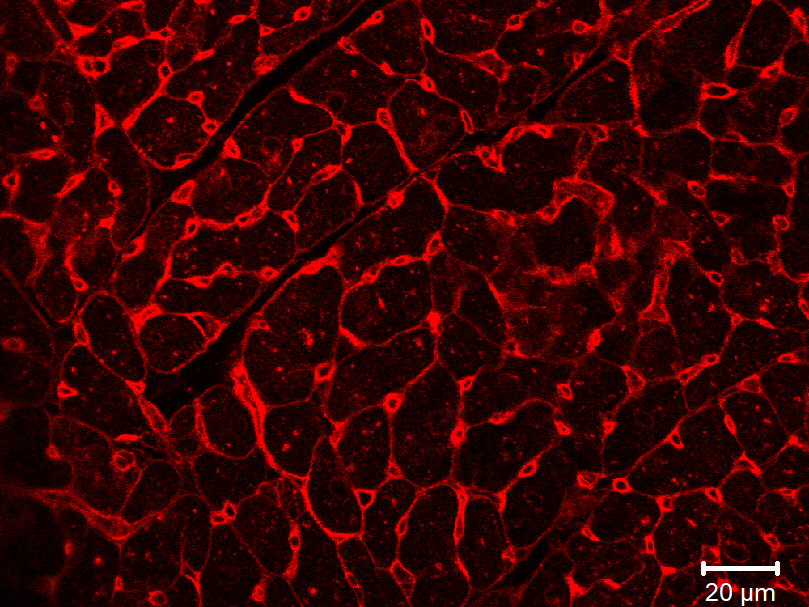

Supplement: Supplementary file 6 — Source data Fig. 6 [file 44319_2026_724_MOESM6_ESM.zip › Figure 6/Figure 6K/Sirt2-KO WGA.tif]

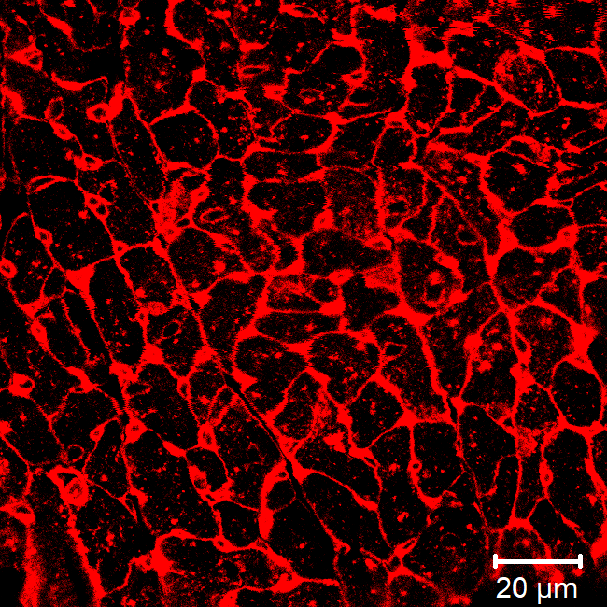

Supplement: Supplementary file 6 — Source data Fig. 6 [file 44319_2026_724_MOESM6_ESM.zip › Figure 6/Figure 6K/WT representative.tif]

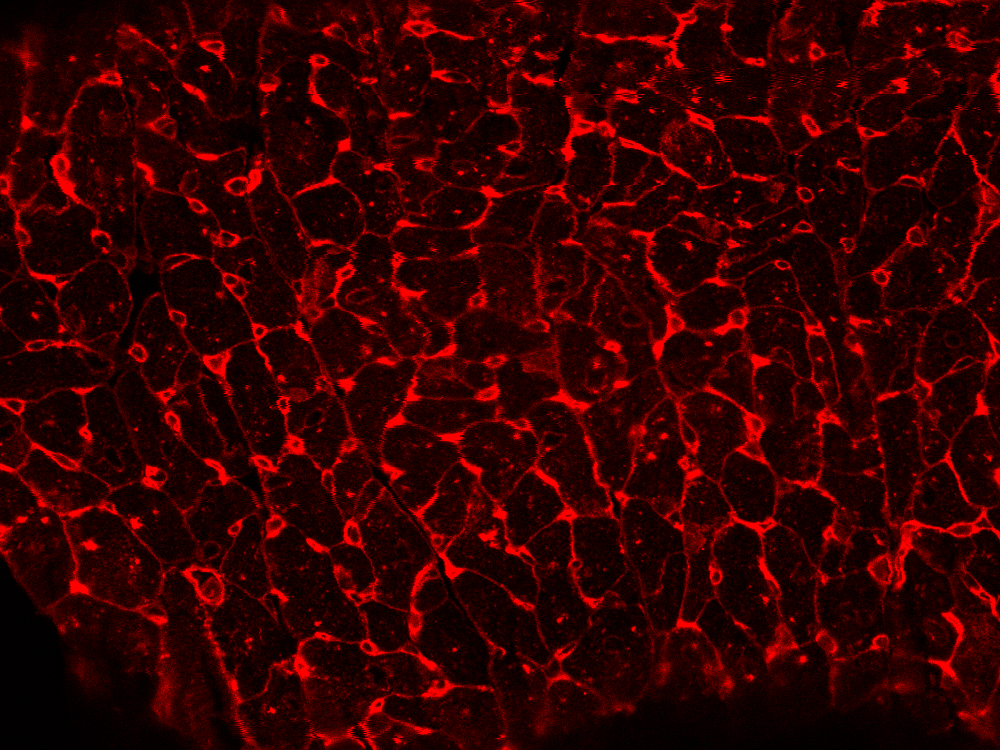

Supplement: Supplementary file 6 — Source data Fig. 6 [file 44319_2026_724_MOESM6_ESM.zip › Figure 6/Figure 6K/WT WGA.tiff]

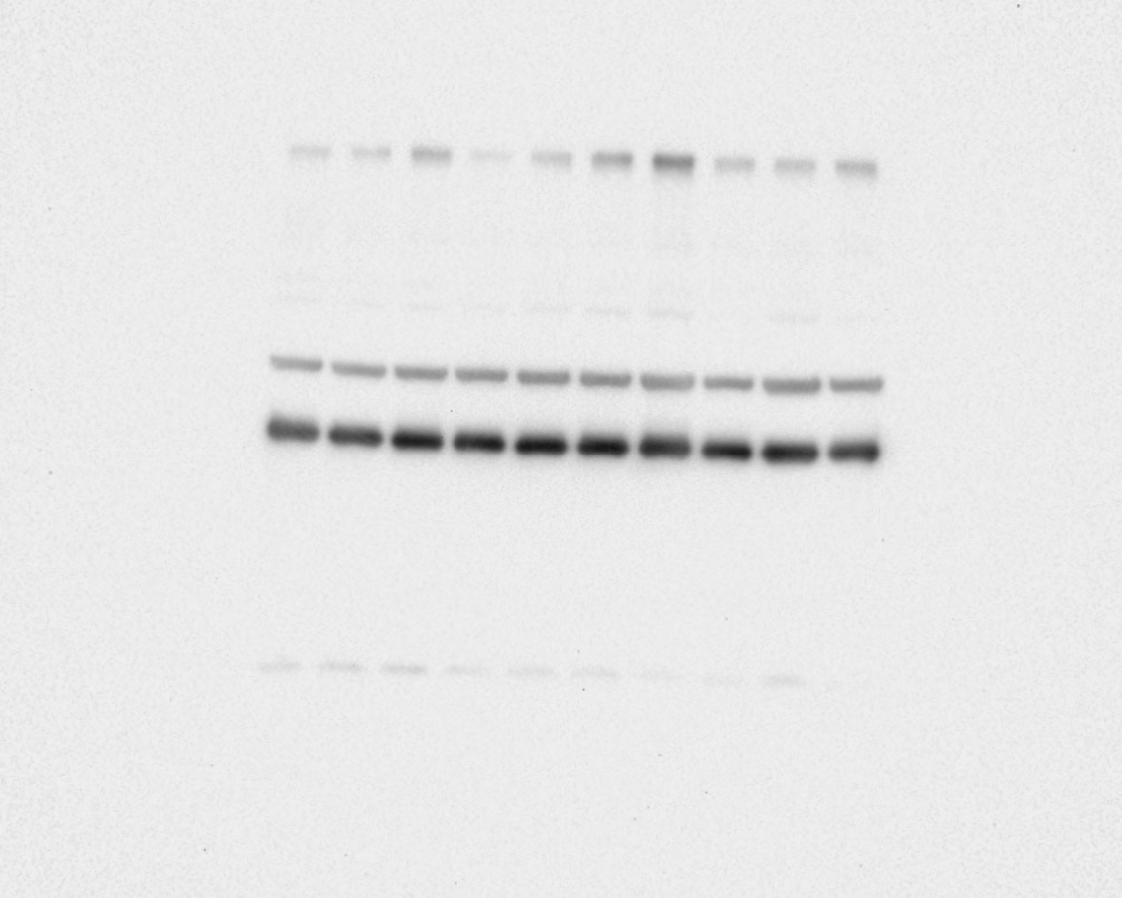

Supplement: Supplementary file 6 — Source data Fig. 6 [file 44319_2026_724_MOESM6_ESM.zip › Figure 6/Figure 6M/Figure 6M GAPDH.tif]

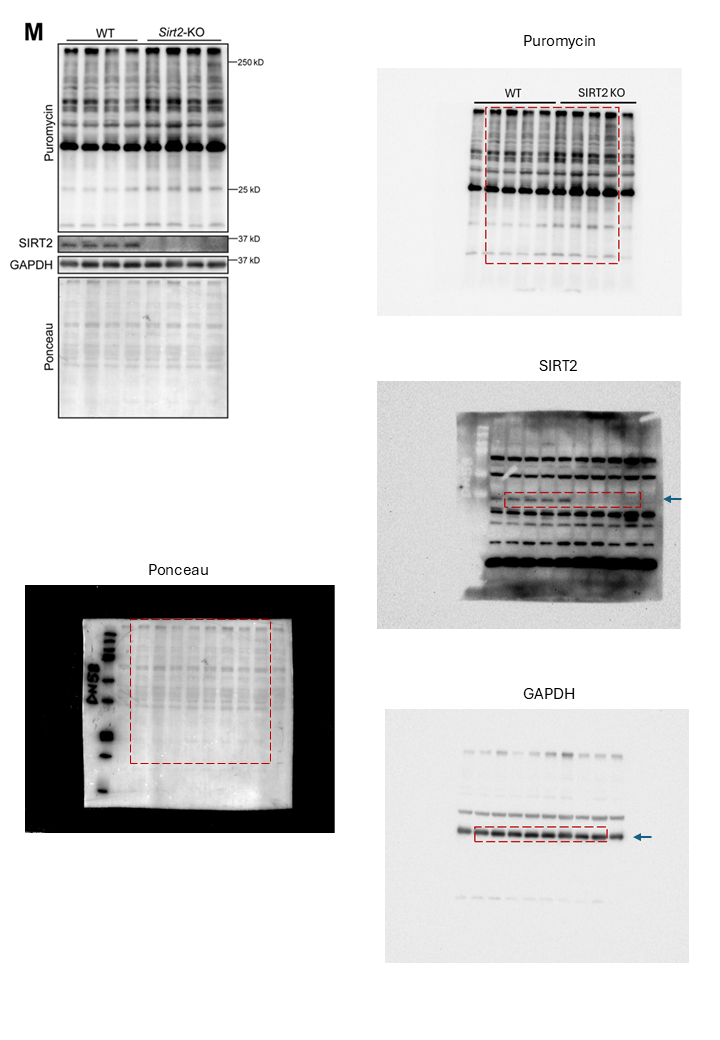

Supplement: Supplementary file 6 — Source data Fig. 6 [file 44319_2026_724_MOESM6_ESM.zip › Figure 6/Figure 6M/Figure 6M merged.tif]

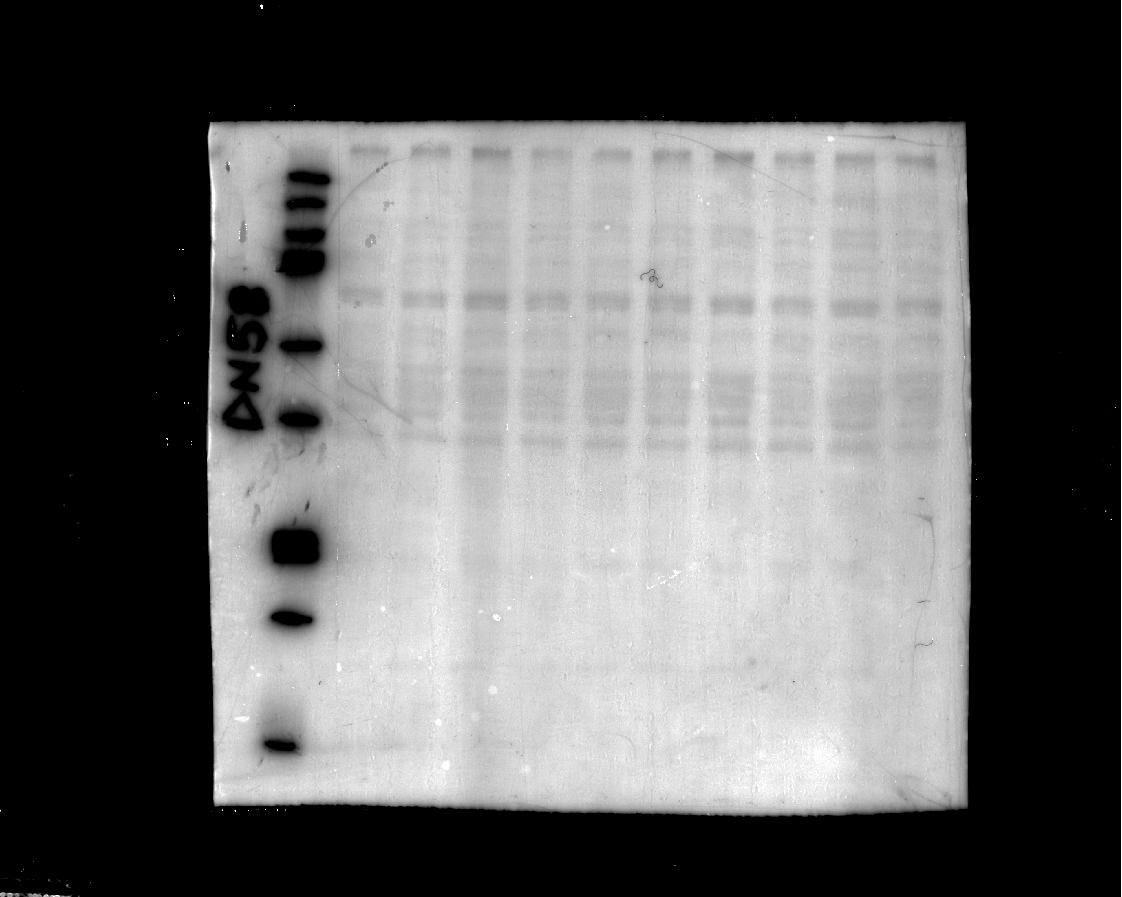

Supplement: Supplementary file 6 — Source data Fig. 6 [file 44319_2026_724_MOESM6_ESM.zip › Figure 6/Figure 6M/Figure 6M Ponceau.tif]

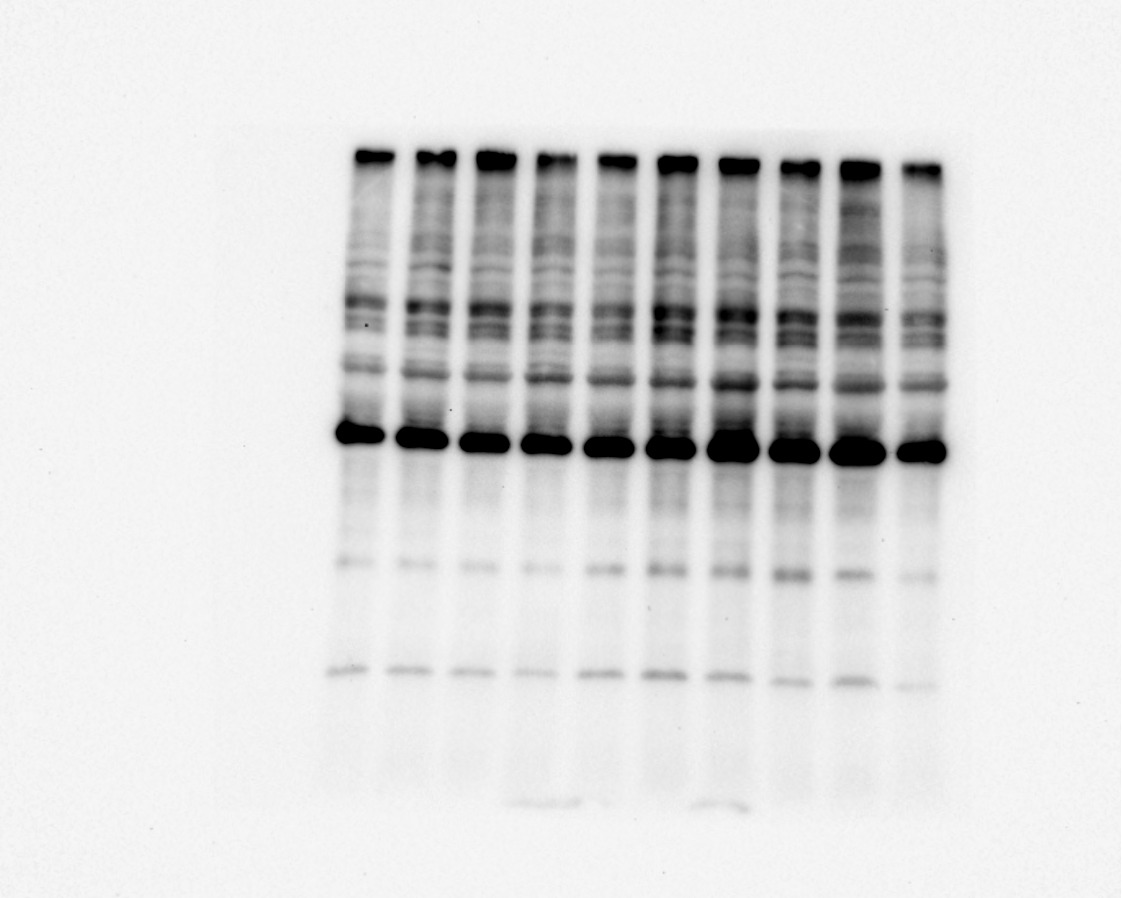

Supplement: Supplementary file 6 — Source data Fig. 6 [file 44319_2026_724_MOESM6_ESM.zip › Figure 6/Figure 6M/Figure 6M puromycin.tif]

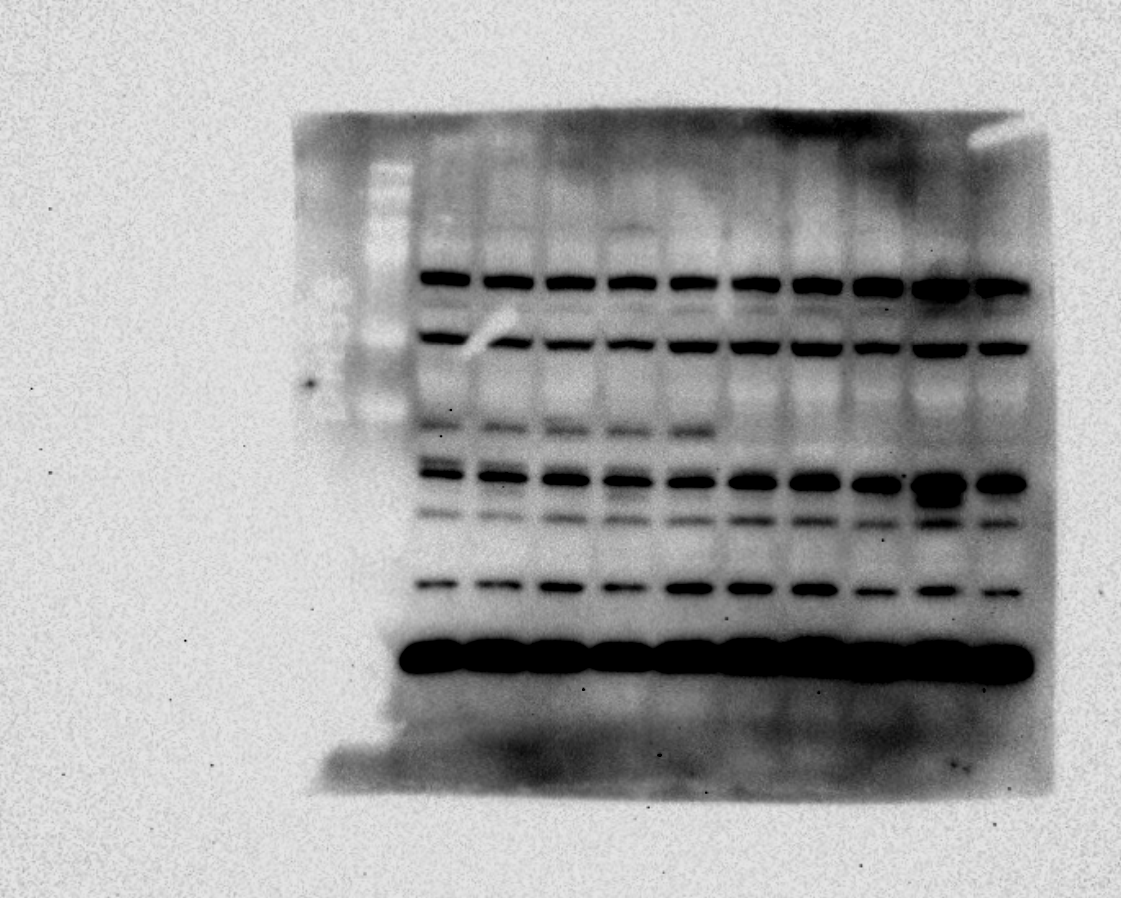

Supplement: Supplementary file 6 — Source data Fig. 6 [file 44319_2026_724_MOESM6_ESM.zip › Figure 6/Figure 6M/Figure 6M SIRT2.tif]

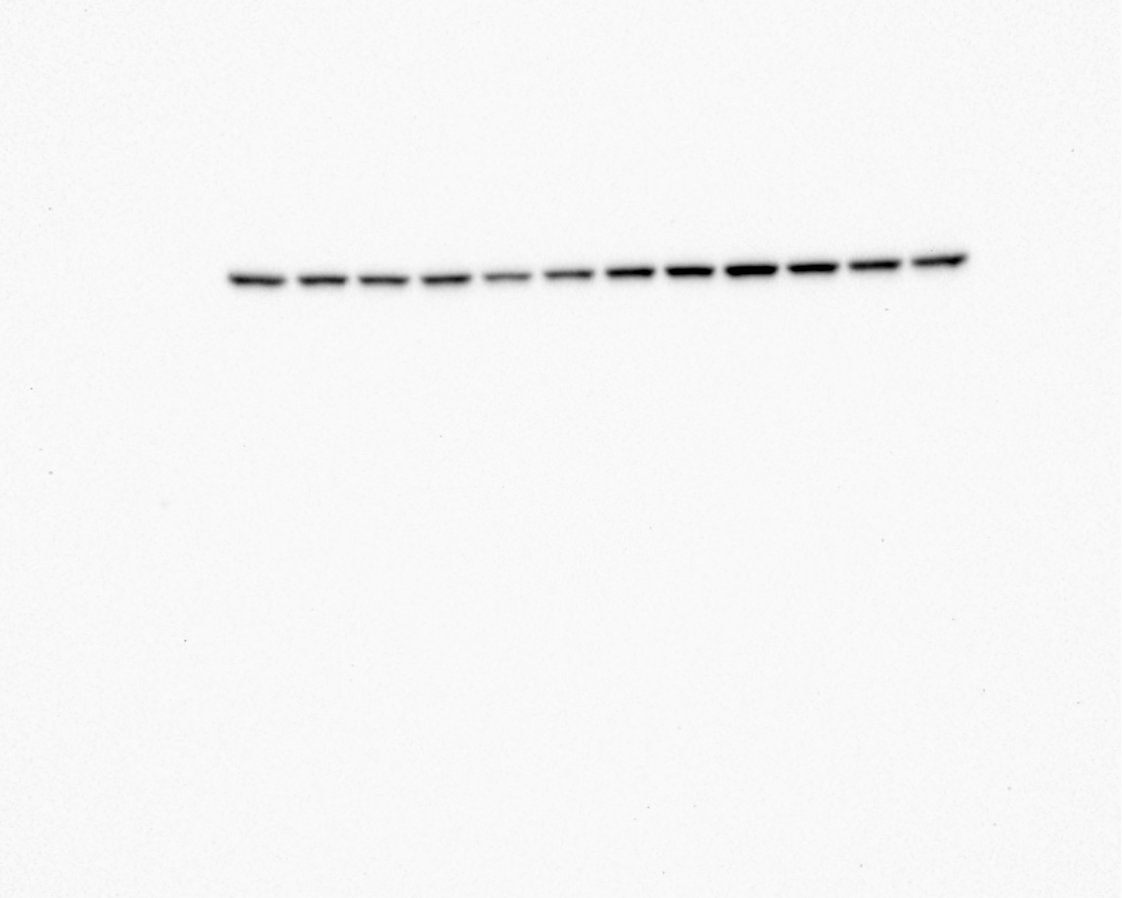

Supplement: Supplementary file 7 — Source data Fig. 7 [file 44319_2026_724_MOESM7_ESM.zip › Figure 7/Figure 7A/Figure 7A acetyl alpha-tubulin.tif]

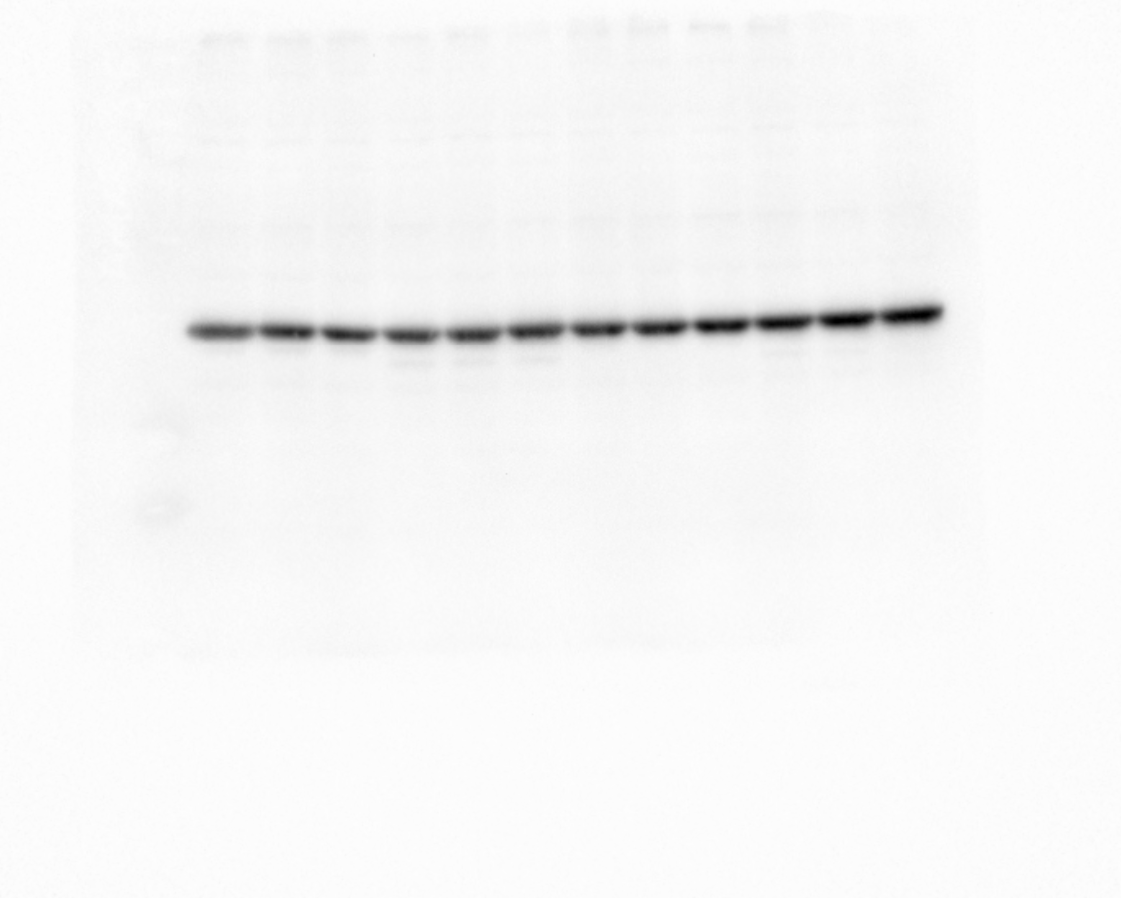

Supplement: Supplementary file 7 — Source data Fig. 7 [file 44319_2026_724_MOESM7_ESM.zip › Figure 7/Figure 7A/Figure 7A GAPDH.tif]

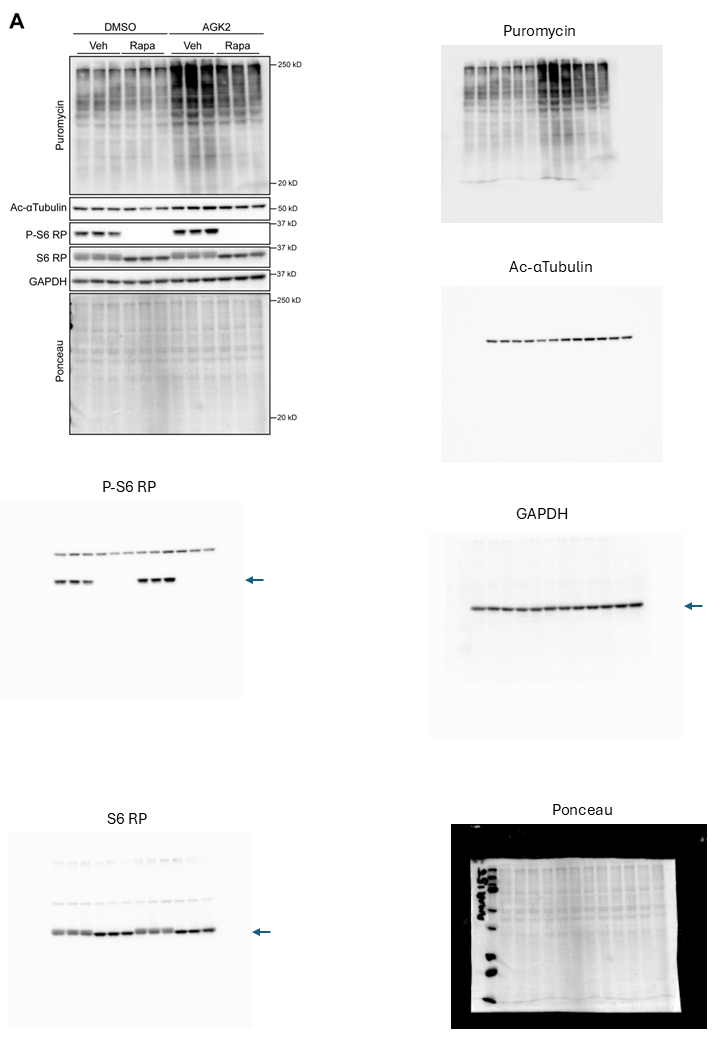

Supplement: Supplementary file 7 — Source data Fig. 7 [file 44319_2026_724_MOESM7_ESM.zip › Figure 7/Figure 7A/Figure 7A merged data.tif]

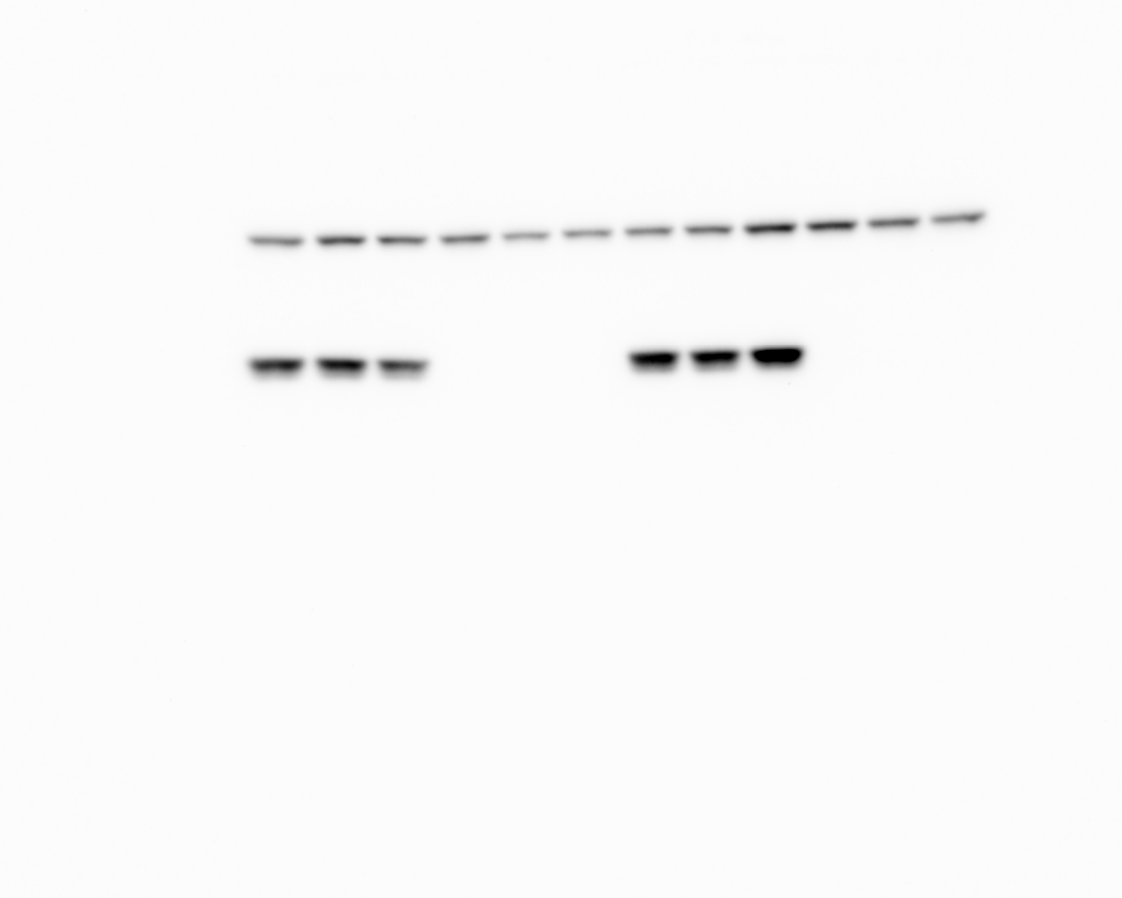

Supplement: Supplementary file 7 — Source data Fig. 7 [file 44319_2026_724_MOESM7_ESM.zip › Figure 7/Figure 7A/Figure 7A P-S6 RP.tif]

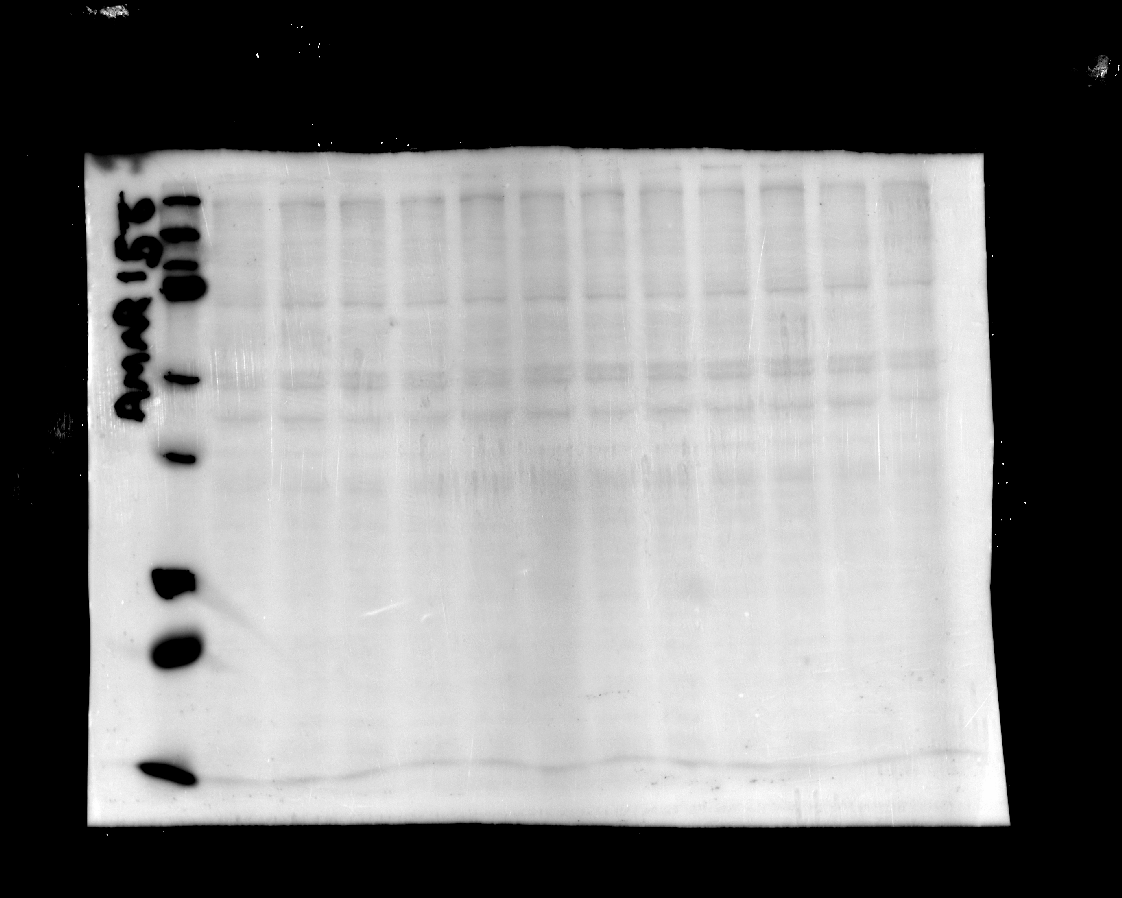

Supplement: Supplementary file 7 — Source data Fig. 7 [file 44319_2026_724_MOESM7_ESM.zip › Figure 7/Figure 7A/Figure 7A Ponceau.tif]

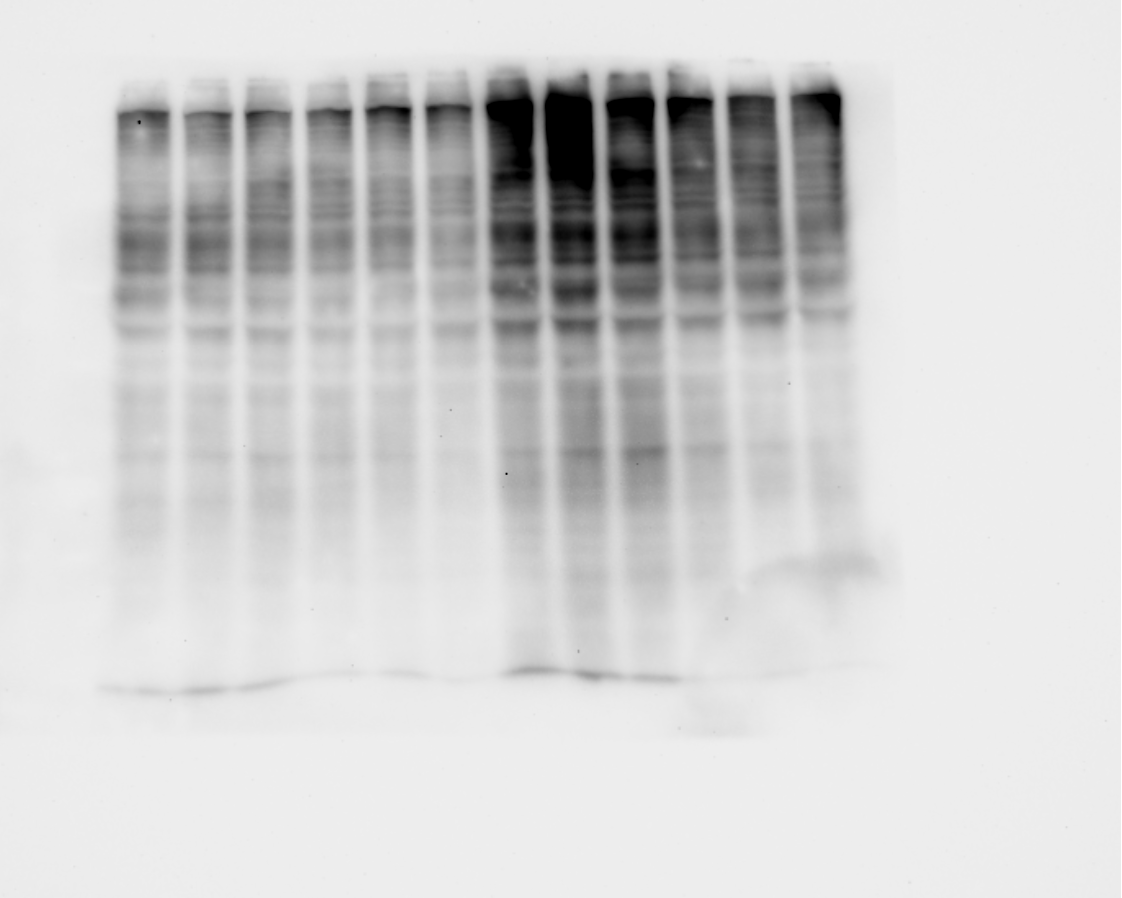

Supplement: Supplementary file 7 — Source data Fig. 7 [file 44319_2026_724_MOESM7_ESM.zip › Figure 7/Figure 7A/Figure 7A Puromycin.tif]

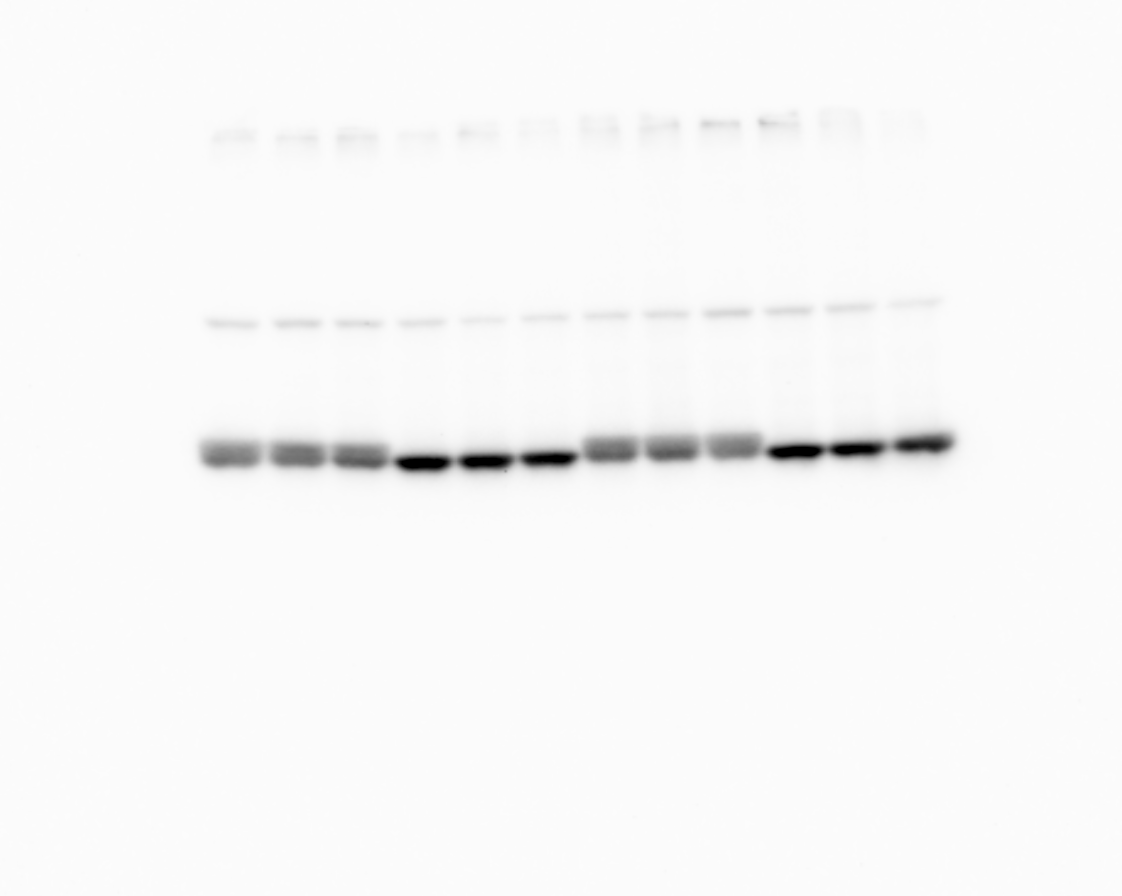

Supplement: Supplementary file 7 — Source data Fig. 7 [file 44319_2026_724_MOESM7_ESM.zip › Figure 7/Figure 7A/Figure 7A S6 RP.tif]

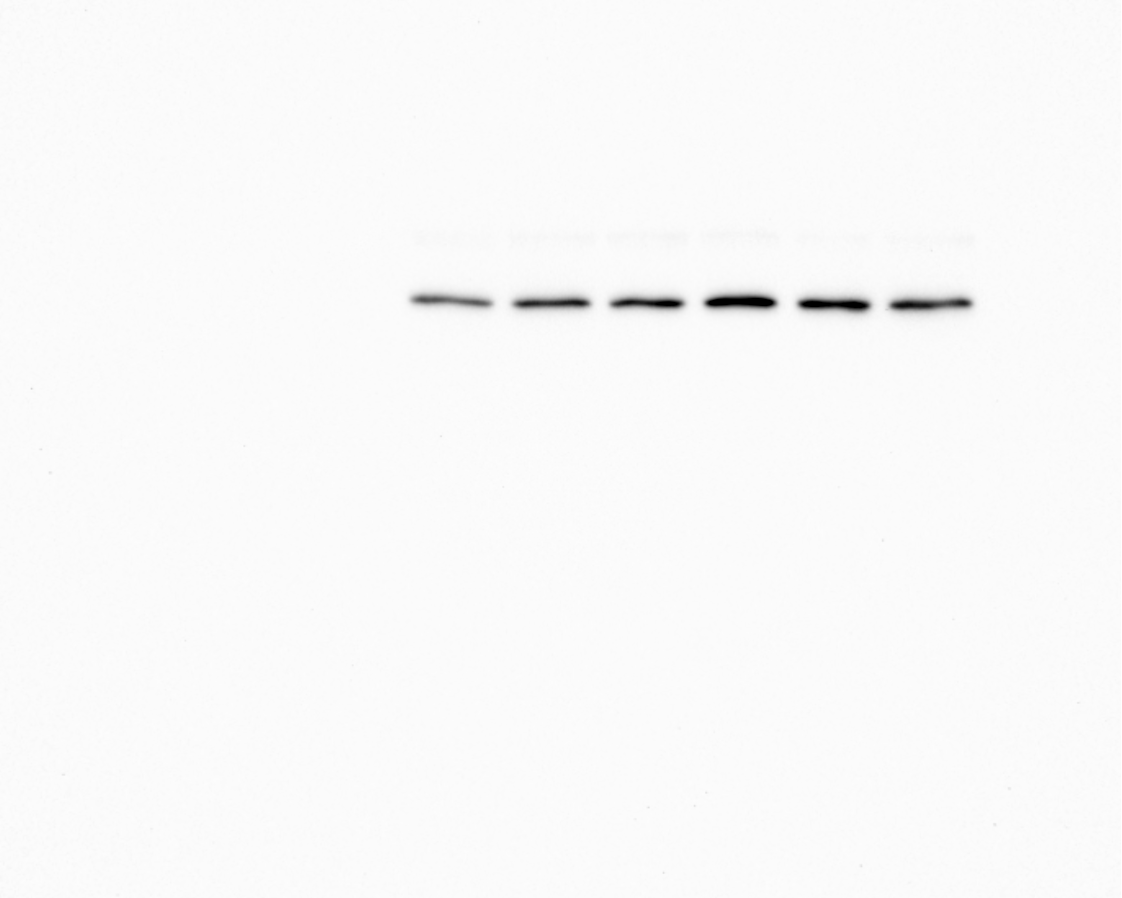

Supplement: Supplementary file 7 — Source data Fig. 7 [file 44319_2026_724_MOESM7_ESM.zip › Figure 7/Figure 7D/Figure 7D Acetyl alpha tubulin.tif]

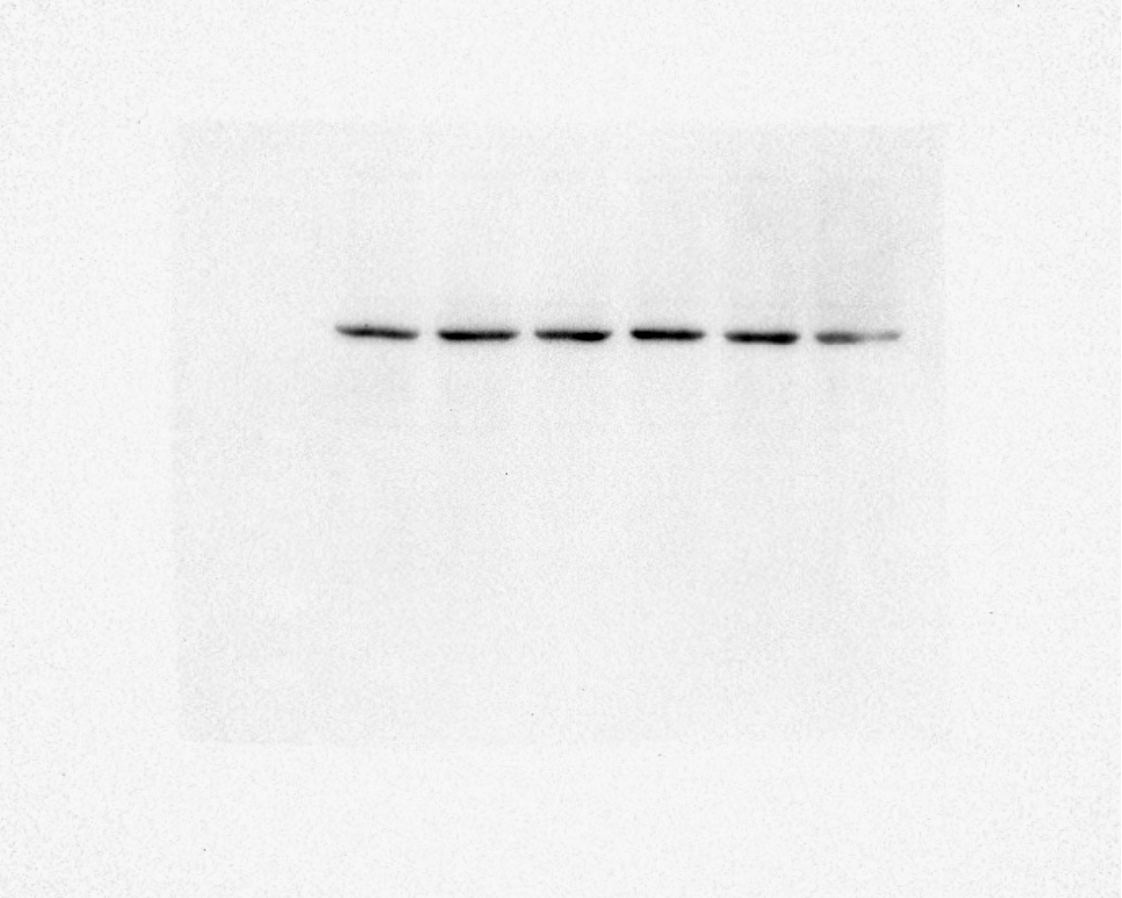

Supplement: Supplementary file 7 — Source data Fig. 7 [file 44319_2026_724_MOESM7_ESM.zip › Figure 7/Figure 7D/Figure 7D actin.tif]

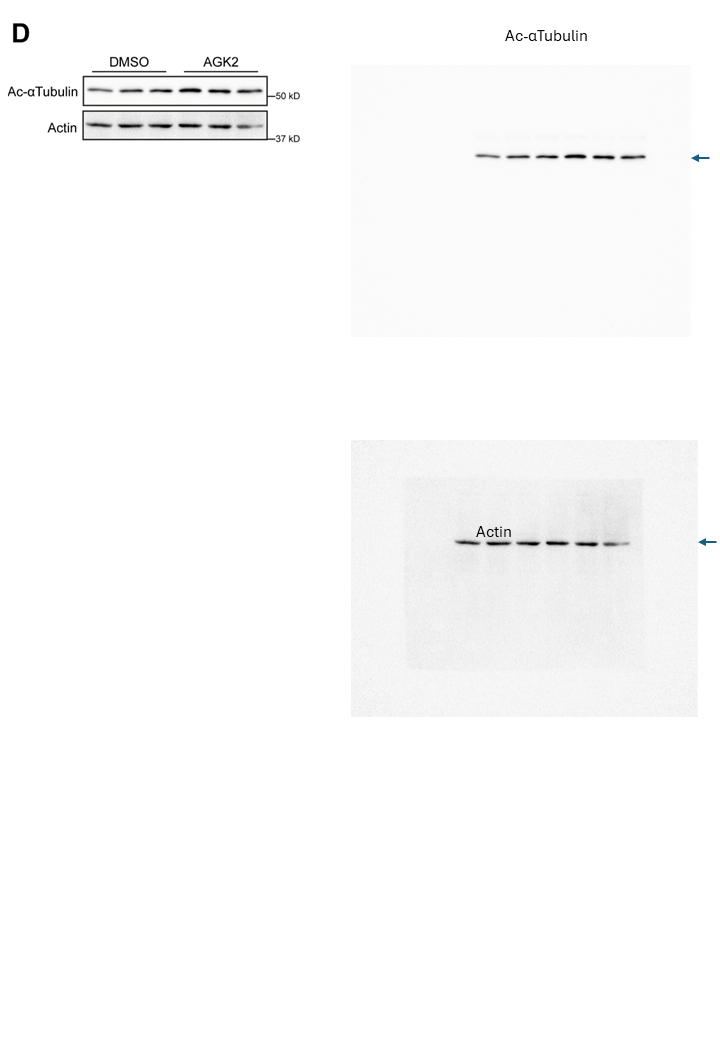

Supplement: Supplementary file 7 — Source data Fig. 7 [file 44319_2026_724_MOESM7_ESM.zip › Figure 7/Figure 7D/Figure 7D merged.tif]

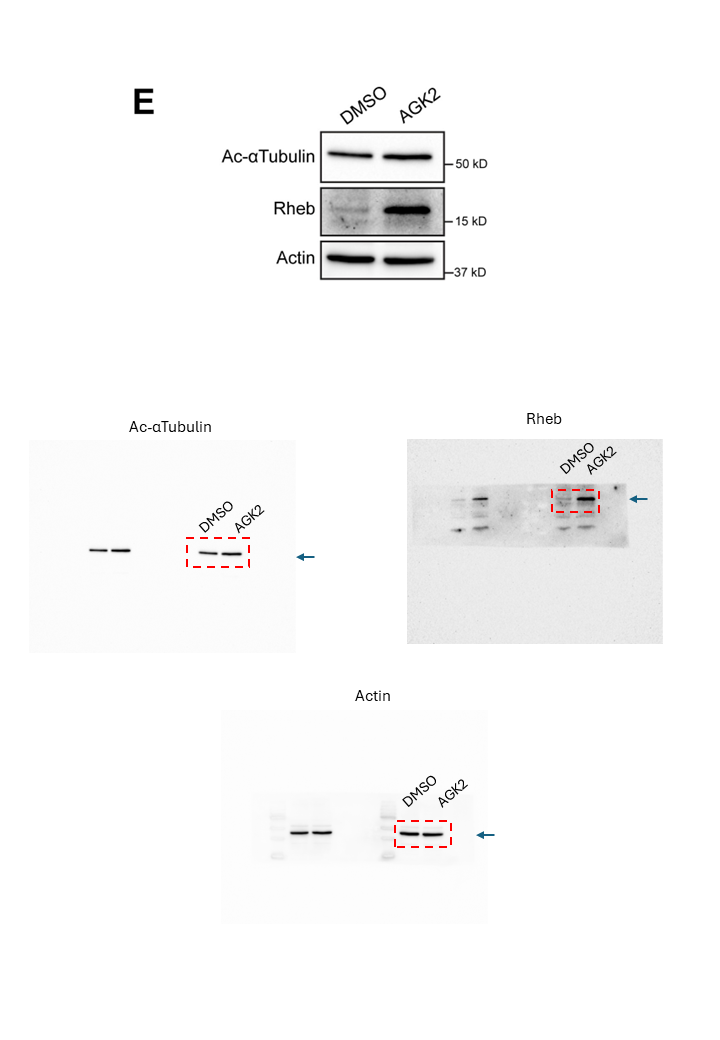

Supplement: Supplementary file 7 — Source data Fig. 7 [file 44319_2026_724_MOESM7_ESM.zip › Figure 7/Figure 7E/Figure 7E merged.tif]

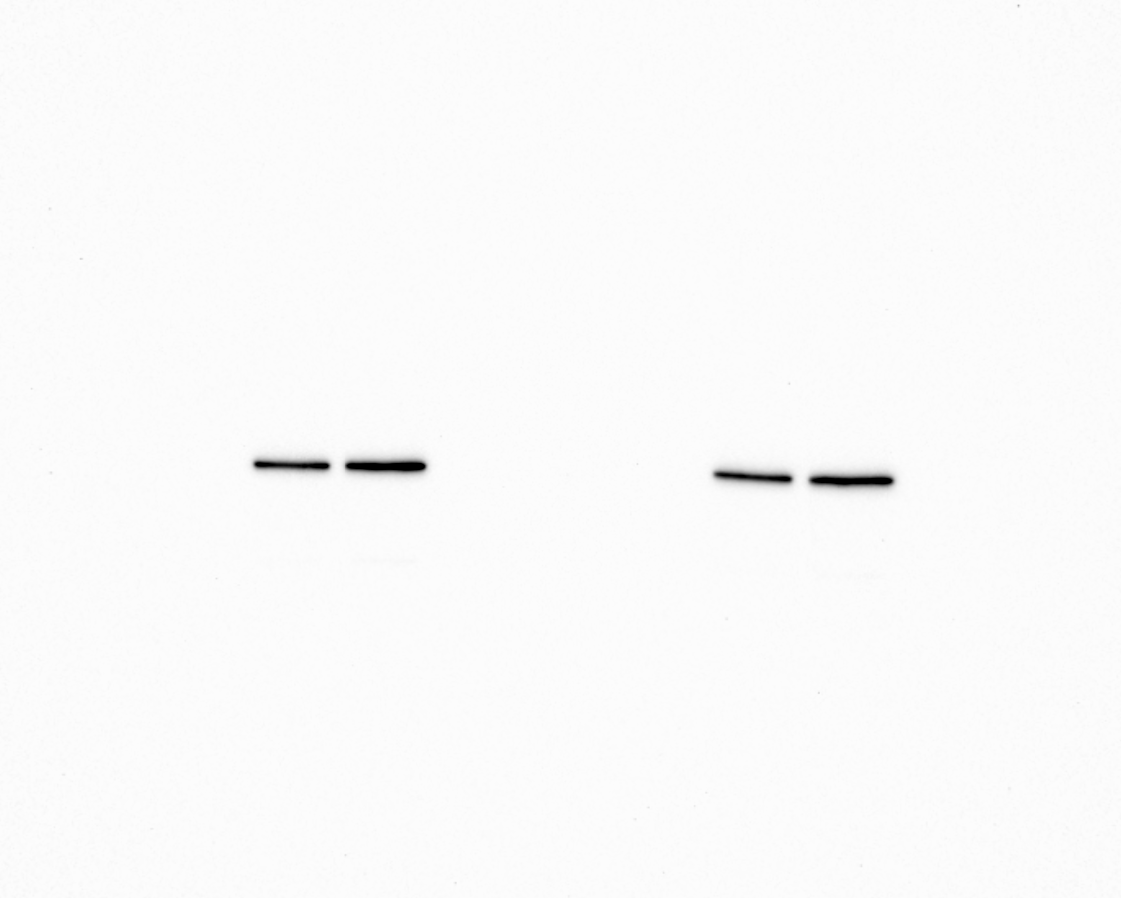

Supplement: Supplementary file 7 — Source data Fig. 7 [file 44319_2026_724_MOESM7_ESM.zip › Figure 7/Figure 7E/Figure 7E acetyl alpha tubulin.tif]

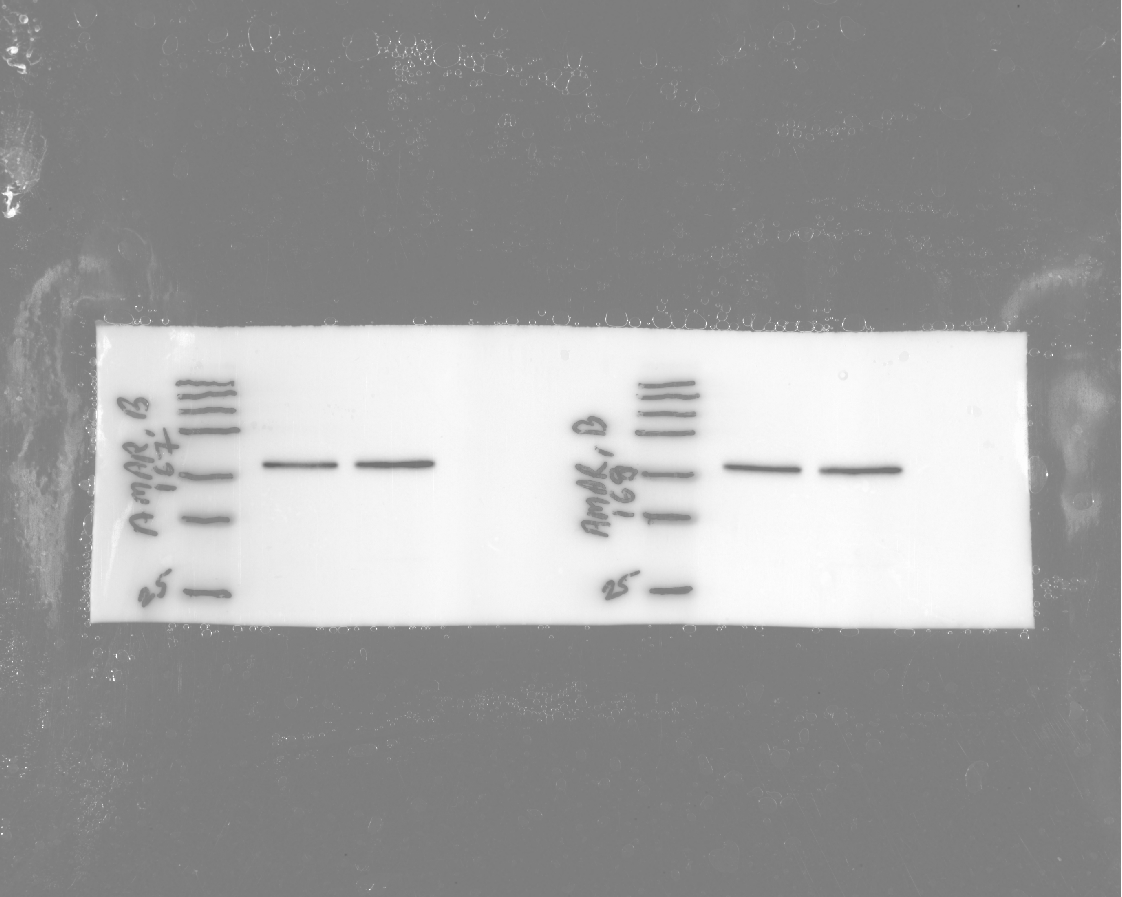

Supplement: Supplementary file 7 — Source data Fig. 7 [file 44319_2026_724_MOESM7_ESM.zip › Figure 7/Figure 7E/Figure 7E acetyl alpha tubulin (Composite).tif]

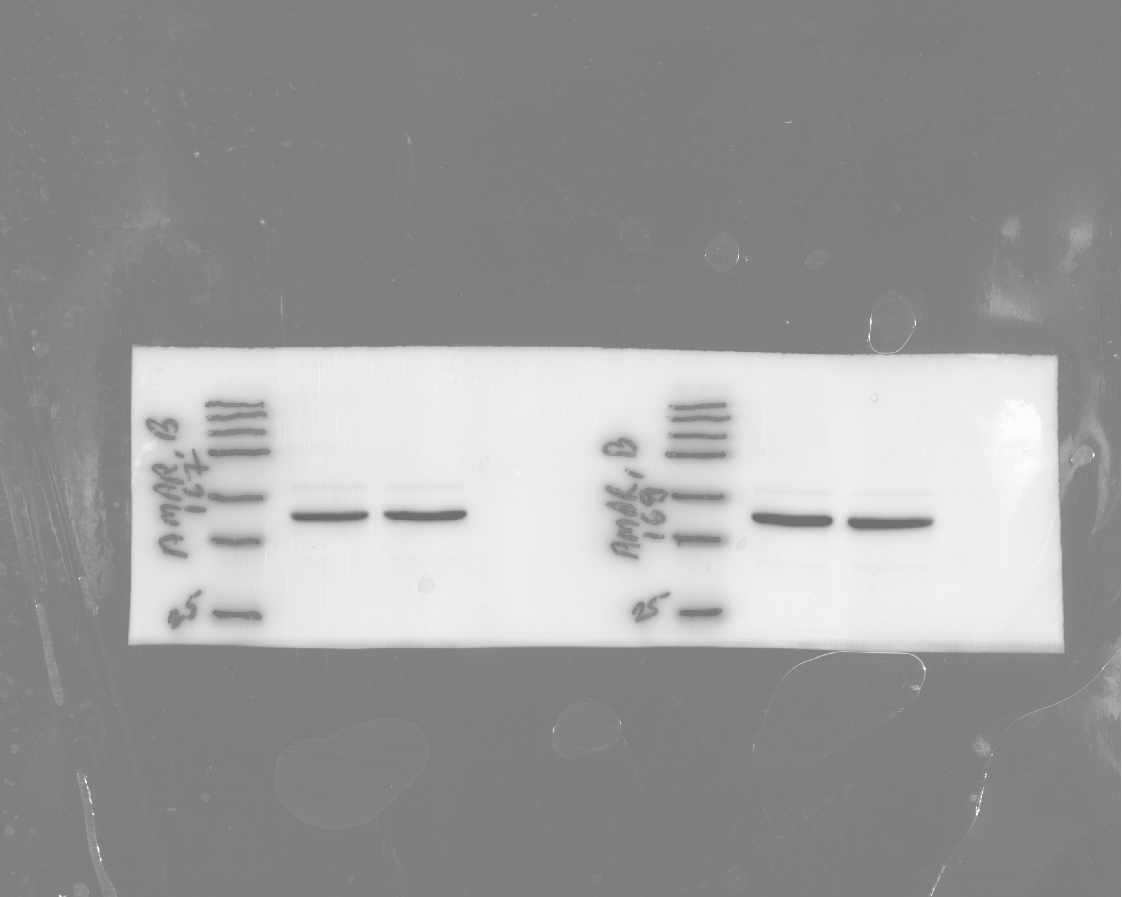

Supplement: Supplementary file 7 — Source data Fig. 7 [file 44319_2026_724_MOESM7_ESM.zip › Figure 7/Figure 7E/Figure 7E Actin (Composite).tif]

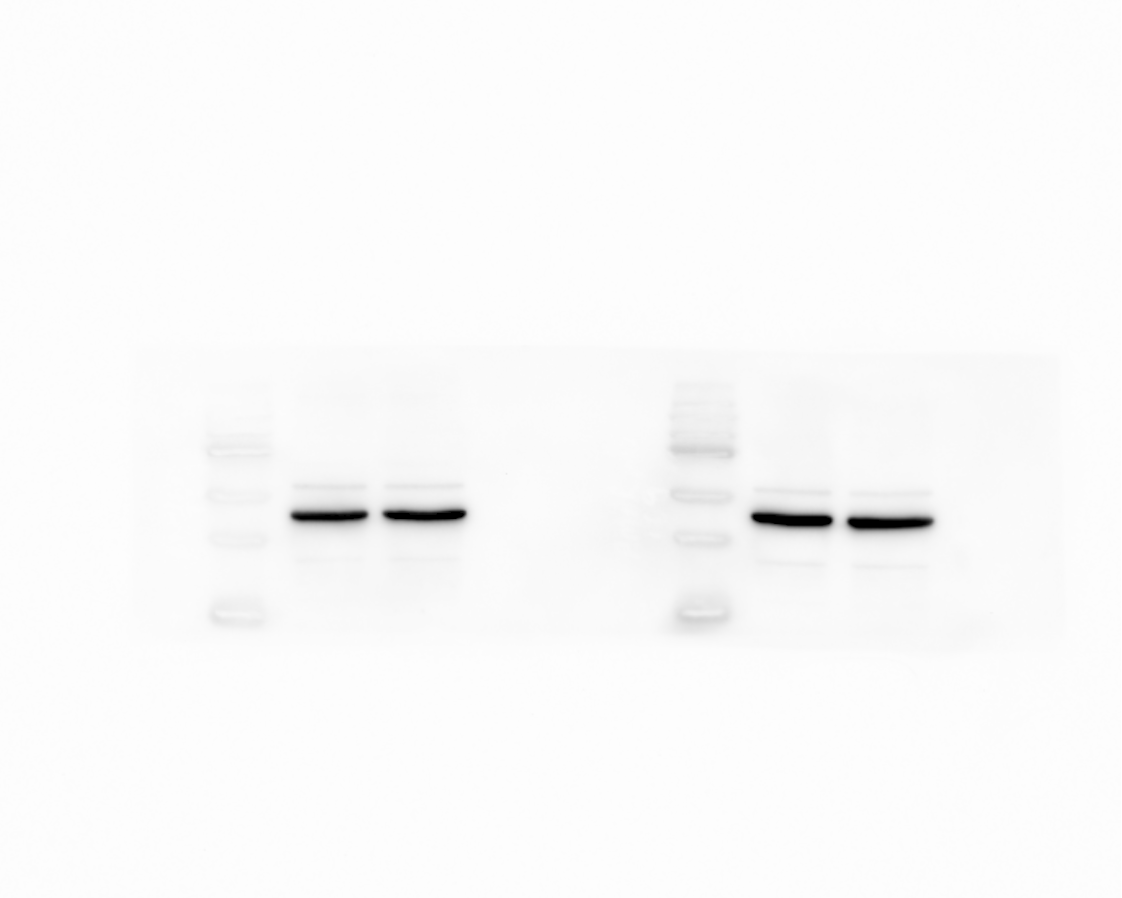

Supplement: Supplementary file 7 — Source data Fig. 7 [file 44319_2026_724_MOESM7_ESM.zip › Figure 7/Figure 7E/Figure 7E Actin.tif]

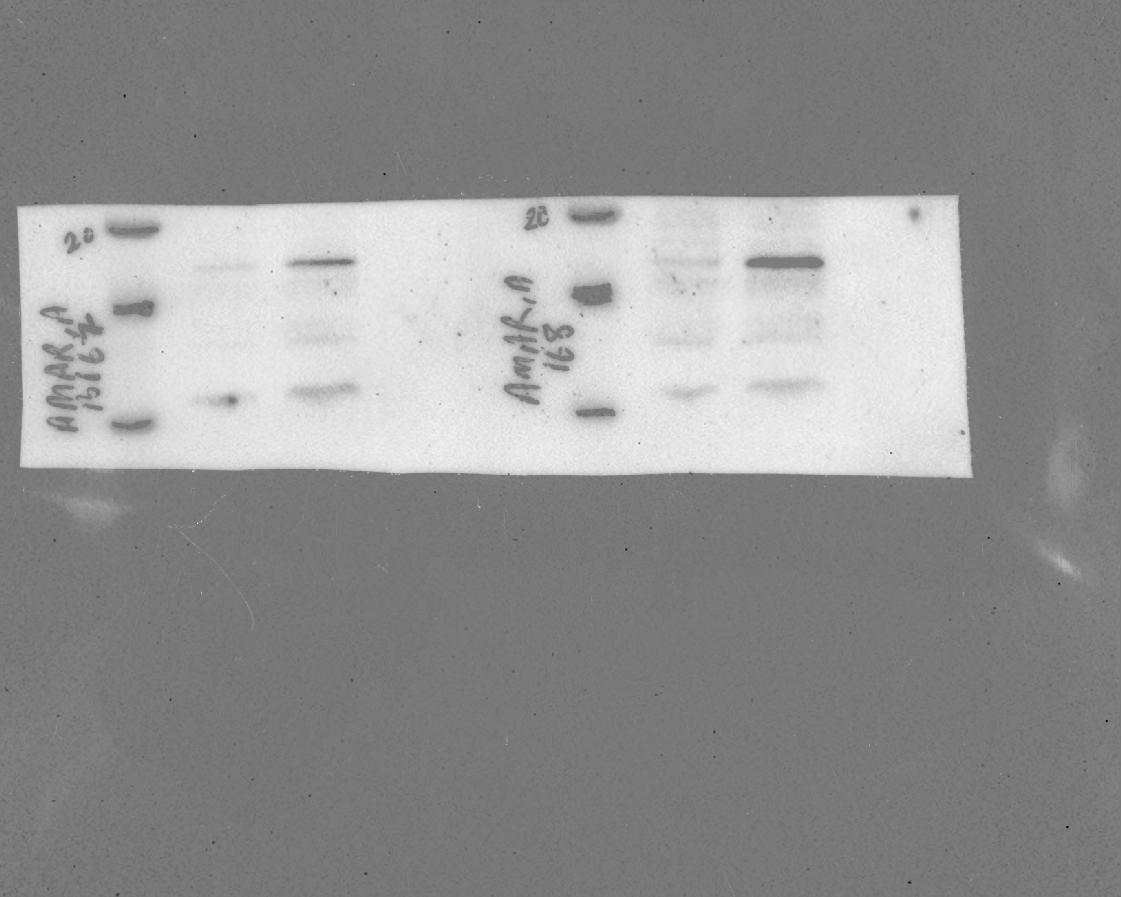

Supplement: Supplementary file 7 — Source data Fig. 7 [file 44319_2026_724_MOESM7_ESM.zip › Figure 7/Figure 7E/Figure 7E Rheb (Composite).tif]

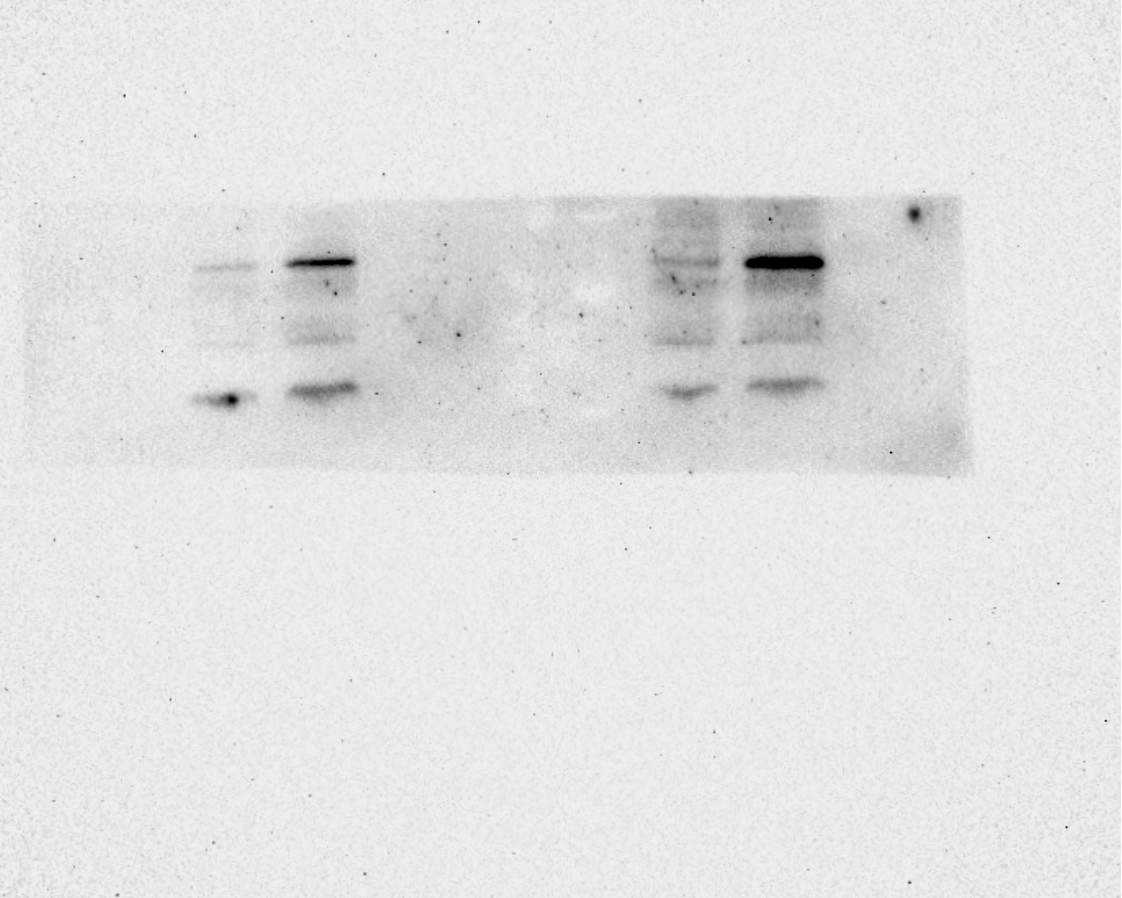

Supplement: Supplementary file 7 — Source data Fig. 7 [file 44319_2026_724_MOESM7_ESM.zip › Figure 7/Figure 7E/Figure 7E Rheb.tif]

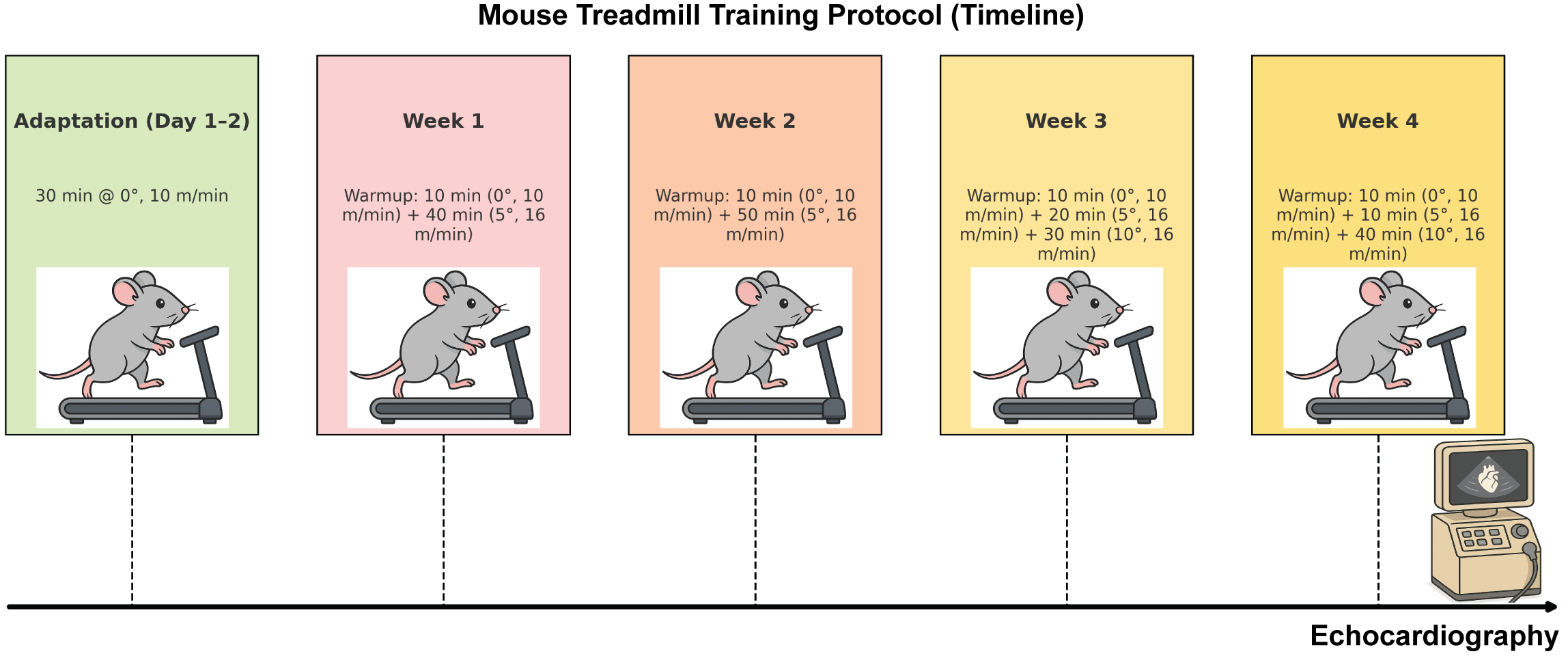

Supplement: Supplementary file 8 — Source data Fig. 8 [file 44319_2026_724_MOESM8_ESM.zip › Figure 8/Figure 8A.tif]

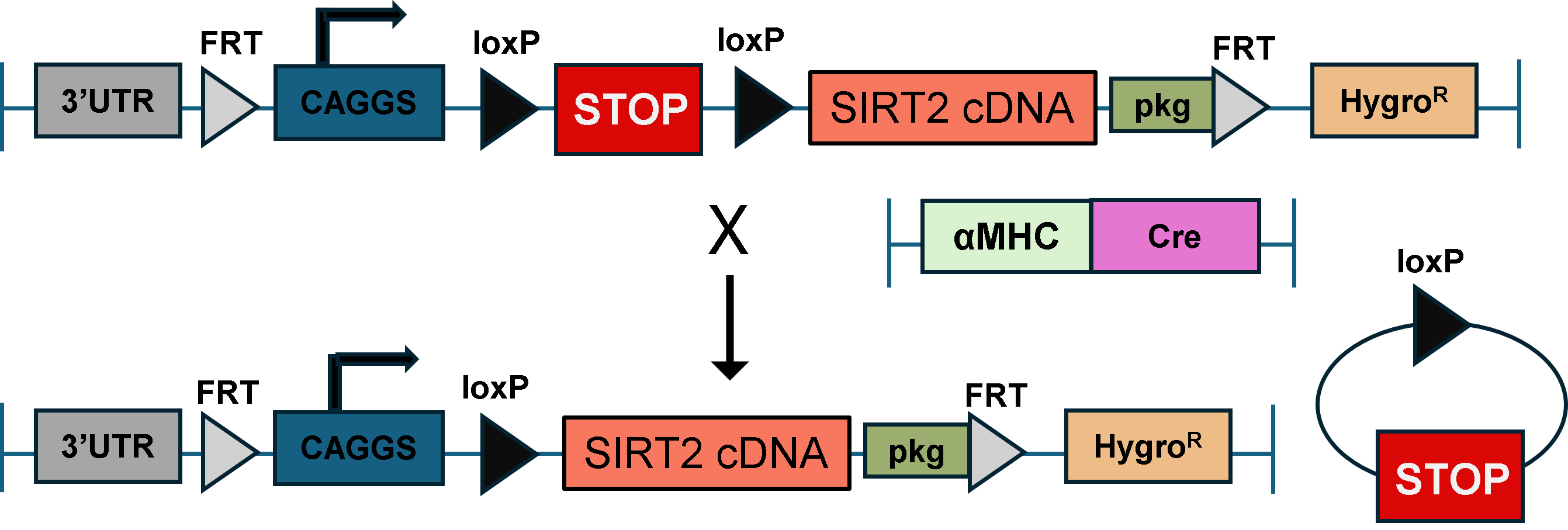

Supplement: Supplementary file 9 — Source data Fig. 9 [file 44319_2026_724_MOESM9_ESM.zip › Figure 9/Figure 9A/Figure 9A.tif]

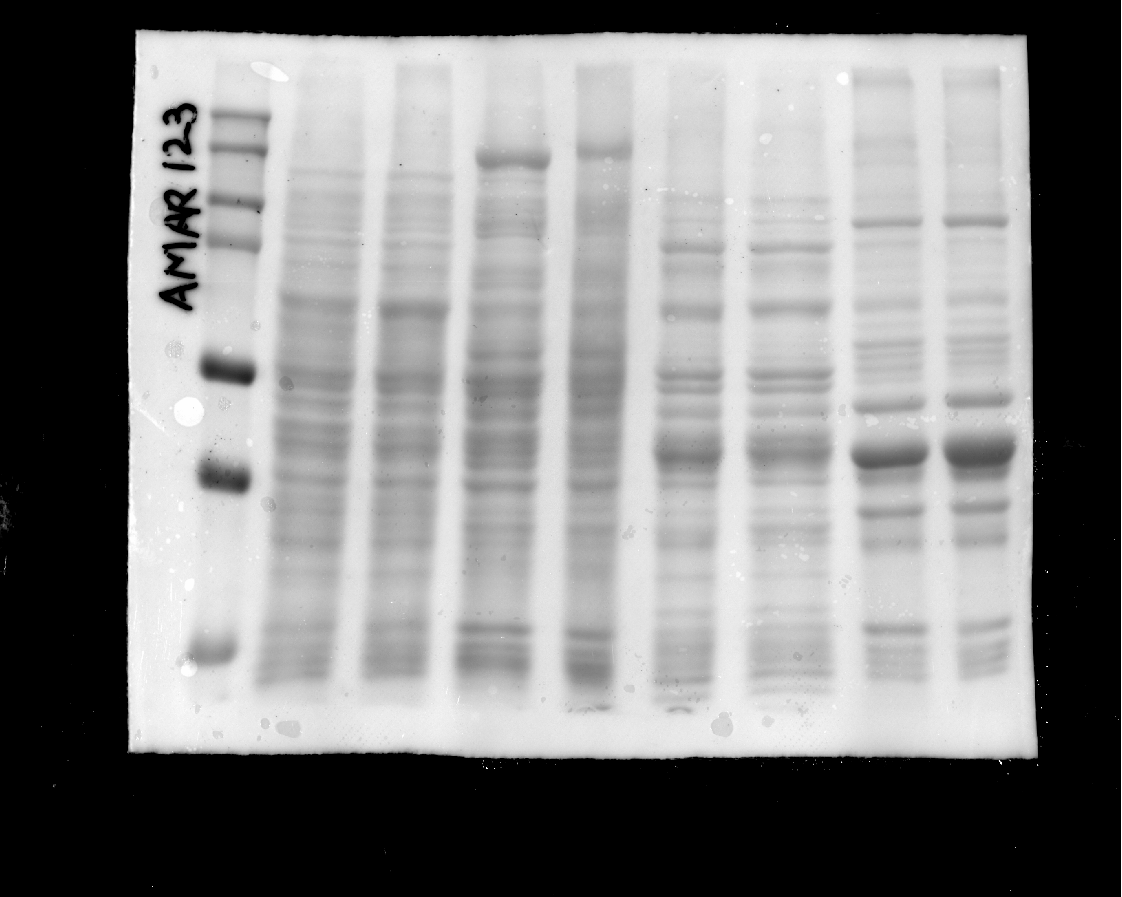

Supplement: Supplementary file 9 — Source data Fig. 9 [file 44319_2026_724_MOESM9_ESM.zip › Figure 9/Figure 9B/Figure 9B Ponceau.tif]

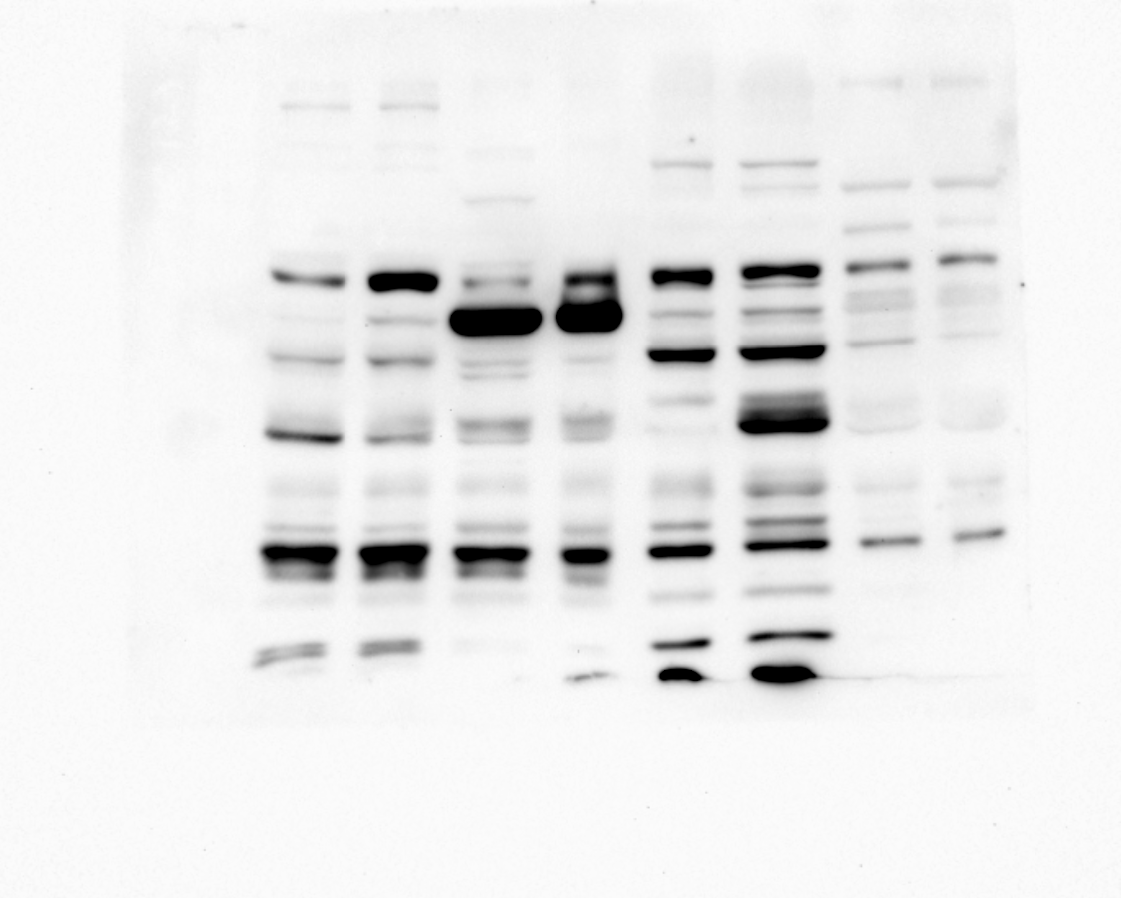

Supplement: Supplementary file 9 — Source data Fig. 9 [file 44319_2026_724_MOESM9_ESM.zip › Figure 9/Figure 9B/Figure 9B SIRT2.tif]

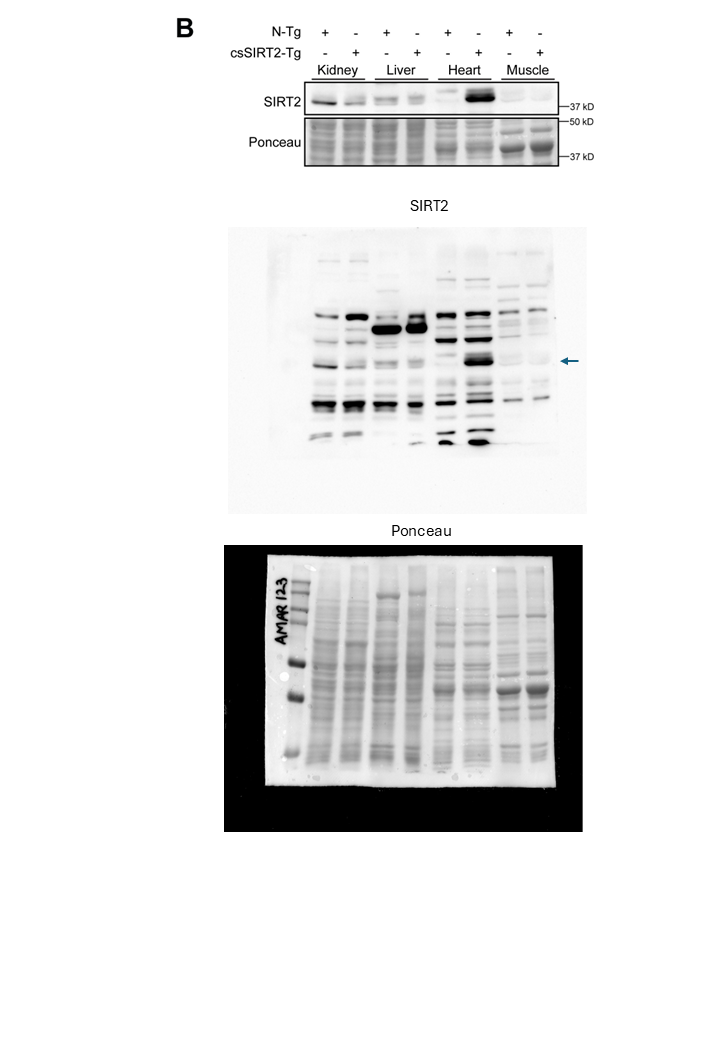

Supplement: Supplementary file 9 — Source data Fig. 9 [file 44319_2026_724_MOESM9_ESM.zip › Figure 9/Figure 9B/Figure 9B.tif]

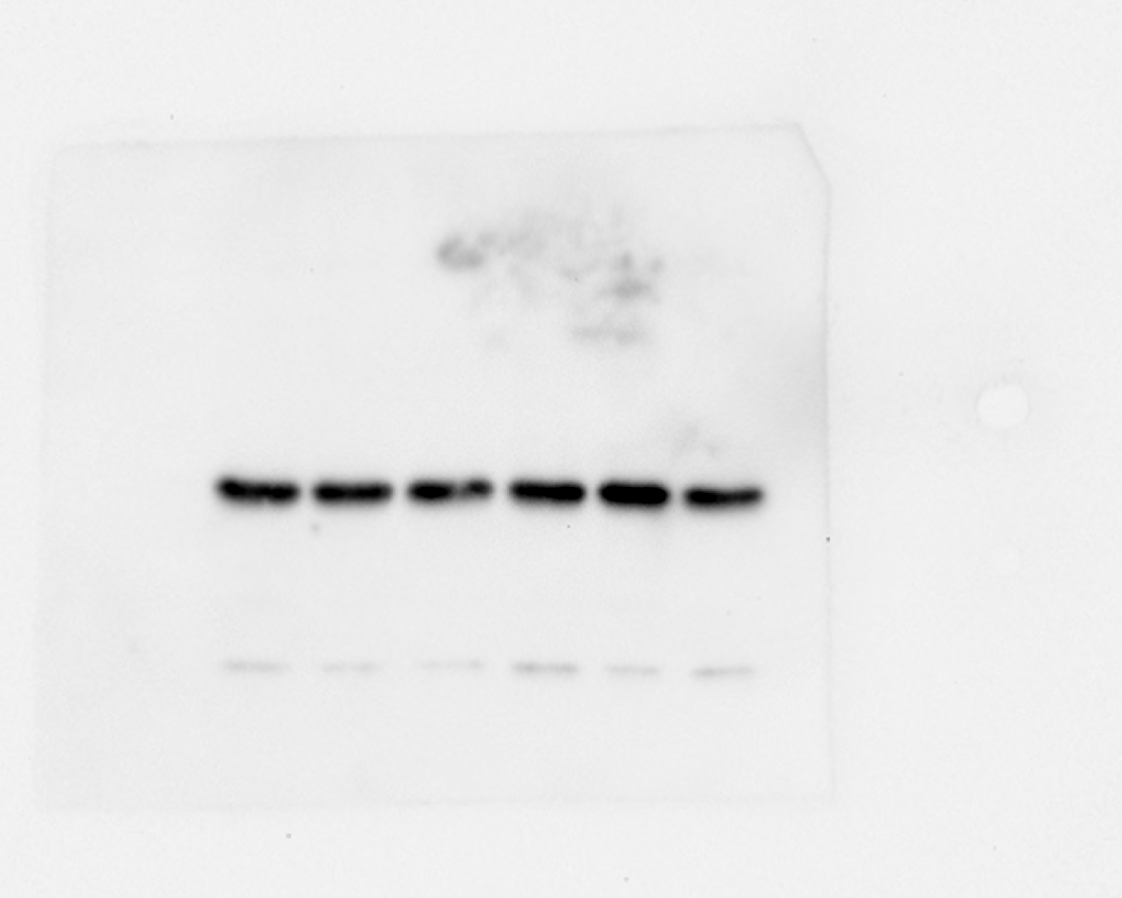

Supplement: Supplementary file 9 — Source data Fig. 9 [file 44319_2026_724_MOESM9_ESM.zip › Figure 9/Figure 9C/Figure 9C GAPDH.tif]

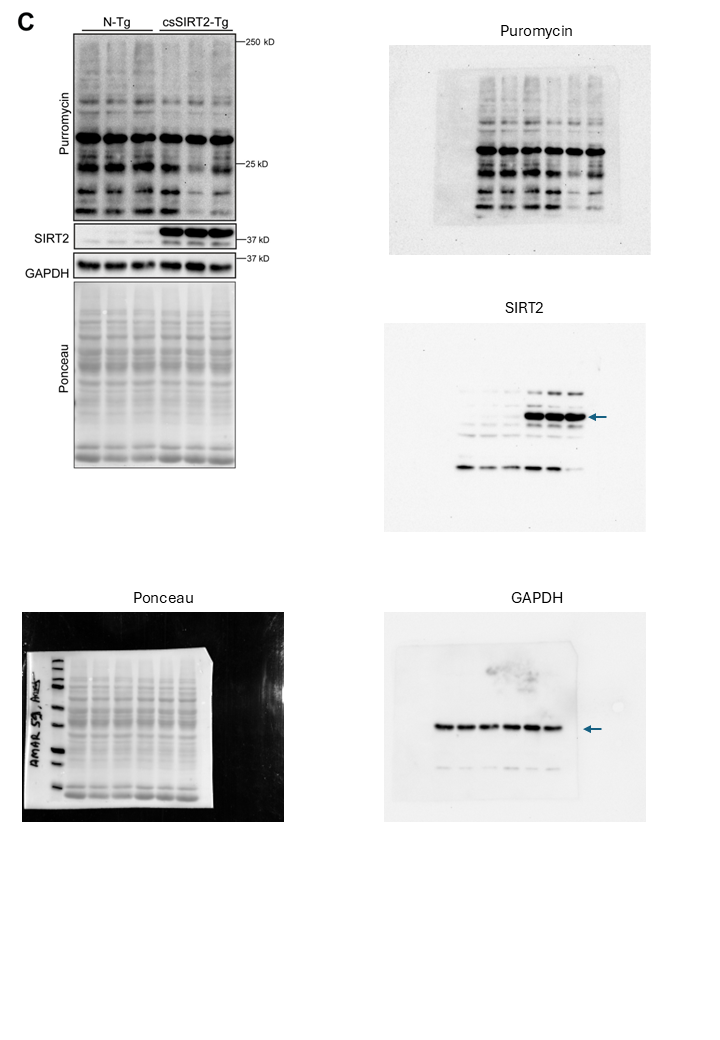

Supplement: Supplementary file 9 — Source data Fig. 9 [file 44319_2026_724_MOESM9_ESM.zip › Figure 9/Figure 9C/Figure 9C merged.tif]

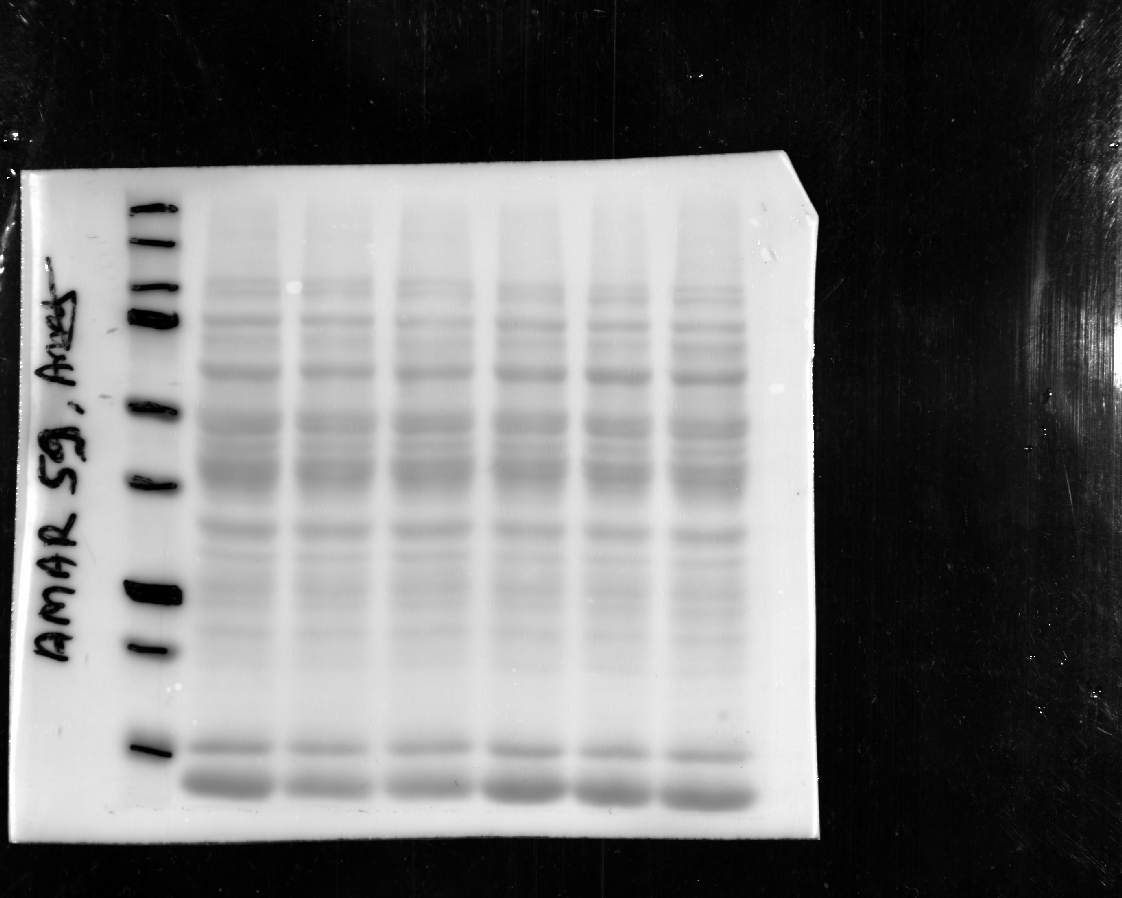

Supplement: Supplementary file 9 — Source data Fig. 9 [file 44319_2026_724_MOESM9_ESM.zip › Figure 9/Figure 9C/Figure 9C Ponceau.tif]

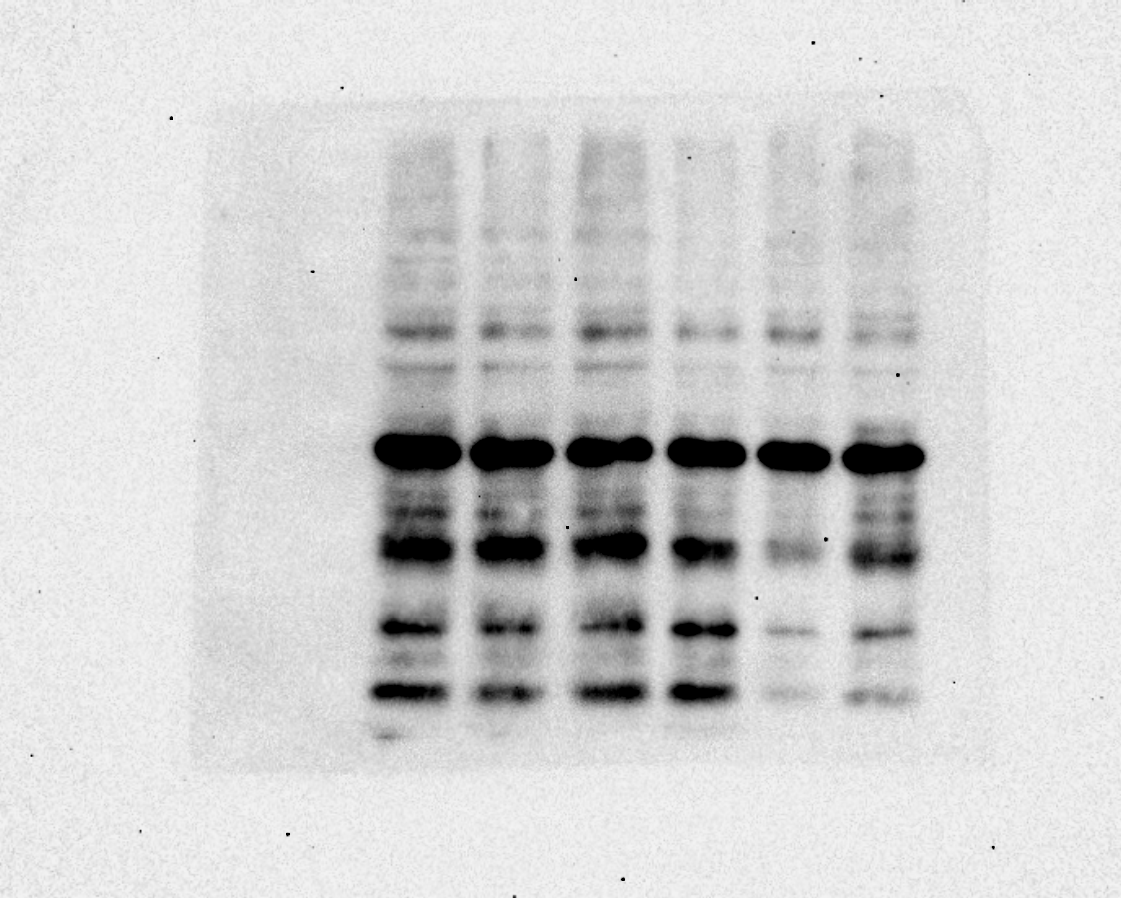

Supplement: Supplementary file 9 — Source data Fig. 9 [file 44319_2026_724_MOESM9_ESM.zip › Figure 9/Figure 9C/Figure 9C puromycin.tif]

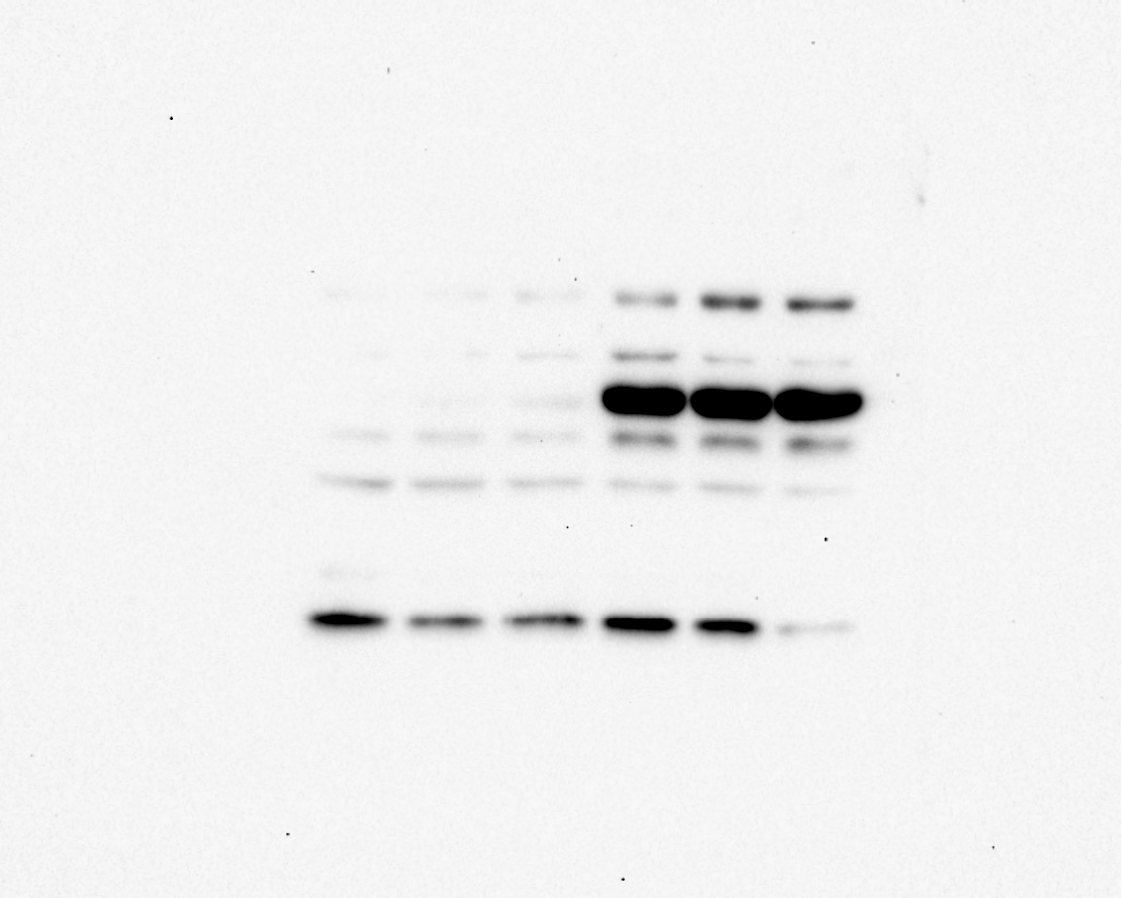

Supplement: Supplementary file 9 — Source data Fig. 9 [file 44319_2026_724_MOESM9_ESM.zip › Figure 9/Figure 9C/Figure 9C SIRT2.tif]

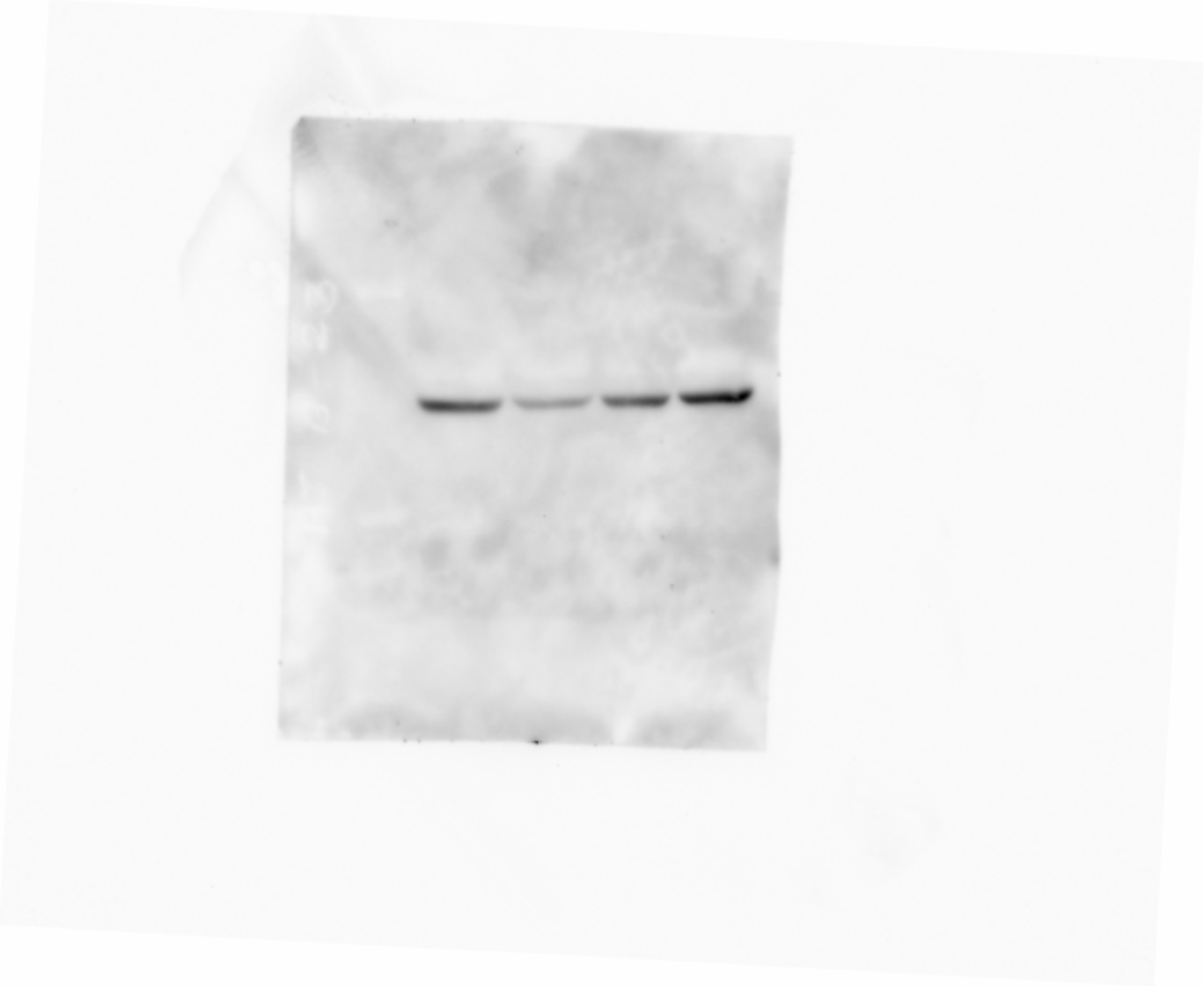

Supplement: Supplementary file 9 — Source data Fig. 9 [file 44319_2026_724_MOESM9_ESM.zip › Figure 9/Figure 9E/Figure 9E Actin.tif]

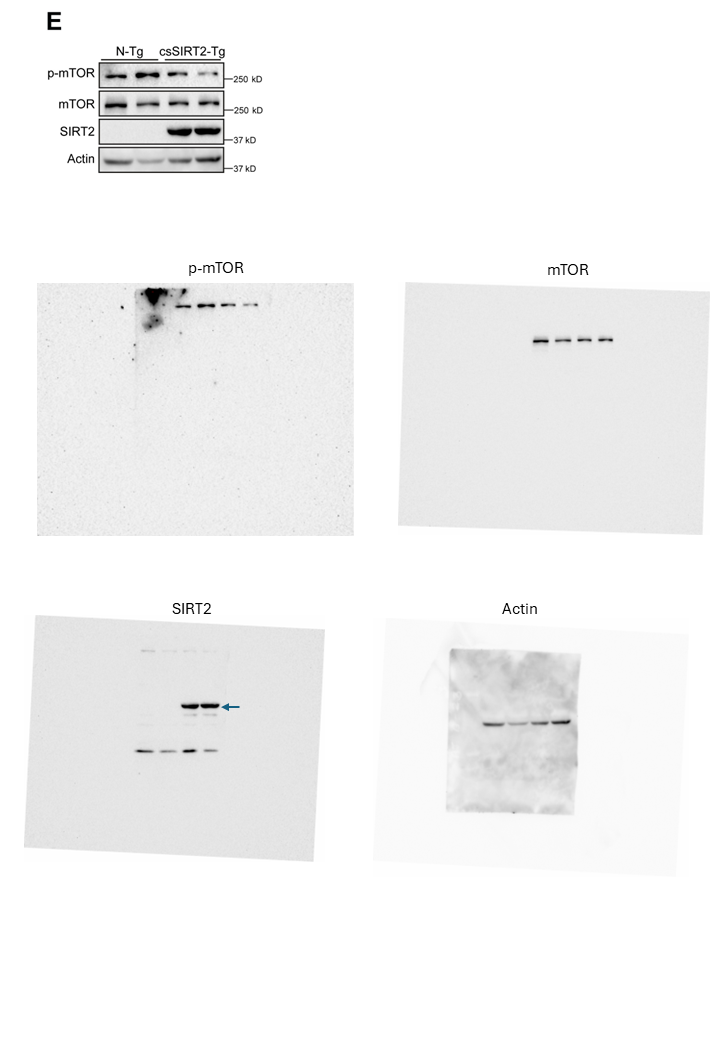

Supplement: Supplementary file 9 — Source data Fig. 9 [file 44319_2026_724_MOESM9_ESM.zip › Figure 9/Figure 9E/Figure 9E merged.tif]

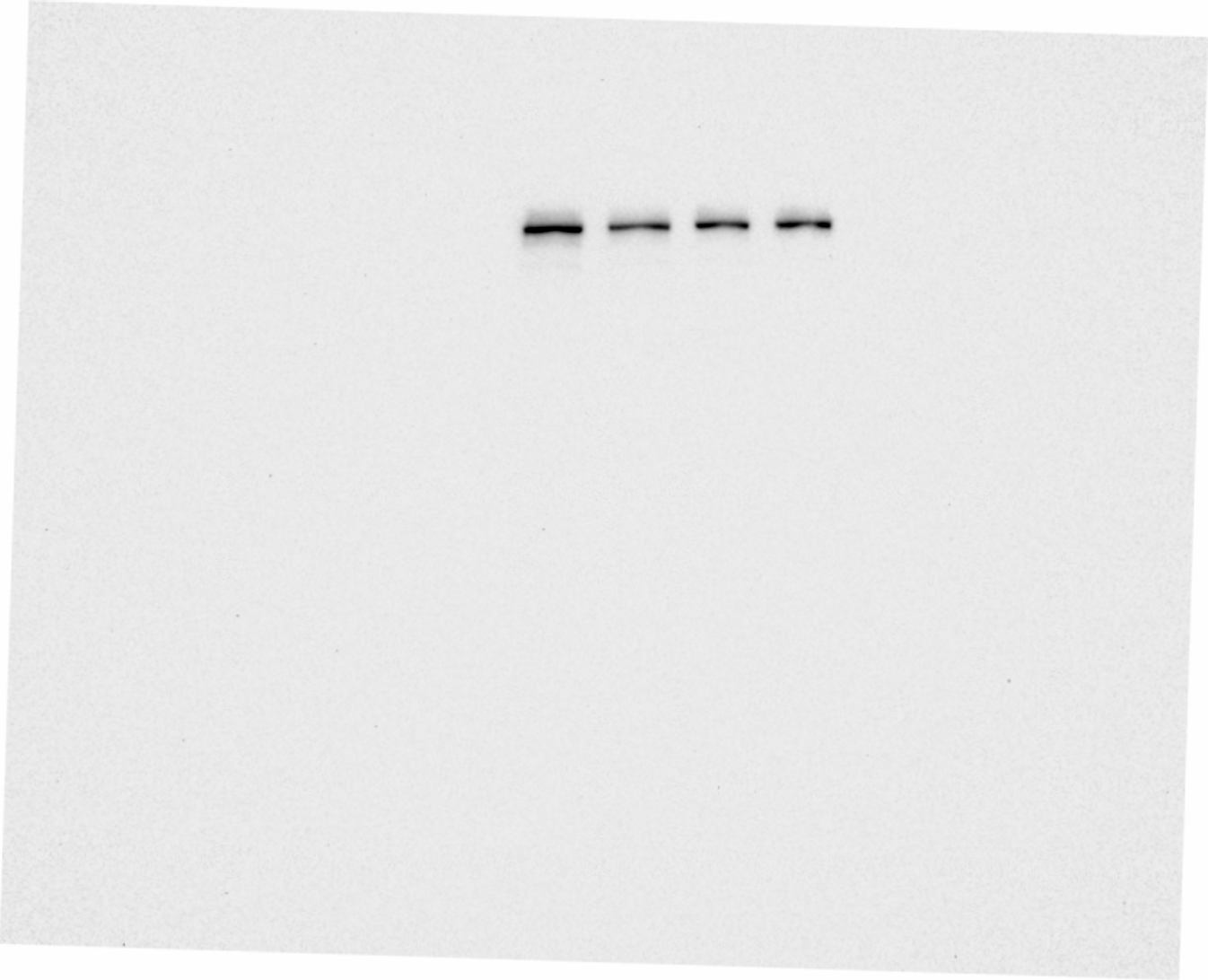

Supplement: Supplementary file 9 — Source data Fig. 9 [file 44319_2026_724_MOESM9_ESM.zip › Figure 9/Figure 9E/Figure 9E mTOR.tif]

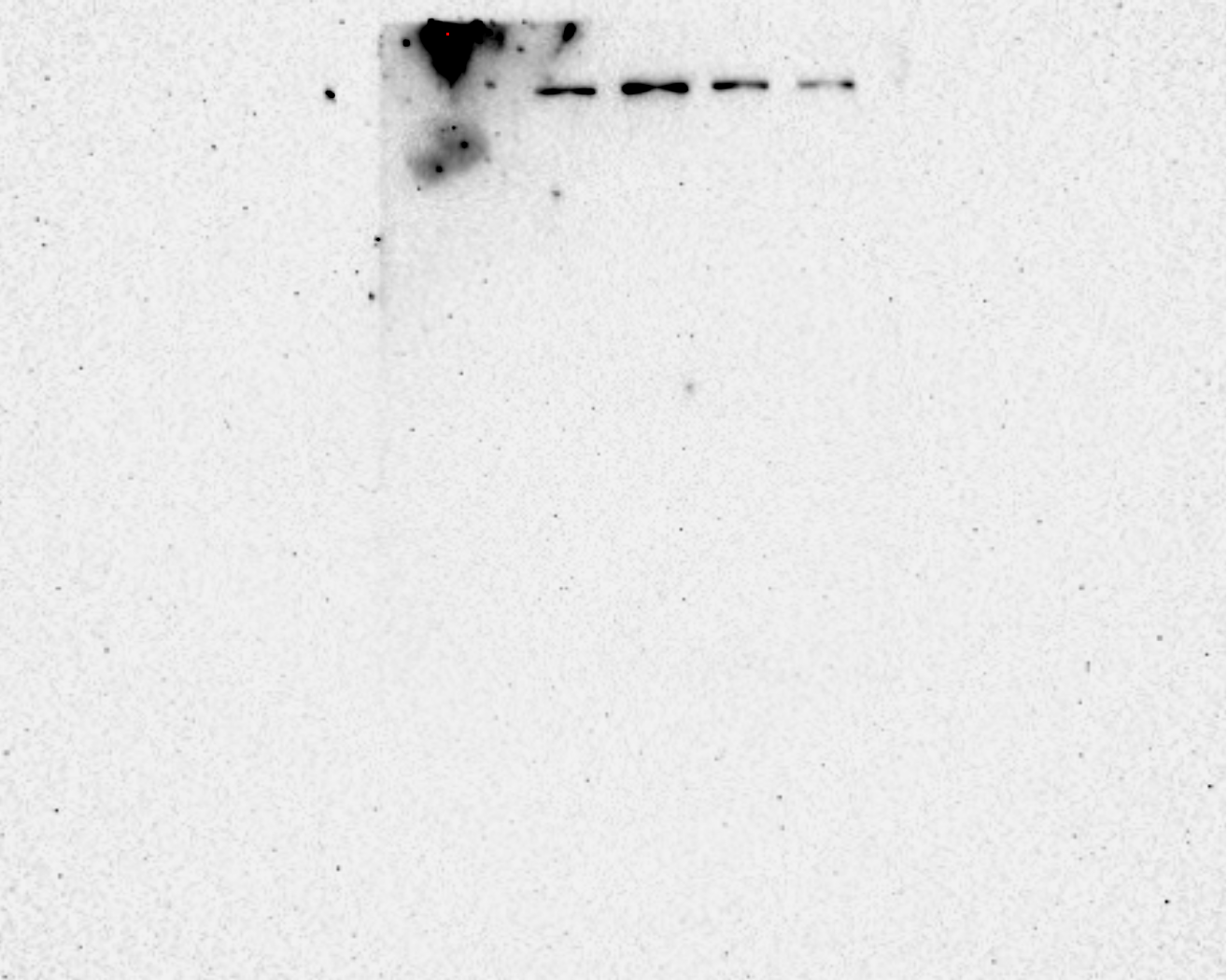

Supplement: Supplementary file 9 — Source data Fig. 9 [file 44319_2026_724_MOESM9_ESM.zip › Figure 9/Figure 9E/Figure 9E p-mTOR.tif]

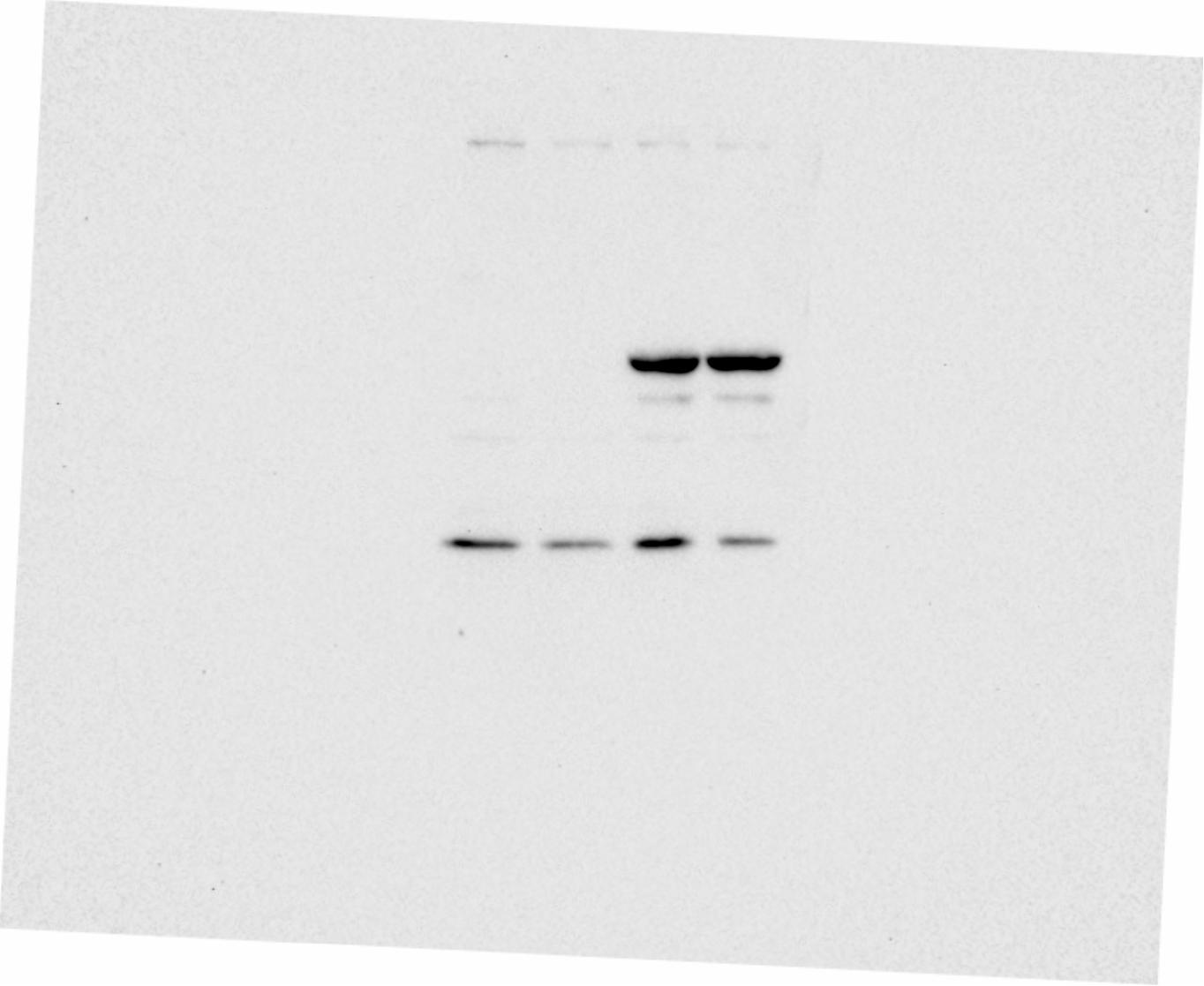

Supplement: Supplementary file 9 — Source data Fig. 9 [file 44319_2026_724_MOESM9_ESM.zip › Figure 9/Figure 9E/Figure 9E SIRT2.tif]
