# Supplementary material for: Sirtuin 2 inhibits global protein synthesis via Rheb-GTPase degradation
Source: EMBO Rep. 2026 Mar 11;27(11):3001–34. doi: 10.1038/s44319-026-00724-5 (PMC13261059; doi:10.1038/s44319-026-00724-5)
Supplement: Supplementary file 10 — Expanded View Figures [file 44319_2026_724_MOESM10_ESM.pdf]

Expanded View Figures

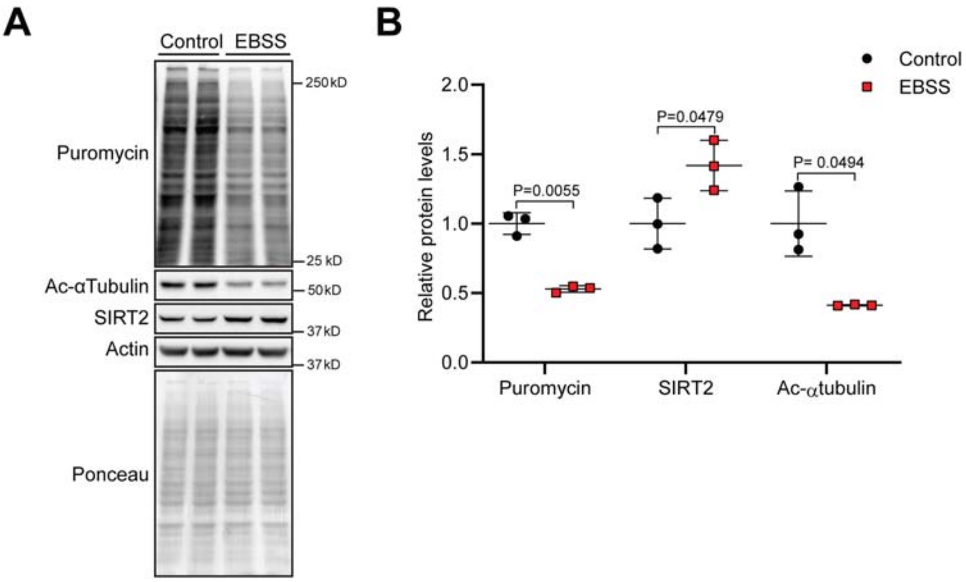

Figure EV1. SIRT2 levels and activity are upregulated in nutrient-deprived conditions along with decreased protein synthesis rates.

(**A**) Representative images of Western blotting analysis indicating the changes in protein synthesis rate (tracked using WB-SUnSET assay) and concomitant changes in the levels of acetyl-tubulin (Lysine 40) and SIRT2, in HeLa cells treated with high glucose DMEM media supplemented with 10% FBS (CM) and complete starvation using EBSS. (**B**) Scatterplot representing quantification of immunoblots from Fig. EV1A. The graph represents relative protein levels of puromycin-incorporated peptides, acetyl-tubulin, and SIRT2. $n = 3$. P values shown are from unpaired, two-tailed Student's t tests (Unpaired t test with Welch's correction), calculated separately for individual proteins. Data are presented as mean ± s.d. $n = 3$.

 © The Author(s)

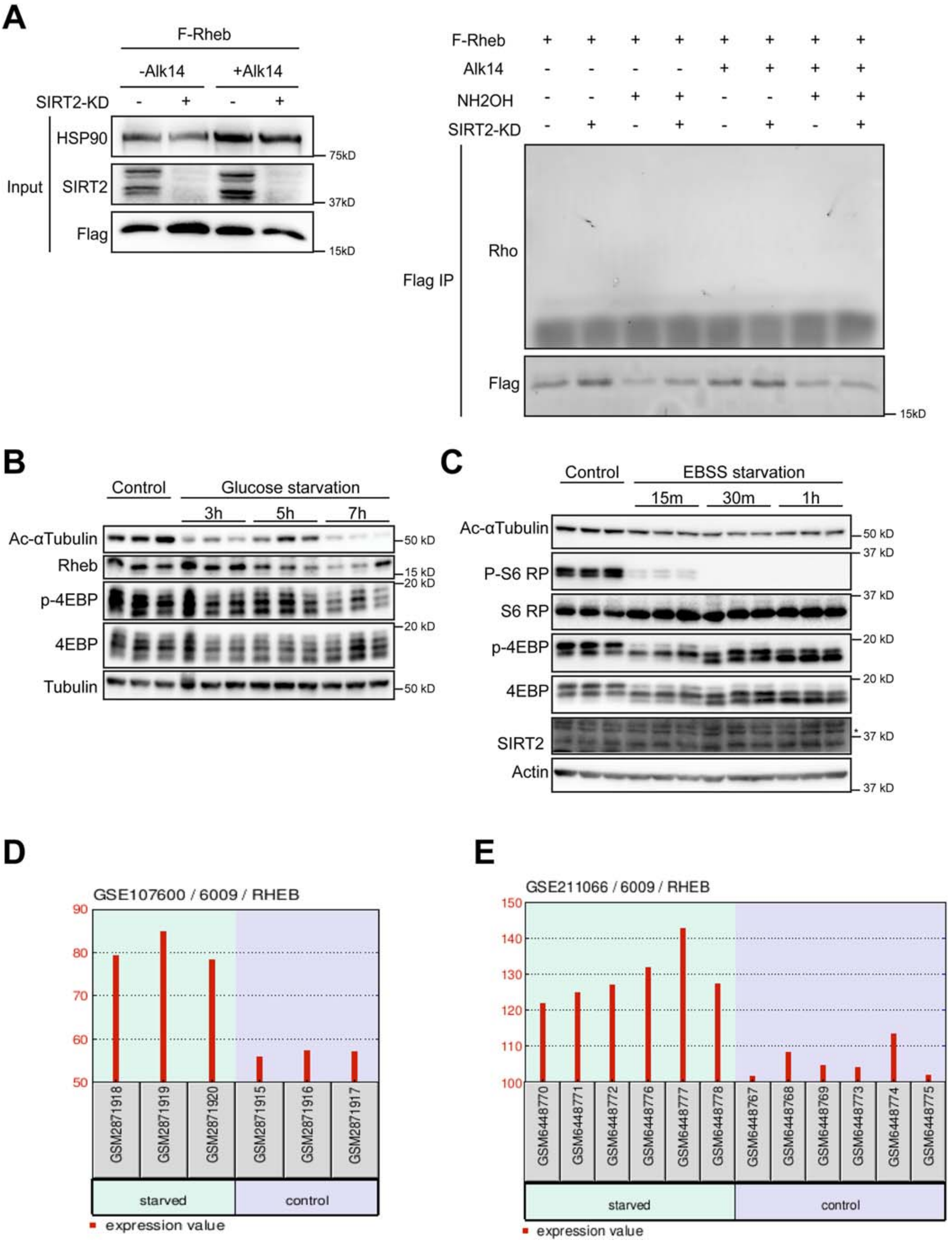
A
F-Rheb
-Alk14
+Alk14
SIRT2-KD - + - +
Input
HSP90
SIRT2
Flag
75kD
37kD
15kD
F-Rheb + + + + + + + +
Alk14 - - - - + + + +
NH2OH - - + + - - + +
SIRT2-KD - + - + - + - +
Flag IP
Rho
Flag
15kD
B
Control
Glucose starvation
3h
5h
7h
Ac-αTubulin
Rheb
p-4EBP
4EBP
Tubulin
50 kD
15 kD
20 kD
20 kD
50 kD
C
Control
EBSS starvation
15m
30m
1h
Ac-αTubulin
P-S6 RP
S6 RP
p-4EBP
4EBP
SIRT2
Actin
50 kD
37 kD
37 kD
20 kD
20 kD
*
37 kD
37 kD
D
GSE107600 / 6009 / RHEB
90
80
70
60
50
GSM2871918
GSM2871919
GSM2871920
GSM2871915
GSM2871916
GSM2871917
starved
control
expression value
E
GSE211066 / 6009 / RHEB
150
140
130
120
110
100
GSM6448770
GSM6448771
GSM6448772
GSM6448776
GSM6448777
GSM6448778
GSM6448767
GSM6448768
GSM6448769
GSM6448773
GSM6448774
GSM6448775
starved
control
expression value

◀ **Figure EV2. Rheb is not endogenously long-chain fatty acylated under normal conditions.**

(**A**) Alk14 metabolic labeling was used to detect Rheb palmitoylation. Flag-tagged RheB was overexpressed in control and SIRT2 knockdown HEK293T cells. Cells were labeled with Alk14, and samples were treated with or without hydroxylamine (which removes cysteine palmitoylation, but not lysine palmitoylation). Click chemistry was performed to conjugate a rhodamine-azide fluorescent label to proteins that are labeled with Alk14. Flag-Rheb was then pulled down, and its potential palmitoylation was detected by in-gel fluorescence. The lack of fluorescence signal indicates Rheb is not palmitoylated under the conditions tested. (**B**) Representative western blot images depicting changes in levels of Rheb protein in HEK293T cells under glucose-deprivation provided for 3, 5, 7 h. Acetylation of α-tubulin (Lysine 40) was assessed to evaluate SIRT2 activity. 4EBP phosphorylation (Threonine 37/46) was assessed to check mTORC1 activity, $n = 3$ independent experiments per group. (**C**) Representative western blot images depicting changes in levels of the proteins involved in the mTOR signaling pathway in neonatal rat cardiomyocytes under EBSS starvation conditions. Acetylation of α-tubulin (Lysine 40) was assessed to evaluate SIRT2 activity, phosphorylation of S6-RP at Serine 235/236, and phosphorylation of 4EBP at Threonine 37/46 was checked. $n = 3$ independent experiments per group. * indicates non-specific band. (**D**) Representative graph obtained from RNA-seq database (GSE107600/6009/RHEB), depicting Rheb expression levels in Human HAP1 cells with 6-h EBSS starvation. (**E**) Representative graph obtained from RNA-seq database (GSE211066/6009/RHEB), depicting Rheb expression levels in HeLa cells with 4-h HBSS starvation.

 © The Author(s)

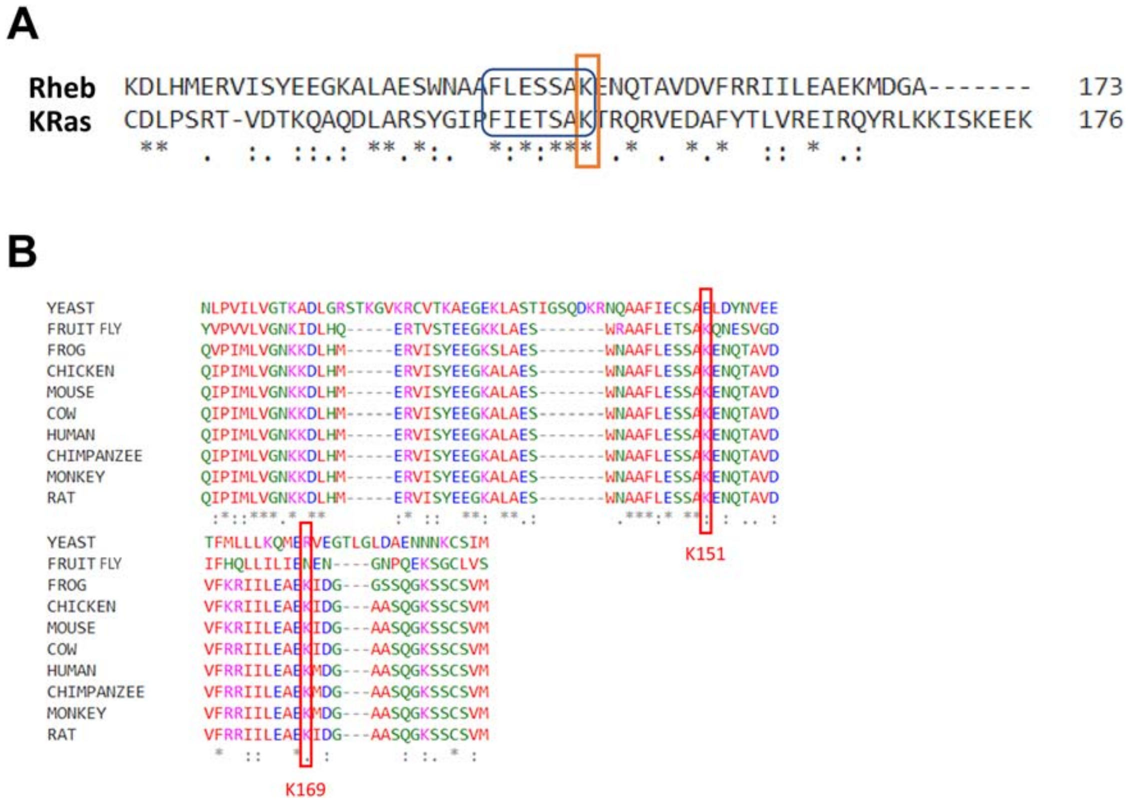

Figure EV3. Lysine 151 is a conserved residue across different species; Rheb and KRas share a common sequence stretch for SIRT2-targeted Lysine residue.

(**A**) Representative image showing sequence similarity in SIRT2 targeted region of Rheb and KRas, Rheb and KRas share a similar sequence stretch of amino acids for K151 and K147 residues, respectively. (**B**) Sequence alignment data depicting K151 residue in Rheb is conserved across different species.

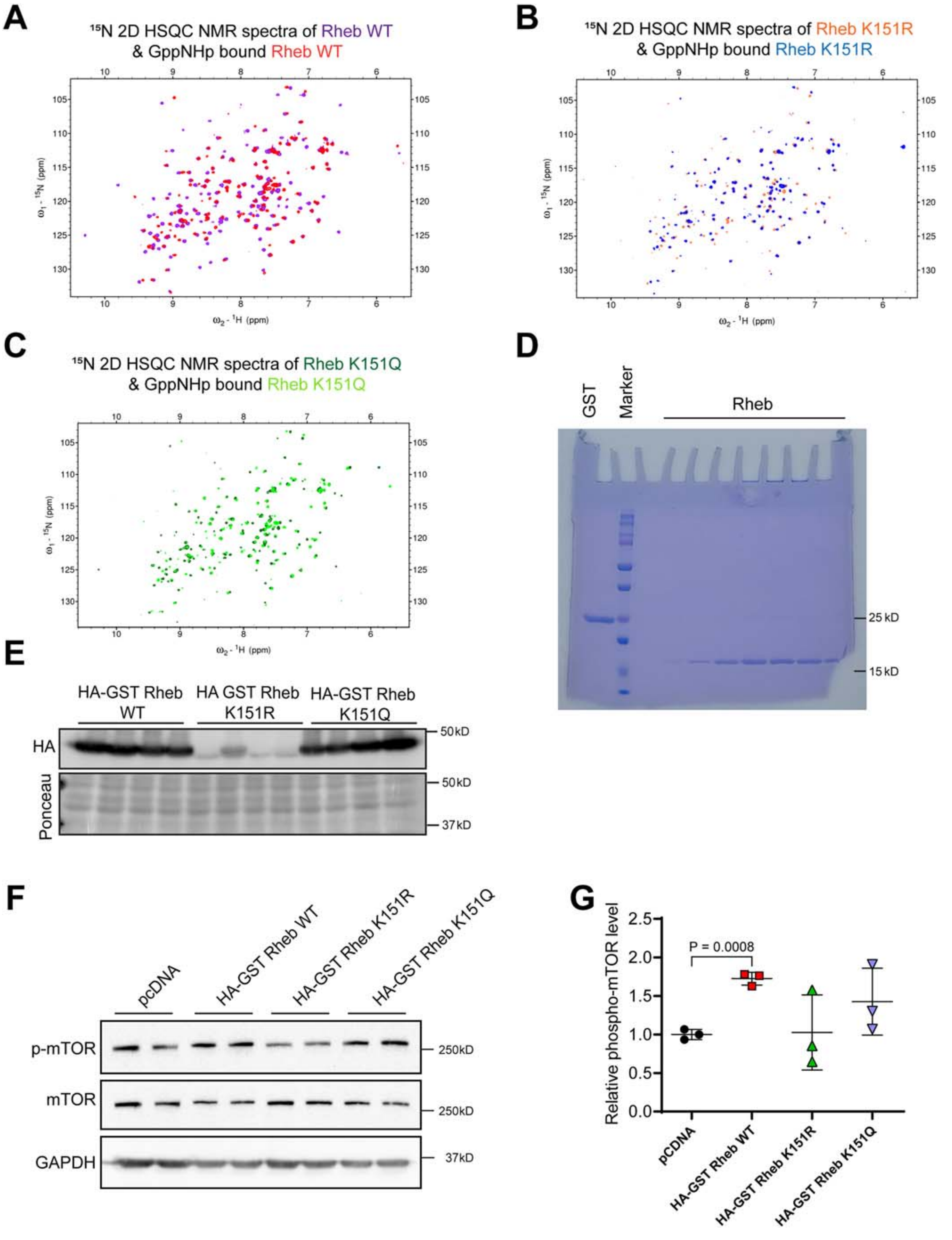
A
15N 2D HSQC NMR spectra of Rheb WT
& GppNHp bound Rheb WT
B
15N 2D HSQC NMR spectra of Rheb K151R
& GppNHp bound Rheb K151R
C
15N 2D HSQC NMR spectra of Rheb K151Q
& GppNHp bound Rheb K151Q
D
GST
Marker
Rheb
25 kD
15 kD
E
HA-GST Rheb WT
HA GST Rheb K151R
HA-GST Rheb K151Q
HA
Ponceau
50kD
37kD
F
pcDNA
HA-GST Rheb WT
HA-GST Rheb K151R
HA-GST Rheb K151Q
p-mTOR
mTOR
GAPDH
250kD
37kD
G
Relative phospho-mTOR level
P = 0.0008

© The Author(s)

Figure EV4. Rheb mutants K151R and K151Q are structurally stable in basal and GppNHp-bound form.

(**A**) Representative overlaid 2D ^{1}H-^{15}N HSQC NMR spectra of Rheb WT & GppNHp bound Rheb WT. (**B**) Representative overlaid 2D ^{1}H-^{15}N HSQC NMR spectra of Rheb K151R & GppNHp bound Rheb K151R. (**C**) Representative overlaid 2D ^{1}H-^{15}N HSQC NMR spectra of Rheb K151Q & GppNHp-bound Rheb K151Q. (**D**) Representative SDS-PAGE gel image showing purified Rheb protein at 17 kD molecular weight. (**E**) Representative images of western blot analysis showing differential expression of HA-GST Rheb, HA-GST Rheb K151R, and HA-GST Rheb K151Q. Ponceau of the same blot is used as a loading control. (**F**) Representative western blotting images depicting changes in mTOR phosphorylation at Serine 2448 in HeLa cells transfected with pcDNA, HA-GST Rheb WT, HA-GST Rheb K151R, and HA-GST Rheb K151Q, $n = 3$. (**G**) Quantification of mTOR phosphorylation depicted in Figure EV4F. The results are expressed as the fold change relative to pcDNA control. P values shown are from the one-way ANOVA test with Dunnett's T3 multiple comparisons. Data are presented as mean ± s.d., $n = 3$.

© The Author(s)

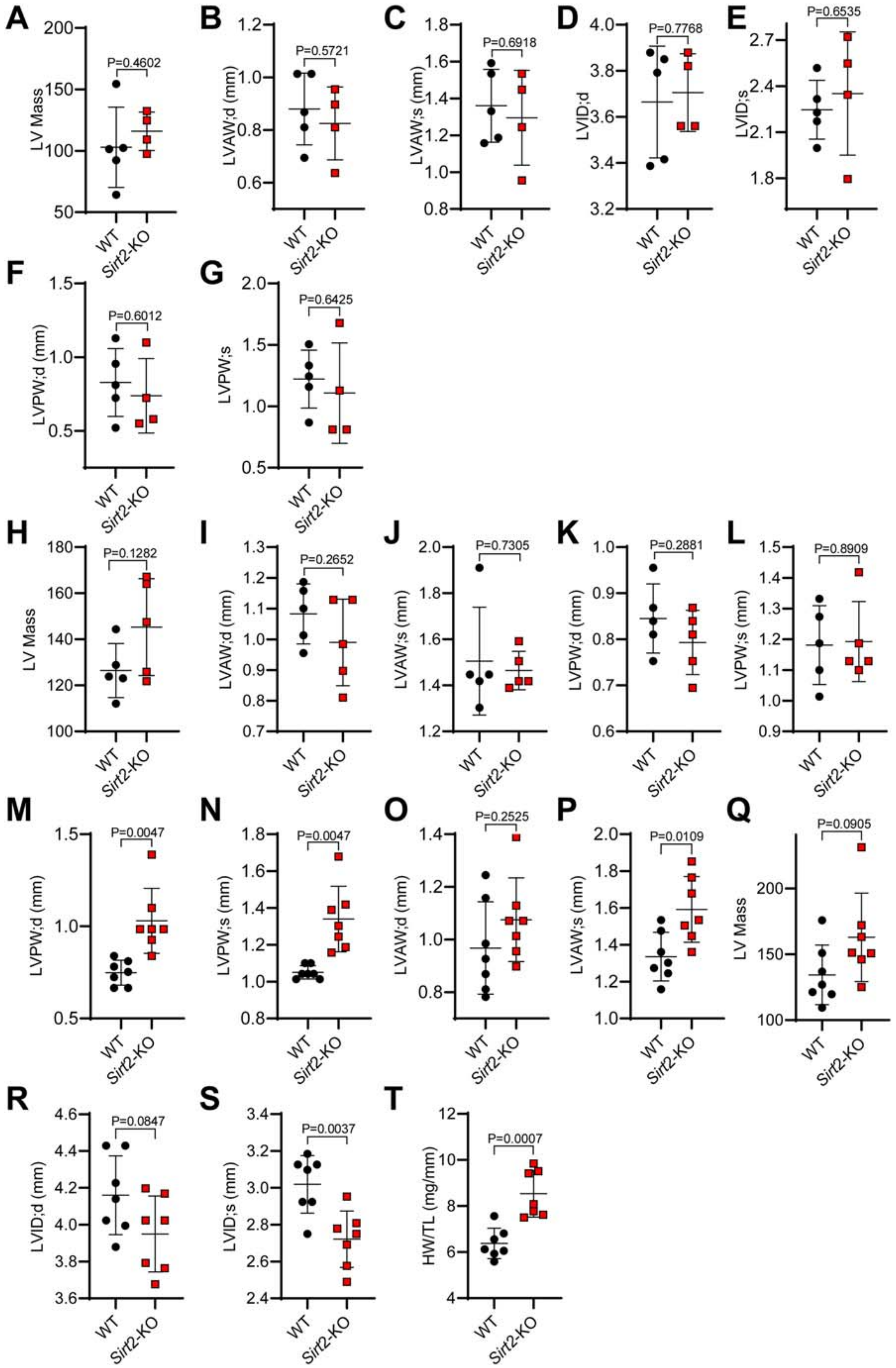
A
LV Mass
P=0.4602
WT
Sirt2-KO
B
LVAW;d (mm)
P=0.5721
C
LVAW;s (mm)
P=0.6918
D
LVID;d
P=0.7768
E
LVID;s
P=0.6535
F
LVPW;d (mm)
P=0.6012
G
LVPW;s
P=0.6425
H
LV Mass
P=0.1282
I
LVAW;d (mm)
P=0.2652
J
LVAW;s (mm)
P=0.7305
K
LVPW;d (mm)
P=0.2881
L
LVPW;s (mm)
P=0.8909
M
LVPW;d (mm)
P=0.0047
N
LVPW;s (mm)
P=0.0047
O
LVAW;d (mm)
P=0.2525
P
LVAW;s (mm)
P=0.0109
Q
LV Mass
P=0.0905
R
LVID;d (mm)
P=0.0847
S
LVID;s (mm)
P=0.0037
T
HW/TL (mg/mm)
P=0.0007

 © The Author(s)

Figure EV5. Representative graph of echocardiographic analysis showing changes in the various cardiac parameters of 4, 9, and 12-months-old *Sirt2-KO* mice.

(**A–G**) Representative graph of echocardiographic analysis showing changes in the various cardiac parameters of 4-month-old *Sirt2*-KO mice when compared with age-matched wild-type mice controls. Data are presented as mean ± s.d., $n = 4$–5 mice per group. *P* values shown are from unpaired, 2-tailed Student's *t* tests. Data are shown as mean ± s.d. (**H–L**) Representative graph of echocardiographic analysis showing changes in the various cardiac parameters of 9-month-old *Sirt2*-KO mice when compared with age-matched wild-type mice controls. Data are presented as mean ± s.d., $n = 5$ mice per group. *P* values shown are from unpaired, 2-tailed Student's *t* tests. Data are shown as mean ± s.d. (**M–T**) Representative graph of echocardiographic analysis showing changes in the various cardiac parameters of 12-month-old *Sirt2*-KO mice when compared with age-matched wild-type mice controls. Data are presented as mean ± s.d., $n = 7$ mice per group. *P* values shown are from unpaired, 2-tailed Student's *t* tests. Data are shown as mean ± s.d.

© The Author(s)

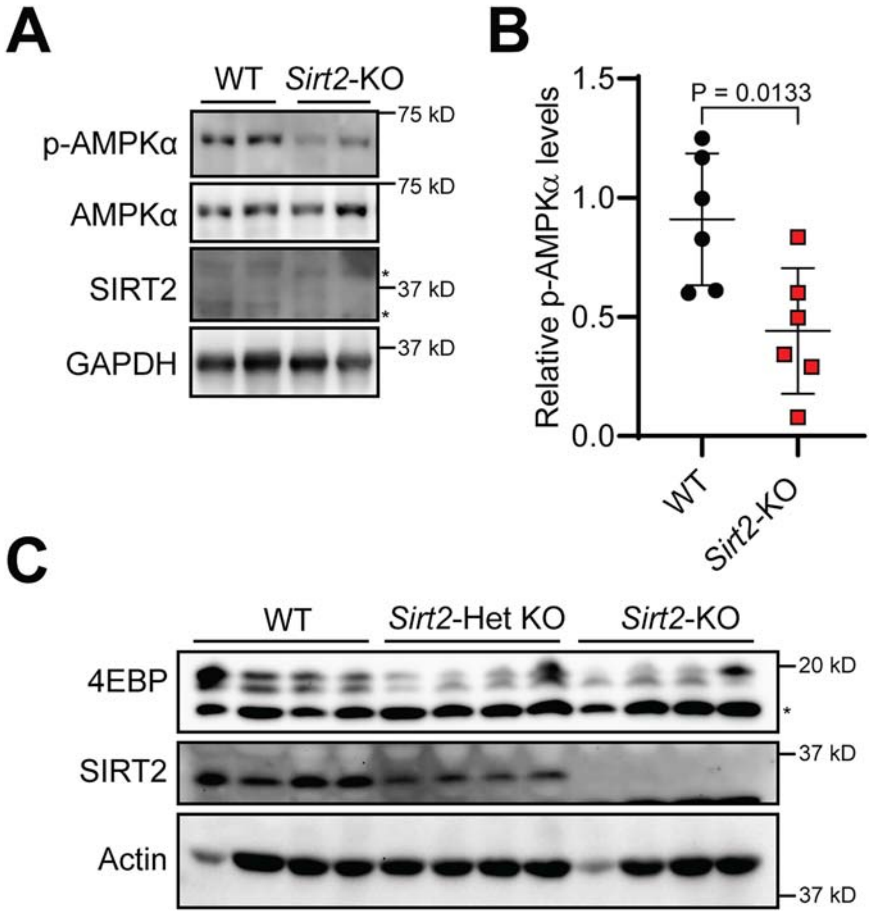

Figure EV6. *Sirt2*-KO mice exhibit inhibition of AMPKα and a decrease in 4EBP levels.

(**A**) Representative western blotting images depicting changes in AMPKα phosphorylation Threonine 172, in heart tissues of WT controls and *Sirt2*-KO mice, $n = 6$ animals per group. * Indicates non-specific band. (**B**) Scatterplot representing the changes in AMPKα protein phosphorylation in heart tissues of WT controls and *Sirt2*-KO mice depicted in Fig. EV6A. *P* values shown are from unpaired, two-tailed Student's *t* test. Data are presented as mean ± s.d., $n = 6$ per group. (**C**) Representative western blotting images depicting changes in 4EBP levels in heart tissues of WT controls, *Sirt2*- Het KO and *Sirt2*-KO mice, $n = 4$ mice per group. * Indicates non-specific band.

 © The Author(s)
