# Supplementary material for: Sirtuin 2 inhibits global protein synthesis via Rheb-GTPase degradation
Source: EMBO Rep. 2026 Mar 11;27(11):3001–34. doi: 10.1038/s44319-026-00724-5 (PMC13261059; doi:10.1038/s44319-026-00724-5)
Supplement: Supplementary file 1 — Peer Review File [file 44319_2026_724_MOESM1_ESM.pdf]

EMBO reports

Sirtuin 2 inhibits global protein synthesis via Rheb-GTPase degradation

Amarjeet Shrama, Yanlin Zi, Anwit Pandit, Kirtika Jha, Vikrant Sinha, Dimple Nagesh, Bhoomika Shivanaiah, Venkatraman Ravi, Souvik Ghosh, Danish Khan, Arathi Prabhashankar, Thoniparambil Sumi, Satish Rajpurohit, Sunayana Ningaraju, Sukanya Raghu, Anand Srivastava, Mahavir Singh, Hening Lin, and Nagalingam Sundaresan

Corresponding author(s): Nagalingam Sundaresan (rsundaresan@iisc.ac.in)

Review Timeline:

Submission Date:	30th Mar 25
Editorial Decision:	26th May 25
Revision Received:	19th Oct 25
Editorial Decision:	17th Dec 25
Revision Received:	24th Dec 25
Accepted:	26th Jan 26

Editor: Esther Schnapp

Transaction Report:

(Note: With the exception of the correction of typographical or spelling errors that could be a source of ambiguity, letters and reports are not edited. Depending on transfer agreements, referee reports obtained elsewhere may or may not be included in this compilation. Referee reports are anonymous unless the Referee chooses to sign their reports.)

1st Editorial Decision **26th May 2025**

Dear Dr. Sundaresan,

Thank you for your patience while your manuscript was peer-reviewed at EMBO reports. We have now received the full set of referee reports as well as referee cross-comments that are all pasted below.

As you will see, the referees acknowledge that the findings are interesting. However, they also all have several suggestions for how the ms could be improved and strengthened. In principle, all suggestions are good and should be addressed, except point 1 by referee 4, which - while certainly very welcome additions - seems to be out of the scope of this study. Naturally, if you already have data that address these concerns, please do add them to the ms. Referee 2 also has a suggestion for how these points could be addressed.

What I find more important are the additional comments by referee 4 on the back-to-back situation of both ms. It is true that the two studies do not integrate each others' findings, despite shared authorships. I would like to ask you to address this issue, and please discuss with the authors of the other ms how both findings could be integrated better. You can send me a revision plan and we can discuss this before you start revising your studies, also in a video chat, if you like.

I would thus like to invite you to revise your manuscript with the understanding that the referee concerns must be fully addressed and their suggestions taken on board. Please address all referee concerns in a complete point-by-point response. Acceptance of the manuscript will depend on a positive outcome of a second round of review. It is EMBO reports policy to allow a single round of major revision only and acceptance or rejection of the manuscript will therefore depend on the completeness of your responses included in the next, final version of the manuscript.

We realize that it is difficult to revise to a specific deadline. In the interest of protecting the conceptual advance provided by the work, we recommend a revision within 3 months (26th Aug 2025). Please discuss the revision progress ahead of this time with the editor if you require more time to complete the revisions.

IMPORTANT NOTE: we perform an initial quality control of all revised manuscripts before re-review. Your manuscript will FAIL this control and the handling will be DELAYED if the following APPLIES:
1) A data availability section providing access to data deposited in public databases is missing. If you have not deposited any data, please add a sentence to the data availability section that explains that.
2) Your manuscript contains statistics and error bars based on n=2. Please use scatter blots in these cases. No statistics should be calculated if n=2.

When submitting your revised manuscript, please carefully review the instructions that follow below. Failure to include requested items will delay the evaluation of your revision.

1) a .docx formatted version of the manuscript text (including legends for main figures, EV figures and tables). Please make sure that the changes are highlighted to be clearly visible.

2) individual production quality figure files as .eps, .tif, .jpg (one file per figure). See https://wol-prod-cdn.literatumonline.com/pb-assets/embo-site/EMBOPress_Figure_Guidelines_061115-1561436025777.pdf for more info on how to prepare your figures.

3) We replaced Supplementary Information with Expanded View (EV) Figures and Tables that are collapsible/expandable online. A maximum of 8 EV Figures can be typeset. EV Figures should be cited as 'Figure EV1, Figure EV2" etc... in the text and their respective legends should be included in the main text after the legends of regular figures.

- For the figures that you do NOT wish to display as Expanded View figures, they should be bundled together with their legends in a single PDF file called *Appendix*, which should start with a short Table of Content. Appendix figures should be referred to in the main text as: "Appendix Figure S1, Appendix Figure S2" etc. See detailed instructions regarding expanded view here: <https://www.embopress.org/page/journal/14693178/authorguide#expandedview>

- Additional Tables/Datasets should be labeled and referred to as Table EV1, Dataset EV1, etc. Legends have to be provided in a separate tab in case of .xls files. Alternatively, the legend can be supplied as a separate text file (README) and zipped together with the Table/Dataset file.

4) a .docx formatted letter INCLUDING the reviewers' reports and your detailed point-by-point responses to their comments. As part of the EMBO Press transparent editorial process, the point-by-point response is part of the Review Process File (RPF), which will be published alongside your paper.

5) a complete author checklist, which you can download from our author guidelines <https://www.embopress.org/page/journal/14693178/authorguide>. Please insert information in the checklist that is also reflected in the manuscript. The completed author checklist will also be part of the RPF.

6) Please note that all corresponding authors are required to supply an ORCID ID for their name upon submission of a revised manuscript (<https://orcid.org/>). Please find instructions on how to link your ORCID ID to your account in our manuscript tracking system in our Author guidelines <https://www.embopress.org/page/journal/14693178/authorguide#authorshipguidelines>

7) Before submitting your revision, primary datasets produced in this study need to be deposited in an appropriate public database (see https://www.embopress.org/page/journal/14693178/authorguide#datadeposition). Please remember to provide a reviewer password if the datasets are not yet public. The accession numbers and database should be listed in a formal "Data Availability" section placed after Materials & Method (see also https://www.embopress.org/page/journal/14693178/authorguide#datadeposition). Please note that the Data Availability Section is restricted to new primary data that are part of this study. * Note - All links should resolve to a page where the data can be accessed. *
If your study has not produced novel datasets, please mention this fact in the Data Availability Section.

8) At EMBO Press we ask authors to provide source data for the main manuscript figures. You will receive a separate email with instructions for providing source data with your revised manuscript, including how to upload and organize the files.

9) Our journal also encourages inclusion of *data citations in the reference list* to directly cite datasets that were re-used and obtained from public databases. Data citations in the article text are distinct from normal bibliographical citations and should directly link to the database records from which the data can be accessed. In the main text, data citations are formatted as follows: "Data ref: Smith et al, 2001" or "Data ref: NCBI Sequence Read Archive PRJNA342805, 2017". In the Reference list, data citations must be labeled with "[DATASET]". A data reference must provide the database name, accession number/identifiers and a resolvable link to the landing page from which the data can be accessed at the end of the reference. Further instructions are available at https://www.embopress.org/page/journal/14693178/authorguide#referencesformat

10) Regarding data quantification (see Figure Legends: https://www.embopress.org/page/journal/14693178/authorguide#figureformat)

The following points must be specified in each figure legend:

- the name of the statistical test used to generate error bars and P values,

- the number (n) of independent experiments (please specify technical or biological replicates) underlying each data point,

- the nature of the bars and error bars (s.d., s.e.m.),

- If the data are obtained from n Program fragment delivered error ``Can't locate object method "less" via package "than" (perhaps you forgot to load "than"?) at //ejpvfs23/sites23b/embor_www/letters/embor_decision_revise_and_review.txt line 56.' 2, use scatter blots showing the individual data points.

Discussion of statistical methodology can be reported in the materials and methods section, but figure legends should contain a basic description of n, P and the test applied.

- Please also include scale bars in all microscopy images.

11) The journal requires a statement specifying whether or not authors have competing interests (defined as all potential or actual interests that could be perceived to influence the presentation or interpretation of an article). In case of competing interests, this must be specified in your disclosure statement. Further information: https://www.embopress.org/competing-interests

12) All Materials and Methods need to be described in the main text using our 'Structured Methods' format, which is required for all research articles. According to this format, the Methods section includes a Reagents and Tools Table (listing key reagents, experimental models, software and relevant equipment and including their sources and relevant identifiers) followed by a Methods and Protocols section describing the methods using a step-by-step protocol format. The aim is to facilitate adoption of the methodologies across labs. More information on how to adhere to this format as well as a downloadable template (.docx) for the Reagents and Tools Table can be found in our author guidelines: https://www.embopress.org/page/journal/14693178/authorguide#structuredmethods.

An example of a Method paper with Structured Methods can be found here: https://www.embopress.org/doi/full/10.1038/s44320-024-00037-6#sec-4

We would also welcome the submission of cover suggestions, or motifs to be used by our Graphics Illustrator in designing a cover.

As part of the EMBO publication's Transparent Editorial Process, EMBO reports publishes online a Review Process File (RPF) to accompany accepted manuscripts. This File will be published in conjunction with your paper and will include the referee reports, your point-by-point response and all pertinent correspondence relating to the manuscript.

You are able to opt out of this by letting the editorial office know (emboreports@embo.org). If you do opt out, the Review Process File link will point to the following statement: "No Review Process File is available with this article, as the authors have chosen not to make the review process public in this case."

I look forward to seeing a revised form of your manuscript when it is ready.

Best regards,
Esther

Esther Schnapp, PhD
Senior Editor
EMBO reports

Referee #1:

In this study, Sharma et al focused on the role of SIRT2 in cardiac hypertrophy growth and protein synthesis in the heart. The authors demonstrated that SIRT2 may directly deacetylate RheB and promote ubiquitination of the latter. As a result, mTOR signaling is diminished, and cardiomyocyte growth is attenuated. A number of biochemical and cellular biology methods have been employed. In addition, both KO and TG mice were used. Overall, this is a solid study with convincing evidence and conclusions.

1. The authors reported in 2018 that SIRT2 deacetylates NFATc2, and this process is involved in cardiac hypertrophic growth (JBC). It is unclear to this reviewer how this mechanism is linked to the current SIRT2-RheB-mTOR axis. Would both mechanisms contribute to the growth phenotype? Which is major?
2. The authors characterized mouse hypertrophy phenotype in KO and TG mice. However, it would be more informative if cardiac function can be assessed. In this regard, pathological stimuli, such as TAC, may be required.
3. To assess mTOR signaling, the authors mainly examined mTOR phosphorylation. What about phosphorylation of S6K1, S6, 4EBP1, and AMPK?

Referee #2:

The manuscript by Shrama et al. shows that SIRT2, a member of the Sirtuin family of deacetylases, targets lysine-151 of the small GTPase protein Rheb for deacetylation. Acetylation of Rheb at K151 is suggested not to affect GTP binding, GDP dissociation, or membrane binding, but allows for its accumulation to stimulate mTOR activity and cap-dependent translation. Deacetylation of Rheb by SIRT2 results in ubiquitin-dependent degradation of Rheb, reduced mTOR activity, and reduced protein synthesis. Finally, the authors show that hypertrophic hearts from SIRT2-KO mice display increased protein synthesis due to increased Rheb levels and increased mTOR activity. With just a few exceptions, the studies carried out by Shrama et al. appear highly rigorous, and their results support the major conclusions of the manuscript. The key finding that Rheb is a major substrate for SIRT2, regulating protein synthesis, is highly significant and will be of general interest to the translation field. Some additional major and minor comments on the manuscript are listed below.

Major comments:

1. Fig. 1D, this panel shows increased expression of acetylated-tubulin following treatment with the SIRT2 inhibitor AGK2, but is not specifically referred to or explained in the text.

2. In Fig. 1G, overexpression of wild-type SIRT2 results in a significant increase in puromycin labeling, suggesting increased protein synthesis. In Fig. 1J, overexpression of wild-type SIRT2 does not result in a significant increase in puromycin labeling. What is the difference between these two experiments, and how do the authors explain this apparent discrepancy?

3. In Fig. 1J and K, overexpression of catalytically inactive SIRT2 (N168A) significantly increased puromycin incorporation, but in Fig. 2D, overexpression of the same mutant had no effect on cap-dependent translation. Please explain this apparent discrepancy?

4. The manuscript shows increased Rheb levels in heart tissue from SIRT2-KO mice, consistent with the idea that deacylation of Rheb by SIRT2 results in its degradation. Are the increased levels of Rheb in the heart acetylated?

5. Figures 6F-I are not sufficiently explained for the reader.

Minor comments:

1. Line 217, deacylase should be deacylated

2. Line 261; the authors find that knock-down of Rheb expression using siRNA blocks the ability of AGK2 inhibitor of SIRT2 to stimulate cap-dependent translation. The sentence describing this effect is worded a bit confusingly: "Furthermore, we observed that downregulating Rheb can rescue the increased cap-dependent translation under SIRT2-inhibited conditions. 262 [Figure 3K, L]."

Referee #3:

Review of Manuscript EMBOR-2025-61644V1 by Shrama et al.
The manuscript presents novel findings suggesting that SIRT2 deficiency increases protein synthesis via a mechanism whereby SIRT2 binds to and deacetylates Rheb at K151 residue to enhance ubiquitin-proteasome-mediated degradation of Rheb.
The manuscript is of very good quality, with well-controlled experiments and mechanistic insights supporting the authors' conclusions. However, the points below should be addressed to strengthen the manuscript further.
- In figure 4C, in the HA IP blot section, the blot of HA (Rheb) shows 1 main band for HA. In the KAc blot, there are 3 bands shown, with one identified with an arrow, presumably meaning that it's Rheb. Can the authors comment on what the other 2 bands are? They also seem to decrease in the K151R mutant lane.
- In figs. 2G and 6E, please indicate which phosphorylation site(s) are being shown.
- Could quantification of blots be provided for fig 6J (and the associated relative puromycin incorporation vs control blots).
- The term "protein translation" is found at several places in the document. Please replace with either "mRNA translation" or "protein synthesis".

Referee #4:

This study reveals an important regulatory mechanism in protein synthesis control. Sharma et al. demonstrate how SIRT2 regulates global protein synthesis through deacetylation of Rheb GTPase. Their work shows that SIRT2 removes specific chemical tags (deacetylation at K151) from Rheb, which promotes its ubiquitination and proteasomal degradation. This degradation reduces mTORC1 activity and subsequently suppresses global protein synthesis. The authors provide evidence that SIRT2-deficient mice develop age-associated cardiac hypertrophy with increased cardiac protein synthesis, while cardiac-specific SIRT2 overexpression reduces protein synthesis in mouse hearts.
The identification of Rheb as a SIRT2 substrate represents a significant advance in our understanding of translational control. However, three important conceptual gaps require attention before this manuscript is suitable for publication in EMBO Reports.
Major Concerns
1. Causal mechanism between deacetylation and ubiquitination
The authors show that SIRT2 removes acetyl groups from Rheb at position K151 and that this promotes ubiquitination and degradation. However, the mechanistic connection between these events remains unclear. Key questions need addressing:
- Which specific E3 ligase targets deacetylated Rheb for degradation?
- How does deacetylation create recognition sites for the ubiquitination machinery?
- What is the structural relationship between K151 deacetylation and the ubiquitination sites?
2. Limited exploration of physiological relevance
The biological importance of this pathway requires stronger experimental support. While the authors demonstrate correlation between SIRT2 deficiency and cardiac hypertrophy, they haven't shown:
- Whether this pathway is essential for cardiac function under physiological stress conditions
- How acute SIRT2 manipulation affects cardiac responses
- Whether mTORC1 inhibition (e.g., rapamycin treatment) can rescue SIRT2-deficient cardiac phenotypes
3. Incomplete characterization of SIRT2-Rheb interaction with mTORC1 signaling
The study needs to address how this newly discovered mechanism integrates with known regulatory pathways. Critical questions include:
- How does Rheb acetylation status affect its interaction with mTORC1?
- Does acetylation influence other Rheb post-translational modifications?
- How do cells coordinate these different regulatory mechanisms during stress?
Minor Concerns
The specificity of SIRT2's effects on cardiac tissue requires additional validation through testing in other tissues or cell types. Additionally, the authors should address how this mechanism relates to other known SIRT2 functions in metabolic regulation, including potential cross-talk between pathways.

Cross-comments from referee 1 on point 1 by referee 4:

I read the paper again and also the comments from reviewer 4. I do not think that it is necessary to address the three questions you mentioned. Admittedly, they are important. However, addressing these new questions would not add too much new insights to the current study.

Cross-comments from referee 2 on point 1 by referee 4:

In my mind, identifying the specific E3 required for the degradation of Sirt2, and determining how the degradation machinery recognizes deacetylated Rheb are mechanistic details for future studies and would require extensive additional experimentation. Concerning point #3 about the structural relationship between the site of acetylation (K151) and the ubiquitination sites, there is a crystal structure of Rheb available, and the authors may be able to comment on this in their discussion.

Additional comments from referee 4:

I'm including some comments directly to you, considering them both together. For my nomenclature: Paper 1 = Zi et al. and Paper 2 = Sharma et al.

Overall, these companion papers present disconnected mechanisms for SIRT2's regulation of protein translation without adequate integration. For example, Paper 1 demonstrates SIRT2 deacetylates 4EBP1 downstream of mTORC1, while Paper 2 shows SIRT2 deacetylates Rheb upstream of mTORC1-with both ultimately suppressing translation; despite shared authorship, the manuscripts fail to reconcile these pathways, explain their relative importance in different contexts, or investigate potential competition between substrates; neither paper presents a unified model showing how these mechanisms interact during stress responses or in cardiac hypertrophy, leaving readers without a comprehensive understanding of SIRT2's role in translation regulation. So while both papers offer valuable insights into SIRT2's role in regulating protein translation, they present largely disconnected mechanisms without adequately addressing how these pathways interact. This represents a significant missed opportunity to develop an integrated framework that would better explain SIRT2's biological functions and potential therapeutic applications. For back-to-back publications, there's a surprising lack of integration that would provide a more comprehensive understanding of SIRT2's role in translation regulation. Here are some specific points:

1. Different Mechanisms for the Same Outcome
Both papers describe SIRT2's role in suppressing protein translation, but through entirely different mechanisms:
- Paper 1 claims SIRT2 deacetylates 4EBP1, stabilizing it and preventing eIF4E from initiating translation
- Paper 2 claims SIRT2 deacetylates Rheb, promoting its degradation and reducing mTORC1 activity

While complementary mechanisms are possible, neither paper acknowledges the alternative pathway described by the other, which is peculiar for back-to-back publications.

2. Insufficient Integration of Findings
Paper 2 briefly mentions Paper 1 in a single sentence: "Interestingly enough, in another manuscript under submission by Prof. Hening Lin's lab, SIRT2 is shown to deacetylate 4EBP1 leading to its stabilization, thereby suppressing translation by inactivating mTORC1."

However, this integration is minimal and doesn't address:
- The relative importance of each mechanism
- Whether these mechanisms operate simultaneously or in different contexts
- How these mechanisms interact in the regulatory network

3. Conflicting Statements About mTORC1 Inactivation
The papers present different accounts of how SIRT2 affects mTORC1:
- Paper 1 suggests SIRT2 acts downstream of mTORC1 by stabilizing 4EBP1 (which is a target of mTORC1)
- Paper 2 claims SIRT2 acts upstream of mTORC1 by destabilizing Rheb (which activates mTORC1)

Paper 2's brief mention that SIRT2's 4EBP1 deacetylation "suppress[es] translation by inactivating mTORC1" contradicts Paper 1, which doesn't claim 4EBP1 inactivates mTORC1 but rather acts downstream of it.

4. Incomplete Physiological Context
Both papers examine SIRT2's role in cardiac hypertrophy:
- Paper 1 only mentions it briefly, and states SIRT2 inhibitors have anticancer activity
- Paper 2 provides more detail about SIRT2-KO mice developing cardiac hypertrophy

However, neither paper fully integrates both mechanisms to explain cardiac hypertrophy development. How do the 4EBP1 and Rheb pathways together contribute to hypertrophy? Which pathway predominates in this condition?

5. Inadequate Comparative Analysis Under Stress Conditions
Both papers examine SIRT2 under stress conditions:
- Paper 1 focuses on amino acid limitation
- Paper 2 examines complete starvation (EBSS)

A systematic comparison of how these different stress conditions affect the two regulatory mechanisms (4EBP1 vs. Rheb) is missing. Do different types of nutrient stress preferentially activate one pathway over the other?

6. Experimental Design Differences
The papers use different experimental approaches:
- Paper 1 primarily uses amino acid deprivation experiments
- Paper 2 mainly uses SIRT2 inhibition, knockdown, and overexpression

While complementarity could be a strength, these differing approaches make direct comparison of results impossible, and neither paper attempts to reconcile methodological differences.

7. Shared Authorship Without Integrated Discussion
Despite having some shared authors (including Yanlin Zi and Hening Lin), the papers don't present a unified conceptual framework. This raises questions about whether these are truly complementary studies or competing models that haven't been fully reconciled.

Response to Reviewer's comments

We sincerely thank all reviewers for their thoughtful and thorough evaluation of our work. We have tried to address all the points raised by the reviewers and made the necessary revisions accordingly. We believe that, thanks to these comments, the revised manuscript is now stronger and more balanced.

Below are our point-by-point responses to each of the reviewers' comments. Reviewer's comments are written in black colored text, and the Authors' responses are written in blue colored text.

Reviewer #1 (Remarks to the Author):

In this study, Sharma et al focused on the role of SIRT2 in cardiac hypertrophy growth and protein synthesis in the heart. The authors demonstrated that SIRT2 may directly deacetylate RheB and promote ubiquitination of the latter. As a result, mTOR signaling is diminished, and cardiomyocyte growth is attenuated. A number of biochemical and cellular biology methods have been employed. In addition, both KO and TG mice were used. Overall, this is a solid study with convincing evidence and conclusions.

1. The authors reported in 2018 that SIRT2 deacetylates NFATc2, and this process is involved in cardiac hypertrophic growth (JBC). It is unclear to this reviewer how this mechanism is linked to the current SIRT2-RheB-mTOR axis. Would both mechanisms contribute to the growth phenotype? Which is major?

Authors' response:

We appreciate the reviewer for raising this important and insightful question regarding the contribution of two distinct mechanisms (NFATc2 and the SIRT2-RheB-mTOR axis) to the hypertrophic growth phenotype. In our previous finding, we reported that SIRT2 deacetylates NFATc2, affecting its nuclear localization and transcriptional activity. NFATc2 is a well-established transcriptional factor that regulates pathological cardiac hypertrophy by regulating the expression of several genes to promote growth in response to stresses such as pressure overload or neurohormonal stress (PMID: 18477567, PMID: 14656927, PMID: 29440391). In the current study, we report that the SIRT2-RheB-mTOR axis also contributes to the development of cardiac hypertrophy. While both mechanisms contribute to the development of a hypertrophic phenotype in SIRT2-depleted mice, SIRT2 acting on NFATc2 suggests a direct nuclear role in regulating hypertrophic gene expression during neurohormonal stress, whereas the SIRT2–Rheb–mTOR axis demonstrates a cytoplasmic/metabolic role, impacting protein synthesis and cell size. Mechanistically, both these mechanisms are independent of each other and regulate different aspects of hypertrophic phenotype.

NFATc2 activation is commonly associated with pathological cardiac hypertrophy, with no significant role in the development of physiological hypertrophy, such as cardiac hypertrophy during exercise or growth factor-induced cardiac growth (PMID: 18477567, PMID: 14656927). Rheb is a crucial protein for cardiac hypertrophic growth even in the absence of any pathological stimuli. A recent study has highlighted the importance of Rheb in the physiological adaptation of cardiomyocytes during exercise, where Rheb is transcriptionally upregulated in the hearts of exercised mice (PMID: 30582446). Furthermore, another study suggests that Rheb overexpression alone is sufficient for cardiac hypertrophic growth concomitant with increased mTORC1 activation; however, overexpression of Rheb alone does not induce expression of hypertrophic mRNA markers like brain natriuretic peptide (BNP) and atrial natriuretic factors (ANP), which are commonly upregulated in the case of NFATc2-associated hypertrophic changes (PMID: 18722381). Furthermore, studies have shown that Rheb levels are upregulated in pressure overload and aging-induced hypertrophic heart (PMID: 30582446, PMID: 31372634).

Although a direct relationship between SIRT2–Rheb–mTOR and NFATc2 activation in the development of cardiac hypertrophy in SIRT2-depleted mice hearts is not proven here, our results suggest that multiple regulators of cardiac hypertrophic growth are deregulated in SIRT2-depleted mice hearts. While NFATc2-mediated hypertrophic changes in these mice hearts are mainly associated with pathological stimuli, such as isoproterenol treatment (PMID: 29440391), persistent upregulated mTORC1 activity and increased protein synthesis in SIRT2-depleted mice hearts cause hypertrophic growth even in the absence of any external stimuli.

We have incorporated additional text about Rheb's role in cardiac hypertrophic development in the discussion section of the manuscript (manuscript lines 486-498). Additionally, to add more hypertrophy-related data, we have also added extra data demonstrating hypertrophic mRNA marker levels in *Sirt2*-KO mice (Figure 6O), along with representative images of mouse hearts showing hypertrophic changes (Figure 6J). (manuscript lines 400, 402, and 407-409)

2. The authors characterized mouse hypertrophy phenotype in KO and TG mice. However, it would be more informative if cardiac function can be assessed. In this regard, pathological stimuli, such as TAC, may be required.

Authors' response:

We appreciate the reviewer for this constructive suggestion. We have performed echocardiographic analysis to assess the cardiac function of *Sirt2*-KO (Figures 6F-H, Figures S5 A-T) and csSIRT2-TG mice (Figures 9F-I). The cardiac phenotype under pathological stress in *Sirt2*-KO and csSIRT2-TG mice is well-documented. A study from our lab has shown that SIRT2-depleted mice exhibit increased cardiac hypertrophic growth in response to isoproterenol treatment (PMID: 29440391) (manuscript line 480-482). Another study has shown that *Sirt2*-KO mice are more susceptible to Angiotensin II-induced hypertrophy, and cardiac-specific overexpression of SIRT2 is protective against Angiotensin II-induced hypertrophy in mice hearts (PMID: 28947430). (manuscript line 501-503)

Although SIRT2's role in the regulation of hypertrophic growth in the heart under pathological stress is well documented, how *Sirt2*-KO mice hearts respond to rigorous exercise training remains largely unexplored. Since Rheb has been reported as a crucial protein during exercise-induced cardiac remodelling, we subjected *Sirt2*-KO mice to 1 month of rigorous exercise training (physiological stress) (Figure 8A) and assessed their cardiac function using echocardiography. We have observed that the cardiac functions of WT running mice remained preserved, whereas *Sirt2*-KO mice under exercise training exhibited decreased Fractional shortening and increased HW/TL (Figures 8B-C), along with upregulated expression of the hypertrophy mRNA marker ANP (Figure 8D), suggesting that *Sirt2*-KO mice exhibit malfunctioned cardiac adaptation in response to physiological stress. (manuscript line 437-451)

3. To assess mTOR signaling, the authors mainly examined mTOR phosphorylation. What about phosphorylation of S6K1, S6, 4EBP1, and AMPK?

Authors' response:

We appreciate the reviewer's concern regarding the incomplete assessment of mTORC1 activation. We have now assessed the phosphorylation of p70S6K, which remains upregulated in *the hearts of Sirt2*-KO mice (Figure 6E). We also report significant downregulation of AMPK phosphorylation in *Sirt2*-KO mice hearts (Figure S6A-B), suggesting that AMPK (mTORC1 signalling inhibitor) is inhibited in *Sirt2*-KO mice hearts. Since 4EBP1 is also known to be regulated by SIRT2, we have checked 4EBP levels in the hearts of *Sirt2*-KO mice and observed that 4EBP levels were downregulated in the hearts of *Sirt2*-KO mice (Figure S3C). Altogether, these findings suggest that *Sirt2*-KO mice hearts exhibit increased mTORC1 activity concomitant with upregulated protein synthesis. (manuscript line 388-395)

Moreover, we have also assessed S6 phosphorylation in neonatal rat cardiomyocytes (NRCMs) to evaluate mTORC1 activation upon AGK2 treatment, and we report that S6 phosphorylation is significantly upregulated in AGK2-treated NRCMs (Figure 7B). (manuscript line 419-421)

Reviewer #2 (Remarks to the Author):

The manuscript by Shrama et al. shows that SIRT2, a member of the Sirtuin family of deacetylases, targets lysine-151 of the small GTPase protein Rheb for deacetylation. Acetylation of Rheb at K151 is suggested not to affect GTP binding, GDP dissociation, or membrane binding, but allows for its accumulation to stimulate mTOR activity and cap-dependent translation. Deacetylation of Rheb by SIRT2 results in ubiquitin-dependent degradation of Rheb, reduced mTOR activity, and reduced protein synthesis. Finally, the authors show that hypertrophic hearts from SIRT2-KO mice display increased protein synthesis due to increased Rheb levels and increased mTOR activity. With just a few exceptions, the studies carried out by Shrama et al. appear highly rigorous, and their results support the major conclusions of the manuscript. The key finding that Rheb is a major substrate for SIRT2, regulating protein synthesis, is highly significant and will be of general interest to the translation field. Some additional major and minor comments on the manuscript are listed below.

Major comments:

1. Fig. 1D, this panel shows increased expression of acetylated-tubulin following treatment with the SIRT2 inhibitor AGK2, but is not specifically referred to or explained in the text.

Authors' response:

We appreciate the reviewer's concern regarding the incomplete information about the increased acetylation of tubulin. Although this is shown in Fig. 1C instead of 1D, we verified tubulin acetylation to confirm whether SIRT2 inhibition by AGK2 was effective. We have now added this crucial information to the main text (manuscript lines 151-152).

2. In Fig. 1G, overexpression of wild-type SIRT2 results in a significant increase in puromycin labeling, suggesting increased protein synthesis. In Fig. 1J, overexpression of wild-type SIRT2 does not result in a significant increase in puromycin labeling. What is the difference between these two experiments, and how do the authors explain this apparent discrepancy?

Authors' response:

We appreciate the reviewer for raising this important and insightful question regarding the discrepancy between the outcomes of these experiments. The use of different statistical tests for calculating statistical significance in these experiments is causing this discrepancy. For Figure 1G, we used Welch's t-test (Two Groups), and the observed 20% decrease is statistically significant. However, in Fig. 1J, we have used one-way ANOVA (Three groups). Now, although the effective difference between pcDNA and wild-type SIRT2 is still like what we have observed in Fig. 1G, ANOVA didn't call it significant since ANOVA controls for family-wise error across all groups, so the threshold for significance is stricter. The addition of group SIRT2-N168A increases variance in the model, providing a larger error term and reduced power to detect differences between pcDNA and wild-type SIRT2.

3. In Fig. 1J and K, overexpression of catalytically inactive SIRT2 (N168A) significantly increased puromycin incorporation, but in Fig. 2D, overexpression of the same mutant had no effect on cap-dependent translation. Please explain this apparent discrepancy.

Authors' response:

We appreciate the reviewer for raising this important and insightful question regarding the discrepancy between the outcomes of these experiments. The observed discrepancy between these two experimental outcomes may be due to the different experimental approaches used to determine global

translation. In the puromycin incorporation experiment, puromycin was added to the media for only 30 minutes. The observed outcome illustrates the translation difference between these groups during the 30-minute period of puromycin incorporation. In contrast, in the cap-dependent translation experiment, the reporter plasmid (EMSV Bicis Luc) was transfected for 24 hours, and the calculated luciferase activity represents the cumulative cap-dependent translation that occurred within the 24-hour time frame and may have approached its saturating limits. Therefore, it is possible that the difference we observed in the short term (30 minutes) in the case of puromycin incorporation may not yield an exactly similar outcome as the cap-dependent translation experiment.

4. The manuscript shows increased Rheb levels in heart tissue from SIRT2-KO mice, consistent with the idea that deacylation of Rheb by SIRT2 results in its degradation. Are the increased levels of Rheb in the heart acetylated?

Authors' response:

We thank the reviewer for emphasizing the importance of assessing Rheb acetylation in SIRT2-KO mouse hearts. Our cell culture experiments have clearly demonstrated that Rheb is more acetylated under SIRT2-depleted conditions, resulting in Rheb stabilization. We have also observed significantly upregulated Rheb levels in SIRT2-KO mice hearts when compared with WT animals (We have now increased the number of animals to 8 per group to ensure this observation incorporates more animals) (Figures 6C-D). To determine if the upregulated Rheb levels in mouse hearts are more acetylated, we attempted to pull down lysine-acetylated proteins from WT and SIRT2-KO mice hearts. However, we were unable to detect Rheb in the pulldown fraction, possibly due to an issue with the antibody. Additionally, it is worth noting that increased levels of endogenous IgG in mouse heart tissue may also interfere with the detection of Rheb acetylation in immunoprecipitation assays by affecting the sensitivity and specificity of acetylation detection on Rheb, particularly for low-abundance post-translational modifications such as acetylation, which can make detection difficult. However, to check if SIRT2 inhibition promotes Rheb stability in cardiomyocytes, we treated NRCMs with AGK2 to inhibit SIRT2 deacetylation function. We observed an increase in Rheb levels in AGK2-treated NRCMs (Figure 7E), suggesting that Rheb regulation by SIRT2 is a conserved mechanism across different cell types. (manuscript lines 384-387 and 431-434)

5. Figures 6F-I are not sufficiently explained for the reader.

Authors' response:

We appreciate the reviewer's concern regarding the incomplete information about echocardiography panels depicting the cardiac functions of SIRT2-KO mice and the heart weight-to-tibia length ratio, which demonstrates increased hypertrophic growth in the hearts of SIRT2-KO mice, and thank you for highlighting this issue. We have now incorporated adequate information about these panels into the main text. (manuscript line 397-401)

Minor comments:

1. Line 217, deacylase should be deacylated

Authors' response:

We thank the reviewer for bringing this to our attention. We have now changed the sentence from "SIRT2 can deacylase multiple small GTPases" to "SIRT2 can deacylate multiple small GTPases" (manuscript line 218)

2. Line 261; the authors find that knockdown of Rheb expression using siRNA blocks the ability of AGK2 inhibitor of SIRT2 to stimulate cap-dependent translation. The sentence describing this effect is worded a bit confusingly: "Furthermore, we observed that downregulating Rheb can rescue the increased cap-dependent translation under SIRT2-inhibited conditions. 262 [Figure 3K, L]."

Authors' response:

We thank the reviewer for bringing this to our attention. We have now rephrased this line to: "Also, we found that reducing Rheb levels can reverse the increase in cap-dependent translation induced by SIRT2 inhibition." (manuscript lines 262-264)

Reviewer #3 (Remarks to the Author):

Review of Manuscript EMBOR-2025-61644V1 by Shrama et al.

The manuscript presents novel findings suggesting that SIRT2 deficiency increases protein synthesis via a mechanism whereby SIRT2 binds to and deacetylates Rheb at K151 residue to enhance ubiquitin-proteasome-mediated degradation of Rheb.

The manuscript is of very good quality, with well-controlled experiments and mechanistic insights supporting the authors' conclusions. However, the points below should be addressed to strengthen the manuscript further.

- In figure 4C, in the HA IP blot section, the blot of HA (Rheb) shows 1 main band for HA. In the KAc blot, there are 3 bands shown, with one identified with an arrow, presumably meaning that it's Rheb. Can the authors comment on what the other 2 bands are? They also seem to decrease in the K151R mutant lane.

Authors' response:

We appreciate the reviewer for raising this important and insightful question. Only the band that was identified by the arrow is Rheb. The other bands are nonspecific bands that do not belong to Rheb. The lysine-acetylation antibody is not a strong antibody, so it tends to pick up a lot of background noise. The identified Rheb band is determined based on the size of Rheb.

- In figs. 2G and 6E, please indicate which phosphorylation site(s) are being shown.

Authors' response:

We thank the reviewer for bringing this to our attention. We have now indicated specific phosphorylation sites in the respective figures' legends, which are illustrated throughout the manuscript. It is certainly very important to include these specific sites, and we have ensured that the revised version will incorporate these additions.

- Could quantification of blots be provided for fig 6J (and the associated relative puromycin incorporation vs control blots).

Authors' response:

We appreciate the reviewer for this suggestion. We have now added quantification for the mentioned panel in the main manuscript (Figure 6N). (manuscript line 402)

- The term "protein translation" is found at several places in the document. Please replace with either "mRNA translation" or "protein synthesis".

Authors' response:

We thank the reviewer for bringing this to our attention. We have now changed the term "protein translation" to "protein synthesis" throughout the manuscript.

Reviewer #4 (Remarks to the Author):

This study reveals an important regulatory mechanism in protein synthesis control. Sharma et al. demonstrate how SIRT2 regulates global protein synthesis through deacetylation of Rheb GTPase.

Their work shows that SIRT2 removes specific chemical tags (deacetylation at K151) from Rheb, which promotes its ubiquitination and proteasomal degradation. This degradation reduces mTORC1 activity and subsequently suppresses global protein synthesis. The authors provide evidence that SIRT2-deficient mice develop age-associated cardiac hypertrophy with increased cardiac protein synthesis, while cardiac-specific SIRT2 overexpression reduces protein synthesis in mouse hearts.

The identification of Rheb as a SIRT2 substrate represents a significant advance in our understanding of translational control. However, three important conceptual gaps require attention before this manuscript is suitable for publication in EMBO Reports.

Major Concerns

1. Causal mechanism between deacetylation and ubiquitination

The authors demonstrate that SIRT2 removes acetyl groups from Rheb at position K151, which promotes ubiquitination and degradation. However, the mechanistic connection between these events remains unclear. Key questions need addressing:

- Which specific E3 ligase targets deacetylated Rheb for degradation?

- How does deacetylation create recognition sites for the ubiquitination machinery?

- What is the structural relationship between K151 deacetylation and the ubiquitination sites?

Authors' response:

We appreciate the reviewer for emphasizing the importance of assessing the causal mechanism between deacetylation and ubiquitination. Addressing these concerns would require extensive structural biology-based studies and is outside the scope of the current study; we would try to work on this aspect in our future studies.

2. Limited exploration of physiological relevance

The biological importance of this pathway requires stronger experimental support. While the authors demonstrate correlation between SIRT2 deficiency and cardiac hypertrophy, they haven't shown:

- Whether this pathway is essential for cardiac function under physiological stress conditions

Authors' response:

We appreciate the reviewer for raising this important and insightful question. Although SIRT2's role in regulating hypertrophic growth in the heart under pathological stress is well-documented, limited studies have emphasized the importance of SIRT2 in the heart during physiological stress. To assess the cardiac function under physiological stress, we utilized rigorous exercise as a model to induce physiological stress. The response of *Sirt2*-KO mice hearts to rigorous exercise training (physiological stress) remains less explored. Moreover, Rheb has been reported as a crucial protein during exercise-induced cardiac remodelling (PMID: 30582446). We subjected SIRT2-KO mice to 1 month of rigorous exercise training using a treadmill (physiological stress) (Figure 7A) and assessed their cardiac function using echocardiography. We have observed that the cardiac functions of WT running mice remained preserved, whereas SIRT2-KO mice under exercise training exhibited decreased Fractional Shortening and increased HW/TL (Figures 7B-C), along with upregulated expression of the hypertrophy mRNA marker ANP (Figure 7D), suggesting that SIRT2-KO mice exhibit malfunctioned cardiac adaptation in response to physiological stress. (manuscript lines 437-451)

- How acute SIRT2 manipulation affects cardiac responses

Authors' response:

We appreciate the reviewer for raising this important and insightful question. We treated neonatal rat cardiomyocytes (NRCMs) with AGK2 for 24 hrs and assessed for hypertrophic changes. We observed a slight increase in ANP mRNA levels and a trend of increased BNP levels (Figures 7C, D). Moreover, another hypertrophic phenotype, protein synthesis, was upregulated in cardiomyocytes treated with AGK2 concomitant with upregulated mTORC1 activity (Figures 7A, B). Furthermore, it was previously reported that acute SIRT2 manipulation in cardiomyocytes promotes a hypertrophic phenotype (PMID: 29440391). Another study has also emphasized that SIRT2 depletion/inhibition can exacerbate pathological stress-induced hypertrophy in cardiomyocytes (PMID: 28947430). Related texts have been added in (manuscript lines 412-436).

- Whether mTORC1 inhibition (e.g., rapamycin treatment) can rescue SIRT2-deficient cardiac phenotypes

Authors' response:

We appreciate the reviewer for raising this important question. To determine if Rapamycin treatment can reverse the SIRT2-deficient cardiac phenotype, NRCMs inhibited for SIRT2 were treated with Rapamycin, and the effectiveness of this treatment was assessed by examining S6 phosphorylation. We observed increased puromycin incorporation in SIRT2-inhibited NRCMs; however, SIRT2-inhibited NRCMs treated with Rapamycin do not exhibit an increase in puromycin incorporation. This observation suggests that Rapamycin treatment can rescue against SIRT2 inhibition-induced protein synthesis in cardiomyocytes (Figure 7A). (manuscript lines 421-424)

3. Incomplete characterization of SIRT2-Rheb interaction with mTORC1 signaling

The study needs to address how this newly discovered mechanism integrates with known regulatory pathways. Critical questions include:

- How does Rheb acetylation status affect its interaction with mTORC1?

Authors' response:

We appreciate the reviewer's concern regarding the incomplete characterization of SIRT2-Rheb interaction with mTORC1 signaling. Various studies have shown that overexpression of Rheb itself is sufficient to activate mTORC1 kinase activity (PMID: 30582446, PMID: 26092121, PMID: 18722381, PMID: 19258434, PMID: 12906785). The mechanism by which Rheb activates mTOR is not yet fully understood. While several mechanisms have been proposed, the exact pathway by which Rheb activates mTORC1 remains unclear. It is likely that multiple factors, including direct interactions, inhibition of endogenous inhibitors, lipid signaling, and nutrient sensing, contribute to the activation of mTORC1 by Rheb. Further research is needed to elucidate the precise molecular details of this regulation.

To investigate whether deacetylation of Rheb at lysine 151 affects its ability to activate mTOR, we examined mTOR phosphorylation and cap-dependent translation. Our data indicate that overexpression of the wild-type (WT) Rheb results in significantly increased mTOR phosphorylation and upregulated cap-dependent translation, whereas overexpression of the deacetylation-mimic mutant (K151R) does not show any change (Figures S2F, G), (Figure 4I). Additionally, to examine whether lysine 151 acetylation-mediated structural changes play a role in GTP binding, GDP release, or Rheb's localization to the lysosomal membrane, we have previously performed NMR experiments (Figures 4F, G), pulling force simulations (Figure 4I), and molecular dynamics simulation studies (Figures 5A-C), respectively. Outcomes of these experiments have suggested that these aspects of Rheb are not influenced by lysine 151 acetylation. Our results indicate that the increased mTORC1 activity is due to the upregulated Rheb levels under SIRT2-depleted/inhibited conditions, as ubiquitination-mediated degradation is suppressed under these conditions. Moreover, studies have shown that Rheb

overexpression alone can promote growth in cardiomyocytes concomitant with increased mTORC1 activity (PMID: 18722381, PMID: 30582446). Altogether, the mentioned literature as well as our current findings strongly suggest that an increase in Rheb levels is sufficient to activate mTORC1 kinase activity and protein synthesis, which can promote cardiomyocyte growth. (manuscript lines 297-348)

- Does acetylation influence other Rheb post-translational modifications?

Authors' response:

We appreciate the reviewer for raising this important question. Based on the available reports, Rheb has 4 post-translational modifications which are reported across different studies: a) Ubiquitination, b) Acetylation, c) acylation, and d) phosphorylation. In our current study, we have demonstrated that SIRT2 inhibition does not affect Rheb acylation (Figure S2A), whereas Rheb acetylation is increased. Our data have shown that acetylation decreases Rheb ubiquitination, suggesting that Rheb ubiquitination is influenced by its acetylation. However, in the current study, we did not check for Rheb phosphorylation since we couldn't find any commercially available antibody against phosphorylated Rheb. (manuscript lines 227-236)

- How do cells coordinate these different regulatory mechanisms during stress?

Authors' response:

We thank the reviewer for the opportunity to clarify how SIRT2-mediated regulation integrates with established stress-responsive pathways to coordinate global protein synthesis. During cellular stress, particularly in the context of nutrient deprivation, translation is tightly controlled through multiple interconnected layers. Canonical upstream nutrient sensors, such as AMPK and TSC1/2, inhibit mTORC1 signaling, reducing the phosphorylation of key effectors (e.g., S6K, 4EBP1) and lowering protein synthesis (PMID: 18439900; PMID: 12906785). Given SIRT2's dependence on NAD^+, its activity is linked to the cellular metabolic state (PMID: 17521387), providing a sensor mechanism that couples energy metabolism to translational control. Moreover, starvation triggers the dephosphorylation and activation of SIRT2 in a cyclin E/CDK2 suppression-dependent manner, inducing autophagy and thereby linking cellular energy status to SIRT2 activation (PMID: 35921421). Our work adds another regulatory role for SIRT2 in this context. Importantly, we demonstrate that SIRT2 directly interacts with and deacetylates Rheb, a small GTPase essential for mTORC1 activation. Deacetylation of Rheb by SIRT2 promotes its ubiquitin-dependent degradation, thereby suppressing mTORC1 activity at the level of its activator. This represents a novel post-translational regulatory layer that complements the upstream kinase-dependent inhibition of mTORC1.

We were interested in understanding if this regulation is relevant during physiological stress in cells. Recently, it was reported that Rheb undergoes K48-linked polyubiquitination under low glucose availability, leading to the inactivation of mTORC1 (PMID: 38862475), suggesting the essentiality of regulating Rheb levels and activity during glucose starvation. To investigate whether SIRT2 levels or activity are affected during glucose starvation, we subjected different cell types to glucose deprivation and assessed Rheb levels, SIRT2 levels, and acetylated α-tubulin levels (as an indicator of SIRT2 activity). Our findings demonstrate that Rheb levels were significantly downregulated in primary cardiomyocytes isolated from hearts of neonatal rats (NRCMs) [Figure 3M, N]. Although we did not observe changes in SIRT2 protein levels, SIRT2 activity was significantly upregulated in neonatal rat cardiomyocytes (NRCMs) subjected to glucose deprivation [Figures 3M and O]. Glucose deprivation was validated by increased phosphorylation of AMPK in cardiomyocytes cultured in glucose-free media [Figure 3M]. Similar findings were observed in HEK293T cells, indicating a conserved response across cell types [Figure S2B]. (manuscript lines 264-290)

Additionally, we also assessed previously published RNA-seq data to check if Rheb is transcriptionally regulated under nutrient starvation and found no significant changes in Rheb expression under 4 hours of HBSS starvation in HeLa cells and 6 hours of EBSS starvation in Human HAP1 cells, suggesting that Rheb regulation during starvation doesn't occur at transcription levels (Figures S2D, E). (manuscript lines 290-296)

Moreover, our companion manuscript also provides insight into how, under amino acid starvation, SIRT2 deacetylates and stabilizes 4EBP1, reinforcing translational repression. Together, these SIRT2-dependent mechanisms coordinate to provide both rapid and sustained repression of translation, integrating stress signals at multiple points to enhance the robustness of the cellular response. Additional stress-responsive pathways also contribute to this network. For example, the Integrated Stress Response (ISR) via eIF2α phosphorylation modulates selective translation under various stresses (PMID: 36943285), and autophagy induction, regulated by the SIRT2-ATG4B axis during starvation, plays a role in proteostasis and energy homeostasis (PMID: 35921421). (manuscript lines 533-548)

In summary, SIRT2 functions as a key integrator of stress-responsive regulatory pathways, modulating both mTORC1 activity and translation effectors to fine-tune protein synthesis during stress, alongside other well-characterized nutrient and energy-sensing mechanisms.

Minor Concerns

The specificity of SIRT2's effects on cardiac tissue requires additional validation through testing in other tissues or cell types. Additionally, the authors should address how this mechanism relates to other known SIRT2 functions in metabolic regulation, including potential cross-talk between pathways.

We appreciate the reviewer for raising this important question. Although we have used cardiac hypertrophy as a model to understand the physiological relevance of SIRT2-mediated control of protein synthesis, our data suggest that SIRT2-mediated regulation of protein synthesis is not specific to cardiomyocytes. We have assessed protein synthesis rate, mTORC1 activation concomitant with increased Rheb levels in primary cardiomyocytes, heart tissue, HEK293T cells, as well as in HeLa cells, and observed similar regulation. Moreover, the regulation of Rheb levels during glucose starvation appears to be conserved in these different cell types (Figure 3M, Figure S2B). (manuscript lines 279-290)

Additionally, in the companion manuscript by Prof. Lin, a similar phenotypic effect of protein synthesis regulation has been observed across multiple cell lines, further strengthening the key finding of this study that SIRT2-mediated negative regulation of protein synthesis is a conserved regulatory mechanism independent of its cell-specific effect.

Regarding addressing how this mechanism relates to other known SIRT2 functions in metabolic regulation, including potential cross-talk between pathways, our response to major comment 3 from reviewer 4, entitled “How do cells coordinate these different regulatory mechanisms during stress?” addresses this concern.

Additional comments from referee 4:

I'm including some comments directly to you, considering them both together. For my nomenclature: Paper 1 = Zi et al. and Paper 2 = Sharma et al.

Overall, these companion papers present disconnected mechanisms for SIRT2's regulation of protein translation without adequate integration. For example, Paper 1 demonstrates SIRT2 deacetylates 4EBP1 downstream of mTORC1, while Paper 2 shows SIRT2 deacetylates Rheb upstream of mTORC1-with both ultimately suppressing translation; despite shared authorship, the manuscripts fail to reconcile these pathways, explain their relative importance in different contexts, or investigate potential competition between substrates; neither paper presents a unified model showing how these mechanisms interact during stress responses or in cardiac hypertrophy, leaving readers without a comprehensive understanding of SIRT2's role in translation regulation. So while both papers offer valuable insights into SIRT2's role in regulating protein translation, they present largely disconnected

mechanisms without adequately addressing how these pathways interact. This represents a significant missed opportunity to develop an integrated framework that would better explain SIRT2's biological functions and potential therapeutic applications. For back-to-back publications, there's a surprising lack of integration that would provide a more comprehensive understanding of SIRT2's role in translation regulation. Here are some specific points:

1. Different Mechanisms for the Same Outcome

Both papers describe SIRT2's role in suppressing protein translation, but through entirely different mechanisms:

- Paper 1 claims SIRT2 deacetylates 4EBP1, stabilizing it and preventing eIF4E from initiating translation

- Paper 2 claims SIRT2 deacetylates Rheb, promoting its degradation and reducing mTORC1 activity

While complementary mechanisms are possible, neither paper acknowledges the alternative pathway described by the other, which is peculiar for back-to-back publications.

We appreciate the reviewer for raising this important question. We have now added additional text information acknowledging the alternative pathway described in our companion manuscript. (manuscript lines 533-548)

2. Insufficient Integration of Findings

Paper 2 briefly mentions Paper 1 in a single sentence: "Interestingly enough, in another manuscript under submission by Prof. Hening Lin's lab, SIRT2 is shown to deacetylate 4EBP1 leading to its stabilization, thereby suppressing translation by inactivating mTORC1."

However, this integration is minimal and doesn't address:

- The relative importance of each mechanism

- Whether these mechanisms operate simultaneously or in different contexts

- How these mechanisms interact in the regulatory network

We thank the reviewer for highlighting this important point. We agree that, taken in isolation, the two manuscripts describe distinct mechanisms by which SIRT2 suppresses protein synthesis. However, rather than being contradictory, these findings are complementary.

The accompanying manuscript establishes that SIRT2 deacetylates 4E-BP1, decreasing its phosphorylation competence and thereby stabilizing its inhibitory interaction with eIF4E. In the current study, we show that SIRT2 also deacetylates Rheb at K151, leading to its destabilization and reduced mTORC1 activity. Together, these findings reveal that SIRT2 suppresses translation through multiple mechanisms. It is quite general that PTM enzymes regulate a pathway via multiple substrates. For example, mTORC1 promotes translation by phosphorylating both 4E-BP1 and S6K. It is possible that in the future, new substrate proteins of SIRT2 are discovered that also contribute to the regulation of translation.

We acknowledge that the original versions of each manuscript did not explicitly reference the other pathway. We have now revised the Discussion section to highlight this integration and to propose that SIRT2 functions as a multi-node regulator of translational control. We believe SIRT2 suppresses translation via multiple mechanisms to ensure tight regulation, just like mTORC1 regulates translation via multiple substrates.

We demonstrate that the hearts of Sirt2-KO mice also exhibit decreased 4EBP levels (Figure S6C), indicating that multiple proteins involved in mTORC1 signalling are deregulated in these mice, which contributes to the increased protein synthesis and development of cardiac hypertrophy. We have also assessed S6 Ribosomal protein (S6-RP) phosphorylation as well as 4EBP protein levels under EBSS-treated conditions in NRCMs, and our data indicate that S6-RP phosphorylation was decreased upon EBSS treatment, suggesting mTORC1 inactivation. Additionally, we observed upregulated 4EBP levels (Figure S2C), stressing the involvement of both mechanisms. We believe these mechanisms operate simultaneously. However, definitive proof, as well as an understanding of how these mechanisms interact, deserves its own separate study. manuscript lines 533-548

3. Conflicting Statements About mTORC1 Inactivation

The papers present different accounts of how SIRT2 affects mTORC1:

- Paper 1 suggests SIRT2 acts downstream of mTORC1 by stabilizing 4EBP1 (which is a target of mTORC1)

- Paper 2 claims SIRT2 acts upstream of mTORC1 by destabilizing Rheb (which activates mTORC1)

Paper 2's brief mention that SIRT2's 4EBP1 deacetylation "suppress[es] translation by inactivating mTORC1" contradicts Paper 1, which doesn't claim 4EBP1 inactivates mTORC1 but rather acts downstream of it.

We thank the reviewer for bringing this to our attention. We have now changed this contradictory statement from "SIRT2's 4EBP1 deacetylation suppresses translation by inactivating mTORC1" to SIRT2's 4EBP1 deacetylation suppresses translation by stabilizing 4EBP1. (manuscript lines 535-536)

We hope that our replies to the additional comments (#1 and #2 above) will address that the findings in the two papers do not contradict each other. Instead, we believe that SIRT2 controls translation through multiple mechanisms. Together, these mechanisms coordinate to provide both rapid and sustained repression of translation, integrating stress signals at multiple points to enhance the robustness of the cellular response during nutrient stress

4. Incomplete Physiological Context

Both papers examine SIRT2's role in cardiac hypertrophy:

- Paper 1 only mentions it briefly, and states SIRT2 inhibitors have anticancer activity

- Paper 2 provides more detail about SIRT2-KO mice developing cardiac hypertrophy

However, neither paper fully integrates both mechanisms to explain the development of cardiac hypertrophy. How do the 4EBP1 and Rheb pathways together contribute to hypertrophy? Which pathway predominates in this condition?

We thank the reviewer for bringing this to our attention. We would like to address that both papers do not examine SIRT2's role in cardiac hypertrophy; the examination of SIRT2's role in cardiac hypertrophy is solely addressed in Paper 2. The key point to emphasize via this study is the contribution of abnormal mTORC1 activity and upregulated protein synthesis in the development of cardiac hypertrophy. We observed increased Rheb levels in the hearts of Sirt2-KO mice. Rheb overexpression is reported to

induce cardiac hypertrophic growth concomitant with increased mTORC1 activation (PMID: 18722381). We also assessed 4EBP levels and observed that 4EBP levels were low in the hearts of SIRT2-depleted mice (Figure S6C). Our findings suggest that multiple effectors of the protein synthesis regulatory pathway are deregulated in the hearts of Sirt2-KO mice, which contributes to the upregulated protein synthesis and further influences the hypertrophic phenotype.

Together, these complementary papers highlight different but synergistic layers of SIRT2 function in regulating translation, both at the upstream level through Rheb and at the downstream level through 4E-BP1. (manuscript lines 384-395)

5. Inadequate Comparative Analysis Under Stress Conditions

Both papers examine SIRT2 under stress conditions:

- Paper 1 focuses on amino acid limitation

- Paper 2 examines complete starvation (EBSS)

A systematic comparison of how these different stress conditions affect the two regulatory mechanisms (4EBP1 vs. Rheb) is missing. Do different types of nutrient stress preferentially activate one pathway over the other?

Our data indicate that both EBSS treatment and amino acid limitation ultimately converge on the same outcomes: upregulation of SIRT2 protein level. Additionally, we have also checked for SIRT2 activity that was upregulated upon EBSS treatment. Once SIRT2 levels and activities are elevated, its enzymatic activity is expected to act on multiple substrates, consistent with the typical mode of action of PTM enzymes. Therefore, we believe both Rheb and 4E-BP1 pathways are engaged under these conditions. Moreover, we have assessed for S6 Ribosomal protein (S6-RP) phosphorylation as well as 4EBP protein levels under EBSS-treated conditions in NRCMs, and our data indicate that S6-RP phosphorylation was decreased upon EBSS treatment, suggesting mTORC1 inactivation, whereas we also observed upregulated 4EBP levels (Figure S2C), indicating that both mechanisms are engaged under EBSS starvation conditions. While our study focused on complete starvation (EBSS) and the accompanying manuscript focused on Amino acid limitation, together our results suggest that SIRT2 functions as a stress-responsive regulator that can integrate nutrient stresses through multiple downstream effectors. Together, these mechanisms coordinate to provide both rapid and sustained repression of translation, integrating stress signals at multiple points to enhance the robustness of the cellular response during nutrient stress. (manuscript lines 541-548)

6. Experimental Design Differences

The papers use different experimental approaches:

- Paper 1 primarily uses amino acid deprivation experiments

- Paper 2 mainly uses SIRT2 inhibition, knockdown, and overexpression

While complementarity could be a strength, these differing approaches make direct comparison of results impossible, and neither paper attempts to reconcile methodological differences.

Both papers have used SIRT2 inhibition, knockdown, and overexpression to examine the effect of SIRT2 on translation and substrate acetylation/degradation. Again, we emphasize that the two papers are complementary instead of conflicting with each other.

7. Shared Authorship Without Integrated Discussion

Despite having some shared authors (including Yanlin Zi and Hening Lin), the papers don't present a unified conceptual framework. This raises questions about whether these are truly complementary studies or competing models that haven't been fully reconciled.

We thank the reviewer for raising this important question. We evaluated 4EBP levels in the hearts of mice to integrate the findings from both studies and provide a more comprehensive interpretation. We believe that the revised manuscript now effectively reconciles and unifies the conclusions of these studies. We have incorporated an expanded discussion in the revised manuscript that addresses this concern.

Dear Dr. Sundaresan

Thank you for the submission of your revised manuscript. We have now received the enclosed reports from the referees that were asked to assess it. Referee 4 still has a few more suggestions that I would like you to address and incorporate before we can proceed with the official acceptance of your manuscript. Please co-submit a point-by-point response to all final comments.

Regarding the back-to-back publication, I would like to clarify that you will not need to experimentally integrate both papers, however, the discussion needs to be improved in this regard. At EMBO reports related papers can just be linked, which is different from a "true back-to-back publication" as mentioned by referee 4. In case there are obvious and straight forward experiments that can be done to increase our understanding of how both papers/mechanisms could be linked, these should be performed of course.

A few editorial requests will also need to be addressed:

- A Data Availability Section needs to be added to the end of the Methods. If no data have been deposited in public databases, you only need to add the following sentence to the DAS: "This study includes no data deposited in external repositories"
The other sections (Resource availability, Lead contact, Materials Availability) need to be deleted.

- The conflict of interest subheading needs to be renamed to "Disclosure and Competing Interests Statement" and the one provided on the title page needs to be removed.

- The author credits need to be removed from the ms file. All credits are entered during online ms submission.

- The REFERENCE format needs to be corrected: et al needs to be used after 10 author names. Please use the EMBO reports reference format.

- The ANRF CRG/2023/000561 and the MHRD Government of India funding info are missing in our online submission system and need to be entered as separate funders. The funding info in the ms file should be part of the Acknowledgement section, so no "Funding Sources" title is needed.

- All figures need to be uploaded as individual figure files. We do not need any figures provided in zip folders, these two folders need to be removed.

- All supplementary figures should be uploaded as EV figures. You can have up to 8 EV figures. Please correct all file names and ms callouts. You can find more information on our figure types in our guide to authors online:
https://link.springer.com/journal/44319/submission-guidelines#cms-Manuscript-organisation-and-formatting

- For the Reagents & Tools table please use the template file from our guide to authors. All primer info should also be within the table.

- The Source Data should be uploaded as 1 file per main figure.

- Materials and methods should be "Methods"

- Please note that the exact p values are not provided in the legends of figures 1E, 2C, 3K, 6L, 7B, please provide exact values as reasonable.

I would like to suggest some minor changes to the title to make it more specific. Please let me know what you think:

Sirtuin 2 inhibits global protein synthesis via Rheb-GTPase degradation

EMBO press papers are accompanied online by A) a short (1-2 sentences) summary of the findings and their significance, B) 2-3 bullet points highlighting key results and C) a synopsis image that is exactly 550 pixels wide and 200-600 pixels high (the height is variable). The synopsis image should provide a sketch of the major findings, like a graphical abstract. Please note that text needs to be readable at the final size. Please send us this information along with the final manuscript.

I look forward to seeing a final version of your manuscript as soon as possible.

Please do not hesitate to contact me if you have any questions.

Best regards,
Esther

Esther Schnapp, PhD
Senior Editor
EMBO reports

Referee #2:

EMBOR-2025-61644V2 Review

This revised manuscript from Sharma et al. investigates the role of SIRT2 in regulating Rheb acetylation, specifically at lysine-151. The authors propose that acetylation of Rheb promotes its accumulation and consequently enhances mTOR signaling and cap-dependent translation, thereby contributing to cardiomyocyte growth and cardiac hypertrophy. In the prior version of the manuscript, several minor concerns were raised regarding insufficient description of certain data and apparent inconsistencies across a few of the experiments. These issues appear to have been satisfactorily addressed in the current revision.

Referee #3:

All the modifications I suggested were addressed in a satisfactory manner.

Referee #4:

Summary
This manuscript identifies Rheb as a SIRT2 substrate, showing that K151 deacetylation promotes Rheb degradation, suppresses mTORC1, and reduces protein synthesis. SIRT2-KO mice develop cardiac hypertrophy with increased synthesis, while cardiac-specific overexpression has the opposite effect. The work is technically sound with good in vivo validation, but significant conceptual issues limit its impact.

Strengths
Novel, well-validated substrate identification
Comprehensive mechanistic studies (NMR, MD simulations, functional assays)
Strong physiological relevance with multiple mouse models
Improved rigor in revision (replicates, statistics, echocardiography)

Remaining Major Concerns
1. The 4EBP1 Data Raises New Questions
Important observation: Figure S6C shows 4EBP1 levels are LOW in SIRT2-KO hearts, which is consistent with the companion manuscript showing SIRT2 stabilizes 4EBP1.
However, this creates a puzzle for your model: If 4EBP1 is low in SIRT2-KO hearts, how does increased protein synthesis occur? Your model proposes that increased Rheb → mTORC1 activation drives translation. But with less 4EBP1 available to be phosphorylated and inactivated, there should be MORE active 4EBP1 suppressing translation, not less.

2. Minimal Experimental Integration with Companion Manuscript
You've added a Discussion paragraph but no cross-substrate experiments.

3. Cardiac Hypertrophy Model Is Now Confusing
You cite previous work showing SIRT2 regulates NFATc2 (pathological hypertrophy), now add Rheb-mTORC1, and the companion adds 4EBP1. Which matters most? When? The Discussion lacks a clear framework explaining how these mechanisms integrate rather than listing them separately.

Minor
E3 ligase: I understand identifying this is beyond scope, but you should explicitly acknowledge this gap and propose candidates based on Rheb biology.
Rheb acetyltransferase: Similarly important for completing the regulatory cycle. Your glucose starvation data (SIRT2 activity increases) suggests SIRT2 is the regulated node, but this needs discussion.
Minor technical issues: AMPK data needs more replicates (Fig S6A-B), echocardiography needs clearer sample size reporting, exercise experiments need proper controls (WT sedentary vs. running).

More comments from referee 4:

These manuscripts should be published separately, not as integrated companion papers. While both describe SIRT2 regulating translation, they actually represent distinct stress-response pathways rather than a unified mechanism. Zi et al. show amino acid starvation increases SIRT2 protein levels (via ISR-mediated translation), which then stabilizes 4EBP1. Sharma et al. show glucose starvation increases SIRT2 activity (not protein), which degrades Rheb and inhibits mTORC1. These are fundamentally different regulatory contexts engaging SIRT2 through different mechanisms.

More importantly, there's no experimental evidence that the pathways work together. Despite reviewer requests, neither manuscript measures both substrates under the same conditions, tests substrate competition, or performs epistasis experiments. The "integration" exists only in the Discussion paragraphs, using nearly identical language about PTM enzymes having multiple substrates. The in vivo data from Sharma et al. actually suggest Rheb regulation dominates-SIRT2-KO hearts have high Rheb, low 4EBP1, yet still show increased protein synthesis and hypertrophy. If 4EBP1 stabilization were equally important, low 4EBP1 levels should provide some protection, but they don't.

The one finding that could meaningfully link them-Zi et al. Figure R8 showing K69 acetylation increases 4EBP1's phosphorylation competence-is buried in revision responses rather than integrated into either narrative. This could explain why both upstream (Rheb) and downstream (4EBP1) regulation matter, but neither manuscript develops this insight.

I would recommend publishing these as independent studies, each acknowledging the other exists but not claiming functional integration that isn't experimentally demonstrated. For true back-to-back publication, you would need at minimum: one experiment testing both substrates under the same stress conditions, an epistasis test showing whether 4EBP1 overexpression rescues SIRT2-KO phenotypes, and a unified model explaining when and where each mechanism predominates. Without this, they're two separate papers about SIRT2, not a coordinated study of multi-substrate regulation.

Response to editorial suggestions and reviewers comments

We thank the Editor for the helpful editorial suggestions and Reviewers for their constructive comments. Each point has been carefully considered, and the manuscript has been revised accordingly. Reviewer 4’s critical and insightful approach has substantially strengthened the discussion section, significantly enhancing the clarity and depth of the discussion. Below, we provide a detailed, point-by-point response to all comments, along with the corresponding manuscript lines where changes have been made.

A few editorial requests will also need to be addressed:

- A Data Availability Section needs to be added to the end of the Methods. If no data have been deposited in public databases, you only need to add the following sentence to the DAS: "This study includes no data deposited in external repositories"

Authors’ response:

We have incorporated this change

The other sections (Resource availability, Lead contact, Materials Availability) need to be deleted.

Authors’ response:

We have incorporated this change

- The conflict of interest subheading needs to be renamed to "Disclosure and Competing Interests Statement" and the one provided on the title page needs to be removed.

Authors’ response:

We have incorporated this change

- The author credits need to be removed from the ms file. All credits are entered during online ms submission.

Authors’ response:

We have incorporated this change

- The REFERENCE format needs to be corrected: et al needs to be used after 10 author names. Please use the EMBO reports reference format.

Authors' response:

We have incorporated this change

- The ANRF CRG/2023/000561 and the MHRD Government of India funding info are missing in our online submission system and need to be entered as separate funders. The funding info in the ms file should be part of the Acknowledgement section, so no "Funding Sources" title is needed.

Authors' response:

We have incorporated this change

- All figures need to be uploaded as individual figure files. We do not need any figures provided in zip folders, these two folders need to be removed.

Authors' response:

We have incorporated this change

- All supplementary figures should be uploaded as EV figures. You can have up to 8 EV figures. Please correct all file names and ms callouts. You can find more information on our figure types in our guide to authors online:

https://link.springer.com/journal/44319/submission-guidelines#cms-Manuscript-organisation-and-formatting

Authors' response:

We have incorporated this change

- For the Reagents & Tools table please use the template file from our guide to authors. All primer info should also be within the table.

Authors' response:

We have incorporated this change; primer and other oligonucleotides information is attached in "Table 1" embedded in the main text file of manuscript and highlighted in "Regents and Tools table" provided as separate word file.

- The Source Data should be uploaded as 1 file per main figure.

Authors' response:

We have incorporated this change

- Materials and methods should be "Methods"

Authors' response:

We have incorporated this change

- Please note that the exact p values are not provided in the legends of figures 1E, 2C, 3K, 6L, 7B, please provide exact values as reasonable.

Authors' response:

These indicated figures do not have an absolute p-value, even when expressed to 15 decimal points; therefore, we cannot provide exact p-values. However, we have provided all the statistical analysis in the source data file of the respective figures, including the original values plotted in the graph, along with the normality test and the respective t-test as required.

I would like to suggest some minor changes to the title to make it more specific. Please let me know what you think:

Sirtuin 2 inhibits global protein synthesis via Rheb-GTPase degradation

Authors' response:

This is a great suggestion. We thank you for this suggestion. We have revised the title accordingly

EMBO press papers are accompanied online by A) a short (1-2 sentences) summary of the findings and their significance, B) 2-3 bullet points highlighting key results and C) a synopsis image that is exactly 550 pixels wide and 200-600 pixels high (the height is variable). The synopsis image should provide a sketch of the major findings, like a graphical abstract. Please note that text needs to be readable at the final size. Please send us this information along with the final manuscript.

Authors' response:

We have added the above-mentioned sections to the manuscript. We have drafted a synopsis image that provides a graphical demonstration of the major findings. We will upload this synopsis image file, entitled "Synopsis Image," along with the revised manuscript.

Referee #2:

EMBOR-2025-61644V2 Review

This revised manuscript from Sharma et al. investigates the role of SIRT2 in regulating Rheb acetylation, specifically at lysine-151. The authors propose that acetylation of Rheb promotes its accumulation and consequently enhances mTOR signaling and cap-dependent translation, thereby contributing to cardiomyocyte growth and cardiac hypertrophy. In the prior version of the manuscript, several minor concerns were raised regarding insufficient description of certain data and apparent inconsistencies across a few of the experiments. These issues appear to have been satisfactorily addressed in the current revision.

Authors' response:

We sincerely thank the reviewer for their thoughtful evaluation and positive feedback on the revised manuscript. We are glad that the additional clarifications and corrections have addressed the earlier concerns regarding data description and experimental consistency. The reviewer's constructive comments have greatly helped us improve the clarity and overall quality of the manuscript.

Referee #3:

All the modifications I suggested were addressed in a satisfactory manner.

Authors' response:

We sincerely thank the reviewer for their constructive suggestions and are glad that the revisions have satisfactorily addressed all the points raised. We greatly appreciate the reviewer's feedback, which has helped improve the manuscript.

Referee #4:

Summary

This manuscript identifies Rheb as a SIRT2 substrate, showing that K151 deacetylation promotes Rheb degradation, suppresses mTORC1, and reduces protein synthesis. SIRT2-KO mice develop cardiac hypertrophy with increased synthesis, while cardiac-specific overexpression has the opposite effect. The work is technically sound with good in vivo validation, but significant conceptual issues limit its impact.

Strengths

Novel, well-validated substrate identification

Comprehensive mechanistic studies (NMR, MD simulations, functional assays)

Strong physiological relevance with multiple mouse models

Improved rigor in revision (replicates, statistics, echocardiography)

Major comment 1

1. The 4EBP1 Data Raises New Questions

Important observation: Figure S6C shows 4EBP1 levels are LOW in SIRT2-KO hearts, which is consistent with the companion manuscript showing SIRT2 stabilizes 4EBP1.

However, this creates a puzzle for your model: If 4EBP1 is low in SIRT2-KO hearts, how does increased protein synthesis occur? Your model proposes that increased Rheb → mTORC1 activation drives translation. But with less 4EBP1 available to be phosphorylated and inactivated, there should be MORE active 4EBP1 suppressing translation, not less.

Authors' response:

We thank the reviewer for this important point. The concern raised by the reviewer suggests that translational repression mediated by 4EBP1 is primarily determined by its phosphorylation state rather than its total abundance. However, the inhibitory action of 4EBP1 depends on the absolute amount of hypo-phosphorylated 4EBP1 available to sequester eIF4E, and reduced total 4EBP1 limits its repressive capacity on cap-dependent translation (PMID: 19239892, PMID: 22552098). Furthermore, studies have shown that 4EBP depletion leads to an upregulation of global protein synthesis (PMID: 27492367, PMID: 40454470). In SIRT2-KO hearts, reduced 4EBP1 protein levels are therefore likely to reduce eIF4E sequestration, causing downregulation of translational repression. Concomitantly, increased Rheb–mTORC1 signaling promotes protein synthesis through both 4EBP1-dependent and 4EBP1-independent mechanisms, including S6K1-mediated ribosomal activation and enhanced translational elongation (PMID: 19339977). Therefore, we emphasize that reduced 4EBP1-mediated repression and heightened mTORC1 activity resulted in a net upregulation in global translation.

Major comment 2

2. Minimal Experimental Integration with Companion Manuscript

You've added a Discussion paragraph but no cross-substrate experiments.

Authors' response:

We thank the reviewer for bringing this important point to our attention. While we have not conducted multiple cross-substrate experiments, we have examined 4EBP levels in Sirt2-KO hearts and observed a reduction, indicating that both Rheb and 4EBP are dysregulated under SIRT2 depletion. Furthermore, we note that, despite mechanistic differences and the use of distinct stress conditions, Rheb under glucose starvation versus 4EBP under amino acid starvation, both studies converge on a similar phenotypic outcome: SIRT2-mediated suppression of global protein synthesis under stress. This suggests that the two papers highlight complementary aspects of a shared phenotype. We have further improved the discussion in this regard. (Manuscript lines 557-577)

Major comment 3

3. Cardiac Hypertrophy Model Is Now Confusing

You cite previous work showing SIRT2 regulates NFATc2 (pathological hypertrophy), now add Rheb-mTORC1, and the companion adds 4EBP1. Which matters most? When? The Discussion lacks a clear framework explaining how these mechanisms integrate rather than listing them separately.

Authors' response:

We thank the reviewer for this insightful comment. We agree that a clearer integrative framework is needed. Pathological cardiac hypertrophy represents a complex, multifactorial remodeling process, and we have now revised the Discussion to explicitly integrate our findings within this context. Pathological cardiac hypertrophy is a complex remodeling process involving metabolic reprogramming, enhanced mTOR-dependent protein synthesis, re-expression of fetal gene programs, and myofibrillar remodeling (PMID: 35185581, 37274127, 34059001, 24663494, 16971981). These features highlight that hypertrophic growth is a highly coordinated remodeling process controlled by distinct signaling effectors acting at different biological levels. While our previous work demonstrated that SIRT2 regulates hypertrophy through NFATc2-dependent transcriptional mechanisms, the current study is specifically focused on defining a complementary SIRT2–Rheb–4E-BP1 axis that controls hypertrophic growth at the level of mTOR-dependent translational regulation. Thus, the present findings extend our earlier work by identifying an additional SIRT2-regulated pathway that contributes to pathological hypertrophy through control of protein synthesis. We have now updated the discussion part in this regard. (Manuscript lines 495-501)

Minor

E3 ligase: I understand identifying this is beyond scope, but you should explicitly acknowledge this gap and propose candidates based on Rheb biology.

Authors' response:

We thank the reviewer for emphasizing the importance of identifying or proposing candidate E3 ubiquitin ligases that may mediate Rheb degradation downstream of SIRT2. While the direct identification of the relevant E3 ligase is beyond the scope of the current study, experimental evidence in the field suggests that candidate ligases, such as the CUL4-DDB1 E3 ubiquitin ligase complex (PMID: 38862475) and the RNF152 E3 ligase (PMID: 30514904), may regulate Rheb stability under specific nutrient stresses. In the current study, we have not experimentally characterized these E3 ligases. Future work is needed to determine which ligase(s) cooperate with SIRT2 to regulate Rheb degradation and mTORC1 signaling under metabolic stress. We have

now added a statement discussing this limitation and its future perspective in the Discussion section. (Manuscript lines 550-556)

Rheb acetyltransferase: Similarly important for completing the regulatory cycle. Your glucose starvation data (SIRT2 activity increases) suggests SIRT2 is the regulated node, but this needs discussion.

Authors' response:

We thank the reviewer for the suggestion. While Rheb acetyltransferase is indeed important for the Rheb regulatory cycle, our findings establish SIRT2 as a critical regulator of Rheb stability, promoting its degradation through direct deacetylation. This positions SIRT2 as a key node in the control of Rheb-mediated signaling, particularly under conditions such as glucose starvation. While our study focuses on SIRT2-mediated Rheb deacetylation, it is important to note that acetyltransferases may also play a complementary role in this regulatory cycle. Identifying the specific acetyl-transferases responsible for Rheb acetylation and understanding how their activity intersects with SIRT2 highlights an important scope for future research. Such studies would provide a more comprehensive understanding of the dynamic acetylation–deacetylation cycle that governs Rheb function and stability. We have now included this part in the discussion. (Manuscript lines 577-583)

Minor technical issues: AMPK data needs more replicates (Fig S6A-B), echocardiography needs clearer sample size reporting, exercise experiments need proper controls (WT sedentary vs. running).

Authors' response:

We thank the reviewer for bringing these issues to our attention. We have included 6 animals per group in the AMPK data; the quantification for these is presented in Figure EV6B.

Sample size reporting is clearly indicated in the respective figure legends throughout the manuscript

We thank the reviewer for bringing this to our notice. We have now revised the panel markings across different figures related to the exercise experiment. We have now included full words "Sedentary" and running" along with properly highlighting WT and *Sirt2*-KO in each panel

More comments from referee 4:

These manuscripts should be published separately, not as integrated companion papers. While both describe SIRT2 regulating translation, they actually represent distinct stress-response pathways rather than a unified mechanism. Zi et al. show amino acid starvation increases SIRT2 protein levels (via ISR-mediated translation), which then stabilizes 4EBP1. Sharma et al. show glucose starvation increases SIRT2 activity (not protein), which degrades Rheb and inhibits mTORC1. These are fundamentally different regulatory contexts engaging SIRT2 through different mechanisms.

More importantly, there's no experimental evidence that the pathways work together. Despite reviewer requests, neither manuscript measures both substrates under the same conditions, tests substrate competition, or performs epistasis experiments. The "integration" exists only in the Discussion paragraphs, using nearly identical language about PTM enzymes having multiple substrates. The in vivo data from Sharma et al. actually suggest Rheb regulation dominates-SIRT2-KO hearts have high Rheb, low 4EBP1, yet still show increased protein synthesis and hypertrophy. If 4EBP1 stabilization were equally important, low 4EBP1 levels should provide some protection, but they don't.

The one finding that could meaningfully link them-Zi et al. Figure R8 showing K69 acetylation increases 4EBP1's phosphorylation competence-is buried in revision responses rather than integrated into either narrative. This could explain why both upstream (Rheb) and downstream (4EBP1) regulation matter, but neither manuscript develops this insight.

I would recommend publishing these as independent studies, each acknowledging the other exists but not claiming functional integration that isn't experimentally demonstrated. For true back-to-back publication, you would need at minimum: one experiment testing both substrates under the same stress conditions, an epistasis test showing whether 4EBP1 overexpression rescues SIRT2-KO phenotypes, and a unified model explaining when and where each mechanism predominates. Without this, they're two separate papers about SIRT2, not a coordinated study of multi-substrate regulation.

Authors' response:

We sincerely thank the reviewer for bringing this concern to our attention. We believe that our response to major comment 1 and major comment 2 raised by reviewer 4, along with the extended discussion added in the revised manuscript, will address these concerns. We would like to clarify that we are not experimentally claiming that these papers are real back-to-back papers. We will be open to an editorial decision regarding the independent publication of this paper, while just linking it to the other companion paper as per suggested by reviewer 4.

Dr. Nagalingam Sundaresan
Indian Institute of Science
Microbiology and Cell Biology
SB 02
Division of Biological Sciences
Bengaluru, Karnataka 560012
India

Dear Dr. Sundaresan,

I am very pleased to accept your manuscript for publication in the next available issue of EMBO reports. Thank you for your contribution to our journal.

Your manuscript will be processed for publication by EMBO Press. It will be copy edited and you will receive page proofs prior to publication. Please note that you will be contacted by Springer Nature Author Services to complete licensing and payment information.

You may qualify for financial assistance for your publication charges - either via a Springer Nature fully open access agreement or an EMBO initiative. Check your eligibility: https://link.springer.com/journal/44319/how-to-publish-with-us

Should you be planning a Press Release on your article, please get in contact with embo_production@springernature.com as early as possible in order to coordinate publication and release dates.

If you have any questions, please do not hesitate to contact the Editorial Office. Thank you for your contribution to EMBO Reports.

Best regards,
Esther

Esther Schnapp, PhD
Senior Editor
EMBO reports

>>> Please note that it is EMBO Reports policy for the transcript of the editorial process (containing referee reports and your response letter) to be published as an online supplement to each paper. If you do NOT want this, you will need to inform the Editorial Office via email immediately. More information is available here: https://link.springer.com/partners/embo-press/editorial-policies#Peer%20review

EMBO Press Author Checklist

Corresponding Author Name: Nagalingam Ravi Sundaresan	
Journal Submitted to: EMBO Reports	
Manuscript Number: EMBOR-2025-61642V1	

USEFUL LINKS FOR COMPLETING THIS FORM
The EMBO Journal - Author Guidelines
EMBO Reports - Author Guidelines
Molecular Systems Biology - Author Guidelines
EMBO Molecular Medicine - Author Guidelines

Reporting Checklist for Life Science Articles (updated January
This checklist is adapted from Materials Design Analysis Reporting (MDAR) Checklist for Authors. MDAR establishes a minimum set of requirements in transparent reporting in the life sciences (see Statement of Task: 10.31222/osf.io/9sm4x). Please follow the journal's guidelines in preparing your
Please note that a copy of this checklist will be published alongside your article.

Abridged guidelines for figures
1. Data
The data shown in figures should satisfy the following conditions:
- the data were obtained and processed according to the field's best practice and are presented to reflect the results of the experiments in an accurate and unbiased manner.
- ideally, figure panels should include only measurements that are directly comparable to each other and obtained with the same assay.
- plots include clearly labeled error bars for independent experiments and sample sizes. Unless justified, error bars should not be shown for technical
- if n<5, the individual data points from each experiment should be plotted. Any statistical test employed should be justified.
- Source Data should be included to report the data underlying figures according to the guidelines set out in the authorship guidelines on Data

2. Captions
Each figure caption should contain the following information, for each panel where they are relevant:
- a specification of the experimental system investigated (eg cell line, species name).
- the assay(s) and method(s) used to carry out the reported observations and measurements.
- an explicit mention of the biological and chemical entity(ies) that are being measured.
- an explicit mention of the biological and chemical entity(ies) that are altered/varied/perturbed in a controlled manner.
- the exact sample size (n) for each experimental group/condition, given as a number, not a range;
- a description of the sample collection allowing the reader to understand whether the samples represent technical or biological replicates (including how many animals, litters, cultures, etc.).
- a statement of how many times the experiment shown was independently replicated in the laboratory.
- definitions of statistical methods and measures:
  - common tests, such as t-test (please specify whether paired vs. unpaired), simple χ2 tests, Wilcoxon and Mann-Whitney tests, can be unambiguously identified by name only, but more complex techniques should be described in the methods section;
  - are tests one-sided or two-sided?
  - are there adjustments for multiple comparisons?
  - exact statistical test results, e.g., P values = x but not P values < x;
  - definition of 'center values' as median or average;
  - definition of error bars as s.d. or s.e.m.

Please complete ALL of the questions below.
Select "Not Applicable" only when the requested information is not relevant for your study.

Materials

Newly Created Materials	Information included in the manuscript?	In which section is the information available? (Reagents and Tools Table, Materials and Methods, Figures, Data Availability Section)
New materials and reagents need to be available; do any restrictions apply?	Not Applicable	

Antibodies	Information included in the manuscript?	In which section is the information available? (Reagents and Tools Table, Materials and Methods, Figures, Data Availability Section)
For **antibodies** provide the following information:<br>- Commercial antibodies: RRID (if possible) or supplier name, catalogue number and or/clone number<br>- Non-commercial: RRID or citation	Yes	Reagents and Tool Table

DNA and RNA sequences	Information included in the manuscript?	In which section is the information available? (Reagents and Tools Table, Materials and Methods, Figures, Data Availability Section)
**Short novel DNA or RNA including primers, probes**: provide the sequences.	Yes	Reagents and Tool Table

Cell materials	Information included in the manuscript?	In which section is the information available? (Reagents and Tools Table, Materials and Methods, Figures, Data Availability Section)
**Cell lines:** Provide species information, strain. Provide accession number in repository **OR** supplier name, catalog number, clone number, and/**OR** RRID.	Yes	Reagent and Tool Table
**Primary cultures:** Provide species, strain, sex of origin, genetic modification status.	Yes	Materials and Methods
Report if the cell lines were recently **authenticated** (e.g., by STR profiling) and tested for mycoplasma contamination.	Not Applicable	

Experimental animals	Information included in the manuscript?	In which section is the information available? (Reagents and Tools Table, Materials and Methods, Figures, Data Availability Section)
**Laboratory animals or Model organisms:** Provide species, strain, sex, age, genetic modification status. Provide accession number in repository **OR** supplier name, catalog number, clone number, **OR** RRID.	Yes	Reagents and Tool Table, Materials and Methods
**Animal observed in or captured from the field:** Provide species, sex, and age where possible.	Not Applicable	
Please detail **housing and husbandry conditions**.	Yes	Materials and Methods

Plants and microbes	Information included in the manuscript?	In which section is the information available? (Reagents and Tools Table, Materials and Methods, Figures, Data Availability Section)
**Plants:** provide species and strain, ecotype and cultivar where relevant, unique accession number if available, and source (including location for collected wild specimens).	Not Applicable	
**Microbes:** provide species and strain, unique accession number if available, and source.	Not Applicable	

Human research participants	Information included in the manuscript?	In which section is the information available? (Reagents and Tools Table, Materials and Methods, Figures, Data Availability Section)
If collected and within the bounds of privacy constraints report on age, sex and gender or ethnicity for all study participants.	Not Applicable	

Core facilities	Information included in the manuscript?	In which section is the information available? (Reagents and Tools Table, Materials and Methods, Figures, Data Availability Section)
If your work benefited from core facilities, was their service mentioned in the acknowledgments section?	Yes	Materials and Methods

Design

Study protocol	Information included in the manuscript?	In which section is the information available? (Reagents and Tools Table, Materials and Methods, Figures, Data Availability Section)
If study protocol has been **pre-registered, provide DOI in the manuscript**. For clinical trials, provide the trial registration number **OR** cite DOI.	Not Applicable	
Report the **clinical trial registration number** (at ClinicalTrials.gov or equivalent), where applicable.	Not Applicable	

Laboratory protocol	Information included in the manuscript?	In which section is the information available? (Reagents and Tools Table, Materials and Methods, Figures, Data Availability Section)
Provide DOI OR other citation details if **external detailed step-by-step protocols** are available.	Yes	Materials and Methods

Experimental study design and statistics	Information included in the manuscript?	In which section is the information available? (Reagents and Tools Table, Materials and Methods, Figures, Data Availability Section)
Include a statement about **sample size** estimate even if no statistical methods were used.	Yes	Figure legends, Figures, and Source data section
Were any steps taken to minimize the effects of subjective bias when allocating animals/samples to treatment (e.g. **randomization procedure**)? If yes, have they been described?	Not Applicable	
Include a statement about **blinding** even if no blinding was done.	Not Applicable	
Describe **inclusion/exclusion criteria** if samples or animals were excluded from the analysis. Were the criteria pre-established?<br><br>If sample or data points were omitted from analysis, report if this was due to attrition or intentional exclusion and provide justification.	Not Applicable	
For every figure, are **statistical tests** justified as appropriate? Do the data meet the assumptions of the tests (e.g., normal distribution)? Describe any methods used to assess it. Is there an estimate of variation within each group of data? Is the variance similar between the groups that are being statistically compared?	Yes	Figures,Figure legends, Materials and Methods,Source data section

Sample definition and in-laboratory replication	Information included in the manuscript?	In which section is the information available? (Reagents and Tools Table, Materials and Methods, Figures, Data Availability Section)
In the figure legends: state number of times the experiment was **replicated** in laboratory.	Yes	Figures, Figure legends
In the figure legends: define whether data describe **technical or biological replicates**.	Yes	Figure Legends, Source data section

Ethics

Ethics	Information included in the manuscript?	In which section is the information available? (Reagents and Tools Table, Materials and Methods, Figures, Data Availability Section)
Studies involving **human participants**: State details of **authority granting ethics approval** (IRB or equivalent committee(s), provide reference number for approval.	Not Applicable	
Studies involving **human participants**: Include a statement confirming that **informed consent** was obtained from all subjects and that the experiments conformed to the principles set out in the WMA Declaration of Helsinki and the Department of Health and Human Services Belmont Report.	Not Applicable	
Studies involving **human participants:** For publication of **patient photos**, include a statement confirming that consent to publish was obtained.	Not Applicable	
Studies involving experimental **animals**: State details of **authority granting ethics approval** (IRB or equivalent committee(s), provide reference number for approval. Include a statement of compliance with ethical regulations.	Yes	Materials and Methods
Studies involving **specimen and field samples:** State if relevant **permits** obtained, provide details of authority approving study; if none were required, explain why.	Not Applicable	

Dual Use Research of Concern (DURC)	Information included in the manuscript?	In which section is the information available? (Reagents and Tools Table, Materials and Methods, Figures, Data Availability Section)
Could your study fall under dual use research restrictions? Please check biosecurity documents and list of **select agents and toxins** (CDC): https://www.selectagents.gov/sat/list.htm	Not Applicable	
If you used a select agent, is the security level of the lab appropriate and reported in the manuscript?	Not Applicable	
If a study is subject to dual use research of concern regulations, is the name of the **authority granting approval and reference number** for the regulatory approval provided in the manuscript?	Not Applicable	

Reporting

The MDAR framework recommends adoption of discipline-specific guidelines, established and endorsed through community initiatives. Journals have their own policy about requiring specific guidelines and recommendations to complement MDAR.

Adherence to community standards	Information included in the manuscript?	In which section is the information available? (Reagents and Tools Table, Materials and Methods, Figures, Data Availability Section)
State if relevant guidelines or checklists (e.g., **ICMJE, MIBBI, ARRIVE, PRISMA**) have been followed or provided.	Yes	Reagents and Tools Table, Materials and Methods
For **tumor marker prognostic studies,** we recommend that you follow the **REMARK** reporting guidelines (see link list at top right). See author guidelines, under 'Reporting Guidelines'. Please confirm you have followed these guidelines.	Not Applicable	
For **phase II and III randomized controlled trials**, please refer to the **CONSORT** flow diagram (see link list at top right) and submit the CONSORT checklist (see link list at top right) with your submission. See author guidelines, under 'Reporting Guidelines'. Please confirm you have submitted this list.	Not Applicable	

Data Availability

Data availability	Information included in the manuscript?	In which section is the information available? (Reagents and Tools Table, Materials and Methods, Figures, Data Availability Section)
Have **primary datasets** been deposited according to the journal's guidelines (see 'Data Deposition' section) and the respective accession numbers provided in the Data Availability Section?	Not Applicable	
Were **human clinical and genomic datasets** deposited in a public access-controlled repository in accordance to ethical obligations to the patients and to the applicable consent agreement?	Not Applicable	
Are **computational models** that are central and integral to a study available without restrictions in a machine-readable form? Were the relevant accession numbers or links provided?	Not Applicable	
If publicly available data were reused, provide the respective **data citations in the reference list**.	Yes	Main Manuscript text, Reference list, Figure
